# Global and regional prevalence of vitamin D deficiency in population-based studies from 2000 to 2022: A pooled analysis of 7.9 million participants

**DOI:** 10.3389/fnut.2023.1070808

**Published:** 2023-03-17

**Authors:** Aiyong Cui, Tiansong Zhang, Peilong Xiao, Zhiqiang Fan, Hu Wang, Yan Zhuang

**Affiliations:** ^1^Department of Orthopaedics, Honghui Hospital, Xi'an Jiaotong University, Xi'an, China; ^2^Jing'an District Hospital of Traditional Chinese Medicine, Shanghai, China; ^3^Department of Orthopaedics, The Fifth Affiliated Hospital of Sun Yat-Sen University, Zhuhai, Guangdong, China; ^4^Department of Pelvic and Acetabular Surgery, Honghui Hospital, Xi'an Jiaotong University, Xi'an, China

**Keywords:** global, prevalence, vitamin D, deficiency, population-based studies

## Abstract

**Background:**

Vitamin D deficiency causes the bone hypomineralization disorder osteomalacia in humans and is associated with many non-skeletal disorders. We aim to estimate the global and regional prevalence of vitamin D deficiency in people aged 1 year or older from 2000 to 2022.

**Methods:**

We systematically searched Web of Science, PubMed (MEDLINE), Embase, Scopus, and Google databases on December 31, 2021, and updated them on August 20, 2022, without language and time restrictions. Meanwhile, we identified references of relevant system reviews and eligible articles and included the latest and unpublished data from the National Health and Nutrition Examination Survey (NHANES, 2015–2016 and 2017–2018) database. The studies investigating the prevalence of vitamin D deficiency in population-based studies were included. A standardized data extraction form was used to collect information from eligible studies. We used a random-effects meta-analysis to estimate the global and regional prevalence of vitamin D deficiency. We stratified meta-analyses by latitude, season, six WHO regions, the World Bank income groups, gender, and age groups. This study was registered with PROSPERO (CRD42021292586).

**Findings:**

Out of 67,340 records searched, 308 studies with 7,947,359 participants from 81 countries were eligible for this study, 202 (7,634,261 participants), 284 (1,475,339 participants), and 165 (561,978 participants) studies for the prevalence of serum 25(OH)D <30, <50, and <75 nmol/L, respectively. We found that globally, 15.7% (95% CrI 13.7–17.8), 47.9% (95% CrI 44.9–50.9), and 76·6% (95% CrI 74.0–79.1) of participants had serum 25-hydroxyvitamin D levels less than 30, 50, and 75 nmol/l, respectively; the prevalence slightly decreased from 2000–2010 to 2011–2022, but it was still at a high level; people living in high latitude areas had a higher prevalence; the prevalence in winter-spring was 1.7 (95% CrI 1.4–2.0) times that in summer-autumn; the Eastern Mediterranean region and Lower-middle-income countries had a higher prevalence; females were vulnerable to vitamin D deficiency; gender, sampling frame, detection assays, sampling region, time of data collection, season, and other factors contributed to heterogeneity between the included studies.

**Interpretation:**

Globally, vitamin D deficiency remained prevalent from 2000 to 2022. The high prevalence of vitamin D deficiency would increase the global burden of disease. Therefore, governments, policymakers, health workers, and individuals should attach importance to the high prevalence of vitamin D deficiency and take its prevention as a public health priority.

**Systematic review registration:**

https://www.crd.york.ac.uk/prospero/display_record.php?ID=CRD42021292586, PROSPERO CRD42021292586.

## Introduction

Vitamin D, a fat-soluble prehormone, is essential to maintain calcium, phosphorus homeostasis, and others (1). Vitamin D deficiency has been considered a global health issue because it can cause the bone hypomineralization disorder osteomalacia in humans (2). Existing studies have reported that vitamin D deficiency is also associated with infectious diseases [severe coronavirus disease 2019 (COVID-19) and upper respiratory tract infection] (3–5). Many studies also reported the links between vitamin D deficiency and other diseases, for instance, muscle weakness, multiple sclerosis, diabetes, hypertension, metabolic syndrome, cancers, autoimmune diseases, cardiovascular disease, and hip or vertebrae fracture in later life (6–9). Vitamin D can be obtained from skin exposure to ultraviolet B radiation (UVB), dietary intake, and foods fortified with vitamin D. Studies show that the risk factors that are responsible for vitamin D deficiency include air pollution, latitude, season, the use of sunscreen, sedentary jobs, diet and others (10).

The circulating concentration of total serum 25(OH)D was most often used to determine the status of vitamin D in the body. However, there are different consensus recommendations on the cut-off definition of vitamin D deficiency. In consideration of the risk of metabolic bone disease, the US Institute of Medicine (IOM) agreed that the value of serum 25(OH)D <30 nmol/L (12 ng/mL) is considered as vitamin D deficiency, 30–50 nmol/L (12–20 ng/mL) is considered as vitamin D insufficiency, and ≥50 nmol/L (20 ng/mL) is considered as being sufficient (11). In the light of increasing levels of circulating parathyroid hormone the Endocrine Society designated the threshold of vitamin D deficiency at ≤ 50 nmol/L (20 ng/mL), the threshold of vitamin D insufficiency at 50–75 nmol/L (20–30 ng/mL), the threshold of vitamin D normal at ≥75 nmol/L (30 ng/mL) (12, 13). Nevertheless, all guidelines have reached the consensus that the value of serum 25(OH)D <25 or 30 nmol/l (10–12 ng/ml) should be averted at all ages (14).

Some previous systematic reviews and meta-analyses have investigated the mean value of serum 25(OH)D in the population worldwide from 1990 to 2011 and the prevalence of vitamin D deficiency in Europe, Africa, and Asia (15–21). In recent years, new studies examining the prevalence of vitamin D deficiency have dramatically increased in each country all over the world. However, there is still no study estimating the global and regional prevalence of vitamin D deficiency. In addition, it is time-consuming, laborious, and expensive to carry out a representative large-scale study on the global population. Therefore, we pooled published data to investigate the global and regional prevalence of vitamin D deficiency in the general population from 2000 to 2022.

## Methods

This meta-analysis was conducted according to the Preferred Reporting Items for Systematic Reviews and Meta-analyses (PRISMA) reporting guideline and the Meta-analysis of Observational Studies in Epidemiology (MOOSE) reporting guideline (22, 23). The protocol of the study was preregistered in the PROSPERO database (CRD42021292586).

### Search and select strategy

To comprehensively describe the global and regional prevalence of vitamin D deficiency, AC and PX, under the guidance of the doctor (TZ) of clinical evidence-based medicine, systematically searched Web of Science, PubMed (MEDLINE), Embase, Scopus, and Google databases. The databases were searched on December 31, 2021, and updated them on August 20, 2022, without language and time restrictions. The details of the retrieval strategy are uploaded in Appendix 1 (Supplementary Tables 1–4). We also tried to retrieve relevant gray articles Google database, but no eligible articles were found. Additionally, we manually searched references of relevant system reviews and eligible articles. Furthermore, we included the latest and unpublished data from the National Health and Nutrition Examination Survey (NHANES, 20015–2016 and 20017–2018) database, (24) which was a nationally representative nutrition survey in the United States.

In order to identify eligible studies, firstly, we used endnote to remove duplicates. Then according to inclusion and exclusion criteria, AC and PX scanned the title and abstract independently. Finally, AC and PX browsed the full text of the remaining articles. All inconsistencies encountered in the selection of studies were resolved through consensus reached by all authors.

### Inclusion and exclusion criteria

Inclusion and exclusion criteria were developed with reference to related published research (17, 18, 25, 26). The aim of our study is to assess the global and regional prevalence of vitamin D deficiency in population aged 1 year or older from 2000 to 2022. Therefore, we included studies reporting the prevalence of vitamin D deficiency in population aged 1 year or older, studies collecting data after 2000, and studies including a sample size of more than 50 (aiming to avoid biases caused by small samples). In articles that used the same data source, only the study with the richest data was included. The included studies are cross-sectional and longitudinal studies.

We excluded the studies that were done in specific educational and occupational population. For instance, athletes have special needs for vitamin D and factory workers often spend less time outdoors. The studies that were done in hospital-based populations were excluded in sight of them suffering more complications. The case-control studies were excluded. Infants, pregnant women, newborn babies, and new mothers were excluded due to their special needs for vitamin D. Although we did not specifically exclude non-English literature, the studies included in the final analysis were all in English.

### Data analysis

A predefined and standardized data extraction form was used to collect information. Two investigators (AC, PX) independently extracted relevant information from the included articles, including first author, publication year, country of investigation, latitude, season, region of study location (African Region, Region of the Americas, South-East Asia Region, European Region, Eastern Mediterranean Region, and Western Pacific region, as designated by WHO; High-income, Upper-middle-income, Lower-middle-income, and Low-income countries, as designated by the World Bank for 2019–2020), sampling method, time of venous blood sample collection, sample size, number of female participants, average age and age range of participants, detection assays, type of study, Sampling location (national, community, or schools et al.), and the prevalence of the value of serum 25(OH)D <25, <30, <50, and <75 nmol/L. In articles where the value of serum 25(OH)D was presented in ng/ml, we converted 1 ng/ml to 2.494 nmol/L by multiplying 2.494. The included articles defined different thresholds for vitamin D deficiency, including <25, 30, or 50 nmol/L. The IOM summarized that the value of serum 25(OH)D <30 nmol/L was associated with an increased risk of symptomatic osteomalacia or rickets (11). Meanwhile, all guidelines suggested that the value of serum 25(OH)D <25 or 30 nmol/l should be avoided at all ages (27). Therefore, for convenience, we merged data on the value of serum 25(OH)D <25 and 30 nmol/L and presented them as <30 nmol/L.

To assess the quality of included articles, AC and PX used the risk of bias tool in prevalence studies developed by Hoy D et al., which includes 10 items and a summary assessment (28). Items 1 to 4 assess the selection and non-response bias of the study, items 5 to 9 assess the domain of measurement bias, and item 10 assesses bias related to the analysis. The summary assessment evaluates the overall risk of study bias based on items 1 to 10.

When encountering inconsistencies in the process of extracting data, the third author (YZ) would re-extracted the data. Then the differences were solved by a consensus reached by all authors.

### Statistical analysis

Considering the anticipated high heterogeneity of the observational studies, AC and PX, under the guidance of the doctor (HW) whose major is clinical epidemiology and evidence-based medicine, employed random-effects meta-analysis to generate estimates. To stabilize variances, study data were transformed using the Freeman-Tukey double arcsine transformation (29). We did meta-analyses of established cutoffs for serum 25-hydroxyvitamin D levels <30, 50, and 75 nmol/l. We stratified meta-analyses by latitude, season, six WHO regions, the World Bank income groups, gender, time of data collection, and age groups. To verify the reliability of the results, we merged the data of vitamin D <30 mmol/L extracted by age stratification and conducted a meta-analysis again. Finally, we found that the results were consistent (Supplementary Figures 1.1, 1.2). All results were generated with an accompanying 95% confidence interval (CI). A 2-sided *P-*value <0.05 was considered statistically significant in all analyses.

The heterogeneity was assessed using the *I*^2^ statistic. To explore the source of heterogeneity, we firstly conducted subgroup analyses by latitude, season, age, gender, six WHO regions, and the World Bank income groups. Then we performed sensitivity analyses to explore the impact of the individual study on the overall effects. Univariate meta-regression models (The covariates including latitude, publication year, world bank income groups, WHO regions, age, the proportion of women, diagnostic method, time of data collection, type of study, sampling method, population, and risk of bias) were further used to assess the associations between the characteristics of studies and the pooled prevalence. We qualitatively and quantitatively detected publication bias using funnel plots and the Egger linear regression test, respectively (30). We used the R software for meta-analyses.

## Results

**Figure 1:**
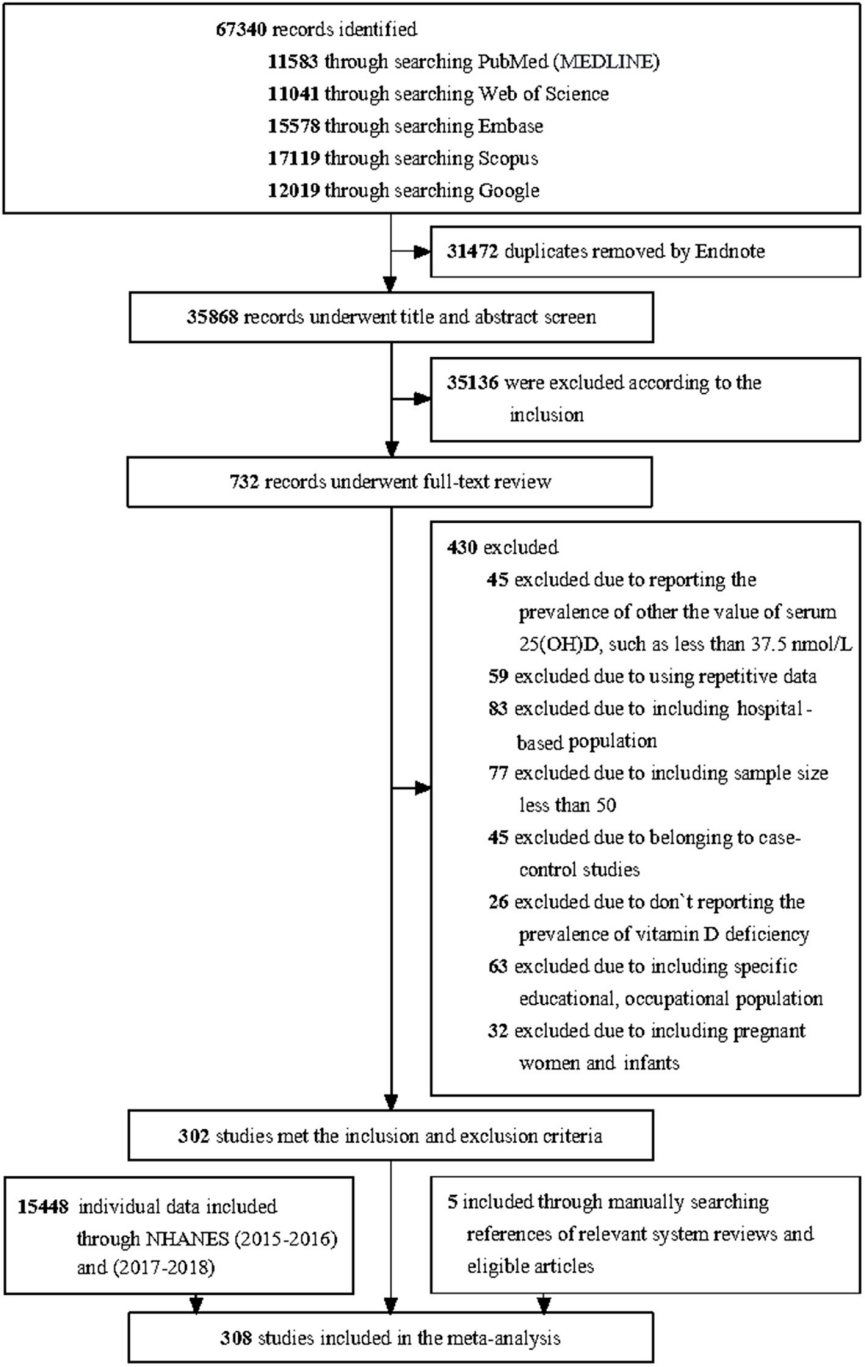
PRISMA flow diagram.

We retrieved a total of 67,340 articles through searching databases, of which 31,472 duplicates were excluded by endnote. 35,136 articles were excluded by browsing the titles and abstracts. 732 remaining articles underwent full-text review, of which 302 peer-reviewed articles met the inclusion criteria. Meanwhile, five studies by manually searching references of relevant system reviews and eligible articles were included. We regard the data on vitamin D from NHANES (2015–2016 and 2017–2018) as an article. Finally, the equivalent of 308 articles was included in this study. The process of article selection is shown in Figure 1.

The eligible 308 articles (Appendix 15) with 7.9 million participants were from 81 countries and six WHO regions all over the world. The number of studies varied from one in Uganda to 23 in India. The sample size of the included studies varied from 52 to 1,316,390. 93 (7,238,477 participants) out of 308 studies were from the Europe region, 51 (206,470 participants) from the region of the Americas, 14 (7,088 participants) from the African region, 64 (85,770 participants) from the Eastern Mediterranean region, 29 (28,780 participants) from the South-East Asia region, and 57 (380,774 participants) from the Western Pacific region. 35 studies were conducted at 0–20°north latitude, 130 at 20–40°north latitude, 92 at 40–60°north latitude, 11 at 60–80°north latitude, 8 at 0–20°south latitude, and 32 at 20–40°south latitude. There were no study at 40–60°and 60–80°south latitude. 169 of 305 studies were published in High-income countries, 70 in Upper middle-income countries, 64 in lower-middle-income countries, and five in Low-income countries. 21 studies reported the seasonal prevalence of vitamin D in detail. The number of females was more than that of males in most of the included studies. Essentially, all studies were cross-sectional. 163 literature used random sampling, and 145 literature didn‘t use random sampling. 68 articles were national surveys, 211 were community surveys, 16 were school surveys and 13 were health check-up surveys. The characteristics and data of the included studies are shown in Appendix 2 (Supplementary Tables 5–7). Ten studies were at high risk of bias, 121 were at moderate risk of bias, and 177 were at low risk of bias [Appendix 3 (Supplementary Table 8)].

The global prevalence was estimated based on 308 studies with 7.9 million participants worldwide. The global prevalence of serum 25(OH)D <30 nmol/L was 15.7% (95% CI 13.7–17.8) [Table 1 and Appendix 4 (Supplementary Figure 1)]; 47.9% (95% CI 44.9–50.9) for serum 25(OH)D <50 nmol/L [Table 2 and Appendix 4 (Supplementary Figure 2)]; 76.6% (95% CI 74.0–79.1) for serum 25(OH)D <75 nmol/L [Table 3 and Appendix 4 (Supplementary Figure 3)]. The prevalence slightly decreased from 2000–2010 to 2011–2022 [Tables 1–3 and Appendix 5 (Supplementary Figures 4–9)]. The prevalence of serum 25(OH)D <30 nmol/L was 17.6% (95% CI 14.4–19.9) in 2000–2010 and decreased to 14.1% (95% CI 11.6–16.7) in 2011–2022.

**Table 1:** The prevalence of serum 25-hydroxyvitamin D levels <30 nmol/l.

	**No. of studies**	**No. of countries**	**Total**	**Events**	**Prevalence**	**95% CI**
**Less than 30 nmol/L**						
All	202	60	7,634,261	2,497,047	15.7%	(13.7–17.8)%
**Latitude**						
60–80°N	10	6	13,717	1,068	10.4%	(5.8–16.1)%
40–60°N	81	29	7,051,984	2,423,716	14.9%	(11.9–18.1)%
20–40°N	79	24	495,169	68,601	23.1%	(18.9–27.6)%
0–20°N	12	7	44,250	1,721	5.9%	(2.9–9.9)%
0–20°S	2	2	2,533	40	1.5%	(1.0–2.0)%
20–40°S	18	6	26,608	1,901	10.5%	(6.8–14.8)%
**World bank income groups**				
HIC	123	33	7,488,490	2,473,442	15.1%	(11.2–19.1)%
UMIC	30	17	98,905	7989	10.2%	(6.8–14.0)%
LMIC	31	9	46,670	15,587	26.7%	(19.2–34.5)%
LIC	1	1	196	29	14.8%	(10.1–20.1)%
**Age**						
18	62	31	143,893	24,702	14.9%	(12.8–17.5)%
19–44	25	19	3,789,069	1,514,233	18.2%	(11.7–25.7)%
45–64	16	13	1,677,407	526,218	13.8%	(8.6–19.9)%
65	26	16	1,017,288	303,154	15.3%	(11.6–19.4)%
**Gender**						
Males	57	34	3,434,089	1,235,560	13.6%	(10.6–16.9)%
Females	57	34	3,607,750	1,189,949	17.8%	(13.9–21.9)%
**Data collection time**						
2000–2010	97	39	7,208,689	2,444,924	17.6%	(14.4–19.9)%
2011–2022	105	49	425,572	52,123	14.1%	(11.6–16.7)%

NO., number; CI, confidence interval; HIC, High-income countries; UMIC, Upper-middle-income countries; LMIC, Lower-middle-income countries; LIC, Low-income countries; Events, number of people with vitamin D deficiency.

**Table 2:** The prevalence of serum 25-hydroxyvitamin D levels <50 nmol/l.

	**No. of studies**	**No. of countries**	**Total**	**Events**	**Prevalence**	**95% CI**
**Less than 50 nmol/L**						
All	284	79	1,475,339	753,756	44.7%	(44.7–50.8)%
**Latitude**						
60–80°N	10	5	13,532	5,197	57.4%	(44.8–69.6)%
40–60°N	89	34	633,299	329,116	45.3%	(40.6–50.0)%
20–40°N	118	31	685,482	376,281	60.2%	(55.5–64.9)%
0–20°N	36	16	64,187	17,458	34.1%	(25.9–42.8)%
0–20°S	8	4	6,890	1,315	18.2%	(11.2–26.5)%
20–40°S	23	6	71,949	24,389	37.4%	(30.5–44.6)%
**World bank income groups**						
HIC	159	39	1,230,487	650,409	49.2%	(45.6–52.8)%
UMIC	60	20	210,918	84,348	38.2%	(32.2–44.2)%
LMIC	51	17	34,523	18,690	56.0%	(47.0–64.7)%
LIC	5	3	918	420	54.2%	(19.0–87.2)%
**Age**						
18	93	50	155,486	69,984	48.5%	(42.5–54.5)%
19–44	33	21	40,053	15,528	47.8%	(38.8–56.9)%
45–64	16	13	23,435	10,099	46.0%	(35.6–56.7)%
65	33	19	25,821	10,987	46.3%	(38.6–54.1)%
**Gender**						
Males	90	45	361,131	192,737	45.3%	(40.4–50.2)%
Females	90	45	421,692	233,540	53.3%	(48.1–58.5)%
**Data collection time**						
2000–2010	133	55	892,087	462,726	48.7%	(44.4–53.0)%
2011–2022	151	64	584,759	291,141	46.9%	(42.8–51.2)%

NO., number; CI, confidence interval; HIC, High-income countries; UMIC, Upper-middle-income countries; LMIC, Lower-middle-income countries; LIC, Low-income countries; Events, number of people with vitamin D deficiency.

**Table 3:** The prevalence of serum 25-hydroxyvitamin D levels <75 nmol/l.

	**No. of studies**	**No. of countries**	**Total**	**Events**	**Prevalence**	**95% CI**
**Less than 75 nmol/L**						
All	165	65	561,978	443,815	76.6%	(74.1–79.1)%
**Latitude**						
60–80°N	6	4	9,836	7,394	83.2%	(70.5–92.9)%
40–60°N	56	26	121,565	88,571	74.6%	(70.5–78.5)%
20–40°N	66	23	345,450	288,729	84.9%	(81.3–88.1)%
0–20°N	14	11	16,302	9,733	52.6%	(40.1–65.1)%
0–20°S	7	3	6,457	4,214	66.2%	(63.7–68.7)%
20–40°S	16	6	62,368	45,174	76.0%	(72.1–79.7)%
**World bank income groups**						
HIC	88	34	434,195	351,804	77.6%	(74.8–80.4)%
UMIC	42	17	110,418	78,870	74.1%	(32.2–44.2)%
LMIC	29	13	18,570	13,808	77.1%	(67.2–85.6)%
LIC	1	1	302	113	37.4%	(68.5–79.3)%
**Age**						
18	51	36	76,474	59,818	78.6%	(73.8–83.1)%
19–44	21	17	30,923	22,732	75.5%	(67.7–82.3)%
45–64	9	7	19,865	14,489	68.5%	(56.7–79.3)%
65	21	15	23,594	17,252	74.0%	(66.0–81.2)%
**Gender**						
Males	54	33	82,393	65,477	77.4%	(72.9–81.6)%
Females	54	33	102,846	84,346	81.5%	(77.5–85.2)%
**Data collection time**						
2000–2010	78	40	377,509	303,119	77.1%	(73.6–80.5)%
2011–2022	87	46	185,976	141,476	75.9%	(72.2–80.0)%

NO., number; CI, confidence interval; HIC, High-income countries; UMIC, Upper-middle-income countries; LMIC, Lower-middle-income countries; LIC, Low-income countries; Events, number of people with vitamin D deficiency.

**Figure 2:**
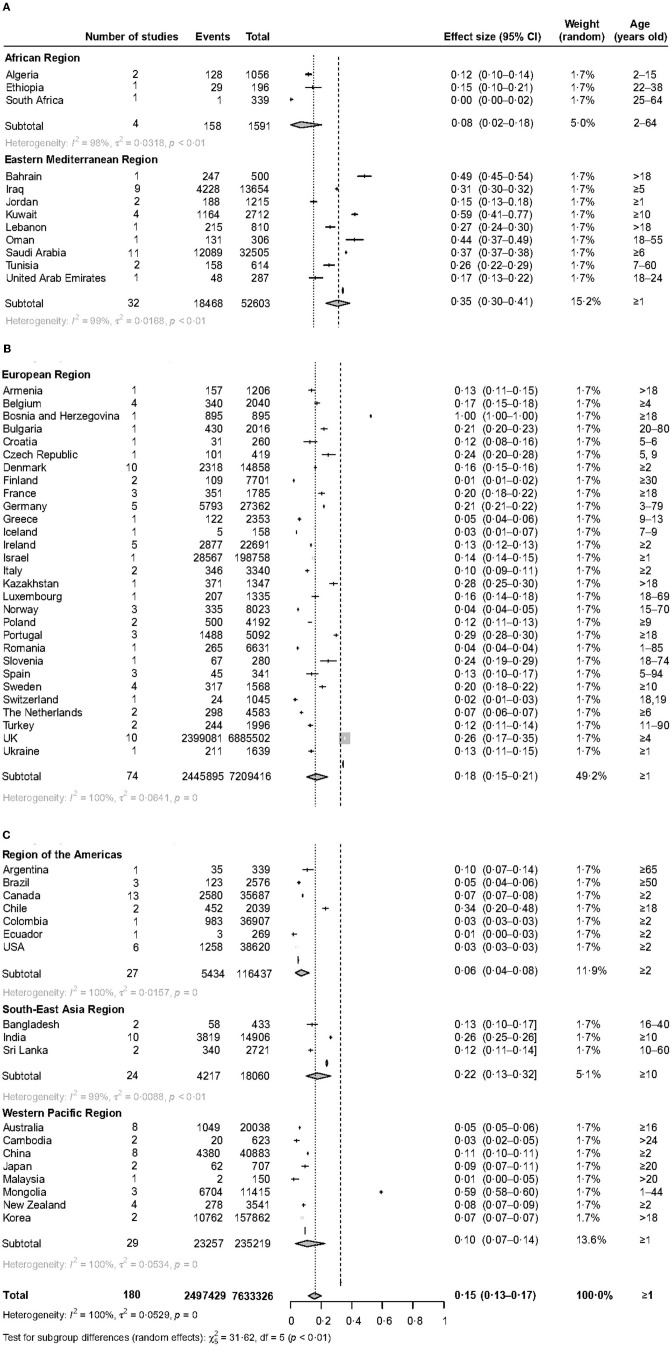
The forest plot of the global prevalence of serum 25 (OH)D <30 nmol/L. This figure consists of three parts **(A–C)**.

**Figure 3:**
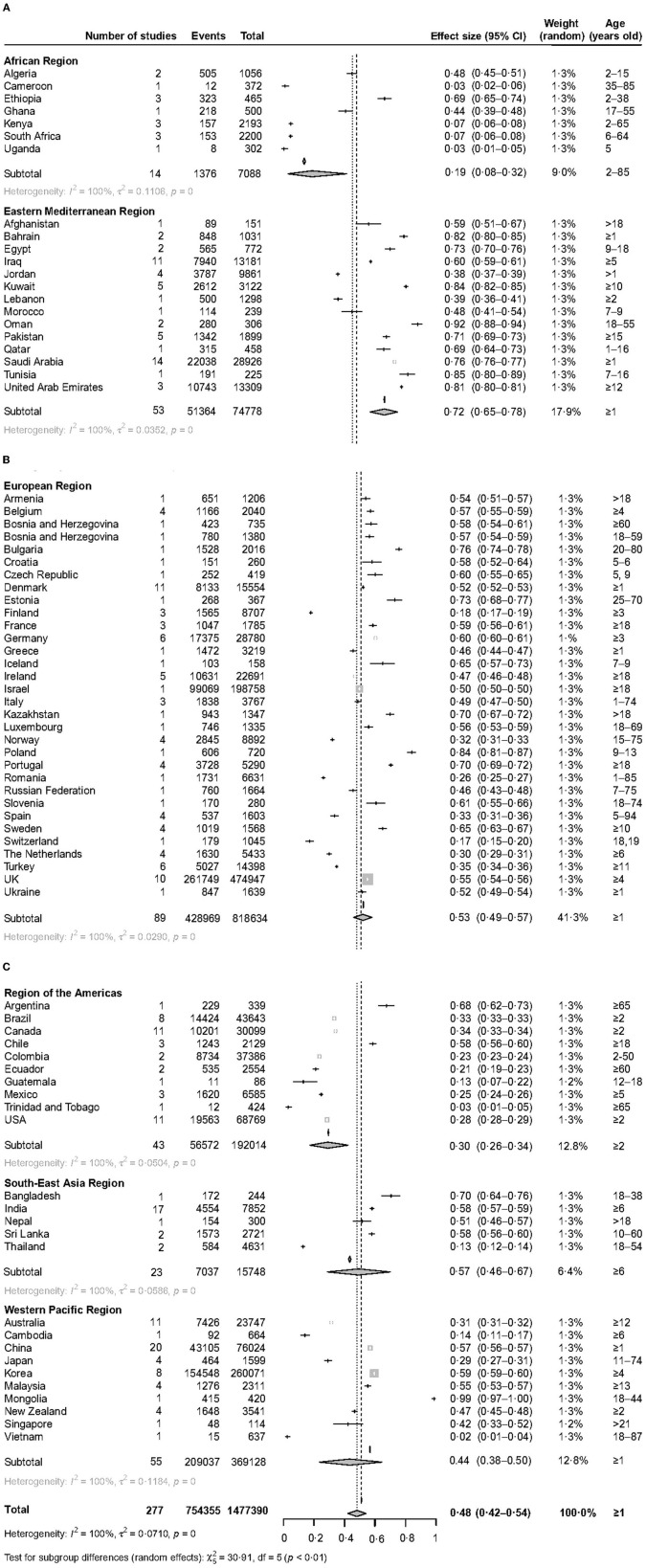
The forest plot of the global prevalence of serum 25 (OH)D <50 nmol/L. This figure consists of three parts **(A–C)**.

The prevalence of vitamin D deficiency varied widely in the six WHO regions. The prevalence of serum 25(OH)D <30 nmol/L varied from 5.5% (95% CI 3.5–7.8) in the Region of the Americas to 35.2% (95% CI 29.6–41.0) in the Eastern Mediterranean Region (Figure 2). The prevalence of serum 25(OH)D <50 nmol/L varied from 18.9% (95% CI 8.4–32.3) in the African Region to 71.8% (95% CI 65.4–77.8) in the Eastern Mediterranean Region (Figure 3). The prevalence of serum 25(OH)D <75 nmol/L was also the lowest in the African Region [55.3% (95% CI 40.0–70.1)] and the highest in the Eastern Mediterranean Region [85.1% (95% CI 78.3–90.8)] [Appendix 6 (Supplementary Figure 10)].

Latitude seems to be significantly associated with prevalence. People living in low latitudes had a lower prevalence (Tables 1–3), 5.9% (95% CI 2.9–9.9) and 1.5% (95% CI 1.0–2.0) for serum 25(OH)D <30 nmol/L at 0–20 degrees north and south latitude, respectively. Higher latitude may be a risk factor for a high prevalence of vitamin D deficiency. 14.9% (95% CI 11.9–18.1) of people living at 40–60 degrees north latitude had serum 25(OH)D <30 nmol/L; 57.4% (95% CI 44.8–69.6) of people living at 60–80 degrees north latitude had serum 25(OH)D <50 nmol/L [Appendix 7 (Supplementary Figures 11–13)].

World Bank income groups also exhibit great differences in terms of vitamin D deficiency, as is shown in Tables 1–3 and Appendix 8 (Supplementary Figures 14–16). Lower-middle-income countries had a higher prevalence; 26.7% (95% CI 19.2–34.5) for serum 25(OH)D <30 nmol/L and 56.0% (95% CI 47.0–64.7) for serum 25(OH)D <50 nmol/L. Significantly lower rates were found in Upper-middle-income countries; 10.2% (95% CI 6.8–14.0) for serum 25(OH)D <30 nmol/L and 38.2% (95% CI 32.2–44.2) for serum 25(OH)D <50 nmol/L. Compared to lower-middle-income countries, high-income countries also had a relatively low prevalence; 15.1% (95% CI 11.2–19.1) for serum 25(OH)D <30 nmol/L and 49.2% (95% CI 45.6–52.8) for serum 25(OH)D <50 nmol/L.

The prevalence of the <18, 19–44, 45–64, and > 65 years old population was estimated to predict the high-risk groups [Tables 1–3 and Appendix 9 (Supplementary Figures 17–28)]. People aged <18 had a higher prevalence of serum 25(OH)D <50 nmol/L [48.5% (95% CI 42.5–54.5)]. The prevalence was lower in people aged 45–64; 13.8% (95% CI 8.6–19.9) and 46.0% (95% CI 35.6–56.7) for serum 25(OH)D <30 and <50 nmol/L, respectively.

Vitamin D deficiency was more common in females than in males [Tables 1–3 and Appendix 10 (Supplementary Figures 29–37)]. 17.8% (95% CI 13.9–21.9) of females and 13.6% (95% CI 10.6–16.9) of males had serum 25(OH)D <30 nmol/L. Women suffered from serum 25(OH)D <30 nmol/L 1.3 (95% CI 1.2–1.4) times more than men; women suffered from serum 25(OH)D <50 nmol/L 1.2 (95% CI 1.1–1.3) times more than men. Vitamin deficiency was also more common in winter-spring than in summer-autumn. People had serum 25(OH)D <50 nmol/L 1.7 (95% CrI 1.4–2.0) times in winter-spring more than in summer-autumn (Appendix 11 [Supplementary Figure 38]).

The meta-regression analyses show that latitude, female (%), sampling frame, detection assays, season, and so on collectively contributed to heterogeneity [Appendix 12 (Supplementary Tables 9, 10)], which may affect the reliability of the results. Sensitivity analyses demonstrate that the estimates are stable when any one of the included studies is deleted [Appendix 13 (Supplementary Figures 39–41)]. The funnel plots indicate that there was no significant publication bias [Appendix 14 (Supplementary Figures 42–44)].

## Discussion

This systematic review and meta-analysis comprehensively investigated the global and regional prevalence of vitamin D deficiency in people aged 1 year or older from 2000 to 2022. The findings of this study emphasize that the prevalence of vitamin D deficiency decreased slightly from 2000–2010 to 2011–2022, but remained at a high level; latitude and season are important risk factors for vitamin D deficiency; more people living in the Eastern Mediterranean region and Lower-middle-income countries suffered from vitamin D deficiency; females were more vulnerable to vitamin D deficiency; the data of prevalence of vitamin D deficiency are lacking in low-income countries.

Our study assessed the prevalence of serum 25(OH)D <30 nmol/L based on the data from the included study. All guidelines and reviews agreed that the value of serum 25(OH)D <25 or 30 nmol/L is harmful to human health (13, 31, 32). Our study shows that the global prevalence of serum 25(OH)D <30 nmol/L was 15.7% between 2000 and 2022. The result is similar to that of a study published in the New England Journal (2). From the findings of our study, vitamin D deficiency is a huge challenge facing the world. It is self-evident that vitamin D plays an important role in bone metabolism. Most studies showed that the value of serum 25(OH)D lower than 30 nmol/L (12 ng/mL) is associated with rickets in children (33, 34). In addition, previous association studies reported that vitamin D deficiency is also an important risk factor for SARS-CoV-2, weak muscle strength, cardiovascular disease, multiple sclerosis, upper respiratory tract infection, certain cancers, and other disease (4, 35–37). In turn, these complications may increase the burden of global disease. Therefore, preventing vitamin D deficiency is a very important and urgent public health issue. It is time to deal with it. Firstly, citizens should be made aware of the harm of vitamin D deficiency to health. Secondly, food fortification is the most efficient and cheapest way to increase population 25OHD levels, the government should take corresponding measures to deal with it (38, 39). Thirdly, citizens should be informed of the strategies for preventing vitamin D deficiency, including avoiding prolonged breastfeeding without vitamin D supplementation, increasing the time of exposure to the sun appropriately, and maintaining fatty fish intake and cod liver oil, and others (7, 12). Fourthly, health care professionals should follow recommendations for supplementation.

The prevalence of vitamin D deficiency within different WHO regions showed a high variation. The prevalence was the highest in the Eastern Mediterranean region. 58.9% of the Kuwait population aged 10 suffered serum 25 (OH)D levels <30 nmol/L. 44.3% of Oman's population aged 18–55 suffered serum 25 (OH)D levels <30 nmol/L. The IOF Committee of Scientific Advisors (CSA) Nutrition Working Group drew the same conclusion (40). Although there is plenty of sunshine in the Middle East, why is there a serious vitamin D deficiency there? This can be largely explained by cultural practices. People there are used to wearing veils, which leads to limited sun exposure (15, 41). However, vitamin D in the body mainly comes from skin exposure to UVB. Other factors that may be involved include insufficient vitamin D supplementation, skin pigmentation, and socioeconomic status and others (33). Conversely, the Region of the Americas had the lowest prevalence. 3.0% of the American population aged 2 and older suffered from serum 25 (OH)D levels <30 nmol/L. This may be attributed to high socioeconomic status, milk and food fortified with vitamin D, growing awareness of health damage caused by vitamin D deficiency, and measures that are taken by the government (42). However, some countries in this region still had a high prevalence of vitamin D deficiency, which should be recognized, for instance, 34.0% of the Chile population aged 18 and older suffered from serum 25 (OH)D levels <30 nmol/L. The prevalence was also low in African Region, with 8.0 and 18.9% for serum 25 (OH)D levels <30 and 50 nmol/L, respectively. However, this finding may be unreliable, because there were only fourteen studies with 7088 participants in Africa. Other possible reasons include ample sunshine in Africa (17) and more young people than the elderly in included studies. In short, large population-based studies are needed to explore it in the future. European Region had a wealth of data (93 studies with 7,238,477 participants), so we can get a more reliable conclusion. The prevalence of serum 25 (OH)D levels <30 and 50 nmol/L was 18.0% and 53.0% in the European region. It can be seen from this that the Europeans suffered from a higher prevalence of vitamin D deficiency (19, 39). This finding can be well exemplified by the British population, with 25.9% and 55.3% for serum 25 (OH)D levels <30 and 50 nmol/L, respectively. A high prevalence of vitamin D deficiency also existed in South-East Asia and Western Pacific Regions. 22.0% of the South-East Asia population and 10.0% of the Western Pacific population had serum 25 (OH)D levels <30 nmol/L. These similar findings were recorded in some recent studies (18, 43, 44). It can be seen from the above that the WHO regions' populations were suffering from a high prevalence of vitamin D deficiency.

From the prevalence results at different latitudes, people living in high latitudes are more likely to suffer from vitamin D deficiency on the whole, which may be caused by insufficient sunshine. However, the prevalence was the highest at 20–40°north latitude; 23.10, 60.20, and 84.90% for serum 25(OH)D <30, <50, and <75 nmol/L, respectively. We speculate that the reason for the abnormally high results is that most of the countries in the Eastern Mediterranean region are located at 20–40°north latitude. As can be seen from the previous analysis, the Eastern Mediterranean region had a higher prevalence, for instance, people living in Saudi Arabia (37.4%), Bahrain (49.4%), and Iraq (31.1%) suffered from serum 25(OH)D <30 nmol/L. Therefore, more attention should be paid to the vitamin status of residents in high-latitude areas.

Other important factors affecting the prevalence were observed in our study. The prevalence of females was higher than that of males. Therefore, some measures should be taken to prevent vitamin D deficiency in women, such as vitamin D supplementation. The prevalence in Winter–spring was higher than that in Summer–autumn. Therefore, in winter, people susceptible to vitamin D deficiency should take vitamin D supplements to prevent vitamin D deficiency. The age-specific prevalence results demonstrate that the prevalence of serum 25(OH)D <30 nmol/L was 14.9, 18.2, 13.8, and 15.3% among people aged <18, 19–44, 45–64, and 65 years and older, respectively. It can be concluded that the prevalence of serum 25(OH)D <30 nmol/L was more pronounced among adults aged 19 to 44 years old. It is speculated that these people supplement less vitamin D but need more vitamin D owing to an increase in muscle mass and fiber and others (31). Socioeconomic status may be associated with vitamin D deficiency. The prevalence of serum 25(OH)D <30 nmol/L within the World Bank income groups varied from 10.2% in Upper-middle-income countries to 26.7% in Lower-middle-income countries. Therefore, these individuals living in Lower-middle-income and Low-income countries deserve more care. The different detection assays are also the main factors affecting the prevalence, so the studies using the same detection assay will improve the comparability of results. Other important risk factors not discussed in our study include genetic traits, dietary intake, clothing style, time spent outdoors, skin pigmentation, and others (5, 10, 34, 43). In general, there is great heterogeneity between observational studies. The risk factors discussed above were the cause of heterogeneity.

Although we included a large of studies and used better statistical methods, there are still some limitations in our study. Firstly, considering the heterogeneity between included studies, our estimate of the global vitamin D deficiency may not be very accurate. Secondly, we cannot accurately estimate the prevalence of vitamin D deficiency in each country owing to the limitations of the number of studies and statistical models. Thirdly, the included studies used different detection assays, which may affect the stability of the results. Fourthly, we extracted data of the prevalence of the value of serum 25(OH)D <25, <30, <50, and <75 nmol/L, data of other value of serum 25(OH)D was not extracted, such as serum 25(OH)D <37.5 nmol/L. We extracted latitude data from the included study that clearly reported latitude data. When estimating prevalence of serum 25(OH)D by latitude, the study that did not report latitude data was excluded. Fifthly, due to the limitations of the original data, we cannot estimate the prevalence differences between races. However, we call for political action to protect these ethnic risk groups (45, 46).

## Conclusion

Globally, vitamin D deficiency remained prevalent from 2000 to 2022. The high prevalence of vitamin D deficiency would increase the global burden of disease. Therefore, governments, policymakers, health care workers, and individuals should pay attention to the high prevalence of vitamin D deficiency and take its prevention as a public health priority.

## Data availability statement

The original contributions presented in the study are included in the article/Supplementary material, further inquiries can be directed to the corresponding authors.

## Author contributions

AC, TZ, PX, and YZ planned the study, designed the methods, and did the statistical analyses. AC and PX contributed to the literature review. AC, PX, and HW extracted data. AC prepared the first draft of the manuscript with important contributions from TZ, PX, and YZ. All authors interpreted the results, commented on drafts of the manuscript, and approved the final version.
