# Supplementary material for: Global and regional prevalence of vitamin D deficiency in population-based studies from 2000 to 2022: A pooled analysis of 7.9 million participants
Source: Front Nutr. 2023 Mar 17;10:1070808. doi: 10.3389/fnut.2023.1070808 (PMC10064807; doi:10.3389/fnut.2023.1070808)
Supplement: Supplementary file 1 [file Data_Sheet_1.PDF]

Supplementary appendix

Table of Contents

Appendix 1: Data sources
Supplementary Table 1 Search strategy in PubMed (MEDLINE)
Supplementary Table 2 Search strategy in Web of Science
Supplementary Table 3 Search strategy in EMBASE
Supplementary Table 4 Search strategy in SCOPUS
Appendix 2: The extracted information of the included studies
Supplementary Table 5 The characteristics of the included studies
Supplementary Table 6 The extracted data of the included studies
Supplementary Table 7 The extracted data that are used to distinguish different studies
Appendix 3: The assessment of the quality of included studies
Supplementary Table 8 The summaries of quality assessment of included studies
Appendix 4: The global prevalence of vitamin D deficiency
Supplementary Figure 1 The global prevalence of serum 25(OH)D < 30 nmol/L
Supplementary Figure 2 The global prevalence of serum 25(OH)D < 50 nmol/L
Supplementary Figure 3 The global prevalence of serum 25(OH)D < 75 nmol/L
Appendix 5: The global prevalence of vitamin D deficiency from 2000–2010 to 2011–2022
Supplementary figure 4 The global prevalence of serum 25(OH)D < 30 nmol/L in 2000–2010
Supplementary figure 5 The global prevalence of serum 25(OH)D < 30 nmol/L in 2011–2022
Supplementary figure 6 The global prevalence of serum 25(OH)D < 50 nmol/L in 2000–2010
Supplementary figure 7 The global prevalence of serum 25(OH)D < 50 nmol/L in 2011–2022
Supplementary figure 8 The global prevalence of serum 25(OH)D < 75 nmol/L in 2000–2010
Supplementary figure 9 The global prevalence of serum 25(OH)D < 75 nmol/L in 2011–2022
Appendix 6: The prevalence of serum 25(OH)D < 75 nmol/L by six WHO regions
Supplementary figure 10 The prevalence of serum 25(OH)D < 75 nmol/L by six WHO regions
Appendix 7: The prevalence of vitamin D deficiency by latitude
Supplementary figure 11 The prevalence of serum 25(OH)D < 30 nmol/L by latitude
Supplementary figure 12 The prevalence of serum 25(OH)D < 50 nmol/L by latitude
Supplementary figure 13 The prevalence of serum 25(OH)D < 75 nmol/L by latitude
Appendix 8: The prevalence of vitamin D deficiency by World Bank income groups
Supplementary figure 14 The prevalence of serum 25(OH)D < 30 nmol/L by World Bank income groups

Supplementary figure 15 The prevalence of serum 25(OH)D < 50 nmol/L by World Bank income groups ....

Supplementary figure 16 The prevalence of serum 25(OH)D < 75 nmol/L by World Bank income groups ....

Appendix 9: The prevalence of vitamin D deficiency by age ....

Supplementary figure 17 The prevalence of serum 25(OH)D < 30 nmol/L among people aged < 18..

Supplementary figure 18 The prevalence of serum 25(OH)D < 30 nmol/L among people aged 19-44

Supplementary figure 19 The prevalence of serum 25(OH)D < 30 nmol/L among people aged 45-64

Supplementary figure 20 The prevalence of serum 25(OH)D < 30 nmol/L among people aged > 65..

Supplementary figure 21 The prevalence of serum 25(OH)D < 50 nmol/L among people aged < 18..

Supplementary figure 22 The prevalence of serum 25(OH)D < 50 nmol/L among people aged 19-44

Supplementary figure 23 The prevalence of serum 25(OH)D < 50 nmol/L among people aged 45-64

Supplementary figure 24 The prevalence of serum 25(OH)D < 50 nmol/L among people aged > 65..

Supplementary figure 25 The prevalence of serum 25(OH)D < 75 nmol/L among people aged < 18..

Supplementary figure 26 The prevalence of serum 25(OH)D < 75 nmol/L among people aged 19-44

Supplementary figure 27 The prevalence of serum 25(OH)D < 75 nmol/L among people aged 45-64

Supplementary figure 28 The prevalence of serum 25(OH)D < 75 nmol/L among people aged > 65..

Appendix 10: The prevalence of vitamin D deficiency by gender ....

Supplementary figure 29 The prevalence of serum 25(OH)D < 30 nmol/L among males ....

Supplementary figure 30 The prevalence of serum 25(OH)D < 30 nmol/L among females ....

Supplementary figure 31 The prevalence of serum 25(OH)D < 50 nmol/L among males ....

Supplementary figure 32 The prevalence of serum 25(OH)D < 50 nmol/L among females ....

Supplementary figure 33 The prevalence of serum 25(OH)D < 75 nmol/L among males ....

Supplementary figure 34 The prevalence of serum 25(OH)D < 75 nmol/L among females ....

Supplementary figure 35 The Risk Ratio of serum 25(OH)D < 30 nmol/L among females and males

Supplementary figure 36 The Risk Ratio of serum 25(OH)D < 50 nmol/L among females and males

Supplementary figure 37 The Risk Ratio of serum 25(OH)D < 75 nmol/L among females and males

Appendix 11: the Risk Ratio of prevalence in Winter-spring and Summer-autumn ....

Supplementary figure 38 The Risk Ratio of serum 25(OH)D < 50 nmol/L in Winter-spring and Summer-autumn ....

Appendix 12: Meta-regression analyses ....

Supplementary Table 9 The univariate meta-regression analyses on the prevalence of serum 25-hydroxyvitamin D levels less than 30 nmol/l. ....

Supplementary Table 10 The univariate meta-regression analyses on the prevalence of serum 25-hydroxyvitamin D levels less than 50 nmol/l. ....

Appendix 13: Sensitivity analyses...

Supplementary figure 39 The sensitivity analyses for the prevalence of serum 25-hydroxyvitamin D levels less than 30 nmol/l ....

Supplementary figure 40 The sensitivity analyses for the prevalence of serum 25-hydroxyvitamin D levels less than 50 nmol/l ....

Supplementary figure 41 The sensitivity analyses for the prevalence of serum 25-hydroxyvitamin D levels less than 30 nmol/l ....

Appendix 14: Publication bias analyses...

Supplementary figure 42 The publication bias analyses for studies on the prevalence of serum 25-hydroxyvitamin D levels less than 30 nmol/l..

Supplementary figure 43 The publication bias analyses for studies on the prevalence of serum 25-hydroxyvitamin D levels less than 50 nmol/l..

Supplementary figure 44 The publication bias analyses for studies on the prevalence of serum 25-hydroxyvitamin D levels less than 75 nmol/l..

Appendix 15: References...

Appendix 1: Data sources

Supplementary Table 1 Search strategy in PubMed (MEDLINE)

Search Number	Query	Results
20	((((((((Vitamin D[MeSH Terms]) OR (Vitamin D[Title/Abstract])) OR ((Vitamin D Deficiency[MeSH Terms]) OR (Vitamin D Deficiency[Title/Abstract]))) OR ((Hypovitaminosis D[MeSH Terms]) OR (Hypovitaminosis D[Title/Abstract]))) OR ((cholecalciferols[MeSH Terms]) OR (cholecalciferols[Title/Abstract]))) OR ((ergocalciferols[MeSH Terms]) OR (ergocalciferols[Title/Abstract]))) OR ((calcifediol[MeSH Terms]) OR (calcifediol[Title/Abstract]))) AND (((((prevalence[MeSH Terms]) OR (prevalence[Title/Abstract])) OR ((incidence[MeSH Terms]) OR (incidence[Title/Abstract]))) OR ((epidemiolog*[MeSH Terms]) OR (epidemiolog*[Title/Abstract]))) OR (status[Title/Abstract]))) AND ((((((national[MeSH Terms]) OR (national[Title/Abstract])) OR ((community[MeSH Terms]) OR (community[Title/Abstract]))) OR ((population[MeSH Terms]) OR (population[Title/Abstract]))) OR ((cross-sectional[MeSH Terms]) OR (cross-sectional[Title/Abstract]))) OR (longitudinal[Title/Abstract]))	11,583
19	((((((((Vitamin D[MeSH Terms]) OR (Vitamin D[Title/Abstract])) OR ((Vitamin D Deficiency[MeSH Terms]) OR (Vitamin D Deficiency[Title/Abstract]))) OR ((Hypovitaminosis D[MeSH Terms]) OR (Hypovitaminosis D[Title/Abstract]))) OR ((cholecalciferols[MeSH Terms]) OR (cholecalciferols[Title/Abstract]))) OR ((ergocalciferols[MeSH Terms]) OR (ergocalciferols[Title/Abstract]))) OR ((calcifediol[MeSH Terms]) OR (calcifediol[Title/Abstract]))) AND (((((prevalence[MeSH Terms]) OR (prevalence[Title/Abstract])) OR ((incidence[MeSH Terms]) OR (incidence[Title/Abstract]))) OR ((epidemiolog*[MeSH Terms]) OR (epidemiolog*[Title/Abstract]))) OR (status[Title/Abstract]))) AND ((((((national[MeSH Terms]) OR (national[Title/Abstract])) OR ((community[MeSH Terms]) OR (community[Title/Abstract]))) OR ((population[MeSH Terms]) OR (population[Title/Abstract]))) OR ((cross-sectional[MeSH Terms]) OR (cross-sectional[Title/Abstract]))) OR (longitudinal[Title/Abstract]))	15,505
18	(((((national[MeSH Terms]) OR (national[Title/Abstract])) OR ((community[MeSH Terms]) OR (community[Title/Abstract]))) OR ((population[MeSH Terms]) OR (population[Title/Abstract]))) OR ((cross-sectional[MeSH Terms]) OR (cross-sectional[Title/Abstract]))) OR (longitudinal[Title/Abstract])	3,395,372
17	longitudinal[Title/Abstract]	286,792

16	(cross-sectional[MeSH Terms]) OR (cross-sectional[Title/Abstract])	565,324
15	(population[MeSH Terms]) OR (population[Title/Abstract])	1,883,947
14	(community[MeSH Terms]) OR (community[Title/Abstract])	611,068
13	(national[MeSH Terms]) OR (national[Title/Abstract])	680,052
12	((((prevalence[MeSH Terms]) OR (prevalence[Title/Abstract])) OR ((incidence[MeSH Terms]) OR (incidence[Title/Abstract]))) OR ((epidemiolog*[MeSH Terms]) OR (epidemiolog*[Title/Abstract]))) OR (status[Title/Abstract])	8,659,165
11	status[Title/Abstract]	967,313
10	(epidemiolog*[MeSH Terms]) OR (epidemiolog*[Title/Abstract])	7,721,829
9	(incidence[MeSH Terms]) OR (incidence[Title/Abstract])	938,803
8	(prevalence[MeSH Terms]) OR (prevalence[Title/Abstract])	796,069
7	((((((Vitamin D[MeSH Terms]) OR (Vitamin D[Title/Abstract])) OR ((Vitamin D Deficiency[MeSH Terms]) OR (Vitamin D Deficiency[Title/Abstract]))) OR ((Hypovitaminosis D[MeSH Terms]) OR (Hypovitaminosis D[Title/Abstract]))) OR ((cholecalciferols[MeSH Terms]) OR (cholecalciferols[Title/Abstract]))) OR ((ergocalciferols[MeSH Terms]) OR (ergocalciferols[Title/Abstract]))) OR ((calcifediol[MeSH Terms]) OR (calcifediol[Title/Abstract]))	101,597
6	(calcifediol[MeSH Terms]) OR (calcifediol[Title/Abstract])	4,364
5	(ergocalciferols[MeSH Terms]) OR (ergocalciferols[Title/Abstract])	4,476
4	(cholecalciferols[MeSH Terms]) OR (cholecalciferols[Title/Abstract])	27,781
3	(Hypovitaminosis D[MeSH Terms]) OR (Hypovitaminosis D[Title/Abstract])	15,925
2	(Vitamin D Deficiency[MeSH Terms]) OR (Vitamin D Deficiency[Title/Abstract])	35,912
1	(Vitamin D[MeSH Terms]) OR (Vitamin D[Title/Abstract])	92,490

Supplementary Table 2 Search strategy in Web of Science

Search Number	Query	Results
1	(AB=(Vitamin D)) OR TS=(Vitamin D)	92,974
2	(AB=(Vitamin D Deficiency)) OR TS=(Vitamin D Deficiency)	27,700
3	(AB=(Hypovitaminosis D)) OR TS=(Hypovitaminosis D)	3,564
4	(AB=(cholecalciferols)) OR TS=(cholecalciferols)	5
5	(AB=(ergocalciferols)) OR TS=(ergocalciferols)	18
6	(AB=(ergocalciferols)) OR TS=(ergocalciferols)	18
7	#6 OR #5 OR #4 OR #3 OR #2 OR #1	93,094
8	(AB=(prevalence)) OR TS=(prevalence)	920,097
9	(AB=(incidence)) OR TS=(incidence)	680,231
10	(AB=(epidemiolog*)) OR TS=(epidemiolog*)	507,082
11	(AB=(status)) OR TS=(status)	1,116,503
12	#8 OR #9 OR #10 OR #11	2,797,367
13	(AB=(national)) OR TS=(national)	953,970
14	(AB=(community)) OR TS=(community)	1,213,434
15	(AB=(population)) OR TS=(population)	2,377,879
16	(AB=(cross-sectional)) OR TS=(cross-sectional)	446,346
17	(AB=(longitudinal)) OR TS=(longitudinal)	420,143
18	#13 OR #14 OR #15 OR #16 OR #17	4,695,385
19	#7 AND #12 AND #18	11,041

Supplementary Table 3 Search strategy in EMBASE

No.	Query	Results
#4	#1 AND #2 AND #3	15,578
#3	national:ab,ti OR community:ab,ti OR population:ab,ti OR cross-sectional:ab,ti OR longitudinal:ab,ti	6,837,925
#2	prevalence:ab,ti OR incidence:ab,ti OR epidemiolog*:ab,ti OR status:ab,ti	9,482,367
#1	Vitamin D:ab,ti OR Vitamin D Deficiency:ab,ti OR Hypovitaminosis D:ab,ti OR cholecalciferols:ab,ti OR ergocalciferols:ab,ti OR calcifediol:ab,ti	12,467

Supplementary Table 4 Search strategy in SCOPUS

No.	Query	Results
4	((TITLE-ABS-KEY (prevalence) OR TITLE-ABS-KEY (incidence) OR TITLE-ABS-KEY (epidemi*) OR TITLE-ABS-KEY (status))) AND ((TITLE-ABS-KEY (Vitamin D) OR TITLE-ABS-KEY (Vitamin D Deficiency) OR TITLE-ABS-KEY (Hypovitaminosis D) OR TITLE-ABS-KEY (cholecalciferols) OR TITLE-ABS-KEY (ergocalciferols) OR TITLE-ABS-KEY (calcifediol))) AND ((TITLE-ABS-KEY (national) OR TITLE-ABS-KEY (community) OR TITLE-ABS-KEY (population) OR TITLE-ABS-KEY (cross-sectional) OR TITLE-ABS-KEY (longitudinal)))	17,119
3	(TITLE-ABS-KEY (national) OR TITLE-ABS-KEY (community) OR TITLE-ABS-KEY (population) OR TITLE-ABS-KEY (cross-sectional) OR TITLE-ABS-KEY (longitudinal))	7,894,568
2	(TITLE-ABS-KEY (Vitamin D) OR TITLE-ABS-KEY (Vitamin D Deficiency) OR TITLE-ABS-KEY (Hypovitaminosis D) OR TITLE-ABS-KEY (cholecalciferols) OR TITLE-ABS-KEY (ergocalciferols) OR TITLE-ABS-KEY (calcifediol))	14,378
1	(TITLE-ABS-KEY (prevalence) OR TITLE-ABS-KEY (incidence) OR TITLE-ABS-KEY (epidemi*) OR TITLE-ABS-KEY (status))	10,278,936

Appendix 2: The extracted information from the included studies

Supplementary Table 5 The characteristics of the included studies

Order	First author	latitude	Year of publication	Country	World Bank income groups	WHO regions	Diagnostic method	Type of study	Sampling method	Study area	population
**1**	Abdulrahman MA	35	2022	Iraq	UMIC	EMR	ELISA	CSS	random sampling	Community	Adults
**1**	Abdulrahman MA	35	2022	Iraq	UMIC	EMR	ELISA	CSS	random sampling	Community	Adults
**1**	Abdulrahman MA	35	2022	Iraq	UMIC	EMR	ELISA	CSS	random sampling	Community	Adolescents
**2**	Abiaka C	21	2013	Oman	HIC	EMR	RIA	CSS	no random sampling	Community	Adults
**3**	Abu-Samak MS	32	2019	Jordan	UMIC	EMR	ECLIA	CSS	no random sampling	Community	Adults
**4**	Al Hayek S	34	2018	Lebanon	UMIC	EMR	ELISA	CSS	no random sampling	Community	Adults
**5**	Al Shaikh A	24	2020	Saudi Arabia	HIC	EMR	ECLIA	CSS	no random sampling	National	Adolescents
**6**	Al Shaikh AM	24	2016	Saudi Arabia	HIC	EMR	ECLIA	CSS	no random sampling	National	Adolescents
**6**	Al Shaikh AM	24	2016	Saudi Arabia	HIC	EMR	ECLIA	CSS	no random sampling	National	Adolescents
**7**	Al Zarooni AAR	23	2019	United Arab Emirates	HIC	EMR	ECLIA	CSS	no random sampling	National	Adults

**8**	Al-Daghri NM	24	2015	Saudi Arabia	HIC	EMR	ECLIA	CSS	no random sampling	Community	Adults
**8**	Al-Daghri NM	24	2015	Saudi Arabia	HIC	EMR	ECLIA	CSS	no random sampling	Community	Adolescents
**9**	Al-Daghri NM	24	2016	Saudi Arabia	HIC	EMR	ECLIA	CSS	no random sampling	Community	Adults
**9**	Al-Daghri NM	24	2016	Saudi Arabia	HIC	EMR	ECLIA	CSS	no random sampling	Community	Adolescents
**10**	Al-Daghri NM	24	2021	Saudi Arabia	HIC	EMR	ECLIA	CSS	random sampling	National	Adults
**10**	Al-Daghri NM	24	2021	Saudi Arabia	HIC	EMR	ECLIA	CSS	random sampling	National	Adolescents
**11**	Aleteng Q	31	2017	China	UMIC	WPR	CLIA	CSS	consecutive sampling	Community	Older
**12**	AlFaris NA	24	2019	Saudi Arabia	HIC	EMR	ECLIA	CSS	no random sampling	Community	Adults
**13**	Alkerwi A	50	2015	Luxembourg	HIC	ER	ECLIA	CSS	random sampling	National	Adults
**14**	Al-Kindi MK	21	2011	Oman	HIC	EMR	ECLIA	CSS	no random sampling	Community	Adults
**15**	Alloubani A	24	2019	Saudi Arabia	HIC	EMR	CLIA	CSS	no random sampling	Community	Adults
**16**	AlQuaiz AM	24	2018	Saudi Arabia	HIC	EMR	ECLIA	CSS	no random sampling	National	Adults
**17**	Al-Saleh Y	24	2015	Saudi Arabia	HIC	EMR	CLIA	CSS	no random sampling	Community	Adolescents

**18**	Al-Taiar A	29	2018	Kuwait	HIC	EMR	LC-MS/MS	CSS	a stratified multistage cluster random sampling	Community	Adolescents
**19**	Alyahya K	29	2014	Kuwait	HIC	EMR	CLIA	CSS	random sampling	Community	Adolescents
**20**	Alyahya KO	29	2020	Kuwait	HIC	EMR	ECLIA	CSS	no random sampling	Community	Adults
**21**	Andersen R	55	2005	Denmark, Finland, Ireland, Poland.	HIC	ER	HPLC	CSS	random sampling	Community	Elderly
**21**	Andersen R	55	2005	Denmark, Finland, Ireland, Poland.	HIC	ER	HPLC	CSS	random sampling	Community	Adolescents
**22**	Andersen R	55	2008	Denmark	HIC	ER	HPLC	CSS	no random sampling	Community	Children
**22**	Andersen R	55	2008	Denmark	HIC	ER	HPLC	CSS	no random sampling	Community	Adults
**23**	Andersen R	55	2013	Denmark	HIC	ER	HPLC	CSS	random sampling	Community	Elderly
**23**	Andersen R	55	2013	Denmark	HIC	ER	HPLC	CSS	random sampling	Community	Children
**24**	Andersen S	69	2013	Denmark	HIC	ER	RIA	CSS	random sampling	Community	Adults

**25**	Arabi A	34	2021	Lebanon	UMIC	EMR	ECLIA	CSS	random sampling	Community	Adults
**26**	Ardawi MS	22	2011	Saudi Arabia	HIC	EMR	CLIA	CSS	random sampling	Community	Adults
**27**	Ardawi MS	22	2012	Saudi Arabia	HIC	EMR	ECLIA	CSS	no random sampling	Community	Adults
**28**	Arnljots R	58	2017	Sweden	HIC	ER	ECLIA	CSS	random sampling	Community	Older
**29**	Arya V	27	2004	India	LMIC	SEAR	RIA	CSS	no random sampling	Community	Adults
**30**	Asakura K	43	2020	Japan	HIC	WPR	LCMS/MS	CSS	no random sampling	Community	Adults
**31**	Aspell N	55	2019	UK	HIC	ER	ECLIA	CSS	multistage stratified probability sampling	National	Older
**32**	Aucoin M	45	2013	Canada	HIC	RA	ECLIA	CSS	no random sampling	Community	Children and Adolescents
**32**	Aucoin M	45	2013	Canada	HIC	RA	ECLIA	CSS	no random sampling	Community	Adults
**33**	Bachhel R	31	2015	India	LMIC	SEAR	NA	CSS	random sampling	Community	Adults
**34**	Bater J	48	2021	Mongolia	LMIC	WPR	ELISA	CSS	no random sampling	Community	Adolescents
**35**	Batieha A	32	2011	Jordan	UMIC	EMR	RIA	CSS	no random sampling	Community	Adults

**36**	Beer RJ	4	2020	Colombia	UMIC	RA	CLIA	CSS	multistage stratified sampling	National	Children
**36**	Beer RJ	4	2020	Colombia	UMIC	RA	CLIA	CSS	multistage stratified sampling	National	Children
**36**	Beer RJ	4	2020	Colombia	UMIC	RA	CLIA	CSS	multistage stratified sampling	National	Adults
**36**	Beer RJ	4	2020	Colombia	UMIC	RA	CLIA	CSS	multistage stratified sampling	National	Adolescents
**37**	Bener A	25	2009	Qatar	HIC	EMR	ECLIA	CSS	no random sampling	Community	Adolescents
**38**	Benjeddou K	32	2019	Morocco	LMIC	EMR	HPLC	CSS	no random sampling	Community	Children
**39**	Bettencourt A	41	2018	Portugal	HIC	ER	ECLIA	CSS	no random sampling	Community	Adults
**40**	Bezrati I	35	2016	Tunisia	LMIC	EMR	CLIA	CSS	no random sampling	Community	Children and Adolescents
**41**	Bhatt SP	28	2014	India	LMIC	SEAR	RIA	CSS	no random sampling	Community	Adults
**42**	Bhattoa HP	47	2013	Hungary	HIC	ER	HPLC	CSS	no random sampling	Community	Older
**43**	Bi X	1	2016	Singapore	HIC	WPR	LC-MS/MS	CSS	no random sampling	Community	Adults

**44**	Bjarnadottir A	64	2014	Iceland	HIC	ER	RIA	CSS	random sampling	School	Children
**45**	Black LJ	-35	2021	Australia	HIC	WPR	LC-MS/MS	CSS	stratified, multistage random sampling	National	Adults
**45**	Black LJ	-35	2021	Australia	HIC	WPR	LC-MS/MS	CSS	stratified, multistage random sampling	National	Adults
**45**	Black LJ	-35	2021	Australia	HIC	WPR	LC-MS/MS	CSS	stratified, multistage random sampling	National	Adults
**45**	Black LJ	-35	2021	Australia	HIC	WPR	LC-MS/MS	CSS	stratified, multistage random sampling	National	Adults
**45**	Black LJ	-35	2021	Australia	HIC	WPR	LC-MS/MS	CSS	stratified, multistage random sampling	National	Adults
**46**	Bodin J	9	2019	Ethiopia	LIC	AR	HPLC	CSS	systematic sampling	Community	Children
**47**	Bolland MJ	-37	2006	New Zealand	HIC	WPR	RIA	CSS	no random sampling	Community	Adults

**48**	Borissova AM	42	2013	Bulgaria	UMIC	ER	LC-MS/MS	CSS	no random sampling	Community	Adults
**49**	Brinkmann K	-35	2015	Chile	HIC	RA	ELISA	CSS	no random sampling	Community	Adults
**50**	Byun EJ	36	2017	Republic of Korea	HIC	WPR	LCMS/MS	CSS	multistage randomly sampling	National	Adolescents
**51**	Cabral MA	-8	2013	Brazil	UMIC	RA	CLIA	CSS	random sampling	Community	Older
**52**	Cairncross CT	-37	2017	New Zealand	HIC	WPR	LC-MS/MS	CSS	random sampling	National	Children
**53**	Capuano R	40	2021	Italy	HIC	ER	ELISA	CSS	random sampling	Community	Adults
**54**	Carrillo-Vega MF	19	2017	Mexico	UMIC	RA	CLIA	CSS	random sampling	Community	Older
**55**	Cashman KD	53	2013	Ireland	HIC	ER	ELISA	CSS	quota sampling	National	Adults
**56**	Ceccarelli M	41	2020	Italy	HIC	ER	CLIA	CSS	no random sampling	Check-up	Children and Adolescents
**57**	Chailurkit LO	13	2011	Thailand	UMIC	SEAR	LCMS/MS	CSS	random sampling	National	Adults
**58**	Chao YS	49	2013	Canada	HIC	RA	NA	CSS	no random sampling	Community	Older
**59**	Chao YS	49	2014	Canada	HIC	RA	NA	CSS	no random sampling	Community	Older
**60**	Chen J	39	2017	China	UMIC	WPR	RIA	CSS	random sampling	National	Older

**61**	Chin KY	3	2014	Malaysia	UMIC	WPR	ELISA	CSS	no random sampling	Community	Adults
**62**	Chirita-Emandi A	44	2015	Romania	UMIC	ER	HPLC	CSS	no random sampling	Check-up	Adults
**63**	Chlebna-Sokół D	52	2019	Poland	HIC	ER	ECLIA	CSS	random sampling	National	Children
**64**	Choi HR	37	2017	Republic of Korea	HIC	WPR	CLIA	CSS	no random sampling	Community	Older
**65**	Chung IH	37	2014	Republic of Korea	HIC	WPR	CLIA	CSS	multistage randomly sampling	National	Adolescents
**66**	Chung JY	37	2013	Republic of Korea	HIC	WPR	CLIA	CSS	multistage randomly sampling	National	Adults
**67**	Cinar N	39	2014	Turkey	UMIC	ER	RIA	CSS	no random sampling	Community	Adults
**68**	Contreras-Manzano A	19	2021	Mexico	UMIC	RA	CLIA	CSS	stratified, multistage probability sampling	National	Adults
**68**	Contreras-Manzano A	19	2021	Mexico	UMIC	RA	CLIA	CSS	stratified, multistage probability sampling	National	Adults
**68**	Contreras-Manzano A	19	2021	Mexico	UMIC	RA	CLIA	CSS	stratified, multistage	National	Adults

									probability sampling		
**69**	Cougnard-Grégoire A	44	2015	France	HIC	ER	CLIA	CSS	random sampling	Community	Elderly
**70**	Courraud J	77	2020	Denmark	HIC	ER	LC-MS/MS	CSS	random sampling	Community	Children
**71**	Crowe FL	51	2019	UK	HIC	ER	NA	CSS	random sampling	National	Adults
**71**	Crowe FL	51	2019	UK	HIC	ER	NA	CSS	random sampling	National	Adults
**71**	Crowe FL	51	2019	UK	HIC	ER	NA	CSS	random sampling	National	Adults
**71**	Crowe FL	51	2019	UK	HIC	ER	NA	CSS	random sampling	National	Adults
**71**	Crowe FL	51	2019	UK	HIC	ER	NA	CSS	random sampling	National	Adults
**71**	Crowe FL	51	2019	UK	HIC	ER	NA	CSS	random sampling	National	Adults
**72**	Dalgård C	62	2010	Denmark	HIC	ER	LC-MS/MS	CSS	random sampling	Community	Elderly
**73**	Daly RM	-35	2012	Australia	HIC	WPR	CLIA	CSS	random sampling	National	Elderly
**73**	Daly RM	-35	2012	Australia	HIC	WPR	CLIA	CSS	random sampling	National	Elderly
**73**	Daly RM	-35	2012	Australia	HIC	WPR	CLIA	CSS	random sampling	National	Adults

**73**	Daly RM	-35	2012	Australia	HIC	WPR	CLIA	CSS	random sampling	National	Adults
**73**	Daly RM	-35	2012	Australia	HIC	WPR	CLIA	CSS	random sampling	National	Adults
**73**	Daly RM	-35	2012	Australia	HIC	WPR	CLIA	CSS	random sampling	National	Adults
**74**	de Oliveira CL	-15	2020	Brazil	UMIC	RA	CLIA	CSS	random sampling	School	Adolescents
**75**	Djennane M	36	2014	Algeria	LMIC	AR	ECLIA	CSS	random sampling	School	Children
**76**	Drali O	36	2021	Algeria	LMIC	AR	ELISA	CSS	random sampling	School	Children
**77**	Duarte C	38	2020	Portugal	HIC	ER	CLIA	CSS	multistage random sampling	National	Adults
**78**	El Hayek J	63	2010	Canada	HIC	RA	ECLIA	CSS	random sampling	Community	Children
**79**	El Hayek J	45	2013	Canada	HIC	RA	CLIA	CSS	no random sampling	Community	Children
**80**	El-Khateeb M	32	2019	Jordan	UMIC	EMR	ELISA	CSS	no random sampling	National	Adults
**81**	Eloi M	-23	2016	Brazil	UMIC	RA	CLIA	CSS	random sampling	Community	Adults
**82**	Fang F	39	2018	China	UMIC	WPR	ECLIA	CSS	stratified cluster sampling	Community	Adults

**83**	Fayet-Moore F	-34	2019	Australia	HIC	WPR	RIA	CSS	no random sampling	Check-up	Adults
**84**	Feketea GM	38	2021	Greece	HIC	ER	ECLIA	CSS	no random sampling	Community	Children and Adolescents
**85**	Feng X	36	2016	China	UMIC	WPR	ELISA	CSS	random sampling	Community	Older
**86**	Fernández Bustillo JM	42	2018	Spain	HIC	ER	CLIA	CSS	no random sampling	Check-up	Children and Adolescents
**87**	Flores ME	19	2021	Mexico	UMIC	RA	CLIA	CSS	stratified, multistage probability sampling	National	Children
**87**	Flores ME	19	2021	Mexico	UMIC	RA	CLIA	CSS	stratified, multistage probability sampling	National	Children
**88**	Ganmaa D	48	2014	Mongolia	LMIC	WPR	ECLIA	CSS	no random sampling	Community	Adults
**89**	Gariballa S	23	2022	United Arab Emirates	HIC	EMR	CLIA	CSS	no random sampling	Community	Adults
**90**	Gebreegziabher T	7	2013	Ethiopia	LIC	AR	ELISA	CSS	no random sampling	Community	Adults
**91**	Gilbert-Diamond D	4	2010	Colombia	UMIC	RA	CLIA	CSS	cluster random-sampling	School	Children
**92**	Gill TK	-36	2014	Australia	HIC	WPR	ELISA	CSS	random sampling	National	Older

**92**	Gill TK	-36	2014	Australia	HIC	WPR	ELISA	CSS	random sampling	National	Elderly
**92**	Gill TK	-36	2014	Australia	HIC	WPR	ELISA	CSS	random sampling	National	Elderly
**92**	Gill TK	-36	2014	Australia	HIC	WPR	ELISA	CSS	random sampling	National	Adults
**92**	Gill TK	-36	2014	Australia	HIC	WPR	ELISA	CSS	random sampling	National	Adults
**92**	Gill TK	-36	2014	Australia	HIC	WPR	ELISA	CSS	random sampling	National	Adults
**92**	Gill TK	-36	2014	Australia	HIC	WPR	ELISA	CSS	random sampling	National	Adults
**93**	Ginter JK	43	2013	Canada	HIC	RA	LC-MS/MS	CSS	no random sampling	Community	Older
**94**	Glatt DU	55	2022	UK	HIC	ER	HPLC	CSS	no random sampling	School	Children
**95**	Gökta O	40	2020	Turkey	UMIC	ER	ECLIA	CSS	no random sampling	National	Adults
**96**	Golbahar J	26	2014	Bahrain	HIC	EMR	LC-MS/MS	CSS	no random sampling	Community	Adults
**97**	González G	-33	2007	Chile	HIC	RA	RIA	CSS	no random sampling	Community	Adults
**98**	González-Gross M	55	2012	Europe	HIC	ER	ELISA	CSS	stratified random and cluster sampling	School	Adolescents

**99**	González-Molero I	40	2011	Spain	HIC	ER	ELISA	CSS	random sampling	Community	Adults
**100**	Gordon CM	42	2004	USA	HIC	RA	CLIA	CSS	no random sampling	Community	Children and Adolescents
**101**	Goswami R	28	2009	India	LMIC	SEAR	ECLIA	CSS	no random sampling	Community	Adults
**102**	Granlund L	63	2016	Sweden	HIC	ER	LC-MS/MS	CSS	random sampling	Community	Adults
**103**	Greene-Finestone LS	43	2011	Canada	HIC	RA	CLIA	CSS	random sampling	National	Elderly
**103**	Greene-Finestone LS	43	2011	Canada	HIC	RA	CLIA	CSS	random sampling	National	Adults
**103**	Greene-Finestone LS	43	2011	Canada	HIC	RA	CLIA	CSS	random sampling	National	Adults
**104**	Griffin TP	53	2020	Ireland	HIC	ER	LC-MS/MS	CSS	no random sampling	Community	Adults
**105**	Gromova O	51	2020	Kazakhstan	UMIC	ER	CLIA	CSS	random sampling	National	Adults
**106**	Guo S	-35	2014	Australia	HIC	WPR	LC-MS/MS	CSS	no random sampling	Community	Adults
**107**	Han B	31	2017	China	UMIC	WPR	CLIA	CSS	random sampling	Community	Adults
**108**	Hansen L	55	2018	Denmark	HIC	ER	LC-MS/MS	CSS	random sampling	Community	Children
**108**	Hansen L	55	2018	Denmark	HIC	ER	LC-MS/MS	CSS	no random sampling	Community	Adults

**109**	Harinarayan CV	13	2007	India	LMIC	SEAR	RIA	CSS	no random sampling	Community	Adults
**110**	Harkness LS	41	2005	USA	HIC	RA	CLIA	CSS	no random sampling	Community	Children and Adolescents
**111**	Hashemipour S	35	2004	Iran	LMIC	EMR	RIA	CSS	random sampling	Community	Adults
**112**	Hatun S	40	2005	Turkey	UMIC	ER	ECLIA	CSS	no random sampling	Community	Adolescents
**113**	Hazell TJ	45	2015	Lebanon	UMIC	EMR	CLIA	CSS	random sampling	Community	Adolescents
**114**	Hekimsoy Z	38	2010	Turkey	UMIC	ER	ECLIA	CSS	no random sampling	Community	Adults
**115**	Herrick KA	38	2019	USA	HIC	RA	RIA	CSS	stratified multi-stage clustered probability sampling	National	Older
**115**	Herrick KA	38	2019	USA	HIC	RA	RIA	CSS	stratified multi-stage clustered probability sampling	National	Children
**115**	Herrick KA	38	2019	USA	HIC	RA	RIA	CSS	stratified multi-stage clustered probability sampling	National	Children
**115**	Herrick KA	38	2019	USA	HIC	RA	RIA	CSS	stratified multi-stage clustered	National	Adults

									probability sampling		
**115**	Herrick KA	38	2019	USA	HIC	RA	RIA	CSS	stratified multi-stage clustered probability sampling	National	Adults
**115**	Herrick KA	38	2019	USA	HIC	RA	RIA	CSS	stratified multi-stage clustered probability sampling	National	Adolescents
**116**	Hintzpeter B	52	2008	Germany	HIC	ER	CLIA	CSS	stratified random sampling	Community	Children and Adolescents
**117**	Hirani V	51	2012	UK	HIC	ER	ECLIA	CSS	stratified random sampling	Community	Elderly
**118**	Hirani V	-33	2013	Australia	HIC	WPR	RIA	CSS	no random sampling	Community	Elderly
**118**	Hirani V	-33	2013	Australia	HIC	WPR	RIA	CSS	no random sampling	Community	Elderly
**118**	Hirani V	-33	2013	Australia	HIC	WPR	RIA	CSS	no random sampling	Community	Elderly
**118**	Hirani V	-33	2013	Australia	HIC	WPR	RIA	CSS	no random sampling	Community	Elderly
**119**	Hoge A	51	2015	Belgium	HIC	ER	ECLIA	CSS	random sampling	National	Adults

**120**	Ho-Pham LT	10	2011	Vietnam	LMIC	WPR	ECLIA	CSS	random sampling	Community	Adults
**121**	Horton-French K	-35	2021	Australia	HIC	WPR	LC-MS/MS	CSS	stratified, multistage random sampling	National	Adults
**121**	Horton-French K	-35	2021	Australia	HIC	WPR	LC-MS/MS	CSS	stratified, multistage random sampling	National	Adolescents
**122**	Houghton LA	-1	2019	Kenya	LMIC	AR	LC-MS/MS	CSS	random stratified sampling	Community	Children
**123**	Hovsepian S	32	2011	Iran	LMIC	EMR	RIA	CSS	no random sampling	Community	Adults
**124**	Hribar M	45	2020	Slovenia	HIC	ER	CLIA	CSS	random sampling	National	Elderly
**124**	Hribar M	45	2020	Slovenia	HIC	ER	CLIA	CSS	random sampling	National	Adults
**125**	Hussain T	30	2021	Afghanistan	LIC	EMR	ECLIA	CSS	no random sampling	Community	Adults
**126**	Hutchings N	40	2022	Armenia	UMIC	ER	LCMS/MS	CSS	no random sampling	Community	Adults
**127**	Ikonen H	65	2021	Finland	HIC	ER	LC-MS/MS	CSS	random sampling	Community	Adults

**128**	Isa H	26	2020	Bahrain	HIC	EMR	LC-MS/MS	CSS	no random sampling	Community	Adolescents
**129**	Islam MZ	23	2002	Bangladesh	LMIC	SEAR	ECLIA	CSS	random sampling	Community	Adults
**130**	Islam MZ	23	2008	Bangladesh	LMIC	SEAR	ELISA	CSS	no random sampling	Community	Adults
**131**	Jääskeläinen T	60	2017	Finland	HIC	ER	CLIA	CSS	multistage stratified random cluster sampling	National	Adults
**132**	Janssen HC	52	2013	The Netherlands	HIC	ER	CLIA	CSS	random sampling	Community	Adults
**133**	Jayashri R	11	2020	India	LMIC	SEAR	ECLIA	CSS	random sampling	Community	Adults
**134**	Jayatissa R	6	2019	Sri Lanka	LMIC	SEAR	CLIA	CSS	cluster random-sampling	National	Adolescents
**135**	Jiang W	28	2020	China	UMIC	WPR	ECLIA	CSS	no random sampling	National	Adults
**136**	Johnson MA	33	2008	USA	HIC	RA	RIA	CSS	no random sampling	Community	Elderly
**137**	Jolliffe DA	51	2016	UK	HIC	ER	LC-MS/MS	CSS	no random sampling	Community	Adults
**138**	Jorde R	59	2010	Norway	HIC	ER	ECLIA	CSS	random sampling	National	Adults
**139**	Joukar F	35	2020	Iran	LMIC	EMR	ECLIA	CSS	no random sampling	National	Adults

**140**	Junaid K	31	2015	Pakistan	LMIC	EMR	ELISA	CSS	no random sampling	Community	Adults
**141**	Kaddam IM	24	2017	Saudi Arabia	HIC	EMR	ECLIA	CSS	multistage cluster random sampling	Community	Adults
**141**	Kaddam IM	24	2017	Saudi Arabia	HIC	EMR	ECLIA	CSS	multistage cluster random sampling	Community	Adolescents
**142**	Kagotho E	-1	2018	Kenya	LMIC	AR	ECLIA	CSS	no random sampling	Check-up	Adults
**143**	Kapil U	32	2018	India	LMIC	SEAR	CLIA	CSS	no random sampling	Community	Adolescents
**143**	Kapil U	32	2018	India	LMIC	SEAR	CLIA	CSS	no random sampling	Community	Adolescents
**144**	Karagüzel G	41	2014	Turkey	UMIC	ER	ELISA	CSS	no random sampling	National	Adolescents
**145**	Karin Z	45	2018	Croatia	HIC	ER	ECLIA	CSS	no random sampling	School	Children
**146**	Karonova T	60	2016	Russian Federation	UMIC	ER	CLIA	CSS	random sampling	Community	Adults
**147**	Kaykhaei MA	30	2011	Iran	LMIC	EMR	ECLIA	CSS	no random sampling	Community	Adults
**148**	Khan AH	24	2012	Pakistan	LMIC	EMR	ECLIA	CSS	no random sampling	Community	Adults

**149**	Kim SY	37	2020	Republic of Korea	HIC	WPR	CLIA	CSS	multistage randomly sampling	National	Adults
**150**	Kim YS	37	2020	Republic of Korea	HIC	WPR	CLIA	CSS	multistage randomly sampling	National	Adolescents
**151**	Klenk J	48	2013	Germany	HIC	ER	ECLIA	CSS	random sampling	Community	Elderly
**152**	Kouda K	35	2013	Japan	HIC	WPR	RIA	CSS	no random sampling	Community	Adolescents
**153**	Koyama S	36	2021	Japan	HIC	WPR	RIA	CSS	no random sampling	Community	Adolescents
**154**	Kremer R	33	2009	USA	HIC	RA	RIA	CSS	no random sampling	Community	Adults
**155**	Kull M Jr	59	2009	Estonia	HIC	ER	RIA	CSS	random sampling	Community	Adults
**156**	Kunz C	51	2018	Germany	HIC	ER	CLIA	CSS	no random sampling	Check-up	Children
**156**	Kunz C	51	2018	Germany	HIC	ER	CLIA	CSS	no random sampling	Check-up	Children
**156**	Kunz C	51	2018	Germany	HIC	ER	CLIA	CSS	no random sampling	Check-up	Children
**156**	Kunz C	51	2018	Germany	HIC	ER	CLIA	CSS	no random sampling	Check-up	Adolescents
**157**	Laird E	53	2018	Ireland	HIC	ER	LC-MS/MS	CSS	stratified clustered	Community	Older

									random sampling		
**158**	Langlois K	45	2010	Canada	HIC	RA	CLIA	CSS	random sampling	National	Adults
**159**	Lappe JM	41	2006	USA	HIC	RA	RIA	CSS	random sampling	Community	Older
**160**	Lardner E	53	2011	Ireland	HIC	ER	RIA	CSS	no random sampling	Community	Adults
**161**	Lategan R	-29	2016	South Africa	UMIC	AR	CLIA	CSS	stratified cluster sampling	Community	Adults
**162**	Le Goaziou MF	45	2011	France	HIC	ER	RIA	CSS	no random sampling	Community	Adults
**163**	Lee J	37	2021	Republic of Korea	HIC	WPR	CLIA	CSS	no random sampling	Community	Adults
**164**	Leung RY	22	2017	Hong kong	HIC	WPR	ELISA	CSS	no random sampling	Community	Adults
**165**	Li H	33	2020	China	UMIC	WPR	CLIA	CSS	multistage sampling	National	Children
**165**	Li H	33	2020	China	UMIC	WPR	CLIA	CSS	multistage sampling	National	Children
**166**	Li L	30	2020	China	UMIC	WPR	LC-MS/ MS	CSS	no random sampling	National	Adults
**167**	Li S	28	2014	China	UMIC	WPR	ELISA	CSS	random sampling	Community	Adults

**168**	Lima-Costa MF	-15	2020	Brazil	UMIC	RA	CLIA	CSS	stratified random sampling	Community	Older
**169**	Lin LY	51	2021	UK	HIC	ER	CLIA	CSS	random sampling	National	Adults
**170**	Liu X	38	2018	USA	HIC	RA	LC-MS/MS	CSS	stratified multi-stage clustered probability sampling	National	Older
**170**	Liu X	38	2018	USA	HIC	RA	LC-MS/MS	CSS	stratified multi-stage clustered probability sampling	National	Adults
**170**	Liu X	38	2018	USA	HIC	RA	LC-MS/MS	CSS	stratified multi-stage clustered probability sampling	National	Adults
**171**	Liu X	22	2020	Macao	HIC	WPR	ECLIA	CSS	multistage sampling	National	Older
**172**	Lopes JB	-23	2009	Brazil	UMIC	RA	RIA	CSS	no random sampling	Community	Elderly
**173**	Lucas JA	-37	2005	New Zealand	HIC	WPR	RIA	CSS	random sampling	Community	Adults
**174**	Madsen KH	56	2014	Denmark	HIC	ER	LC-MS/MS	CSS	stratified random sampling	Community	Children

**174**	Madsen KH	56	2014	Denmark	HIC	ER	LC-MS/MS	CSS	stratified random sampling	Community	Adults
**175**	Maguire JL	43	2011	Canada	HIC	RA	LC–MS/MS	CSS	no random sampling	Community	Children
**176**	Marzban M	29	2021	Iran	LMIC	EMR	ELISA	CSS	random sampling	Community	Adults
**177**	Majumdar V	13	2011	India	LMIC	SEAR	ELISA	CSS	random sampling	Community	Adults
**178**	Maldonado G	-1	2017	Ecuador	UMIC	RA	CLIA	CSS	no random sampling	Community	Adults
**179**	Mallah EM	31	2011	Jordan	UMIC	EMR	ELISA	CSS	no random sampling	Community	Adults
**180**	Man PW	52	2016	The Netherlands	HIC	ER	LC-MS/MS	CSS	no random sampling	Community	Adults
**181**	Manios Y	38	2017	Greece	HIC	ER	CLIA	CSS	multistage stratified random sampling	School	Children and Adolescents
**182**	Mansbach JM	38	2009	USA	HIC	RA	RIA	CSS	stratified multi-stage clustered probability sampling	National	Children
**182**	Mansbach JM	38	2009	USA	HIC	RA	RIA	CSS	stratified multi-stage clustered	National	Children

									probability sampling		
**183**	Masoud MS	24	2020	Saudi Arabia	HIC	EMR	ECLIA	CSS	random sampling	Community	Adolescents
**184**	Matheï C	51	2013	Belgium	HIC	ER	ECLIA	CSS	random sampling	Community	Elderly
**185**	Mechenro J	12	2018	India	LMIC	SEAR	ELISA	CSS	no random sampling	Community	Adults
**186**	Mechenro J	12	2018	India	LMIC	SEAR	ELISA	CSS	no random sampling	Community	Adults
**186**	Meddeb N	36	2005	Tunisia	LMIC	EMR	RIA	CSS	no random sampling	Community	Adults
**186**	Mechenro J	12	2018	India	LMIC	SEAR	ELISA	CSS	no random sampling	Community	Adults
**187**	Mehboobali N	24	2015	Pakistan	LMIC	EMR	ECLIA	CSS	random sampling	Community	Adults
**188**	Metwally ASM	24	2021	Saudi Arabia	HIC	EMR	ELISA	CSS	random sampling	National	Adolescents
**189**	Meyer HE	59	2004	Norway	HIC	ER	RIA	CSS	random sampling	Community	Adults
**190**	Meyer HE	7	2008	Sri Lanka	LMIC	SEAR	RIA	CSS	no random sampling	Community	Adults
**191**	Miljkovic I	11	2011	Trinidad and Tobago	HIC	RA	LC-MS/MS	CSS	no random sampling	Community	Elderly
**192**	Misra P	28	2017	India	LMIC	SEAR	ECLIA	CSS	random sampling	Community	Adults

**193**	Mitchell DM	52	2012	USA	HIC	RA	ELISA	CSS	no random sampling	Community	Adults
**194**	Mogire RM	no	2021	Kenya, Uganda, Burkina Faso, Gambia, South Africa	LMIC	AR	CLIA	CSS	random sampling	Community	Children
**195**	Moreno-Reyes R	50	2009	Belgium	HIC	ER	RIA	CSS	multistage stratified probability sampling	National	Adults
**196**	Moussavi M	33	2005	Iran	LMIC	EMR	RIA	CSS	multistage sampling	Community	Adolescents
**197**	Moy FM	3	2011	Malaysia	UMIC	WPR	CLIA	CSS	no random sampling	Community	Adults
**198**	Moy FM	3	2017	Malaysia	UMIC	WPR	ECLIA	CSS	no random sampling	Community	Adults
**199**	Muhairi SJ	23	2013	United Arab Emirates	HIC	EMR	RIA	CSS	no random sampling	Community	Adolescents
**200**	Mutua AM	0.1	2020	Uganda	LIC	AR	CLIA	CSS	no random sampling	Community	Children
**201**	Nadeem S	24	2018	Pakistan	LMIC	EMR	NA	CSS	no random sampling	Community	Adults
**202**	Naeem Z	24	2011	Saudi Arabia	HIC	EMR	ELISA	CSS	random sampling	Community	Adults

**203**	Nakamura K	37	2008	Japan	HIC	WPR	RIA	CSS	random sampling	National	Older
**204**	Nakhaee S	32	2019	Iran	LMIC	EMR	ECLIA	CSS	stratified sampling	Community	Adults
**205**	Nälsén C	59	2020	Sweden	HIC	ER	LCMS/MS	CSS	random sampling	National	Adults
**206**	Nälsén C	59	2020	Sweden	HIC	ER	LC-MS/MS	CSS	random sampling	National	Children
**206**	Naqvi A	15	2017	Guatemala	UMIC	RA	RIA	CSS	no random sampling	Community	Adolescents
**207**	Ní Chaoimh C	51	2018	Ireland	HIC	ER	LC-MS/MS	CSS	no random sampling	Community	Children
**208**	NHANES(2015–2016)	52	2022	USA	HIC	RA	LC-MS/MS	CSS	multistage randomly sampling	National	Elderly
**208**	NHANES(2015–2016)	52	2022	USA	HIC	RA	LC-MS/MS	CSS	multistage randomly sampling	National	Children
**208**	NHANES(2015–2016)	52	2022	USA	HIC	RA	LC-MS/MS	CSS	multistage randomly sampling	National	Adults
**208**	NHANES (2015–2016)	52	2022	USA	HIC	RA	LC-MS/MS	CSS	multistage randomly sampling	National	Adults

**208**	NHANES(2017–2018)	52	2022	USA	HIC	RA	LC-MS/MS	CSS	multistage randomly sampling	National	Elderly
**208**	NHANES(2017–2018)	52	2022	USA	HIC	RA	LC-MS/MS	CSS	multistage randomly sampling	National	Children
**208**	NHANES(2017–2018)	52	2022	USA	HIC	RA	LC-MS/MS	CSS	multistage randomly sampling	National	Adults
**208**	NHANES(2017–2018)	52	2022	USA	HIC	RA	LC-MS/MS	CSS	multistage randomly sampling	National	Adults
**209**	Niafar M	38	2009	Iran	LMIC	EMR	CLIA	CSS	simple random sampling	Community	Older
**210**	Nichols EK	31	2015	Jordan	UMIC	EMR	CLIA	CSS	no random sampling	Community	Children
**211**	Nielsen NO	65	2014	Denmark	HIC	ER	LC-MS/MS	CSS	stratified random sampling	Community	Adults
**212**	Nikooyeh B	34	2017	Iran	LMIC	EMR	ELISA	CSS	no random sampling	Community	Adults
**213**	Nimitphong H	13	2013	Thailand	UMIC	SEAR	LC-MS/MS	CSS	no random sampling	Community	Adults
**213**	Nikooyeh B	34	2017	Iran	LMIC	EMR	ELISA	CSS	no random sampling	Community	Adults

**214**	Oberg J	69	2014	Norway	HIC	ER	LC-MS/MS	CSS	random sampling	Community	Adolescents
**215**	Oliveri B	-34	2004	Argentina	UMIC	RA	RIA	CSS	no random sampling	National	Elderly
**216**	Orces CH	-5	2015	Ecuador	UMIC	RA	LC–MS/MS	CSS	random sampling	National	Elderly
**217**	Orces CH	-5	2015	Ecuador	UMIC	RA	LC–MS/MS	CSS	random sampling	National	Older
**217**	Orces CH	-5	2015	Ecuador	UMIC	RA	LC–MS/MS	CSS	random sampling	National	Elderly
**217**	Orwoll E	52	2009	USA	HIC	RA	LC–MS/MS	CSS	random sampling	Community	Elderly
**218**	Öztürk ZA	36	2017	Turkey	UMIC	ER	NA	CSS	no random sampling	Community	Adults
**219**	Pan T	24	2018	India	LMIC	SEAR	RIA	CSS	random sampling	Community	Adults
**220**	Patel JV	54	2013	UK	HIC	ER	LC-MS/MS	CSS	random sampling	Community	Adults
**221**	Paul TV	11	2008	India	LMIC	SEAR	RIA	CSS	no random sampling	Community	Older
**222**	Penrose K	42	2012	USA	HIC	RA	NA	CSS	no random sampling	Community	Adults
**223**	Pérez-Llamas F	37	2008	Spain	HIC	ER	HPLC	CSS	no random sampling	Community	Elderly
**224**	Perna L	49	2012	Germany	HIC	ER	CLIA	CSS	random sampling	Community	Older

**225**	Peters BS	-23	2009	Brazil	UMIC	RA	RIA	CSS	no random sampling	School	Adolescents
**226**	Petrenya N	69	2020	Norway	HIC	ER	CLIA	CSS	based on a large proportion sampling	National	Adults
**227**	Qorbani M	35	2021	Iran	LMIC	EMR	ECLIA	CSS	random sampling	National	Adolescents
**228**	Rabenberg M	52	2015	Germany	HIC	ER	CLIA	CSS	stratified random sampling	National	Elderly
**228**	Rabenberg M	52	2015	Germany	HIC	ER	CLIA	CSS	stratified random sampling	National	Adults
**228**	Rabenberg M	52	2015	Germany	HIC	ER	CLIA	CSS	stratified random sampling	National	Adults
**229**	Rabufetti A	46	2019	Switzerland	HIC	ER	CLIA	CSS	no random sampling	Check-up	Adolescents
**230**	Rafraf M	38	2014	Iran	LMIC	EMR	ELISA	CSS	multistage stratified random sampling	Community	Adolescents
**231**	Rahmadhani R	3	2017	Malaysia	UMIC	WPR	ECLIA	CSS	no random sampling	Community	Adolescents

**232**	Rahman A	29	2020	Kuwait	HIC	EMR	CLIA	CSS	no random sampling	Community	Adolescents
**233**	Ramakrishnan S	30	2011	India	LMIC	SEAR	ELISA	CSS	random sampling	Community	Adults
**234**	Raposo L	41	2017	Portugal	HIC	ER	CLIA	CSS	random sampling	National	Adults
**235**	Riverin B	52	2013	Canada	HIC	RA	RIA	CSS	random sampling	Community	Adults
**236**	Riverin B	52	2014	Canada	HIC	RA	RIA	CSS	random sampling	Community	Children and Adolescents
**237**	Robinson PJ	-35	2013	Australia	HIC	WPR	NA	CSS	no random sampling	Community	Elderly
**237**	Robinson PJ	-35	2013	Australia	HIC	WPR	NA	CSS	no random sampling	Community	Elderly
**238**	Rodríguez-Rodríguez E	40	2011	Spain	HIC	ER	ELISA	CSS	no random sampling	School	Children and Adolescents
**239**	Saeed BQ	24	2021	United Arab Emirates	HIC	EMR	ECLIA	CSS	no random sampling	Community	Adults
**240**	Saki F	30	2017	Iran	LMIC	EMR	HPLC	CSS	random sampling	Community	Adolescents
**241**	Sakyi SA	8	2021	Ghana	LMIC	AR	ELISA	CSS	systematic random sampling	Check-up	Adults
**242**	Saliba W	31	2012	Israel	HIC	ER	CLIA	CSS	no random sampling	National	Adults

**242**	Saliba W	31	2012	Israel	HIC	ER	CLIA	CSS	no random sampling	National	Adolescents
**243**	Samefors M	65	2014	Sweden	HIC	ER	HPLC	CSS	random sampling	Community	Elderly
**244**	Santos A	38	2017	Portugal	HIC	ER	CLIA	CSS	quota sampling	National	Elderly
**245**	Santos BR	-25	2012	Brazil	UMIC	RA	RIA	CSS	no random sampling	School	Children and Adolescents
**246**	Santos BR	-30	2019	Brazil	UMIC	RA	CLIA	CSS	no random sampling	Community	Adults
**247**	Sarafin K	45	2015	Canada	HIC	RA	ECLIA	CSS	random sampling	Community	Adults
**248**	Saraiva GL	-23	2005	Brazil	UMIC	RA	RIA	CSS	random sampling	Community	Elderly
**249**	Scalco R	-30	2008	Brazil	UMIC	RA	CLIA	CSS	no random sampling	Community	Elderly
**250**	Schramm S	50	2017	Germany	HIC	ER	ELISA	CSS	random sampling	Community	Adults
**251**	Science M	43	2017	Canada	HIC	RA	NA	CSS	no random sampling	Community	Children and Adolescents
**252**	Seo JA	37	2013	Republic of Korea	HIC	WPR	CLIA	CSS	no random sampling	Community	Older
**253**	Shady MM	30	2015	Egypt	LMIC	EMR	ELISA	CSS	no random sampling	School	Children
**254**	Sharawat IK	25	2019	India	LMIC	SEAR	ELISA	CSS	random sampling	Community	Adolescents

**255**	Shchubelka K	48	2020	Ukraine	LMIC	ER	ECLIA	CSS	random sampling	Community	Adults
**256**	Sheikh A	25	2012	Pakistan	LMIC	EMR	RIA	CSS	no random sampling	Community	Adults
**257**	Sherchand O	27	2018	Nepal	LMIC	SEAR	CLIA	CSS	no random sampling	Community	Adults
**258**	Sherief LM	30	2021	Egypt	LMIC	EMR	RIA	CSS	random sampling	School	Adolescents
**259**	Shetty S	13	2014	India	LMIC	SEAR	ELISA	CSS	cluster random sampling	Community	Older
**260**	Shivane VK	19	2011	India	LMIC	SEAR	RIA	CSS	no random sampling	Community	Adults
**261**	Sioen I	51	2012	Belgium	HIC	ER	RIA	CSS	random sampling	Community	Children
**262**	Skull SA	-37	2003	Australia	HIC	WPR	RIA	CSS	no random sampling	Community	Adults
**263**	Smith G	12	2016	Cambodia	LMIC	WPR	ELISA	CSS	no random sampling	National	Older
**263**	Smith G	12	2016	Cambodia	LMIC	WPR	ELISA	CSS	no random sampling	National	Children
**263**	Smith G	12	2016	Cambodia	LMIC	WPR	ELISA	CSS	no random sampling	National	Adults
**264**	Smith N	51	2021	UK	HIC	ER	ECLIA	CSS	no random sampling	Community	Adults
**265**	Sochorová L	50	2018	Czech Republic	HIC	ER	ECLIA	CSS	no random sampling	Check-up	Children

**266**	Sokolovic S	44	2017	Bosnia and Herzegovina	UMIC	ER	CLIA	CSS	no random sampling	Check-up	Older
**266**	Sokolovic S	44	2017	Bosnia and Herzegovina	UMIC	ER	CLIA	CSS	no random sampling	Check-up	Adults
**266**	Sokolovic S	44	2017	Bosnia and Herzegovina	UMIC	ER	CLIA	CSS	no random sampling	Check-up	Adults
**267**	Solis-Urra P	-33	2019	Chile	HIC	RA	LC–MS/MS	CSS	stratified multistage probability sample	National	Elderly
**267**	Solis-Urra P	-33	2019	Chile	HIC	RA	LC–MS/MS	CSS	stratified multistage probability sample	National	Adults
**268**	Song HR	35	2014	Republic of Korea	HIC	WPR	CLIA	CSS	no random sampling	National	Older
**269**	Souberbielle JC	48	2016	France	HIC	ER	ECLIA	CSS	random sampling	Community	Adults
**270**	Srimani S	24	2017	India	LMIC	SEAR	ELISA	CSS	random sampling	Community	Adults
**271**	Sulimani RA	24	2016	Saudi Arabia	HIC	EMR	ECLIA	CSS	no random sampling	Community	Adolescents
**272**	Suryanarayana P	17	2018	India	LMIC	SEAR	CLIA	CSS	random sampling	Community	Older
**273**	Tangoh DA	4	2018	Cameroon	LMIC	AR	ELISA	CSS	convenient sampling	Community	Adults

**274**	Ten Haaf DSM	52	2019	The Netherlands	HIC	ER	HPLC	CSS	no random sampling	Community	Elderly
**275**	Thuesen B	55	2012	Denmark	HIC	ER	HPLC	CSS	stratified random sampling	Community	Adults
**276**	Tolppanen AM	51	2012	UK	HIC	ER	HPLC	CSS	no random sampling	Community	Children
**277**	Tønnesen R	55	2016	Denmark	HIC	ER	CLIA	CSS	no random sampling	Community	Adults
**278**	Tran B	-35	2013	Australia	HIC	WPR	CLIA	CSS	random sampling	Community	Older
**278**	Tran B	-35	2013	Australia	HIC	WPR	CLIA	CSS	random sampling	Community	Elderly
**278**	Tran B	-35	2013	Australia	HIC	WPR	CLIA	CSS	random sampling	Community	Elderly
**278**	Tran B	-35	2013	Australia	HIC	WPR	CLIA	CSS	random sampling	Community	Elderly
**278**	Tran B	-35	2013	Australia	HIC	WPR	CLIA	CSS	random sampling	Community	Elderly
**279**	Tseng M	40	2009	USA	HIC	RA	CLIA	CSS	no random sampling	Community	Adults
**280**	Unger MD	-23	2010	Brazil	UMIC	RA	ELISA	CSS	no random sampling	Community	Adults
**281**	Uush T	47	2013	Mongolia	LMIC	WPR	ELISA	CSS	no random sampling	National	Children

**281**	Uush T	47	2013	Mongolia	LMIC	WPR	ELISA	CSS	no random sampling	National	Children
**282**	Vallejo MS	-34	2020	Chile	HIC	RA	ECLIA	CSS	random sampling	Community	Adults
**283**	Vallianou N	37	2012	Greece	HIC	ER	ECLIA	CSS	no random sampling	Check-up	Adults
**284**	Vasudevan B	11	2021	India	LMIC	SEAR	CLIA	CSS	no random sampling	Community	Adults
**285**	Vierucci F	43	2014	Italy	HIC	ER	RIA	CSS	no random sampling	Check-up	Children and Adolescents
**286**	von Hurst PR	-37	2010	New Zealand	HIC	WPR	RIA	CSS	no random sampling	Community	Adults
**287**	Voortman T	52	2015	The Netherlands	HIC	ER	LC-MS/MS	CSS	no random sampling	Community	Children
**288**	Vupputuri MR	28	2006	India	LMIC	SEAR	RIA	CSS	no random sampling	Community	Adults
**289**	Wakayo T	8	2015	Ethiopia	LIC	AR	LC-MS/MS	CSS	multi-stage stratified random sampling	School	Adolescents
**290**	Ward M	51	2011	UK	HIC	ER	ELISA	CSS	random sampling	Community	Adults
**291**	White Z	-25	2019	South Africa	UMIC	AR	CLIA	CSS	no random sampling	School	Children
**292**	Wyskida M	52	2018	Poland	HIC	ER	ELISA	CSS	random sampling	National	Elderly

**293**	Yan X	38	2019	China	UMIC	WPR	HPLC	CSS	no random sampling	Community	Adults
**294**	Yang K	36	2020	China	UMIC	WPR	ELISA	CSS	no random sampling	Community	Adults
**295**	Yousef S	45	2021	Canada	HIC	RA	CLIA	CSS	random sampling	National	Elderly
**295**	Yousef S	45	2021	Canada	HIC	RA	CLIA	CSS	random sampling	National	Children
**295**	Yousef S	45	2021	Canada	HIC	RA	CLIA	CSS	random sampling	National	Children
**295**	Yousef S	45	2021	Canada	HIC	RA	CLIA	CSS	random sampling	National	Adults
**295**	Yousef S	45	2021	Canada	HIC	RA	CLIA	CSS	random sampling	National	Adolescents
**296**	Yu L	24	2020	China	UMIC	WPR	ECLIA	CSS	no random sampling	Community	Children
**296**	Yu L	24	2020	China	UMIC	WPR	ECLIA	CSS	no random sampling	Community	Children
**297**	Yu S	35	2015	China	UMIC	WPR	LC-MS/MS	CSS	no random sampling	National	Adults
**298**	Zargar AH	34	2007	India	LMIC	SEAR	RIA	CSS	no random sampling	Community	Adults
**299**	Zgaga L	55	2011	UK	HIC	ER	LC-MS/MS	CSS	random sampling	Community	Adults
**300**	Zhang FF	29	2016	Kuwait	HIC	EMR	ECLIA	CSS	cluster sampling	National	Adults

**301**	Zhao Y	30	2021	China	UMIC	WPR	ECLIA	CSS	no random sampling	Community	Older
**302**	Zhen D	36	2015	China	UMIC	WPR	ELISA	CSS	random sampling	Community	Older
**303**	Zhou SJ	-35	2015	Australia	HIC	WPR	RIA	CSS	stratified random sampling	Community	Children
**304**	Zhu W	31	2018	China	UMIC	WPR	ELISA	CSS	cluster sampling	Community	Adults
**305**	Zhu Z	30	2012	China	UMIC	WPR	ELISA	CSS	no random sampling	Check-up	Children
**305**	Zhu Z	30	2012	China	UMIC	WPR	ELISA	CSS	no random sampling	Check-up	Children
**305**	Zhu Z	30	2012	China	UMIC	WPR	ELISA	CSS	no random sampling	Check-up	Children
**306**	Middelkoop K	-34	2022	South Africa	UMIC	AR	LC-MS/MS	CSS	no random sampling	Community	Children
**306**	Middelkoop K	-34	2022	South Africa	UMIC	AR	LC-MS/MS	CSS	no random sampling	Community	Children
**306**	Middelkoop K	-34	2022	South Africa	UMIC	AR	LC-MS/MS	CSS	no random sampling	Community	Children
**306**	Middelkoop K	-34	2022	South Africa	UMIC	AR	LC-MS/MS	CSS	no random sampling	Community	Children
**306**	Middelkoop K	-34	2022	South Africa	UMIC	AR	LC-MS/MS	CSS	no random sampling	Community	Children

**306**	Middelkoop K	-34	2022	South Africa	UMIC	AR	LC-MS/MS	CSS	no random sampling	Community	Children
**307**	Lin L	19	2022	China	UMIC	WPR	ECLIA	CSS	random sampling	Community	Older
**307**	Lin L	19	2022	China	UMIC	WPR	ECLIA	CSS	random sampling	Community	Adults
**307**	Lin L	19	2022	China	UMIC	WPR	ECLIA	CSS	random sampling	Community	Adults
**308**	Chakrabarty S	28	2022	India	LMIC	SEAR	CNNS	CSS	random sampling	Community	adolescents
**308**	Chakrabarty S	28	2022	India	LMIC	SEAR	CNNS	CSS	random sampling	Community	adolescents

Latitude: the unit is degrees; positive value indicates north latitude; negative value indicates south latitude. HIC: High-income countries; UMIC: Upper-middle-income countries; LMIC: Lower-middle-income countries; LIC: Low-income countries; EMR: the Eastern Mediterranean region; WPR: the Western Pacific region; ER: the European region; SEAR: the South-East Asia region; RA: the region of the Americas; AR: the African region; ELISA: enzyme-linked immunosorbent assays; RIA: radioimmunoassays; ECLIA: electrochemiluminescence immunoassays; CLIA: chemiluminescent assays; LC-MS/MS: HPLC: chemical assays. Chemical assays included high-performance liquid chromatography; CSS: cross sectional study.

Supplementary Table 6 The extracted data from the included studies

Order	First author	Year of publication	Country	Age(years) mean(sd)	Age range	No. of total	No. of female	No. of serum 25 (OH)D levels of <30 nmol/L	No. of serum 25 (OH)D levels of <50 nmol/L	No. of serum 25 (OH)D levels of <75 nmol/L	Time of data collection (year)
**1**	Abdulrahman MA	2022	Iraq	NA	>45	63	NA	13	34	45	2020
**1**	Abdulrahman MA	2022	Iraq	NA	16–45	278	NA	50	112	161	2020
**1**	Abdulrahman MA	2022	Iraq	NA	16–19	50	NA	16	30	40	2020
**2**	Abiaka C	2013	Oman	28.8(8.9)	18–55	206	105	80	180	202	2010
**3**	Abu-Samak MS	2019	Jordan	27.4(10.1)	17–52	371	267			338	2015–2017
**4**	Al Hayek S	2018	Lebanon	42.6(11.5)	20–74	344	172	33	108	214	2016
**5**	Al Shaikh A	2020	Saudi Arabia	14.7(2.6)	6–19	3613	1867	1687	3289		2015–2016
**6**	Al Shaikh AM	2016	Saudi Arabia	NA	13–15	1906	NA	899	1829		2013–2014
**6**	Al Shaikh AM	2016	Saudi Arabia	NA	6–12	204	NA	61	184		2013–2014
**7**	Al Zarooni AAR	2019	United Arab Emirates	38.5	18–106	12346	7785		10314	11765	2011–2012
**8**	Al-Daghri NM	2015	Saudi Arabia	NA	18–50	830	462	235	619		2013
**8**	Al-Daghri NM	2015	Saudi Arabia	NA	13–17	2225	1038	718	1901		2013
**9**	Al-Daghri NM	2016	Saudi Arabia	31.4(0.3)	18–56	561	326	150	408		2013
**9**	Al-Daghri NM	2016	Saudi Arabia	14.6(0.1)	10–17	808	345	276	711		2013
**10**	Al-Daghri NM	2021	Saudi Arabia	38.9(12.1)	>18	4247	NA	1082	2780		2008–2017
**10**	Al-Daghri NM	2021	Saudi Arabia	14.2(1.8)	<18	3111	NA	1083	2609		2008–2017

**11**	Aleteng Q	2017	China	64.2(9.9)	>45	1829	984		1105	1659	2010–2011
**12**	AlFaris NA	2019	Saudi Arabia	NA	30–65	166	166		100		2015–2016
**13**	Alkerwi A	2015	Luxembourg	NA	18–69	1335	NA	207	746	1107	2007–2008
**14**	Al-Kindi MK	2011	Oman	29.0(6.0)	18–45	100	100	51	100		2006
**15**	Alloubani A	2019	Saudi Arabia	NA	18–60	350	350		266		2017
**16**	AlQuaiz AM	2018	Saudi Arabia	42.9(10.8)	30–75	2832	1870	729	1686	2215	2014–2015
**17**	Al-Saleh Y	2015	Saudi Arabia	15.1(2.1)	13–17	2226	1038	718	1187		NA
**18**	Al-Taiar A	2018	Kuwait	12.5(0.9)	11–16	1416	722	559	1140	1365	2016
**19**	Alyahya K	2014	Kuwait	15.4(1.7)	10–18	232	232	169	229		NA
**20**	Alyahya KO	2020	Kuwait	27.0(6.2)	19–47	104	104	88	96		2011–2012
**21**	Andersen R	2005	Denmark,<br>Finland,<br>Ireland,<br>Poland.	71.8(1.4)	NA	221	all	17	67		2002
**21**	Andersen R	2005	Denmark,<br>Finland,<br>Ireland,<br>Poland.	12.6(0.5)	NA	199	all	37	92		2002
**22**	Andersen R	2008	Denmark	NA	10–15	37	all	30	35		2002
**22**	Andersen R	2008	Denmark	NA	18–64	219	95	158	201		2002
**23**	Andersen R	2013	Denmark	71.6 (1.4)	70–75	52	all	8	25		2013
**23**	Andersen R	2013	Denmark	12.5(0.5)	11–13	54	all	13	47		2013
**24**	Andersen S	2013	Denmark	NA	30–49	97	49		68		2001–2002
**25**	Arabi A	2021	Lebanon	45.3(15.0)	>18	466	295	182	335	427	2014

**26**	Ardawi MS	2011	Saudi Arabia	50.9(12.6)	20–79	1172	1172	534	934	1034	2008–2009
**27**	Ardawi MS	2012	Saudi Arabia	42.1(13.9)	20–74	843	0	264	429	510	2008–2009
**28**	Arnljots R	2017	Sweden	86.0(6.9)	56–102	545	370	224	448	510	2012
**29**	Arya V	2004	India	34.2(6.7)	24–53	92	67	44			NA
**30**	Asakura K	2020	Japan	NA	20–69	107	54	40	88		2018
**31**	Aspell N	2019	UK	66.4(8.8)	≥ 50	6004	3291	1423	3317		2012–2013
**32**	Aucoin M	2013	Canada	NA	2–19	756	NA	78	318	617	2005–2010
**32**	Aucoin M	2013	Canada	NA	20–45	461	NA	96	279	405	2005–2010
**33**	Bachhel R	2015	India	36.0	17–68	150	102	60		135	NA
**34**	Bater J	2021	Mongolia	9.4(1.6)	6–13	9595	4764	5692			NA
**35**	Batieha A	2011	Jordan	41.9(13.4)	>18	4590	3462		192	991	2009
**36**	Beer RJ	2020	Colombia	NA	2–5	6813	NA	129	1887		2015–2016
**36**	Beer RJ	2020	Colombia	NA	5–13	16454	NA	395	3587		2015–2016
**36**	Beer RJ	2020	Colombia	NA	18-50	7170	all	265	1706		2015–2016
**36**	Beer RJ	2020	Colombia	NA	13–18	6470	NA	194	1346		2015–2016
**37**	Bener A	2009	Qatar	10.4(3.5)	1–16	458	230		315		2007–2008
**38**	Benjeddou K	2019	Morocco	NA	7–9	239	NA		114		2011
**39**	Bettencourt A	2018	Portugal	43.1(12.1)	18–67	198	95		95	155	2015–2016
**40**	Bezrati I	2016	Tunisia	NA	7–16	225	0	92	191		2014
**41**	Bhatt SP	2014	India	40.2(7.9)	18–60	137	63	9		120	NA
**42**	Bhattoa HP	2013	Hungary	NA	≥50	206	0			109	2009–2010
**43**	Bi X	2016	Singapore	31.5(12.4)	>21	114	55		48		2013
**44**	Bjarnadottir A	2014	Iceland	NA	7–9	158	85	5	103		2006
**45**	Black LJ	2021	Australia	NA	≥65	331	191	16	96	245	2011–2013
**45**	Black LJ	2021	Australia	NA	25–34	652	436	43	172	440	2011–2013
**45**	Black LJ	2021	Australia	NA	35–44	730	433	26	188	540	2011–2013

**45**	Black LJ	2021	Australia	NA	45–54	648	386	30	187	450	2011–2013
**45**	Black LJ	2021	Australia	NA	55–64	490	263	23	147	347	2011–2013
**46**	Bodin J	2019	Ethiopia	NA	2–5	95	48		85		2014
**47**	Bolland MJ	2006	New Zealand	57.0(11.0)	40–88	378	0	2	34		2004–2005
**48**	Borissova AM	2013	Bulgaria	NA	20–80	2016	1068	430	1528		2012
**49**	Brinkmann K	2015	Chile	9.6(0.5)	NA	108	54	67	104	107	2013
**50**	Byun EJ	2017	Republic of Korea	14.4(0.1)	10–18	2515	1201		1843		2008–2011
**51**	Cabral MA	2013	Brazil	69.4(6.5)	≥60	234	0		74	156	2010–2011
**52**	Cairncross CT	2017	New Zealand	NA	2–5	1329	648	86	642	1183	2012
**53**	Capuano R	2021	Italy	NA	25–74	1200	600	137	686	1053	2018–2019
**54**	Carrillo-Vega MF	2017	Mexico	69.6(7.7)	≥60	1128	578		416		2012
**55**	Cashman KD	2013	Ireland	NA	18–84	1132	NA	76	453		2008–2010
**56**	Ceccarelli M	2020	Italy	5.6(3.1)	1–18	2140	851	209	939	1657	2008–2015
**57**	Chailurkit LO	2011	Thailand	40.3(0.3)	18–44	2641	1320		151	1194	2008–2009
**58**	Chao YS	2013	Canada	NA	≥50	6101	3180			2896	2007–2012
**59**	Chao YS	2014	Canada	NA	≥50	1493	971	13	129		2012–2013
**60**	Chen J	2017	China	67.5(65.4)	>60	6014	3066	731	2491		2010–2013
**61**	Chin KY	2014	Malaysia	39.4(17.0)	>20	150	0	2	51		2009
**62**	Chirita-Emandi A	2015	Romania	39.5(22.1)	1–85	6631	NA	265	1731	3926	2012–2014
**63**	Chlebna-Sokół D	2019	Poland	NA	9–13	720	409	145	606		2011
**64**	Choi HR	2017	Republic of Korea	71.6	>60	651	382	110		626	2008–2011
**65**	Chung IH	2014	Republic of Korea	9.1(2.3)	4–15	1212	338		710	1177	2012–2013

**66**	Chung JY	2013	Republic of Korea	NA	20–85	18305	10348		11587	16973	2008–2010
**67**	Cinar N	2014	Turkey	34.1(7.4)	21–52	118	65		99	114	2008
**68**	Contreras-Manzano A	2021	Mexico	NA	20–29	477	all		150	367	2018–2019
**68**	Contreras-Manzano A	2021	Mexico	NA	30–39	381	all		120	287	2018–2019
**68**	Contreras-Manzano A	2021	Mexico	NA	40–49	404	all		127	324	2018–2019
**69**	Cougnard-Grégoire A	2015	France	72.7(4.4)	≥65	697	433	190	580		2001
**70**	Courraud J	2020	Denmark	NA	6–18	177	81	31	121		2007–2008
**71**	Crowe FL	2019	UK	NA	≥65	993895	567649	299730			2005–2015
**71**	Crowe FL	2019	UK	NA	18–24	1269812	650233	536113			2005–2015
**71**	Crowe FL	2019	UK	NA	25–34	1316390	677677	531681			2005–2015
**71**	Crowe FL	2019	UK	NA	35–44	1180086	567154	443448			2005–2015
**71**	Crowe FL	2019	UK	NA	45–54	901352	436154	300265			2005–2015
**71**	Crowe FL	2019	UK	NA	55–64	755174	376707	223186			2005–2015
**72**	Dalgård C	2010	Denmark	72.4(1.2)	70–74	669	327	124	359		
**73**	Daly RM	2012	Australia	NA	≥75	810	448	66	354	673	2000
**73**	Daly RM	2012	Australia	NA	65–74	1564	834	76	545	1235	2000
**73**	Daly RM	2012	Australia	NA	25–34	1393	803	46	308	868	2000
**73**	Daly RM	2012	Australia	NA	35–44	2556	1464	108	762	1824	2000
**73**	Daly RM	2012	Australia	NA	45–54	2884	1541	111	983	2202	2000
**73**	Daly RM	2012	Australia	NA	55–64	2011	1088	77	742	1598	2000
**74**	de Oliveira CL	2020	Brazil	NA	12–17	1152	703		298	777	2013–2014

**75**	Djennane M	2014	Algeria	NA	5–15	435	232	35	130	254	2010
**76**	Drali O	2021	Algeria	NA	2–5	621	NA	93	375	528	2014–2016
**77**	Duarte C	2020	Portugal	NA	≥ 18	3092	1995	706	2170	3000	2011–2013
**78**	El Hayek J	2010	Canada	4.4(0.9)	3–5	282	150	39		178	2007–2008
**79**	El Hayek J	2013	Canada	NA	2–5	508	263	3	54	257	2010–2011
**80**	El-Khateeb M	2019	Jordan	43.7(14.2)	>18	4056	2856		2815	3548	2017
**81**	Eloi M	2016	Brazil	NA	2–95	39004	NA		13222	27576	2010–2014
**82**	Fang F	2018	China	24.6(3.9)	>18	1814	1052	139	949		2014
**83**	Fayet-Moore F	2019	Australia	36.0(9.0)	19–64	103	62		30		2010
**84**	Feketea GM	2021	Greece	7.6(4.9)	1–18	376	192		102	249	2018–2019
**85**	Feng X	2016	China	NA	60–89	686	376		359	572	2009–2010
**86**	Fernández Bustillo JM	2018	Spain	NA	5–15	153	89	9	101		2015–2016
**87**	Flores ME	2021	Mexico	NA	3–4	713	NA		208	520	2018–2019
**87**	Flores ME	2021	Mexico	NA	5–11	3482	1741		599	2612	2018–2019
**88**	Ganmaa D	2014	Mongolia	34.9(4.8)	18–44	420	420	333	415	419	2009
**89**	Gariballa S	2022	United Arab Emirates	38.0(12.0)	>18	648	491		286		NA
**90**	Gebreegziabher T	2013	Ethiopia	NA	22–38	196	all	29	165		2009
**91**	Gilbert-Diamond D	2010	Colombia	8.9(1.6)	5–12	479	250		208	430	2006
**92**	Gill TK	2014	Australia	NA	55–64	390	202		83	245	2008–2010
**92**	Gill TK	2014	Australia	NA	≥75	265	157		66	150	2008–2010
**92**	Gill TK	2014	Australia	NA	65–74	263	141		54	165	2008–2010
**92**	Gill TK	2014	Australia	NA	≥24	2413	1249	21	256		2008–2010
**92**	Gill TK	2014	Australia	NA	24–34	500	248		107	311	2008–2010

**92**	Gill TK	2014	Australia	NA	35–44	510	256		110	346	2008–2010
**92**	Gill TK	2014	Australia	NA	45–54	486	245		130	333	2008–2010
**93**	Ginter JK	2013	Canada	NA	60–90	224	185	3	27	87	2012
**94**	Glatt DU	2022	UK	NA	4–11	49	28	3	24		2019–2020
**95**	Gökta O	2020	Turkey	46.5(16.9)	>18	11893	9268		3454		2017–2018
**96**	Golbahar J	2014	Bahrain	33.7(10.1)	>18	500	250	247	432		2010–2011
**97**	González G	2007	Chile	63.7(9.7)	NA	90	all		52		
**98**	González-Gross M	2012	Europe	14.9(1.2)	3–18	1006	536		427	817	2006–2007
**99**	González-Molero I	2011	Spain	50.31(4.4)	20–83	1262	719		334	968	2002–2005
**100**	Gordon CM	2004	USA	14.7(2.0)	11–18	307	200		129		2001–2003
**101**	Goswami R	2009	India	33.7(13.5)	15–60	642	244	559			2006–2007
**102**	Granlund L	2016	Sweden	40.4(10.9)	25–65	216	111	26	157	208	2009–2010
**103**	Greene-Finestone LS	2011	Canada	NA	≥70	312	182	8	61	185	2005–2007
**103**	Greene-Finestone LS	2011	Canada	NA	35–50	823	422	19	155	464	2005–2007
**103**	Greene-Finestone LS	2011	Canada	NA	51–70	777	393	18	174	484	2005–2007
**104**	Griffin TP	2020	Ireland	52.0(16.4)	≥ 18	15319	10979	2047	7610		2011– 2015
**105**	Gromova O	2020	Kazakhstan	43.0(12.0)	>18	1347	819	371	943	1232	2018
**106**	Guo S	2014	Australia	NA	18–24	100	54		34		2012–2013
**107**	Han B	2017	China	52.5(13.5)	>18	6597	3784		5496		2014
**108**	Hansen L	2018	Denmark	NA	2–17	527	263	79	321	482	2013–2014
**108**	Hansen L	2018	Denmark	NA	18–69	2565	1517	309	1281	2045	2013–2014
**109**	Harinarayan CV	2007	India	43.0(1.0)	>18	913	572		159	328	2008

**110**	Harkness LS	2005	USA	NA	12–18	370	all		200		2000–2002
**111**	Hashemipour S	2004	Iran	NA	20–69	1210	715	812			2001
**112**	Hatun S	2005	Turkey	14.8(0.6)	13–17	89	89	19	39		2001
**113**	Hazell TJ	2015	Lebanon	3.7(1.0)	2–6	488	244		57	203	2010–2011
**114**	Hekimsoy Z	2010	Turkey	45.1(17.3)	>20	391	272		293	347	2007
**115**	Herrick KA	2019	USA	NA	≥60	3267	NA	94	496		2011–2014
**115**	Herrick KA	2019	USA	NA	2–5	1438	NA	7	102		2011–2014
**115**	Herrick KA	2019	USA	NA	6–11	2060	NA	29	282		2011–2014
**115**	Herrick KA	2019	USA	NA	20–39	3564	NA	271	1119		2011–2014
**115**	Herrick KA	2019	USA	NA	40–59	3496	NA	199	849		2011–2014
**115**	Herrick KA	2019	USA	NA	12–19	2355	NA	113	648		2011–2014
**116**	Hintzpeter B	2008	Germany	NA	3–17	9001	4419	1797	5895	7911	2003–2006
**117**	Hirani V	2012	UK	NA	≥65	2070	1120	292	968	1788	2005
**118**	Hirani V	2013	Australia	NA	≥85	174	0	27	85	133	2005–2007
**118**	Hirani V	2013	Australia	NA	70–74	659	0	57	287	554	2005–2007
**118**	Hirani V	2013	Australia	NA	75–79	522	0	45	217	421	2005–2007
**118**	Hirani V	2013	Australia	NA	80–84	304	0	30	124	248	2005–2007
**119**	Hoge A	2015	Belgium	NA	20–69	915	464	58	405	719	2010–2012
**120**	Ho-Pham LT	2011	Vietnam	46.4(17.6)	18–87	637	432		15	240	2009
**121**	Horton-French K	2021	Australia	20.9(2.0)	18–24	400	214		112	257	2011–2013
**121**	Horton-French K	2021	Australia	14.4(2.6)	12–17	692	353		112	445	2011–2013
**122**	Houghton LA	2019	Kenya	NA	3–5	433	251		2		2013
**123**	Hovsepian S	2011	Iran	41.4	20–80	1111	868	231	522	725	2006–2007
**124**	Hribar M	2020	Slovenia	68.6(2.8)	65–74	155	79	36	98	131	2017–2018
**124**	Hribar M	2020	Slovenia	46.5(13.2)	18–64	125	73	31	72	104	2017–2018

**125**	Hussain T	2021	Afghanistan	NA	>18	151	151		89		2020–2021
**126**	Hutchings N	2022	Armenia	46.1(20.6)	>18	1206	1206	157	651		NA
**127**	Ikonen H	2021	Finland	46.0	46	3650	1599	92	857	2597	2012–2013
**128**	Isa H	2020	Bahrain	NA	1–16	531	261		416	496	2016
**129**	Islam MZ	2002	Bangladesh	26.0(4.0)	16–40	189	189	27			2001
**130**	Islam MZ	2008	Bangladesh	22·6(3·7)	18–38	244	244	31	172		NA
**131**	Jääskeläinen T	2017	Finland	56.0	≥30	4051	2240	17	281	3068	2011–2012
**132**	Janssen HC	2013	The Netherlands	60.2(11.3)	40–80	400	0		144	276	2001–2002
**133**	Jayashri R	2020	India	46.0(12.0)	20–80	1500	668		823	1360	2012–2013
**134**	Jayatissa R	2019	Sri Lanka	14.0(2.0)	10–18	2525	1347	333	1484		2017
**135**	Jiang W	2020	China	37.9	18–65	14302	11299	1197	7185	11874	2014–2017
**136**	Johnson MA	2008	USA	NA	≥80	317	253		105		2002–2005
**137**	Jolliffe DA	2016	UK	72.0 (9.2)	48-94	222	133	55	144	205	2010–2012
**138**	Jorde R	2010	Norway	NA	21–70	2668	1737	157	1084		2008
**139**	Joukar F	2020	Iran	51.5(8.9)	35–70	5096	5096	1189	2729		2014–2017
**140**	Junaid K	2015	Pakistan	NA	15–45	215	215		156		2012
**141**	Kaddam IM	2017	Saudi Arabia	NA	>20	2104	1033	912			2013–2014
**141**	Kaddam IM	2017	Saudi Arabia	NA	6–19	4035	2001	1979			2013–2014
**142**	Kagotho E	2018	Kenya	NA	18–65	253	NA		44	152	2015
**143**	Kapil U	2018	India	NA	12–18	848	428		618	767	2015–2016
**143**	Kapil U	2018	India	NA	6–11	374	187		307	356	2015–2016
**144**	Karagüzel G	2014	Turkey	14.6(1.9)	11–18	746	349	75	265	350	2010
**145**	Karin Z	2018	Croatia	6.0(0.4)	5–6	260	128	31	151	226	2017

**146**	Karonova T	2016	Russian Federation	NA	7–75	1664	1341		760	1384	2009–2013
**147**	Kaykhaei MA	2011	Iran	36.7(14.3)	20–88	993	562		846	940	2008
**148**	Khan AH	2012	Pakistan	31.9(8.0)	>18	305	305		275	292	2011
**149**	Kim SY	2020	Republic of Korea	39.4(7.2)	>18	157211	60439	10652	79950		2012–2017
**150**	Kim YS	2020	Republic of Korea	15.5(0.5)	12–18	2314	NA		1805		2012–2017
**151**	Klenk J	2013	Germany	75.5(6.57)	65–91	1418	579		228	291	2009–2010
**152**	Kouda K	2013	Japan	11.2(0.3)	11–12	400	203		26	198	2010–2011
**153**	Koyama S	2021	Japan	NA	12–13	492	245		138	486	2016
**154**	Kremer R	2009	USA	NA	16–22	90	all			53	
**155**	Kull M Jr	2009	Estonia	48.9(12.2)	25–70	367	200		268		2006
**156**	Kunz C	2018	Germany	NA	11–13	528	NA	106	325	469	2009–2014
**156**	Kunz C	2018	Germany	NA	3–6	327	NA	63	205	241	2009–2014
**156**	Kunz C	2018	Germany	NA	7–10	520	NA	112	318	453	2009–2014
**156**	Kunz C	2018	Germany	NA	14–17	456	NA	111	283	400	2009–2014
**157**	Laird E	2018	Ireland	62.9	50–98	5356	2860	728	2303		2009–2011
**158**	Langlois K	2010	Canada	NA	6–79	5306	2740	218		3428	2007–2009
**159**	Lappe JM	2006	USA	66.7(7.3)	≥50	1179	all		170		2000–2001
**160**	Lardner E	2011	Ireland	61.0	40–85	143	all	14	67		2006–2007
**161**	Lategan R	2016	South Africa	44.3(10.6)	25–64	339	263	1	14		2015
**162**	Le Goaziou MF	2011	France	33.4(7.7)	19–49	196	all	105	158	188	2008
**163**	Lee J	2021	Republic of Korea	37.7(6.6)	>37	68457	31698		50995		2015
**164**	Leung RY	2017	Hong kong	NA	>20	5276	3920		2309	4752	1995–2010

**165**	Li H	2020	China	NA	6–11	4989	NA	1020	3907		2013–2015
**165**	Li H	2020	China	NA	6–11	5707	NA	1020	4078		2013–2015
**166**	Li L	2020	China	NA	18–75	2317	1136		806	1683	2018–2019
**167**	Li S	2014	China	62.2(6.1)	>40	578	578		417		2008
**168**	Lima-Costa MF	2020	Brazil	NA	≥50	2264	1200	37	362	1404	2015–2016
**169**	Lin LY	2021	UK	NA	40–69	448601	240263	60687	248478		2006–2010
**170**	Liu X	2018	USA	NA	≥60	8182	NA		2753	6041	2001–2010
**170**	Liu X	2018	USA	NA	18–39	10343	NA		4200	7985	2001–2010
**170**	Liu X	2018	USA	NA	40–59	7485	NA		2811	5785	2001–2010
**171**	Liu X	2020	Macao	NA	>55	207	108		76		2011
**172**	Lopes JB	2009	Brazil	72.1(4.4)	≥65	415	all			363	2005–2007
**173**	Lucas JA	2005	New Zealand	73.7(4.3)	≥55	1606	all	92	781		2000
**174**	Madsen KH	2014	Denmark	NA	4–17	340	178		24	197	2010
**174**	Madsen KH	2014	Denmark	NA	18–60	415	208	4	41	211	2010
**175**	Maguire JL	2011	Canada	NA	2	91	NA	1	29	75	2007–2008
**176**	Marzban M	2021	Iran	46±14	23–94	1806	1175		505		NA
**177**	Majumdar V	2011	India	39.7(12.8)	18–75	441	204		289		NA
**178**	Maldonado G	2017	Ecuador	54.7(16.6)	NA	269	228	3	47	188	2015–2016
**179**	Mallah EM	2011	Jordan	30.4(9.1)	>18	300	201	8	263	292	2010
**180**	Man PW	2016	The Netherlands	56.2(11.2)	≥18	416	313	40	236	332	2014
**181**	Manios Y	2017	Greece	11.2(0.7)	9–13	2353	1174	122	1235	2266	2007–2008
**182**	Mansbach JM	2009	USA	NA	2–5	1799	895	17	268	1131	2001–2006
**182**	Mansbach JM	2009	USA	NA	6–11	2759	1400	27	578	2013	2001–2006
**183**	Masoud MS	2020	Saudi Arabia	14.6(1.7)	10–17	170	100	117	170		NA

**184**	Matheï C	2013	Belgium	84.7(3.6)	≥80	367	234	129	248	323	2008–2009
**185**	Mechenro J	2018	India	NA	>60	48	NA	4	22		2015–2016
**186**	Mechenro J	2018	India	NA	18–45	275	NA	46	162		2015–2016
**186**	Meddeb N	2005	Tunisia	NA	20–60	389	261	66			2002
**186**	Mechenro J	2018	India	NA	45–60	101	NA	41	92		2015–2016
**187**	Mehboobali N	2015	Pakistan	32.5(10.7)	18–60	858	507		541	810	2009–2010
**188**	Metwally ASM	2021	Saudi Arabia	14.7(1.7)	13–17	1864	1315		1692		2019–2020
**189**	Meyer HE	2004	Norway	NA	45–75	869	NA		122		2000–2001
**190**	Meyer HE	2008	Sri Lanka	46.7(8.2)	30–60	196	111	7	89		2005
**191**	Miljkovic I	2011	Trinidad and Tobago	NA	≥65	424	0		12	114	2004–2007
**192**	Misra P	2017	India	37.7(11.7)	20–60	381	381		346		2013–2014
**193**	Mitchell DM	2012	USA	29.0(8.0)	18–50	634	288	45	245	407	2006–2008
**194**	Mogire RM	2021	Kenya, Uganda, Burkina Faso, Gambia, South Africa	NA	2–4	1507	NA		111	780	2002
**195**	Moreno-Reyes R	2009	Belgium	NA	40–60	401	200	135	306		2002–2005
**196**	Moussavi M	2005	Iran	16.2(3.1)	14–18	318	165		147		2004
**197**	Moy FM	2011	Malaysia	48.5(5.2)	>35	380	222		258		2010
**198**	Moy FM	2017	Malaysia	41.2	>20	770	770		557		2013
**199**	Muhairi SJ	2013	United Arab Emirates	16.0(0.6)	12–18	315	165		143		2010

**200**	Mutua AM	2020	Uganda	NA	5	302	156		8	113	2007
**201**	Nadeem S	2018	Pakistan	23.0(2.6)	19–25	221	191		197	213	NA
**202**	Naeem Z	2011	Saudi Arabia	40.8	19–72	180	97		53	121	2011–2012
**203**	Nakamura K	2008	Japan	63.5(5.8)	55–74	600	600	22	212		2006
**204**	Nakhaee S	2019	Iran	53.3(10.0)	40–91	400	212	115	296		NA
**205**	Nälsén C	2020	Sweden	50.1(16.8)	19–80	268	144	9	60		2010–2011
**206**	Nälsén C	2020	Sweden	11.3(0.5)	10–12	206	92	10	87		2014
**206**	Naqvi A	2017	Guatemala	NA	12–18	86	48		11	55	2014
**207**	NHANES(2015–2016)	2022	USA	NA	≥65	1231	624	23	170	474	2015–2016
**207**	NHANES(2015–2016)	2022	USA	NA	2–18	2618	1280	26	500	1833	2015–2016
**207**	NHANES(2015–2016)	2022	USA	NA	19–44	2413	1267	85	753	1730	2015–2016
**207**	NHANES (2015–2016)	2022	USA	NA	45–64	1777	912	37	332	992	2015–2016
**208**	NHANES(2017–2018)	2022	USA	NA	≥65	1321	no	12	127	439	2015–2016
**208**	Ní Chaoimh C	2018	Ireland	2.0	2	741	NA	12	198	530	2011
**208**	NHANES(2017–2018)	2022	USA	NA	<18	2184	no	37	415	1500	2015–2016
**208**	NHANES(2017–2018)	2022	USA	NA	18–44	2094	no	80	633	1533	2015–2016
**208**	NHANES(2017–2018)	2022	USA	NA	44–64	1810	no	43	336	950	2015–2016
**209**	Niafar M	2009	Iran	63.4(4.6)	53–80	300	300	115	184		2008

**210**	Nichols EK	2015	Jordan	NA	1–6	915	443	180	517		2010
**211**	Nielsen NO	2014	Denmark	NA	18–95	2877	1621	525	1626		2005–2010
**212**	Nikooyeh B	2017	Iran	38.4(8.5)	19–60	1406	751	974	1318		2014
**213**	Nimitphong H	2013	Thailand	39.9(0.4)	25–54	1990	541		433		NA
**213**	Nikooyeh B	2017	Iran	10.8(3.8)	5–18	667	345	374	622		2014
**214**	Oberg J	2014	Norway	16.1(0.5)	15–18	890	415	147	536	780	2010–2011
**215**	Oliveri B	2004	Argentina	71.3(5.2)	≥65	339	226	35	229	307	2001
**216**	Orces CH	2015	Ecuador	NA	70–79	724	NA		162	510	2009
**217**	Orces CH	2015	Ecuador	NA	60–69	1158	NA		211	750	2009
**217**	Orces CH	2015	Ecuador	NA	≥80	403	NA		115	277	2009
**217**	Orwoll E	2009	USA	73.8(5.9)	65–99	1606	NA	47	413	1142	2000–2002
**218**	Öztürk ZA	2017	Turkey	43.6(15.9)	18–90	1161	363	150	877	1102	2016–2017
**219**	Pan T	2018	India	56.9 (8.9)	>40	194	194		38	137	2017
**220**	Patel JV	2013	UK	NA	≥45	1904	916	1283	1732		2006–2009
**221**	Paul TV	2008	India	60.1(5.0)	50–80	150	150	15	74		NA
**222**	Penrose K	2012	USA	NA	NA	2160	1266		929	1685	2007–2009
**223**	Pérez-Llamas F	2008	Spain	77.4(8.1)	65–94	86	57	28	50		2005–2006
**224**	Perna L	2012	Germany	NA	50–74	5386	all	844	3677		2000–2002
**225**	Peters BS	2009	Brazil	NA	16–20	136	72			84	2006
**226**	Petrenya N	2020	Norway	56.5(8.4)	40–69	4465	2424	31	1103	3313	2012–2014
**227**	Qorbani M	2021	Iran	12.2(3.0)	7–18	2596	1166	276		1846	NA
**228**	Rabenberg M	2015	Germany	NA	65–79	1816	906	540	1201	1687	2008–2011
**228**	Rabenberg M	2015	Germany	NA	18–44	2456	1277	756	1439	2054	2008–2011
**228**	Rabenberg M	2015	Germany	NA	45–64	2723	1452	804	1700	2465	2008–2011
**229**	Rabufetti A	2019	Switzerland	NA	18,19	1045	0	24	179		2014–2016

**230**	Rafraf M	2014	Iran	15.9(1.0)	14–17	216	216		207	215	2012
**231**	Rahmadhani R	2017	Malaysia	13.0	13	1011	576		410		2013–2015
**232**	Rahman A	2020	Kuwait	12.4(0.9)	11–16	410	203		352	393	2015
**233**	Ramakrishnan S	2011	India	19.4(1.5)	18–25	237	156		74	117	2007–2008
**234**	Raposo L	2017	Portugal	53.0(19.3)	NA	500	286	188	428		2007–2009
**235**	Riverin B	2013	Canada	NA	15–91	944	538	54	457	834	2005–2009
**236**	Riverin B	2014	Canada	NA	8–14	52	25	3	22	41	2007
**237**	Robinson PJ	2013	Australia	NA	≥80	267	all	37	164	238	2008–2009
**237**	Robinson PJ	2013	Australia	NA	70–79	640	all	74	337	562	2008–2009
**238**	Rodríguez-Rodríguez E	2011	Spain	NA	9–13	102	NA	8	52		2007–2008
**239**	Saeed BQ	2021	United Arab Emirates	19.9(1.6)	18–24	287	189	48		244	2020
**240**	Saki F	2017	Iran	13.08(2.7)	9–18	477	263	63	388	458	2011
**241**	Sakyi SA	2021	Ghana	27.9(8.9)	17–55	500	NA		218		
**242**	Saliba W	2012	Israel	NA	>20	192240	139446	27676	96055	160952	2008–2009
**242**	Saliba W	2012	Israel	NA	0–19	6518	3728	891	3014	5237	2008–2009
**243**	Samefors M	2014	Sweden	NA	≥65	333	226	48	267	318	2007–2011
**244**	Santos A	2017	Portugal	NA	≥65	1500	872	594	1035		2015–2016
**245**	Santos BR	2012	Brazil	13.0(1.9)	7–18	234	all		85	212	2008–2011
**246**	Santos BR	2019	Brazil	53.4(9.4)	20–72	443	all		176		2005–2012
**247**	Sarafin K	2015	Canada	NA	9–79	11336	NA	839	4172		2007–2011
**248**	Saraiva GL	2005	Brazil	NA	≥65	214	148	33	123		2000–2001
**249**	Scalco R	2008	Brazil	77.8(9.0)	≥65	98	59	53	84		2005
**250**	Schramm S	2017	Germany	59.7(7.8)	45–75	4149	2091	660	2104	3456	2000–2003
**251**	Science M	2017	Canada	9.3(3.4)	3–15	743	390	4	152	565	2008–2009

**252**	Seo JA	2013	Republic of Korea	56.8(7.3)	40–69	1081	736		856		2009–2010
**253**	Shady MM	2015	Egypt	10.4(0.6)	9–11	200	102		23		
**254**	Sharawat IK	2019	India	7.0	6–11	100	45	29	63		2011
**255**	Shchubelka K	2020	Ukraine	NA	NA	1639	NA	211	847		2019
**256**	Sheikh A	2012	Pakistan	48.0	38–55	300	106		173	253	2011
**257**	Sherchand O	2018	Nepal	38.3(10.2)	>18	300	191		154	236	2017
**258**	Sherief LM	2021	Egypt	17.6(0.7)	14–18	572	302		542	566	2018–2019
**259**	Shetty S	2014	India	58.0(11.8)	>50	252	0	18	133		NA
**260**	Shivane VK	2011	India	30.4(3.6)	25–35	1137	579	220	805	1055	2004
**261**	Sioen I	2012	Belgium	8.1(1.5)	4–11	357	173	18	207	350	2010
**262**	Skull SA	2003	Australia	NA	≥16	116	72	61	107		2000
**263**	Smith G	2016	Cambodia	NA	>60	128	128	7	25	54	2014
**263**	Smith G	2016	Cambodia	NA	6–11	41	41		3	4	2014
**263**	Smith G	2016	Cambodia	NA	24–59	495	495	13	64	185	2014
**264**	Smith N	2021	UK	46.4(6.8)	35–59	148	all	19	89		2007–2010
**265**	Sochorová L	2018	Czech Republic	NA	5, 9	419	185	101	252		2016–2017
**266**	Sokolovic S	2017	Bosnia and Herzegovina	NA	≥60	735	NA	235	423	659	2013–2014
**266**	Sokolovic S	2017	Bosnia and Herzegovina	NA	18–39	450	NA	93	179	263	2013–2014
**266**	Sokolovic S	2017	Bosnia and Herzegovina	NA	40–59	645	NA	185	357	513	2013–2014
**267**	Solis-Urra P	2019	Chile	NA	≥65	686	NA	181	445		2016–2017

**267**	Solis-Urra P	2019	Chile	NA	18–64	1245	NA	204	642		2016–2017
**268**	Song HR	2014	Republic of Korea	65.1(8.1)	>50	8976	5389		6802	8720	2007–2010
**269**	Souberbielle JC	2016	France	39.7(18.6)	18–89	892	429	56	309	716	2011–2012
**270**	Srimani S	2017	India	NA	45–70	222	222		49	167	2014–2016
**271**	Sulimani RA	2016	Saudi Arabia	16.1(1.8)	12–18	1618	1618	645	1191		2011–2014
**272**	Suryanarayana P	2018	India	66.7(0.3)	>45	298	104		161		2014–2015
**273**	Tangoh DA	2018	Cameroon	NA	35–85	372	260		12	96	2015
**274**	Ten Haaf DSM	2019	The Netherlands	71.9(6.8)	65–93	450	99		9	108	2015–2016
**275**	Thuesen B	2012	Denmark	NA	30–60	6146	NA	848	3208		2001
**276**	Tolppanen AM	2012	UK	NA	7–13	7560	3744	124	2158	5631	2001–2002
**277**	Tønnesen R	2016	Denmark	NA	18–25	700	339	135	373		2012–2014
**278**	Tran B	2013	Australia	NA	60–64	148	74	11	107		2010–2011
**278**	Tran B	2013	Australia	NA	65–69	148	73	11	106		2010–2011
**278**	Tran B	2013	Australia	NA	70–74	134	59	9	92		2010–2011
**278**	Tran B	2013	Australia	NA	75–79	131	58	15	102		2010–2011
**278**	Tran B	2013	Australia	NA	80–84	92	37	20	76		2010–2011
**279**	Tseng M	2009	USA	49.6(8.4)	35–69	194	0	66		192	2007
**280**	Unger MD	2010	Brazil	47.8(13.4)	18–90	603	485			473	2006
**281**	Uush T	2013	Mongolia	NA	1–6	524	NA	222			2010
**281**	Uush T	2013	Mongolia	NA	1–6	876	876	457			2010
**282**	Vallejo MS	2020	Chile	51.6(17.0)	18–89	1329	668				2016–2017
**283**	Vallianou N	2012	Greece	NA	≥35	490	NA		135	352	2009–2010
**284**	Vasudevan B	2021	India	NA	40–60	184	184		164		2018–2019

**285**	Vierucci F	2014	Italy	NA	10–21	427	214		213	351	2010–2012
**286**	von Hurst PR	2010	New Zealand	40.6(10.3)	≥20	228	all	98	191		2007
**287**	Voortman T	2015	The Netherlands	NA	6	4167	0	258	1241	3020	2006–2012
**288**	Vupputuri MR	2006	India	43.3(9.7)	>18	105	54		99		NA
**289**	Wakayo T	2015	Ethiopia	NA	11–18	174	99		73		2013
**290**	Ward M	2011	UK	NA	≥45	6154	NA		3107		2002–2004
**291**	White Z	2019	South Africa	NA	7–9	59	NA		4	39	2016
**292**	Wyskida M	2018	Poland	NA	≥65	3472	1658	355		3002	2007–2011
**293**	Yan X	2019	China	NA	>18	302	126	81	216		2017
**294**	Yang K	2020	China	42.9	>18	1982	970		1681	1893	2015–2016
**295**	Yousef S	2021	Canada	NA	≥64	1361	NA	63	305	799	2012–2015
**295**	Yousef S	2021	Canada	NA	3–5	267	NA	5	42	179	2012–2015
**295**	Yousef S	2021	Canada	NA	6–11	932	NA	38	231	727	2012–2015
**295**	Yousef S	2021	Canada	NA	18–64	8161	NA	988	3248	6386	2012–2015
**295**	Yousef S	2021	Canada	NA	12–17	858	NA	90	346	723	2012–2015
**296**	Yu L	2020	China	NA	1–3	1200	NA		40		2017–2020
**296**	Yu L	2020	China	NA	3–6	1200	NA		87		2017–2020
**297**	Yu S	2015	China	NA	>18	2173	1096	128	1215		2013
**298**	Zargar AH	2007	India	28.8(4.9)	18–40	92	28		76		2003
**299**	Zgaga L	2011	UK	61.3(10.5)	21–82	2235	988	772	1732		2006
**300**	Zhang FF	2016	Kuwait	43.6(14.6)	>20	960	524	348	795		2008–2009
**301**	Zhao Y	2021	China	67.7(6.0)	60–95	2661	1571		1677	2523	2018
**302**	Zhen D	2015	China	NA	40–75	10100	7158		7544		NA
**303**	Zhou SJ	2015	Australia	NA	1–5	221	105	9	44		2005–2007
**304**	Zhu W	2018	China	NA	18–74	508	344		254	227	2012–2014

**305**	Zhu Z	2012	China	NA	12–16	183	48	6	85	164	2008–2011
**305**	Zhu Z	2012	China	NA	2–5	2269	815	26	498	1555	2008–2011
**305**	Zhu Z	2012	China	NA	6–11	1440	421	29	582	1271	2008–2011
**306**	Middelkoop K	2022	South Africa	6	6	168	no		4		2017–2021
**306**	Middelkoop K	2022	South Africa	7	7	281	no		8		2017–2021
**306**	Middelkoop K	2022	South Africa	8	8	346	no		18		2017–2021
**306**	Middelkoop K	2022	South Africa	9	9	503	no		35		2017–2021
**306**	Middelkoop K	2022	South Africa	10	10	396	no		48		2017–2021
**306**	Middelkoop K	2022	South Africa	11	11	108	no		22		2017–2021
**307**	Lin L	2022	China	NA	≥65	353	223	1	6	61	2018–2020
**307**	Lin L	2022	China	NA	18–39	477	322	1	20	161	2018–2020
**307**	Lin L	2022	China	NA	40–59	860	538	1	22	173	2018–2020
**308**	Chakrabarty S	2022	India	no	10–14	6798	no	1608			2016–2018
**308**	Chakrabarty S	2022	India	no	15–19	5024	no	1166			2016–2018

SD: standard deviation; NO.: number.

Supplementary Table 7 The extracted data that are used to distinguish different studies

Order	First author	Year of publication	Country	Study participants or sits or population
**1**	Abdulrahman MA	2022	Iraq	This cross-sectional study was carried out at Central Laboratory, Duhok/Kurdistan Region of Iraq from December to March 2020. A total of 391 apparently healthy volunteers (219 female and 172 Male) with ages ranged between (18 to 70) years old were informed and interviewed about the nature of the study, then were asked to take part in the study and a written consent were obtained from them.
**2**	Abiaka C	2013	Oman	Oman is situated in the northern hemisphere, 21° north of the equator. The study was carried out from November to March 2010, when the average temperature is 25° C and skies are mostly clear and sunny. The study population, 206 healthy Omani volunteers aged 18–55 years (105 women [mean = 26.8 years] and 101 men [mean = 31.1 years]) was composed of university students, educators, administrators, office secretaries, and their relatives.
**3**	Abu-Samak MS	2019	Jordan	This was a prospective observational cohort study carried out at the Applied Science Private University (ASU), Amman, Jordan during the period from October 2015 to May 2017. To avoid some anticipated variations in the study sample, only male and female Jordanian ASU students and employees who live in Amman participated in the study.
**4**	Al Hayek S	2018	Lebanon	A cross-sectional study was carried out on Notre Dame University (NDU) employees, in the Zouk Mosbeh, North, and Shouf campuses.
**5**	Al Shaikh A	2020	Saudi Arabia	This cross-sectional study included 3.613 school children, aged 6-19 years, in Saudi Arabia. The study was conducted during the period from 2015 to 2016.
**6**	Al Shaikh AM	2016	Saudi Arabia	The study was approved by the King Abdullah International Medical Research Center (KAIMRC) and conducted during the years 2013-2014. It included 2110 apparently healthy male and female children (1013 male, 1097 female) aged 6-15 years. The subjects were recruited from primary, intermediate, and secondary schools of the Western, Central, and Eastern regions of Saudi Arabia.
**7**	Al Zarooni AAR	2019	United Arab Emirates	All adult participants ⩾ 18 years of age, who presented at Ambulatory Health Care clinic for Weqaya Screening Program between Octobers 2011 and November 2012, were recruited.
**8**	Al-Daghri NM	2015	Saudi Arabia	A total of 2225 apparently healthy Saudi adolescents (1187 boys and 1038 girls, aged 13–17 years old) and 830 apparently healthy adults (368 men and 462 women, aged

				18–50 years old) were recruited respectively from different public and private schools within Riyadh, Saudi Arabia from February to October, 2013.
**9**	Al-Daghri NM	2016	Saudi Arabia	For the present study, 30 schools were randomly chosen covering north, south, east, west and central Riyadh. The study included both Saudi students and adult Saudi staff working in the participating schools, including the teachers.The study started in February 2013 and was completed in April 2013. A total of 808 children (10-17 years old) and 561 adults (18-48 years old) who were not on vitamin D supplements and who provided fasting plasma samples were included.
**10**	Al-Daghri NM	2021	Saudi Arabia	The series of cross-sectional studies were conducted between January 2008 and December 2017 in central region of Saudi Arabia. Participants for this study were selected from multiple cohorts. The 2008–2010 database was taken from the project involving province wide biomarker screening in Riyadh (2008–2010; N = 1460), a project of King Saud University, Ministry of Health, and the Chair for Biomarkers of Chronic Diseases (CBCD) (previously the Biomarkers Research Program, BRP), where participants were randomly recruited from their homes [24,25]. Participants from 2011 to 2017 were also taken from multiple cohorts including the Osteoporosis Registry (2014–2017; N = 1225) [26], Gestational Diabetes Mellitus cohort (2014–2017, N = 281) [27], Vitamin D School Project (2011–2017; N = 3039) [28] and Prediabetes cohort (2012–2017; N = 1355) [29] master databases. Vitamin school study was a project of King Saud University, Ministry of Education and the CBCD, where they were recruited from different schools in Riyadh. Whereas, GDM, prediabetes and Osteoporosis registry were the project of the Chair for Biomarkers of Chronic Diseases (CBCD). The younger subjects (n = 3267) were from school surveys and biomaker screening in Riyadh region. The adults (n = 2480) subjects were from multiple cohorts including biomaker screening in Riyadh region, vitamin D school study, prediabetes project, gestational diabetes mellitus project and osteoporosis registry. The elderly (n = 1613) population were from biomarker screeing in Riyadh region, osteoporosis registry and prediabetes project.
**11**	Aleteng Q	2017	China	Consecutive numbering 2,223 males and females who were subjected to the vitamin D level detection, from May 2010 to June 2011, were initially screened.

**12**	AlFaris NA	2019	Saudi Arabia	This study is a cross-sectional study. One hundred and sixty-eight women were recruited to participate from the King Saud Medical City in Riyadh, Saudi Arabia during the period from May 2015 to June 2016.
**13**	Alkerwi A	2015	Luxembourg	The ORISCAV-LUX study was a nationwide population-based survey conducted in 2007–2008 to determine the prevalence of potentially modifiable cardiovascular disease risk factors in adult population resident in Luxembourg.
**14**	Al-Kindi MK	2011	Oman	Forty-one apparently healthy women aged 18–45 years with (mean ± standard deviation (SD), 29 ± 6 years), working in various departments of the Royal Hospital, Muscat, Oman, volunteered for this study.
**15**	Alloubani A	2019	Saudi Arabia	A descriptive, cross-sectional, and correlational design wereused in this study, a convenience sampling method of 350 malesand females participants, aged from 18 to 60 years in Tabuk city, inthe north-west coast of the Saudi Arabia
**16**	AlQuaiz AM	2018	Saudi Arabia	Saudi adults between 30 and 75 years of age and permanent residents of Riyadh were eligible to participate in the WISHES study
**17**	Al-Saleh Y	2015	Saudi Arabia	A total of 2226 apparently healthy Saudi students were recruited from randomly selected public secondary schools within Riyadh, Saudi Arabia, and consisted of 1188 boys (mean age, 15.1 ± 2.2 years) and 1038 girls (15.1 ± 2.0).
**18**	Al-Taiar A	2018	Kuwait	The study population comprised students between 11 and 16 years old in public middle schools from all governorates of Kuwait. A school-based, cross-sectional study was conducted on students from 12 public middle schools, which were selected using a stratified multistage cluster random sampling with a probability proportional to size.
**19**	Alyahya K	2014	Kuwait	This was a cross-sectional study, in which 232 healthy females(10–18 years) were recruited from public schools from each governorate in Kuwait.
**20**	Alyahya KO	2020	Kuwait	This observational, cross-sectional study was approved by the Joint Committee for the Protection of Human Subjects in Research by the Kuwait Institute for Medical Specialization, Ministry of Health and AbdulMihsin Al-Abdulrezzag Health Sciences Centre, Health Sciences Centre- Kuwait University. The study was carried out between December 2011 and March 2012.
**21**	Andersen R	2005	Denmark, Finland, Ireland, Poland.	The study is the baseline part of a 1-y long observational study involving four European countries (Denmark, Finland, Ireland and Poland). Two age groups, adolescent girls and elderly women, had S-25OHD measured in a standardised way during February and March 2002 in all four countries.

**22**	Andersen R	2008	Denmark	The study is the baseline part of a 1-year long double-blinded randomized placebo-controlled intervention study with two doses of vitamin D (10 and 20 mg/day). The subjects were adolescent girls (median age 12.2 years, range 10.1–14.7), pre-menopausal women (median age 36.2 years, range 18.1–52.7) and men (median age 38.3 years, range 17.9–63.5). All subjects were of Pakistani origin (immigrants or descendants with Pakistani parents) primarily living in the Copenhagen area, Denmark (551N).
**23**	Andersen R	2013	Denmark	The subjects were randomly selected from the Danish National Central Offices of Civil Registrations. Among a total of 3380 girls and 8671 women living in the Copenhagen and Frederiksberg municipality (55.41N), 235 girls (11–13 years) and 346 women (70–75 years) were randomly selected by use of date of birth and invited by letter to participate in the study
**24**	Andersen S	2013	Denmark	A random sample was drawn and participants in Ilulissat were stratified by age, sex and place of birth (Greenland or Denmark), aiming at a balanced representation of the age groups 30–39 and 40–49 years, men and women and the three groups consisting of subjects born in Greenland and living in the town, living in the settlement and subjects who were not born in Greenland.
**25**	Arabi A	2021	Lebanon	This is a secondary analysis of deidentified data generated from the bisphenol A (BPA) study. The original BPA study was a cross-sectional, community-based study where 501 community-dwelling adult men and women residing in the Greater Beirut area were randomly recruited between March and May 2014
**26**	Ardawi MS	2011	Saudi Arabia	Over a period of 12 months (June 2008-June 2009), a total of 2,369 Saudi women were prospectively recruited at random during a health survey from 40 primary health care centers (PHCCs) scattered around the city of Jeddah (divided into seven geographical sectors) to ensure that the average health status of the studied group will reflect a randomly selected adult population.
**27**	Ardawi MS	2012	Saudi Arabia	From January 2008 through December 2009, a total of 1,722 Saudi Arabian men were prospectively recruited at random during a health survey from 40 primary healthcare centers (PHCCs) scattered around the city of Jeddah (latitude of 21.7° N and longitude of 39.2° E, western part of Saudi Arabia) and agreed to participate in the study.
**28**	Arnljots R	2017	Sweden	During the first 3 months of 2012, a case report form was completed and blood samples collected from all included

				residents of 22 nursing homes in south-western Sweden (latitude 57.58° North to 57.82° North).
**29**	Arya V	2004	India	The study was carried out at Lucknow, which is geographically situated at 26.55 N, 80.59 E latitude. We studied 92 young healthy volunteers (67 females, 25 males). All the volunteers wereunselected hospital staff working at Sanjay Gandhi Post Graduate Institute of Medical Sciences, Lucknow, in different capacities
**30**	Asakura K	2020	Japan	We recruited apparently healthy adult volunteers aged 20 to 69 years from two areas of Japan, in cooperation with local governments.
**31**	Aspell N	2019	UK	The current study includes participants from the English Longitudinal Study of Ageing (ELSA), which is an ongoing nationally representative study of health. ELSA consists of men and women born on or after the 29 February 1952.
**32**	Aucoin M	2013	Canada	The program aims to screen refugees within weeks of their arrival to Calgary and cares for patients up to 2 years after their arrival. All charts were searched electronically between June 2005 and January 2010.
**33**	Bachhel R	2015	India	Healthy volunteers (N = 150) of either sex, belonging to different backgrounds, were randomly enrolled after ethical permission was obtained.
**34**	Bater J	2021	Mongolia	We conducted a cross-sectional analysis of baseline data collected from children attending 18 public schools (located in six districts of Ulaanbaatar) who were being screened for participation in a randomized, controlled trial of vitamin D supplementation for the prevention of latent Tuberculosis infection (LTBI)
**35**	Batieha A	2011	Jordan	A national population-based household sample was selected from the 12 governorates of Jordan. These 12 governorates belong to the 3 regions of the country, i.e. the north, middle, and south.
**36**	Beer RJ	2020	Colombia	Colombia is located at 4◦N and 72◦W; its climate is tropicaland isothermal with a generally dry, sunny season from Decemberto March. The third Colombian National Nutrition Survey[Encuesta Nacional de Situación Nutricional (ENSIN)]) wasconducted in 2015 and 2016 by the Colombian Institute of Family Welfare, the Ministry of Health and Social Protection, the Colombian National Institute of Health, the Administrative Department for Social Prosperity, and the National University of Colombia.
**37**	Bener A	2009	Qatar	A cross-sectional study carried out among children below 16 years of age who visited the Primary Health Care Centers (PHCs). The survey was conducted over a period from

				August 2007 to March 2008. Qatari nationals, male and female, aged below 16 years.
**38**	Benjeddou K	2019	Morocco	A total of 239 children aged between 7 and 9 years old were chosen to participate in the study.
**39**	Bettencourt A	2018	Portugal	The study was conducted in Porto (~41° N; elevation: 104 m), in July and August 2015 (summer time) and April 2016 (winter time). Two hundred healthy blood donors voluntarily participated in this study.
**40**	Bezrati I	2016	Tunisia	The study included 225 boys aged 716 years, recruited from two centers of a football academy in the area of Tunis (latitude, 358N).
**41**	Bhatt SP	2014	India	This cross-sectional population-based study involved 137 adults without diabetes (74 males, 63 females) and was conducted at the All India Institute of Medical Sciences and the Fortis Hospital, New Delhi, India, from April 2006 to April 2011.
**42**	Bhattoa HP	2013	Hungary	Men residing in Debrecen, Hungary (latitude, 47°29′25″; longitude, 21°36′39″; altitude, 107.9 m) were invited to participate in the study from September 2009 to September 2010. During this period, a total of 229 randomly selected volunteersagreed to participate.
**43**	Bi X	2016	Singapore	This study was conducted using a cross-sectional design which was a subsection of a larger study. A total of 114 healthy adults consisting of 59 males and 55 females were recruited from the general public in Singapore through advertisements and posters that were placed on website.
**44**	Bjarnadottir A	2014	Iceland	The study sample was obtained from an intervention study on the lifestyle of 7–9-year-old children aiming to increase physical activity and promote healthy diet in 2006–2008.
**45**	Black LJ	2021	Australia	The 2012-2013 AATSIHS comprises the National Aboriginal and Torres Strait Islander Health Survey (NATSIHS), the National Aboriginal and Torres Strait Islander Nutrition and Physical Activity Survey (NATSINPAS), and the National Aboriginal and Torres Strait Islander Health Measures Survey (NATSIHMS).
**46**	Bodin J	2019	Ethiopia	This study is a follow-up of a cross-sectional cohort study designed to investigate the prevalence of influenza and its effect on child health in a sub-tropical climate, Addis Ababa, Ethiopia (9°0′49.75″ N/38°42′21.49″ E). Ethiopian preschool children were invited to participate in a 12-month follow-up surveillance of health status from March 2014.
**47**	Bolland MJ	2006	New Zealand	Between January 2004 and May 2005, 378 healthy independent-living middle-aged or older men underwent biochemical assessment for a study of calcium

				supplementation. Participants were volunteers over 40 years of age who responded to newspaper advertisements.
**48**	Borissova AM	2013	Bulgaria	The study was designed as a multicenter cross-sectional population-based study and was carried out between January 3 and February 6, 2012 in vive regions (41°–44°N). The study encompassed 12 cities and towns with the adjacent smaller settlements.
**49**	Brinkmann K	2015	Chile	Se realizó un estudio de intervención abierto prospectivo de suplementación nutricional con VD3 en ninos ˜ y ninas ˜ de entre 8 y 10 anos ˜ de edad de 7 escuelas públicas dependientes de la Corporación Municipal de Punta Arenas.
**50**	Byun EJ	2017	Republic of Korea	This study was based on data acquired from the Korean National Health and Nutrition Examination Survey (KNHANES), a survey conducted by the Korea Centers for Disease Control and Prevention to provide nationally representative and reliable statistical data regarding the health, behaviour associated with health, nutrition and food intake status of the Korean population. Data were collected from 2008 to 2011, which corresponds to the second and third year of KNHANES IV (2007–2009) and the first and second year of KNHANES V (2010–2012).
**51**	Cabral MA	2013	Brazil	This was an analytical cross-sectional study, which involved 234 men aged over 60 years of age, randomly recruited from a basic care unit in the fifth health district (5th HD) of the city of Recife in the state of Pernambuco, during the Brazilian summer months, from October 2010 to January 2011.
**52**	Cairncross CT	2017	New Zealand	In this cross-sectional study, 1329 children aged 2 to <5 years were recruited from cities and towns from around New Zealand. Participants were recruited and tested during the 10-week period from August to October 2012 (late winter – early spring).
**53**	Capuano R	2021	Italy	Data presented in this study are included in the 2018–2019 cross-sectional phase of the Irno Valley Prevention Project (VIP) [36], a randomized epidemiological and primary prevention study. Between January 2018 and July 2019, 1200 adults (aged 25–74 years), 600 males and 600 females, representative of the Irno Valley (Campania region), an area of Southern Italy, were randomly included from the electoral lists of two municipalities (Mercato S. Severino and Baronissi), following the rules of the Monica Project–MONICA Cardiovascular Diseases

**54**	Carrillo-Vega MF	2017	Mexico	This was a cross-sectional analysis of the third (2012) wave of the Mexican Health and Aging Study (MHAS), which was a prospective panel study conducted in Mexico.
**55**	Cashman KD	2013	Ireland	The fieldwork phase of the National Adult Nutrition Survey (NANS) was carried out between October 2008 and April 2010
**56**	Ceccarelli M	2020	Italy	We retrospectively gathered data from 2140 children observed in five different NWGMC-ISP centers, variously located in Italy, during a period extending from January 2008 to December 2015.
**57**	Chailurkit LO	2011	Thailand	Subjects consisted of 2,641 adults, aged 15-98 years, randomly selected from the Thai 4th National Health Examination Survey (2008-9) cohort.
**58**	Chao YS	2013	Canada	A cross-sectional study using data from a health programme enrolling workers mostly from Northern Alberta, Canada.
**59**	Chao YS	2014	Canada	This is a cross-sectional study based on information gathered at baseline visits from volunteer participants before starting a wellness programme by the Pure North S'Energy Foundation (PN)
**60**	Chen J	2017	China	Data for the present analysis were extracted from the China National Nutrition and Health Survey (CNNHS), 2010–2013.
**61**	Chin KY	2014	Malaysia	The subjects of this study were participants who attended the health screening session of the Malaysian Aging Male Study from September 2009 to September 2011
**62**	Chirita-Emandi A	2015	Romania	This is a cross-sectional study, where a group of 6631 unique individuals had performed a total of 7544 vitamin D, 415 ionized calcium and 611 PTH assessments between 1st January 2012 and 30th August 2014 in a chain of private laboratories.
**63**	Chlebna-Sokół D	2019	Poland	This two-stage study (initial cross-sectional investigation in March and re-examination in October) was conducted nationally in 2011, and included 720 healthy Caucasian children (311 boys, 409 girls) aged 9.0–12.99 years, i.e., individuals attending grades 2–5 of public primary schools with similar physical education curricula. Children residing in six representative geographical locations in Poland were screened for inclusion criteria and enrolled to the survey.
**64**	Choi HR	2017	Republic of Korea	A cross-sectional study was conducted on 269 men and 382 women (mean age, 71.6 years) enrolled in the Korean Social Life, Health, and Aging Project (KSHAP), a population-based longitudinal study of health determinants in elderly Koreans.

**65**	Chung IH	2014	Republic of Korea	Participants were 1,212 children aged 4 to 15 years, who visited Bundang CHA Medical Center (located at 37°N) between March 2012 and February 2013.
**66**	Chung JY	2013	Republic of Korea	This study was based on data obtained from the 2008-2010 KNHANES, a nationally-representative survey conducted by the Korean Ministry of Health and Welfare.
**67**	Cinar N	2014	Turkey	This study was conducted in Ankara located at 39° 52' 30" N, 32° 52' E. The study consisted of 118 premenopausal women and men aged between 21 and 52 years-old.
**68**	Contreras-Manzano A	2021	Mexico	In 1262 women aged 20 to 49 years, the prevalence of VDD/IVD was estimated and the factors associated with it were evaluated with a multinomial regression model.
**69**	Cougnard-Grégoire A	2015	France	Antioxydants, Lipides Essentiels, Nutrition et maladies OculaiRes (ALIENOR) is a population-based study on eye diseases performed in elderly residents of Bordeaux, France.
**70**	Courraud J	2020	Denmark	This work is an ancillary study of a cross-sectional study that has already been published (published data included cardiovascular fitness and metabolic risk factors of the children)
**71**	Crowe FL	2019	UK	This study was an open cohort design using THIN primary care database that contains health records for >11 million patients from over 600 GPs in the UK.
**72**	Dalgård C	2010	Denmark	The present study is part of a larger study of cardiovascular and neurobehavioural effects of lifetime methylmercury exposure.
**73**	Daly RM	2012	Australia	The AusDiab study was a national population-based survey conducted in 1999–2000 to determine the prevalence of diabetes, obesity and other cardiovascular disease risk factors in Australian adults
**74**	de Oliveira CL	2020	Brazil	This tudy is part of the larger ERICA project, which is a multi-center, cross-sectional, school-based study.
**75**	Djennane M	2014	Algeria	Four hundred thirty-five children were evaluated and had a blood sample in September 2010. Among these 435 children, 408 were sampled again in March 2011. To be included in the study, all children had to be 5–15 years old and live and go to school in the Tizi-Ouzou urban area, a city of approximately 135,000 inhabitants situated in Northern Algeria at a latitude of 36° 43 min N. In practice, 10 of the 48 public primary schools, one of the 10 private primary schools, eight of the 19 public secondary schools, and one of the 8 private secondary schools of the city were randomly chosen.

**76**	Drali O	2021	Algeria	The target population comprised a series of healthy preschool children living in the municipality of Hussein Dey, which is located east of the province of Algiers.
**77**	Duarte C	2020	Portugal	Data and sera samples were collected in the context of EpiReumaPt, a nationwide health survey conducted between September 2011 and December 2013 to assess the prevalence of Rheumatic Diseases in Portuguese adults. A random sample of noninstitutionalized and living in private houses subjects with ⩾ 18 years of age was drawn.
**78**	El Hayek J	2010	Canada	s The sample consisted of Inuit preschool children (3–5 y of age) recruited in the late summer and early fall of 2007 (August– November) and 2008 (August–September) in 16 of the 25 communities of Nunavut, representing all 3 regions of the territory (Kivalliq, Baffin, and Kitikmeot). The communities were selected to be representative of latitude, region, and community size. Latitude of the communities ranged from 568 32’N to 728 40’N. I
**79**	El Hayek J	2013	Canada	Preschoolers (age 2–5 y) were studied between June 2010 and June 2011 in a random sample of licensed daycares (n = 77) in greater Montreal, Canada .
**80**	El-Khateeb M	2019	Jordan	This national cross-sectional study was conducted among Jordanian adults over a period of 4months between May and August 2017. A population-based household sample was selected from 12 governorates covering the three regions of the country; the north, middle, and south.
**81**	Eloi M	2016	Brazil	The study included 39,004 25(OH)D and 14,829 iPTH results, performed as routine care, for patients of both genders, from age 2 to 95 years old, living in São Paulo greater area, Brazil, from January 2010 through August 2014.
**82**	Fang F	2018	China	This study is an extension of a survey conducted in Tianjin, which is part of the BThyroid Disorders, Iodine Status, and Diabetes: A National Epidemiological Survey - 2014. Residents from both urban and rural areas of Tianjin were enrolled for the study and paired by sex and age.
**83**	Fayet-Moore F	2019	Australia	This study was conducted at the Nestlé Rhodes Head Office with employees based in Sydney, a city situated at 33.9 ° south in New South Wales, Australia.
**84**	Feketea GM	2021	Greece	We conducted a prospective 13-month study, from September 2018 through September 2019 in Amaliada, Greece. The participants in this study were recruited from the children who were addressed to our laboratory for their routine blood testing.

**85**	Feng X	2016	China	This was a cross-sectional study of northern Chinese elderly. A total of 686 subjects (310 men and 376 women) were screened by stratified random sampling from the participants in a communitybased osteoporosis prevention study. The study was conducted by the Health Centre at Qianfoshan Hospital of Shandong University from 2009 to 2010.
**86**	Fernández Bustillo JM	2018	Spain	This is a descriptive, observational and transversal study. A prospective recruitment of children aged up to 15 years during February 2015 to February 2016 was carried out. Children underwent a standard routine blood test in the Paediatrics unit of the Bertamiráns (Ames, A Coruña) primary care centre, located in north-west Spain (latitude 42°51′36″N, longitude 8°39′0″W). This study was approved by the “Primary Care Directorate” of SERGAS (the Galician regional health service).
**87**	Flores ME	2021	Mexico	Data and serum samples of child participants were collected in the Ensanut 2018-19. Data of 4 691 children aged 1-11 years were analyzed.
**88**	Ganmaa D	2014	Mongolia	Study participants were the mothers of school children attending two primary schools in Ulaanbaatar. Using schools as the recruitment sites provided a central location for data collection. Data were collected in two half-day sessions in March and April, 2009.
**89**	Gariballa S	2022	United Arab Emirates	Participants in this study includes Emirati (UAE citizens) and expatriates from other Arab countries aged 18 years and over. They were part of a trial to assess the clinical benefit of vitamin D3 supplements. Participants were recruited from community health centers and local hospitals.
**90**	Gebreegziabher T	2013	Ethiopia	Data were collected in July 2009 from 202 nonpregnant women living in three adjacent communities in rural southern Ethiopia who volunteered to participate in his study. The study area is located in the Rift Valley at 7°3’ N latitude.
**91**	Gilbert-Diamond D	2010	Colombia	In February 2006, we recruited 3202 children aged 5–12 y from public schools in Bogota, Colombia as part of an observational longitudinal study in nutrition and health.
**92**	Gill TK	2014	Australia	This paper reports data collected as part of Stage 3 of the North West Adelaide Health Study (NWAHS). The NWAHS is a representative longitudinal study of 4056 randomly selected adults aged 18 years and over at the time of recruitment from the north-west region of Adelaide, the capital of SA.

**93**	Ginter JK	2013	Canada	Recruitment of participants for this study took place in February and early March of 2012 at the Square One Older Adult Centre (SOOAC), located in Mississauga, Ontario.
**94**	Glatt DU	2022	UK	The D-VinCHI study (D Vitamin in Children) is an ongoing multidisciplinary study at Ulster University, investigating vitamin D status in school children and the impact of vitamin D supplementation on 25(OH)D, muscle strength and sensorimotor performance, cognitive function and bone and immune health.
**95**	Gökta O	2020	Turkey	After obtaining the necessary approvals, data on 25 OH vitamin D levels measured in patients admitted between 1 December 2017 and 30 November 2018 were analysed retrospectively from the records of adult patients aged ⩾ 18 years who were admitted to 24 family medicine centres located in different districts of Bursa.
**96**	Golbahar J	2014	Bahrain	The present study was conducted between October 2010 and October 2011 among volunteers attending the blood bank centre in Bahrain Defense Force (BDF) Hospital, the second largest hospital in Bahrain.
**97**	González G	2007	Chile	Healthy ambulatory women were invited, through notices in the community, to participate in this study.
**98**	González-Gross M	2012	Europe	The HELENA cross-sectional study was a multi-centre crosssectional study aiming to obtain reliable and comparable data from a random sample of 3000 European adolescents aged between 12·5 and 17·49 years on a broad battery of nutrition and health-related parameters.
**99**	González-Molero I	2011	Spain	The study was carried out in two population-based cohorts, one in the north and the other in the south of Spain, both studied at the same time and using similar methods. The study populations and the design of these two surveys have been described previously (Soriguer et al., 2002, 2008; Valde´s et al., 2007).
**100**	Gordon CM	2004	USA	We studied 307 primary care patients (aged 11-18 years) who presented consecutively for annual physical examinations between July 1, 2001, and June 30, 2003, to the adolescent outpatient clinic at Children's Hospital Boston and were undergoing a routine blood draw (eg, complete blood cell count).
**101**	Goswami R	2009	India	The study was carried out in four schools and a medical college of Delhi in November 2006 to March 2007, corresponding to winter months in India.
**102**	Granlund L	2016	Sweden	The Vitamin D Deficiency in Immigrants Survey (VIDI1) was designed as a population-based cross-sectional study and carried out in Umeå, a university town in Northern

				Sweden (latitude 63º N) with a population of 114 000 citizens at the time of the study. T
**103**	Greene-Finestone LS	2011	Canada	Data for this study come from the Canadian Multicentre Osteoporosis Study (CaMos), an ongoing longitudinal, population-based cohort study in nine Canadian city-based centers.
**104**	Griffin TP	2020	Ireland	The primary aim of this study was to compare Vitamin D status and serum 25-hydroxyvitamin D (25(OH)D) concentrations among adults sampled in the community, in outpatient clinics, as hospital inpatients, and in nursing homes in the West of Ireland (latitude 53.27°N).
**105**	Gromova O	2020	Kazakhstan	This cross-sectional study enrolled 1,347 healthy adults (out of them 819 were females), residing in 6 regions of Kazakhstan with a mean age of 44±14 years, who attended a single-consultation outpatient clinic for routine check-up.
**106**	Guo S	2014	Australia	Northeast Asian Australians with Chinese, Japanese and Korean ancestry (n = 100), aged 18–80 years were recruited through community organizations, distribution of information brochures, and snowball recruiting between May 2012 and April 2013.
**107**	Han B	2017	China	The study was conducted in three urban and three rural sites in Shanghai, one urban and six rural sites in Jiangxi Province, and three rural sites in Zhejiang Province.
**108**	Hansen L	2018	Denmark	The StatusD study was established between 2012 and 2014, and 3408 persons between 2 and 69 years from the general population (recruited in three regions in Denmark: Copenhagen Central area (63%), Odense (19%), and Kolding (18%)) were enrolled using a web-based enrollment system.
**109**	Harinarayan CV	2007	India	The study was conducted in 943 urban and 205 rural healthy subjects of Tirupati, southern Andhra Pradesh, India (lat 13.4°N, long 79.2°E).
**110**	Harkness LS	2005	USA	Healthy, postmenarcheal girls (age, 12–18 y), who were participating in a larger National Institutes of Health–sponsored clinical trial, were eligible for this cross-sectional analysis.
**111**	Hashemipour S	2004	Iran	1272 healthy men and women aged 20–69 years were selected based on randomized clustered sampling from 50 blocks in Tehran.
**112**	Hatun S	2005	Turkey	The study was conducted in Kocaeli, a relatively developed region of Turkey, located at 30°E and 40°N.
**113**	Hazell TJ	2015	Lebanon	A total of 488 children (age range: 1.8e6 yr) were studied between June 2010 and June 2011 from a random sample of licensed day cares in the Montreal area.

**114**	Hekimsoy Z	2010	Turkey	This cross-sectional study was conducted in the city of Manisa (38.36º N latitude), a non-coastal city in the Aegean region of Turkey with a population of 303,155.
**115**	Herrick KA	2019	USA	The aim of this study was to describe vitamin D status in the US population in 2011–2014 and trends from 2003 to 2014.
**116**	Hintzpeter B	2008	Germany	KiGGS is a representative survey of children and adolescents aged0–17 y, conducted fromMay 2003 toMay 2006.
**117**	Hirani V	2012	UK	Data were analysed from the 2005 HSE, an annual survey designed to measure health and health-related behaviours in a nationally representative sample of adults and children living in private households in England.
**118**	Hirani V	2013	Australia	The Concord Health and Ageing in Men Project (CHAMP) is an epidemiological study of a wide range of health issues in Australian men aged 70 years and over.
**119**	Hoge A	2015	Belgium	Data on vitamin D were available from the NESCaV, a population-based, cross-sectional survey of cardiovascular risk factors in Wallonia.
**120**	Ho-Pham LT	2011	Vietnam	The study was designed as a cross-sectional investigation in the setting of Ho Chi Minh City (formerly Saigon).
**121**	Horton-French K	2021	Australia	This cross-sectional study used data from the 2011–2013 AHS, which comprised the National Health Survey (NHS), the National Nutrition and Physical Activity Survey (NNPAS), and the National Health Measures Survey (NHMS).
**122**	Houghton LA	2019	Kenya	This cross‑sectional survey was conducted in March 2013 and based in Emali, a town situated in the county of Makueni that borders Kajiado County in southern Kenya.
**123**	Hovsepian S	2011	Iran	In this cross-sectional study in Isfahan, a sunny city located in the central part of Iran, 1,111 healthy individuals—243 men and 868 women—aged 41.4 (mean 14 and range 20-80) years, who attended a single-consultation outpatient clinic for routine check-up, were consecutively selected.
**124**	Hribar M	2020	Slovenia	The Nutrihealth study was conducted as an upgrade to the Slovenian national dietary survey SI.Menu 2017/2018, which was carried out following the EFSA Guidance on EU Menu Methodology
**125**	Hussain T	2021	Afghanistan	A cross-sectional study was performed between November 2020 and April 2021. Convenience sampling was used. Inclusion criteria were housewives of all ethnicities who gave consent and aged >18 years, visiting the outpatient department of a tertiary care hospital in Quetta, Pakistan.

**126**	Hutchings N	2022	Armenia	We conducted a cross-sectional cluster model study to measure levels of 25-OH D from a representative sample of women in Armenia.
**127**	Ikonen H	2021	Finland	The study population was derived from a prospective, general population-based birth cohort, the Northern Finland Birth Cohort 1966 (NFBC1966).
**128**	Isa H	2020	Bahrain	Medical records of children aged 1 to 16 years who attended a vitamin D screening campaign at Al Kindi Specialized Hospital, Bahrain between September and October 2016 were reviewed.
**129**	Islam MZ	2002	Bangladesh	The study was conducted in two regions of Bangladesh. Bangladesh lies within a tropical to subtropical monsoon climate zone. A total of 189 women were included from the two socio-economic groups aged 16 – 40 y.
**130**	Islam MZ	2008	Bangladesh	The present study was conducted in an export-oriented garment factory located in an urban area belonging to Standard Group Bangladesh, which maintains a high-quality working environment for its workers. A total of 200 subjects aged 18–36 years were randomly selected from the garment factory.
**131**	Jääskeläinen T	2017	Finland	This study was based on a nationally representative sample of Finnish adults aged $30 y from H2000, which was conducted from 2000 to 2001 (20), and its follow-up, H2011, which was conducted from 2011 to 2012.
**132**	Janssen HC	2013	The Netherlands	This is a cross-sectional, single-center study in 802 independently living men and women 40–80 years of age, in the central part of the Netherlands (52 degrees northern latitude). Male subjects (n = 400, 40–80 years) were extracted from the Hamlet study.
**133**	Jayashri R	2020	India	The study participants were recruited from the Chennai Urban Rural Epidemiology Study (CURES) in whom a ten-year follow up wasconducted between 2012 and 2013. The methodology of CURES has been published elsewhere
**134**	Jayatissa R	2019	Sri Lanka	This was a cross-sectional study among school children aged 10-18 years at national level. A representative sample of 2525 children were recruited from July to November 2017.
**135**	Jiang W	2020	China	we designed a cross-sectional study of different populations in different regions of China. The study was conducted simultaneously from January 2014 to December 2017.
**136**	Johnson MA	2008	USA	Study participants were part of the Georgia Centenarian Study, a population-based multidisciplinary study of centenarians conducted in 44 counties in northern Georgia (USA) from 2002 to 2005.

**137**	Jolliffe DA	2016	UK	We conducted a cross-sectional study in 222 older adults living in sheltered accommodation in London, UK, who were screened for participation in a clinical trial of vitamin D supplementation for the prevention of acute respiratory infection.
**138**	Jorde R	2010	Norway	The Tromsø Study, conducted by the University of Tromsø in cooperation with the National Health Screening Service, is a longitudinal, population-based, multipurpose study focusing on lifestyle-related diseases.
**139**	Joukar F	2020	Iran	We evaluated, the women of the PGCS ("PERSIAN Guilan Cohort Study") cohort, a prospective, population-based cohort study in Guilan, Iran which has been previously described in details
**140**	Junaid K	2015	Pakistan	Healthy adult females were screened for eligibility to participate in the study from January 2012 to July 2012 in Lahore, Pakistan.
**141**	Kaddam IM	2017	Saudi Arabia	A school-based cross-sectional study was conducted in 3 regions of Saudi Arabia between January 2013 and December 2014.
**142**	Kagotho E	2018	Kenya	This was a cross sectional study involving blood donors that was conducted at the AKUH,N Kenya blood donor unit from March to May 2015. AKUH,N is a private referral hospital that caters for the residents of Nairobi, Kenya and the greater East African region and receives 400–500 blood donors per month.
**143**	Kapil U	2018	India	A community based cross-sectional study was conducted in the year 2015-2016. Two districts (namely: Kangra and Kullu) of Himachal Pradesh state, India was selected for the present study.
**144**	Karagüzel G	2014	Turkey	This cross-sectional study was conducted in Trabzon province, which is geographically located at latitude 41º N in northeastern Turkey. A total of 21 public schools located in urban and suburban areas were included in the study.
**145**	Karin Z	2018	Croatia	The participants in this cross-sectional study were healthy preschool-age children from the southern part of Croatia (age range: 5–6 years; mean age: 6.0 ± 0.4 years; n = 260; 128 females).
**146**	Karonova T	2016	Russian Federation	We examined 1,664 residents from North-West region of Russia (St. Petersburg and Petrozavodsk, 59-61º North latitude) between 2009-2013 as a cross-sectional study that was conducted at two sites: Federal North-West Medical Research Centre and Pavlov First State Medical University, St.Petersburg.

**147**	Kaykhaei MA	2011	Iran	This population-based cross-sectional study was performed on 993 individual from June 2008 to November 2008 in Zahedan, southeast Iran.
**148**	Khan AH	2012	Pakistan	A cross-sectional study was conducted in randomly selected communities downtown (Saddar) and suburbs (Gulshan and Malir Town) in Karachi, Pakistan.
**149**	Kim SY	2020	Republic of Korea	Our study included 168,561 Korean adults aged 18 years and older, who had visited and undertaken comprehensive health examinations at Kangbuk Samsung Hospital Health Screening Center, between 1 January 2012 and 31 December 2017. South Korea guarantees all employees free annual or biennial health examinations as per the Industrial Safety and Health Law.
**150**	Kim YS	2020	Republic of Korea	This cross-sectional descriptive study involved 2314 adolescents aged 12-18years. Participant data were extracted from the Korean National Health and Nutrition Examination Survey conducted between January 2010 and December 2014.
**151**	Klenk J	2013	Germany	Between March 2009 and April 2010 the 25(OH)D serum level was assessed in 1,418 community-dwelling individuals living in Germany aged ⩾65 years (56.7% men) with no subscribed vitamin D supplementation.
**152**	Kouda K	2013	Japan	The source population comprised 521 fifth-grade children who attended either of the two public schools in Hamamatsu, Japan.
**153**	Koyama S	2021	Japan	A total of 492 adolescents (247 boys and 245 girls), who were in the frst grade of junior high school (12- to 13-yearolds) and had general health examinations of lifestyle disease, were enrolled in this study. They were recruited from seven junior high schools located in Otawara city, Japan, which lies at 36.52°north (N) latitude and 140° east longitude.
**154**	Kremer R	2009	USA	This was a cross-sectional study. Anthropometric measures, serum 25OHD radioimmunoassay values, and computed tomography and dual-energy x-ray absorptiometry values of BF and bone structure in 90 postpubertal females, aged 16-22 yr, residing in California were measured.
**155**	Kull M Jr	2009	Estonia	The study was conducted in Väike-Maarja municipality in Estonia in 2006.
**156**	Kunz C	2018	Germany	For this 6-year study (January 2009-December 2014) carried out in Mülheim an der Ruhr, Germany, healthy children and adolescents (n = 1929, age range 1-17 years, median age 11.0 years, 46.9% female) consulting a pediatric group practice were recruited.

**157**	Laird E	2018	Ireland	Participants were members of the TILDA Study, a nationally representative cohort of community-dwelling adults aged ≥ 50 years in Ireland
**158**	Langlois K	2010	Canada	The data are from the 2007 to 2009 Canadian Health Measures Survey.
**159**	Lappe JM	2006	USA	The subjects were 1,179 community-dwelling white women randomly selected from the population of healthy postmenopausal women over 55 years of age in a nine-county farming area of eastern Nebraska, U.S.A., centered at latitude 41° N.
**160**	Lardner E	2011	Ireland	A group of 197 apparently healthy, free living white women aged between 40 and 85 years with a mean age of 61 years were assessed in the study. Local general practitioners selected healthy women attending their practice between July 2006 and May 2007.
**161**	Lategan R	2016	South Africa	For this cross-sectional study, baseline data from the Assuring Health for All in the Free State (AHA-FS) study in an urban setting were used.
**162**	Le Goaziou MF	2011	France	This cross-sectional study was carried out by 13 GPs working in the Rhone Alps area at 45 ° N latitude.
**163**	Lee J	2021	Republic of Korea	This study began with data from 189,154 individuals who underwent a comprehensive health examination at Kangbuk Samsung Hospital Health Screening Centers in 2015.
**164**	Leung RY	2017	Hong kong	The Hong Kong Osteoporosis Study is a prospective follow-up study on musculoskeletal and mineral metabolism related conditions.
**165**	Li H	2020	China	The CCACH study was a large, nationwide and multicenter observational study conducted between 2013 and 2015, which was designed to examine body composition, cardiovascular health, and nutritional status among Chinese children and adolescents.
**166**	Li L	2020	China	The multi-center cross-sectional study was conducted across four seasons from May 21, 2018, to April 21, 2019, in 6 regions around Sichuan province, including Chengdu (CD), Nanchong (NC), Luzhou (LZ), Maerkang (MK), Guangyuan (GY), and Panzhihua (PH).
**167**	Li S	2014	China	From May 2008 to July 2008, a total of 578 urban postmenopausal Chinese women were recruited at random from community centers scattered around the city of Changsha (latitude 28°N, central south China) to ensure that the average health status of the participants will reflect a randomly selected adult population
**168**	Lima-Costa MF	2020	Brazil	Data came from the baseline survey of the Brazilian Longitudinal Study of Aging (ELSI), conducted in 2015–16.

**169**	Lin LY	2021	UK	The UK Biobank was compiled from 2006 to 2010 by recruiting participants throughout the UK.
**170**	Liu X	2018	USA	Serum 25-hydroxyvitamin D (25(OH)D) measurements were collected from 26 010 adults aged ≥18 years from the National Health and Nutrition Examination Survey (NHANES) 2001-2010.
**171**	Liu X	2020	Macao	A representative population sample from Macao was investigated in 2014.
**172**	Lopes JB	2009	Brazil	Cross-sectional study conducted for 2 years in the city of São Paulo, Brazil including community-dwelling elderly women.
**173**	Lucas JA	2005	New Zealand	We performed a cross-sectional study of 1,606 healthy, postmenopausal women recruited over a 33-month period.
**174**	Madsen KH	2014	Denmark	The present cross-sectional study used baseline data obtained from the VitmaD study conducted in Denmark.
**175**	Maguire JL	2011	Canada	A cross-sectional study was performed on healthy two-year-old children attending a well-child visit in Toronto, Ontario (latitude 43.4°N).
**176**	Marzban M	2021	Iran	The study was based in the Mitchells Plain/Klipfontein sub-district of Cape Town.
**177**	Majumdar V	2011	India	We investigated 441 randomly selected subjects 18–75 years of age.
**178**	Maldonado G	2017	Ecuador	Retrospective study of Ecuadorian subjects from the city of Guayaquil, Ecuador, who had an initial study of serum 25 (OH)-D, as the indicator of vitamin D status, from 2015 to 2016.
**179**	Mallah EM	2011	Jordan	This study was performed in November 2010 to determine the prevalence of vitamin D deficiency in apparently healthy Jordanian volunteers.
**180**	Man PW	2016	The Netherlands	We carried out an observational study among men and women, aged 18 years and older with a Chinese background and residing in the Netherlands in March 2014.
**181**	Manios Y	2017	Greece	The HGS was a large-scale cross-sectional epidemiological study initiated in May 2007 and completed in June 2009.
**182**	Mansbach JM	2009	USA	Serum 25(OH)D levels were obtained for participants aged 6 to 11 years from 2001–2006 and for those aged 1 to 5 years from 2003 to 2006.
**183**	Masoud MS	2020	Saudi Arabia	Arab adolescents aged 10-17 years randomly selected from the Vitamin D School Project Database (170 Saudi students; 100 girls, seventy boys). Vitamin D School Project Database, King Saud University (2014-2016).
**184**	Matheï C	2013	Belgium	Because of increasing reports of vitamin D deficiency among refugee and immigrant populations, between June 2007 and September 2009, blood testing for 25-

				hydroxyvitamin D level was included in routine health screening for refugees resettled in Massachusetts.
**185**	Mechenro J	2018	India	The BFC80+ is a prospective, observational, populationbased cohort study of Caucasian subjects aged 80 years and older in three well-circumscribed areas in Belgium.
**186**	Mechenro J	2018	India	Between June 2015 and July 2016, 424 healthy adults residing in Kattankulathur block in Tamil Nadu, India, provided venous blood samples and answered questions by personal interview. 25-hydroxy vitamin D was estimated by ELISA.
**187**	Mehboobali N	2015	Pakistan	A transversal descriptive study was conducted between January and March 2002. A total of 389 subjects aged 20–60 years were selected from an urban area near Tunis (Ariana) and interviewed in their homes.
**188**	Metwally ASM	2021	Saudi Arabia	The cross-sectional study was conducted in a low-income, unplanned settlement in Karachi, and comprised apparently healthy adults who were recruited randomly with informed consent.
**189**	Meyer HE	2004	Norway	A total of 1864 apparently healthy Saudi adolescent boys (N = 549) and girls (N = 1315) aged 13–17 (mean age 14.7 ± 1.7 years) from different public, private schools within Riyadh, SA were invited to participate in this cross-sectional study from November 2019 until March 2020 before the national lockdown was imposed.
**190**	Meyer HE	2008	Sri Lanka	We studied the prevalence of poor vitamin D status and the association with bone density in men and women born in Norway (quoted as Norwegians, n = 869) and Pakistan (quoted as Pakistanis, n = 177) in the population-based Oslo Health Study, 2000-2001.
**191**	Miljkovic I	2011	Trinidad and Tobago	A total of 196 participants aged 30-60 years in a cross-sectional population-based study in Kandy, Sri Lanka (latitude 7 degrees north) and 242 Sri Lankans aged 31-60 years participating in a cross-sectional population-based study in Oslo, Norway (latitude 60 degrees north) were included in the analysis.
**192**	Misra P	2017	India	Between 2000 and 2003, 3170 previously unscreened men were recruited for a population-based prostate cancer screening study on the Caribbean Island of Tobago, Trinidad and Tobago.
**193**	Mitchell DM	2012	USA	The present study is an analytical cross-sectional study conducted among females aged 20-60 years in rural Ballabgarh.

**194**	Mogire RM	2021	Kenya, Uganda, Burkina Faso, Gambia, South Africa	Healthy volunteers, aged 18 to 50 years, were recruited for endocrine research studies through mass mailings and advertisements in local newspapers and Internet sites. Visits occurred between January 2006 and May 2008.
**195**	Moreno-Reyes R	2009	Belgium	This study included young children living in Kenya (n = 1361), Uganda (n = 1301), Burkina Faso (n = 329), The Gambia (n = 629) and South Africa (n = 889).
**196**	Moussavi M	2005	Iran	We conducted a cross-sectional survey in a stratified random sample of 401 subjects aged between 40 and 60 years living in Brussels, and drawn from 4 different ethnic backgrounds: autochthonous Belgian, Moroccan, Turkish and Congolese.
**197**	Moy FM	2011	Malaysia	In a cross-sectional study in Isfahan, a city located in the central part of Iran (32° 39 N lat.), 318 high school students (153 males and 165 females), aged 14–18 years, were selected by multistage sampling.
**198**	Moy FM	2017	Malaysia	This was an analytical cross-sectional study. The study population was a group of Malay employees from a health screening program of a public university in Kuala Lumpur.
**199**	Muhairi SJ	2013	United Arab Emirates	This was a cross-sectional study conducted between March and October 2013. The study was conducted in public secondary schools in Kuala Lumpur, the federal capital of Malaysia.
**200**	Mutua AM	2020	Uganda	This was a cross-sectional study in urban schools. Healthy adolescents (N=315) from a sample of 8 schools were randomly selected from the 142 schools in Al Ain, Abu Dhabi Emirate.
**201**	Nadeem S	2018	Pakistan	The Entebbe Mother and Baby Study (EMaBS) prospective birth cohort was initially designed as a double-blind randomized controlled trial of the effects of anthelmintic treatment during pregnancy and early childhood on immunological and disease outcomes in childhood (ISRCTN32849447).
**202**	Naeem Z	2011	Saudi Arabia	This cross sectional study included responses gathered on questionnaire from medical students of Karachi Medical &Dental College from 4th of August 2017 till 30th April 2018.
**203**	Nakamura K	2008	Japan	One hundred and eighty healthy males and females subjects above the age of 18 years were randomly selected from five primary health care centers of Qassim region.
**204**	Nakhaee S	2019	Iran	All 1310 women who lived in Yokogoshi area (Niigata City, Japan) aged between 55 and 74 years on March 31, 2006, were invited to participate in the Yokogoshi Study, a cross-

				sectional, epidemiologic, community-based investigation of bone health for postmenopausal women. The study was conducted in November 2005.
**205**	Nälsén C	2020	Sweden	In a descriptive-analytic study, 400 subjects over 40 years of age were enrolled. The population of Birjand's comprehensive urban health centers was considered to determine the share of each center.
**206**	Nälsén C	2020	Sweden	The results are based on two cross-sectional studies in Sweden. One study included adults, and one study included school children. Both studies were performed by the Swedish National Food Agency (NFA).
**207**	NHANES (2015–2016)	2022	USA	The study was conducted in the province of Izabal on the Caribbean coast of Guatemala, in two different locations.
**208**	NHANES(2017–2018)	2022	USA	National Health and Nutrition Examination Survey is a nationally representative nutrition survey of general populations in the United States using a stratified, multi-stage random sampling design. The latest data for vitamin D status are available as of NHANES 2017–2018,
**209**	Niafar M	2009	Iran	The Cork BASELINE (Babies after SCOPE: Evaluating the Longitudinal Impact using Neurological and Nutritional Endpoints) Birth Cohort Study was initiated in 2008 and is an ongoing prospective mother–infant birth cohort study based in Cork, Ireland.
**210**	Nichols EK	2015	Jordan	The subjects of this study were enrolled from the participants of a community-based survey which was carried out from January 2008 to April 2008 in Tabriz, the capital city of East Azerbaijan, a province in north-west Iran.
**211**	Nielsen NO	2014	Denmark	Survey staff collected data during a national household-based micronutrient survey of women (15–49 years) and children (12–59 months) in Jordan during March–April 2010.
**212**	Nikooyeh B	2017	Iran	A total of 2877 randomly selected Inuit (⩾ 18 years) from the Inuit Health in Transition study were included.
**213**	Nimitphong H	2013	Thailand	A total of 1406 healthy subjects aged 19-60 years were randomly selected from six regions of Iran across latitudinal gradient from 29° N to 37.5° N.
**214**	Oberg J	2014	Norway	This cross-sectional study was a part of health survey of employees of the Electricity Generating Authority of Thailand. There were 1,990 healthy subjects (72.8% male) in this study.
**215**	Oliveri B	2004	Argentina	The data presented come from The Tromsø Study: Fit Futures, during the school year 2010/2011 (not including the

				summer months), where 1,038 (92% of those invited) participated.
**216**	Orces CH	2015	Ecuador	In all, 386 ambulatory subjects over 65 y of age, from seven cities in Argentina (between latitude 261S and 551S) were asked to participate in the study, to be performed between the end of winter and the beginning of spring (15 August–15 October).
**217**	Orces CH	2015	Ecuador	The present study was based on data from participants in the National Survey of Health, Wellbeing, and Aging (Encuesta de Salud, Bienestar y Envejecimiento) conducted in 2009.
**218**	Öztürk ZA	2017	Turkey	We conducted a cross-sectional evaluation of 1606 older men in the general community who were enrolled in the Osteoporotic Fractures in Men Study. A randomly selected subcohort of a large population of men from six U.S. communities participated in the study.
**219**	Pan T	2018	India	In this study, the population was comprised of 363 females and 798 males between the ages of 18 and 90 years.
**220**	Patel JV	2013	UK	A community-based cross-sectional study was conducted from April-December 2017 among 194 women aged 40 years and above residing in the village of Singur, West Bengal.
**221**	Paul TV	2008	India	South Asian (SA) and Black African-Caribbean (AC) were recruited as part of a community heart failure study from 20 primary care practices, Birmingham, UK.
**222**	Penrose K	2012	USA	We conducted a community-based cross-sectional study in a semiurban region. The study cohort consisted of 150 ambulatory postmenopausal women (> or = 50 years old).
**223**	Pérez-Llamas F	2008	Spain	The cross-sectional study included 86 subjects, 65-94 y of age (29 men and 57 women), who lived in three nursing homes in Murcia, a Spanish Mediterranean area.
**224**	Perna L	2012	Germany	We standardized immunoassay-based measurements of 25(OH)D with LC-MS/MS in a population-based sample of 5386 women aged 50-74 recruited in 2000-2002 in Germany.
**225**	Peters BS	2009	Brazil	One hundred and thirty-six adolescents, 64 boys and 72 girls, aged 16-20 years old, living in a rural town in the state of São Paulo, Brazil, participated in this study.
**226**	Petrenya N	2020	Norway	Cross-sectional data from the second survey of the Population-based Study on Health and Living Conditions in Regions with Sami and Norwegian Populations (the SAMINOR 2 Clinical Survey, 2012-2014).
**227**	Qorbani M	2021	Iran	This nationwide cross-sectional study was performed as part of a surveillance program in Iran. Participants were 2596 students, aged 7 to 18 years, living in 30 provinces.

**228**	Rabenberg M	2015	Germany	the 'German Health Interview and Examination Survey for Adults' (DEGS1),a national health survey among adults in Germany conducted by the Robert Koch Institute between 2008 and 2011, included 6,995 persons with available serum 25(OH)D levels.
**229**	Rabufetti A	2019	Switzerland	All apparently healthy subjects attending for the medical evaluation before the compulsory military service in Southern Switzerland during 2014-2016 were eligible.
**230**	Rafraf M	2014	Iran	In the current cross-sectional study, a sample of 216 girls (14–17 years old) was selected from high schools in Boukan city during winter (in February) 2012 by a multistage stratified random sampling technique.
**231**	Rahmadhani R	2017	Malaysia	Thirteen-year-old participants were recruited via multistage sampling from 23 randomly selected government-funded secondary schools across the city of Kuala Lumpur, Malaysia from January 2012 to July 2012.
**232**	Rahman A	2020	Kuwait	We evaluated the prevalence of VDD in adolescents (11-16 years-old; n = 410) by both methods in a cross-sectional study. Subjects were selected from public middle schools from all the 6 Governorates of Kuwait using stratified multistage cluster random sampling.
**233**	Ramakrishnan S	2011	India	Four colleges in Chandigarh (latitude 30° 42′ north and longitude 76° 54′ east; altitude varying from 304.8 to 365.76 m above sea level) were chosen by simple random sampling as sites for study subjects.
**234**	Raposo L	2017	Portugal	PORMETS (PORtuguese METabolic Syndrome) is a national cross-sectional study that includes a sample of adults registered in primary health care centers of the Portuguese mainland.
**235**	Riverin B	2013	Canada	Data for these analyses are from the Nituuchischaayihtitaau Aschii: A Multi-Community Environment and Health Longitudinal Study in James Bay Cree communities.
**236**	Riverin B	2014	Canada	Data for these analyses were from the Nituuchischaayihtitaau Aschii Study. The study is a comprehensive health survey of the James Bay Cree communities resulting from a research partnership among individual Eeyou First Nations, the Cree Board of Health and Social Services of James Bay, the Centre de recherche du CHU de Québec, Laval University (Laval, Quebec), McMaster University (Hamilton, Ontario) and McGill University (Montreal, Quebec).
**237**	Robinson PJ	2013	Australia	For the PROSPECT study, 267 GPs, recruited from 17 628 invited GPs across Australia, contributed patients to the study, primarily located in each state capital city.

**238**	Rodríguez-Rodríguez E	2011	Spain	This was a cross-sectional observational study, carried out in schools in Madrid (Spain), during 2007-2008. The study enrolled 102 schoolchildren (aged 9-13 years).
**239**	Saeed BQ	2021	United Arab Emirates	A cross-sectional and prospective design was used. Our sample consisted of 287 students aged 18-24 years from the University of Sharjah-UAE.
**240**	Saki F	2017	Iran	Cross-sectional study. Iranian children (n 477) aged 9-18 years. Fars Province, Iran, 2011.
**241**	Sakyi SA	2021	Ghana	In a cross-sectional study, a total of five hundred (500) healthy blood donors from three geographical areas in Ghana were enrolled.
**242**	Saliba W	2012	Israel	Clalit Health Services (CHS) is a non-for-profit health maintenance organization (HMO) covering more than half of the Israeli population (3,871,215 members). The study population includes all CHS members for whom a 25(OH) D test result in 2009 was available and who were not taking vitamin D supplements in 2008–2009 previous to the 25 (OH)D test. If more than one test was available, we selected the last test in 2009.
**243**	Samefors M	2014	Sweden	The Study of Health and Drugs in the Elderly (SHADES) is a prospective cohort study among elderly people (>65 years) in 11 nursing homes in Sweden.
**244**	Santos A	2017	Portugal	A cross-sectional observational study was conducted in Portugal in a sample of 1500 Portuguese subjects ⩾65 years old.
**245**	Santos BR	2012	Brazil	This cross-sectional study was carried out between April 2008 and January 2011 and included 234 apparently healthy girls aged 7 to 18 years who had parental consent to participate in the study. Two hundred and thirteen girls recruited at four public schools from the four main regions of the city of Curitiba (North, South, East, and West), in the state of Paraná, Brazil (latitude −25°), and 21 girls enrolled at a vaccination facility or University adolescent clinic in the city of Porto Alegre (latitude −30°), state of Rio Grande do Sul, were included in the study.
**246**	Santos BR	2019	Brazil	This is a cross-sectional study of biorepository samples collected from 443 women aged 20 to 72 years, with no evidence of clinical disease, living in southern Brazil (30th parallel South).
**247**	Sarafin K	2015	Canada	The Canadian Health Measures Survey (CHMS) is an ongoing cross-sectional national survey that includes a measure of 25-hydroxyvitamin D [25(OH)D] by immunoassay. For cycles 1 and 2, the collection period

				occurred approximately every 2 y, with a new sample of ~5600 individuals.
**248**	Saraiva GL	2005	Brazil	This cross-sectional study comprised 250 free-living elderly people (>65 years) (173 women and 77 men) with a mean age of 79.1 (5.9) years belonging to the fifth biannual cycle (2000–2001) of a prospective cohort study of the Geriatrics Discipline of the Federal University of Sa˜o Paulo.
**249**	Scalco R	2008	Brazil	From 320 elders living in the two nonprofit old-age houses at Porto Alegre, 102 subjects agreed to participate.
**250**	Schramm S	2017	Germany	We used baseline data of 4149 participants (45-75 years, 50% women) of the population-based Heinz Nixdorf Recall study.
**251**	Science M	2017	Canada	We conducted a cross-sectional study of children aged 3 to 15 living in Canadian Hutterite communities. Serum 25(OH)D levels were measured between October 2008 and April 2009 using a chemiluminescence assay.
**252**	Seo JA	2013	Republic of Korea	All study subjects were derived from the Ansan cohort of the Korean Genome Epidemiology Study (KoGES), an ongoing population-based cohort study that began in 2001.
**253**	Shady MM	2015	Egypt	Two hundred boys and girls aged from 9 to 11 years were recruited from two primary public schools situated in Giza governorate in Egypt.
**254**	Sharawat IK	2019	India	This was a cross-sectional study, conducted in 100 apparently healthy school-going children of both sexes, aged between 60 and 120 months, studying in Government Primary School from north western state of India, in the month of May to July 2011.
**255**	Shchubelka K	2020	Ukraine	This retrospective study included 1823 randomly selected subjects among those, whose serum concentration of 25(OH) D was recorded in 2019 at the medical laboratory center "Astra Dia" (www.astra-dia.ua) in Transcarpathian region, Ukraine.
**256**	Sheikh A	2012	Pakistan	The study was conducted as a cross-sectional study, set in urban metropolis of Karachi - the largest city of Pakistan during the period of January 2011.
**257**	Sherchand O	2018	Nepal	This cross section study was conducted in the department of Biochemistry at B.P Koirala Institute of Health Sciences, Dharan from February to June 2017. The study population comprised of patients between the ages of 18 years and above who were sent from various clinical departments for serum vitamin D measurement.
**258**	Sherief LM	2021	Egypt	A cross-sectional study was conducted on 572 school children (270 males and 302 females) aged 14 to 18 years,

				who were randomly selected from high schools in one governorate in Egypt.
**259**	Shetty S	2014	India	We conducted a survey of the total number of houses in an urban region of south India. Men above 50 years of age in that locality were recruited by cluster random sampling after obtaining a written informed consent.
**260**	Shivane VK	2011	India	This cross-sectional study was conducted at a tertiary care centre in western India. A total of 1137 young (age: 25-35 years), healthy volunteers of both sexes were included in the study.
**261**	Sioen I	2012	Belgium	Participating children were drawn from the Belgian control region cohort of the EU 6th Framework Programme IDEFICS Study, residents from the city of Aalter in the northern Dutch-speaking part of Belgium.
**262**	Skull SA	2003	Australia	Vitamin D levels were performed on 116/126 (92%) participants, who were born in: (i) Somalia (53%), (ii) Eritrea (25%), (iii) Ethiopia (17%) and (iv) Sudan (5%).
**263**	Smith G	2016	Cambodia	In 2014, The Cambodian Demographic Health Survey (CDHS) conducted a nationallyrepresentative survey of women and men between the ages of 15 and 49 years of age, in 16,356 households.
**264**	Smith N	2021	UK	The cross-sectional study compared serum vitamin D levels among 149 women aged 35-59, comprising British-Bangladeshi migrants (n = 50), white British neighbors (n = 54) and Bangladeshi sedentees (n = 45).
**265**	Sochorová L	2018	Czech Republic	The study subjects were 419 healthy children aged 5 and 9 years. Czech children.
**266**	Sokolovic S	2017	Bosnia and Herzegovina	This study was performed as a cross-sectional epidemiologic analysis. During the one-year period, from 1 April 2013 to 31 March 2014, blood was collected from patients of different clinics at the Sarajevo University Clinical Center.
**267**	Solis-Urra P	2019	Chile	The 2016–2017 Chilean National Health Survey was a representative household survey with a stratified multistage probability sample of 6233 non-institutionalized participants over 14 years old from the 15 regions in Chile, both urban and rural.
**268**	Song HR	2014	Republic of Korea	The Dong-gu Study enrolled 9,260 subjects (3,711 men and 5,549 women) aged 50 yr and older between April and July from 2007 to 2010 in the Dong-gu district of Gwangju Metropolitan City in Korea (35°N).
**269**	Souberbielle JC	2016	France	We studied healthy volunteers who participated in the VARIETE study, a population-based cross-sectional study designed to recruit a reference population in order to

				harmonize normal adult serum IGF-I values (ClinicalTrials.-gov identifier: NCT01831648).
**270**	Srimani S	2017	India	This cross-sectional study was conducted among 222 randomly selected rural PMW in Singur Block, West Bengal, India.
**271**	Sulimani RA	2016	Saudi Arabia	In this prospective study, a total of 2000 school girls (aged 12-18 years) from intermediate and secondary schools in Riyadh, KSA (latitude, 24.6º N) were recruited during the period September 2011 to June 2014.
**272**	Suryanarayana P	2018	India	A community-based cross-sectional study adopting random sampling procedure was carried out among urban elderly in Hyderabad metropolitan city in south India during the year 2014-15. The latitude of the city is “17.3850° N, 78.4867° E”.
**273**	Tangoh DA	2018	Cameroon	The study was a community-based prospective longitudinal study. It was carried out during the dry and rainy seasons between the months of July and December 2015 in the South West Region of Cameroon involving 372 participants aged 35 years and above.
**274**	Ten Haaf DSM	2019	The Netherlands	Participants of the 4 Days Marches of 2015 or 2016, an annual 4 day walking event in the Netherlands that takes place in July, were recruited via newsletters and internet advertisements. Participants had to be 65 year or older and Caucasian.
**275**	Thuesen B	2012	Denmark	The persons included in this study were participants in the Inter99 study.
**276**	Tolppanen AM	2012	UK	The Avon Longitudinal Study of Parents and Children is a population-based contemporary birth cohort (children born in 1991-1992) from southwest England.
**277**	Tønnesen R	2016	Denmark	The cross-sectional population study was carried out among Copenhagen citizens from June 2012 to May 2014.
**278**	Tran B	2013	Australia	the pilot D-Health trial was a populationbased, randomized, placebo-controlled double-blind chemoprevention trial of vitamin D3 (cholecalciferol). We recruited 644 people aged between 60 and 84 years who were residents of one of the four eastern states of Australia.
**279**	Tseng M	2009	USA	Participants in this cross-sectional analysis were 194 African American men in the Philadelphia region who were enrolled in a risk assessment program for prostate cancer from 10/96-10/07.
**280**	Unger MD	2010	Brazil	603 (118M and 485F) healthy Brazilian volunteers aged 18-90 years from a university hospital were selected after the winter of 2006. From the initial sample, 209 volunteers

				(31M and 178F) accepted to participate in a second health check after the subsequent summer.
**281**	Uush T	2013	Mongolia	A total of 400 households were randomly selected from each of 4 economic regions and Ulaanbaatar city.
**282**	Vallejo MS	2020	Chile	A cross-sectional study was conducted with 1329 healthy subjects (668 women and 661 men) aged 18-89 years in Santiago, Chile.
**283**	Vallianou N	2012	Greece	To assess vitamin D status and health correlates in a sample of apparently healthy Caucasian participants residing in an urban area, Athens. Men and women 35+ years from a selected population (n = 490) were studied.
**284**	Vasudevan B	2021	India	A cross-sectional study was conducted among 184 perimenopausal women in two districts of Kerala from July 2018 to February 2019.
**285**	Vierucci F	2014	Italy	We enrolled 427 Italian adolescents (10.0-21.0 years) recruited from the Pediatric Clinic of the University of Pisa, living in the Northwestern area of Tuscany, Central Italy (latitude 43°43′N) during a period of 24 months (October 2010-September 2012).
**286**	von Hurst PR	2010	New Zealand	Women of South Asian origin (n 235) aged 20 years and older were tested for serum 25(OH)D, and 228 were included in these analyses.
**287**	Voortman T	2015	The Netherlands	The aims of this study were to describe vitamin D status in the Generation R study, a large multiethnic cohort of 6-y-old children in The Netherlands.
**288**	Vupputuri MR	2006	India	Study subjects included 105 healthy persons. The BMD was measured in all subjects at the All India Institute of Medical Sciences under the Indian Council of Medical Research task force study to establish normative data of BMD in Asian Indians.
**289**	Wakayo T	2015	Ethiopia	A school based cross-sectional study was conducted from May 20–June 22, 2013, in Adama City (n = 89) and in Rural Adama woreda (n = 85) located in Central Ethiopia (latitude: 8°33'- 8°36'N).
**290**	Ward M	2011	UK	Participants are from the 1958 British birth cohort, which consists of a large sample of individuals born in England, Scotland or Wales in 1 week in March and enrolled in the Perinatal Mortality Survey.
**291**	White Z	2019	South Africa	Data were collected by means of a cross-sectional study on 84 conveniently sampled black preadolescent South African children (44 girls, 40 boys; mean ± SD age 8.5 ± 1.4 years) from September to November (spring season) 2016 in Pretoria, South Africa, at a latitude of 25 °S.

**292**	Wyskida M	2018	Poland	The PolSenior project, conducted between 2007 and 2011, was a large national, multicentre, interdisciplinary study that assessed the social and health situation of old people in Poland.
**293**	Yan X	2019	China	The study subjects were adult residents living in Jinzhong city (latitude 37.68° north). During November and December 2017, we randomly selected 500 people, stratified by age, from 10 neighborhoods.
**294**	Yang K	2020	China	A total of 1928 volunteers (aged 18–87 years) were selected to participate in the National Survey Of Diabetes Prevalence in Gansu province, including 958 males and 970 females. The survey and blood collection took place during December 2015 and May 2016.
**295**	Yousef S	2021	Canada	We used a cross-sectional design with data from the national Canadian Health Measures Survey (Cycles 3 and 4) (11,579 participants aged 3–79 years).
**296**	Yu L	2020	China	This study included children who underwent a routine physical examination between March 1 and June 30 during 2017–2020.
**297**	Yu S	2015	China	The study population was a part of the International Federation of Clinical Chemistry (IFCC) Global Multicenter study of reference intervals in China. According to the IFCC Committee on Reference Intervals and Decision Limits protocol,21 2627 healthy volunteers were recruited from 5 representative geographical cities in China—Dalian (northeast), Beijing (north), Hangzhou (east), Guangzhou (south), and Urumqi (northwest)—between May 1 and September 30, 2013.
**298**	Zargar AH	2007	India	92 healthy natives (64 men and 28 non-pregnant/non-lactating women, aged 18-40 years), residing in Kashmir for at least last 5 years
**299**	Zgaga L	2011	UK	The study population comprised 2235 healthy adults identified through the Community Health Index and invited by their general practitioner to take part as controls in a national case control study of colorectal cancer in Scotland.
**300**	Zhang FF	2016	Kuwait	In a cross-sectional study of 960 adults enrolled in the first National Nutrition Survey of the State of Kuwait (NNSSK).
**301**	Zhao Y	2021	China	Data was based on the baseline of West China Health and Aging Trends study (WCHAT). All of the participants were older than 60 years old in the present study.
**302**	Zhen D	2015	China	This cross-sectional study involved 2942 men and 7158 women aged 40-75years who were randomly selected from 3 communities in the Lanzhou urban district and examined medically.

**303**	Zhou SJ	2015	Australia	A cross-sectional sample of 1329 children aged 2 to <5 years were enrolled from throughout New Zealand in late-winter to spring 2012.
**304**	Zhu W	2018	China	Data for this study were originally collected as part of the Study of Urban Residents Eatingout Behavior (SUREB) (13), a cross-sectional community-based survey that has evaluated dining-out behaviors and nutritional status of urban residents in Shanghai, China.
**305**	Zhu Z	2012	China	Data collection took place between March 2008 and February 2011, throughout the year, during winter (December through February), spring (March through May), summer (June through August), and autumn (September through November). The children aged 1 to 16 years who came to the child health care department of our hospital, the Children's Hospital affiliated to Zhejiang university school of medicine, for health examination were taken blood for 25(OH)D measurement.
**306**	Middelkoop K	2022	South Africa	The study used secondary data of 11,822 adolescent children from the Community National Nutrition Survey (CNNS), undertaken in 2016-18.
**307**	Lin L	2022	China	The rural inhabitants of more than 25 years old from three mountainous, plain, and seashore areas of Bushehr province were selected through a stratified multi-cluster random sampling method. A total of 1806 (means ±SD, 46± 14 years old) rural subjects (35 % males and 65 % females) participated in this study.
**308**	Chakrabarty S	2022	India	A total of 1,700 healthy adults, aged 18-86 years (617 men and 1,073 women), were enrolled in our cross-sectional descriptive study. adult residents in Hainan, the tropical island province of China.

Appendix 3: The assessment of the quality of included studies

We used the risk of bias tool for prevalence studies developed by Hoy D et, al., which comprises 10 items plus a summary assessment. Items 1 to 4 assess the external validity of the study (domains are selection and nonresponse bias), and items 5 to 10 assess the internal validity (items 5 to 9 assess the domain of measurement bias, and item 10 assesses bias related to the analysis). The summary assessment evaluates the overall risk of study bias and is based on the rater`s subjective judgment given responses to the preceding 10 items. Response options for individual items were either low or high risk of bias; Response options for the summary assessment were low, moderate, or high risk of bias.

10 items plus a summary assessment are shown below:

1. Was the study`s target population a close representation of the national population in relation to relevant variables, e.g. age, sex, occupation?

2. Was the sampling frame a true or close representation of the target population?

3. Was some form of random selection used to select the sample, OR, was a census undertaken?

4. Was the likelihood of non-response bias minimal?

5. Were data collected directly from the subjects (as opposed to a proxy)?

6. Was an acceptable case definition used in the study?

7. Was the study instrument that measured the parameter of interest (e.g. prevalence of low back pain) shown to have reliability and validity (if necessary)?

8. Was the same mode of data collection used for all subjects?

9. Was the length of the shortest prevalence period for the parameter of interest appropriate?

10. Were the numerator(s) and denominator(s) for the parameter of interest appropriate?

11. Summary item on the overall risk of study bias

Reference

Hoy D, Brooks P, Woolf A, Blyth F, March L, Bain C, Baker P, Smith E, Buchbinder R. Assessing risk of bias in prevalence studies: modification of an existing tool and evidence of interrater agreement. J Clin Epidemiol. 2012 Sep;65(9):934-9.

Supplementary Table 8 The summaries of quality assessment of included studies

Author	Year	Country	1	2	3	4	5	6	7	8	9	10	11
Abdulrahman MA	2022	Iraq											
Abiaka C	2013	Oman											
Abu-Samak MS	2019	Jordan											
Al Shaikh A	2020	Saudi											
Al Shaikh AM	2015	Saudi											
Al Shaikh AM	2016	Saudi											
Al Shaikh AM	2016	Saudi											
Al Shaikh AM	2021	Saudi											
Al Zarooni AAR	2019	United Arab Emirates											
Aleteng Q	2017	China											
AlFaris NA	2019	Saudi											
Alkerwi A	2015	Luxembourg											
Al-Kindi MK	2011	Oman											
Alloubani A	2019	Saudi											

AlQuaiz AM	2018	Saudi											
Al-Saleh Y	2015	Saudi											
Al-Taiar A	2018	Kuwait											
Alyahya K	2014	Kuwait											
Alyahya KO	2020	Kuwait											
Andersen R	2005	Europe											
Andersen R	2009	Denmark											
Andersen R	2013	Denmark											
Andersen S	2013	Denmark											
Arabi A	2021	Lebanon											
Ardawi MS	2011	Saudi											
Ardawi MS	2012	Saudi											
Arnljots R	2017	Sweden											
Arya V	2004	India											
Asakura K	2020	Japan											
Aspell N	2019	UK											
Aucoin M	2013	Canada											
Bachhel R	2015	India											
Bater J	2021	Mongolia											
Batieha A	2011	Jordan											
Beer RJ	2020	Colombia											
Bener A	2009	Qatar											
Benjeddou K	2019	Morocco											
Bettencourt A	2018	Portugal											

Bezrati I	2016	Tunisia											
Bhatt SP	2014	India											
Bhattoa HP	2013	Hungary											
Bi X	2016	Singapore											
Bjarnadottir A	2014	Iceland											
Black LJ	2021	Australia											
Bodin J	2019	Ethiopia											
Bolland MJ	2006	New Zealand											
Borissova AM	2013	Bulgaria											
Brinkmann K	2015	Chile											
Byun EJ	2017	Krean											
Cabral MA	2013	Brazil											
Cairncross CT	2017	New Zealand											
Capuano R	2021	Italy											
Carrillo-Vega MF	2017	Mexico											
Cashman KD	2013	Ireland											
Ceccarelli M	2020	Italy											
Chailurkit LO	2011	Thailand											
Chao YS	2013	Canada											
Chao YS	2014	Canada											
Chakrabarty S	2022	India											
Chen J	2017	China											
Chin KY	2014	Malaysia											
Chirita-Emandi A	2015	Romania											

Chlebna-Sokół D	2019	Poland											
Choi HR	2017	Krean											
Chung IH	2014	Krean											
Chung JY	2013	Krean											
Cinar N	2014	Turkey											
Contreras-Manzano A	2021	Mexico											
Cougnard-Grégoire A	2015	France											
Courraud J	2020	Denmark											
Crowe FL	2019	UK											
Dalgård C	2010	Denmark											
Daly RM	2012	Australia											
de Oliveira CL	2020	Brazil											
Djennane M	2014	Algeria											
Drali O	2021	Algeria											
Duarte C	2020	Portugal											
El Hayek J	2010	Canada											
El Hayek J	2013	Canada											
El-Khateeb M	2019	Jordan											
Eloi M	2016	Brazil											
Fang F	2018	China											
Fayet-Moore F	2019	Australia											
Feketea GM	2021	Greece											
Feng X	2016	China											
Fernández Bustillo JM	2018	Spain											

Flores ME	2021	Mexico
Ganmaa D	2014	Mongolia
Gariballa S	2022	United Arab Emirates
Gebreegziabher T	2013	Ethiopia
Gilbert-Diamond D	2010	Colombia
Gill TK	2014	Australia
Ginter JK	2013	Canada
Glatt DU	2022	UK
Gökta O	2020	Turkey
Golbahar J	2014	Bahrain
González G	2007	Chile
González-Gross M	2012	Europe
González-Molero I	2011	Spain
Gordon CM	2004	USA
Goswami R	2009	India
Granlund L	2016	Sweden
Greene-Finestone LS	2011	Canada
Griffin TP	2020	Ireland
Gromova O	2020	Kazakhstan
Guo S	2014	Australia
H. E. Meyer	2008	Sri Lanka
Han B	2017	China
Hansen L	2018	Denmark
Harinarayan CV	2007	India

Harkness LS	2005	USA											
Hashemipour S	2004	Iran											
Hatun S	2005	Turkey											
Hazell TJ	2015	Lebanon											
Hekimsoy Z	2010	Turkey											
Herrick KA	2019	USA											
Hintzpeter B	2008	Germany											
Hirani V	2012	UK											
Hirani V	2013	Australia											
Hoge A	2015	Belgium											
Ho-Pham LT	2011	Vietnam											
Horton-French K	2021	Australia											
Houghton LA	2019	Kenya											
Hovsepian S	2011	Iran											
Hribar M	2020	Slovenia											
Hussain T	2021	Afghanistan											
Hutchings N	2022	Armenia											
Ikonen H	2021	Finland											
Isa H	2020	Bahrain											
Islam MZ	2002	Bangladesh											
Islam MZ	2008	Bangladesh											

Jääskeläinen T	2017	Finland											
Janssen HC	2013	The Netherlands											
Jayashri R	2020	India											
Jiang W	2020	China											
Johnson MA	2008	USA											
Jolliffe DA	2016	UK											
Jorde R	2010	Norway											
Joukar F	2020	Iran											
Junaid K	2015	Pakistan											
Kaddam IM	2017	Saudi											
Kagotho E	2018	Kenya											
Kapil U	2018	India											
Karagüzel G	2014	Turkey											
Karin Z	2018	Croatia											
Karonova T	2016	Russian Federation											
Kaykhaei MA	2011	Iran											
Khan AH	2012	Pakistan											
Kim SY	2020	Krean											
Kim YS	2018	Krean											
Klenk J	2013	Germany											

Kouda K	2013	Japan											
Koyama S	2021	Japan											
Kremer R	2009	USA											
Kull M Jr	2009	Estonia											
Kunz C	2019	Germany											
Laird E	2018	Ireland											
Langlois K	2010	Canada											
Lappe JM	2006	USA											
Lardner E	2011	Ireland											
Lategan R	2016	South Africa											
Le Goaziou MF	2011	France											
Lee J	2021	Krean											
Leung RY	2017	Hong kong											
Lin L	2022	China											
Li H	2020	China											
Li L	2020	China											
Li S	2014	China											
Lima-Costa MF	2020	Brazil											
Lin LY	2021	UK											

Liu X	2018	USA											
Liu X	2020	China Macao											
Lopes JB	2009	Brazil											
Lucas JA	2005	New Zealand											
Middelkoop K	2022	South Africa											
Madsen KH	2014	Denmark											
Maguire JL	2011	Canada											
Majumdar V	2011	India											
Maldonado G	2017	Ecuador											
Mallah EM	2011	Jordan											
Man PW	2019	The Netherlands											
Manios Y	2017	Greece											
Mansbach JM	2009	USA											
Masoud MS	2020	Saudi											
Penrose K	2012	USA											
Matheï C	2013	Belgium											
Mechenro J	2018	India											
Meddeb N	2005	Tunisia											
Mehboobali N	2015	Pakistan											

Metwally ASM	2021	Saudi											
Meyer HE	2004	Norway											
Miljkovic I	2011	Republic of Trinidad and Tobago											
Misra P	2017	India											
Mitchell DM	2012	USA											
Mogire RM	2021	Kenya, Uganda, Burkina Faso, The Gambia, South Africa											
Moreno-Reyes R	2009	Belgium											
Moussavi M	2005	Iran											
Moy FM	2011	Malaysia											
Moy FM	2017	Malaysia											
Muhairi SJ	2013	United Arab Emirates											
Mutua AM	2020	Uganda											
Nadeem S	2018	Pakistan											
Naeem Z	2011	Saudi											
Nakamura K	2008	Japan											
Nakhaee S	2019	Iran											
Nälsén C	2020	Sweden											
Naqvi A	2017	Guatemala											

NHANES (2015–2016 and 2017–2018)	2022	USA											
Ní Chaoimh C	2018	Ireland											
Niafar M	2009	Iran											
Nichols EK	2015	Jordan											
Nielsen NO	2014	Denmark											
Nikooyeh B	2017	Iran											
Nikooyeh B	2017	Iran											
Nimitphong H	2013	Thailand											
Oberg J	2014	Norway											
Oliveri B	2004	Argentina											
Orces CH	2015	Ecuador											
Orwoll E	2009	USA											
Öztürk ZA	2017	Turkey											
Pan T	2018	India											
Patel JV	2013	UK											
Paul TV	2008	India											
Pérez-Llamas F	2008	Spain											
Perna L	2012	Germany											

Peters BS	2009	Brazil											
Petrenya N	2020	Norway											
Qorbani M	2021	Iran											
R. Jayatissa	2019	Sri Lanka											
Rabenberg M	2015	Germany											
Rabufetti A	2019	Switzerland											
Rafraf M	2014	Iran											
Rahmadhani R	2017	Malaysia											
Rahman A	2020	Kuwait											
Ramakrishnan S	2011	India											
Raposo L	2017	Portugal											
Riverin B	2013	Canada											
Riverin B	2014	Canada											
Robinson PJ	2013	Australia											
Rodríguez-Rodríguez E	2011	Spain											
S. Al Hayek	2018	Lebanon											
Saeed BQ	2021	United Arab Emirates											
Saki F	2017	Iran											
Sakyi SA	2021	Ghana											

Saliba W	2012	Israel											
Samefors M	2014	Sweden											
Santos A	2017	Portugal											
Santos BR	2012	Brazil											
Santos BR	2019	Brazil											
Sarafin K	2015	Canada											
Saraiva GL	2005	Brazil											
Scalco R	2008	Brazil											
Schramm S	2017	Germany											
Science M	2017	Canada											
Seo JA	2013	Krean											
Shady MM	2015	Egypt											
Sharawat IK	2017	India											
Shchubelka K	2020	Ukraine											
Sheikh A	2012	Pakistan											
Sherchand O	2018	Nepal											
Sherief LM	2021	Egypt											
Shetty S	2014	India											
Shivane VK	2011	India											
Sioen I	2012	Belgium											
Skull SA	2003	Australia											

Smith G	2016	China
Smith N	2021	UK
Sochorová L	2018	Czech Republic
Sokolovic S	2017	Bosnia and Herzegovina
Solis-Urra P	2019	Chile
Song HR	2014	Krean
Souberbielle JC	2016	France
Srimani S	2017	India
Sulimani RA	2016	Saudi
Suryanarayana P	2018	India
Tangoh DA	2018	Cameroon
Ten Haaf DSM	2019	The Netherlands
Thuesen B	2012	Denmark
Tolppanen AM	2012	UK
Tønnesen R	2016	Denmark
Tran B	2013	Australia
Tseng M	2009	USA
Unger MD	2010	Brazil
Uush T	2013	Mongolia
Vallejo MS	2020	Chile
Vallianou N	2012	Greece
Vasudevan B	2021	India
Vierucci F	2014	Italy
von Hurst PR	2010	New Zealand

Voortman T	2015	The Netherlands											
Vupputuri MR	2006	India											
Wakayo T	2015	Ethiopia											
Ward M	2011	UK											
White Z	2019	South Africa											
Wyskida M	2018	Poland											
Yan X	2019	China											
Yang K	2020	China											
Yousef S	2021	Canada											
Yu L	2020	China											
Yu S	2015	China											
Zargar AH	2007	India											
Zgaga L	2011	UK											
Zhang FF	2016	Kuwait											
Zhao Y	2021	China											
Zhen D	2015	China											
Zhou SJ	2015	Australia											
Zhu W	2018	China											
Zhu Z	2012	China											

high risk of bias   moderate risk of bias   low risk of bias

Appendix 4: The global prevalence of vitamin D deficiency

Study	Events	Total	Proportion	95%−CI	Weight (common)	Weight (random)
Beer RJ	129	6813	0.02	[0.02; 0.02]	0.1%	0.4%
Beer RJ	194	6470	0.03	[0.03; 0.03]	0.1%	0.4%
Beer RJ	265	7170	0.04	[0.03; 0.04]	0.1%	0.4%
Beer RJ	395	16454	0.02	[0.02; 0.03]	0.2%	0.4%
Chin KY	2	150	0.01	[0.00; 0.05]	0.0%	0.4%
Gebreegziabher T	29	196	0.15	[0.10; 0.21]	0.0%	0.4%
Jayatissa R	333	2525	0.13	[0.12; 0.15]	0.0%	0.4%
Lin L	1	860	0.00	[0.00; 0.01]	0.0%	0.4%
Lin L	1	353	0.00	[0.00; 0.02]	0.0%	0.4%
Lin L	1	477	0.00	[0.00; 0.01]	0.0%	0.4%
Mechenro J	4	48	0.08	[0.02; 0.20]	0.0%	0.4%
Mechenro J	41	101	0.41	[0.31; 0.51]	0.0%	0.4%
Mechenro J	46	275	0.17	[0.13; 0.22]	0.0%	0.4%
Meyer HE	7	196	0.04	[0.01; 0.07]	0.0%	0.4%
Paul TV	15	150	0.10	[0.06; 0.16]	0.0%	0.4%
Shetty S	18	252	0.07	[0.04; 0.11]	0.0%	0.4%
Shivane VK	220	1137	0.19	[0.17; 0.22]	0.0%	0.4%
Smith G	7	128	0.05	[0.02; 0.11]	0.0%	0.4%
Smith G	13	495	0.03	[0.01; 0.04]	0.0%	0.4%
Abdulrahman MA	13	63	0.21	[0.11; 0.33]	0.0%	0.4%
Abdulrahman MA	16	50	0.32	[0.20; 0.47]	0.0%	0.4%
Abdulrahman MA	50	278	0.18	[0.14; 0.23]	0.0%	0.4%
Abiaka C	80	206	0.39	[0.32; 0.46]	0.0%	0.4%
Al Hayek S	33	344	0.10	[0.07; 0.13]	0.0%	0.4%
Al Shaikh A	1687	3613	0.47	[0.45; 0.48]	0.0%	0.4%
Al Shaikh AM	61	204	0.30	[0.24; 0.37]	0.0%	0.4%
Al Shaikh AM	899	1906	0.47	[0.45; 0.49]	0.0%	0.4%
Al−Daghri NM	150	561	0.27	[0.23; 0.31]	0.0%	0.4%
Al−Daghri NM	235	830	0.28	[0.25; 0.32]	0.0%	0.4%
Al−Daghri NM	276	808	0.34	[0.31; 0.38]	0.0%	0.4%
Al−Daghri NM	718	2225	0.32	[0.30; 0.34]	0.0%	0.4%
Al−Daghri NM	1082	4247	0.25	[0.24; 0.27]	0.1%	0.4%
Al−Daghri NM	1083	3111	0.35	[0.33; 0.37]	0.0%	0.4%
Al−Kindi MK	51	100	0.51	[0.41; 0.61]	0.0%	0.4%
AlQuaiz AM	729	2832	0.26	[0.24; 0.27]	0.0%	0.4%
Al−Saleh Y	718	2226	0.32	[0.30; 0.34]	0.0%	0.4%
Al−Taiar A	559	1416	0.39	[0.37; 0.42]	0.0%	0.4%
Alyahya K	169	232	0.73	[0.67; 0.78]	0.0%	0.4%
Alyahya KO	88	104	0.85	[0.76; 0.91]	0.0%	0.4%
Arabi A	182	466	0.39	[0.35; 0.44]	0.0%	0.4%
Ardawi MS	264	843	0.31	[0.28; 0.35]	0.0%	0.4%
Ardawi MS	534	1172	0.46	[0.43; 0.48]	0.0%	0.4%
Arya V	44	92	0.48	[0.37; 0.58]	0.0%	0.4%
Bachhel R	60	150	0.40	[0.32; 0.48]	0.0%	0.4%
Bezrati I	92	225	0.41	[0.34; 0.48]	0.0%	0.4%
Bhatt SP	9	137	0.07	[0.03; 0.12]	0.0%	0.4%
Capuano R	137	1200	0.11	[0.10; 0.13]	0.0%	0.4%
Chakrabarty S	1166	5024	0.23	[0.22; 0.24]	0.1%	0.4%
Chakrabarty S	1608	6798	0.24	[0.23; 0.25]	0.1%	0.4%
Chen J	731	6014	0.12	[0.11; 0.13]	0.1%	0.4%
Choi HR	110	651	0.17	[0.14; 0.20]	0.0%	0.4%
Djennane M	35	435	0.08	[0.06; 0.11]	0.0%	0.4%
Drali O	93	621	0.15	[0.12; 0.18]	0.0%	0.4%
Duarte C	706	3092	0.23	[0.21; 0.24]	0.0%	0.4%
Fang F	139	1814	0.08	[0.06; 0.09]	0.0%	0.4%
Golbahar J	247	500	0.49	[0.45; 0.54]	0.0%	0.4%
Goswami R	559	642	0.87	[0.84; 0.90]	0.0%	0.4%
Hashemipour S	812	1210	0.67	[0.64; 0.70]	0.0%	0.4%
Hatun S	19	89	0.21	[0.13; 0.31]	0.0%	0.4%
Herrick KA	7	1438	0.00	[0.00; 0.01]	0.0%	0.4%
Herrick KA	29	2060	0.01	[0.01; 0.02]	0.0%	0.4%
Herrick KA	94	3267	0.03	[0.02; 0.04]	0.0%	0.4%
Herrick KA	113	2355	0.05	[0.04; 0.06]	0.0%	0.4%
Herrick KA	199	3496	0.06	[0.05; 0.07]	0.0%	0.4%
Herrick KA	271	3564	0.08	[0.07; 0.09]	0.0%	0.4%
Hovsepian S	231	1111	0.21	[0.18; 0.23]	0.0%	0.4%
Hutchings N	157	1206	0.13	[0.11; 0.15]	0.0%	0.4%
Islam MZ	27	189	0.14	[0.10; 0.20]	0.0%	0.4%
Islam MZ	31	244	0.13	[0.09; 0.18]	0.0%	0.4%
Jiang W	1197	14302	0.08	[0.08; 0.09]	0.2%	0.4%
Joukar F	1189	5096	0.23	[0.22; 0.25]	0.1%	0.4%
Kaddam IM	912	2104	0.43	[0.41; 0.45]	0.0%	0.4%
Kaddam IM	1979	4035	0.49	[0.47; 0.51]	0.1%	0.4%
Kim SY	10652	157211	0.07	[0.07; 0.07]	2.1%	0.4%
Li H	1020	4989	0.20	[0.19; 0.22]	0.1%	0.4%
Li H	1020	5707	0.18	[0.17; 0.19]	0.1%	0.4%
Mallah EM	8	300	0.03	[0.01; 0.05]	0.0%	0.4%
Manios Y	122	2353	0.05	[0.04; 0.06]	0.0%	0.4%
Mansbach JM	17	1799	0.01	[0.01; 0.02]	0.0%	0.4%
Mansbach JM	27	2759	0.01	[0.01; 0.01]	0.0%	0.4%
Masoud MS	117	170	0.69	[0.61; 0.76]	0.0%	0.4%
Meddeb N	66	389	0.17	[0.13; 0.21]	0.0%	0.4%
Nakamura K	22	600	0.04	[0.02; 0.05]	0.0%	0.4%
Nakhaee S	115	400	0.29	[0.24; 0.33]	0.0%	0.4%
Niafar M	115	300	0.38	[0.33; 0.44]	0.0%	0.4%
Nichols EK	180	915	0.20	[0.17; 0.22]	0.0%	0.4%
Nikooyeh B	374	667	0.56	[0.52; 0.60]	0.0%	0.4%
Nikooyeh B	974	1406	0.69	[0.67; 0.72]	0.0%	0.4%
Öztürk ZA	150	1161	0.13	[0.11; 0.15]	0.0%	0.4%
Pérez−Llamas F	28	86	0.33	[0.23; 0.44]	0.0%	0.4%
Qorbani M	276	2596	0.11	[0.09; 0.12]	0.0%	0.4%
Rodríguez−Rodríguez E	8	102	0.08	[0.03; 0.15]	0.0%	0.4%
Saeed BQ	48	287	0.17	[0.13; 0.22]	0.0%	0.4%
Saki F	63	477	0.13	[0.10; 0.17]	0.0%	0.4%
Saliba W	891	6518	0.14	[0.13; 0.15]	0.1%	0.4%
Saliba W	27676	192240	0.14	[0.14; 0.15]	2.5%	0.4%
Santos A	594	1500	0.40	[0.37; 0.42]	0.0%	0.4%
Sharawat IK	29	100	0.29	[0.20; 0.39]	0.0%	0.4%
Sulimani RA	645	1618	0.40	[0.37; 0.42]	0.0%	0.4%
Tseng M	66	194	0.34	[0.27; 0.41]	0.0%	0.4%
Yan X	81	302	0.27	[0.22; 0.32]	0.0%	0.4%
Yu S	128	2173	0.06	[0.05; 0.07]	0.0%	0.4%
Zhang FF	348	960	0.36	[0.33; 0.39]	0.0%	0.4%
Zhu Z	6	183	0.03	[0.01; 0.07]	0.0%	0.4%
Zhu Z	26	2269	0.01	[0.01; 0.02]	0.0%	0.4%
Zhu Z	29	1440	0.02	[0.01; 0.03]	0.0%	0.4%
Alkerwi A	207	1335	0.16	[0.14; 0.18]	0.0%	0.4%
Andersen R	8	52	0.15	[0.07; 0.28]	0.0%	0.4%
Andersen R	13	54	0.24	[0.13; 0.38]	0.0%	0.4%
Andersen R	17	221	0.08	[0.05; 0.12]	0.0%	0.4%
Andersen R	30	37	0.81	[0.65; 0.92]	0.0%	0.3%
Andersen R	37	199	0.19	[0.13; 0.25]	0.0%	0.4%
Andersen R	158	219	0.72	[0.66; 0.78]	0.0%	0.4%
Arnljots R	224	545	0.41	[0.37; 0.45]	0.0%	0.4%
Asakura K	40	107	0.37	[0.28; 0.47]	0.0%	0.4%
Aspell N	1423	6004	0.24	[0.23; 0.25]	0.1%	0.4%
Aucoin M	78	756	0.10	[0.08; 0.13]	0.0%	0.4%
Aucoin M	96	461	0.21	[0.17; 0.25]	0.0%	0.4%
Bater J	5692	9595	0.59	[0.58; 0.60]	0.1%	0.4%
Borissova AM	430	2016	0.21	[0.20; 0.23]	0.0%	0.4%
Cashman KD	76	1132	0.07	[0.05; 0.08]	0.0%	0.4%
Ceccarelli M	209	2140	0.10	[0.09; 0.11]	0.0%	0.4%
Chao YS	13	1493	0.01	[0.00; 0.01]	0.0%	0.4%
Chirita−Emandi A	265	6631	0.04	[0.04; 0.04]	0.1%	0.4%
Chlebna−Sokó.. D	145	720	0.20	[0.17; 0.23]	0.0%	0.4%
Cougnard−Grégoire A	190	697	0.27	[0.24; 0.31]	0.0%	0.4%
Crowe FL	223186	755174	0.30	[0.29; 0.30]	9.9%	0.4%
Crowe FL	299730	993895	0.30	[0.30; 0.30]	13.0%	0.4%
Crowe FL	300265	901352	0.33	[0.33; 0.33]	11.8%	0.4%
Crowe FL	443448	1180086	0.38	[0.37; 0.38]	15.5%	0.4%
Crowe FL	531681	1316390	0.40	[0.40; 0.40]	17.2%	0.4%
Crowe FL	536113	1269812	0.42	[0.42; 0.42]	16.6%	0.4%
El Hayek J	3	508	0.01	[0.00; 0.02]	0.0%	0.4%
Fernández Bustillo JM	9	153	0.06	[0.03; 0.11]	0.0%	0.4%
Ganmaa D	333	420	0.79	[0.75; 0.83]	0.0%	0.4%
Ginter JK	3	224	0.01	[0.00; 0.04]	0.0%	0.4%
Glatt DU	3	49	0.06	[0.01; 0.17]	0.0%	0.4%
Greene−Finestone LS	8	312	0.03	[0.01; 0.05]	0.0%	0.4%
Greene−Finestone LS	18	777	0.02	[0.01; 0.04]	0.0%	0.4%
Greene−Finestone LS	19	823	0.02	[0.01; 0.04]	0.0%	0.4%
Griffin TP	2047	15319	0.13	[0.13; 0.14]	0.2%	0.4%
Gromova O	371	1347	0.28	[0.25; 0.30]	0.0%	0.4%
Hansen L	79	527	0.15	[0.12; 0.18]	0.0%	0.4%
Hansen L	309	2565	0.12	[0.11; 0.13]	0.0%	0.4%
Hintzpeter B	1797	9001	0.20	[0.19; 0.21]	0.1%	0.4%
Hirani V	292	2070	0.14	[0.13; 0.16]	0.0%	0.4%
Hoge A	58	915	0.06	[0.05; 0.08]	0.0%	0.4%
Hribar M	31	125	0.25	[0.18; 0.33]	0.0%	0.4%
Hribar M	36	155	0.23	[0.17; 0.31]	0.0%	0.4%
Jääskeläinen T	17	4051	0.00	[0.00; 0.01]	0.1%	0.4%
Jolliffe DA	55	222	0.25	[0.19; 0.31]	0.0%	0.4%
Jorde R	157	2668	0.06	[0.05; 0.07]	0.0%	0.4%
Karagüzel G	75	746	0.10	[0.08; 0.12]	0.0%	0.4%
Karin Z	31	260	0.12	[0.08; 0.16]	0.0%	0.4%
Kunz C	63	327	0.19	[0.15; 0.24]	0.0%	0.4%
Kunz C	106	528	0.20	[0.17; 0.24]	0.0%	0.4%
Kunz C	111	456	0.24	[0.20; 0.29]	0.0%	0.4%
Kunz C	112	520	0.22	[0.18; 0.25]	0.0%	0.4%
Laird E	728	5356	0.14	[0.13; 0.15]	0.1%	0.4%
Langlois K	218	5306	0.04	[0.04; 0.05]	0.1%	0.4%
Lardner E	14	143	0.10	[0.05; 0.16]	0.0%	0.4%
Le Goaziou MF	105	196	0.54	[0.46; 0.61]	0.0%	0.4%
Lin LY	60687	448601	0.14	[0.13; 0.14]	5.9%	0.4%
Madsen KH	4	415	0.01	[0.00; 0.02]	0.0%	0.4%
Maguire JL	1	91	0.01	[0.00; 0.06]	0.0%	0.4%
Man PW	40	416	0.10	[0.07; 0.13]	0.0%	0.4%
Matheï C	129	367	0.35	[0.30; 0.40]	0.0%	0.4%
Mitchell DM	45	634	0.07	[0.05; 0.09]	0.0%	0.4%
Moreno−Reyes R	135	401	0.34	[0.29; 0.39]	0.0%	0.4%
Nälsén C	9	268	0.03	[0.02; 0.06]	0.0%	0.4%
Nälsén C	10	206	0.05	[0.02; 0.09]	0.0%	0.4%
NHANES (2015...2016)	37	1777	0.02	[0.01; 0.03]	0.0%	0.4%
NHANES(2015...2016)	23	1231	0.02	[0.01; 0.03]	0.0%	0.4%
NHANES(2015...2016)	26	2618	0.01	[0.01; 0.01]	0.0%	0.4%
NHANES(2015...2016)	85	2413	0.04	[0.03; 0.04]	0.0%	0.4%
NHANES(2017...2018)	12	1321	0.01	[0.00; 0.02]	0.0%	0.4%
NHANES(2017...2018)	37	2184	0.02	[0.01; 0.02]	0.0%	0.4%
NHANES(2017...2018)	43	1810	0.02	[0.02; 0.03]	0.0%	0.4%
NHANES(2017...2018)	80	2094	0.04	[0.03; 0.05]	0.0%	0.4%
Ní Chaoimh C	12	741	0.02	[0.01; 0.03]	0.0%	0.4%
Orwoll E	47	1606	0.03	[0.02; 0.04]	0.0%	0.4%
Patel JV	1283	1904	0.67	[0.65; 0.69]	0.0%	0.4%
Perna L	844	5386	0.16	[0.15; 0.17]	0.1%	0.4%
Rabenberg M	540	1816	0.30	[0.28; 0.32]	0.0%	0.4%
Rabenberg M	756	2456	0.31	[0.29; 0.33]	0.0%	0.4%
Rabenberg M	804	2723	0.30	[0.28; 0.31]	0.0%	0.4%
Rabufetti A	24	1045	0.02	[0.01; 0.03]	0.0%	0.4%
Raposo L	188	500	0.38	[0.33; 0.42]	0.0%	0.4%
Riverin B	3	52	0.06	[0.01; 0.16]	0.0%	0.4%
Riverin B	54	944	0.06	[0.04; 0.07]	0.0%	0.4%
Sarafin K	839	11336	0.07	[0.07; 0.08]	0.1%	0.4%
Schramm S	660	4149	0.16	[0.15; 0.17]	0.1%	0.4%
Science M	4	743	0.01	[0.00; 0.01]	0.0%	0.4%
Shchubelka K	211	1639	0.13	[0.11; 0.15]	0.0%	0.4%
Sioen I	18	357	0.05	[0.03; 0.08]	0.0%	0.4%
Smith N	19	148	0.13	[0.08; 0.19]	0.0%	0.4%
Sochorová L	101	419	0.24	[0.20; 0.28]	0.0%	0.4%
Sokolovic S	93	450	0.21	[0.17; 0.25]	0.0%	0.4%
Sokolovic S	185	645	0.29	[0.25; 0.32]	0.0%	0.4%
Sokolovic S	235	735	0.32	[0.29; 0.35]	0.0%	0.4%
Souberbielle JC	56	892	0.06	[0.05; 0.08]	0.0%	0.4%
Thuesen B	848	6146	0.14	[0.13; 0.15]	0.1%	0.4%
Tolppanen AM	124	7560	0.02	[0.01; 0.02]	0.1%	0.4%
Tønnesen R	135	700	0.19	[0.16; 0.22]	0.0%	0.4%
Uush T	222	524	0.42	[0.38; 0.47]	0.0%	0.4%
Uush T	457	876	0.52	[0.49; 0.56]	0.0%	0.4%
Voortman T	258	4167	0.06	[0.05; 0.07]	0.1%	0.4%
Wyskida M	355	3472	0.10	[0.09; 0.11]	0.0%	0.4%
Yousef S	5	267	0.02	[0.01; 0.04]	0.0%	0.4%
Yousef S	38	932	0.04	[0.03; 0.06]	0.0%	0.4%
Yousef S	63	1361	0.05	[0.04; 0.06]	0.0%	0.4%
Yousef S	90	858	0.10	[0.09; 0.13]	0.0%	0.4%
Yousef S	988	8161	0.12	[0.11; 0.13]	0.1%	0.4%
Zgaga L	772	2235	0.35	[0.33; 0.37]	0.0%	0.4%
Bjarnadottir A	5	158	0.03	[0.01; 0.07]	0.0%	0.4%
Courraud J	31	177	0.18	[0.12; 0.24]	0.0%	0.4%
Dalgård C	124	669	0.19	[0.16; 0.22]	0.0%	0.4%
El Hayek J	39	282	0.14	[0.10; 0.18]	0.0%	0.4%
Granlund L	26	216	0.12	[0.08; 0.17]	0.0%	0.4%
Ikonen H	92	3650	0.03	[0.02; 0.03]	0.0%	0.4%
Nielsen NO	525	2877	0.18	[0.17; 0.20]	0.0%	0.4%
Oberg J	147	890	0.17	[0.14; 0.19]	0.0%	0.4%
Petrenya N	31	4465	0.01	[0.00; 0.01]	0.1%	0.4%
Samefors M	48	333	0.14	[0.11; 0.19]	0.0%	0.4%
Lima−Costa MF	37	2264	0.02	[0.01; 0.02]	0.0%	0.4%
Maldonado G	3	269	0.01	[0.00; 0.03]	0.0%	0.4%
Black LJ	16	331	0.05	[0.03; 0.08]	0.0%	0.4%
Black LJ	23	490	0.05	[0.03; 0.07]	0.0%	0.4%
Black LJ	26	730	0.04	[0.02; 0.05]	0.0%	0.4%
Black LJ	30	648	0.05	[0.03; 0.07]	0.0%	0.4%
Black LJ	43	652	0.07	[0.05; 0.09]	0.0%	0.4%
Bolland MJ	2	378	0.01	[0.00; 0.02]	0.0%	0.4%
Brinkmann K	67	108	0.62	[0.52; 0.71]	0.0%	0.4%
Cairncross CT	86	1329	0.06	[0.05; 0.08]	0.0%	0.4%
Daly RM	46	1393	0.03	[0.02; 0.04]	0.0%	0.4%
Daly RM	66	810	0.08	[0.06; 0.10]	0.0%	0.4%
Daly RM	76	1564	0.05	[0.04; 0.06]	0.0%	0.4%
Daly RM	77	2011	0.04	[0.03; 0.05]	0.0%	0.4%
Daly RM	108	2556	0.04	[0.03; 0.05]	0.0%	0.4%
Daly RM	111	2884	0.04	[0.03; 0.05]	0.0%	0.4%
Gill TK	21	2413	0.01	[0.01; 0.01]	0.0%	0.4%
Hirani V	27	174	0.16	[0.10; 0.22]	0.0%	0.4%
Hirani V	30	304	0.10	[0.07; 0.14]	0.0%	0.4%
Hirani V	45	522	0.09	[0.06; 0.11]	0.0%	0.4%
Hirani V	57	659	0.09	[0.07; 0.11]	0.0%	0.4%
Lategan R	1	339	0.00	[0.00; 0.02]	0.0%	0.4%
Lucas JA	92	1606	0.06	[0.05; 0.07]	0.0%	0.4%
Oliveri B	35	339	0.10	[0.07; 0.14]	0.0%	0.4%
Robinson PJ	37	267	0.14	[0.10; 0.19]	0.0%	0.4%
Robinson PJ	74	640	0.12	[0.09; 0.14]	0.0%	0.4%
Saraiva GL	33	214	0.15	[0.11; 0.21]	0.0%	0.4%
Scalco R	53	98	0.54	[0.44; 0.64]	0.0%	0.4%
Skull SA	61	116	0.53	[0.43; 0.62]	0.0%	0.4%
Solis−Urra P	181	686	0.26	[0.23; 0.30]	0.0%	0.4%
Solis−Urra P	204	1245	0.16	[0.14; 0.19]	0.0%	0.4%
Tran B	9	134	0.07	[0.03; 0.12]	0.0%	0.4%
Tran B	11	148	0.07	[0.04; 0.13]	0.0%	0.4%
Tran B	11	148	0.07	[0.04; 0.13]	0.0%	0.4%
Tran B	15	131	0.11	[0.07; 0.18]	0.0%	0.4%
Tran B	20	92	0.22	[0.14; 0.32]	0.0%	0.4%
von Hurst PR	98	228	0.43	[0.36; 0.50]	0.0%	0.4%
Zhou SJ	9	221	0.04	[0.02; 0.08]	0.0%	0.4%
**Common effect model**		**7634261**	**0.32**	**[0.32; 0.32]**	**100.0%**	**−−**
**Random effects model**			**0.16**	**[0.14; 0.18]**	**−−**	**100.0%**

Heterogeneity: $I^2 = 100\%$, $\tau^2 = 0.0547$, $p = 0$

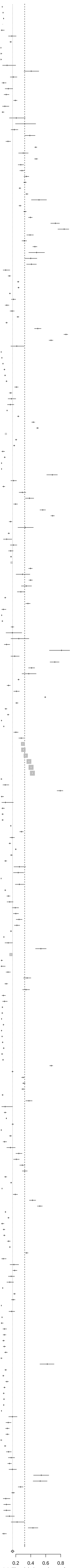

Supplementary figure 1.1 The global prevalence of serum 25(OH)D < 30 nmol/L

Study	Events	Total	Proportion	95%−CI	Weight (common)	Weight (random)
Abdulrahman MA2022Iraq	79	391	0.20	[0.16; 0.25]	0.0%	0.5%
Abiaka C2013Oman	80	206	0.39	[0.32; 0.46]	0.0%	0.5%
Al Shaikh A2020Saudi Arabia	1687	3613	0.47	[0.45; 0.48]	0.0%	0.6%
Al Shaikh AM2016Saudi Arabia	61	204	0.30	[0.24; 0.37]	0.0%	0.5%
Al Shaikh AM2016Saudi Arabia	899	1906	0.47	[0.45; 0.49]	0.0%	0.6%
Al−Daghri NM2015Saudi Arabia	953	3055	0.31	[0.30; 0.33]	0.0%	0.6%
Al−Daghri NM2016Saudi Arabia	426	1369	0.31	[0.29; 0.34]	0.0%	0.6%
Al−Daghri NM2021Saudi Arabia	2165	7358	0.29	[0.28; 0.30]	0.1%	0.6%
Alkerwi A2015Luxembourg	207	1335	0.16	[0.14; 0.18]	0.0%	0.6%
Al−Kindi MK2011Oman	51	100	0.51	[0.41; 0.61]	0.0%	0.5%
AlQuaiz AM2018Saudi Arabia	729	2832	0.26	[0.24; 0.27]	0.0%	0.6%
Al−Saleh Y2015Saudi Arabia	718	2226	0.32	[0.30; 0.34]	0.0%	0.6%
Al−Taiar A2018Kuwait	559	1416	0.39	[0.37; 0.42]	0.0%	0.6%
Alyahya K2014Kuwait	169	232	0.73	[0.67; 0.78]	0.0%	0.5%
Alyahya KO2020Kuwait	88	104	0.85	[0.76; 0.91]	0.0%	0.5%
Andersen R2005Denmark, Finland, Ireland, Poland.	54	420	0.13	[0.10; 0.16]	0.0%	0.5%
Andersen R2008Denmark	188	256	0.73	[0.68; 0.79]	0.0%	0.5%
Andersen R2013Denmark	21	106	0.20	[0.13; 0.29]	0.0%	0.5%
Arabi A2021Lebanon	182	466	0.39	[0.35; 0.44]	0.0%	0.5%
Ardawi MS2011Saudi Arabia	534	1172	0.46	[0.43; 0.48]	0.0%	0.6%
Ardawi MS2012Saudi Arabia	264	843	0.31	[0.28; 0.35]	0.0%	0.6%
Arnljots R2017Sweden	224	545	0.41	[0.37; 0.45]	0.0%	0.5%
Arya V2004India	44	92	0.48	[0.37; 0.58]	0.0%	0.5%
Asakura K2020Japan	40	107	0.37	[0.28; 0.47]	0.0%	0.5%
Aspell N2019UK	1423	6004	0.24	[0.23; 0.25]	0.1%	0.6%
Aucoin M2013Canada	174	1217	0.14	[0.12; 0.16]	0.0%	0.6%
Bachhel R2015India	60	150	0.40	[0.32; 0.48]	0.0%	0.5%
Bater J2021Mongolia	5692	9595	0.59	[0.58; 0.60]	0.1%	0.6%
Beer RJ2020Colombia	983	36907	0.03	[0.03; 0.03]	0.5%	0.6%
Bezrati I2016Tunisia	92	225	0.41	[0.34; 0.48]	0.0%	0.5%
Bhatt SP2014India	9	137	0.07	[0.03; 0.12]	0.0%	0.5%
Bjarnadottir A2014Iceland	5	158	0.03	[0.01; 0.07]	0.0%	0.5%
Black LJ2021Australia	138	2851	0.05	[0.04; 0.06]	0.0%	0.6%
Bolland MJ2006New Zealand	2	378	0.01	[0.00; 0.02]	0.0%	0.5%
Borissova AM2013Bulgaria	430	2016	0.21	[0.20; 0.23]	0.0%	0.6%
Brinkmann K2015Chile	67	108	0.62	[0.52; 0.71]	0.0%	0.5%
Cairncross CT2017New Zealand	86	1329	0.06	[0.05; 0.08]	0.0%	0.6%
Capuano R2021Italy	137	1200	0.11	[0.10; 0.13]	0.0%	0.6%
Cashman KD2013Ireland	76	1132	0.07	[0.05; 0.08]	0.0%	0.6%
Ceccarelli M2020Italy	209	2140	0.10	[0.09; 0.11]	0.0%	0.6%
Chao YS2014Canada	13	1493	0.01	[0.00; 0.01]	0.0%	0.6%
Chen J2017China	731	6014	0.12	[0.11; 0.13]	0.1%	0.6%
Chin KY2014Malaysia	2	150	0.01	[0.00; 0.05]	0.0%	0.5%
Chirita−Emandi A2015Romania	265	6631	0.04	[0.04; 0.04]	0.1%	0.6%
Chlebna−Sokó.. D2019Poland	145	720	0.20	[0.17; 0.23]	0.0%	0.5%
Choi HR2017Republic of Korea	110	651	0.17	[0.14; 0.20]	0.0%	0.5%
Cougnard−Grégoire A2015France	190	697	0.27	[0.24; 0.31]	0.0%	0.5%
Courraud J2020Denmark	31	177	0.18	[0.12; 0.24]	0.0%	0.5%
Crowe FL2019UK	2334423	6416709	0.36	[0.36; 0.36]	84.3%	0.6%
Dalgård C2010Denmark	124	669	0.19	[0.16; 0.22]	0.0%	0.5%
Daly RM2012Australia	484	11218	0.04	[0.04; 0.05]	0.1%	0.6%
Djennane M2014Algeria	35	435	0.08	[0.06; 0.11]	0.0%	0.5%
Drali O2021Algeria	93	621	0.15	[0.12; 0.18]	0.0%	0.5%
Duarte C2020Portugal	706	3092	0.23	[0.21; 0.24]	0.0%	0.6%
El Hayek J2010Canada	39	282	0.14	[0.10; 0.18]	0.0%	0.5%
El Hayek J2013Canada	3	508	0.01	[0.00; 0.02]	0.0%	0.5%
Fang F2018China	139	1814	0.08	[0.06; 0.09]	0.0%	0.6%
Fernández Bustillo JM2018Spain	9	153	0.06	[0.03; 0.11]	0.0%	0.5%
Ganmaa D2014Mongolia	333	420	0.79	[0.75; 0.83]	0.0%	0.5%
Gebreegziabher T2013Ethiopia	29	196	0.15	[0.10; 0.21]	0.0%	0.5%
Gill TK2014Australia	21	2413	0.01	[0.01; 0.01]	0.0%	0.6%
Ginter JK2013Canada	3	224	0.01	[0.00; 0.04]	0.0%	0.5%
Glatt DU2022UK	3	49	0.06	[0.01; 0.17]	0.0%	0.5%
Golbahar J2014Bahrain	247	500	0.49	[0.45; 0.54]	0.0%	0.5%
Goswami R2009India	559	642	0.87	[0.84; 0.90]	0.0%	0.5%
Granlund L2016Sweden	26	216	0.12	[0.08; 0.17]	0.0%	0.5%
Greene−Finestone LS2011Canada	45	1912	0.02	[0.02; 0.03]	0.0%	0.6%
Griffin TP2020Ireland	2047	15319	0.13	[0.13; 0.14]	0.2%	0.6%
Gromova O2020Kazakhstan	371	1347	0.28	[0.25; 0.30]	0.0%	0.6%
Hansen L2018Denmark	388	3092	0.13	[0.11; 0.14]	0.0%	0.6%
Hashemipour S2004Iran	812	1210	0.67	[0.64; 0.70]	0.0%	0.6%
Hatun S2005Turkey	19	89	0.21	[0.13; 0.31]	0.0%	0.5%
Herrick KA2019USA	713	16180	0.04	[0.04; 0.05]	0.2%	0.6%
Hintzpeter B2008Germany	1797	9001	0.20	[0.19; 0.21]	0.1%	0.6%
Hirani V2012UK	292	2070	0.14	[0.13; 0.16]	0.0%	0.6%
Hirani V2013Australia	159	1659	0.10	[0.08; 0.11]	0.0%	0.6%
Hoge A2015Belgium	58	915	0.06	[0.05; 0.08]	0.0%	0.5%
Hovsepian S2011Iran	231	1111	0.21	[0.18; 0.23]	0.0%	0.6%
Hribar M2020Slovenia	67	280	0.24	[0.19; 0.29]	0.0%	0.5%
Hutchings N2022Armenia	157	1206	0.13	[0.11; 0.15]	0.0%	0.6%
Sharawat KI2017India	29	100	0.29	[0.20; 0.39]	0.0%	0.5%
Ikonen H2021Finland	92	3650	0.03	[0.02; 0.03]	0.0%	0.6%
Islam MZ2002Bangladesh	27	189	0.14	[0.10; 0.20]	0.0%	0.5%
Islam MZ2008Bangladesh	31	244	0.13	[0.09; 0.18]	0.0%	0.5%
Jääskeläinen T2017Finland	17	4051	0.00	[0.00; 0.01]	0.1%	0.6%
Jayatissa R2019Sri Lanka	333	2525	0.13	[0.12; 0.15]	0.0%	0.6%
Jiang W2020China	1197	14302	0.08	[0.08; 0.09]	0.2%	0.6%
Jolliffe DA2016UK	55	222	0.25	[0.19; 0.31]	0.0%	0.5%
Jorde R2010Norway	157	2668	0.06	[0.05; 0.07]	0.0%	0.6%
Joukar F2020Iran	1189	5096	0.23	[0.22; 0.25]	0.1%	0.6%
Kaddam IM2017Saudi Arabia	2891	6139	0.47	[0.46; 0.48]	0.1%	0.6%
Karagüzel G2014Turkey	75	746	0.10	[0.08; 0.12]	0.0%	0.5%
Karin Z2018Croatia	31	260	0.12	[0.08; 0.16]	0.0%	0.5%
Kim SY2020Republic of Korea	10652	157211	0.07	[0.07; 0.07]	2.1%	0.6%
Kunz C2019Germany	392	1831	0.21	[0.20; 0.23]	0.0%	0.6%
Laird E2018Ireland	728	5356	0.14	[0.13; 0.15]	0.1%	0.6%
Langlois K2010Canada	218	5306	0.04	[0.04; 0.05]	0.1%	0.6%
Lardner E2011Ireland	14	143	0.10	[0.05; 0.16]	0.0%	0.5%
Lategan R2016South Africa	1	339	0.00	[0.00; 0.02]	0.0%	0.5%
Le Goaziou MF2011France	105	196	0.54	[0.46; 0.61]	0.0%	0.5%
Li H2020China	2040	10696	0.19	[0.18; 0.20]	0.1%	0.6%
Lima−Costa MF2020Brazil	37	2264	0.02	[0.01; 0.02]	0.0%	0.6%
Lin LY2021UK	60687	448601	0.14	[0.13; 0.14]	5.9%	0.6%
Lucas JA2005New Zealand	92	1606	0.06	[0.05; 0.07]	0.0%	0.6%
Madsen KH2014Denmark	4	415	0.01	[0.00; 0.02]	0.0%	0.5%
Maguire JL2011Canada	1	91	0.01	[0.00; 0.06]	0.0%	0.5%
Maldonado G2017Ecuador	3	269	0.01	[0.00; 0.03]	0.0%	0.5%
Mallah EM2011Jordan	8	300	0.03	[0.01; 0.05]	0.0%	0.5%
Man PW2019The Netherlands	40	416	0.10	[0.07; 0.13]	0.0%	0.5%
Manios Y2017Greece	122	2353	0.05	[0.04; 0.06]	0.0%	0.6%
Mansbach JM2009USA	44	4558	0.01	[0.01; 0.01]	0.1%	0.6%
Masoud MS2020Saudi Arabia	117	170	0.69	[0.61; 0.76]	0.0%	0.5%
Matheï C2013Belgium	129	367	0.35	[0.30; 0.40]	0.0%	0.5%
Mechenro J2018India	91	424	0.21	[0.18; 0.26]	0.0%	0.5%
Meddeb N2005Tunisia	66	389	0.17	[0.13; 0.21]	0.0%	0.5%
Meyer HE2008Sri Lanka	7	196	0.04	[0.01; 0.07]	0.0%	0.5%
Mitchell DM2012USA	45	634	0.07	[0.05; 0.09]	0.0%	0.5%
Moreno−Reyes R2009Belgium	135	401	0.34	[0.29; 0.39]	0.0%	0.5%
Nakamura K2008Japan	22	600	0.04	[0.02; 0.05]	0.0%	0.5%
Nakhaee S2019Iran	115	400	0.29	[0.24; 0.33]	0.0%	0.5%
Nälsén C2020Sweden	19	474	0.04	[0.02; 0.06]	0.0%	0.5%
NHANES(2015...2016)2022USA	171	8039	0.02	[0.02; 0.02]	0.1%	0.6%
Ní Chaoimh C2018Ireland	12	741	0.02	[0.01; 0.03]	0.0%	0.5%
Niafar M2009Iran	115	300	0.38	[0.33; 0.44]	0.0%	0.5%
Nichols EK2015Jordan	180	915	0.20	[0.17; 0.22]	0.0%	0.6%
Nielsen NO2014Denmark	525	2877	0.18	[0.17; 0.20]	0.0%	0.6%
Nikooyeh B2017Iran	1348	2073	0.65	[0.63; 0.67]	0.0%	0.6%
Oberg J2018Norway	147	890	0.17	[0.14; 0.19]	0.0%	0.5%
Oliveri B2004Argentina	35	339	0.10	[0.07; 0.14]	0.0%	0.5%
Orwoll E2009USA	47	1606	0.03	[0.02; 0.04]	0.0%	0.6%
Öztürk ZA2017Turkey	150	1161	0.13	[0.11; 0.15]	0.0%	0.6%
Patel JV2013UK	1283	1904	0.67	[0.65; 0.69]	0.0%	0.6%
Paul TV2008India	15	150	0.10	[0.06; 0.16]	0.0%	0.5%
Pérez−Llamas F2008Spain	28	86	0.33	[0.23; 0.44]	0.0%	0.5%
Perna L2012Germany	844	5386	0.16	[0.15; 0.17]	0.1%	0.6%
Petrenya N2020Norway	31	4465	0.01	[0.00; 0.01]	0.1%	0.6%
Qorbani M2021Iran	276	2596	0.11	[0.09; 0.12]	0.0%	0.6%
Rabenberg M2015Germany	2100	6995	0.30	[0.29; 0.31]	0.1%	0.6%
Rabufetti A2019Switzerland	24	1045	0.02	[0.01; 0.03]	0.0%	0.6%
Raposo L2017Portugal	188	500	0.38	[0.33; 0.42]	0.0%	0.5%
Riverin B2013Canada	54	944	0.06	[0.04; 0.07]	0.0%	0.5%
Riverin B2014Canada	3	52	0.06	[0.01; 0.16]	0.0%	0.5%
Robinson PJ2013Australia	111	907	0.12	[0.10; 0.15]	0.0%	0.5%
Rodríguez−Rodríguez E2011Spain	8	102	0.08	[0.03; 0.15]	0.0%	0.5%
Hayek SAI2018Lebanon	33	344	0.10	[0.07; 0.13]	0.0%	0.5%
Saeed BQ2021United Arab Emirates	48	287	0.17	[0.13; 0.22]	0.0%	0.5%
Saki F2017Iran	63	477	0.13	[0.10; 0.17]	0.0%	0.5%
Saliba W2012Israel	28567	198758	0.14	[0.14; 0.15]	2.6%	0.6%
Samefors M2014Sweden	48	333	0.14	[0.11; 0.19]	0.0%	0.5%
Santos A2017Portugal	594	1500	0.40	[0.37; 0.42]	0.0%	0.6%
Sarafin K2015Canada	839	11336	0.07	[0.07; 0.08]	0.1%	0.6%
Saraiva GL 2005Brazil	33	214	0.15	[0.11; 0.21]	0.0%	0.5%
Scalco R 2008Brazil	53	98	0.54	[0.44; 0.64]	0.0%	0.5%
Schramm S2017Germany	660	4149	0.16	[0.15; 0.17]	0.1%	0.6%
Science M2017Canada	4	743	0.01	[0.00; 0.01]	0.0%	0.5%
Shchubelka K2020Ukraine	211	1639	0.13	[0.11; 0.15]	0.0%	0.6%
Shetty S2014India	18	252	0.07	[0.04; 0.11]	0.0%	0.5%
Shivane VK2011India	220	1137	0.19	[0.17; 0.22]	0.0%	0.6%
Sioen I2012Belgium	18	357	0.05	[0.03; 0.08]	0.0%	0.5%
Skull SA2003Australia	61	116	0.53	[0.43; 0.62]	0.0%	0.5%
Smith G2016Cambodia	20	623	0.03	[0.02; 0.05]	0.0%	0.5%
Smith N2021UK	19	148	0.13	[0.08; 0.19]	0.0%	0.5%
Sochorová L2018Czech Republic	101	419	0.24	[0.20; 0.28]	0.0%	0.5%
Sokolovic S2017Bosnia and Herzegovina	513	1830	0.28	[0.26; 0.30]	0.0%	0.6%
Solis−Urra P2019Chile	385	1931	0.20	[0.18; 0.22]	0.0%	0.6%
Souberbielle JC2016France	56	892	0.06	[0.05; 0.08]	0.0%	0.5%
Sulimani RA2016Saudi Arabia	645	1618	0.40	[0.37; 0.42]	0.0%	0.6%
Thuesen B2012Denmark	848	6146	0.14	[0.13; 0.15]	0.1%	0.6%
Tolppanen AM2012UK	124	7560	0.02	[0.01; 0.02]	0.1%	0.6%
Tønnesen R2016Denmark	135	700	0.19	[0.16; 0.22]	0.0%	0.5%
Tran B2013Australia	66	653	0.10	[0.08; 0.13]	0.0%	0.5%
Tseng M2009USA	66	194	0.34	[0.27; 0.41]	0.0%	0.5%
Uush T2013Mongolia	679	1400	0.48	[0.46; 0.51]	0.0%	0.6%
von Hurst PR2010New Zealand	98	228	0.43	[0.36; 0.50]	0.0%	0.5%
Voortman T2015The Netherlands	258	4167	0.06	[0.05; 0.07]	0.1%	0.6%
Wyskida M2018Poland	355	3472	0.10	[0.09; 0.11]	0.0%	0.6%
Yan X2019China	81	302	0.27	[0.22; 0.32]	0.0%	0.5%
Yousef S2021Canada	1184	11579	0.10	[0.10; 0.11]	0.2%	0.6%
Yu S2015China	128	2173	0.06	[0.05; 0.07]	0.0%	0.6%
Zgaga L2011UK	772	2235	0.35	[0.33; 0.37]	0.0%	0.6%
Zhang FF2016Kuwait	348	960	0.36	[0.33; 0.39]	0.0%	0.6%
Zhou SJ2015Australia	9	221	0.04	[0.02; 0.08]	0.0%	0.5%
Zhu Z2012China	61	3892	0.02	[0.01; 0.02]	0.1%	0.6%
**Common effect model**		**7634261**	**0.32**	**[0.32; 0.32]**	**100.0%**	**--**
**Random effects model**			**0.16**	**[0.14; 0.18]**	**--**	**100.0%**
Heterogeneity: $I^2 = 100\%$, $\tau^2 = 0.0566$, $p = 0$						

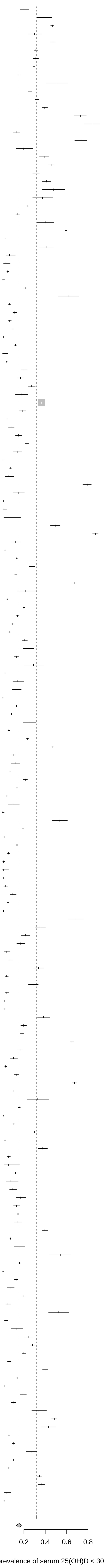

Supplementary figure 1.2 The global prevalence of serum 25(OH)D < 30 nmol/L

Appendix 4: The global prevalence of vitamin D deficiency

Study	Events	Total	Proportion	95%−CI	Weight (common)	Weight (random)
Houghton LA	2	433	0.00	[0.00; 0.02]	0.0%	0.3%
Smith G	3	41	0.07	[0.02; 0.20]	0.0%	0.3%
White Z	4	59	0.07	[0.02; 0.16]	0.0%	0.3%
Middelkoop K	4	168	0.02	[0.01; 0.06]	0.0%	0.3%
Lin L	6	353	0.02	[0.01; 0.04]	0.0%	0.3%
Middelkoop K	8	281	0.03	[0.01; 0.06]	0.0%	0.3%
Mutua AM	8	302	0.03	[0.01; 0.05]	0.0%	0.3%
Ten Haaf DSM	9	450	0.02	[0.01; 0.04]	0.0%	0.3%
Naqvi A	11	86	0.13	[0.07; 0.22]	0.0%	0.3%
Tangoh DA	12	372	0.03	[0.02; 0.06]	0.0%	0.3%
Miljkovic I	12	424	0.03	[0.01; 0.05]	0.0%	0.3%
Lategan R	14	339	0.04	[0.02; 0.07]	0.0%	0.3%
Ho−Pham LT	15	637	0.02	[0.01; 0.04]	0.0%	0.3%
Middelkoop K	18	346	0.05	[0.03; 0.08]	0.0%	0.3%
Lin L	20	477	0.04	[0.03; 0.06]	0.0%	0.3%
Riverin B	22	52	0.42	[0.29; 0.57]	0.0%	0.3%
Lin L	22	860	0.03	[0.02; 0.04]	0.1%	0.3%
Mechenro J	22	48	0.46	[0.31; 0.61]	0.0%	0.3%
Middelkoop K	22	108	0.20	[0.13; 0.29]	0.0%	0.3%
Shady MM	23	200	0.12	[0.07; 0.17]	0.0%	0.3%
Madsen KH	24	340	0.07	[0.05; 0.10]	0.0%	0.3%
Glatt DU	24	49	0.49	[0.34; 0.64]	0.0%	0.3%
Smith G	25	128	0.20	[0.13; 0.27]	0.0%	0.3%
Andersen R	25	52	0.48	[0.34; 0.62]	0.0%	0.3%
Kouda K	26	400	0.06	[0.04; 0.09]	0.0%	0.3%
Ginter JK	27	224	0.12	[0.08; 0.17]	0.0%	0.3%
Maguire JL	29	91	0.32	[0.22; 0.42]	0.0%	0.3%
Fayet−Moore F	30	103	0.29	[0.21; 0.39]	0.0%	0.3%
Abdulrahman MA	30	50	0.60	[0.45; 0.74]	0.0%	0.3%
Guo S	34	100	0.34	[0.25; 0.44]	0.0%	0.3%
Abdulrahman MA	34	63	0.54	[0.41; 0.67]	0.0%	0.3%
Bolland MJ	34	378	0.09	[0.06; 0.12]	0.0%	0.3%
Andersen R	35	37	0.95	[0.82; 0.99]	0.0%	0.3%
Middelkoop K	35	503	0.07	[0.05; 0.10]	0.0%	0.3%
Pan T	38	194	0.20	[0.14; 0.26]	0.0%	0.3%
Hatun S	39	89	0.44	[0.33; 0.55]	0.0%	0.3%
Yu L	40	1200	0.03	[0.02; 0.05]	0.1%	0.3%
Madsen KH	41	415	0.10	[0.07; 0.13]	0.0%	0.3%
Yousef S	42	267	0.16	[0.12; 0.21]	0.0%	0.3%
Zhou SJ	44	221	0.20	[0.15; 0.26]	0.0%	0.3%
Kagotho E	44	253	0.17	[0.13; 0.23]	0.0%	0.3%
Andersen R	47	54	0.87	[0.75; 0.95]	0.0%	0.3%
Maldonado G	47	269	0.17	[0.13; 0.23]	0.0%	0.3%
Bi X	48	114	0.42	[0.33; 0.52]	0.0%	0.3%
Middelkoop K	48	396	0.12	[0.09; 0.16]	0.0%	0.3%
Srimani S	49	222	0.22	[0.17; 0.28]	0.0%	0.3%
Pérez−Llamas F	50	86	0.58	[0.47; 0.69]	0.0%	0.3%
Chin KY	51	150	0.34	[0.26; 0.42]	0.0%	0.3%
González G	52	90	0.58	[0.47; 0.68]	0.0%	0.3%
Rodríguez−Rodríguez E	52	102	0.51	[0.41; 0.61]	0.0%	0.3%
Naeem Z	53	180	0.29	[0.23; 0.37]	0.0%	0.3%
Gill TK	54	263	0.21	[0.16; 0.26]	0.0%	0.3%
El Hayek J	54	508	0.11	[0.08; 0.14]	0.0%	0.3%
Hazell TJ	57	488	0.12	[0.09; 0.15]	0.0%	0.3%
Nälsén C	60	268	0.22	[0.18; 0.28]	0.0%	0.3%
Greene−Finestone LS	61	312	0.20	[0.15; 0.24]	0.0%	0.3%
Sharawat IK	63	100	0.63	[0.53; 0.72]	0.0%	0.3%
Smith G	64	495	0.13	[0.10; 0.16]	0.0%	0.3%
Gill TK	66	265	0.25	[0.20; 0.31]	0.0%	0.3%
Andersen R	67	221	0.30	[0.24; 0.37]	0.0%	0.3%
Lardner E	67	143	0.47	[0.38; 0.55]	0.0%	0.3%
Andersen S	68	97	0.70	[0.60; 0.79]	0.0%	0.3%
Hribar M	72	125	0.58	[0.48; 0.66]	0.0%	0.3%
Wakayo T	73	174	0.42	[0.35; 0.50]	0.0%	0.3%
Cabral MA	74	234	0.32	[0.26; 0.38]	0.0%	0.3%
Ramakrishnan S	74	237	0.31	[0.25; 0.38]	0.0%	0.3%
Paul TV	74	150	0.49	[0.41; 0.58]	0.0%	0.3%
Tran B	76	92	0.83	[0.73; 0.90]	0.0%	0.3%
Zargar AH	76	92	0.83	[0.73; 0.90]	0.0%	0.3%
Liu X	76	207	0.37	[0.30; 0.44]	0.0%	0.3%
Gill TK	83	390	0.21	[0.17; 0.26]	0.0%	0.3%
Scalco R	84	98	0.86	[0.77; 0.92]	0.0%	0.3%
Hirani V	85	174	0.49	[0.41; 0.57]	0.0%	0.3%
Santos BR	85	234	0.36	[0.30; 0.43]	0.0%	0.3%
Zhu Z	85	183	0.46	[0.39; 0.54]	0.0%	0.3%
Bodin J	85	95	0.89	[0.81; 0.95]	0.0%	0.3%
Yu L	87	1200	0.07	[0.06; 0.09]	0.1%	0.3%
Nälsén C	87	206	0.42	[0.35; 0.49]	0.0%	0.3%
Asakura K	88	107	0.82	[0.74; 0.89]	0.0%	0.3%
Hussain T	89	151	0.59	[0.51; 0.67]	0.0%	0.3%
Meyer HE	89	196	0.45	[0.38; 0.53]	0.0%	0.3%
Smith N	89	148	0.60	[0.52; 0.68]	0.0%	0.3%
Tran B	92	134	0.69	[0.60; 0.76]	0.0%	0.3%
Andersen R	92	199	0.46	[0.39; 0.53]	0.0%	0.3%
Mechenro J	92	101	0.91	[0.84; 0.96]	0.0%	0.3%
Bettencourt A	95	198	0.48	[0.41; 0.55]	0.0%	0.3%
Black LJ	96	331	0.29	[0.24; 0.34]	0.0%	0.3%
Alyahya KO	96	104	0.92	[0.85; 0.97]	0.0%	0.3%
Hribar M	98	155	0.63	[0.55; 0.71]	0.0%	0.3%
Vupputuri MR	99	105	0.94	[0.88; 0.98]	0.0%	0.3%
Cinar N	99	118	0.84	[0.76; 0.90]	0.0%	0.3%
Al−Kindi MK	100	100	1.00	[0.96; 1.00]	0.0%	0.3%
AlFaris NA	100	166	0.60	[0.52; 0.68]	0.0%	0.3%
Fernández Bustillo JM	101	153	0.66	[0.58; 0.73]	0.0%	0.3%
Tran B	102	131	0.78	[0.70; 0.85]	0.0%	0.3%
Feketea GM	102	376	0.27	[0.23; 0.32]	0.0%	0.3%
Herrick KA	102	1438	0.07	[0.06; 0.09]	0.1%	0.3%
Bjarnadottir A	103	158	0.65	[0.57; 0.73]	0.0%	0.3%
Brinkmann K	104	108	0.96	[0.91; 0.99]	0.0%	0.3%
Johnson MA	105	317	0.33	[0.28; 0.39]	0.0%	0.3%
Tran B	106	148	0.72	[0.64; 0.79]	0.0%	0.3%
Gill TK	107	500	0.21	[0.18; 0.25]	0.0%	0.3%
Tran B	107	148	0.72	[0.64; 0.79]	0.0%	0.3%
Skull SA	107	116	0.92	[0.86; 0.96]	0.0%	0.3%
Al Hayek S	108	344	0.31	[0.27; 0.37]	0.0%	0.3%
Gill TK	110	510	0.22	[0.18; 0.25]	0.0%	0.3%
Mogire RM	111	1507	0.07	[0.06; 0.09]	0.1%	0.3%
Horton−French K	112	692	0.16	[0.14; 0.19]	0.0%	0.3%
Horton−French K	112	400	0.28	[0.24; 0.33]	0.0%	0.3%
Abdulrahman MA	112	278	0.40	[0.34; 0.46]	0.0%	0.3%
Benjeddou K	114	239	0.48	[0.41; 0.54]	0.0%	0.3%
Orces CH	115	403	0.29	[0.24; 0.33]	0.0%	0.3%
Contreras−Manzano A	120	381	0.31	[0.27; 0.36]	0.0%	0.3%
Courraud J	121	177	0.68	[0.61; 0.75]	0.0%	0.3%
Meyer HE	122	869	0.14	[0.12; 0.17]	0.1%	0.3%
Saraiva GL	123	214	0.57	[0.51; 0.64]	0.0%	0.3%
Hirani V	124	304	0.41	[0.35; 0.47]	0.0%	0.3%
Contreras−Manzano A	127	404	0.31	[0.27; 0.36]	0.0%	0.3%
NHANES(2017...2018)	127	1321	0.10	[0.08; 0.11]	0.1%	0.3%
Chao YS	129	1493	0.09	[0.07; 0.10]	0.1%	0.3%
Gordon CM	129	307	0.42	[0.36; 0.48]	0.0%	0.3%
Djennane M	130	435	0.30	[0.26; 0.34]	0.0%	0.3%
Gill TK	130	486	0.27	[0.23; 0.31]	0.0%	0.3%
Shetty S	133	252	0.53	[0.46; 0.59]	0.0%	0.3%
Vallianou N	135	490	0.28	[0.24; 0.32]	0.0%	0.3%
Koyama S	138	492	0.28	[0.24; 0.32]	0.0%	0.3%
Muhairi SJ	143	315	0.45	[0.40; 0.51]	0.0%	0.3%
Janssen HC	144	400	0.36	[0.31; 0.41]	0.0%	0.3%
Jolliffe DA	144	222	0.65	[0.58; 0.71]	0.0%	0.3%
Black LJ	147	490	0.30	[0.26; 0.34]	0.0%	0.3%
Moussavi M	147	318	0.46	[0.41; 0.52]	0.0%	0.3%
Contreras−Manzano A	150	477	0.31	[0.27; 0.36]	0.0%	0.3%
Karin Z	151	260	0.58	[0.52; 0.64]	0.0%	0.3%
Chailurkit LO	151	2641	0.06	[0.05; 0.07]	0.2%	0.3%
Science M	152	743	0.20	[0.18; 0.24]	0.1%	0.3%
Sherchand O	154	300	0.51	[0.46; 0.57]	0.0%	0.3%
Greene−Finestone LS	155	823	0.19	[0.16; 0.22]	0.1%	0.3%
Junaid K	156	215	0.73	[0.66; 0.78]	0.0%	0.3%
Granlund L	157	216	0.73	[0.66; 0.79]	0.0%	0.3%
Le Goaziou MF	158	196	0.81	[0.74; 0.86]	0.0%	0.3%
Harinarayan CV	159	913	0.17	[0.15; 0.20]	0.1%	0.3%
Suryanarayana P	161	298	0.54	[0.48; 0.60]	0.0%	0.3%
Orces CH	162	724	0.22	[0.19; 0.26]	0.0%	0.3%
Mechenro J	162	275	0.59	[0.53; 0.65]	0.0%	0.3%
Robinson PJ	164	267	0.61	[0.55; 0.67]	0.0%	0.3%
Vasudevan B	164	184	0.89	[0.84; 0.93]	0.0%	0.3%
Gebreegziabher T	165	196	0.84	[0.78; 0.89]	0.0%	0.3%
Masoud MS	170	170	1.00	[0.98; 1.00]	0.0%	0.3%
NHANES(2015...2016)	170	1231	0.14	[0.12; 0.16]	0.1%	0.3%
Lappe JM	170	1179	0.14	[0.12; 0.17]	0.1%	0.3%
Black LJ	172	652	0.26	[0.23; 0.30]	0.0%	0.3%
Islam MZ	172	244	0.70	[0.64; 0.76]	0.0%	0.3%
Sheikh A	173	300	0.58	[0.52; 0.63]	0.0%	0.3%
Greene−Finestone LS	174	777	0.22	[0.20; 0.25]	0.1%	0.3%
Santos BR	176	443	0.40	[0.35; 0.44]	0.0%	0.3%
Sokolovic S	179	450	0.40	[0.35; 0.44]	0.0%	0.3%
Rabufetti A	179	1045	0.17	[0.15; 0.20]	0.1%	0.3%
Abiaka C	180	206	0.87	[0.82; 0.92]	0.0%	0.3%
Niafar M	184	300	0.61	[0.56; 0.67]	0.0%	0.3%
Al Shaikh AM	184	204	0.90	[0.85; 0.94]	0.0%	0.3%
Black LJ	187	648	0.29	[0.25; 0.33]	0.0%	0.3%
Black LJ	188	730	0.26	[0.23; 0.29]	0.0%	0.3%
von Hurst PR	191	228	0.84	[0.78; 0.88]	0.0%	0.3%
Bezrati I	191	225	0.85	[0.80; 0.89]	0.0%	0.3%
Batieha A	192	4590	0.04	[0.04; 0.05]	0.3%	0.3%
Nadeem S	197	221	0.89	[0.84; 0.93]	0.0%	0.3%
Ní Chaoimh C	198	741	0.27	[0.24; 0.30]	0.1%	0.3%
Harkness LS	200	370	0.54	[0.49; 0.59]	0.0%	0.3%
Andersen R	201	219	0.92	[0.87; 0.95]	0.0%	0.3%
Kunz C	205	327	0.63	[0.57; 0.68]	0.0%	0.3%
Sioen I	207	357	0.58	[0.53; 0.63]	0.0%	0.3%
Rafraf M	207	216	0.96	[0.92; 0.98]	0.0%	0.3%
Gilbert−Diamond D	208	479	0.43	[0.39; 0.48]	0.0%	0.3%
Flores ME	208	713	0.29	[0.26; 0.33]	0.0%	0.3%
Orces CH	211	1158	0.18	[0.16; 0.21]	0.1%	0.3%
Nakamura K	212	600	0.35	[0.32; 0.39]	0.0%	0.3%
Vierucci F	213	427	0.50	[0.45; 0.55]	0.0%	0.3%
Yan X	216	302	0.72	[0.66; 0.77]	0.0%	0.3%
Hirani V	217	522	0.42	[0.37; 0.46]	0.0%	0.3%
Sakyi SA	218	500	0.44	[0.39; 0.48]	0.0%	0.3%
Klenk J	228	1418	0.16	[0.14; 0.18]	0.1%	0.3%
Oliveri B	229	339	0.68	[0.62; 0.73]	0.0%	0.3%
Alyahya K	229	232	0.99	[0.96; 1.00]	0.0%	0.3%
Yousef S	231	932	0.25	[0.22; 0.28]	0.1%	0.3%
Man PW	236	416	0.57	[0.52; 0.62]	0.0%	0.3%
Mitchell DM	245	634	0.39	[0.35; 0.43]	0.0%	0.3%
Matheï C	248	367	0.68	[0.63; 0.72]	0.0%	0.3%
Sochorová L	252	419	0.60	[0.55; 0.65]	0.0%	0.3%
Zhu W	254	508	0.50	[0.46; 0.54]	0.0%	0.3%
Gill TK	256	2413	0.11	[0.09; 0.12]	0.2%	0.3%
Moy FM	258	380	0.68	[0.63; 0.73]	0.0%	0.3%
Mallah EM	263	300	0.88	[0.83; 0.91]	0.0%	0.3%
Karagüzel G	265	746	0.36	[0.32; 0.39]	0.1%	0.3%
Alloubani A	266	350	0.76	[0.71; 0.80]	0.0%	0.3%
Samefors M	267	333	0.80	[0.75; 0.84]	0.0%	0.3%
Kull M Jr	268	367	0.73	[0.68; 0.77]	0.0%	0.3%
Mansbach JM	268	1799	0.15	[0.13; 0.17]	0.1%	0.3%
Khan AH	275	305	0.90	[0.86; 0.93]	0.0%	0.3%
Aucoin M	279	461	0.61	[0.56; 0.65]	0.0%	0.3%
Jääskeläinen T	281	4051	0.07	[0.06; 0.08]	0.3%	0.3%
Herrick KA	282	2060	0.14	[0.12; 0.15]	0.1%	0.3%
Kunz C	283	456	0.62	[0.57; 0.67]	0.0%	0.3%
Gariballa S	286	648	0.44	[0.40; 0.48]	0.0%	0.3%
Hirani V	287	659	0.44	[0.40; 0.47]	0.0%	0.3%
Majumdar V	289	441	0.66	[0.61; 0.70]	0.0%	0.3%
Hekimsoy Z	293	391	0.75	[0.70; 0.79]	0.0%	0.3%
Nakhaee S	296	400	0.74	[0.69; 0.78]	0.0%	0.3%
de Oliveira CL	298	1152	0.26	[0.23; 0.28]	0.1%	0.3%
Yousef S	305	1361	0.22	[0.20; 0.25]	0.1%	0.3%
Moreno−Reyes R	306	401	0.76	[0.72; 0.80]	0.0%	0.3%
Kapil U	307	374	0.82	[0.78; 0.86]	0.0%	0.3%
Daly RM	308	1393	0.22	[0.20; 0.24]	0.1%	0.3%
Souberbielle JC	309	892	0.35	[0.32; 0.38]	0.1%	0.3%
Bener A	315	458	0.69	[0.64; 0.73]	0.0%	0.3%
Aucoin M	318	756	0.42	[0.39; 0.46]	0.1%	0.3%
Kunz C	318	520	0.61	[0.57; 0.65]	0.0%	0.3%
Hansen L	321	527	0.61	[0.57; 0.65]	0.0%	0.3%
Kunz C	325	528	0.62	[0.57; 0.66]	0.0%	0.3%
NHANES (2015...2016)	332	1777	0.19	[0.17; 0.21]	0.1%	0.3%
González−Molero I	334	1262	0.26	[0.24; 0.29]	0.1%	0.3%
Arabi A	335	466	0.72	[0.68; 0.76]	0.0%	0.3%
NHANES(2017...2018)	336	1810	0.19	[0.17; 0.20]	0.1%	0.3%
Robinson PJ	337	640	0.53	[0.49; 0.57]	0.0%	0.3%
Yousef S	346	858	0.40	[0.37; 0.44]	0.1%	0.3%
Misra P	346	381	0.91	[0.87; 0.94]	0.0%	0.3%
Rahman A	352	410	0.86	[0.82; 0.89]	0.0%	0.3%
Daly RM	354	810	0.44	[0.40; 0.47]	0.1%	0.3%
Sokolovic S	357	645	0.55	[0.51; 0.59]	0.0%	0.3%
Feng X	359	686	0.52	[0.49; 0.56]	0.0%	0.3%
Dalgård C	359	669	0.54	[0.50; 0.57]	0.0%	0.3%
Lima−Costa MF	362	2264	0.16	[0.15; 0.18]	0.2%	0.3%
Tønnesen R	373	700	0.53	[0.50; 0.57]	0.0%	0.3%
Drali O	375	621	0.60	[0.56; 0.64]	0.0%	0.3%
Saki F	388	477	0.81	[0.78; 0.85]	0.0%	0.3%
Hoge A	405	915	0.44	[0.41; 0.48]	0.1%	0.3%
Al−Daghri NM	408	561	0.73	[0.69; 0.76]	0.0%	0.3%
Rahmadhani R	410	1011	0.41	[0.38; 0.44]	0.1%	0.3%
Orwoll E	413	1606	0.26	[0.24; 0.28]	0.1%	0.3%
Ganmaa D	415	420	0.99	[0.97; 1.00]	0.0%	0.3%
NHANES(2017...2018)	415	2184	0.19	[0.17; 0.21]	0.1%	0.3%
Isa H	416	531	0.78	[0.75; 0.82]	0.0%	0.3%
Carrillo−Vega MF	416	1128	0.37	[0.34; 0.40]	0.1%	0.3%
Li S	417	578	0.72	[0.68; 0.76]	0.0%	0.3%
Sokolovic S	423	735	0.58	[0.54; 0.61]	0.0%	0.3%
González−Gross M	427	1006	0.42	[0.39; 0.46]	0.1%	0.3%
Raposo L	428	500	0.86	[0.82; 0.89]	0.0%	0.3%
Ardawi MS	429	843	0.51	[0.47; 0.54]	0.1%	0.3%
Golbahar J	432	500	0.86	[0.83; 0.89]	0.0%	0.3%
Nimitphong H	433	1990	0.22	[0.20; 0.24]	0.1%	0.3%
Solis−Urra P	445	686	0.65	[0.61; 0.68]	0.0%	0.3%
Arnljots R	448	545	0.82	[0.79; 0.85]	0.0%	0.3%
Cashman KD	453	1132	0.40	[0.37; 0.43]	0.1%	0.3%
Riverin B	457	944	0.48	[0.45; 0.52]	0.1%	0.3%
Herrick KA	496	3267	0.15	[0.14; 0.16]	0.2%	0.3%
Zhu Z	498	2269	0.22	[0.20; 0.24]	0.2%	0.3%
NHANES(2015...2016)	500	2618	0.19	[0.18; 0.21]	0.2%	0.3%
Marzban M	505	1806	0.28	[0.26; 0.30]	0.1%	0.3%
Nichols EK	517	915	0.57	[0.53; 0.60]	0.1%	0.3%
Hovsepian S	522	1111	0.47	[0.44; 0.50]	0.1%	0.3%
Oberg J	536	890	0.60	[0.57; 0.63]	0.1%	0.3%
Mehboobali N	541	858	0.63	[0.60; 0.66]	0.1%	0.3%
Sherief LM	542	572	0.95	[0.93; 0.96]	0.0%	0.3%
Daly RM	545	1564	0.35	[0.32; 0.37]	0.1%	0.3%
Moy FM	557	770	0.72	[0.69; 0.75]	0.1%	0.3%
Mansbach JM	578	2759	0.21	[0.19; 0.23]	0.2%	0.3%
Cougnard−Grégoire A	580	697	0.83	[0.80; 0.86]	0.0%	0.3%
Zhu Z	582	1440	0.40	[0.38; 0.43]	0.1%	0.3%
Flores ME	599	3482	0.17	[0.16; 0.18]	0.2%	0.3%
Chlebna−Sokó.. D	606	720	0.84	[0.81; 0.87]	0.0%	0.3%
Kapil U	618	848	0.73	[0.70; 0.76]	0.1%	0.3%
Al−Daghri NM	619	830	0.75	[0.71; 0.78]	0.1%	0.3%
Nikooyeh B	622	667	0.93	[0.91; 0.95]	0.0%	0.3%
NHANES(2017...2018)	633	2094	0.30	[0.28; 0.32]	0.1%	0.3%
Solis−Urra P	642	1245	0.52	[0.49; 0.54]	0.1%	0.3%
Cairncross CT	642	1329	0.48	[0.46; 0.51]	0.1%	0.3%
Herrick KA	648	2355	0.28	[0.26; 0.29]	0.2%	0.3%
Hutchings N	651	1206	0.54	[0.51; 0.57]	0.1%	0.3%
Capuano R	686	1200	0.57	[0.54; 0.60]	0.1%	0.3%
Chung IH	710	1212	0.59	[0.56; 0.61]	0.1%	0.3%
Al−Daghri NM	711	808	0.88	[0.86; 0.90]	0.1%	0.3%
Daly RM	742	2011	0.37	[0.35; 0.39]	0.1%	0.3%
Alkerwi A	746	1335	0.56	[0.53; 0.59]	0.1%	0.3%
NHANES(2015...2016)	753	2413	0.31	[0.29; 0.33]	0.2%	0.3%
Karonova T	760	1664	0.46	[0.43; 0.48]	0.1%	0.3%
Daly RM	762	2556	0.30	[0.28; 0.32]	0.2%	0.3%
Lucas JA	781	1606	0.49	[0.46; 0.51]	0.1%	0.3%
Zhang FF	795	960	0.83	[0.80; 0.85]	0.1%	0.3%
Shivane VK	805	1137	0.71	[0.68; 0.73]	0.1%	0.3%
Li L	806	2317	0.35	[0.33; 0.37]	0.2%	0.3%
Jayashri R	823	1500	0.55	[0.52; 0.57]	0.1%	0.3%
Kaykhaei MA	846	993	0.85	[0.83; 0.87]	0.1%	0.3%
Shchubelka K	847	1639	0.52	[0.49; 0.54]	0.1%	0.3%
Herrick KA	849	3496	0.24	[0.23; 0.26]	0.2%	0.3%
Seo JA	856	1081	0.79	[0.77; 0.82]	0.1%	0.3%
Ikonen H	857	3650	0.23	[0.22; 0.25]	0.2%	0.3%
Öztürk ZA	877	1161	0.76	[0.73; 0.78]	0.1%	0.3%
Penrose K	929	2160	0.43	[0.41; 0.45]	0.1%	0.3%
Ardawi MS	934	1172	0.80	[0.77; 0.82]	0.1%	0.3%
Ceccarelli M	939	2140	0.44	[0.42; 0.46]	0.1%	0.3%
Gromova O	943	1347	0.70	[0.67; 0.72]	0.1%	0.3%
Fang F	949	1814	0.52	[0.50; 0.55]	0.1%	0.3%
Hirani V	968	2070	0.47	[0.45; 0.49]	0.1%	0.3%
Daly RM	983	2884	0.34	[0.32; 0.36]	0.2%	0.3%
Santos A	1035	1500	0.69	[0.67; 0.71]	0.1%	0.3%
Jorde R	1084	2668	0.41	[0.39; 0.43]	0.2%	0.3%
Petrenya N	1103	4465	0.25	[0.23; 0.26]	0.3%	0.3%
Aleteng Q	1105	1829	0.60	[0.58; 0.63]	0.1%	0.3%
Herrick KA	1119	3564	0.31	[0.30; 0.33]	0.2%	0.3%
Al−Taiar A	1140	1416	0.81	[0.78; 0.83]	0.1%	0.3%
Al−Saleh Y	1187	2226	0.53	[0.51; 0.55]	0.2%	0.3%
Sulimani RA	1191	1618	0.74	[0.71; 0.76]	0.1%	0.3%
Rabenberg M	1201	1816	0.66	[0.64; 0.68]	0.1%	0.3%
Yu S	1215	2173	0.56	[0.54; 0.58]	0.1%	0.3%
Manios Y	1235	2353	0.52	[0.50; 0.55]	0.2%	0.3%
Voortman T	1241	4167	0.30	[0.28; 0.31]	0.3%	0.3%
Hansen L	1281	2565	0.50	[0.48; 0.52]	0.2%	0.3%
Nikooyeh B	1318	1406	0.94	[0.92; 0.95]	0.1%	0.3%
Beer RJ	1346	6470	0.21	[0.20; 0.22]	0.4%	0.3%
Rabenberg M	1439	2456	0.59	[0.57; 0.61]	0.2%	0.3%
Jayatissa R	1484	2525	0.59	[0.57; 0.61]	0.2%	0.3%
Borissova AM	1528	2016	0.76	[0.74; 0.78]	0.1%	0.3%
Nielsen NO	1626	2877	0.57	[0.55; 0.58]	0.2%	0.3%
Zhao Y	1677	2661	0.63	[0.61; 0.65]	0.2%	0.3%
Yang K	1681	1982	0.85	[0.83; 0.86]	0.1%	0.3%
AlQuaiz AM	1686	2832	0.60	[0.58; 0.61]	0.2%	0.3%
Metwally ASM	1692	1864	0.91	[0.89; 0.92]	0.1%	0.3%
Rabenberg M	1700	2723	0.62	[0.61; 0.64]	0.2%	0.3%
Beer RJ	1706	7170	0.24	[0.23; 0.25]	0.5%	0.3%
Chirita−Emandi A	1731	6631	0.26	[0.25; 0.27]	0.4%	0.3%
Zgaga L	1732	2235	0.77	[0.76; 0.79]	0.2%	0.3%
Patel JV	1732	1904	0.91	[0.90; 0.92]	0.1%	0.3%
Kim YS	1805	2314	0.78	[0.76; 0.80]	0.2%	0.3%
Al Shaikh AM	1829	1906	0.96	[0.95; 0.97]	0.1%	0.3%
Byun EJ	1843	2515	0.73	[0.72; 0.75]	0.2%	0.3%
Beer RJ	1887	6813	0.28	[0.27; 0.29]	0.5%	0.3%
Al−Daghri NM	1901	2225	0.85	[0.84; 0.87]	0.2%	0.3%
Schramm S	2104	4149	0.51	[0.49; 0.52]	0.3%	0.3%
Tolppanen AM	2158	7560	0.29	[0.28; 0.30]	0.5%	0.3%
Duarte C	2170	3092	0.70	[0.69; 0.72]	0.2%	0.3%
Laird E	2303	5356	0.43	[0.42; 0.44]	0.4%	0.3%
Leung RY	2309	5276	0.44	[0.42; 0.45]	0.4%	0.3%
Chen J	2491	6014	0.41	[0.40; 0.43]	0.4%	0.3%
Al−Daghri NM	2609	3111	0.84	[0.83; 0.85]	0.2%	0.3%
Joukar F	2729	5096	0.54	[0.52; 0.55]	0.3%	0.3%
Liu X	2753	8182	0.34	[0.33; 0.35]	0.6%	0.3%
Al−Daghri NM	2780	4247	0.65	[0.64; 0.67]	0.3%	0.3%
Liu X	2811	7485	0.38	[0.36; 0.39]	0.5%	0.3%
El−Khateeb M	2815	4056	0.69	[0.68; 0.71]	0.3%	0.3%
Saliba W	3014	6518	0.46	[0.45; 0.47]	0.4%	0.3%
Ward M	3107	6154	0.50	[0.49; 0.52]	0.4%	0.3%
Thuesen B	3208	6146	0.52	[0.51; 0.53]	0.4%	0.3%
Yousef S	3248	8161	0.40	[0.39; 0.41]	0.6%	0.3%
Al Shaikh A	3289	3613	0.91	[0.90; 0.92]	0.2%	0.3%
Aspell N	3317	6004	0.55	[0.54; 0.57]	0.4%	0.3%
Gökta O	3454	11893	0.29	[0.28; 0.30]	0.8%	0.3%
Beer RJ	3587	16454	0.22	[0.21; 0.22]	1.1%	0.3%
Perna L	3677	5386	0.68	[0.67; 0.70]	0.4%	0.3%
Li H	3907	4989	0.78	[0.77; 0.79]	0.3%	0.3%
Li H	4078	5707	0.71	[0.70; 0.73]	0.4%	0.3%
Sarafin K	4172	11336	0.37	[0.36; 0.38]	0.8%	0.3%
Liu X	4200	10343	0.41	[0.40; 0.42]	0.7%	0.3%
Han B	5496	6597	0.83	[0.82; 0.84]	0.4%	0.3%
Hintzpeter B	5895	9001	0.65	[0.65; 0.66]	0.6%	0.3%
Song HR	6802	8976	0.76	[0.75; 0.77]	0.6%	0.3%
Jiang W	7185	14302	0.50	[0.49; 0.51]	1.0%	0.3%
Zhen D	7544	10100	0.75	[0.74; 0.76]	0.7%	0.3%
Griffin TP	7610	15319	0.50	[0.49; 0.50]	1.0%	0.3%
Al Zarooni AAR	10314	12346	0.84	[0.83; 0.84]	0.8%	0.3%
Chung JY	11587	18305	0.63	[0.63; 0.64]	1.2%	0.3%
Eloi M	13222	39004	0.34	[0.33; 0.34]	2.6%	0.3%
Lee J	50995	68457	0.74	[0.74; 0.75]	4.6%	0.3%
Kim SY	79950	157211	0.51	[0.51; 0.51]	10.6%	0.3%
Saliba W	96055	192240	0.50	[0.50; 0.50]	13.0%	0.3%
Lin LY	248478	448601	0.55	[0.55; 0.56]	30.4%	0.3%
**Common effect model**		**1476846**	**0.51**	**[0.51; 0.51]**	**100.0%**	**−−**
**Random effects model**			**0.48**	**[0.45; 0.51]**	**−−**	**100.0%**

Heterogeneity: $I^2 = 100\%$, $\tau^2 = 0.0875$, $p = 0$

Supplementary figure 2 The global prevalence of serum 25(OH)D < 50 nmol/L

Appendix 4: The global prevalence of vitamin D deficiency

Study	Events	Total	Proportion	95%−CI	Weight (common)	Weight (random)
Chailurkit LO	1194	2641	0.45	[0.43; 0.47]	0.5%	0.5%
Contreras−Manzano A	324	404	0.80	[0.76; 0.84]	0.1%	0.5%
Contreras−Manzano A	287	381	0.75	[0.71; 0.80]	0.1%	0.5%
Contreras−Manzano A	367	477	0.77	[0.73; 0.81]	0.1%	0.5%
Flores ME	520	713	0.73	[0.70; 0.76]	0.1%	0.5%
Flores ME	2612	3482	0.75	[0.74; 0.76]	0.6%	0.5%
Gilbert−Diamond D	430	479	0.90	[0.87; 0.92]	0.1%	0.5%
Harinarayan CV	328	913	0.36	[0.33; 0.39]	0.2%	0.5%
Ho−Pham LT	240	637	0.38	[0.34; 0.42]	0.1%	0.5%
Jayashri R	1360	1500	0.91	[0.89; 0.92]	0.3%	0.5%
Miljkovic I	114	424	0.27	[0.23; 0.31]	0.1%	0.5%
Mutua AM	113	302	0.37	[0.32; 0.43]	0.1%	0.5%
Naqvi A	55	86	0.64	[0.53; 0.74]	0.0%	0.4%
Shivane VK	1055	1137	0.93	[0.91; 0.94]	0.2%	0.5%
Smith G	4	41	0.10	[0.03; 0.23]	0.0%	0.4%
Smith G	54	128	0.42	[0.34; 0.51]	0.0%	0.4%
Smith G	185	495	0.37	[0.33; 0.42]	0.1%	0.5%
Tangoh DA	96	372	0.26	[0.21; 0.31]	0.1%	0.5%
Lin L	61	353	0.17	[0.13; 0.22]	0.1%	0.5%
Lin L	161	477	0.34	[0.30; 0.38]	0.1%	0.5%
Lin L	173	860	0.20	[0.17; 0.23]	0.2%	0.5%
Abdulrahman MA	40	50	0.80	[0.66; 0.90]	0.0%	0.4%
Abdulrahman MA	45	63	0.71	[0.59; 0.82]	0.0%	0.4%
Abdulrahman MA	161	278	0.58	[0.52; 0.64]	0.0%	0.5%
Abiaka C	202	206	0.98	[0.95; 0.99]	0.0%	0.4%
Abu−Samak MS	338	371	0.91	[0.88; 0.94]	0.1%	0.5%
Al Hayek S	214	344	0.62	[0.57; 0.67]	0.1%	0.5%
Al Zarooni AAR	11765	12346	0.95	[0.95; 0.96]	2.2%	0.5%
Aleteng Q	1659	1829	0.91	[0.89; 0.92]	0.3%	0.5%
AlQuaiz AM	2215	2832	0.78	[0.77; 0.80]	0.5%	0.5%
Al−Taiar A	1365	1416	0.96	[0.95; 0.97]	0.3%	0.5%
Arabi A	427	466	0.92	[0.89; 0.94]	0.1%	0.5%
Ardawi MS	1034	1172	0.88	[0.86; 0.90]	0.2%	0.5%
Ardawi MS	510	843	0.60	[0.57; 0.64]	0.2%	0.5%
Bachhel R	135	150	0.90	[0.84; 0.94]	0.0%	0.4%
Batieha A	991	4590	0.22	[0.20; 0.23]	0.8%	0.5%
Bhatt SP	120	137	0.88	[0.81; 0.93]	0.0%	0.4%
Capuano R	1053	1200	0.88	[0.86; 0.90]	0.2%	0.5%
Choi HR	626	651	0.96	[0.94; 0.97]	0.1%	0.5%
Chung IH	1177	1212	0.97	[0.96; 0.98]	0.2%	0.5%
Chung JY	16973	18305	0.93	[0.92; 0.93]	3.3%	0.5%
Cinar N	114	118	0.97	[0.92; 0.99]	0.0%	0.4%
Djennane M	254	435	0.58	[0.54; 0.63]	0.1%	0.5%
Drali O	528	621	0.85	[0.82; 0.88]	0.1%	0.5%
Duarte C	3000	3092	0.97	[0.96; 0.98]	0.6%	0.5%
El−Khateeb M	3548	4056	0.87	[0.86; 0.88]	0.7%	0.5%
Feketea GM	249	376	0.66	[0.61; 0.71]	0.1%	0.5%
Feng X	572	686	0.83	[0.80; 0.86]	0.1%	0.5%
González−Molero I	968	1262	0.77	[0.74; 0.79]	0.2%	0.5%
Hekimsoy Z	347	391	0.89	[0.85; 0.92]	0.1%	0.5%
Hovsepian S	725	1111	0.65	[0.62; 0.68]	0.2%	0.5%
Isa H	496	531	0.93	[0.91; 0.95]	0.1%	0.5%
Jiang W	11874	14302	0.83	[0.82; 0.84]	2.5%	0.5%
Kapil U	356	374	0.95	[0.93; 0.97]	0.1%	0.5%
Kapil U	767	848	0.90	[0.88; 0.92]	0.2%	0.5%
Kaykhaei MA	940	993	0.95	[0.93; 0.96]	0.2%	0.5%
Khan AH	292	305	0.96	[0.93; 0.98]	0.1%	0.5%
Kouda K	198	400	0.50	[0.44; 0.55]	0.1%	0.5%
Koyama S	486	492	0.99	[0.97; 1.00]	0.1%	0.5%
Kremer R	53	90	0.59	[0.48; 0.69]	0.0%	0.4%
Leung RY	4752	5276	0.90	[0.89; 0.91]	0.9%	0.5%
Li L	1683	2317	0.73	[0.71; 0.74]	0.4%	0.5%
Liu X	5785	7485	0.77	[0.76; 0.78]	1.3%	0.5%
Liu X	6041	8182	0.74	[0.73; 0.75]	1.5%	0.5%
Liu X	7985	10343	0.77	[0.76; 0.78]	1.8%	0.5%
Mallah EM	292	300	0.97	[0.95; 0.99]	0.1%	0.5%
Manios Y	2266	2353	0.96	[0.95; 0.97]	0.4%	0.5%
Mansbach JM	1131	1799	0.63	[0.61; 0.65]	0.3%	0.5%
Mansbach JM	2013	2759	0.73	[0.71; 0.75]	0.5%	0.5%
Mehboobali N	810	858	0.94	[0.93; 0.96]	0.2%	0.5%
Nadeem S	213	221	0.96	[0.93; 0.98]	0.0%	0.4%
Naeem Z	121	180	0.67	[0.60; 0.74]	0.0%	0.4%
Öztürk ZA	1102	1161	0.95	[0.93; 0.96]	0.2%	0.5%
Pan T	137	194	0.71	[0.64; 0.77]	0.0%	0.4%
Qorbani M	1846	2596	0.71	[0.69; 0.73]	0.5%	0.5%
Rafraf M	215	216	1.00	[0.97; 1.00]	0.0%	0.4%
Rahman A	393	410	0.96	[0.93; 0.98]	0.1%	0.5%
Ramakrishnan S	117	237	0.49	[0.43; 0.56]	0.0%	0.5%
Saeed BQ	244	287	0.85	[0.80; 0.89]	0.1%	0.5%
Saki F	458	477	0.96	[0.94; 0.98]	0.1%	0.5%
Saliba W	5237	6518	0.80	[0.79; 0.81]	1.2%	0.5%
Saliba W	160952	192240	0.84	[0.84; 0.84]	34.2%	0.5%
Sheikh A	253	300	0.84	[0.80; 0.88]	0.1%	0.5%
Sherchand O	236	300	0.79	[0.74; 0.83]	0.1%	0.5%
Sherief LM	566	572	0.99	[0.98; 1.00]	0.1%	0.5%
Song HR	8720	8976	0.97	[0.97; 0.97]	1.6%	0.5%
Srimani S	167	222	0.75	[0.69; 0.81]	0.0%	0.4%
Tseng M	192	194	0.99	[0.96; 1.00]	0.0%	0.4%
Vallianou N	352	490	0.72	[0.68; 0.76]	0.1%	0.5%
Yang K	1893	1982	0.96	[0.95; 0.96]	0.4%	0.5%
Zhao Y	2523	2661	0.95	[0.94; 0.96]	0.5%	0.5%
Zhu W	227	508	0.45	[0.40; 0.49]	0.1%	0.5%
Zhu Z	164	183	0.90	[0.84; 0.94]	0.0%	0.4%
Zhu Z	1271	1440	0.88	[0.86; 0.90]	0.3%	0.5%
Zhu Z	1555	2269	0.69	[0.67; 0.70]	0.4%	0.5%
Alkerwi A	1107	1335	0.83	[0.81; 0.85]	0.2%	0.5%
Arnljots R	510	545	0.94	[0.91; 0.95]	0.1%	0.5%
Aucoin M	405	461	0.88	[0.85; 0.91]	0.1%	0.5%
Aucoin M	617	756	0.82	[0.79; 0.84]	0.1%	0.5%
Bettencourt A	155	198	0.78	[0.72; 0.84]	0.0%	0.4%
Bhattoa HP	109	206	0.53	[0.46; 0.60]	0.0%	0.4%
Ceccarelli M	1657	2140	0.77	[0.76; 0.79]	0.4%	0.5%
Chao YS	2896	6101	0.47	[0.46; 0.49]	1.1%	0.5%
Chirita−Emandi A	3926	6631	0.59	[0.58; 0.60]	1.2%	0.5%
El Hayek J	257	508	0.51	[0.46; 0.55]	0.1%	0.5%
Ganmaa D	419	420	1.00	[0.99; 1.00]	0.1%	0.5%
Ginter JK	87	224	0.39	[0.32; 0.46]	0.0%	0.5%
González−Gross M	817	1006	0.81	[0.79; 0.84]	0.2%	0.5%
Greene−Finestone LS	185	312	0.59	[0.54; 0.65]	0.1%	0.5%
Greene−Finestone LS	484	777	0.62	[0.59; 0.66]	0.1%	0.5%
Greene−Finestone LS	464	823	0.56	[0.53; 0.60]	0.1%	0.5%
Gromova O	1232	1347	0.91	[0.90; 0.93]	0.2%	0.5%
Hansen L	482	527	0.91	[0.89; 0.94]	0.1%	0.5%
Hansen L	2045	2565	0.80	[0.78; 0.81]	0.5%	0.5%
Hazell TJ	203	488	0.42	[0.37; 0.46]	0.1%	0.5%
Hintzpeter B	7911	9001	0.88	[0.87; 0.89]	1.6%	0.5%
Hirani V	1788	2070	0.86	[0.85; 0.88]	0.4%	0.5%
Hoge A	719	915	0.79	[0.76; 0.81]	0.2%	0.5%
Hribar M	104	125	0.83	[0.75; 0.89]	0.0%	0.4%
Hribar M	131	155	0.85	[0.78; 0.90]	0.0%	0.4%
Jääskeläinen T	3068	4051	0.76	[0.74; 0.77]	0.7%	0.5%
Janssen HC	276	400	0.69	[0.64; 0.74]	0.1%	0.5%
Jolliffe DA	205	222	0.92	[0.88; 0.95]	0.0%	0.4%
Karagüzel G	350	746	0.47	[0.43; 0.51]	0.1%	0.5%
Karin Z	226	260	0.87	[0.82; 0.91]	0.0%	0.5%
Karonova T	1384	1664	0.83	[0.81; 0.85]	0.3%	0.5%
Klenk J	291	1418	0.21	[0.18; 0.23]	0.3%	0.5%
Kunz C	400	456	0.88	[0.84; 0.91]	0.1%	0.5%
Kunz C	469	528	0.89	[0.86; 0.91]	0.1%	0.5%
Kunz C	453	520	0.87	[0.84; 0.90]	0.1%	0.5%
Kunz C	241	327	0.74	[0.69; 0.78]	0.1%	0.5%
Langlois K	3428	5306	0.65	[0.63; 0.66]	0.9%	0.5%
Le Goaziou MF	188	196	0.96	[0.92; 0.98]	0.0%	0.4%
Madsen KH	197	340	0.58	[0.52; 0.63]	0.1%	0.5%
Madsen KH	211	415	0.51	[0.46; 0.56]	0.1%	0.5%
Maguire JL	75	91	0.82	[0.73; 0.90]	0.0%	0.4%
Man PW	332	416	0.80	[0.76; 0.84]	0.1%	0.5%
Matheï C	323	367	0.88	[0.84; 0.91]	0.1%	0.5%
Mitchell DM	407	634	0.64	[0.60; 0.68]	0.1%	0.5%
NHANES (2015...2016)	992	1777	0.56	[0.53; 0.58]	0.3%	0.5%
NHANES(2015...2016)	1730	2413	0.72	[0.70; 0.73]	0.4%	0.5%
NHANES(2015...2016)	474	1231	0.39	[0.36; 0.41]	0.2%	0.5%
NHANES(2015...2016)	1833	2618	0.70	[0.68; 0.72]	0.5%	0.5%
Ní Chaoimh C	530	741	0.72	[0.68; 0.75]	0.1%	0.5%
Orwoll E	1142	1606	0.71	[0.69; 0.73]	0.3%	0.5%
Penrose K	1685	2160	0.78	[0.76; 0.80]	0.4%	0.5%
Rabenberg M	1687	1816	0.93	[0.92; 0.94]	0.3%	0.5%
Rabenberg M	2465	2723	0.91	[0.89; 0.92]	0.5%	0.5%
Rabenberg M	2054	2456	0.84	[0.82; 0.85]	0.4%	0.5%
Riverin B	834	944	0.88	[0.86; 0.90]	0.2%	0.5%
Riverin B	41	52	0.79	[0.65; 0.89]	0.0%	0.4%
Schramm S	3456	4149	0.83	[0.82; 0.84]	0.7%	0.5%
Science M	565	743	0.76	[0.73; 0.79]	0.1%	0.5%
Sioen I	350	357	0.98	[0.96; 0.99]	0.1%	0.5%
Sokolovic S	659	735	0.90	[0.87; 0.92]	0.1%	0.5%
Sokolovic S	513	645	0.80	[0.76; 0.83]	0.1%	0.5%
Sokolovic S	263	450	0.58	[0.54; 0.63]	0.1%	0.5%
Souberbielle JC	716	892	0.80	[0.78; 0.83]	0.2%	0.5%
Ten Haaf DSM	108	450	0.24	[0.20; 0.28]	0.1%	0.5%
Tolppanen AM	5631	7560	0.74	[0.73; 0.75]	1.3%	0.5%
Vierucci F	351	427	0.82	[0.78; 0.86]	0.1%	0.5%
Voortman T	3020	4167	0.72	[0.71; 0.74]	0.7%	0.5%
Wyskida M	3002	3472	0.86	[0.85; 0.88]	0.6%	0.5%
Yousef S	179	267	0.67	[0.61; 0.73]	0.0%	0.5%
Yousef S	723	858	0.84	[0.82; 0.87]	0.2%	0.5%
Yousef S	727	932	0.78	[0.75; 0.81]	0.2%	0.5%
Yousef S	799	1361	0.59	[0.56; 0.61]	0.2%	0.5%
Yousef S	6386	8161	0.78	[0.77; 0.79]	1.5%	0.5%
NHANES(2017...2018)	1533	2094	0.73	[0.71; 0.75]	0.4%	0.5%
NHANES(2017...2018)	1500	2184	0.69	[0.67; 0.71]	0.4%	0.5%
NHANES(2017...2018)	950	1810	0.52	[0.50; 0.55]	0.3%	0.5%
NHANES(2017...2018)	439	1321	0.33	[0.31; 0.36]	0.2%	0.5%
El Hayek J	178	282	0.63	[0.57; 0.69]	0.1%	0.5%
Granlund L	208	216	0.96	[0.93; 0.98]	0.0%	0.4%
Ikonen H	2597	3650	0.71	[0.70; 0.73]	0.6%	0.5%
Oberg J	780	890	0.88	[0.85; 0.90]	0.2%	0.5%
Petrenya N	3313	4465	0.74	[0.73; 0.75]	0.8%	0.5%
Samefors M	318	333	0.95	[0.93; 0.97]	0.1%	0.5%
Cabral MA	156	234	0.67	[0.60; 0.73]	0.0%	0.5%
de Oliveira CL	777	1152	0.67	[0.65; 0.70]	0.2%	0.5%
Kagotho E	152	253	0.60	[0.54; 0.66]	0.0%	0.5%
Lima−Costa MF	1404	2264	0.62	[0.60; 0.64]	0.4%	0.5%
Maldonado G	188	269	0.70	[0.64; 0.75]	0.0%	0.5%
Orces CH	510	724	0.70	[0.67; 0.74]	0.1%	0.5%
Orces CH	277	403	0.69	[0.64; 0.73]	0.1%	0.5%
Orces CH	750	1158	0.65	[0.62; 0.68]	0.2%	0.5%
Black LJ	245	331	0.74	[0.69; 0.79]	0.1%	0.5%
Black LJ	347	490	0.71	[0.67; 0.75]	0.1%	0.5%
Black LJ	540	730	0.74	[0.71; 0.77]	0.1%	0.5%
Black LJ	450	648	0.69	[0.66; 0.73]	0.1%	0.5%
Black LJ	440	652	0.67	[0.64; 0.71]	0.1%	0.5%
Brinkmann K	107	108	0.99	[0.95; 1.00]	0.0%	0.4%
Cairncross CT	1183	1329	0.89	[0.87; 0.91]	0.2%	0.5%
Daly RM	673	810	0.83	[0.80; 0.86]	0.1%	0.5%
Daly RM	1235	1564	0.79	[0.77; 0.81]	0.3%	0.5%
Daly RM	1598	2011	0.79	[0.78; 0.81]	0.4%	0.5%
Daly RM	868	1393	0.62	[0.60; 0.65]	0.2%	0.5%
Daly RM	2202	2884	0.76	[0.75; 0.78]	0.5%	0.5%
Daly RM	1824	2556	0.71	[0.70; 0.73]	0.5%	0.5%
Eloi M	27576	39004	0.71	[0.70; 0.71]	6.9%	0.5%
Gill TK	165	263	0.63	[0.57; 0.69]	0.0%	0.5%
Gill TK	150	265	0.57	[0.50; 0.63]	0.0%	0.5%
Gill TK	245	390	0.63	[0.58; 0.68]	0.1%	0.5%
Gill TK	333	486	0.69	[0.64; 0.73]	0.1%	0.5%
Gill TK	346	510	0.68	[0.64; 0.72]	0.1%	0.5%
Gill TK	311	500	0.62	[0.58; 0.66]	0.1%	0.5%
Hirani V	133	174	0.76	[0.69; 0.83]	0.0%	0.4%
Hirani V	248	304	0.82	[0.77; 0.86]	0.1%	0.5%
Hirani V	421	522	0.81	[0.77; 0.84]	0.1%	0.5%
Hirani V	554	659	0.84	[0.81; 0.87]	0.1%	0.5%
Horton−French K	257	400	0.64	[0.59; 0.69]	0.1%	0.5%
Horton−French K	445	692	0.64	[0.61; 0.68]	0.1%	0.5%
Lopes JB	363	415	0.87	[0.84; 0.90]	0.1%	0.5%
Oliveri B	307	339	0.91	[0.87; 0.93]	0.1%	0.5%
Peters BS	84	136	0.62	[0.53; 0.70]	0.0%	0.4%
Robinson PJ	238	267	0.89	[0.85; 0.93]	0.0%	0.5%
Robinson PJ	562	640	0.88	[0.85; 0.90]	0.1%	0.5%
Santos BR	212	234	0.91	[0.86; 0.94]	0.0%	0.5%
Unger MD	473	603	0.78	[0.75; 0.82]	0.1%	0.5%
White Z	39	59	0.66	[0.53; 0.78]	0.0%	0.4%
**Common effect model**		**561978**	**0.80**	**[0.80; 0.80]**	**100.0%**	**−−**
**Random effects model**			**0.77**	**[0.74; 0.79]**	**−−**	**100.0%**

Heterogeneity: $I^2 = 100\%$, $\tau^2 = 0.0501$, $p = 0$

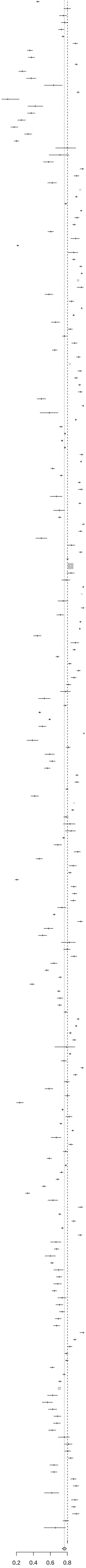

Supplementary figure 3 The global prevalence of serum 25(OH)D < 75 nmol/L

Appendix 5: The global prevalence of vitamin D deficiency from 2000–2010 to 2011–2022

Study	Events	Total	Proportion	95%−CI	Weight (common)	Weight (random)
Maguire JL	1	91	0.01	[0.00; 0.06]	0.0%	0.8%
Chin KY	2	150	0.01	[0.00; 0.05]	0.0%	0.8%
Bolland MJ	2	378	0.01	[0.00; 0.02]	0.0%	0.8%
Riverin B	3	52	0.06	[0.01; 0.16]	0.0%	0.8%
Science M	4	743	0.01	[0.00; 0.01]	0.0%	0.8%
Madsen KH	4	415	0.01	[0.00; 0.02]	0.0%	0.8%
Bjarnadottir A	5	158	0.03	[0.01; 0.07]	0.0%	0.8%
Zhu Z	6	183	0.03	[0.01; 0.07]	0.0%	0.8%
Meyer HE	7	196	0.04	[0.01; 0.07]	0.0%	0.8%
Mallah EM	8	300	0.03	[0.01; 0.05]	0.0%	0.8%
Greene−Finestone LS	8	312	0.03	[0.01; 0.05]	0.0%	0.8%
Rodríguez−Rodríguez E	8	102	0.08	[0.03; 0.15]	0.0%	0.8%
Bhatt SP	9	137	0.07	[0.03; 0.12]	0.0%	0.8%
Zhou SJ	9	221	0.04	[0.02; 0.08]	0.0%	0.8%
Lardner E	14	143	0.10	[0.05; 0.16]	0.0%	0.8%
Paul TV	15	150	0.10	[0.06; 0.16]	0.0%	0.8%
Mansbach JM	17	1799	0.01	[0.01; 0.02]	0.0%	0.8%
Andersen R	17	221	0.08	[0.05; 0.12]	0.0%	0.8%
Sioen I	18	357	0.05	[0.03; 0.08]	0.0%	0.8%
Greene−Finestone LS	18	777	0.02	[0.01; 0.04]	0.0%	0.8%
Shetty S	18	252	0.07	[0.04; 0.11]	0.0%	0.8%
Greene−Finestone LS	19	823	0.02	[0.01; 0.04]	0.0%	0.8%
Hatun S	19	89	0.21	[0.13; 0.31]	0.0%	0.8%
Smith N	19	148	0.13	[0.08; 0.19]	0.0%	0.8%
Gill TK	21	2413	0.01	[0.01; 0.01]	0.0%	0.8%
Nakamura K	22	600	0.04	[0.02; 0.05]	0.0%	0.8%
Granlund L	26	216	0.12	[0.08; 0.17]	0.0%	0.8%
Zhu Z	26	2269	0.01	[0.01; 0.02]	0.0%	0.8%
Hirani V	27	174	0.16	[0.10; 0.22]	0.0%	0.8%
Mansbach JM	27	2759	0.01	[0.01; 0.01]	0.0%	0.8%
Islam MZ	27	189	0.14	[0.10; 0.20]	0.0%	0.8%
Pérez−Llamas F	28	86	0.33	[0.23; 0.44]	0.0%	0.8%
Zhu Z	29	1440	0.02	[0.01; 0.03]	0.0%	0.8%
Gebreegziabher T	29	196	0.15	[0.10; 0.21]	0.0%	0.8%
Hirani V	30	304	0.10	[0.07; 0.14]	0.0%	0.8%
Andersen R	30	37	0.81	[0.65; 0.92]	0.0%	0.7%
Courraud J	31	177	0.18	[0.12; 0.24]	0.0%	0.8%
Islam MZ	31	244	0.13	[0.09; 0.18]	0.0%	0.8%
Saraiva GL	33	214	0.15	[0.11; 0.21]	0.0%	0.8%
Oliveri B	35	339	0.10	[0.07; 0.14]	0.0%	0.8%
Djennane M	35	435	0.08	[0.06; 0.11]	0.0%	0.8%
Robinson PJ	37	267	0.14	[0.10; 0.19]	0.0%	0.8%
Andersen R	37	199	0.19	[0.13; 0.25]	0.0%	0.8%
El Hayek J	39	282	0.14	[0.10; 0.18]	0.0%	0.8%
Arya V	44	92	0.48	[0.37; 0.58]	0.0%	0.8%
Hirani V	45	522	0.09	[0.06; 0.11]	0.0%	0.8%
Mitchell DM	45	634	0.07	[0.05; 0.09]	0.0%	0.8%
Daly RM	46	1393	0.03	[0.02; 0.04]	0.0%	0.8%
Orwoll E	47	1606	0.03	[0.02; 0.04]	0.0%	0.8%
Samefors M	48	333	0.14	[0.11; 0.19]	0.0%	0.8%
Al−Kindi MK	51	100	0.51	[0.41; 0.61]	0.0%	0.8%
Scalco R	53	98	0.54	[0.44; 0.64]	0.0%	0.8%
Riverin B	54	944	0.06	[0.04; 0.07]	0.0%	0.8%
Hirani V	57	659	0.09	[0.07; 0.11]	0.0%	0.8%
Bachhel R	60	150	0.40	[0.32; 0.48]	0.0%	0.8%
Skull SA	61	116	0.53	[0.43; 0.62]	0.0%	0.8%
Tseng M	66	194	0.34	[0.27; 0.41]	0.0%	0.8%
Daly RM	66	810	0.08	[0.06; 0.10]	0.0%	0.8%
Meddeb N	66	389	0.17	[0.13; 0.21]	0.0%	0.8%
Robinson PJ	74	640	0.12	[0.09; 0.14]	0.0%	0.8%
Karagüzel G	75	746	0.10	[0.08; 0.12]	0.0%	0.8%
Daly RM	76	1564	0.05	[0.04; 0.06]	0.0%	0.8%
Cashman KD	76	1132	0.07	[0.05; 0.08]	0.0%	0.8%
Daly RM	77	2011	0.04	[0.03; 0.05]	0.0%	0.8%
Aucoin M	78	756	0.10	[0.08; 0.13]	0.0%	0.8%
Abiaka C	80	206	0.39	[0.32; 0.46]	0.0%	0.8%
Lucas JA	92	1606	0.06	[0.05; 0.07]	0.0%	0.8%
Aucoin M	96	461	0.21	[0.17; 0.25]	0.0%	0.8%
von Hurst PR	98	228	0.43	[0.36; 0.50]	0.0%	0.8%
Le Goaziou MF	105	196	0.54	[0.46; 0.61]	0.0%	0.8%
Daly RM	108	2556	0.04	[0.03; 0.05]	0.0%	0.8%
Choi HR	110	651	0.17	[0.14; 0.20]	0.0%	0.8%
Daly RM	111	2884	0.04	[0.03; 0.05]	0.0%	0.8%
Niafar M	115	300	0.38	[0.33; 0.44]	0.0%	0.8%
Masoud MS	117	170	0.69	[0.61; 0.76]	0.0%	0.8%
Manios Y	122	2353	0.05	[0.04; 0.06]	0.0%	0.8%
Tolppanen AM	124	7560	0.02	[0.01; 0.02]	0.1%	0.8%
Dalgård C	124	669	0.19	[0.16; 0.22]	0.0%	0.8%
Matheï C	129	367	0.35	[0.30; 0.40]	0.0%	0.8%
Moreno−Reyes R	135	401	0.34	[0.29; 0.39]	0.0%	0.8%
Jorde R	157	2668	0.06	[0.05; 0.07]	0.0%	0.8%
Andersen R	158	219	0.72	[0.66; 0.78]	0.0%	0.8%
Alyahya K	169	232	0.73	[0.67; 0.78]	0.0%	0.8%
Nichols EK	180	915	0.20	[0.17; 0.22]	0.0%	0.8%
Raposo L	188	500	0.38	[0.33; 0.42]	0.0%	0.8%
Cougnard−Grégoire A	190	697	0.27	[0.24; 0.31]	0.0%	0.8%
Alkerwi A	207	1335	0.16	[0.14; 0.18]	0.0%	0.8%
Langlois K	218	5306	0.04	[0.04; 0.05]	0.1%	0.8%
Shivane VK	220	1137	0.19	[0.17; 0.22]	0.0%	0.8%
Uush T	222	524	0.42	[0.38; 0.47]	0.0%	0.8%
Hovsepian S	231	1111	0.21	[0.18; 0.23]	0.0%	0.8%
Voortman T	258	4167	0.06	[0.05; 0.07]	0.1%	0.8%
Ardawi MS	264	843	0.31	[0.28; 0.35]	0.0%	0.8%
Hirani V	292	2070	0.14	[0.13; 0.16]	0.0%	0.8%
Ganmaa D	333	420	0.79	[0.75; 0.83]	0.0%	0.8%
Zhang FF	348	960	0.36	[0.33; 0.39]	0.0%	0.8%
Wyskida M	355	3472	0.10	[0.09; 0.11]	0.0%	0.8%
Uush T	457	876	0.52	[0.49; 0.56]	0.0%	0.8%
Nielsen NO	525	2877	0.18	[0.17; 0.20]	0.0%	0.8%
Ardawi MS	534	1172	0.46	[0.43; 0.48]	0.0%	0.8%
Rabenberg M	540	1816	0.30	[0.28; 0.32]	0.0%	0.8%
Goswami R	559	642	0.87	[0.84; 0.90]	0.0%	0.8%
Schramm S	660	4149	0.16	[0.15; 0.17]	0.1%	0.8%
Al−Saleh Y	718	2226	0.32	[0.30; 0.34]	0.0%	0.8%
Laird E	728	5356	0.14	[0.13; 0.15]	0.1%	0.8%
Rabenberg M	756	2456	0.31	[0.29; 0.33]	0.0%	0.8%
Zgaga L	772	2235	0.35	[0.33; 0.37]	0.0%	0.8%
Rabenberg M	804	2723	0.30	[0.28; 0.31]	0.0%	0.8%
Hashemipour S	812	1210	0.67	[0.64; 0.70]	0.0%	0.8%
Sarafin K	839	11336	0.07	[0.07; 0.08]	0.2%	0.8%
Perna L	844	5386	0.16	[0.15; 0.17]	0.1%	0.8%
Thuesen B	848	6146	0.14	[0.13; 0.15]	0.1%	0.8%
Saliba W	891	6518	0.14	[0.13; 0.15]	0.1%	0.8%
Patel JV	1283	1904	0.67	[0.65; 0.69]	0.0%	0.8%
Hintzpeter B	1797	9001	0.20	[0.19; 0.21]	0.1%	0.8%
Saliba W	27676	192240	0.14	[0.14; 0.15]	2.7%	0.8%
Lin LY	60687	448601	0.14	[0.13; 0.14]	6.2%	0.8%
Crowe FL	223186	755174	0.30	[0.29; 0.30]	10.5%	0.8%
Crowe FL	299730	993895	0.30	[0.30; 0.30]	13.8%	0.8%
Crowe FL	300265	901352	0.33	[0.33; 0.33]	12.5%	0.8%
Crowe FL	443448	1180086	0.38	[0.37; 0.38]	16.4%	0.8%
Crowe FL	531681	1316390	0.40	[0.40; 0.40]	18.3%	0.8%
Crowe FL	536113	1269812	0.42	[0.42; 0.42]	17.6%	0.8%
**Common effect model**		**7208689**	**0.33**	**[0.33; 0.33]**	**100.0%**	**−−**
**Random effects model**			**0.18**	**[0.14; 0.21]**	**−−**	**100.0%**

Heterogeneity: I^2 = 100%, τ^2 = 0.0621, p = 0

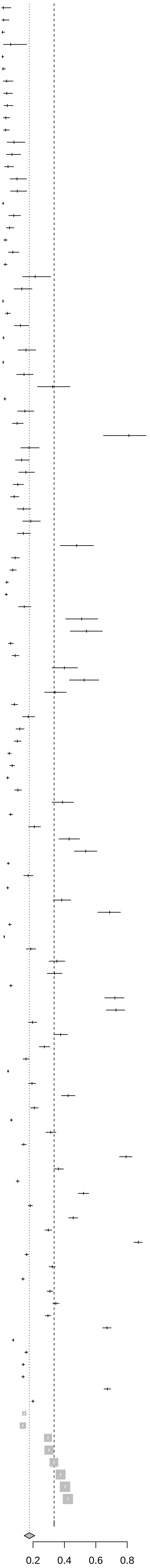

Supplementary figure 4 The global prevalence of serum 25(OH)D < 30 nmol/L in 2000–2010

Appendix 5: The global prevalence of vitamin D deficiency from 2000–2010 to 2011–2022

Study	Events	Total	Proportion	95%−CI	Weight (common)	Weight (random)
Lin L	1	353	0.00	[0.00; 0.02]	0.1%	0.7%
Lin L	1	477	0.00	[0.00; 0.01]	0.1%	0.7%
Lin L	1	860	0.00	[0.00; 0.01]	0.2%	0.7%
Lategan R	1	339	0.00	[0.00; 0.02]	0.1%	0.7%
Maldonado G	3	269	0.01	[0.00; 0.03]	0.1%	0.7%
Ginter JK	3	224	0.01	[0.00; 0.04]	0.1%	0.7%
El Hayek J	3	508	0.01	[0.00; 0.02]	0.1%	0.7%
Glatt DU	3	49	0.06	[0.01; 0.17]	0.0%	0.7%
Mechenro J	4	48	0.08	[0.02; 0.20]	0.0%	0.7%
Yousef S	5	267	0.02	[0.01; 0.04]	0.1%	0.7%
Smith G	7	128	0.05	[0.02; 0.11]	0.0%	0.7%
Herrick KA	7	1438	0.00	[0.00; 0.01]	0.3%	0.7%
Andersen R	8	52	0.15	[0.07; 0.28]	0.0%	0.7%
Tran B	9	134	0.07	[0.03; 0.12]	0.0%	0.7%
Nälsén C	9	268	0.03	[0.02; 0.06]	0.1%	0.7%
Fernández Bustillo JM	9	153	0.06	[0.03; 0.11]	0.0%	0.7%
Nälsén C	10	206	0.05	[0.02; 0.09]	0.0%	0.7%
Tran B	11	148	0.07	[0.04; 0.13]	0.0%	0.7%
Tran B	11	148	0.07	[0.04; 0.13]	0.0%	0.7%
Ní Chaoimh C	12	741	0.02	[0.01; 0.03]	0.2%	0.7%
NHANES(2017...2018)	12	1321	0.01	[0.00; 0.02]	0.3%	0.7%
Abdulrahman MA	13	63	0.21	[0.11; 0.33]	0.0%	0.7%
Smith G	13	495	0.03	[0.01; 0.04]	0.1%	0.7%
Andersen R	13	54	0.24	[0.13; 0.38]	0.0%	0.7%
Chao YS	13	1493	0.01	[0.00; 0.01]	0.4%	0.7%
Tran B	15	131	0.11	[0.07; 0.18]	0.0%	0.7%
Abdulrahman MA	16	50	0.32	[0.20; 0.47]	0.0%	0.7%
Black LJ	16	331	0.05	[0.03; 0.08]	0.1%	0.7%
Jääskeläinen T	17	4051	0.00	[0.00; 0.01]	1.0%	0.7%
Tran B	20	92	0.22	[0.14; 0.32]	0.0%	0.7%
Black LJ	23	490	0.05	[0.03; 0.07]	0.1%	0.7%
NHANES(2015...2016)	23	1231	0.02	[0.01; 0.03]	0.3%	0.7%
Rabufetti A	24	1045	0.02	[0.01; 0.03]	0.2%	0.7%
Black LJ	26	730	0.04	[0.02; 0.05]	0.2%	0.7%
NHANES(2015...2016)	26	2618	0.01	[0.01; 0.01]	0.6%	0.7%
Sharawat IK	29	100	0.29	[0.20; 0.39]	0.0%	0.7%
Herrick KA	29	2060	0.01	[0.01; 0.02]	0.5%	0.7%
Black LJ	30	648	0.05	[0.03; 0.07]	0.2%	0.7%
Hribar M	31	125	0.25	[0.18; 0.33]	0.0%	0.7%
Karin Z	31	260	0.12	[0.08; 0.16]	0.1%	0.7%
Petrenya N	31	4465	0.01	[0.00; 0.01]	1.0%	0.7%
Al Hayek S	33	344	0.10	[0.07; 0.13]	0.1%	0.7%
Hribar M	36	155	0.23	[0.17; 0.31]	0.0%	0.7%
NHANES(2017...2018)	37	2184	0.02	[0.01; 0.02]	0.5%	0.7%
NHANES (2015...2016)	37	1777	0.02	[0.01; 0.03]	0.4%	0.7%
Lima−Costa MF	37	2264	0.02	[0.01; 0.02]	0.5%	0.7%
Yousef S	38	932	0.04	[0.03; 0.06]	0.2%	0.7%
Man PW	40	416	0.10	[0.07; 0.13]	0.1%	0.7%
Asakura K	40	107	0.37	[0.28; 0.47]	0.0%	0.7%
Mechenro J	41	101	0.41	[0.31; 0.51]	0.0%	0.7%
Black LJ	43	652	0.07	[0.05; 0.09]	0.2%	0.7%
NHANES(2017...2018)	43	1810	0.02	[0.02; 0.03]	0.4%	0.7%
Mechenro J	46	275	0.17	[0.13; 0.22]	0.1%	0.7%
Saeed BQ	48	287	0.17	[0.13; 0.22]	0.1%	0.7%
Abdulrahman MA	50	278	0.18	[0.14; 0.23]	0.1%	0.7%
Jolliffe DA	55	222	0.25	[0.19; 0.31]	0.1%	0.7%
Souberbielle JC	56	892	0.06	[0.05; 0.08]	0.2%	0.7%
Hoge A	58	915	0.06	[0.05; 0.08]	0.2%	0.7%
Al Shaikh AM	61	204	0.30	[0.24; 0.37]	0.0%	0.7%
Saki F	63	477	0.13	[0.10; 0.17]	0.1%	0.7%
Kunz C	63	327	0.19	[0.15; 0.24]	0.1%	0.7%
Yousef S	63	1361	0.05	[0.04; 0.06]	0.3%	0.7%
Brinkmann K	67	108	0.62	[0.52; 0.71]	0.0%	0.7%
Hansen L	79	527	0.15	[0.12; 0.18]	0.1%	0.7%
NHANES(2017...2018)	80	2094	0.04	[0.03; 0.05]	0.5%	0.7%
Yan X	81	302	0.27	[0.22; 0.32]	0.1%	0.7%
NHANES(2015...2016)	85	2413	0.04	[0.03; 0.04]	0.6%	0.7%
Cairncross CT	86	1329	0.06	[0.05; 0.08]	0.3%	0.7%
Alyahya KO	88	104	0.85	[0.76; 0.91]	0.0%	0.7%
Yousef S	90	858	0.10	[0.09; 0.13]	0.2%	0.7%
Ikonen H	92	3650	0.03	[0.02; 0.03]	0.9%	0.7%
Bezrati I	92	225	0.41	[0.34; 0.48]	0.1%	0.7%
Drali O	93	621	0.15	[0.12; 0.18]	0.1%	0.7%
Sokolovic S	93	450	0.21	[0.17; 0.25]	0.1%	0.7%
Herrick KA	94	3267	0.03	[0.02; 0.04]	0.8%	0.7%
Sochorová L	101	419	0.24	[0.20; 0.28]	0.1%	0.7%
Kunz C	106	528	0.20	[0.17; 0.24]	0.1%	0.7%
Kunz C	111	456	0.24	[0.20; 0.29]	0.1%	0.7%
Kunz C	112	520	0.22	[0.18; 0.25]	0.1%	0.7%
Herrick KA	113	2355	0.05	[0.04; 0.06]	0.6%	0.7%
Nakhaee S	115	400	0.29	[0.24; 0.33]	0.1%	0.7%
Yu S	128	2173	0.06	[0.05; 0.07]	0.5%	0.7%
Beer RJ	129	6813	0.02	[0.02; 0.02]	1.6%	0.7%
Tønnesen R	135	700	0.19	[0.16; 0.22]	0.2%	0.7%
Capuano R	137	1200	0.11	[0.10; 0.13]	0.3%	0.7%
Fang F	139	1814	0.08	[0.06; 0.09]	0.4%	0.7%
Chlebna−Sokó.. D	145	720	0.20	[0.17; 0.23]	0.2%	0.7%
Oberg J	147	890	0.17	[0.14; 0.19]	0.2%	0.7%
Öztürk ZA	150	1161	0.13	[0.11; 0.15]	0.3%	0.7%
Al−Daghri NM	150	561	0.27	[0.23; 0.31]	0.1%	0.7%
Hutchings N	157	1206	0.13	[0.11; 0.15]	0.3%	0.7%
Solis−Urra P	181	686	0.26	[0.23; 0.30]	0.2%	0.7%
Arabi A	182	466	0.39	[0.35; 0.44]	0.1%	0.7%
Sokolovic S	185	645	0.29	[0.25; 0.32]	0.2%	0.7%
Beer RJ	194	6470	0.03	[0.03; 0.03]	1.5%	0.7%
Herrick KA	199	3496	0.06	[0.05; 0.07]	0.8%	0.7%
Solis−Urra P	204	1245	0.16	[0.14; 0.19]	0.3%	0.7%
Ceccarelli M	209	2140	0.10	[0.09; 0.11]	0.5%	0.7%
Shchubelka K	211	1639	0.13	[0.11; 0.15]	0.4%	0.7%
Arnljots R	224	545	0.41	[0.37; 0.45]	0.1%	0.7%
Sokolovic S	235	735	0.32	[0.29; 0.35]	0.2%	0.7%
Al−Daghri NM	235	830	0.28	[0.25; 0.32]	0.2%	0.7%
Golbahar J	247	500	0.49	[0.45; 0.54]	0.1%	0.7%
Chirita−Emandi A	265	6631	0.04	[0.04; 0.04]	1.6%	0.7%
Beer RJ	265	7170	0.04	[0.03; 0.04]	1.7%	0.7%
Herrick KA	271	3564	0.08	[0.07; 0.09]	0.8%	0.7%
Qorbani M	276	2596	0.11	[0.09; 0.12]	0.6%	0.7%
Al−Daghri NM	276	808	0.34	[0.31; 0.38]	0.2%	0.7%
Hansen L	309	2565	0.12	[0.11; 0.13]	0.6%	0.7%
Jayatissa R	333	2525	0.13	[0.12; 0.15]	0.6%	0.7%
Gromova O	371	1347	0.28	[0.25; 0.30]	0.3%	0.7%
Nikooyeh B	374	667	0.56	[0.52; 0.60]	0.2%	0.7%
Beer RJ	395	16454	0.02	[0.02; 0.03]	3.9%	0.7%
Borissova AM	430	2016	0.21	[0.20; 0.23]	0.5%	0.7%
Al−Taiar A	559	1416	0.39	[0.37; 0.42]	0.3%	0.7%
Santos A	594	1500	0.40	[0.37; 0.42]	0.4%	0.7%
Sulimani RA	645	1618	0.40	[0.37; 0.42]	0.4%	0.7%
Duarte C	706	3092	0.23	[0.21; 0.24]	0.7%	0.7%
Al−Daghri NM	718	2225	0.32	[0.30; 0.34]	0.5%	0.7%
AlQuaiz AM	729	2832	0.26	[0.24; 0.27]	0.7%	0.7%
Chen J	731	6014	0.12	[0.11; 0.13]	1.4%	0.7%
Al Shaikh AM	899	1906	0.47	[0.45; 0.49]	0.4%	0.7%
Kaddam IM	912	2104	0.43	[0.41; 0.45]	0.5%	0.7%
Nikooyeh B	974	1406	0.69	[0.67; 0.72]	0.3%	0.7%
Yousef S	988	8161	0.12	[0.11; 0.13]	1.9%	0.7%
Li H	1020	4989	0.20	[0.19; 0.22]	1.2%	0.7%
Li H	1020	5707	0.18	[0.17; 0.19]	1.3%	0.7%
Al−Daghri NM	1082	4247	0.25	[0.24; 0.27]	1.0%	0.7%
Al−Daghri NM	1083	3111	0.35	[0.33; 0.37]	0.7%	0.7%
Chakrabarty S	1166	5024	0.23	[0.22; 0.24]	1.2%	0.7%
Joukar F	1189	5096	0.23	[0.22; 0.25]	1.2%	0.7%
Jiang W	1197	14302	0.08	[0.08; 0.09]	3.4%	0.7%
Aspell N	1423	6004	0.24	[0.23; 0.25]	1.4%	0.7%
Chakrabarty S	1608	6798	0.24	[0.23; 0.25]	1.6%	0.7%
Al Shaikh A	1687	3613	0.47	[0.45; 0.48]	0.8%	0.7%
Kaddam IM	1979	4035	0.49	[0.47; 0.51]	0.9%	0.7%
Griffin TP	2047	15319	0.13	[0.13; 0.14]	3.6%	0.7%
Bater J	5692	9595	0.59	[0.58; 0.60]	2.3%	0.7%
Kim SY	10652	157211	0.07	[0.07; 0.07]	36.9%	0.7%
**Common effect model**		**425572**	**0.10**	**[0.10; 0.10]**	**100.0%**	**−−**
**Random effects model**			**0.14**	**[0.12; 0.17]**	**−−**	**100.0%**

Heterogeneity: $I^2 = 100\%$, $\tau^2 = 0.0476$, $p = 0$

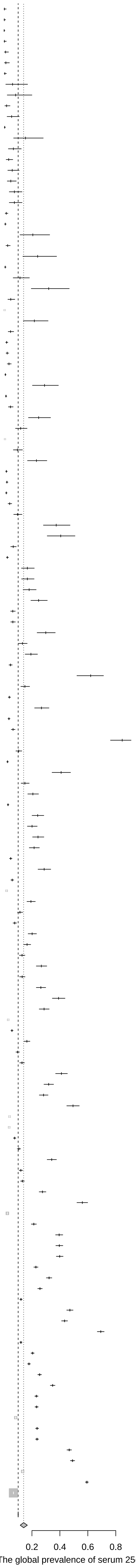

Supplementary figure 5 The global prevalence of serum 25(OH)D < 30 nmol/L in 2011–2022

Appendix 5: The global prevalence of vitamin D deficiency from 2000–2010 to 2011–2022

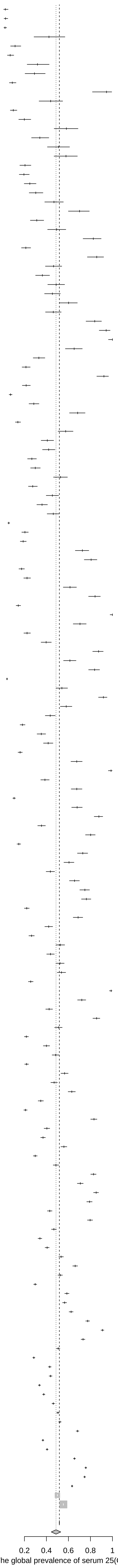

Study	Events	Total	Proportion	95%−CI	Weight (common)	Weight (random)
Mutua AM	8	302	0.03	[0.01; 0.05]	0.0%	0.6%
Miljkovic I	12	424	0.03	[0.01; 0.05]	0.0%	0.6%
Ho−Pham LT	15	637	0.02	[0.01; 0.04]	0.1%	0.6%
Riverin B	22	52	0.42	[0.29; 0.57]	0.0%	0.6%
Shady MM	23	200	0.12	[0.07; 0.17]	0.0%	0.6%
Madsen KH	24	340	0.07	[0.05; 0.10]	0.0%	0.6%
Maguire JL	29	91	0.32	[0.22; 0.42]	0.0%	0.6%
Fayet−Moore F	30	103	0.29	[0.21; 0.39]	0.0%	0.6%
Bolland MJ	34	378	0.09	[0.06; 0.12]	0.0%	0.6%
Andersen R	35	37	0.95	[0.82; 0.99]	0.0%	0.6%
Hatun S	39	89	0.44	[0.33; 0.55]	0.0%	0.6%
Madsen KH	41	415	0.10	[0.07; 0.13]	0.0%	0.6%
Zhou SJ	44	221	0.20	[0.15; 0.26]	0.0%	0.6%
Pérez−Llamas F	50	86	0.58	[0.47; 0.69]	0.0%	0.6%
Chin KY	51	150	0.34	[0.26; 0.42]	0.0%	0.6%
Rodríguez−Rodríguez E	52	102	0.51	[0.41; 0.61]	0.0%	0.6%
González G	52	90	0.58	[0.47; 0.68]	0.0%	0.6%
Gill TK	54	263	0.21	[0.16; 0.26]	0.0%	0.6%
Greene−Finestone LS	61	312	0.20	[0.15; 0.24]	0.0%	0.6%
Gill TK	66	265	0.25	[0.20; 0.31]	0.0%	0.6%
Andersen R	67	221	0.30	[0.24; 0.37]	0.0%	0.6%
Lardner E	67	143	0.47	[0.38; 0.55]	0.0%	0.6%
Andersen S	68	97	0.70	[0.60; 0.79]	0.0%	0.6%
Ramakrishnan S	74	237	0.31	[0.25; 0.38]	0.0%	0.6%
Paul TV	74	150	0.49	[0.41; 0.58]	0.0%	0.6%
Zargar AH	76	92	0.83	[0.73; 0.90]	0.0%	0.6%
Gill TK	83	390	0.21	[0.17; 0.26]	0.0%	0.6%
Scalco R	84	98	0.86	[0.77; 0.92]	0.0%	0.6%
Zhu Z	85	183	0.46	[0.39; 0.54]	0.0%	0.6%
Santos BR	85	234	0.36	[0.30; 0.43]	0.0%	0.6%
Hirani V	85	174	0.49	[0.41; 0.57]	0.0%	0.6%
Meyer HE	89	196	0.45	[0.38; 0.53]	0.0%	0.6%
Smith N	89	148	0.60	[0.52; 0.68]	0.0%	0.6%
Andersen R	92	199	0.46	[0.39; 0.53]	0.0%	0.6%
Cinar N	99	118	0.84	[0.76; 0.90]	0.0%	0.6%
Vupputuri MR	99	105	0.94	[0.88; 0.98]	0.0%	0.6%
Al−Kindi MK	100	100	1.00	[0.96; 1.00]	0.0%	0.6%
Bjarnadottir A	103	158	0.65	[0.57; 0.73]	0.0%	0.6%
Johnson MA	105	317	0.33	[0.28; 0.39]	0.0%	0.6%
Gill TK	107	500	0.21	[0.18; 0.25]	0.1%	0.6%
Skull SA	107	116	0.92	[0.86; 0.96]	0.0%	0.6%
Gill TK	110	510	0.22	[0.18; 0.25]	0.1%	0.6%
Mogire RM	111	1507	0.07	[0.06; 0.09]	0.2%	0.6%
Orces CH	115	403	0.29	[0.24; 0.33]	0.0%	0.6%
Courraud J	121	177	0.68	[0.61; 0.75]	0.0%	0.6%
Meyer HE	122	869	0.14	[0.12; 0.17]	0.1%	0.6%
Saraiva GL	123	214	0.57	[0.51; 0.64]	0.0%	0.6%
Hirani V	124	304	0.41	[0.35; 0.47]	0.0%	0.6%
Gordon CM	129	307	0.42	[0.36; 0.48]	0.0%	0.6%
Gill TK	130	486	0.27	[0.23; 0.31]	0.1%	0.6%
Djennane M	130	435	0.30	[0.26; 0.34]	0.0%	0.6%
Shetty S	133	252	0.53	[0.46; 0.59]	0.0%	0.6%
Vallianou N	135	490	0.28	[0.24; 0.32]	0.1%	0.6%
Muhairi SJ	143	315	0.45	[0.40; 0.51]	0.0%	0.6%
Janssen HC	144	400	0.36	[0.31; 0.41]	0.0%	0.6%
Moussavi M	147	318	0.46	[0.41; 0.52]	0.0%	0.6%
Chailurkit LO	151	2641	0.06	[0.05; 0.07]	0.3%	0.6%
Science M	152	743	0.20	[0.18; 0.24]	0.1%	0.6%
Greene−Finestone LS	155	823	0.19	[0.16; 0.22]	0.1%	0.6%
Granlund L	157	216	0.73	[0.66; 0.79]	0.0%	0.6%
Le Goaziou MF	158	196	0.81	[0.74; 0.86]	0.0%	0.6%
Harinarayan CV	159	913	0.17	[0.15; 0.20]	0.1%	0.6%
Orces CH	162	724	0.22	[0.19; 0.26]	0.1%	0.6%
Robinson PJ	164	267	0.61	[0.55; 0.67]	0.0%	0.6%
Gebreegziabher T	165	196	0.84	[0.78; 0.89]	0.0%	0.6%
Lappe JM	170	1179	0.14	[0.12; 0.17]	0.1%	0.6%
Masoud MS	170	170	1.00	[0.98; 1.00]	0.0%	0.6%
Islam MZ	172	244	0.70	[0.64; 0.76]	0.0%	0.6%
Greene−Finestone LS	174	777	0.22	[0.20; 0.25]	0.1%	0.6%
Santos BR	176	443	0.40	[0.35; 0.44]	0.0%	0.6%
Abiaka C	180	206	0.87	[0.82; 0.92]	0.0%	0.6%
Niafar M	184	300	0.61	[0.56; 0.67]	0.0%	0.6%
von Hurst PR	191	228	0.84	[0.78; 0.88]	0.0%	0.6%
Batieha A	192	4590	0.04	[0.04; 0.05]	0.5%	0.6%
Harkness LS	200	370	0.54	[0.49; 0.59]	0.0%	0.6%
Andersen R	201	219	0.92	[0.87; 0.95]	0.0%	0.6%
Sioen I	207	357	0.58	[0.53; 0.63]	0.0%	0.6%
Gilbert−Diamond D	208	479	0.43	[0.39; 0.48]	0.1%	0.6%
Orces CH	211	1158	0.18	[0.16; 0.21]	0.1%	0.6%
Nakamura K	212	600	0.35	[0.32; 0.39]	0.1%	0.6%
Hirani V	217	522	0.42	[0.37; 0.46]	0.1%	0.6%
Klenk J	228	1418	0.16	[0.14; 0.18]	0.2%	0.6%
Oliveri B	229	339	0.68	[0.62; 0.73]	0.0%	0.6%
Alyahya K	229	232	0.99	[0.96; 1.00]	0.0%	0.6%
Mitchell DM	245	634	0.39	[0.35; 0.43]	0.1%	0.6%
Matheï C	248	367	0.68	[0.63; 0.72]	0.0%	0.6%
Gill TK	256	2413	0.11	[0.09; 0.12]	0.3%	0.6%
Moy FM	258	380	0.68	[0.63; 0.73]	0.0%	0.6%
Mallah EM	263	300	0.88	[0.83; 0.91]	0.0%	0.6%
Karagüzel G	265	746	0.36	[0.32; 0.39]	0.1%	0.6%
Samefors M	267	333	0.80	[0.75; 0.84]	0.0%	0.6%
Mansbach JM	268	1799	0.15	[0.13; 0.17]	0.2%	0.6%
Kull M Jr	268	367	0.73	[0.68; 0.77]	0.0%	0.6%
Aucoin M	279	461	0.61	[0.56; 0.65]	0.1%	0.6%
Hirani V	287	659	0.44	[0.40; 0.47]	0.1%	0.6%
Majumdar V	289	441	0.66	[0.61; 0.70]	0.0%	0.6%
Hekimsoy Z	293	391	0.75	[0.70; 0.79]	0.0%	0.6%
Moreno−Reyes R	306	401	0.76	[0.72; 0.80]	0.0%	0.6%
Daly RM	308	1393	0.22	[0.20; 0.24]	0.2%	0.6%
Bener A	315	458	0.69	[0.64; 0.73]	0.1%	0.6%
Aucoin M	318	756	0.42	[0.39; 0.46]	0.1%	0.6%
González−Molero I	334	1262	0.26	[0.24; 0.29]	0.1%	0.6%
Robinson PJ	337	640	0.53	[0.49; 0.57]	0.1%	0.6%
Daly RM	354	810	0.44	[0.40; 0.47]	0.1%	0.6%
Feng X	359	686	0.52	[0.49; 0.56]	0.1%	0.6%
Dalgård C	359	669	0.54	[0.50; 0.57]	0.1%	0.6%
Orwoll E	413	1606	0.26	[0.24; 0.28]	0.2%	0.6%
Ganmaa D	415	420	0.99	[0.97; 1.00]	0.0%	0.6%
Li S	417	578	0.72	[0.68; 0.76]	0.1%	0.6%
González−Gross M	427	1006	0.42	[0.39; 0.46]	0.1%	0.6%
Raposo L	428	500	0.86	[0.82; 0.89]	0.1%	0.6%
Ardawi MS	429	843	0.51	[0.47; 0.54]	0.1%	0.6%
Nimitphong H	433	1990	0.22	[0.20; 0.24]	0.2%	0.6%
Cashman KD	453	1132	0.40	[0.37; 0.43]	0.1%	0.6%
Riverin B	457	944	0.48	[0.45; 0.52]	0.1%	0.6%
Zhu Z	498	2269	0.22	[0.20; 0.24]	0.3%	0.6%
Nichols EK	517	915	0.57	[0.53; 0.60]	0.1%	0.6%
Hovsepian S	522	1111	0.47	[0.44; 0.50]	0.1%	0.6%
Mehboobali N	541	858	0.63	[0.60; 0.66]	0.1%	0.6%
Daly RM	545	1564	0.35	[0.32; 0.37]	0.2%	0.6%
Mansbach JM	578	2759	0.21	[0.19; 0.23]	0.3%	0.6%
Cougnard−Grégoire A	580	697	0.83	[0.80; 0.86]	0.1%	0.6%
Zhu Z	582	1440	0.40	[0.38; 0.43]	0.2%	0.6%
Daly RM	742	2011	0.37	[0.35; 0.39]	0.2%	0.6%
Alkerwi A	746	1335	0.56	[0.53; 0.59]	0.1%	0.6%
Daly RM	762	2556	0.30	[0.28; 0.32]	0.3%	0.6%
Lucas JA	781	1606	0.49	[0.46; 0.51]	0.2%	0.6%
Zhang FF	795	960	0.83	[0.80; 0.85]	0.1%	0.6%
Shivane VK	805	1137	0.71	[0.68; 0.73]	0.1%	0.6%
Kaykhaei MA	846	993	0.85	[0.83; 0.87]	0.1%	0.6%
Seo JA	856	1081	0.79	[0.77; 0.82]	0.1%	0.6%
Penrose K	929	2160	0.43	[0.41; 0.45]	0.2%	0.6%
Ardawi MS	934	1172	0.80	[0.77; 0.82]	0.1%	0.6%
Hirani V	968	2070	0.47	[0.45; 0.49]	0.2%	0.6%
Daly RM	983	2884	0.34	[0.32; 0.36]	0.3%	0.6%
Jorde R	1084	2668	0.41	[0.39; 0.43]	0.3%	0.6%
Al−Saleh Y	1187	2226	0.53	[0.51; 0.55]	0.2%	0.6%
Rabenberg M	1201	1816	0.66	[0.64; 0.68]	0.2%	0.6%
Manios Y	1235	2353	0.52	[0.50; 0.55]	0.3%	0.6%
Voortman T	1241	4167	0.30	[0.28; 0.31]	0.5%	0.6%
Rabenberg M	1439	2456	0.59	[0.57; 0.61]	0.3%	0.6%
Nielsen NO	1626	2877	0.57	[0.55; 0.58]	0.3%	0.6%
Rabenberg M	1700	2723	0.62	[0.61; 0.64]	0.3%	0.6%
Zgaga L	1732	2235	0.77	[0.76; 0.79]	0.3%	0.6%
Patel JV	1732	1904	0.91	[0.90; 0.92]	0.2%	0.6%
Byun EJ	1843	2515	0.73	[0.72; 0.75]	0.3%	0.6%
Schramm S	2104	4149	0.51	[0.49; 0.52]	0.5%	0.6%
Tolppanen AM	2158	7560	0.29	[0.28; 0.30]	0.8%	0.6%
Laird E	2303	5356	0.43	[0.42; 0.44]	0.6%	0.6%
Leung RY	2309	5276	0.44	[0.42; 0.45]	0.6%	0.6%
Liu X	2753	8182	0.34	[0.33; 0.35]	0.9%	0.6%
Liu X	2811	7485	0.38	[0.36; 0.39]	0.8%	0.6%
Saliba W	3014	6518	0.46	[0.45; 0.47]	0.7%	0.6%
Ward M	3107	6154	0.50	[0.49; 0.52]	0.7%	0.6%
Thuesen B	3208	6146	0.52	[0.51; 0.53]	0.7%	0.6%
Perna L	3677	5386	0.68	[0.67; 0.70]	0.6%	0.6%
Sarafin K	4172	11336	0.37	[0.36; 0.38]	1.3%	0.6%
Liu X	4200	10343	0.41	[0.40; 0.42]	1.2%	0.6%
Hintzpeter B	5895	9001	0.65	[0.65; 0.66]	1.0%	0.6%
Song HR	6802	8976	0.76	[0.75; 0.77]	1.0%	0.6%
Zhen D	7544	10100	0.75	[0.74; 0.76]	1.1%	0.6%
Chung JY	11587	18305	0.63	[0.63; 0.64]	2.1%	0.6%
Saliba W	96055	192240	0.50	[0.50; 0.50]	21.5%	0.6%
Lin LY	248478	448601	0.55	[0.55; 0.56]	50.3%	0.6%
**Common effect model**		**892087**	**0.52**	**[0.52; 0.52]**	**100.0%**	**−−**
**Random effects model**			**0.49**	**[0.44; 0.53]**	**−−**	**100.0%**

Heterogeneity: $I^2 = 100\%$, $\tau^2 = 0.0778$, $p = 0$

Supplementary figure 6 The global prevalence of serum 25(OH)D < 50 nmol/L in 2000–2010

Appendix 5: The global prevalence of vitamin D deficiency from 2000–2010 to 2011–2022

Study	Events	Total	Proportion	95%−CI	Weight (common)	Weight (random)
Houghton LA	2	433	0.00	[0.00; 0.02]	0.1%	0.5%
Smith G	3	41	0.07	[0.02; 0.20]	0.0%	0.5%
White Z	4	59	0.07	[0.02; 0.16]	0.0%	0.5%
Middelkoop K	4	168	0.02	[0.01; 0.06]	0.0%	0.5%
Lin L	6	353	0.02	[0.01; 0.04]	0.1%	0.5%
Middelkoop K	8	281	0.03	[0.01; 0.06]	0.0%	0.5%
Ten Haaf DSM	9	450	0.02	[0.01; 0.04]	0.1%	0.5%
Naqvi A	11	86	0.13	[0.07; 0.22]	0.0%	0.5%
Tangoh DA	12	372	0.03	[0.02; 0.06]	0.1%	0.5%
Lategan R	14	339	0.04	[0.02; 0.07]	0.1%	0.5%
Middelkoop K	18	346	0.05	[0.03; 0.08]	0.1%	0.5%
Lin L	20	477	0.04	[0.03; 0.06]	0.1%	0.5%
Lin L	22	860	0.03	[0.02; 0.04]	0.1%	0.5%
Mechenro J	22	48	0.46	[0.31; 0.61]	0.0%	0.5%
Middelkoop K	22	108	0.20	[0.13; 0.29]	0.0%	0.5%
Glatt DU	24	49	0.49	[0.34; 0.64]	0.0%	0.5%
Smith G	25	128	0.20	[0.13; 0.27]	0.0%	0.5%
Andersen R	25	52	0.48	[0.34; 0.62]	0.0%	0.5%
Kouda K	26	400	0.06	[0.04; 0.09]	0.1%	0.5%
Ginter JK	27	224	0.12	[0.08; 0.17]	0.0%	0.5%
Abdulrahman MA	30	50	0.60	[0.45; 0.74]	0.0%	0.5%
Abdulrahman MA	34	63	0.54	[0.41; 0.67]	0.0%	0.5%
Guo S	34	100	0.34	[0.25; 0.44]	0.0%	0.5%
Middelkoop K	35	503	0.07	[0.05; 0.10]	0.1%	0.5%
Pan T	38	194	0.20	[0.14; 0.26]	0.0%	0.5%
Yu L	40	1200	0.03	[0.02; 0.05]	0.2%	0.5%
Yousef S	42	267	0.16	[0.12; 0.21]	0.0%	0.5%
Kagotho E	44	253	0.17	[0.13; 0.23]	0.0%	0.5%
Maldonado G	47	269	0.17	[0.13; 0.23]	0.0%	0.5%
Andersen R	47	54	0.87	[0.75; 0.95]	0.0%	0.5%
Bi X	48	114	0.42	[0.33; 0.52]	0.0%	0.5%
Middelkoop K	48	396	0.12	[0.09; 0.16]	0.1%	0.5%
Srimani S	49	222	0.22	[0.17; 0.28]	0.0%	0.5%
Naeem Z	53	180	0.29	[0.23; 0.37]	0.0%	0.5%
El Hayek J	54	508	0.11	[0.08; 0.14]	0.1%	0.5%
Hazell TJ	57	488	0.12	[0.09; 0.15]	0.1%	0.5%
Nälsén C	60	268	0.22	[0.18; 0.28]	0.0%	0.5%
Sharawat IK	63	100	0.63	[0.53; 0.72]	0.0%	0.5%
Smith G	64	495	0.13	[0.10; 0.16]	0.1%	0.5%
Hribar M	72	125	0.58	[0.48; 0.66]	0.0%	0.5%
Wakayo T	73	174	0.42	[0.35; 0.50]	0.0%	0.5%
Cabral MA	74	234	0.32	[0.26; 0.38]	0.0%	0.5%
Liu X	76	207	0.37	[0.30; 0.44]	0.0%	0.5%
Tran B	76	92	0.83	[0.73; 0.90]	0.0%	0.5%
Bodin J	85	95	0.89	[0.81; 0.95]	0.0%	0.5%
Nälsén C	87	206	0.42	[0.35; 0.49]	0.0%	0.5%
Yu L	87	1200	0.07	[0.06; 0.09]	0.2%	0.5%
Asakura K	88	107	0.82	[0.74; 0.89]	0.0%	0.5%
Hussain T	89	151	0.59	[0.51; 0.67]	0.0%	0.5%
Tran B	92	134	0.69	[0.60; 0.76]	0.0%	0.5%
Mechenro J	92	101	0.91	[0.84; 0.96]	0.0%	0.5%
Bettencourt A	95	198	0.48	[0.41; 0.55]	0.0%	0.5%
Black LJ	96	331	0.29	[0.24; 0.34]	0.1%	0.5%
Alyahya KO	96	104	0.92	[0.85; 0.97]	0.0%	0.5%
Hribar M	98	155	0.63	[0.55; 0.71]	0.0%	0.5%
AlFaris NA	100	166	0.60	[0.52; 0.68]	0.0%	0.5%
Fernández Bustillo JM	101	153	0.66	[0.58; 0.73]	0.0%	0.5%
Feketea GM	102	376	0.27	[0.23; 0.32]	0.1%	0.5%
Tran B	102	131	0.78	[0.70; 0.85]	0.0%	0.5%
Herrick KA	102	1438	0.07	[0.06; 0.09]	0.2%	0.5%
Brinkmann K	104	108	0.96	[0.91; 0.99]	0.0%	0.5%
Tran B	106	148	0.72	[0.64; 0.79]	0.0%	0.5%
Tran B	107	148	0.72	[0.64; 0.79]	0.0%	0.5%
Al Hayek S	108	344	0.31	[0.27; 0.37]	0.1%	0.5%
Abdulrahman MA	112	278	0.40	[0.34; 0.46]	0.0%	0.5%
Horton−French K	112	400	0.28	[0.24; 0.33]	0.1%	0.5%
Horton−French K	112	692	0.16	[0.14; 0.19]	0.1%	0.5%
Benjeddou K	114	239	0.48	[0.41; 0.54]	0.0%	0.5%
Contreras−Manzano A	120	381	0.31	[0.27; 0.36]	0.1%	0.5%
Contreras−Manzano A	127	404	0.31	[0.27; 0.36]	0.1%	0.5%
NHANES(2017...2018)	127	1321	0.10	[0.08; 0.11]	0.2%	0.5%
Chao YS	129	1493	0.09	[0.07; 0.10]	0.3%	0.5%
Koyama S	138	492	0.28	[0.24; 0.32]	0.1%	0.5%
Jolliffe DA	144	222	0.65	[0.58; 0.71]	0.0%	0.5%
Black LJ	147	490	0.30	[0.26; 0.34]	0.1%	0.5%
Contreras−Manzano A	150	477	0.31	[0.27; 0.36]	0.1%	0.5%
Karin Z	151	260	0.58	[0.52; 0.64]	0.0%	0.5%
Sherchand O	154	300	0.51	[0.46; 0.57]	0.1%	0.5%
Junaid K	156	215	0.73	[0.66; 0.78]	0.0%	0.5%
Suryanarayana P	161	298	0.54	[0.48; 0.60]	0.1%	0.5%
Mechenro J	162	275	0.59	[0.53; 0.65]	0.0%	0.5%
Vasudevan B	164	184	0.89	[0.84; 0.93]	0.0%	0.5%
NHANES(2015...2016)	170	1231	0.14	[0.12; 0.16]	0.2%	0.5%
Black LJ	172	652	0.26	[0.23; 0.30]	0.1%	0.5%
Sheikh A	173	300	0.58	[0.52; 0.63]	0.1%	0.5%
Sokolovic S	179	450	0.40	[0.35; 0.44]	0.1%	0.5%
Rabufetti A	179	1045	0.17	[0.15; 0.20]	0.2%	0.5%
Al Shaikh AM	184	204	0.90	[0.85; 0.94]	0.0%	0.5%
Black LJ	187	648	0.29	[0.25; 0.33]	0.1%	0.5%
Black LJ	188	730	0.26	[0.23; 0.29]	0.1%	0.5%
Bezrati I	191	225	0.85	[0.80; 0.89]	0.0%	0.5%
Nadeem S	197	221	0.89	[0.84; 0.93]	0.0%	0.5%
Ní Chaoimh C	198	741	0.27	[0.24; 0.30]	0.1%	0.5%
Kunz C	205	327	0.63	[0.57; 0.68]	0.1%	0.5%
Rafraf M	207	216	0.96	[0.92; 0.98]	0.0%	0.5%
Flores ME	208	713	0.29	[0.26; 0.33]	0.1%	0.5%
Vierucci F	213	427	0.50	[0.45; 0.55]	0.1%	0.5%
Yan X	216	302	0.72	[0.66; 0.77]	0.1%	0.5%
Sakyi SA	218	500	0.44	[0.39; 0.48]	0.1%	0.5%
Yousef S	231	932	0.25	[0.22; 0.28]	0.2%	0.5%
Man PW	236	416	0.57	[0.52; 0.62]	0.1%	0.5%
Sochorová L	252	419	0.60	[0.55; 0.65]	0.1%	0.5%
Zhu W	254	508	0.50	[0.46; 0.54]	0.1%	0.5%
Alloubani A	266	350	0.76	[0.71; 0.80]	0.1%	0.5%
Khan AH	275	305	0.90	[0.86; 0.93]	0.1%	0.5%
Jääskeläinen T	281	4051	0.07	[0.06; 0.08]	0.7%	0.5%
Herrick KA	282	2060	0.14	[0.12; 0.15]	0.4%	0.5%
Kunz C	283	456	0.62	[0.57; 0.67]	0.1%	0.5%
Gariballa S	286	648	0.44	[0.40; 0.48]	0.1%	0.5%
Nakhaee S	296	400	0.74	[0.69; 0.78]	0.1%	0.5%
de Oliveira CL	298	1152	0.26	[0.23; 0.28]	0.2%	0.5%
Yousef S	305	1361	0.22	[0.20; 0.25]	0.2%	0.5%
Kapil U	307	374	0.82	[0.78; 0.86]	0.1%	0.5%
Souberbielle JC	309	892	0.35	[0.32; 0.38]	0.2%	0.5%
Kunz C	318	520	0.61	[0.57; 0.65]	0.1%	0.5%
Hansen L	321	527	0.61	[0.57; 0.65]	0.1%	0.5%
Kunz C	325	528	0.62	[0.57; 0.66]	0.1%	0.5%
NHANES (2015...2016)	332	1777	0.19	[0.17; 0.21]	0.3%	0.5%
Arabi A	335	466	0.72	[0.68; 0.76]	0.1%	0.5%
NHANES(2017...2018)	336	1810	0.19	[0.17; 0.20]	0.3%	0.5%
Yousef S	346	858	0.40	[0.37; 0.44]	0.1%	0.5%
Misra P	346	381	0.91	[0.87; 0.94]	0.1%	0.5%
Rahman A	352	410	0.86	[0.82; 0.89]	0.1%	0.5%
Sokolovic S	357	645	0.55	[0.51; 0.59]	0.1%	0.5%
Lima−Costa MF	362	2264	0.16	[0.15; 0.18]	0.4%	0.5%
Tønnesen R	373	700	0.53	[0.50; 0.57]	0.1%	0.5%
Drali O	375	621	0.60	[0.56; 0.64]	0.1%	0.5%
Saki F	388	477	0.81	[0.78; 0.85]	0.1%	0.5%
Hoge A	405	915	0.44	[0.41; 0.48]	0.2%	0.5%
Al−Daghri NM	408	561	0.73	[0.69; 0.76]	0.1%	0.5%
Rahmadhani R	410	1011	0.41	[0.38; 0.44]	0.2%	0.5%
NHANES(2017...2018)	415	2184	0.19	[0.17; 0.21]	0.4%	0.5%
Isa H	416	531	0.78	[0.75; 0.82]	0.1%	0.5%
Carrillo−Vega MF	416	1128	0.37	[0.34; 0.40]	0.2%	0.5%
Sokolovic S	423	735	0.58	[0.54; 0.61]	0.1%	0.5%
Golbahar J	432	500	0.86	[0.83; 0.89]	0.1%	0.5%
Solis−Urra P	445	686	0.65	[0.61; 0.68]	0.1%	0.5%
Arnljots R	448	545	0.82	[0.79; 0.85]	0.1%	0.5%
Herrick KA	496	3267	0.15	[0.14; 0.16]	0.6%	0.5%
NHANES(2015...2016)	500	2618	0.19	[0.18; 0.21]	0.4%	0.5%
Marzban M	505	1806	0.28	[0.26; 0.30]	0.3%	0.5%
Oberg J	536	890	0.60	[0.57; 0.63]	0.2%	0.5%
Sherief LM	542	572	0.95	[0.93; 0.96]	0.1%	0.5%
Moy FM	557	770	0.72	[0.69; 0.75]	0.1%	0.5%
Flores ME	599	3482	0.17	[0.16; 0.18]	0.6%	0.5%
Chlebna−Sokó.. D	606	720	0.84	[0.81; 0.87]	0.1%	0.5%
Kapil U	618	848	0.73	[0.70; 0.76]	0.1%	0.5%
Al−Daghri NM	619	830	0.75	[0.71; 0.78]	0.1%	0.5%
Nikooyeh B	622	667	0.93	[0.91; 0.95]	0.1%	0.5%
NHANES(2017...2018)	633	2094	0.30	[0.28; 0.32]	0.4%	0.5%
Cairncross CT	642	1329	0.48	[0.46; 0.51]	0.2%	0.5%
Solis−Urra P	642	1245	0.52	[0.49; 0.54]	0.2%	0.5%
Herrick KA	648	2355	0.28	[0.26; 0.29]	0.4%	0.5%
Hutchings N	651	1206	0.54	[0.51; 0.57]	0.2%	0.5%
Capuano R	686	1200	0.57	[0.54; 0.60]	0.2%	0.5%
Chung IH	710	1212	0.59	[0.56; 0.61]	0.2%	0.5%
Al−Daghri NM	711	808	0.88	[0.86; 0.90]	0.1%	0.5%
NHANES(2015...2016)	753	2413	0.31	[0.29; 0.33]	0.4%	0.5%
Karonova T	760	1664	0.46	[0.43; 0.48]	0.3%	0.5%
Li L	806	2317	0.35	[0.33; 0.37]	0.4%	0.5%
Jayashri R	823	1500	0.55	[0.52; 0.57]	0.3%	0.5%
Shchubelka K	847	1639	0.52	[0.49; 0.54]	0.3%	0.5%
Herrick KA	849	3496	0.24	[0.23; 0.26]	0.6%	0.5%
Ikonen H	857	3650	0.23	[0.22; 0.25]	0.6%	0.5%
Öztürk ZA	877	1161	0.76	[0.73; 0.78]	0.2%	0.5%
Ceccarelli M	939	2140	0.44	[0.42; 0.46]	0.4%	0.5%
Gromova O	943	1347	0.70	[0.67; 0.72]	0.2%	0.5%
Fang F	949	1814	0.52	[0.50; 0.55]	0.3%	0.5%
Santos A	1035	1500	0.69	[0.67; 0.71]	0.3%	0.5%
Petrenya N	1103	4465	0.25	[0.23; 0.26]	0.8%	0.5%
Aleteng Q	1105	1829	0.60	[0.58; 0.63]	0.3%	0.5%
Herrick KA	1119	3564	0.31	[0.30; 0.33]	0.6%	0.5%
Al−Taiar A	1140	1416	0.81	[0.78; 0.83]	0.2%	0.5%
Sulimani RA	1191	1618	0.74	[0.71; 0.76]	0.3%	0.5%
Yu S	1215	2173	0.56	[0.54; 0.58]	0.4%	0.5%
Hansen L	1281	2565	0.50	[0.48; 0.52]	0.4%	0.5%
Nikooyeh B	1318	1406	0.94	[0.92; 0.95]	0.2%	0.5%
Beer RJ	1346	6470	0.21	[0.20; 0.22]	1.1%	0.5%
Jayatissa R	1484	2525	0.59	[0.57; 0.61]	0.4%	0.5%
Borissova AM	1528	2016	0.76	[0.74; 0.78]	0.3%	0.5%
Zhao Y	1677	2661	0.63	[0.61; 0.65]	0.5%	0.5%
Yang K	1681	1982	0.85	[0.83; 0.86]	0.3%	0.5%
AlQuaiz AM	1686	2832	0.60	[0.58; 0.61]	0.5%	0.5%
Metwally ASM	1692	1864	0.91	[0.89; 0.92]	0.3%	0.5%
Beer RJ	1706	7170	0.24	[0.23; 0.25]	1.2%	0.5%
Chirita−Emandi A	1731	6631	0.26	[0.25; 0.27]	1.1%	0.5%
Kim YS	1805	2314	0.78	[0.76; 0.80]	0.4%	0.5%
Al Shaikh AM	1829	1906	0.96	[0.95; 0.97]	0.3%	0.5%
Beer RJ	1887	6813	0.28	[0.27; 0.29]	1.2%	0.5%
Al−Daghri NM	1901	2225	0.85	[0.84; 0.87]	0.4%	0.5%
Duarte C	2170	3092	0.70	[0.69; 0.72]	0.5%	0.5%
Chen J	2491	6014	0.41	[0.40; 0.43]	1.0%	0.5%
Al−Daghri NM	2609	3111	0.84	[0.83; 0.85]	0.5%	0.5%
Joukar F	2729	5096	0.54	[0.52; 0.55]	0.9%	0.5%
Al−Daghri NM	2780	4247	0.65	[0.64; 0.67]	0.7%	0.5%
El−Khateeb M	2815	4056	0.69	[0.68; 0.71]	0.7%	0.5%
Yousef S	3248	8161	0.40	[0.39; 0.41]	1.4%	0.5%
Al Shaikh A	3289	3613	0.91	[0.90; 0.92]	0.6%	0.5%
Aspell N	3317	6004	0.55	[0.54; 0.57]	1.0%	0.5%
Gökta O	3454	11893	0.29	[0.28; 0.30]	2.0%	0.5%
Beer RJ	3587	16454	0.22	[0.21; 0.22]	2.8%	0.5%
Li H	3907	4989	0.78	[0.77; 0.79]	0.9%	0.5%
Li H	4078	5707	0.71	[0.70; 0.73]	1.0%	0.5%
Han B	5496	6597	0.83	[0.82; 0.84]	1.1%	0.5%
Jiang W	7185	14302	0.50	[0.49; 0.51]	2.4%	0.5%
Griffin TP	7610	15319	0.50	[0.49; 0.50]	2.6%	0.5%
Al Zarooni AAR	10314	12346	0.84	[0.83; 0.84]	2.1%	0.5%
Eloi M	13222	39004	0.34	[0.33; 0.34]	6.7%	0.5%
Lee J	50995	68457	0.74	[0.74; 0.75]	11.7%	0.5%
Kim SY	79950	157211	0.51	[0.51; 0.51]	26.9%	0.5%
**Common effect model**		**584759**	**0.50**	**[0.50; 0.50]**	**100.0%**	**−−**
**Random effects model**			**0.47**	**[0.43; 0.51]**	**−−**	**100.0%**

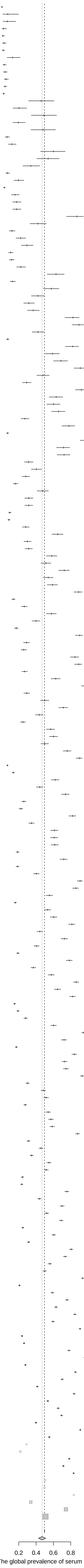

Heterogeneity: $I^2 = 100\%$, $\tau^2 = 0.0953$, $p = 0$

Supplementary figure 7 The global prevalence of serum 25(OH)D < 50 nmol/L in 2011–2022

Appendix 5: The global prevalence of vitamin D deficiency from 2000–2010 to 2011–2022

Study	Events	Total	Proportion	95%−CI	Weight (common)	Weight (random)
Riverin B	41	52	0.79	[0.65; 0.89]	0.0%	0.9%
Kremer R	53	90	0.59	[0.48; 0.69]	0.0%	0.9%
Maguire JL	75	91	0.82	[0.73; 0.90]	0.0%	0.9%
Peters BS	84	136	0.62	[0.53; 0.70]	0.0%	0.9%
Bhattoa HP	109	206	0.53	[0.46; 0.60]	0.1%	0.9%
Mutua AM	113	302	0.37	[0.32; 0.43]	0.1%	0.9%
Cinar N	114	118	0.97	[0.92; 0.99]	0.0%	0.9%
Miljkovic I	114	424	0.27	[0.23; 0.31]	0.1%	0.9%
Ramakrishnan S	117	237	0.49	[0.43; 0.56]	0.1%	0.9%
Bhatt SP	120	137	0.88	[0.81; 0.93]	0.0%	0.9%
Hirani V	133	174	0.76	[0.69; 0.83]	0.0%	0.9%
Bachhel R	135	150	0.90	[0.84; 0.94]	0.0%	0.9%
Gill TK	150	265	0.57	[0.50; 0.63]	0.1%	0.9%
Zhu Z	164	183	0.90	[0.84; 0.94]	0.0%	0.9%
Gill TK	165	263	0.63	[0.57; 0.69]	0.1%	0.9%
El Hayek J	178	282	0.63	[0.57; 0.69]	0.1%	0.9%
Greene−Finestone LS	185	312	0.59	[0.54; 0.65]	0.1%	0.9%
Le Goaziou MF	188	196	0.96	[0.92; 0.98]	0.1%	0.9%
Tseng M	192	194	0.99	[0.96; 1.00]	0.1%	0.9%
Madsen KH	197	340	0.58	[0.52; 0.63]	0.1%	0.9%
Abiaka C	202	206	0.98	[0.95; 0.99]	0.1%	0.9%
Granlund L	208	216	0.96	[0.93; 0.98]	0.1%	0.9%
Madsen KH	211	415	0.51	[0.46; 0.56]	0.1%	0.9%
Santos BR	212	234	0.91	[0.86; 0.94]	0.1%	0.9%
Robinson PJ	238	267	0.89	[0.85; 0.93]	0.1%	0.9%
Ho−Pham LT	240	637	0.38	[0.34; 0.42]	0.2%	0.9%
Gill TK	245	390	0.63	[0.58; 0.68]	0.1%	0.9%
Hirani V	248	304	0.82	[0.77; 0.86]	0.1%	0.9%
Djennane M	254	435	0.58	[0.54; 0.63]	0.1%	0.9%
Janssen HC	276	400	0.69	[0.64; 0.74]	0.1%	0.9%
Orces CH	277	403	0.69	[0.64; 0.73]	0.1%	0.9%
Klenk J	291	1418	0.21	[0.18; 0.23]	0.4%	1.0%
Mallah EM	292	300	0.97	[0.95; 0.99]	0.1%	0.9%
Oliveri B	307	339	0.91	[0.87; 0.93]	0.1%	0.9%
Gill TK	311	500	0.62	[0.58; 0.66]	0.1%	0.9%
Samefors M	318	333	0.95	[0.93; 0.97]	0.1%	0.9%
Matheï C	323	367	0.88	[0.84; 0.91]	0.1%	0.9%
Harinarayan CV	328	913	0.36	[0.33; 0.39]	0.2%	0.9%
Gill TK	333	486	0.69	[0.64; 0.73]	0.1%	0.9%
Gill TK	346	510	0.68	[0.64; 0.72]	0.1%	0.9%
Hekimsoy Z	347	391	0.89	[0.85; 0.92]	0.1%	0.9%
Sioen I	350	357	0.98	[0.96; 0.99]	0.1%	0.9%
Karagüzel G	350	746	0.47	[0.43; 0.51]	0.2%	0.9%
Vallianou N	352	490	0.72	[0.68; 0.76]	0.1%	0.9%
Lopes JB	363	415	0.87	[0.84; 0.90]	0.1%	0.9%
Aucoin M	405	461	0.88	[0.85; 0.91]	0.1%	0.9%
Mitchell DM	407	634	0.64	[0.60; 0.68]	0.2%	0.9%
Ganmaa D	419	420	1.00	[0.99; 1.00]	0.1%	0.9%
Hirani V	421	522	0.81	[0.77; 0.84]	0.1%	0.9%
Gilbert−Diamond D	430	479	0.90	[0.87; 0.92]	0.1%	0.9%
Greene−Finestone LS	464	823	0.56	[0.53; 0.60]	0.2%	0.9%
Unger MD	473	603	0.78	[0.75; 0.82]	0.2%	0.9%
Greene−Finestone LS	484	777	0.62	[0.59; 0.66]	0.2%	0.9%
Orces CH	510	724	0.70	[0.67; 0.74]	0.2%	0.9%
Ardawi MS	510	843	0.60	[0.57; 0.64]	0.2%	0.9%
Hirani V	554	659	0.84	[0.81; 0.87]	0.2%	0.9%
Robinson PJ	562	640	0.88	[0.85; 0.90]	0.2%	0.9%
Science M	565	743	0.76	[0.73; 0.79]	0.2%	0.9%
Feng X	572	686	0.83	[0.80; 0.86]	0.2%	0.9%
Aucoin M	617	756	0.82	[0.79; 0.84]	0.2%	0.9%
Choi HR	626	651	0.96	[0.94; 0.97]	0.2%	0.9%
Daly RM	673	810	0.83	[0.80; 0.86]	0.2%	0.9%
Hovsepian S	725	1111	0.65	[0.62; 0.68]	0.3%	0.9%
Orces CH	750	1158	0.65	[0.62; 0.68]	0.3%	1.0%
Mogire RM	780	1507	0.52	[0.49; 0.54]	0.4%	1.0%
Mehboobali N	810	858	0.94	[0.93; 0.96]	0.2%	0.9%
González−Gross M	817	1006	0.81	[0.79; 0.84]	0.3%	0.9%
Riverin B	834	944	0.88	[0.86; 0.90]	0.3%	0.9%
Daly RM	868	1393	0.62	[0.60; 0.65]	0.4%	1.0%
Kaykhaei MA	940	993	0.95	[0.93; 0.96]	0.3%	0.9%
González−Molero I	968	1262	0.77	[0.74; 0.79]	0.3%	1.0%
Batieha A	991	4590	0.22	[0.20; 0.23]	1.2%	1.0%
Ardawi MS	1034	1172	0.88	[0.86; 0.90]	0.3%	1.0%
Shivane VK	1055	1137	0.93	[0.91; 0.94]	0.3%	1.0%
Alkerwi A	1107	1335	0.83	[0.81; 0.85]	0.4%	1.0%
Mansbach JM	1131	1799	0.63	[0.61; 0.65]	0.5%	1.0%
Orwoll E	1142	1606	0.71	[0.69; 0.73]	0.4%	1.0%
Chailurkit LO	1194	2641	0.45	[0.43; 0.47]	0.7%	1.0%
Daly RM	1235	1564	0.79	[0.77; 0.81]	0.4%	1.0%
Zhu Z	1271	1440	0.88	[0.86; 0.90]	0.4%	1.0%
Zhu Z	1555	2269	0.69	[0.67; 0.70]	0.6%	1.0%
Daly RM	1598	2011	0.79	[0.78; 0.81]	0.5%	1.0%
Penrose K	1685	2160	0.78	[0.76; 0.80]	0.6%	1.0%
Rabenberg M	1687	1816	0.93	[0.92; 0.94]	0.5%	1.0%
Hirani V	1788	2070	0.86	[0.85; 0.88]	0.5%	1.0%
Daly RM	1824	2556	0.71	[0.70; 0.73]	0.7%	1.0%
Mansbach JM	2013	2759	0.73	[0.71; 0.75]	0.7%	1.0%
Rabenberg M	2054	2456	0.84	[0.82; 0.85]	0.7%	1.0%
Daly RM	2202	2884	0.76	[0.75; 0.78]	0.8%	1.0%
Manios Y	2266	2353	0.96	[0.95; 0.97]	0.6%	1.0%
Rabenberg M	2465	2723	0.91	[0.89; 0.92]	0.7%	1.0%
Chao YS	2896	6101	0.47	[0.46; 0.49]	1.6%	1.0%
Wyskida M	3002	3472	0.86	[0.85; 0.88]	0.9%	1.0%
Voortman T	3020	4167	0.72	[0.71; 0.74]	1.1%	1.0%
Langlois K	3428	5306	0.65	[0.63; 0.66]	1.4%	1.0%
Schramm S	3456	4149	0.83	[0.82; 0.84]	1.1%	1.0%
Leung RY	4752	5276	0.90	[0.89; 0.91]	1.4%	1.0%
Saliba W	5237	6518	0.80	[0.79; 0.81]	1.7%	1.0%
Tolppanen AM	5631	7560	0.74	[0.73; 0.75]	2.0%	1.0%
Liu X	5785	7485	0.77	[0.76; 0.78]	2.0%	1.0%
Liu X	6041	8182	0.74	[0.73; 0.75]	2.2%	1.0%
Hintzpeter B	7911	9001	0.88	[0.87; 0.89]	2.4%	1.0%
Liu X	7985	10343	0.77	[0.76; 0.78]	2.7%	1.0%
Song HR	8720	8976	0.97	[0.97; 0.97]	2.4%	1.0%
Chung JY	16973	18305	0.93	[0.92; 0.93]	4.8%	1.0%
Saliba W	160952	192240	0.84	[0.84; 0.84]	50.9%	1.0%
**Common effect model**		**377509**	**0.81**	**[0.81; 0.81]**	**100.0%**	**−−**
**Random effects model**			**0.77**	**[0.74; 0.81]**	**−−**	**100.0%**

Heterogeneity: $I^2 = 100\%$, $\tau^2 = 0.0465$, $p = 0$

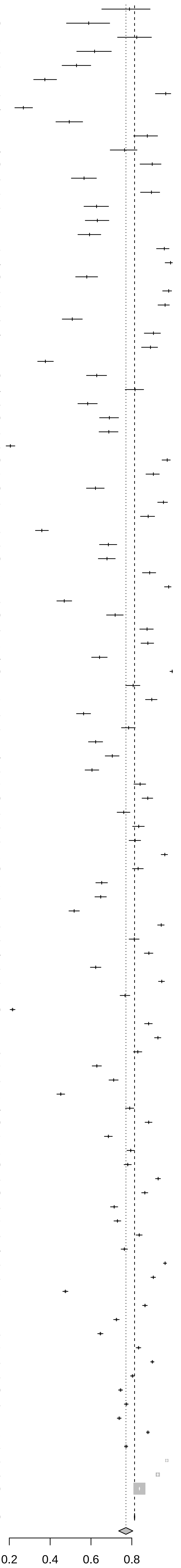

Supplementary figure 8 The global prevalence of serum 25(OH)D < 75 nmol/L in 2000–2010

Appendix 5: The global prevalence of vitamin D deficiency from 2000–2010 to 2011–2022

Study	Events	Total	Proportion	95%−CI	Weight (common)	Weight (random)
Smith G	4	41	0.10	[0.03; 0.23]	0.0%	0.8%
White Z	39	59	0.66	[0.53; 0.78]	0.0%	0.8%
Abdulrahman MA	40	50	0.80	[0.66; 0.90]	0.0%	0.8%
Abdulrahman MA	45	63	0.71	[0.59; 0.82]	0.0%	0.8%
Smith G	54	128	0.42	[0.34; 0.51]	0.1%	0.8%
Naqvi A	55	86	0.64	[0.53; 0.74]	0.0%	0.8%
Lin L	61	353	0.17	[0.13; 0.22]	0.2%	0.9%
Ginter JK	87	224	0.39	[0.32; 0.46]	0.1%	0.9%
Tangoh DA	96	372	0.26	[0.21; 0.31]	0.2%	0.9%
Hribar M	104	125	0.83	[0.75; 0.89]	0.1%	0.8%
Brinkmann K	107	108	0.99	[0.95; 1.00]	0.1%	0.8%
Ten Haaf DSM	108	450	0.24	[0.20; 0.28]	0.2%	0.9%
Naeem Z	121	180	0.67	[0.60; 0.74]	0.1%	0.9%
Hribar M	131	155	0.85	[0.78; 0.90]	0.1%	0.9%
Pan T	137	194	0.71	[0.64; 0.77]	0.1%	0.9%
Kagotho E	152	253	0.60	[0.54; 0.66]	0.1%	0.9%
Bettencourt A	155	198	0.78	[0.72; 0.84]	0.1%	0.9%
Cabral MA	156	234	0.67	[0.60; 0.73]	0.1%	0.9%
Abdulrahman MA	161	278	0.58	[0.52; 0.64]	0.1%	0.9%
Lin L	161	477	0.34	[0.30; 0.38]	0.3%	0.9%
Srimani S	167	222	0.75	[0.69; 0.81]	0.1%	0.9%
Lin L	173	860	0.20	[0.17; 0.23]	0.5%	0.9%
Yousef S	179	267	0.67	[0.61; 0.73]	0.1%	0.9%
Smith G	185	495	0.37	[0.33; 0.42]	0.3%	0.9%
Maldonado G	188	269	0.70	[0.64; 0.75]	0.1%	0.9%
Kouda K	198	400	0.50	[0.44; 0.55]	0.2%	0.9%
Hazell TJ	203	488	0.42	[0.37; 0.46]	0.3%	0.9%
Jolliffe DA	205	222	0.92	[0.88; 0.95]	0.1%	0.9%
Nadeem S	213	221	0.96	[0.93; 0.98]	0.1%	0.9%
Al Hayek S	214	344	0.62	[0.57; 0.67]	0.2%	0.9%
Rafraf M	215	216	1.00	[0.97; 1.00]	0.1%	0.9%
Karin Z	226	260	0.87	[0.82; 0.91]	0.1%	0.9%
Zhu W	227	508	0.45	[0.40; 0.49]	0.3%	0.9%
Sherchand O	236	300	0.79	[0.74; 0.83]	0.2%	0.9%
Kunz C	241	327	0.74	[0.69; 0.78]	0.2%	0.9%
Saeed BQ	244	287	0.85	[0.80; 0.89]	0.2%	0.9%
Black LJ	245	331	0.74	[0.69; 0.79]	0.2%	0.9%
Feketea GM	249	376	0.66	[0.61; 0.71]	0.2%	0.9%
Sheikh A	253	300	0.84	[0.80; 0.88]	0.2%	0.9%
Horton−French K	257	400	0.64	[0.59; 0.69]	0.2%	0.9%
El Hayek J	257	508	0.51	[0.46; 0.55]	0.3%	0.9%
Sokolovic S	263	450	0.58	[0.54; 0.63]	0.2%	0.9%
Contreras−Manzano A	287	381	0.75	[0.71; 0.80]	0.2%	0.9%
Khan AH	292	305	0.96	[0.93; 0.98]	0.2%	0.9%
Contreras−Manzano A	324	404	0.80	[0.76; 0.84]	0.2%	0.9%
Man PW	332	416	0.80	[0.76; 0.84]	0.2%	0.9%
Abu−Samak MS	338	371	0.91	[0.88; 0.94]	0.2%	0.9%
Black LJ	347	490	0.71	[0.67; 0.75]	0.3%	0.9%
Vierucci F	351	427	0.82	[0.78; 0.86]	0.2%	0.9%
Kapil U	356	374	0.95	[0.93; 0.97]	0.2%	0.9%
Contreras−Manzano A	367	477	0.77	[0.73; 0.81]	0.3%	0.9%
Rahman A	393	410	0.96	[0.93; 0.98]	0.2%	0.9%
Kunz C	400	456	0.88	[0.84; 0.91]	0.2%	0.9%
Arabi A	427	466	0.92	[0.89; 0.94]	0.3%	0.9%
NHANES(2017...2018)	439	1321	0.33	[0.31; 0.36]	0.7%	0.9%
Black LJ	440	652	0.67	[0.64; 0.71]	0.4%	0.9%
Horton−French K	445	692	0.64	[0.61; 0.68]	0.4%	0.9%
Black LJ	450	648	0.69	[0.66; 0.73]	0.3%	0.9%
Kunz C	453	520	0.87	[0.84; 0.90]	0.3%	0.9%
Saki F	458	477	0.96	[0.94; 0.98]	0.3%	0.9%
Kunz C	469	528	0.89	[0.86; 0.91]	0.3%	0.9%
NHANES(2015...2016)	474	1231	0.39	[0.36; 0.41]	0.7%	0.9%
Hansen L	482	527	0.91	[0.89; 0.94]	0.3%	0.9%
Koyama S	486	492	0.99	[0.97; 1.00]	0.3%	0.9%
Isa H	496	531	0.93	[0.91; 0.95]	0.3%	0.9%
Arnljots R	510	545	0.94	[0.91; 0.95]	0.3%	0.9%
Sokolovic S	513	645	0.80	[0.76; 0.83]	0.3%	0.9%
Flores ME	520	713	0.73	[0.70; 0.76]	0.4%	0.9%
Drali O	528	621	0.85	[0.82; 0.88]	0.3%	0.9%
Ní Chaoimh C	530	741	0.72	[0.68; 0.75]	0.4%	0.9%
Black LJ	540	730	0.74	[0.71; 0.77]	0.4%	0.9%
Sherief LM	566	572	0.99	[0.98; 1.00]	0.3%	0.9%
Sokolovic S	659	735	0.90	[0.87; 0.92]	0.4%	0.9%
Souberbielle JC	716	892	0.80	[0.78; 0.83]	0.5%	0.9%
Hoge A	719	915	0.79	[0.76; 0.81]	0.5%	0.9%
Yousef S	723	858	0.84	[0.82; 0.87]	0.5%	0.9%
Yousef S	727	932	0.78	[0.75; 0.81]	0.5%	0.9%
Kapil U	767	848	0.90	[0.88; 0.92]	0.5%	0.9%
de Oliveira CL	777	1152	0.67	[0.65; 0.70]	0.6%	0.9%
Oberg J	780	890	0.88	[0.85; 0.90]	0.5%	0.9%
Yousef S	799	1361	0.59	[0.56; 0.61]	0.7%	0.9%
NHANES(2017...2018)	950	1810	0.52	[0.50; 0.55]	1.0%	0.9%
NHANES (2015...2016)	992	1777	0.56	[0.53; 0.58]	1.0%	0.9%
Capuano R	1053	1200	0.88	[0.86; 0.90]	0.6%	0.9%
Öztürk ZA	1102	1161	0.95	[0.93; 0.96]	0.6%	0.9%
Chung IH	1177	1212	0.97	[0.96; 0.98]	0.7%	0.9%
Cairncross CT	1183	1329	0.89	[0.87; 0.91]	0.7%	0.9%
Gromova O	1232	1347	0.91	[0.90; 0.93]	0.7%	0.9%
Jayashri R	1360	1500	0.91	[0.89; 0.92]	0.8%	0.9%
Al−Taiar A	1365	1416	0.96	[0.95; 0.97]	0.8%	0.9%
Karonova T	1384	1664	0.83	[0.81; 0.85]	0.9%	0.9%
Lima−Costa MF	1404	2264	0.62	[0.60; 0.64]	1.2%	0.9%
NHANES(2017...2018)	1500	2184	0.69	[0.67; 0.71]	1.2%	0.9%
NHANES(2017...2018)	1533	2094	0.73	[0.71; 0.75]	1.1%	0.9%
Ceccarelli M	1657	2140	0.77	[0.76; 0.79]	1.2%	0.9%
Aleteng Q	1659	1829	0.91	[0.89; 0.92]	1.0%	0.9%
Li L	1683	2317	0.73	[0.71; 0.74]	1.2%	0.9%
NHANES(2015...2016)	1730	2413	0.72	[0.70; 0.73]	1.3%	0.9%
NHANES(2015...2016)	1833	2618	0.70	[0.68; 0.72]	1.4%	0.9%
Qorbani M	1846	2596	0.71	[0.69; 0.73]	1.4%	0.9%
Yang K	1893	1982	0.96	[0.95; 0.96]	1.1%	0.9%
Hansen L	2045	2565	0.80	[0.78; 0.81]	1.4%	0.9%
AlQuaiz AM	2215	2832	0.78	[0.77; 0.80]	1.5%	0.9%
Zhao Y	2523	2661	0.95	[0.94; 0.96]	1.4%	0.9%
Ikonen H	2597	3650	0.71	[0.70; 0.73]	2.0%	0.9%
Flores ME	2612	3482	0.75	[0.74; 0.76]	1.9%	0.9%
Duarte C	3000	3092	0.97	[0.96; 0.98]	1.7%	0.9%
Jääskeläinen T	3068	4051	0.76	[0.74; 0.77]	2.2%	0.9%
Petrenya N	3313	4465	0.74	[0.73; 0.75]	2.4%	0.9%
El−Khateeb M	3548	4056	0.87	[0.86; 0.88]	2.2%	0.9%
Chirita−Emandi A	3926	6631	0.59	[0.58; 0.60]	3.6%	0.9%
Yousef S	6386	8161	0.78	[0.77; 0.79]	4.4%	0.9%
Al Zarooni AAR	11765	12346	0.95	[0.95; 0.96]	6.6%	0.9%
Jiang W	11874	14302	0.83	[0.82; 0.84]	7.7%	0.9%
Eloi M	27576	39004	0.71	[0.70; 0.71]	21.0%	0.9%
**Common effect model**		**185976**	**0.78**	**[0.77; 0.78]**	**100.0%**	**−−**
**Random effects model**			**0.76**	**[0.72; 0.80]**	**−−**	**100.0%**

Heterogeneity: $I^2 = 99\%$, $\tau^2 = 0.0540$, $p = 0$

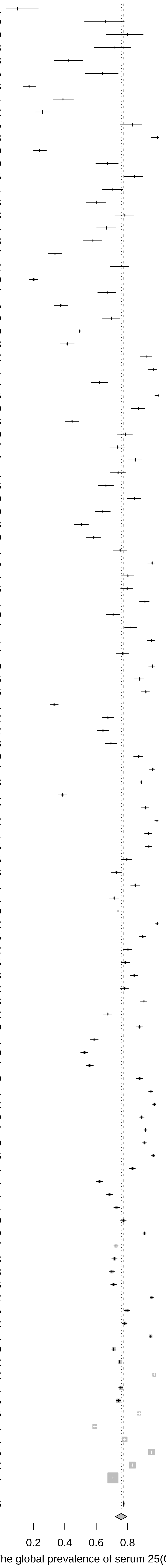

Supplementary figure 9 The global prevalence of serum 25(OH)D < 75 nmol/L in 2011–2022

Appendix 6: The prevalence of serum 25(OH)D < 75 nmol/L by six WHO regions

Study	Events	Total	Proportion	95%−CI	Weight (common)	Weight (random)
**WHO = AR**						
Djennane M	254	435	0.58	[0.54; 0.63]	0.1%	0.5%
Drali O	528	621	0.85	[0.82; 0.88]	0.1%	0.5%
Kagotho E	152	253	0.60	[0.54; 0.66]	0.0%	0.4%
Mogire RM	780	1507	0.52	[0.49; 0.54]	0.3%	0.5%
Mutua AM	113	302	0.37	[0.32; 0.43]	0.1%	0.5%
Tangoh DA	96	372	0.26	[0.21; 0.31]	0.1%	0.5%
White Z	39	59	0.66	[0.53; 0.78]	0.0%	0.4%
Common effect model		3549	0.56	[0.54; 0.57]	0.6%	−−
Random effects model			0.55	[0.40; 0.70]	−−	3.1%
Heterogeneity: $I^2 = 99\%$, $\tau^2 = 0.0418$, $p < 0.01$						
**WHO = EMR**						
Abdulrahman MA	40	50	0.80	[0.66; 0.90]	0.0%	0.4%
Abdulrahman MA	45	63	0.71	[0.59; 0.82]	0.0%	0.4%
Abdulrahman MA	161	278	0.58	[0.52; 0.64]	0.0%	0.4%
Abiaka C	202	206	0.98	[0.95; 0.99]	0.0%	0.4%
Abu−Samak MS	338	371	0.91	[0.88; 0.94]	0.1%	0.5%
Al Hayek S	214	344	0.62	[0.57; 0.67]	0.1%	0.5%
Al Zarooni AAR	11765	12346	0.95	[0.95; 0.96]	2.2%	0.5%
AlQuaiz AM	2215	2832	0.78	[0.77; 0.80]	0.5%	0.5%
Al−Taiar A	1365	1416	0.96	[0.95; 0.97]	0.3%	0.5%
Arabi A	427	466	0.92	[0.89; 0.94]	0.1%	0.5%
Ardawi MS	510	843	0.60	[0.57; 0.64]	0.1%	0.5%
Ardawi MS	1034	1172	0.88	[0.86; 0.90]	0.2%	0.5%
Batieha A	991	4590	0.22	[0.20; 0.23]	0.8%	0.5%
El−Khateeb M	3548	4056	0.87	[0.86; 0.88]	0.7%	0.5%
Hazell TJ	203	488	0.42	[0.37; 0.46]	0.1%	0.5%
Hovsepian S	725	1111	0.65	[0.62; 0.68]	0.2%	0.5%
Isa H	496	531	0.93	[0.91; 0.95]	0.1%	0.5%
Kaykhaei MA	940	993	0.95	[0.93; 0.96]	0.2%	0.5%
Khan AH	292	305	0.96	[0.93; 0.98]	0.1%	0.5%
Mallah EM	292	300	0.97	[0.95; 0.99]	0.1%	0.5%
Mehboobali N	810	858	0.94	[0.93; 0.96]	0.2%	0.5%
Nadeem S	213	221	0.96	[0.93; 0.98]	0.0%	0.4%
Naeem Z	121	180	0.67	[0.60; 0.74]	0.0%	0.4%
Qorbani M	1846	2596	0.71	[0.69; 0.73]	0.5%	0.5%
Rafraf M	215	216	1.00	[0.97; 1.00]	0.0%	0.4%
Rahman A	393	410	0.96	[0.93; 0.98]	0.1%	0.5%
Saeed BQ	244	287	0.85	[0.80; 0.89]	0.1%	0.5%
Saki F	458	477	0.96	[0.94; 0.98]	0.1%	0.5%
Sheikh A	253	300	0.84	[0.80; 0.88]	0.1%	0.5%
Sherief LM	566	572	0.99	[0.98; 1.00]	0.1%	0.5%
Common effect model		38878	0.83	[0.83; 0.84]	6.9%	−−
Random effects model			0.85	[0.78; 0.91]	−−	13.5%
Heterogeneity: $I^2 = 100\%$, $\tau^2 = 0.0593$, $p = 0$						
**WHO = ER**						
Alkerwi A	1107	1335	0.83	[0.81; 0.85]	0.2%	0.5%
Arnljots R	510	545	0.94	[0.91; 0.95]	0.1%	0.5%
Bettencourt A	155	198	0.78	[0.72; 0.84]	0.0%	0.4%
Bhattoa HP	109	206	0.53	[0.46; 0.60]	0.0%	0.4%
Capuano R	1053	1200	0.88	[0.86; 0.90]	0.2%	0.5%
Ceccarelli M	1657	2140	0.77	[0.76; 0.79]	0.4%	0.5%
Chirita−Emandi A	3926	6631	0.59	[0.58; 0.60]	1.2%	0.5%
Cinar N	114	118	0.97	[0.92; 0.99]	0.0%	0.4%
Duarte C	3000	3092	0.97	[0.96; 0.98]	0.5%	0.5%
Feketea GM	249	376	0.66	[0.61; 0.71]	0.1%	0.5%
González−Gross M	817	1006	0.81	[0.79; 0.84]	0.2%	0.5%
González−Molero I	968	1262	0.77	[0.74; 0.79]	0.2%	0.5%
Granlund L	208	216	0.96	[0.93; 0.98]	0.0%	0.4%
Gromova O	1232	1347	0.91	[0.90; 0.93]	0.2%	0.5%
Hansen L	482	527	0.91	[0.89; 0.94]	0.1%	0.5%
Hansen L	2045	2565	0.80	[0.78; 0.81]	0.5%	0.5%
Hekimsoy Z	347	391	0.89	[0.85; 0.92]	0.1%	0.5%
Hintzpeter B	7911	9001	0.88	[0.87; 0.89]	1.6%	0.5%
Hirani V	1788	2070	0.86	[0.85; 0.88]	0.4%	0.5%
Hoge A	719	915	0.79	[0.76; 0.81]	0.2%	0.5%
Hribar M	104	125	0.83	[0.75; 0.89]	0.0%	0.4%
Hribar M	131	155	0.85	[0.78; 0.90]	0.0%	0.4%
Ikonen H	2597	3650	0.71	[0.70; 0.73]	0.6%	0.5%
Jääskeläinen T	3068	4051	0.76	[0.74; 0.77]	0.7%	0.5%
Janssen HC	276	400	0.69	[0.64; 0.74]	0.1%	0.5%
Jolliffe DA	205	222	0.92	[0.88; 0.95]	0.0%	0.4%
Karagüzel G	350	746	0.47	[0.43; 0.51]	0.1%	0.5%
Karin Z	226	260	0.87	[0.82; 0.91]	0.0%	0.4%
Karonova T	1384	1664	0.83	[0.81; 0.85]	0.3%	0.5%
Klenk J	291	1418	0.21	[0.18; 0.23]	0.3%	0.5%
Kunz C	241	327	0.74	[0.69; 0.78]	0.1%	0.5%
Kunz C	400	456	0.88	[0.84; 0.91]	0.1%	0.5%
Kunz C	453	520	0.87	[0.84; 0.90]	0.1%	0.5%
Kunz C	469	528	0.89	[0.86; 0.91]	0.1%	0.5%
Le Goaziou MF	188	196	0.96	[0.92; 0.98]	0.0%	0.4%
Madsen KH	197	340	0.58	[0.52; 0.63]	0.1%	0.5%
Madsen KH	211	415	0.51	[0.46; 0.56]	0.1%	0.5%
Man PW	332	416	0.80	[0.76; 0.84]	0.1%	0.5%
Manios Y	2266	2353	0.96	[0.95; 0.97]	0.4%	0.5%
Matheï C	323	367	0.88	[0.84; 0.91]	0.1%	0.5%
Ní Chaoimh C	530	741	0.72	[0.68; 0.75]	0.1%	0.5%
Oberg J	780	890	0.88	[0.85; 0.90]	0.2%	0.5%
Öztürk ZA	1102	1161	0.95	[0.93; 0.96]	0.2%	0.5%
Petrenya N	3313	4465	0.74	[0.73; 0.75]	0.8%	0.5%
Rabenberg M	1687	1816	0.93	[0.92; 0.94]	0.3%	0.5%
Rabenberg M	2054	2456	0.84	[0.82; 0.85]	0.4%	0.5%
Rabenberg M	2465	2723	0.91	[0.89; 0.92]	0.5%	0.5%
Saliba W	5237	6518	0.80	[0.79; 0.81]	1.2%	0.5%
Saliba W	160952	192240	0.84	[0.84; 0.84]	34.1%	0.5%
Samefors M	318	333	0.95	[0.93; 0.97]	0.1%	0.5%
Schramm S	3456	4149	0.83	[0.82; 0.84]	0.7%	0.5%
Sioen I	350	357	0.98	[0.96; 0.99]	0.1%	0.5%
Sokolovic S	263	450	0.58	[0.54; 0.63]	0.1%	0.5%
Sokolovic S	513	645	0.80	[0.76; 0.83]	0.1%	0.5%
Sokolovic S	659	735	0.90	[0.87; 0.92]	0.1%	0.5%
Souberbielle JC	716	892	0.80	[0.78; 0.83]	0.2%	0.5%
Ten Haaf DSM	108	450	0.24	[0.20; 0.28]	0.1%	0.5%
Tolppanen AM	5631	7560	0.74	[0.73; 0.75]	1.3%	0.5%
Vallianou N	352	490	0.72	[0.68; 0.76]	0.1%	0.5%
Vierucci F	351	427	0.82	[0.78; 0.86]	0.1%	0.5%
Voortman T	3020	4167	0.72	[0.71; 0.74]	0.7%	0.5%
Wyskida M	3002	3472	0.86	[0.85; 0.88]	0.6%	0.5%
Common effect model		290887	0.83	[0.83; 0.83]	51.6%	−−
Random effects model			0.81	[0.77; 0.85]	−−	28.1%
Heterogeneity: $I^2 = 99\%$, $\tau^2 = 0.0362$, $p = 0$						
**WHO = RA**						
Aucoin M	405	461	0.88	[0.85; 0.91]	0.1%	0.5%
Aucoin M	617	756	0.82	[0.79; 0.84]	0.1%	0.5%
Brinkmann K	107	108	0.99	[0.95; 1.00]	0.0%	0.4%
Cabral MA	156	234	0.67	[0.60; 0.73]	0.0%	0.4%
Chao YS	2896	6101	0.47	[0.46; 0.49]	1.1%	0.5%
Contreras−Manzano A	287	381	0.75	[0.71; 0.80]	0.1%	0.5%
Contreras−Manzano A	324	404	0.80	[0.76; 0.84]	0.1%	0.5%
Contreras−Manzano A	367	477	0.77	[0.73; 0.81]	0.1%	0.5%
de Oliveira CL	777	1152	0.67	[0.65; 0.70]	0.2%	0.5%
El Hayek J	178	282	0.63	[0.57; 0.69]	0.1%	0.5%
El Hayek J	257	508	0.51	[0.46; 0.55]	0.1%	0.5%
Eloi M	27576	39004	0.71	[0.70; 0.71]	6.9%	0.5%
Flores ME	520	713	0.73	[0.70; 0.76]	0.1%	0.5%
Flores ME	2612	3482	0.75	[0.74; 0.76]	0.6%	0.5%
Gilbert−Diamond D	430	479	0.90	[0.87; 0.92]	0.1%	0.5%
Ginter JK	87	224	0.39	[0.32; 0.46]	0.0%	0.4%
Greene−Finestone LS	185	312	0.59	[0.54; 0.65]	0.1%	0.5%
Greene−Finestone LS	464	823	0.56	[0.53; 0.60]	0.1%	0.5%
Greene−Finestone LS	484	777	0.62	[0.59; 0.66]	0.1%	0.5%
Kremer R	53	90	0.59	[0.48; 0.69]	0.0%	0.4%
Langlois K	3428	5306	0.65	[0.63; 0.66]	0.9%	0.5%
Lima−Costa MF	1404	2264	0.62	[0.60; 0.64]	0.4%	0.5%
Liu X	5785	7485	0.77	[0.76; 0.78]	1.3%	0.5%
Liu X	6041	8182	0.74	[0.73; 0.75]	1.5%	0.5%
Liu X	7985	10343	0.77	[0.76; 0.78]	1.8%	0.5%
Lopes JB	363	415	0.87	[0.84; 0.90]	0.1%	0.5%
Maguire JL	75	91	0.82	[0.73; 0.90]	0.0%	0.4%
Maldonado G	188	269	0.70	[0.64; 0.75]	0.0%	0.4%
Mansbach JM	1131	1799	0.63	[0.61; 0.65]	0.3%	0.5%
Mansbach JM	2013	2759	0.73	[0.71; 0.75]	0.5%	0.5%
Miljkovic I	114	424	0.27	[0.23; 0.31]	0.1%	0.5%
Mitchell DM	407	634	0.64	[0.60; 0.68]	0.1%	0.5%
Naqvi A	55	86	0.64	[0.53; 0.74]	0.0%	0.4%
NHANES (2015...2016)	992	1777	0.56	[0.53; 0.58]	0.3%	0.5%
NHANES(2015...2016)	474	1231	0.39	[0.36; 0.41]	0.2%	0.5%
NHANES(2015...2016)	1730	2413	0.72	[0.70; 0.73]	0.4%	0.5%
NHANES(2015...2016)	1833	2618	0.70	[0.68; 0.72]	0.5%	0.5%
NHANES(2017...2018)	439	1321	0.33	[0.31; 0.36]	0.2%	0.5%
NHANES(2017...2018)	950	1810	0.52	[0.50; 0.55]	0.3%	0.5%
NHANES(2017...2018)	1500	2184	0.69	[0.67; 0.71]	0.4%	0.5%
NHANES(2017...2018)	1533	2094	0.73	[0.71; 0.75]	0.4%	0.5%
Oliveri B	307	339	0.91	[0.87; 0.93]	0.1%	0.5%
Orces CH	277	403	0.69	[0.64; 0.73]	0.1%	0.5%
Orces CH	510	724	0.70	[0.67; 0.74]	0.1%	0.5%
Orces CH	750	1158	0.65	[0.62; 0.68]	0.2%	0.5%
Orwoll E	1142	1606	0.71	[0.69; 0.73]	0.3%	0.5%
Penrose K	1685	2160	0.78	[0.76; 0.80]	0.4%	0.5%
Peters BS	84	136	0.62	[0.53; 0.70]	0.0%	0.4%
Riverin B	41	52	0.79	[0.65; 0.89]	0.0%	0.4%
Riverin B	834	944	0.88	[0.86; 0.90]	0.2%	0.5%
Santos BR	212	234	0.91	[0.86; 0.94]	0.0%	0.4%
Science M	565	743	0.76	[0.73; 0.79]	0.1%	0.5%
Tseng M	192	194	0.99	[0.96; 1.00]	0.0%	0.4%
Unger MD	473	603	0.78	[0.75; 0.82]	0.1%	0.5%
Yousef S	179	267	0.67	[0.61; 0.73]	0.0%	0.4%
Yousef S	723	858	0.84	[0.82; 0.87]	0.2%	0.5%
Yousef S	727	932	0.78	[0.75; 0.81]	0.2%	0.5%
Yousef S	799	1361	0.59	[0.56; 0.61]	0.2%	0.5%
Yousef S	6386	8161	0.78	[0.77; 0.79]	1.4%	0.5%
Common effect model		133148	0.70	[0.70; 0.71]	23.6%	−−
Random effects model			0.71	[0.67; 0.75]	−−	26.7%
Heterogeneity: $I^2 = 99\%$, $\tau^2 = 0.0291$, $p = 0$						
**WHO = SEAR**						
Bachhel R	135	150	0.90	[0.84; 0.94]	0.0%	0.4%
Bhatt SP	120	137	0.88	[0.81; 0.93]	0.0%	0.4%
Chailurkit LO	1194	2641	0.45	[0.43; 0.47]	0.5%	0.5%
Harinarayan CV	328	913	0.36	[0.33; 0.39]	0.2%	0.5%
Jayashri R	1360	1500	0.91	[0.89; 0.92]	0.3%	0.5%
Kapil U	356	374	0.95	[0.93; 0.97]	0.1%	0.5%
Kapil U	767	848	0.90	[0.88; 0.92]	0.2%	0.5%
Pan T	137	194	0.71	[0.64; 0.77]	0.0%	0.4%
Ramakrishnan S	117	237	0.49	[0.43; 0.56]	0.0%	0.4%
Sherchand O	236	300	0.79	[0.74; 0.83]	0.1%	0.5%
Shivane VK	1055	1137	0.93	[0.91; 0.94]	0.2%	0.5%
Srimani S	167	222	0.75	[0.69; 0.81]	0.0%	0.4%
Common effect model		8653	0.72	[0.71; 0.73]	1.5%	−−
Random effects model			0.77	[0.65; 0.88]	−−	5.4%
Heterogeneity: $I^2 = 100\%$, $\tau^2 = 0.0572$, $p = 0$						
**WHO = WPR**						
Aleteng Q	1659	1829	0.91	[0.89; 0.92]	0.3%	0.5%
Black LJ	245	331	0.74	[0.69; 0.79]	0.1%	0.5%
Black LJ	347	490	0.71	[0.67; 0.75]	0.1%	0.5%
Black LJ	440	652	0.67	[0.64; 0.71]	0.1%	0.5%
Black LJ	450	648	0.69	[0.66; 0.73]	0.1%	0.5%
Black LJ	540	730	0.74	[0.71; 0.77]	0.1%	0.5%
Cairncross CT	1183	1329	0.89	[0.87; 0.91]	0.2%	0.5%
Choi HR	626	651	0.96	[0.94; 0.97]	0.1%	0.5%
Chung IH	1177	1212	0.97	[0.96; 0.98]	0.2%	0.5%
Chung JY	16973	18305	0.93	[0.92; 0.93]	3.2%	0.5%
Daly RM	673	810	0.83	[0.80; 0.86]	0.1%	0.5%
Daly RM	868	1393	0.62	[0.60; 0.65]	0.2%	0.5%
Daly RM	1235	1564	0.79	[0.77; 0.81]	0.3%	0.5%
Daly RM	1598	2011	0.79	[0.78; 0.81]	0.4%	0.5%
Daly RM	1824	2556	0.71	[0.70; 0.73]	0.5%	0.5%
Daly RM	2202	2884	0.76	[0.75; 0.78]	0.5%	0.5%
Feng X	572	686	0.83	[0.80; 0.86]	0.1%	0.5%
Ganmaa D	419	420	1.00	[0.99; 1.00]	0.1%	0.5%
Gill TK	150	265	0.57	[0.50; 0.63]	0.0%	0.4%
Gill TK	165	263	0.63	[0.57; 0.69]	0.0%	0.4%
Gill TK	245	390	0.63	[0.58; 0.68]	0.1%	0.5%
Gill TK	311	500	0.62	[0.58; 0.66]	0.1%	0.5%
Gill TK	333	486	0.69	[0.64; 0.73]	0.1%	0.5%
Gill TK	346	510	0.68	[0.64; 0.72]	0.1%	0.5%
Hirani V	133	174	0.76	[0.69; 0.83]	0.0%	0.4%
Hirani V	248	304	0.82	[0.77; 0.86]	0.1%	0.5%
Hirani V	421	522	0.81	[0.77; 0.84]	0.1%	0.5%
Hirani V	554	659	0.84	[0.81; 0.87]	0.1%	0.5%
Ho−Pham LT	240	637	0.38	[0.34; 0.42]	0.1%	0.5%
Horton−French K	257	400	0.64	[0.59; 0.69]	0.1%	0.5%
Horton−French K	445	692	0.64	[0.61; 0.68]	0.1%	0.5%
Jiang W	11874	14302	0.83	[0.82; 0.84]	2.5%	0.5%
Kouda K	198	400	0.50	[0.44; 0.55]	0.1%	0.5%
Koyama S	486	492	0.99	[0.97; 1.00]	0.1%	0.5%
Leung RY	4752	5276	0.90	[0.89; 0.91]	0.9%	0.5%
Li L	1683	2317	0.73	[0.71; 0.74]	0.4%	0.5%
Lin L	61	353	0.17	[0.13; 0.22]	0.1%	0.5%
Lin L	161	477	0.34	[0.30; 0.38]	0.1%	0.5%
Lin L	173	860	0.20	[0.17; 0.23]	0.2%	0.5%
Robinson PJ	238	267	0.89	[0.85; 0.93]	0.0%	0.4%
Robinson PJ	562	640	0.88	[0.85; 0.90]	0.1%	0.5%
Smith G	4	41	0.10	[0.03; 0.23]	0.0%	0.4%
Smith G	54	128	0.42	[0.34; 0.51]	0.0%	0.4%
Smith G	185	495	0.37	[0.33; 0.42]	0.1%	0.5%
Song HR	8720	8976	0.97	[0.97; 0.97]	1.6%	0.5%
Yang K	1893	1982	0.96	[0.95; 0.96]	0.4%	0.5%
Zhao Y	2523	2661	0.95	[0.94; 0.96]	0.5%	0.5%
Zhu W	227	508	0.45	[0.40; 0.49]	0.1%	0.5%
Zhu Z	164	183	0.90	[0.84; 0.94]	0.0%	0.4%
Zhu Z	1271	1440	0.88	[0.86; 0.90]	0.3%	0.5%
Zhu Z	1555	2269	0.69	[0.67; 0.70]	0.4%	0.5%
Common effect model		88370	0.85	[0.85; 0.86]	15.7%	−−
Random effects model			0.74	[0.67; 0.80]	−−	23.1%
Heterogeneity: $I^2 = 100\%$, $\tau^2 = 0.0672$, $p = 0$						
**Common effect model**		**563485**	**0.80**	**[0.80; 0.80]**	**100.0%**	**−−**
**Random effects model**			**0.77**	**[0.74; 0.79]**	**−−**	**100.0%**
Heterogeneity: $I^2 = 100\%$, $\tau^2 = 0.0502$, $p = 0$						

0.2 0.4 0.6 0.8

Test for subgroup differences (random effects): $\chi^2_5 = 28.53$, df = 5 ($p < 0.01$)

Supplementary figure 10 The prevalence of serum 25(OH)D < 75 nmol/L by six WHO regions

Appendix 7: The prevalence of vitamin D deficiency by latitude

Study	Events	Total	Proportion	95%−CI	Weight (common)	Weight (random)
**latitude = 0-20°N**						
Beer RJ	129	6813	0.02	[0.02; 0.02]	0.1%	0.4%
Beer RJ	194	6470	0.03	[0.03; 0.03]	0.1%	0.4%
Beer RJ	265	7170	0.04	[0.03; 0.04]	0.1%	0.4%
Beer RJ	395	16454	0.02	[0.02; 0.03]	0.2%	0.4%
Chin KY	2	150	0.01	[0.00; 0.05]	0.0%	0.4%
Gebreegziabher T	29	196	0.15	[0.10; 0.21]	0.0%	0.4%
Jayatissa R	333	2525	0.13	[0.12; 0.15]	0.0%	0.4%
Lin L	1	477	0.00	[0.00; 0.01]	0.0%	0.4%
Lin L	1	860	0.00	[0.00; 0.01]	0.0%	0.4%
Lin L	1	353	0.00	[0.00; 0.02]	0.0%	0.4%
Mechenro J	4	48	0.08	[0.02; 0.20]	0.0%	0.4%
Mechenro J	41	101	0.41	[0.31; 0.51]	0.0%	0.4%
Mechenro J	46	275	0.17	[0.13; 0.22]	0.0%	0.4%
Meyer HE	7	196	0.04	[0.01; 0.07]	0.0%	0.4%
Paul TV	15	150	0.10	[0.06; 0.16]	0.0%	0.4%
Shetty S	18	252	0.07	[0.04; 0.11]	0.0%	0.4%
Shivane VK	220	1137	0.19	[0.17; 0.22]	0.0%	0.4%
Smith G	7	128	0.05	[0.02; 0.11]	0.0%	0.4%
Smith G	13	495	0.03	[0.01; 0.04]	0.0%	0.4%
Common effect model		44250	0.03	[0.03; 0.03]	0.6%	−−
Random effects model			0.06	[0.03; 0.10]	−−	7.2%
Heterogeneity: $I^2 = 98\%$, $\tau^2 = 0.0253$, $p < 0.01$						
**latitude = 20-40°N**						
Abdulrahman MA	13	63	0.21	[0.11; 0.33]	0.0%	0.4%
Abdulrahman MA	16	50	0.32	[0.20; 0.47]	0.0%	0.4%
Abdulrahman MA	50	278	0.18	[0.14; 0.23]	0.0%	0.4%
Abiaka C	80	206	0.39	[0.32; 0.46]	0.0%	0.4%
Al Hayek S	33	344	0.10	[0.07; 0.13]	0.0%	0.4%
Al Shaikh A	1687	3613	0.47	[0.45; 0.48]	0.0%	0.4%
Al Shaikh AM	61	204	0.30	[0.24; 0.37]	0.0%	0.4%
Al Shaikh AM	899	1906	0.47	[0.45; 0.49]	0.0%	0.4%
Al−Daghri NM	150	561	0.27	[0.23; 0.31]	0.0%	0.4%
Al−Daghri NM	235	830	0.28	[0.25; 0.32]	0.0%	0.4%
Al−Daghri NM	276	808	0.34	[0.31; 0.38]	0.0%	0.4%
Al−Daghri NM	718	2225	0.32	[0.30; 0.34]	0.0%	0.4%
Al−Daghri NM	1082	4247	0.25	[0.24; 0.27]	0.1%	0.4%
Al−Daghri NM	1083	3111	0.35	[0.33; 0.37]	0.0%	0.4%
Al−Kindi MK	51	100	0.51	[0.41; 0.61]	0.0%	0.4%
AlQuaiz AM	729	2832	0.26	[0.24; 0.27]	0.0%	0.4%
Al−Saleh Y	718	2226	0.32	[0.30; 0.34]	0.0%	0.4%
Al−Taiar A	559	1416	0.39	[0.37; 0.42]	0.0%	0.4%
Alyahya K	169	232	0.73	[0.67; 0.78]	0.0%	0.4%
Alyahya KO	88	104	0.85	[0.76; 0.91]	0.0%	0.4%
Arabi A	182	466	0.39	[0.35; 0.44]	0.0%	0.4%
Ardawi MS	264	843	0.31	[0.28; 0.35]	0.0%	0.4%
Ardawi MS	534	1172	0.46	[0.43; 0.48]	0.0%	0.4%
Arya V	44	92	0.48	[0.37; 0.58]	0.0%	0.4%
Bachhel R	60	150	0.40	[0.32; 0.48]	0.0%	0.4%
Bezrati I	92	225	0.41	[0.34; 0.48]	0.0%	0.4%
Bhatt SP	9	137	0.07	[0.03; 0.12]	0.0%	0.4%
Capuano R	137	1200	0.11	[0.10; 0.13]	0.0%	0.4%
Chakrabarty S	1166	5024	0.23	[0.22; 0.24]	0.1%	0.4%
Chakrabarty S	1608	6798	0.24	[0.23; 0.25]	0.1%	0.4%
Chen J	731	6014	0.12	[0.11; 0.13]	0.1%	0.4%
Choi HR	110	651	0.17	[0.14; 0.20]	0.0%	0.4%
Djennane M	35	435	0.08	[0.06; 0.11]	0.0%	0.4%
Drali O	93	621	0.15	[0.12; 0.18]	0.0%	0.4%
Duarte C	706	3092	0.23	[0.21; 0.24]	0.0%	0.4%
Fang F	139	1814	0.08	[0.06; 0.09]	0.0%	0.4%
Golbahar J	247	500	0.49	[0.45; 0.54]	0.0%	0.4%
Goswami R	559	642	0.87	[0.84; 0.90]	0.0%	0.4%
Hashemipour S	812	1210	0.67	[0.64; 0.70]	0.0%	0.4%
Hatun S	19	89	0.21	[0.13; 0.31]	0.0%	0.4%
Herrick KA	7	1438	0.00	[0.00; 0.01]	0.0%	0.4%
Herrick KA	29	2060	0.01	[0.01; 0.02]	0.0%	0.4%
Herrick KA	94	3267	0.03	[0.02; 0.04]	0.0%	0.4%
Herrick KA	113	2355	0.05	[0.04; 0.06]	0.0%	0.4%
Herrick KA	199	3496	0.06	[0.05; 0.07]	0.0%	0.4%
Herrick KA	271	3564	0.08	[0.07; 0.09]	0.0%	0.4%
Hovsepian S	231	1111	0.21	[0.18; 0.23]	0.0%	0.4%
Hutchings N	157	1206	0.13	[0.11; 0.15]	0.0%	0.4%
Islam MZ	27	189	0.14	[0.10; 0.20]	0.0%	0.4%
Islam MZ	31	244	0.13	[0.09; 0.18]	0.0%	0.4%
Jiang W	1197	14302	0.08	[0.08; 0.09]	0.2%	0.4%
Joukar F	1189	5096	0.23	[0.22; 0.25]	0.1%	0.4%
Kaddam IM	912	2104	0.43	[0.41; 0.45]	0.0%	0.4%
Kaddam IM	1979	4035	0.49	[0.47; 0.51]	0.1%	0.4%
Kim SY	10652	157211	0.07	[0.07; 0.07]	2.1%	0.4%
Li H	1020	4989	0.20	[0.19; 0.22]	0.1%	0.4%
Li H	1020	5707	0.18	[0.17; 0.19]	0.1%	0.4%
Mallah EM	8	300	0.03	[0.01; 0.05]	0.0%	0.4%
Manios Y	122	2353	0.05	[0.04; 0.06]	0.0%	0.4%
Mansbach JM	17	1799	0.01	[0.01; 0.02]	0.0%	0.4%
Mansbach JM	27	2759	0.01	[0.01; 0.01]	0.0%	0.4%
Masoud MS	117	170	0.69	[0.61; 0.76]	0.0%	0.4%
Meddeb N	66	389	0.17	[0.13; 0.21]	0.0%	0.4%
Nakamura K	22	600	0.04	[0.02; 0.05]	0.0%	0.4%
Nakhaee S	115	400	0.29	[0.24; 0.33]	0.0%	0.4%
Niafar M	115	300	0.38	[0.33; 0.44]	0.0%	0.4%
Nichols EK	180	915	0.20	[0.17; 0.22]	0.0%	0.4%
Nikooyeh B	374	667	0.56	[0.52; 0.60]	0.0%	0.4%
Nikooyeh B	974	1406	0.69	[0.67; 0.72]	0.0%	0.4%
Öztürk ZA	150	1161	0.13	[0.11; 0.15]	0.0%	0.4%
Pérez−Llamas F	28	86	0.33	[0.23; 0.44]	0.0%	0.4%
Qorbani M	276	2596	0.11	[0.09; 0.12]	0.0%	0.4%
Rodríguez−Rodríguez E	8	102	0.08	[0.03; 0.15]	0.0%	0.4%
Saeed BQ	48	287	0.17	[0.13; 0.22]	0.0%	0.4%
Saki F	63	477	0.13	[0.10; 0.17]	0.0%	0.4%
Saliba W	891	6518	0.14	[0.13; 0.15]	0.1%	0.4%
Saliba W	27676	192240	0.14	[0.14; 0.15]	2.5%	0.4%
Santos A	594	1500	0.40	[0.37; 0.42]	0.0%	0.4%
Sharawat IK	29	100	0.29	[0.20; 0.39]	0.0%	0.4%
Sulimani RA	645	1618	0.40	[0.37; 0.42]	0.0%	0.4%
Tseng M	66	194	0.34	[0.27; 0.41]	0.0%	0.4%
Yan X	81	302	0.27	[0.22; 0.32]	0.0%	0.4%
Yu S	128	2173	0.06	[0.05; 0.07]	0.0%	0.4%
Zhang FF	348	960	0.36	[0.33; 0.39]	0.0%	0.4%
Zhu Z	6	183	0.03	[0.01; 0.07]	0.0%	0.4%
Zhu Z	26	2269	0.01	[0.01; 0.02]	0.0%	0.4%
Zhu Z	29	1440	0.02	[0.01; 0.03]	0.0%	0.4%
Common effect model		495169	0.13	[0.13; 0.13]	6.5%	−−
Random effects model			0.23	[0.19; 0.28]	−−	33.2%
Heterogeneity: $I^2 = 100\%$, $\tau^2 = 0.0593$, $p = 0$						
**latitude = 40-60°N**						
Alkerwi A	207	1335	0.16	[0.14; 0.18]	0.0%	0.4%
Andersen R	8	52	0.15	[0.07; 0.28]	0.0%	0.4%
Andersen R	13	54	0.24	[0.13; 0.38]	0.0%	0.4%
Andersen R	17	221	0.08	[0.05; 0.12]	0.0%	0.4%
Andersen R	30	37	0.81	[0.65; 0.92]	0.0%	0.3%
Andersen R	37	199	0.19	[0.13; 0.25]	0.0%	0.4%
Andersen R	158	219	0.72	[0.66; 0.78]	0.0%	0.4%
Arnljots R	224	545	0.41	[0.37; 0.45]	0.0%	0.4%
Asakura K	40	107	0.37	[0.28; 0.47]	0.0%	0.4%
Aspell N	1423	6004	0.24	[0.23; 0.25]	0.1%	0.4%
Aucoin M	78	756	0.10	[0.08; 0.13]	0.0%	0.4%
Aucoin M	96	461	0.21	[0.17; 0.25]	0.0%	0.4%
Bater J	5692	9595	0.59	[0.58; 0.60]	0.1%	0.4%
Borissova AM	430	2016	0.21	[0.20; 0.23]	0.0%	0.4%
Cashman KD	76	1132	0.07	[0.05; 0.08]	0.0%	0.4%
Ceccarelli M	209	2140	0.10	[0.09; 0.11]	0.0%	0.4%
Chao YS	13	1493	0.01	[0.00; 0.01]	0.0%	0.4%
Chirita−Emandi A	265	6631	0.04	[0.04; 0.04]	0.1%	0.4%
Chlebna−Sokó.. D	145	720	0.20	[0.17; 0.23]	0.0%	0.4%
Cougnard−Grégoire A	190	697	0.27	[0.24; 0.31]	0.0%	0.4%
Crowe FL	223186	755174	0.30	[0.29; 0.30]	9.9%	0.4%
Crowe FL	299730	993895	0.30	[0.30; 0.30]	13.0%	0.4%
Crowe FL	300265	901352	0.33	[0.33; 0.33]	11.8%	0.4%
Crowe FL	443448	1180086	0.38	[0.37; 0.38]	15.5%	0.4%
Crowe FL	531681	1316390	0.40	[0.40; 0.40]	17.2%	0.4%
Crowe FL	536113	1269812	0.42	[0.42; 0.42]	16.6%	0.4%
El Hayek J	3	508	0.01	[0.00; 0.02]	0.0%	0.4%
Fernández Bustillo JM	9	153	0.06	[0.03; 0.11]	0.0%	0.4%
Ganmaa D	333	420	0.79	[0.75; 0.83]	0.0%	0.4%
Ginter JK	3	224	0.01	[0.00; 0.04]	0.0%	0.4%
Glatt DU	3	49	0.06	[0.01; 0.17]	0.0%	0.4%
Greene−Finestone LS	8	312	0.03	[0.01; 0.05]	0.0%	0.4%
Greene−Finestone LS	18	777	0.02	[0.01; 0.04]	0.0%	0.4%
Greene−Finestone LS	19	823	0.02	[0.01; 0.04]	0.0%	0.4%
Griffin TP	2047	15319	0.13	[0.13; 0.14]	0.2%	0.4%
Gromova O	371	1347	0.28	[0.25; 0.30]	0.0%	0.4%
Hansen L	79	527	0.15	[0.12; 0.18]	0.0%	0.4%
Hansen L	309	2565	0.12	[0.11; 0.13]	0.0%	0.4%
Hintzpeter B	1797	9001	0.20	[0.19; 0.21]	0.1%	0.4%
Hirani V	292	2070	0.14	[0.13; 0.16]	0.0%	0.4%
Hoge A	58	915	0.06	[0.05; 0.08]	0.0%	0.4%
Hribar M	31	125	0.25	[0.18; 0.33]	0.0%	0.4%
Hribar M	36	155	0.23	[0.17; 0.31]	0.0%	0.4%
Jääskeläinen T	17	4051	0.00	[0.00; 0.01]	0.1%	0.4%
Jolliffe DA	55	222	0.25	[0.19; 0.31]	0.0%	0.4%
Jorde R	157	2668	0.06	[0.05; 0.07]	0.0%	0.4%
Karagüzel G	75	746	0.10	[0.08; 0.12]	0.0%	0.4%
Karin Z	31	260	0.12	[0.08; 0.16]	0.0%	0.4%
Kunz C	63	327	0.19	[0.15; 0.24]	0.0%	0.4%
Kunz C	106	528	0.20	[0.17; 0.24]	0.0%	0.4%
Kunz C	111	456	0.24	[0.20; 0.29]	0.0%	0.4%
Kunz C	112	520	0.22	[0.18; 0.25]	0.0%	0.4%
Laird E	728	5356	0.14	[0.13; 0.15]	0.1%	0.4%
Langlois K	218	5306	0.04	[0.04; 0.05]	0.1%	0.4%
Lardner E	14	143	0.10	[0.05; 0.16]	0.0%	0.4%
Le Goaziou MF	105	196	0.54	[0.46; 0.61]	0.0%	0.4%
Lin LY	60687	448601	0.14	[0.13; 0.14]	5.9%	0.4%
Madsen KH	4	415	0.01	[0.00; 0.02]	0.0%	0.4%
Maguire JL	1	91	0.01	[0.00; 0.06]	0.0%	0.4%
Man PW	40	416	0.10	[0.07; 0.13]	0.0%	0.4%
Matheï C	129	367	0.35	[0.30; 0.40]	0.0%	0.4%
Mitchell DM	45	634	0.07	[0.05; 0.09]	0.0%	0.4%
Moreno−Reyes R	135	401	0.34	[0.29; 0.39]	0.0%	0.4%
Nälsén C	9	268	0.03	[0.02; 0.06]	0.0%	0.4%
Nälsén C	10	206	0.05	[0.02; 0.09]	0.0%	0.4%
NHANES (2015...2016)	37	1777	0.02	[0.01; 0.03]	0.0%	0.4%
NHANES(2015...2016)	23	1231	0.02	[0.01; 0.03]	0.0%	0.4%
NHANES(2015...2016)	26	2618	0.01	[0.01; 0.01]	0.0%	0.4%
NHANES(2015...2016)	85	2413	0.04	[0.03; 0.04]	0.0%	0.4%
NHANES(2017...2018)	12	1321	0.01	[0.00; 0.02]	0.0%	0.4%
NHANES(2017...2018)	37	2184	0.02	[0.01; 0.02]	0.0%	0.4%
NHANES(2017...2018)	43	1810	0.02	[0.02; 0.03]	0.0%	0.4%
NHANES(2017...2018)	80	2094	0.04	[0.03; 0.05]	0.0%	0.4%
Ní Chaoimh C	12	741	0.02	[0.01; 0.03]	0.0%	0.4%
Orwoll E	47	1606	0.03	[0.02; 0.04]	0.0%	0.4%
Patel JV	1283	1904	0.67	[0.65; 0.69]	0.0%	0.4%
Perna L	844	5386	0.16	[0.15; 0.17]	0.1%	0.4%
Rabenberg M	540	1816	0.30	[0.28; 0.32]	0.0%	0.4%
Rabenberg M	756	2456	0.31	[0.29; 0.33]	0.0%	0.4%
Rabenberg M	804	2723	0.30	[0.28; 0.31]	0.0%	0.4%
Rabufetti A	24	1045	0.02	[0.01; 0.03]	0.0%	0.4%
Raposo L	188	500	0.38	[0.33; 0.42]	0.0%	0.4%
Riverin B	3	52	0.06	[0.01; 0.16]	0.0%	0.4%
Riverin B	54	944	0.06	[0.04; 0.07]	0.0%	0.4%
Sarafin K	839	11336	0.07	[0.07; 0.08]	0.1%	0.4%
Schramm S	660	4149	0.16	[0.15; 0.17]	0.1%	0.4%
Science M	4	743	0.01	[0.00; 0.01]	0.0%	0.4%
Shchubelka K	211	1639	0.13	[0.11; 0.15]	0.0%	0.4%
Sioen I	18	357	0.05	[0.03; 0.08]	0.0%	0.4%
Smith N	19	148	0.13	[0.08; 0.19]	0.0%	0.4%
Sochorová L	101	419	0.24	[0.20; 0.28]	0.0%	0.4%
Sokolovic S	93	450	0.21	[0.17; 0.25]	0.0%	0.4%
Sokolovic S	185	645	0.29	[0.25; 0.32]	0.0%	0.4%
Sokolovic S	235	735	0.32	[0.29; 0.35]	0.0%	0.4%
Souberbielle JC	56	892	0.06	[0.05; 0.08]	0.0%	0.4%
Thuesen B	848	6146	0.14	[0.13; 0.15]	0.1%	0.4%
Tolppanen AM	124	7560	0.02	[0.01; 0.02]	0.1%	0.4%
Tønnesen R	135	700	0.19	[0.16; 0.22]	0.0%	0.4%
Uush T	222	524	0.42	[0.38; 0.47]	0.0%	0.4%
Uush T	457	876	0.52	[0.49; 0.56]	0.0%	0.4%
Voortman T	258	4167	0.06	[0.05; 0.07]	0.1%	0.4%
Wyskida M	355	3472	0.10	[0.09; 0.11]	0.0%	0.4%
Yousef S	5	267	0.02	[0.01; 0.04]	0.0%	0.4%
Yousef S	38	932	0.04	[0.03; 0.06]	0.0%	0.4%
Yousef S	63	1361	0.05	[0.04; 0.06]	0.0%	0.4%
Yousef S	90	858	0.10	[0.09; 0.13]	0.0%	0.4%
Yousef S	988	8161	0.12	[0.11; 0.13]	0.1%	0.4%
Zgaga L	772	2235	0.35	[0.33; 0.37]	0.0%	0.4%
Common effect model		7051984	0.34	[0.34; 0.34]	92.4%	−−
Random effects model			0.15	[0.12; 0.18]	−−	41.3%
Heterogeneity: $I^2 = 100\%$, $\tau^2 = 0.0515$, $p = 0$						
**latitude = 60-80°N**						
Bjarnadottir A	5	158	0.03	[0.01; 0.07]	0.0%	0.4%
Courraud J	31	177	0.18	[0.12; 0.24]	0.0%	0.4%
Dalgård C	124	669	0.19	[0.16; 0.22]	0.0%	0.4%
El Hayek J	39	282	0.14	[0.10; 0.18]	0.0%	0.4%
Granlund L	26	216	0.12	[0.08; 0.17]	0.0%	0.4%
Ikonen H	92	3650	0.03	[0.02; 0.03]	0.0%	0.4%
Nielsen NO	525	2877	0.18	[0.17; 0.20]	0.0%	0.4%
Oberg J	147	890	0.17	[0.14; 0.19]	0.0%	0.4%
Petrenya N	31	4465	0.01	[0.00; 0.01]	0.1%	0.4%
Samefors M	48	333	0.14	[0.11; 0.19]	0.0%	0.4%
Common effect model		13717	0.06	[0.05; 0.06]	0.2%	−−
Random effects model			0.10	[0.06; 0.16]	−−	3.8%
Heterogeneity: $I^2 = 99\%$, $\tau^2 = 0.0178$, $p < 0.01$						
**latitude = 0-20°S**						
Lima−Costa MF	37	2264	0.02	[0.01; 0.02]	0.0%	0.4%
Maldonado G	3	269	0.01	[0.00; 0.03]	0.0%	0.4%
Common effect model		2533	0.02	[0.01; 0.02]	0.0%	−−
Random effects model			0.02	[0.01; 0.02]	−−	0.8%
Heterogeneity: $I^2 = 0\%$, $\tau^2 = 0$, $p = 0.64$						
**latitude = 20-40°S**						
Black LJ	16	331	0.05	[0.03; 0.08]	0.0%	0.4%
Black LJ	23	490	0.05	[0.03; 0.07]	0.0%	0.4%
Black LJ	26	730	0.04	[0.02; 0.05]	0.0%	0.4%
Black LJ	30	648	0.05	[0.03; 0.07]	0.0%	0.4%
Black LJ	43	652	0.07	[0.05; 0.09]	0.0%	0.4%
Bolland MJ	2	378	0.01	[0.00; 0.02]	0.0%	0.4%
Brinkmann K	67	108	0.62	[0.52; 0.71]	0.0%	0.4%
Cairncross CT	86	1329	0.06	[0.05; 0.08]	0.0%	0.4%
Daly RM	46	1393	0.03	[0.02; 0.04]	0.0%	0.4%
Daly RM	66	810	0.08	[0.06; 0.10]	0.0%	0.4%
Daly RM	76	1564	0.05	[0.04; 0.06]	0.0%	0.4%
Daly RM	77	2011	0.04	[0.03; 0.05]	0.0%	0.4%
Daly RM	108	2556	0.04	[0.03; 0.05]	0.0%	0.4%
Daly RM	111	2884	0.04	[0.03; 0.05]	0.0%	0.4%
Gill TK	21	2413	0.01	[0.01; 0.01]	0.0%	0.4%
Hirani V	27	174	0.16	[0.10; 0.22]	0.0%	0.4%
Hirani V	30	304	0.10	[0.07; 0.14]	0.0%	0.4%
Hirani V	45	522	0.09	[0.06; 0.11]	0.0%	0.4%
Hirani V	57	659	0.09	[0.07; 0.11]	0.0%	0.4%
Lategan R	1	339	0.00	[0.00; 0.02]	0.0%	0.4%
Lucas JA	92	1606	0.06	[0.05; 0.07]	0.0%	0.4%
Oliveri B	35	339	0.10	[0.07; 0.14]	0.0%	0.4%
Robinson PJ	37	267	0.14	[0.10; 0.19]	0.0%	0.4%
Robinson PJ	74	640	0.12	[0.09; 0.14]	0.0%	0.4%
Saraiva GL	33	214	0.15	[0.11; 0.21]	0.0%	0.4%
Scalco R	53	98	0.54	[0.44; 0.64]	0.0%	0.4%
Skull SA	61	116	0.53	[0.43; 0.62]	0.0%	0.4%
Solis−Urra P	181	686	0.26	[0.23; 0.30]	0.0%	0.4%
Solis−Urra P	204	1245	0.16	[0.14; 0.19]	0.0%	0.4%
Tran B	9	134	0.07	[0.03; 0.12]	0.0%	0.4%
Tran B	11	148	0.07	[0.04; 0.13]	0.0%	0.4%
Tran B	11	148	0.07	[0.04; 0.13]	0.0%	0.4%
Tran B	15	131	0.11	[0.07; 0.18]	0.0%	0.4%
Tran B	20	92	0.22	[0.14; 0.32]	0.0%	0.4%
von Hurst PR	98	228	0.43	[0.36; 0.50]	0.0%	0.4%
Zhou SJ	9	221	0.04	[0.02; 0.08]	0.0%	0.4%
Common effect model		26608	0.06	[0.06; 0.06]	0.3%	−−
Random effects model			0.10	[0.07; 0.15]	−−	13.7%
Heterogeneity: $I^2 = 98\%$, $\tau^2 = 0.0382$, $p < 0.01$						
**Common effect model**		**7634261**	**0.32**	**[0.32; 0.32]**	**100.0%**	**−−**
**Random effects model**			**0.16**	**[0.14; 0.18]**	**−−**	**100.0%**
Heterogeneity: $I^2 = 100\%$ $\tau^2 = 0.0547$ $p = 0$						

Test for subgroup differences (random effects): $\chi^2_5 = 287.20$, df = 5 ($p < 0.01$)

Supplementary figure 11 The prevalence of serum 25(OH)D < 30 nmol/L by latitude

Appendix 7: The prevalence of vitamin D deficiency by latitude

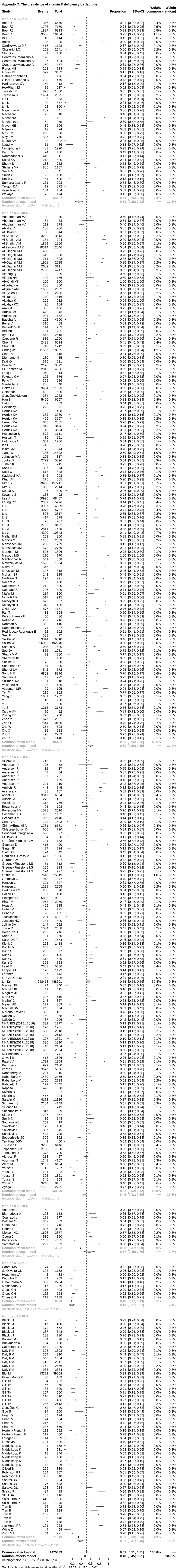

Supplementary figure 12 The prevalence of serum 25(OH)D < 50 nmol/L by latitude

Appendix 7: The prevalence of vitamin D deficiency by latitude

Study	Events	Total	Proportion	95%−CI	Weight (common)	Weight (random)
**latitude = 0-20°N**						
Chailurkit LO	1194	2641	0.45	[0.43; 0.47]	0.5%	0.5%
Contreras−Manzano A	324	404	0.80	[0.76; 0.84]	0.1%	0.5%
Contreras−Manzano A	287	381	0.75	[0.71; 0.80]	0.1%	0.5%
Contreras−Manzano A	367	477	0.77	[0.73; 0.81]	0.1%	0.5%
Flores ME	520	713	0.73	[0.70; 0.76]	0.1%	0.5%
Flores ME	2612	3482	0.75	[0.74; 0.76]	0.6%	0.5%
Gilbert−Diamond D	430	479	0.90	[0.87; 0.92]	0.1%	0.5%
Harinarayan CV	328	913	0.36	[0.33; 0.39]	0.2%	0.5%
Ho−Pham LT	240	637	0.38	[0.34; 0.42]	0.1%	0.5%
Jayashri R	1360	1500	0.91	[0.89; 0.92]	0.3%	0.5%
Lin L	61	353	0.17	[0.13; 0.22]	0.1%	0.5%
Lin L	161	477	0.34	[0.30; 0.38]	0.1%	0.5%
Lin L	173	860	0.20	[0.17; 0.23]	0.2%	0.5%
Miljkovic I	114	424	0.27	[0.23; 0.31]	0.1%	0.5%
Mutua AM	113	302	0.37	[0.32; 0.43]	0.1%	0.5%
Naqvi A	55	86	0.64	[0.53; 0.74]	0.0%	0.4%
Shivane VK	1055	1137	0.93	[0.91; 0.94]	0.2%	0.5%
Smith G	4	41	0.10	[0.03; 0.23]	0.0%	0.4%
Smith G	54	128	0.42	[0.34; 0.51]	0.0%	0.4%
Smith G	185	495	0.37	[0.33; 0.42]	0.1%	0.5%
Tangoh DA	96	372	0.26	[0.21; 0.31]	0.1%	0.5%
**Common effect model**		16302	0.61	[0.60; 0.62]	2.9%	−−
**Random effects model**			0.53	[0.40; 0.65]	−−	9.5%
Heterogeneity: I^2 = 100%, τ^2 = 0.0858, p = 0						
**latitude = 20-40°N**						
Abdulrahman MA	40	50	0.80	[0.66; 0.90]	0.0%	0.4%
Abdulrahman MA	45	63	0.71	[0.59; 0.82]	0.0%	0.4%
Abdulrahman MA	161	278	0.58	[0.52; 0.64]	0.0%	0.5%
Abiaka C	202	206	0.98	[0.95; 0.99]	0.0%	0.4%
Abu−Samak MS	338	371	0.91	[0.88; 0.94]	0.1%	0.5%
Al Hayek S	214	344	0.62	[0.57; 0.67]	0.1%	0.5%
Al Zarooni AAR	11765	12346	0.95	[0.95; 0.96]	2.2%	0.5%
Aleteng Q	1659	1829	0.91	[0.89; 0.92]	0.3%	0.5%
AlQuaiz AM	2215	2832	0.78	[0.77; 0.80]	0.5%	0.5%
Al−Taiar A	1365	1416	0.96	[0.95; 0.97]	0.3%	0.5%
Arabi A	427	466	0.92	[0.89; 0.94]	0.1%	0.5%
Ardawi MS	1034	1172	0.88	[0.86; 0.90]	0.2%	0.5%
Ardawi MS	510	843	0.60	[0.57; 0.64]	0.2%	0.5%
Bachhel R	135	150	0.90	[0.84; 0.94]	0.0%	0.4%
Batieha A	991	4590	0.22	[0.20; 0.23]	0.8%	0.5%
Bhatt SP	120	137	0.88	[0.81; 0.93]	0.0%	0.4%
Capuano R	1053	1200	0.88	[0.86; 0.90]	0.2%	0.5%
Choi HR	626	651	0.96	[0.94; 0.97]	0.1%	0.5%
Chung IH	1177	1212	0.97	[0.96; 0.98]	0.2%	0.5%
Chung JY	16973	18305	0.93	[0.92; 0.93]	3.3%	0.5%
Cinar N	114	118	0.97	[0.92; 0.99]	0.0%	0.4%
Djennane M	254	435	0.58	[0.54; 0.63]	0.1%	0.5%
Drali O	528	621	0.85	[0.82; 0.88]	0.1%	0.5%
Duarte C	3000	3092	0.97	[0.96; 0.98]	0.6%	0.5%
El−Khateeb M	3548	4056	0.87	[0.86; 0.88]	0.7%	0.5%
Feketea GM	249	376	0.66	[0.61; 0.71]	0.1%	0.5%
Feng X	572	686	0.83	[0.80; 0.86]	0.1%	0.5%
González−Molero I	968	1262	0.77	[0.74; 0.79]	0.2%	0.5%
Hekimsoy Z	347	391	0.89	[0.85; 0.92]	0.1%	0.5%
Hovsepian S	725	1111	0.65	[0.62; 0.68]	0.2%	0.5%
Isa H	496	531	0.93	[0.91; 0.95]	0.1%	0.5%
Jiang W	11874	14302	0.83	[0.82; 0.84]	2.5%	0.5%
Kapil U	356	374	0.95	[0.93; 0.97]	0.1%	0.5%
Kapil U	767	848	0.90	[0.88; 0.92]	0.2%	0.5%
Kaykhaei MA	940	993	0.95	[0.93; 0.96]	0.2%	0.5%
Khan AH	292	305	0.96	[0.93; 0.98]	0.1%	0.5%
Kouda K	198	400	0.50	[0.44; 0.55]	0.1%	0.5%
Koyama S	486	492	0.99	[0.97; 1.00]	0.1%	0.5%
Kremer R	53	90	0.59	[0.48; 0.69]	0.0%	0.4%
Leung RY	4752	5276	0.90	[0.89; 0.91]	0.9%	0.5%
Li L	1683	2317	0.73	[0.71; 0.74]	0.4%	0.5%
Liu X	5785	7485	0.77	[0.76; 0.78]	1.3%	0.5%
Liu X	6041	8182	0.74	[0.73; 0.75]	1.5%	0.5%
Liu X	7985	10343	0.77	[0.76; 0.78]	1.8%	0.5%
Mallah EM	292	300	0.97	[0.95; 0.99]	0.1%	0.5%
Manios Y	2266	2353	0.96	[0.95; 0.97]	0.4%	0.5%
Mansbach JM	1131	1799	0.63	[0.61; 0.65]	0.3%	0.5%
Mansbach JM	2013	2759	0.73	[0.71; 0.75]	0.5%	0.5%
Mehboobali N	810	858	0.94	[0.93; 0.96]	0.2%	0.5%
Nadeem S	213	221	0.96	[0.93; 0.98]	0.0%	0.4%
Naeem Z	121	180	0.67	[0.60; 0.74]	0.0%	0.4%
Öztürk ZA	1102	1161	0.95	[0.93; 0.96]	0.2%	0.5%
Pan T	137	194	0.71	[0.64; 0.77]	0.0%	0.4%
Qorbani M	1846	2596	0.71	[0.69; 0.73]	0.5%	0.5%
Rafraf M	215	216	1.00	[0.97; 1.00]	0.0%	0.4%
Rahman A	393	410	0.96	[0.93; 0.98]	0.1%	0.5%
Ramakrishnan S	117	237	0.49	[0.43; 0.56]	0.0%	0.5%
Saeed BQ	244	287	0.85	[0.80; 0.89]	0.1%	0.5%
Saki F	458	477	0.96	[0.94; 0.98]	0.1%	0.5%
Saliba W	5237	6518	0.80	[0.79; 0.81]	1.2%	0.5%
Saliba W	160952	192240	0.84	[0.84; 0.84]	34.2%	0.5%
Sheikh A	253	300	0.84	[0.80; 0.88]	0.1%	0.5%
Sherchand O	236	300	0.79	[0.74; 0.83]	0.1%	0.5%
Sherief LM	566	572	0.99	[0.98; 1.00]	0.1%	0.5%
Song HR	8720	8976	0.97	[0.97; 0.97]	1.6%	0.5%
Srimani S	167	222	0.75	[0.69; 0.81]	0.0%	0.4%
Tseng M	192	194	0.99	[0.96; 1.00]	0.0%	0.4%
Vallianou N	352	490	0.72	[0.68; 0.76]	0.1%	0.5%
Yang K	1893	1982	0.96	[0.95; 0.96]	0.4%	0.5%
Zhao Y	2523	2661	0.95	[0.94; 0.96]	0.5%	0.5%
Zhu W	227	508	0.45	[0.40; 0.49]	0.1%	0.5%
Zhu Z	164	183	0.90	[0.84; 0.94]	0.0%	0.4%
Zhu Z	1271	1440	0.88	[0.86; 0.90]	0.3%	0.5%
Zhu Z	1555	2269	0.69	[0.67; 0.70]	0.4%	0.5%
**Common effect model**		345450	0.85	[0.84; 0.85]	61.5%	−−
**Random effects model**			0.85	[0.81; 0.88]	−−	33.6%
Heterogeneity: I^2 = 100%, τ^2 = 0.0424, p = 0						
**latitude = 40-60°N**						
Alkerwi A	1107	1335	0.83	[0.81; 0.85]	0.2%	0.5%
Arnljots R	510	545	0.94	[0.91; 0.95]	0.1%	0.5%
Aucoin M	405	461	0.88	[0.85; 0.91]	0.1%	0.5%
Aucoin M	617	756	0.82	[0.79; 0.84]	0.1%	0.5%
Bettencourt A	155	198	0.78	[0.72; 0.84]	0.0%	0.4%
Bhattoa HP	109	206	0.53	[0.46; 0.60]	0.0%	0.4%
Ceccarelli M	1657	2140	0.77	[0.76; 0.79]	0.4%	0.5%
Chao YS	2896	6101	0.47	[0.46; 0.49]	1.1%	0.5%
Chirita−Emandi A	3926	6631	0.59	[0.58; 0.60]	1.2%	0.5%
El Hayek J	257	508	0.51	[0.46; 0.55]	0.1%	0.5%
Ganmaa D	419	420	1.00	[0.99; 1.00]	0.1%	0.5%
Ginter JK	87	224	0.39	[0.32; 0.46]	0.0%	0.5%
González−Gross M	817	1006	0.81	[0.79; 0.84]	0.2%	0.5%
Greene−Finestone LS	185	312	0.59	[0.54; 0.65]	0.1%	0.5%
Greene−Finestone LS	484	777	0.62	[0.59; 0.66]	0.1%	0.5%
Greene−Finestone LS	464	823	0.56	[0.53; 0.60]	0.1%	0.5%
Gromova O	1232	1347	0.91	[0.90; 0.93]	0.2%	0.5%
Hansen L	482	527	0.91	[0.89; 0.94]	0.1%	0.5%
Hansen L	2045	2565	0.80	[0.78; 0.81]	0.5%	0.5%
Hazell TJ	203	488	0.42	[0.37; 0.46]	0.1%	0.5%
Hintzpeter B	7911	9001	0.88	[0.87; 0.89]	1.6%	0.5%
Hirani V	1788	2070	0.86	[0.85; 0.88]	0.4%	0.5%
Hoge A	719	915	0.79	[0.76; 0.81]	0.2%	0.5%
Hribar M	104	125	0.83	[0.75; 0.89]	0.0%	0.4%
Hribar M	131	155	0.85	[0.78; 0.90]	0.0%	0.4%
Jääskeläinen T	3068	4051	0.76	[0.74; 0.77]	0.7%	0.5%
Janssen HC	276	400	0.69	[0.64; 0.74]	0.1%	0.5%
Jolliffe DA	205	222	0.92	[0.88; 0.95]	0.0%	0.4%
Karagüzel G	350	746	0.47	[0.43; 0.51]	0.1%	0.5%
Karin Z	226	260	0.87	[0.82; 0.91]	0.0%	0.5%
Karonova T	1384	1664	0.83	[0.81; 0.85]	0.3%	0.5%
Klenk J	291	1418	0.21	[0.18; 0.23]	0.3%	0.5%
Kunz C	400	456	0.88	[0.84; 0.91]	0.1%	0.5%
Kunz C	469	528	0.89	[0.86; 0.91]	0.1%	0.5%
Kunz C	453	520	0.87	[0.84; 0.90]	0.1%	0.5%
Kunz C	241	327	0.74	[0.69; 0.78]	0.1%	0.5%
Langlois K	3428	5306	0.65	[0.63; 0.66]	0.9%	0.5%
Le Goaziou MF	188	196	0.96	[0.92; 0.98]	0.0%	0.4%
Madsen KH	197	340	0.58	[0.52; 0.63]	0.1%	0.5%
Madsen KH	211	415	0.51	[0.46; 0.56]	0.1%	0.5%
Maguire JL	75	91	0.82	[0.73; 0.90]	0.0%	0.4%
Man PW	332	416	0.80	[0.76; 0.84]	0.1%	0.5%
Matheï C	323	367	0.88	[0.84; 0.91]	0.1%	0.5%
Mitchell DM	407	634	0.64	[0.60; 0.68]	0.1%	0.5%
NHANES (2015...2016)	992	1777	0.56	[0.53; 0.58]	0.3%	0.5%
NHANES(2015...2016)	1730	2413	0.72	[0.70; 0.73]	0.4%	0.5%
NHANES(2015...2016)	474	1231	0.39	[0.36; 0.41]	0.2%	0.5%
NHANES(2015...2016)	1833	2618	0.70	[0.68; 0.72]	0.5%	0.5%
NHANES(2017...2018)	1533	2094	0.73	[0.71; 0.75]	0.4%	0.5%
NHANES(2017...2018)	1500	2184	0.69	[0.67; 0.71]	0.4%	0.5%
NHANES(2017...2018)	950	1810	0.52	[0.50; 0.55]	0.3%	0.5%
NHANES(2017...2018)	439	1321	0.33	[0.31; 0.36]	0.2%	0.5%
Ní Chaoimh C	530	741	0.72	[0.68; 0.75]	0.1%	0.5%
Orwoll E	1142	1606	0.71	[0.69; 0.73]	0.3%	0.5%
Penrose K	1685	2160	0.78	[0.76; 0.80]	0.4%	0.5%
Rabenberg M	1687	1816	0.93	[0.92; 0.94]	0.3%	0.5%
Rabenberg M	2465	2723	0.91	[0.89; 0.92]	0.5%	0.5%
Rabenberg M	2054	2456	0.84	[0.82; 0.85]	0.4%	0.5%
Riverin B	834	944	0.88	[0.86; 0.90]	0.2%	0.5%
Riverin B	41	52	0.79	[0.65; 0.89]	0.0%	0.4%
Schramm S	3456	4149	0.83	[0.82; 0.84]	0.7%	0.5%
Science M	565	743	0.76	[0.73; 0.79]	0.1%	0.5%
Sioen I	350	357	0.98	[0.96; 0.99]	0.1%	0.5%
Sokolovic S	659	735	0.90	[0.87; 0.92]	0.1%	0.5%
Sokolovic S	513	645	0.80	[0.76; 0.83]	0.1%	0.5%
Sokolovic S	263	450	0.58	[0.54; 0.63]	0.1%	0.5%
Souberbielle JC	716	892	0.80	[0.78; 0.83]	0.2%	0.5%
Ten Haaf DSM	108	450	0.24	[0.20; 0.28]	0.1%	0.5%
Tolppanen AM	5631	7560	0.74	[0.73; 0.75]	1.3%	0.5%
Vierucci F	351	427	0.82	[0.78; 0.86]	0.1%	0.5%
Voortman T	3020	4167	0.72	[0.71; 0.74]	0.7%	0.5%
Wyskida M	3002	3472	0.86	[0.85; 0.88]	0.6%	0.5%
Yousef S	179	267	0.67	[0.61; 0.73]	0.0%	0.5%
Yousef S	723	858	0.84	[0.82; 0.87]	0.2%	0.5%
Yousef S	727	932	0.78	[0.75; 0.81]	0.2%	0.5%
Yousef S	799	1361	0.59	[0.56; 0.61]	0.2%	0.5%
Yousef S	6386	8161	0.78	[0.77; 0.79]	1.5%	0.5%
**Common effect model**		121565	0.74	[0.74; 0.74]	21.6%	−−
**Random effects model**			0.75	[0.71; 0.78]	−−	35.1%
Heterogeneity: I^2 = 99%, τ^2 = 0.0410, p = 0						
**latitude = 60-80°N**						
El Hayek J	178	282	0.63	[0.57; 0.69]	0.1%	0.5%
Granlund L	208	216	0.96	[0.93; 0.98]	0.0%	0.4%
Ikonen H	2597	3650	0.71	[0.70; 0.73]	0.6%	0.5%
Oberg J	780	890	0.88	[0.85; 0.90]	0.2%	0.5%
Petrenya N	3313	4465	0.74	[0.73; 0.75]	0.8%	0.5%
Samefors M	318	333	0.95	[0.93; 0.97]	0.1%	0.5%
**Common effect model**		9836	0.76	[0.75; 0.77]	1.8%	−−
**Random effects model**			0.83	[0.71; 0.93]	−−	2.7%
Heterogeneity: I^2 = 99%, τ^2 = 0.0355, p < 0.01						
**latitude = 0-20°S**						
Cabral MA	156	234	0.67	[0.60; 0.73]	0.0%	0.5%
de Oliveira CL	777	1152	0.67	[0.65; 0.70]	0.2%	0.5%
Kagotho E	152	253	0.60	[0.54; 0.66]	0.0%	0.5%
Lima−Costa MF	1404	2264	0.62	[0.60; 0.64]	0.4%	0.5%
Maldonado G	188	269	0.70	[0.64; 0.75]	0.0%	0.5%
Orces CH	510	724	0.70	[0.67; 0.74]	0.1%	0.5%
Orces CH	277	403	0.69	[0.64; 0.73]	0.1%	0.5%
Orces CH	750	1158	0.65	[0.62; 0.68]	0.2%	0.5%
**Common effect model**		6457	0.65	[0.64; 0.66]	1.1%	−−
**Random effects model**			0.66	[0.64; 0.69]	−−	3.6%
Heterogeneity: I^2 = 76%, τ^2 = 0.0010, p < 0.01						
**latitude = 20-40°S**						
Black LJ	245	331	0.74	[0.69; 0.79]	0.1%	0.5%
Black LJ	347	490	0.71	[0.67; 0.75]	0.1%	0.5%
Black LJ	540	730	0.74	[0.71; 0.77]	0.1%	0.5%
Black LJ	450	648	0.69	[0.66; 0.73]	0.1%	0.5%
Black LJ	440	652	0.67	[0.64; 0.71]	0.1%	0.5%
Brinkmann K	107	108	0.99	[0.95; 1.00]	0.0%	0.4%
Cairncross CT	1183	1329	0.89	[0.87; 0.91]	0.2%	0.5%
Daly RM	673	810	0.83	[0.80; 0.86]	0.1%	0.5%
Daly RM	1235	1564	0.79	[0.77; 0.81]	0.3%	0.5%
Daly RM	1598	2011	0.79	[0.78; 0.81]	0.4%	0.5%
Daly RM	868	1393	0.62	[0.60; 0.65]	0.2%	0.5%
Daly RM	2202	2884	0.76	[0.75; 0.78]	0.5%	0.5%
Daly RM	1824	2556	0.71	[0.70; 0.73]	0.5%	0.5%
Eloi M	27576	39004	0.71	[0.70; 0.71]	6.9%	0.5%
Gill TK	165	263	0.63	[0.57; 0.69]	0.0%	0.5%
Gill TK	150	265	0.57	[0.50; 0.63]	0.0%	0.5%
Gill TK	245	390	0.63	[0.58; 0.68]	0.1%	0.5%
Gill TK	333	486	0.69	[0.64; 0.73]	0.1%	0.5%
Gill TK	346	510	0.68	[0.64; 0.72]	0.1%	0.5%
Gill TK	311	500	0.62	[0.58; 0.66]	0.1%	0.5%
Hirani V	133	174	0.76	[0.69; 0.83]	0.0%	0.4%
Hirani V	248	304	0.82	[0.77; 0.86]	0.1%	0.5%
Hirani V	421	522	0.81	[0.77; 0.84]	0.1%	0.5%
Hirani V	554	659	0.84	[0.81; 0.87]	0.1%	0.5%
Horton−French K	257	400	0.64	[0.59; 0.69]	0.1%	0.5%
Horton−French K	445	692	0.64	[0.61; 0.68]	0.1%	0.5%
Lopes JB	363	415	0.87	[0.84; 0.90]	0.1%	0.5%
Oliveri B	307	339	0.91	[0.87; 0.93]	0.1%	0.5%
Peters BS	84	136	0.62	[0.53; 0.70]	0.0%	0.4%
Robinson PJ	238	267	0.89	[0.85; 0.93]	0.0%	0.5%
Robinson PJ	562	640	0.88	[0.85; 0.90]	0.1%	0.5%
Santos BR	212	234	0.91	[0.86; 0.94]	0.0%	0.5%
Unger MD	473	603	0.78	[0.75; 0.82]	0.1%	0.5%
White Z	39	59	0.66	[0.53; 0.78]	0.0%	0.4%
**Common effect model**		62368	0.73	[0.72; 0.73]	11.1%	−−
**Random effects model**			0.76	[0.72; 0.80]	−−	15.4%
Heterogeneity: I^2 = 97%, τ^2 = 0.0170, p < 0.01						
**Common effect model**		**561978**	**0.80**	**[0.80; 0.80]**	**100.0%**	**−−**
**Random effects model**			**0.77**	**[0.74; 0.79]**	**−−**	**100.0%**
Heterogeneity: I^2 = 100%, τ^2 = 0.0501, p = 0						

Test for subgroup differences (random effects): χ^2_5 = 82.22, df = 5 (p < 0.01)

Supplementary figure 13 The prevalence of serum 25(OH)D < 75 nmol/L by latitude

Appendix 8: The prevalence of vitamin D deficiency by World Bank income groups

Study	Events	Total	Proportion	95%−CI	Weight (common)	Weight (random)
**High-income countries**						
Abiaka C	80	206	0.39	[0.32; 0.46]	0.0%	0.4%
Al Shaikh A	1687	3613	0.47	[0.45; 0.48]	0.0%	0.4%
Al Shaikh AM	61	204	0.30	[0.24; 0.37]	0.0%	0.4%
Al Shaikh AM	899	1906	0.47	[0.45; 0.49]	0.0%	0.4%
Al−Daghri NM	150	561	0.27	[0.23; 0.31]	0.0%	0.4%
Al−Daghri NM	235	830	0.28	[0.25; 0.32]	0.0%	0.4%
Al−Daghri NM	276	808	0.34	[0.31; 0.38]	0.0%	0.4%
Al−Daghri NM	718	2225	0.32	[0.30; 0.34]	0.0%	0.4%
Al−Daghri NM	1082	4247	0.25	[0.24; 0.27]	0.1%	0.4%
Al−Daghri NM	1083	3111	0.35	[0.33; 0.37]	0.0%	0.4%
Alkerwi A	207	1335	0.16	[0.14; 0.18]	0.0%	0.4%
Al−Kindi MK	51	100	0.51	[0.41; 0.61]	0.0%	0.4%
AlQuaiz AM	729	2832	0.26	[0.24; 0.27]	0.0%	0.4%
Al−Saleh Y	718	2226	0.32	[0.30; 0.34]	0.0%	0.4%
Al−Taiar A	559	1416	0.39	[0.37; 0.42]	0.0%	0.4%
Alyahya K	169	232	0.73	[0.67; 0.78]	0.0%	0.4%
Alyahya KO	88	104	0.85	[0.76; 0.91]	0.0%	0.4%
Andersen R	8	52	0.15	[0.07; 0.28]	0.0%	0.4%
Andersen R	13	54	0.24	[0.13; 0.38]	0.0%	0.4%
Andersen R	17	221	0.08	[0.05; 0.12]	0.0%	0.4%
Andersen R	30	37	0.81	[0.65; 0.92]	0.0%	0.3%
Andersen R	37	199	0.19	[0.13; 0.25]	0.0%	0.4%
Andersen R	158	219	0.72	[0.66; 0.78]	0.0%	0.4%
Ardawi MS	264	843	0.31	[0.28; 0.35]	0.0%	0.4%
Ardawi MS	534	1172	0.46	[0.43; 0.48]	0.0%	0.4%
Arnljots R	224	545	0.41	[0.37; 0.45]	0.0%	0.4%
Asakura K	40	107	0.37	[0.28; 0.47]	0.0%	0.4%
Aspell N	1423	6004	0.24	[0.23; 0.25]	0.1%	0.4%
Aucoin M	78	756	0.10	[0.08; 0.13]	0.0%	0.4%
Aucoin M	96	461	0.21	[0.17; 0.25]	0.0%	0.4%
Bjarnadottir A	5	158	0.03	[0.01; 0.07]	0.0%	0.4%
Black LJ	16	331	0.05	[0.03; 0.08]	0.0%	0.4%
Black LJ	23	490	0.05	[0.03; 0.07]	0.0%	0.4%
Black LJ	26	730	0.04	[0.02; 0.05]	0.0%	0.4%
Black LJ	30	648	0.05	[0.03; 0.07]	0.0%	0.4%
Black LJ	43	652	0.07	[0.05; 0.09]	0.0%	0.4%
Bolland MJ	2	378	0.01	[0.00; 0.02]	0.0%	0.4%
Brinkmann K	67	108	0.62	[0.52; 0.71]	0.0%	0.4%
Cairncross CT	86	1329	0.06	[0.05; 0.08]	0.0%	0.4%
Capuano R	137	1200	0.11	[0.10; 0.13]	0.0%	0.4%
Cashman KD	76	1132	0.07	[0.05; 0.08]	0.0%	0.4%
Ceccarelli M	209	2140	0.10	[0.09; 0.11]	0.0%	0.4%
Chao YS	13	1493	0.01	[0.00; 0.01]	0.0%	0.4%
Chlebna−Sokó.. D	145	720	0.20	[0.17; 0.23]	0.0%	0.4%
Choi HR	110	651	0.17	[0.14; 0.20]	0.0%	0.4%
Cougnard−Grégoire A	190	697	0.27	[0.24; 0.31]	0.0%	0.4%
Courraud J	31	177	0.18	[0.12; 0.24]	0.0%	0.4%
Crowe FL	223186	755174	0.30	[0.29; 0.30]	9.9%	0.4%
Crowe FL	299730	993895	0.30	[0.30; 0.30]	13.0%	0.4%
Crowe FL	300265	901352	0.33	[0.33; 0.33]	11.8%	0.4%
Crowe FL	443448	1180086	0.38	[0.37; 0.38]	15.5%	0.4%
Crowe FL	531681	1316390	0.40	[0.40; 0.40]	17.2%	0.4%
Crowe FL	536113	1269812	0.42	[0.42; 0.42]	16.6%	0.4%
Dalgård C	124	669	0.19	[0.16; 0.22]	0.0%	0.4%
Daly RM	46	1393	0.03	[0.02; 0.04]	0.0%	0.4%
Daly RM	66	810	0.08	[0.06; 0.10]	0.0%	0.4%
Daly RM	76	1564	0.05	[0.04; 0.06]	0.0%	0.4%
Daly RM	77	2011	0.04	[0.03; 0.05]	0.0%	0.4%
Daly RM	108	2556	0.04	[0.03; 0.05]	0.0%	0.4%
Daly RM	111	2884	0.04	[0.03; 0.05]	0.0%	0.4%
Duarte C	706	3092	0.23	[0.21; 0.24]	0.0%	0.4%
El Hayek J	3	508	0.01	[0.00; 0.02]	0.0%	0.4%
El Hayek J	39	282	0.14	[0.10; 0.18]	0.0%	0.4%
Fernández Bustillo JM	9	153	0.06	[0.03; 0.11]	0.0%	0.4%
Gill TK	21	2413	0.01	[0.01; 0.01]	0.0%	0.4%
Ginter JK	3	224	0.01	[0.00; 0.04]	0.0%	0.4%
Glatt DU	3	49	0.06	[0.01; 0.17]	0.0%	0.4%
Golbahar J	247	500	0.49	[0.45; 0.54]	0.0%	0.4%
Granlund L	26	216	0.12	[0.08; 0.17]	0.0%	0.4%
Greene−Finestone LS	8	312	0.03	[0.01; 0.05]	0.0%	0.4%
Greene−Finestone LS	18	777	0.02	[0.01; 0.04]	0.0%	0.4%
Greene−Finestone LS	19	823	0.02	[0.01; 0.04]	0.0%	0.4%
Griffin TP	2047	15319	0.13	[0.13; 0.14]	0.2%	0.4%
Hansen L	79	527	0.15	[0.12; 0.18]	0.0%	0.4%
Hansen L	309	2565	0.12	[0.11; 0.13]	0.0%	0.4%
Herrick KA	7	1438	0.00	[0.00; 0.01]	0.0%	0.4%
Herrick KA	29	2060	0.01	[0.01; 0.02]	0.0%	0.4%
Herrick KA	94	3267	0.03	[0.02; 0.04]	0.0%	0.4%
Herrick KA	113	2355	0.05	[0.04; 0.06]	0.0%	0.4%
Herrick KA	199	3496	0.06	[0.05; 0.07]	0.0%	0.4%
Herrick KA	271	3564	0.08	[0.07; 0.09]	0.0%	0.4%
Hintzpeter B	1797	9001	0.20	[0.19; 0.21]	0.1%	0.4%
Hirani V	27	174	0.16	[0.10; 0.22]	0.0%	0.4%
Hirani V	30	304	0.10	[0.07; 0.14]	0.0%	0.4%
Hirani V	45	522	0.09	[0.06; 0.11]	0.0%	0.4%
Hirani V	57	659	0.09	[0.07; 0.11]	0.0%	0.4%
Hirani V	292	2070	0.14	[0.13; 0.16]	0.0%	0.4%
Hoge A	58	915	0.06	[0.05; 0.08]	0.0%	0.4%
Hribar M	31	125	0.25	[0.18; 0.33]	0.0%	0.4%
Hribar M	36	155	0.23	[0.17; 0.31]	0.0%	0.4%
Ikonen H	92	3650	0.03	[0.02; 0.03]	0.0%	0.4%
Jääskeläinen T	17	4051	0.00	[0.00; 0.01]	0.1%	0.4%
Jolliffe DA	55	222	0.25	[0.19; 0.31]	0.0%	0.4%
Jorde R	157	2668	0.06	[0.05; 0.07]	0.0%	0.4%
Kaddam IM	912	2104	0.43	[0.41; 0.45]	0.0%	0.4%
Kaddam IM	1979	4035	0.49	[0.47; 0.51]	0.1%	0.4%
Karin Z	31	260	0.12	[0.08; 0.16]	0.0%	0.4%
Kim SY	10652	157211	0.07	[0.07; 0.07]	2.1%	0.4%
Kunz C	63	327	0.19	[0.15; 0.24]	0.0%	0.4%
Kunz C	106	528	0.20	[0.17; 0.24]	0.0%	0.4%
Kunz C	111	456	0.24	[0.20; 0.29]	0.0%	0.4%
Kunz C	112	520	0.22	[0.18; 0.25]	0.0%	0.4%
Laird E	728	5356	0.14	[0.13; 0.15]	0.1%	0.4%
Langlois K	218	5306	0.04	[0.04; 0.05]	0.1%	0.4%
Lardner E	14	143	0.10	[0.05; 0.16]	0.0%	0.4%
Le Goaziou MF	105	196	0.54	[0.46; 0.61]	0.0%	0.4%
Lin LY	60687	448601	0.14	[0.13; 0.14]	5.9%	0.4%
Lucas JA	92	1606	0.06	[0.05; 0.07]	0.0%	0.4%
Madsen KH	4	415	0.01	[0.00; 0.02]	0.0%	0.4%
Maguire JL	1	91	0.01	[0.00; 0.06]	0.0%	0.4%
Man PW	40	416	0.10	[0.07; 0.13]	0.0%	0.4%
Manios Y	122	2353	0.05	[0.04; 0.06]	0.0%	0.4%
Mansbach JM	17	1799	0.01	[0.01; 0.02]	0.0%	0.4%
Mansbach JM	27	2759	0.01	[0.01; 0.01]	0.0%	0.4%
Masoud MS	117	170	0.69	[0.61; 0.76]	0.0%	0.4%
Matheï C	129	367	0.35	[0.30; 0.40]	0.0%	0.4%
Mitchell DM	45	634	0.07	[0.05; 0.09]	0.0%	0.4%
Moreno−Reyes R	135	401	0.34	[0.29; 0.39]	0.0%	0.4%
Nakamura K	22	600	0.04	[0.02; 0.05]	0.0%	0.4%
Nälsén C	9	268	0.03	[0.02; 0.06]	0.0%	0.4%
Nälsén C	10	206	0.05	[0.02; 0.09]	0.0%	0.4%
NHANES (2015...2016)	37	1777	0.02	[0.01; 0.03]	0.0%	0.4%
NHANES(2015...2016)	23	1231	0.02	[0.01; 0.03]	0.0%	0.4%
NHANES(2015...2016)	26	2618	0.01	[0.01; 0.01]	0.0%	0.4%
NHANES(2015...2016)	85	2413	0.04	[0.03; 0.04]	0.0%	0.4%
NHANES(2017...2018)	12	1321	0.01	[0.00; 0.02]	0.0%	0.4%
NHANES(2017...2018)	37	2184	0.02	[0.01; 0.02]	0.0%	0.4%
NHANES(2017...2018)	43	1810	0.02	[0.02; 0.03]	0.0%	0.4%
NHANES(2017...2018)	80	2094	0.04	[0.03; 0.05]	0.0%	0.4%
Ní Chaoimh C	12	741	0.02	[0.01; 0.03]	0.0%	0.4%
Nielsen NO	525	2877	0.18	[0.17; 0.20]	0.0%	0.4%
Oberg J	147	890	0.17	[0.14; 0.19]	0.0%	0.4%
Orwoll E	47	1606	0.03	[0.02; 0.04]	0.0%	0.4%
Patel JV	1283	1904	0.67	[0.65; 0.69]	0.0%	0.4%
Pérez−Llamas F	28	86	0.33	[0.23; 0.44]	0.0%	0.4%
Perna L	844	5386	0.16	[0.15; 0.17]	0.1%	0.4%
Petrenya N	31	4465	0.01	[0.00; 0.01]	0.1%	0.4%
Rabenberg M	540	1816	0.30	[0.28; 0.32]	0.0%	0.4%
Rabenberg M	756	2456	0.31	[0.29; 0.33]	0.0%	0.4%
Rabenberg M	804	2723	0.30	[0.28; 0.31]	0.0%	0.4%
Rabufetti A	24	1045	0.02	[0.01; 0.03]	0.0%	0.4%
Raposo L	188	500	0.38	[0.33; 0.42]	0.0%	0.4%
Riverin B	3	52	0.06	[0.01; 0.16]	0.0%	0.4%
Riverin B	54	944	0.06	[0.04; 0.07]	0.0%	0.4%
Robinson PJ	37	267	0.14	[0.10; 0.19]	0.0%	0.4%
Robinson PJ	74	640	0.12	[0.09; 0.14]	0.0%	0.4%
Rodríguez−Rodríguez E	8	102	0.08	[0.03; 0.15]	0.0%	0.4%
Saeed BQ	48	287	0.17	[0.13; 0.22]	0.0%	0.4%
Saliba W	891	6518	0.14	[0.13; 0.15]	0.1%	0.4%
Saliba W	27676	192240	0.14	[0.14; 0.15]	2.5%	0.4%
Samefors M	48	333	0.14	[0.11; 0.19]	0.0%	0.4%
Santos A	594	1500	0.40	[0.37; 0.42]	0.0%	0.4%
Sarafin K	839	11336	0.07	[0.07; 0.08]	0.1%	0.4%
Schramm S	660	4149	0.16	[0.15; 0.17]	0.1%	0.4%
Science M	4	743	0.01	[0.00; 0.01]	0.0%	0.4%
Sioen I	18	357	0.05	[0.03; 0.08]	0.0%	0.4%
Skull SA	61	116	0.53	[0.43; 0.62]	0.0%	0.4%
Smith N	19	148	0.13	[0.08; 0.19]	0.0%	0.4%
Sochorová L	101	419	0.24	[0.20; 0.28]	0.0%	0.4%
Solis−Urra P	181	686	0.26	[0.23; 0.30]	0.0%	0.4%
Solis−Urra P	204	1245	0.16	[0.14; 0.19]	0.0%	0.4%
Souberbielle JC	56	892	0.06	[0.05; 0.08]	0.0%	0.4%
Sulimani RA	645	1618	0.40	[0.37; 0.42]	0.0%	0.4%
Thuesen B	848	6146	0.14	[0.13; 0.15]	0.1%	0.4%
Tolppanen AM	124	7560	0.02	[0.01; 0.02]	0.1%	0.4%
Tønnesen R	135	700	0.19	[0.16; 0.22]	0.0%	0.4%
Tran B	9	134	0.07	[0.03; 0.12]	0.0%	0.4%
Tran B	11	148	0.07	[0.04; 0.13]	0.0%	0.4%
Tran B	11	148	0.07	[0.04; 0.13]	0.0%	0.4%
Tran B	15	131	0.11	[0.07; 0.18]	0.0%	0.4%
Tran B	20	92	0.22	[0.14; 0.32]	0.0%	0.4%
Tseng M	66	194	0.34	[0.27; 0.41]	0.0%	0.4%
von Hurst PR	98	228	0.43	[0.36; 0.50]	0.0%	0.4%
Voortman T	258	4167	0.06	[0.05; 0.07]	0.1%	0.4%
Wyskida M	355	3472	0.10	[0.09; 0.11]	0.0%	0.4%
Yousef S	5	267	0.02	[0.01; 0.04]	0.0%	0.4%
Yousef S	38	932	0.04	[0.03; 0.06]	0.0%	0.4%
Yousef S	63	1361	0.05	[0.04; 0.06]	0.0%	0.4%
Yousef S	90	858	0.10	[0.09; 0.13]	0.0%	0.4%
Yousef S	988	8161	0.12	[0.11; 0.13]	0.1%	0.4%
Zgaga L	772	2235	0.35	[0.33; 0.37]	0.0%	0.4%
Zhang FF	348	960	0.36	[0.33; 0.39]	0.0%	0.4%
Zhou SJ	9	221	0.04	[0.02; 0.08]	0.0%	0.4%
**Common effect model**		**7488490**	**0.32**	**[0.32; 0.32]**	**98.1%**	**−−**
**Random effects model**			**0.15**	**[0.13; 0.18]**	**−−**	**69.9%**
Heterogeneity: $I^2 = 100\%$, $\tau^2 = 0.0515$, $p = 0$						
**Low-income countries**						
Gebreegziabher T	29	196	0.15	[0.10; 0.21]	0.0%	0.4%
**Lower-middle-income countries**						
Arya V	44	92	0.48	[0.37; 0.58]	0.0%	0.4%
Bachhel R	60	150	0.40	[0.32; 0.48]	0.0%	0.4%
Bater J	5692	9595	0.59	[0.58; 0.60]	0.1%	0.4%
Bezrati I	92	225	0.41	[0.34; 0.48]	0.0%	0.4%
Bhatt SP	9	137	0.07	[0.03; 0.12]	0.0%	0.4%
Chakrabarty S	1166	5024	0.23	[0.22; 0.24]	0.1%	0.4%
Chakrabarty S	1608	6798	0.24	[0.23; 0.25]	0.1%	0.4%
Djennane M	35	435	0.08	[0.06; 0.11]	0.0%	0.4%
Drali O	93	621	0.15	[0.12; 0.18]	0.0%	0.4%
Ganmaa D	333	420	0.79	[0.75; 0.83]	0.0%	0.4%
Goswami R	559	642	0.87	[0.84; 0.90]	0.0%	0.4%
Hashemipour S	812	1210	0.67	[0.64; 0.70]	0.0%	0.4%
Hovsepian S	231	1111	0.21	[0.18; 0.23]	0.0%	0.4%
Islam MZ	27	189	0.14	[0.10; 0.20]	0.0%	0.4%
Islam MZ	31	244	0.13	[0.09; 0.18]	0.0%	0.4%
Jayatissa R	333	2525	0.13	[0.12; 0.15]	0.0%	0.4%
Joukar F	1189	5096	0.23	[0.22; 0.25]	0.1%	0.4%
Mechenro J	4	48	0.08	[0.02; 0.20]	0.0%	0.4%
Mechenro J	41	101	0.41	[0.31; 0.51]	0.0%	0.4%
Mechenro J	46	275	0.17	[0.13; 0.22]	0.0%	0.4%
Meddeb N	66	389	0.17	[0.13; 0.21]	0.0%	0.4%
Meyer HE	7	196	0.04	[0.01; 0.07]	0.0%	0.4%
Nakhaee S	115	400	0.29	[0.24; 0.33]	0.0%	0.4%
Niafar M	115	300	0.38	[0.33; 0.44]	0.0%	0.4%
Nikooyeh B	374	667	0.56	[0.52; 0.60]	0.0%	0.4%
Nikooyeh B	974	1406	0.69	[0.67; 0.72]	0.0%	0.4%
Paul TV	15	150	0.10	[0.06; 0.16]	0.0%	0.4%
Qorbani M	276	2596	0.11	[0.09; 0.12]	0.0%	0.4%
Saki F	63	477	0.13	[0.10; 0.17]	0.0%	0.4%
Sharawat IK	29	100	0.29	[0.20; 0.39]	0.0%	0.4%
Shchubelka K	211	1639	0.13	[0.11; 0.15]	0.0%	0.4%
Shetty S	18	252	0.07	[0.04; 0.11]	0.0%	0.4%
Shivane VK	220	1137	0.19	[0.17; 0.22]	0.0%	0.4%
Smith G	7	128	0.05	[0.02; 0.11]	0.0%	0.4%
Smith G	13	495	0.03	[0.01; 0.04]	0.0%	0.4%
Uush T	222	524	0.42	[0.38; 0.47]	0.0%	0.4%
Uush T	457	876	0.52	[0.49; 0.56]	0.0%	0.4%
**Common effect model**		**46670**	**0.32**	**[0.31; 0.32]**	**0.6%**	**−−**
**Random effects model**			**0.27**	**[0.20; 0.34]**	**−−**	**14.1%**
Heterogeneity: $I^2 = 100\%$, $\tau^2 = 0.0679$, $p = 0$						
**Upper-middle-income countries**						
Abdulrahman MA	13	63	0.21	[0.11; 0.33]	0.0%	0.4%
Abdulrahman MA	16	50	0.32	[0.20; 0.47]	0.0%	0.4%
Abdulrahman MA	50	278	0.18	[0.14; 0.23]	0.0%	0.4%
Al Hayek S	33	344	0.10	[0.07; 0.13]	0.0%	0.4%
Arabi A	182	466	0.39	[0.35; 0.44]	0.0%	0.4%
Beer RJ	129	6813	0.02	[0.02; 0.02]	0.1%	0.4%
Beer RJ	194	6470	0.03	[0.03; 0.03]	0.1%	0.4%
Beer RJ	265	7170	0.04	[0.03; 0.04]	0.1%	0.4%
Beer RJ	395	16454	0.02	[0.02; 0.03]	0.2%	0.4%
Borissova AM	430	2016	0.21	[0.20; 0.23]	0.0%	0.4%
Chen J	731	6014	0.12	[0.11; 0.13]	0.1%	0.4%
Chin KY	2	150	0.01	[0.00; 0.05]	0.0%	0.4%
Chirita−Emandi A	265	6631	0.04	[0.04; 0.04]	0.1%	0.4%
Fang F	139	1814	0.08	[0.06; 0.09]	0.0%	0.4%
Gromova O	371	1347	0.28	[0.25; 0.30]	0.0%	0.4%
Hatun S	19	89	0.21	[0.13; 0.31]	0.0%	0.4%
Hutchings N	157	1206	0.13	[0.11; 0.15]	0.0%	0.4%
Jiang W	1197	14302	0.08	[0.08; 0.09]	0.2%	0.4%
Karagüzel G	75	746	0.10	[0.08; 0.12]	0.0%	0.4%
Lategan R	1	339	0.00	[0.00; 0.02]	0.0%	0.4%
Li H	1020	4989	0.20	[0.19; 0.22]	0.1%	0.4%
Li H	1020	5707	0.18	[0.17; 0.19]	0.1%	0.4%
Lima−Costa MF	37	2264	0.02	[0.01; 0.02]	0.0%	0.4%
Lin L	1	353	0.00	[0.00; 0.02]	0.0%	0.4%
Lin L	1	477	0.00	[0.00; 0.01]	0.0%	0.4%
Lin L	1	860	0.00	[0.00; 0.01]	0.0%	0.4%
Maldonado G	3	269	0.01	[0.00; 0.03]	0.0%	0.4%
Mallah EM	8	300	0.03	[0.01; 0.05]	0.0%	0.4%
Nichols EK	180	915	0.20	[0.17; 0.22]	0.0%	0.4%
Oliveri B	35	339	0.10	[0.07; 0.14]	0.0%	0.4%
Öztürk ZA	150	1161	0.13	[0.11; 0.15]	0.0%	0.4%
Saraiva GL	33	214	0.15	[0.11; 0.21]	0.0%	0.4%
Scalco R	53	98	0.54	[0.44; 0.64]	0.0%	0.4%
Sokolovic S	93	450	0.21	[0.17; 0.25]	0.0%	0.4%
Sokolovic S	185	645	0.29	[0.25; 0.32]	0.0%	0.4%
Sokolovic S	235	735	0.32	[0.29; 0.35]	0.0%	0.4%
Yan X	81	302	0.27	[0.22; 0.32]	0.0%	0.4%
Yu S	128	2173	0.06	[0.05; 0.07]	0.0%	0.4%
Zhu Z	6	183	0.03	[0.01; 0.07]	0.0%	0.4%
Zhu Z	26	2269	0.01	[0.01; 0.02]	0.0%	0.4%
Zhu Z	29	1440	0.02	[0.01; 0.03]	0.0%	0.4%
**Common effect model**		**98905**	**0.07**	**[0.06; 0.07]**	**1.3%**	**−−**
**Random effects model**			**0.10**	**[0.07; 0.14]**	**−−**	**15.6%**
Heterogeneity: $I^2 = 99\%$, $\tau^2 = 0.0365$, $p = 0$						
**Common effect model**		**7634261**	**0.32**	**[0.32; 0.32]**	**100.0%**	**−−**
**Random effects model**			**0.16**	**[0.14; 0.18]**	**−−**	**100.0%**
Heterogeneity: $I^2 = 100\%$, $\tau^2 = 0.0547$, $p = 0$						

0.2 0.4 0.6 0.8

Test for subgroup differences (random effects): $\chi^2_3 = 17.09$, df = 3 ($p < 0.01$)

Supplementary figure 14 The prevalence of serum 25(OH)D < 30 nmol/L by World Bank income groups

Appendix 8: The prevalence of vitamin D deficiency by World Bank income groups

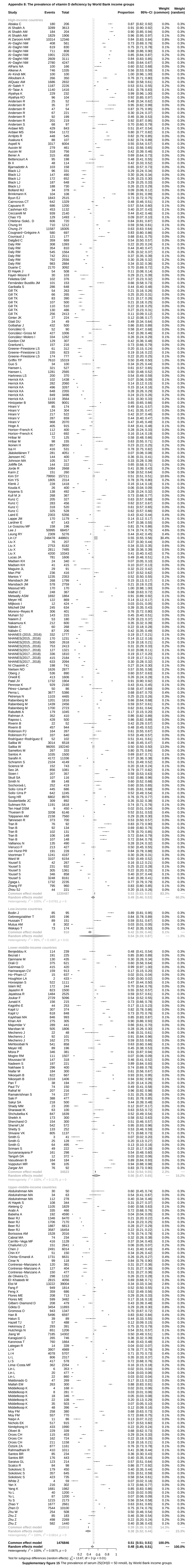

Supplementary figure 15 The prevalence of serum 25(OH)D < 50 nmol/L by World Bank income groups

Appendix 8: The prevalence of vitamin D deficiency by World Bank income groups

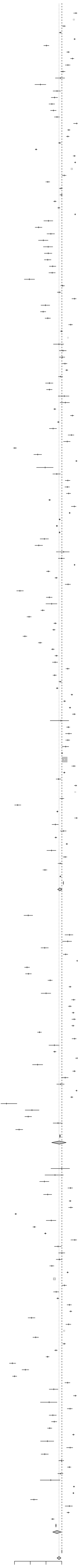

Study	Events	Total	Proportion	95%−CI	Weight (common)	Weight (random)
**High-income countries**						
Abiaka C	202	206	0.98	[0.95; 0.99]	0.0%	0.4%
Al Zarooni AAR	11765	12346	0.95	[0.95; 0.96]	2.2%	0.5%
Alkerwi A	1107	1335	0.83	[0.81; 0.85]	0.2%	0.5%
AlQuaiz AM	2215	2832	0.78	[0.77; 0.80]	0.5%	0.5%
Al−Taiar A	1365	1416	0.96	[0.95; 0.97]	0.3%	0.5%
Ardawi MS	510	843	0.60	[0.57; 0.64]	0.1%	0.5%
Ardawi MS	1034	1172	0.88	[0.86; 0.90]	0.2%	0.5%
Arnljots R	510	545	0.94	[0.91; 0.95]	0.1%	0.5%
Aucoin M	405	461	0.88	[0.85; 0.91]	0.1%	0.5%
Aucoin M	617	756	0.82	[0.79; 0.84]	0.1%	0.5%
Bettencourt A	155	198	0.78	[0.72; 0.84]	0.0%	0.4%
Bhattoa HP	109	206	0.53	[0.46; 0.60]	0.0%	0.4%
Black LJ	245	331	0.74	[0.69; 0.79]	0.1%	0.5%
Black LJ	347	490	0.71	[0.67; 0.75]	0.1%	0.5%
Black LJ	440	652	0.67	[0.64; 0.71]	0.1%	0.5%
Black LJ	450	648	0.69	[0.66; 0.73]	0.1%	0.5%
Black LJ	540	730	0.74	[0.71; 0.77]	0.1%	0.5%
Brinkmann K	107	108	0.99	[0.95; 1.00]	0.0%	0.4%
Cairncross CT	1183	1329	0.89	[0.87; 0.91]	0.2%	0.5%
Capuano R	1053	1200	0.88	[0.86; 0.90]	0.2%	0.5%
Ceccarelli M	1657	2140	0.77	[0.76; 0.79]	0.4%	0.5%
Chao YS	2896	6101	0.47	[0.46; 0.49]	1.1%	0.5%
Choi HR	626	651	0.96	[0.94; 0.97]	0.1%	0.5%
Chung IH	1177	1212	0.97	[0.96; 0.98]	0.2%	0.5%
Chung JY	16973	18305	0.93	[0.92; 0.93]	3.2%	0.5%
Daly RM	673	810	0.83	[0.80; 0.86]	0.1%	0.5%
Daly RM	868	1393	0.62	[0.60; 0.65]	0.2%	0.5%
Daly RM	1235	1564	0.79	[0.77; 0.81]	0.3%	0.5%
Daly RM	1598	2011	0.79	[0.78; 0.81]	0.4%	0.5%
Daly RM	1824	2556	0.71	[0.70; 0.73]	0.5%	0.5%
Daly RM	2202	2884	0.76	[0.75; 0.78]	0.5%	0.5%
Duarte C	3000	3092	0.97	[0.96; 0.98]	0.5%	0.5%
El Hayek J	178	282	0.63	[0.57; 0.69]	0.1%	0.5%
El Hayek J	257	508	0.51	[0.46; 0.55]	0.1%	0.5%
Feketea GM	249	376	0.66	[0.61; 0.71]	0.1%	0.5%
Gill TK	150	265	0.57	[0.50; 0.63]	0.0%	0.4%
Gill TK	165	263	0.63	[0.57; 0.69]	0.0%	0.4%
Gill TK	245	390	0.63	[0.58; 0.68]	0.1%	0.5%
Gill TK	311	500	0.62	[0.58; 0.66]	0.1%	0.5%
Gill TK	333	486	0.69	[0.64; 0.73]	0.1%	0.5%
Gill TK	346	510	0.68	[0.64; 0.72]	0.1%	0.5%
Ginter JK	87	224	0.39	[0.32; 0.46]	0.0%	0.4%
González−Gross M	817	1006	0.81	[0.79; 0.84]	0.2%	0.5%
González−Molero I	968	1262	0.77	[0.74; 0.79]	0.2%	0.5%
Granlund L	208	216	0.96	[0.93; 0.98]	0.0%	0.4%
Greene−Finestone LS	185	312	0.59	[0.54; 0.65]	0.1%	0.5%
Greene−Finestone LS	464	823	0.56	[0.53; 0.60]	0.1%	0.5%
Greene−Finestone LS	484	777	0.62	[0.59; 0.66]	0.1%	0.5%
Hansen L	482	527	0.91	[0.89; 0.94]	0.1%	0.5%
Hansen L	2045	2565	0.80	[0.78; 0.81]	0.5%	0.5%
Hintzpeter B	7911	9001	0.88	[0.87; 0.89]	1.6%	0.5%
Hirani V	133	174	0.76	[0.69; 0.83]	0.0%	0.4%
Hirani V	248	304	0.82	[0.77; 0.86]	0.1%	0.5%
Hirani V	421	522	0.81	[0.77; 0.84]	0.1%	0.5%
Hirani V	554	659	0.84	[0.81; 0.87]	0.1%	0.5%
Hirani V	1788	2070	0.86	[0.85; 0.88]	0.4%	0.5%
Hoge A	719	915	0.79	[0.76; 0.81]	0.2%	0.5%
Horton−French K	257	400	0.64	[0.59; 0.69]	0.1%	0.5%
Horton−French K	445	692	0.64	[0.61; 0.68]	0.1%	0.5%
Hribar M	104	125	0.83	[0.75; 0.89]	0.0%	0.4%
Hribar M	131	155	0.85	[0.78; 0.90]	0.0%	0.4%
Ikonen H	2597	3650	0.71	[0.70; 0.73]	0.6%	0.5%
Isa H	496	531	0.93	[0.91; 0.95]	0.1%	0.5%
Jääskeläinen T	3068	4051	0.76	[0.74; 0.77]	0.7%	0.5%
Janssen HC	276	400	0.69	[0.64; 0.74]	0.1%	0.5%
Jolliffe DA	205	222	0.92	[0.88; 0.95]	0.0%	0.4%
Karin Z	226	260	0.87	[0.82; 0.91]	0.0%	0.4%
Klenk J	291	1418	0.21	[0.18; 0.23]	0.3%	0.5%
Kouda K	198	400	0.50	[0.44; 0.55]	0.1%	0.5%
Koyama S	486	492	0.99	[0.97; 1.00]	0.1%	0.5%
Kremer R	53	90	0.59	[0.48; 0.69]	0.0%	0.4%
Kunz C	241	327	0.74	[0.69; 0.78]	0.1%	0.5%
Kunz C	400	456	0.88	[0.84; 0.91]	0.1%	0.5%
Kunz C	453	520	0.87	[0.84; 0.90]	0.1%	0.5%
Kunz C	469	528	0.89	[0.86; 0.91]	0.1%	0.5%
Langlois K	3428	5306	0.65	[0.63; 0.66]	0.9%	0.5%
Le Goaziou MF	188	196	0.96	[0.92; 0.98]	0.0%	0.4%
Leung RY	4752	5276	0.90	[0.89; 0.91]	0.9%	0.5%
Liu X	5785	7485	0.77	[0.76; 0.78]	1.3%	0.5%
Liu X	6041	8182	0.74	[0.73; 0.75]	1.5%	0.5%
Liu X	7985	10343	0.77	[0.76; 0.78]	1.8%	0.5%
Madsen KH	197	340	0.58	[0.52; 0.63]	0.1%	0.5%
Madsen KH	211	415	0.51	[0.46; 0.56]	0.1%	0.5%
Maguire JL	75	91	0.82	[0.73; 0.90]	0.0%	0.4%
Man PW	332	416	0.80	[0.76; 0.84]	0.1%	0.5%
Manios Y	2266	2353	0.96	[0.95; 0.97]	0.4%	0.5%
Mansbach JM	1131	1799	0.63	[0.61; 0.65]	0.3%	0.5%
Mansbach JM	2013	2759	0.73	[0.71; 0.75]	0.5%	0.5%
Matheï C	323	367	0.88	[0.84; 0.91]	0.1%	0.5%
Miljkovic I	114	424	0.27	[0.23; 0.31]	0.1%	0.5%
Mitchell DM	407	634	0.64	[0.60; 0.68]	0.1%	0.5%
Naeem Z	121	180	0.67	[0.60; 0.74]	0.0%	0.4%
NHANES (2015...2016)	992	1777	0.56	[0.53; 0.58]	0.3%	0.5%
NHANES(2015...2016)	474	1231	0.39	[0.36; 0.41]	0.2%	0.5%
NHANES(2015...2016)	1730	2413	0.72	[0.70; 0.73]	0.4%	0.5%
NHANES(2015...2016)	1833	2618	0.70	[0.68; 0.72]	0.5%	0.5%
NHANES(2017...2018)	439	1321	0.33	[0.31; 0.36]	0.2%	0.5%
NHANES(2017...2018)	950	1810	0.52	[0.50; 0.55]	0.3%	0.5%
NHANES(2017...2018)	1500	2184	0.69	[0.67; 0.71]	0.4%	0.5%
NHANES(2017...2018)	1533	2094	0.73	[0.71; 0.75]	0.4%	0.5%
Ní Chaoimh C	530	741	0.72	[0.68; 0.75]	0.1%	0.5%
Oberg J	780	890	0.88	[0.85; 0.90]	0.2%	0.5%
Orwoll E	1142	1606	0.71	[0.69; 0.73]	0.3%	0.5%
Penrose K	1685	2160	0.78	[0.76; 0.80]	0.4%	0.5%
Petrenya N	3313	4465	0.74	[0.73; 0.75]	0.8%	0.5%
Rabenberg M	1687	1816	0.93	[0.92; 0.94]	0.3%	0.5%
Rabenberg M	2054	2456	0.84	[0.82; 0.85]	0.4%	0.5%
Rabenberg M	2465	2723	0.91	[0.89; 0.92]	0.5%	0.5%
Rahman A	393	410	0.96	[0.93; 0.98]	0.1%	0.5%
Riverin B	41	52	0.79	[0.65; 0.89]	0.0%	0.4%
Riverin B	834	944	0.88	[0.86; 0.90]	0.2%	0.5%
Robinson PJ	238	267	0.89	[0.85; 0.93]	0.0%	0.4%
Robinson PJ	562	640	0.88	[0.85; 0.90]	0.1%	0.5%
Saeed BQ	244	287	0.85	[0.80; 0.89]	0.1%	0.5%
Saliba W	5237	6518	0.80	[0.79; 0.81]	1.2%	0.5%
Saliba W	160952	192240	0.84	[0.84; 0.84]	34.1%	0.5%
Samefors M	318	333	0.95	[0.93; 0.97]	0.1%	0.5%
Schramm S	3456	4149	0.83	[0.82; 0.84]	0.7%	0.5%
Science M	565	743	0.76	[0.73; 0.79]	0.1%	0.5%
Sioen I	350	357	0.98	[0.96; 0.99]	0.1%	0.5%
Song HR	8720	8976	0.97	[0.97; 0.97]	1.6%	0.5%
Souberbielle JC	716	892	0.80	[0.78; 0.83]	0.2%	0.5%
Ten Haaf DSM	108	450	0.24	[0.20; 0.28]	0.1%	0.5%
Tolppanen AM	5631	7560	0.74	[0.73; 0.75]	1.3%	0.5%
Tseng M	192	194	0.99	[0.96; 1.00]	0.0%	0.4%
Vallianou N	352	490	0.72	[0.68; 0.76]	0.1%	0.5%
Vierucci F	351	427	0.82	[0.78; 0.86]	0.1%	0.5%
Voortman T	3020	4167	0.72	[0.71; 0.74]	0.7%	0.5%
Wyskida M	3002	3472	0.86	[0.85; 0.88]	0.6%	0.5%
Yousef S	179	267	0.67	[0.61; 0.73]	0.0%	0.4%
Yousef S	723	858	0.84	[0.82; 0.87]	0.2%	0.5%
Yousef S	727	932	0.78	[0.75; 0.81]	0.2%	0.5%
Yousef S	799	1361	0.59	[0.56; 0.61]	0.2%	0.5%
Yousef S	6386	8161	0.78	[0.77; 0.79]	1.4%	0.5%
**Common effect model**		**434195**	**0.82**	**[0.82; 0.82]**	**77.1%**	**−−**
**Random effects model**			**0.78**	**[0.75; 0.80]**	**−−**	**60.8%**
Heterogeneity: $I^2 = 100\%$, $\tau^2 = 0.0385$, $p = 0$						
**Low-income countries**						
Mutua AM	113	302	0.37	[0.32; 0.43]	0.1%	0.5%
**Lower-middle-income countries**						
Bachhel R	135	150	0.90	[0.84; 0.94]	0.0%	0.4%
Bhatt SP	120	137	0.88	[0.81; 0.93]	0.0%	0.4%
Djennane M	254	435	0.58	[0.54; 0.63]	0.1%	0.5%
Drali O	528	621	0.85	[0.82; 0.88]	0.1%	0.5%
Ganmaa D	419	420	1.00	[0.99; 1.00]	0.1%	0.5%
Harinarayan CV	328	913	0.36	[0.33; 0.39]	0.2%	0.5%
Ho−Pham LT	240	637	0.38	[0.34; 0.42]	0.1%	0.5%
Hovsepian S	725	1111	0.65	[0.62; 0.68]	0.2%	0.5%
Jayashri R	1360	1500	0.91	[0.89; 0.92]	0.3%	0.5%
Kagotho E	152	253	0.60	[0.54; 0.66]	0.0%	0.4%
Kapil U	356	374	0.95	[0.93; 0.97]	0.1%	0.5%
Kapil U	767	848	0.90	[0.88; 0.92]	0.2%	0.5%
Kaykhaei MA	940	993	0.95	[0.93; 0.96]	0.2%	0.5%
Khan AH	292	305	0.96	[0.93; 0.98]	0.1%	0.5%
Mehboobali N	810	858	0.94	[0.93; 0.96]	0.2%	0.5%
Mogire RM	780	1507	0.52	[0.49; 0.54]	0.3%	0.5%
Nadeem S	213	221	0.96	[0.93; 0.98]	0.0%	0.4%
Pan T	137	194	0.71	[0.64; 0.77]	0.0%	0.4%
Qorbani M	1846	2596	0.71	[0.69; 0.73]	0.5%	0.5%
Rafraf M	215	216	1.00	[0.97; 1.00]	0.0%	0.4%
Ramakrishnan S	117	237	0.49	[0.43; 0.56]	0.0%	0.4%
Saki F	458	477	0.96	[0.94; 0.98]	0.1%	0.5%
Sheikh A	253	300	0.84	[0.80; 0.88]	0.1%	0.5%
Sherchand O	236	300	0.79	[0.74; 0.83]	0.1%	0.5%
Sherief LM	566	572	0.99	[0.98; 1.00]	0.1%	0.5%
Shivane VK	1055	1137	0.93	[0.91; 0.94]	0.2%	0.5%
Smith G	4	41	0.10	[0.03; 0.23]	0.0%	0.4%
Smith G	54	128	0.42	[0.34; 0.51]	0.0%	0.4%
Smith G	185	495	0.37	[0.33; 0.42]	0.1%	0.5%
Srimani S	167	222	0.75	[0.69; 0.81]	0.0%	0.4%
Tangoh DA	96	372	0.26	[0.21; 0.31]	0.1%	0.5%
**Common effect model**		**18570**	**0.78**	**[0.77; 0.78]**	**3.3%**	**−−**
**Random effects model**			**0.77**	**[0.67; 0.86]**	**−−**	**14.0%**
Heterogeneity: $I^2 = 99\%$, $\tau^2 = 0.0955$, $p = 0$						
**Upper-middle-income countries**						
Abdulrahman MA	40	50	0.80	[0.66; 0.90]	0.0%	0.4%
Abdulrahman MA	45	63	0.71	[0.59; 0.82]	0.0%	0.4%
Abdulrahman MA	161	278	0.58	[0.52; 0.64]	0.0%	0.4%
Abu−Samak MS	338	371	0.91	[0.88; 0.94]	0.1%	0.5%
Al Hayek S	214	344	0.62	[0.57; 0.67]	0.1%	0.5%
Aleteng Q	1659	1829	0.91	[0.89; 0.92]	0.3%	0.5%
Arabi A	427	466	0.92	[0.89; 0.94]	0.1%	0.5%
Batieha A	991	4590	0.22	[0.20; 0.23]	0.8%	0.5%
Cabral MA	156	234	0.67	[0.60; 0.73]	0.0%	0.4%
Chailurkit LO	1194	2641	0.45	[0.43; 0.47]	0.5%	0.5%
Chirita−Emandi A	3926	6631	0.59	[0.58; 0.60]	1.2%	0.5%
Cinar N	114	118	0.97	[0.92; 0.99]	0.0%	0.4%
Contreras−Manzano A	287	381	0.75	[0.71; 0.80]	0.1%	0.5%
Contreras−Manzano A	324	404	0.80	[0.76; 0.84]	0.1%	0.5%
Contreras−Manzano A	367	477	0.77	[0.73; 0.81]	0.1%	0.5%
de Oliveira CL	777	1152	0.67	[0.65; 0.70]	0.2%	0.5%
El−Khateeb M	3548	4056	0.87	[0.86; 0.88]	0.7%	0.5%
Eloi M	27576	39004	0.71	[0.70; 0.71]	6.9%	0.5%
Feng X	572	686	0.83	[0.80; 0.86]	0.1%	0.5%
Flores ME	520	713	0.73	[0.70; 0.76]	0.1%	0.5%
Flores ME	2612	3482	0.75	[0.74; 0.76]	0.6%	0.5%
Gilbert−Diamond D	430	479	0.90	[0.87; 0.92]	0.1%	0.5%
Gromova O	1232	1347	0.91	[0.90; 0.93]	0.2%	0.5%
Hazell TJ	203	488	0.42	[0.37; 0.46]	0.1%	0.5%
Hekimsoy Z	347	391	0.89	[0.85; 0.92]	0.1%	0.5%
Jiang W	11874	14302	0.83	[0.82; 0.84]	2.5%	0.5%
Karagüzel G	350	746	0.47	[0.43; 0.51]	0.1%	0.5%
Karonova T	1384	1664	0.83	[0.81; 0.85]	0.3%	0.5%
Li L	1683	2317	0.73	[0.71; 0.74]	0.4%	0.5%
Lima−Costa MF	1404	2264	0.62	[0.60; 0.64]	0.4%	0.5%
Lin L	61	353	0.17	[0.13; 0.22]	0.1%	0.5%
Lin L	161	477	0.34	[0.30; 0.38]	0.1%	0.5%
Lin L	173	860	0.20	[0.17; 0.23]	0.2%	0.5%
Lopes JB	363	415	0.87	[0.84; 0.90]	0.1%	0.5%
Maldonado G	188	269	0.70	[0.64; 0.75]	0.0%	0.4%
Mallah EM	292	300	0.97	[0.95; 0.99]	0.1%	0.5%
Naqvi A	55	86	0.64	[0.53; 0.74]	0.0%	0.4%
Oliveri B	307	339	0.91	[0.87; 0.93]	0.1%	0.5%
Orces CH	277	403	0.69	[0.64; 0.73]	0.1%	0.5%
Orces CH	510	724	0.70	[0.67; 0.74]	0.1%	0.5%
Orces CH	750	1158	0.65	[0.62; 0.68]	0.2%	0.5%
Öztürk ZA	1102	1161	0.95	[0.93; 0.96]	0.2%	0.5%
Peters BS	84	136	0.62	[0.53; 0.70]	0.0%	0.4%
Santos BR	212	234	0.91	[0.86; 0.94]	0.0%	0.4%
Sokolovic S	263	450	0.58	[0.54; 0.63]	0.1%	0.5%
Sokolovic S	513	645	0.80	[0.76; 0.83]	0.1%	0.5%
Sokolovic S	659	735	0.90	[0.87; 0.92]	0.1%	0.5%
Unger MD	473	603	0.78	[0.75; 0.82]	0.1%	0.5%
White Z	39	59	0.66	[0.53; 0.78]	0.0%	0.4%
Yang K	1893	1982	0.96	[0.95; 0.96]	0.4%	0.5%
Zhao Y	2523	2661	0.95	[0.94; 0.96]	0.5%	0.5%
Zhu W	227	508	0.45	[0.40; 0.49]	0.1%	0.5%
Zhu Z	164	183	0.90	[0.84; 0.94]	0.0%	0.4%
Zhu Z	1271	1440	0.88	[0.86; 0.90]	0.3%	0.5%
Zhu Z	1555	2269	0.69	[0.67; 0.70]	0.4%	0.5%
**Common effect model**		**110418**	**0.73**	**[0.72; 0.73]**	**19.6%**	**−−**
**Random effects model**			**0.74**	**[0.68; 0.79]**	**−−**	**24.8%**
Heterogeneity: $I^2 = 100\%$, $\tau^2 = 0.0534$, $p = 0$						
**Common effect model**		**563485**	**0.80**	**[0.80; 0.80]**	**100.0%**	**−−**
**Random effects model**			**0.77**	**[0.74; 0.79]**	**−−**	**100.0%**
Heterogeneity: $I^2 = 100\%$, $\tau^2 = 0.0502$, $p = 0$						

Test for subgroup differences (random effects): $\chi^2_3 = 163.30$, df = 3 ($p < 0.01$)

Supplementary figure 16 The prevalence of serum 25(OH)D < 75 nmol/L by World Bank income groups

Appendix 9: The prevalence of vitamin D deficiency by age

Study	Events	Total	Proportion	95%−CI	Weight (common)	Weight (random)
Saliba W	891	6518	0.14	[0.13; 0.15]	4.5%	1.3%
Djennane M	35	435	0.08	[0.06; 0.11]	0.3%	1.3%
Drali O	93	621	0.15	[0.12; 0.18]	0.4%	1.3%
Zhou SJ	9	221	0.04	[0.02; 0.08]	0.2%	1.3%
Sioen I	18	357	0.05	[0.03; 0.08]	0.2%	1.3%
Aucoin M	78	756	0.10	[0.08; 0.13]	0.5%	1.3%
El Hayek J	39	282	0.14	[0.10; 0.18]	0.2%	1.3%
El Hayek J	3	508	0.01	[0.00; 0.02]	0.4%	1.3%
Maguire JL	1	91	0.01	[0.00; 0.06]	0.1%	1.3%
Riverin B	3	52	0.06	[0.01; 0.16]	0.0%	1.2%
Science M	4	743	0.01	[0.00; 0.01]	0.5%	1.3%
Yousef S	5	267	0.02	[0.01; 0.04]	0.2%	1.3%
Yousef S	38	932	0.04	[0.03; 0.06]	0.6%	1.3%
Yousef S	90	858	0.10	[0.09; 0.13]	0.6%	1.3%
Li H	2040	10696	0.19	[0.18; 0.20]	7.4%	1.3%
Zhu Z	6	183	0.03	[0.01; 0.07]	0.1%	1.3%
Zhu Z	26	2269	0.01	[0.01; 0.02]	1.6%	1.3%
Zhu Z	29	1440	0.02	[0.01; 0.03]	1.0%	1.3%
Beer RJ	129	6813	0.02	[0.02; 0.02]	4.7%	1.3%
Beer RJ	194	6470	0.03	[0.03; 0.03]	4.5%	1.3%
Beer RJ	395	16454	0.02	[0.02; 0.03]	11.4%	1.3%
Karin Z	31	260	0.12	[0.08; 0.16]	0.2%	1.3%
Sochorová L	101	419	0.24	[0.20; 0.28]	0.3%	1.3%
Andersen R	30	37	0.81	[0.65; 0.92]	0.0%	1.2%
Andersen R	13	54	0.24	[0.13; 0.38]	0.0%	1.2%
Courraud J	31	177	0.18	[0.12; 0.24]	0.1%	1.3%
Hansen L	79	527	0.15	[0.12; 0.18]	0.4%	1.3%
Hansen L	6	75	0.08	[0.03; 0.17]	0.1%	1.3%
Hintzpeter B	1797	9001	0.20	[0.19; 0.21]	6.3%	1.3%
Kunz C	63	327	0.19	[0.15; 0.24]	0.2%	1.3%
Kunz C	106	528	0.20	[0.17; 0.24]	0.4%	1.3%
Kunz C	111	456	0.24	[0.20; 0.29]	0.3%	1.3%
Kunz C	112	520	0.22	[0.18; 0.25]	0.4%	1.3%
Manios Y	122	2353	0.05	[0.04; 0.06]	1.6%	1.3%
Bjarnadottir A	5	158	0.03	[0.01; 0.07]	0.1%	1.3%
Sharawat IK	29	100	0.29	[0.20; 0.39]	0.1%	1.3%
Nikooyeh B	374	667	0.56	[0.52; 0.60]	0.5%	1.3%
Qorbani M	276	2596	0.11	[0.09; 0.12]	1.8%	1.3%
Saki F	63	477	0.13	[0.10; 0.17]	0.3%	1.3%
Abdulrahman MA	16	50	0.32	[0.20; 0.47]	0.0%	1.2%
Ní Chaoimh C	12	741	0.02	[0.01; 0.03]	0.5%	1.3%
Ceccarelli M	209	2140	0.10	[0.09; 0.11]	1.5%	1.3%
Mallah EM	180	915	0.20	[0.17; 0.22]	0.6%	1.3%
Al−Taiar A	559	1416	0.39	[0.37; 0.42]	1.0%	1.3%
Alyahya K	169	232	0.73	[0.67; 0.78]	0.2%	1.3%
Bater J	5692	9595	0.59	[0.58; 0.60]	6.7%	1.3%
Uush T	679	1400	0.48	[0.46; 0.51]	1.0%	1.3%
Cairncross CT	86	1329	0.06	[0.05; 0.08]	0.9%	1.3%
Oberg J	147	890	0.17	[0.14; 0.19]	0.6%	1.3%
Chlebna−Sokó.. D	145	720	0.20	[0.17; 0.23]	0.5%	1.3%
Al Shaikh A	1687	3613	0.47	[0.45; 0.48]	2.5%	1.3%
Al Shaikh AM	61	204	0.30	[0.24; 0.37]	0.1%	1.3%
Al Shaikh AM	899	1906	0.47	[0.45; 0.49]	1.3%	1.3%
Al−Daghri NM	718	2225	0.32	[0.30; 0.34]	1.5%	1.3%
Al−Daghri NM	276	808	0.34	[0.31; 0.38]	0.6%	1.3%
Al−Daghri NM	1083	3111	0.35	[0.33; 0.37]	2.2%	1.3%
Al−Saleh Y	718	2226	0.32	[0.30; 0.34]	1.5%	1.3%
Kaddam IM	1979	4035	0.49	[0.47; 0.51]	2.8%	1.3%
Masoud MS	117	170	0.69	[0.61; 0.76]	0.1%	1.3%
Sulimani RA	645	1618	0.40	[0.37; 0.42]	1.1%	1.3%
Fernández Bustillo JM	9	153	0.06	[0.03; 0.11]	0.1%	1.3%
Rodríguez−Rodríguez E	8	102	0.08	[0.03; 0.15]	0.1%	1.3%
Jayatissa R	333	2525	0.13	[0.12; 0.15]	1.8%	1.3%
Nälsén C	10	206	0.05	[0.02; 0.09]	0.1%	1.3%
Voortman T	258	4167	0.06	[0.05; 0.07]	2.9%	1.3%
Bezrati I	92	225	0.41	[0.34; 0.48]	0.2%	1.3%
Hatun S	19	89	0.21	[0.13; 0.31]	0.1%	1.3%
Karagüzel G	75	746	0.10	[0.08; 0.12]	0.5%	1.3%
Glatt DU	3	49	0.06	[0.01; 0.17]	0.0%	1.2%
Tolppanen AM	124	7560	0.02	[0.01; 0.02]	5.3%	1.3%
NHANES(2015...2016)	26	2618	0.01	[0.01; 0.01]	1.8%	1.3%
Herrick KA	7	1438	0.00	[0.00; 0.01]	1.0%	1.3%
Herrick KA	29	2060	0.01	[0.01; 0.02]	1.4%	1.3%
Herrick KA	113	2355	0.05	[0.04; 0.06]	1.6%	1.3%
Mansbach JM	17	1799	0.01	[0.01; 0.02]	1.3%	1.3%
Mansbach JM	27	2759	0.01	[0.01; 0.01]	1.9%	1.3%
**Common effect model**		**143893**	**0.13**	**[0.13; 0.13]**	**100.0%**	**−−**
**Random effects model**			**0.15**	**[0.11; 0.19]**	**−−**	**100.0%**

Heterogeneity: $I^2 = 100\%$, $\tau^2 = 0.0588$, $p = 0$

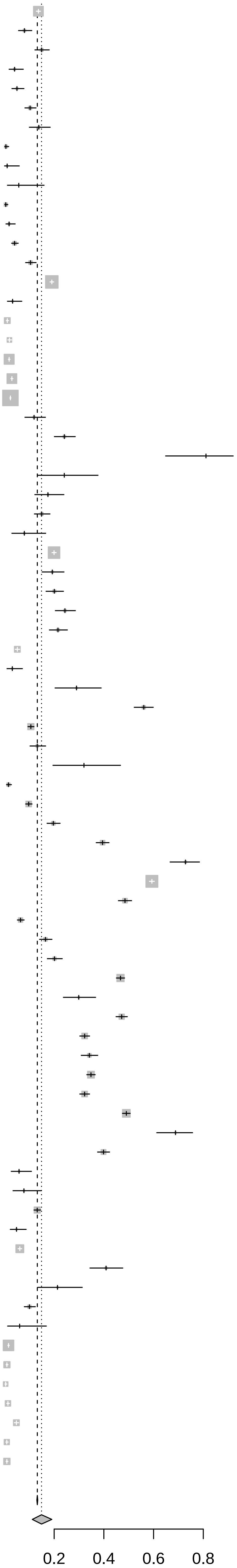

Supplementary figure 17 The prevalence of serum 25(OH)D < 30 nmol/L among people aged < 18

Appendix 9: The prevalence of vitamin D deficiency by age

Study	Events	Total	Proportion	95%−CI	Weight (common)	Weight (random)
Abdulrahman MA	50	278	0.18	[0.14; 0.23]	0.0%	3.2%
Al−Kindi MK	51	100	0.51	[0.41; 0.61]	0.0%	3.1%
Alyahya KO	88	104	0.85	[0.76; 0.91]	0.0%	3.2%
Aucoin M	96	461	0.21	[0.17; 0.25]	0.0%	3.2%
Black LJ	14	399	0.04	[0.02; 0.06]	0.0%	3.2%
Black LJ	28	730	0.04	[0.03; 0.05]	0.0%	3.2%
Black LJ	41	652	0.06	[0.05; 0.08]	0.0%	3.2%
Borissova AM	170	894	0.19	[0.16; 0.22]	0.0%	3.3%
Crowe FL	443448	1180086	0.38	[0.37; 0.38]	31.1%	3.3%
Crowe FL	531681	1316390	0.40	[0.40; 0.40]	34.7%	3.3%
Crowe FL	536113	1269812	0.42	[0.42; 0.42]	33.5%	3.3%
Daly RM	44	1393	0.03	[0.02; 0.04]	0.0%	3.3%
Daly RM	108	2556	0.04	[0.03; 0.05]	0.1%	3.3%
Duarte C	26	155	0.17	[0.11; 0.24]	0.0%	3.2%
Duarte C	81	355	0.23	[0.19; 0.28]	0.0%	3.2%
Ganmaa D	333	420	0.79	[0.75; 0.83]	0.0%	3.2%
Gebreegziabher T	29	196	0.15	[0.10; 0.21]	0.0%	3.2%
Granlund L	15	136	0.11	[0.06; 0.18]	0.0%	3.2%
Herrick KA	271	3564	0.08	[0.07; 0.09]	0.1%	3.3%
Madsen KH	2	198	0.01	[0.00; 0.04]	0.0%	3.2%
Mechenro J	46	275	0.17	[0.13; 0.22]	0.0%	3.2%
Nälsén C	3	44	0.07	[0.01; 0.19]	0.0%	3.0%
NHANES(2015...2016)	85	2413	0.04	[0.03; 0.04]	0.1%	3.3%
Nielsen NO	199	535	0.37	[0.33; 0.41]	0.0%	3.2%
Rabenberg M	756	2456	0.31	[0.29; 0.33]	0.1%	3.3%
Rabufetti A	24	1045	0.02	[0.01; 0.03]	0.0%	3.3%
Saeed BQ	48	287	0.17	[0.13; 0.22]	0.0%	3.2%
Sarafin K	132	1283	0.10	[0.09; 0.12]	0.0%	3.3%
Shchubelka K	23	702	0.03	[0.02; 0.05]	0.0%	3.2%
Sokolovic S	93	450	0.21	[0.17; 0.25]	0.0%	3.2%
Tønnesen R	135	700	0.19	[0.16; 0.22]	0.0%	3.2%
**Common effect model**		**3789069**	**0.40**	**[0.40; 0.40]**	**100.0%**	**−−**
**Random effects model**			**0.18**	**[0.12; 0.26]**	**−−**	**100.0%**

Heterogeneity: $I^2 = 100\%$, $\tau^2 = 0.0655$, $p = 0$

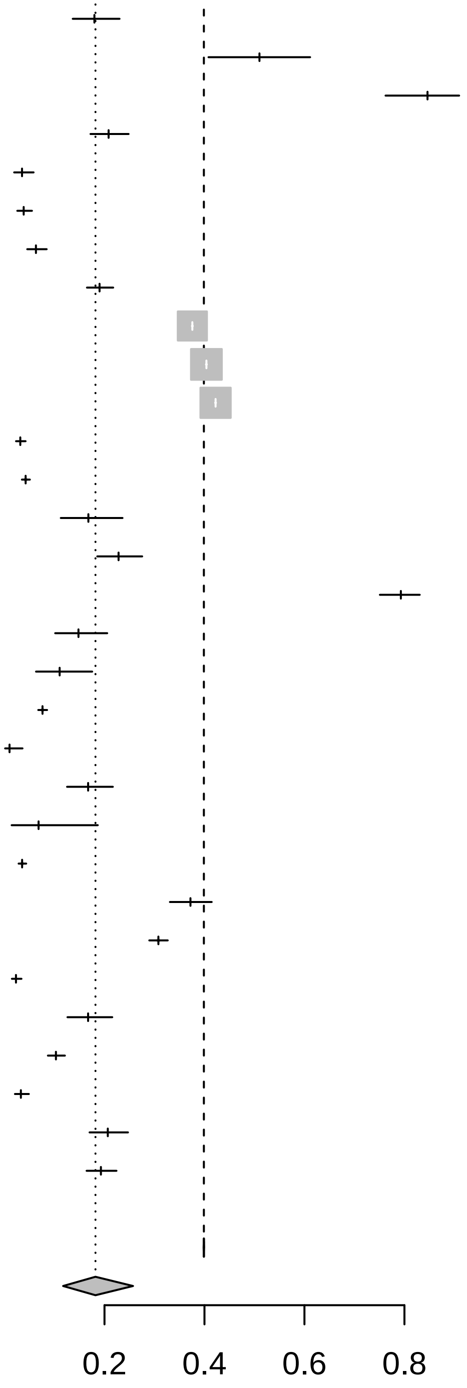

Supplementary figure 18 The prevalence of serum 25(OH)D < 30 nmol/L among people aged 19-44

Appendix 9: The prevalence of vitamin D deficiency by age

Study	Events	Total	Proportion	95%−CI	Weight (common)	Weight (random)
Black LJ	23	490	0.05	[0.03; 0.07]	0.0%	4.8%
Black LJ	31	648	0.05	[0.03; 0.07]	0.0%	4.8%
Daly RM	77	2011	0.04	[0.03; 0.05]	0.1%	4.8%
Daly RM	111	2884	0.04	[0.03; 0.05]	0.2%	4.8%
Tran B	11	148	0.07	[0.04; 0.13]	0.0%	4.6%
Moreno−Reyes R	135	401	0.34	[0.29; 0.39]	0.0%	4.8%
Sokolovic S	185	645	0.29	[0.25; 0.32]	0.0%	4.8%
Borissova AM	112	534	0.21	[0.18; 0.25]	0.0%	4.8%
Madsen KH	2	227	0.01	[0.00; 0.03]	0.0%	4.7%
Ikonen H	92	3650	0.03	[0.02; 0.03]	0.2%	4.8%
Rabenberg M	804	2723	0.30	[0.28; 0.31]	0.2%	4.8%
Rabenberg M	804	2723	0.30	[0.28; 0.31]	0.2%	4.8%
Mechenro J	41	101	0.41	[0.31; 0.51]	0.0%	4.5%
Duarte C	113	608	0.19	[0.16; 0.22]	0.0%	4.8%
Duarte C	149	723	0.21	[0.18; 0.24]	0.0%	4.8%
Granlund L	11	80	0.14	[0.07; 0.23]	0.0%	4.5%
Nälsén C	5	96	0.05	[0.02; 0.12]	0.0%	4.5%
Crowe FL	223186	755174	0.30	[0.29; 0.30]	45.0%	4.9%
Crowe FL	300265	901352	0.33	[0.33; 0.33]	53.7%	4.9%
Shchubelka K	24	412	0.06	[0.04; 0.09]	0.0%	4.8%
NHANES(2015...2016)	37	1777	0.02	[0.01; 0.03]	0.1%	4.8%
**Common effect model**		**1677407**	**0.31**	**[0.31; 0.31]**	**100.0%**	**−−**
**Random effects model**			**0.14**	**[0.09; 0.20]**	**−−**	**100.0%**

Heterogeneity: I^2 = 100%, τ^2 = 0.0355, p = 0

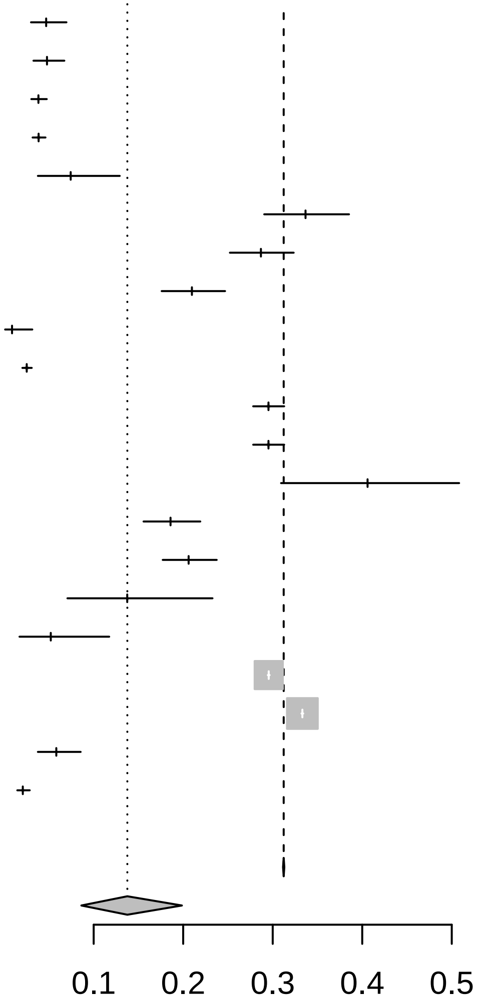

Supplementary figure 19 The prevalence of serum 25(OH)D < 30 nmol/L among people aged 45-64

Appendix 9: The prevalence of vitamin D deficiency by age

Study	Events	Total	Proportion	95%−CI	Weight (common)	Weight (random)
Oliveri B	35	339	0.10	[0.07; 0.14]	0.0%	2.9%
Saraiva GL	33	214	0.15	[0.11; 0.21]	0.0%	2.8%
Scalco R	53	98	0.54	[0.44; 0.64]	0.0%	2.7%
Pérez−Llamas F	28	86	0.33	[0.23; 0.44]	0.0%	2.7%
Orwoll E	47	1606	0.03	[0.02; 0.04]	0.2%	2.9%
Dalgård C	124	669	0.19	[0.16; 0.22]	0.1%	2.9%
Greene−Finestone LS	8	312	0.03	[0.01; 0.05]	0.0%	2.9%
Daly RM	66	810	0.08	[0.06; 0.10]	0.1%	2.9%
Daly RM	75	1564	0.05	[0.04; 0.06]	0.2%	2.9%
Hirani V	292	2070	0.14	[0.13; 0.16]	0.2%	2.9%
Hirani V	27	174	0.16	[0.10; 0.22]	0.0%	2.8%
Hirani V	30	304	0.10	[0.07; 0.14]	0.0%	2.9%
Hirani V	45	522	0.09	[0.06; 0.11]	0.1%	2.9%
Hirani V	57	659	0.09	[0.07; 0.11]	0.1%	2.9%
Robinson PJ	37	267	0.14	[0.10; 0.19]	0.0%	2.9%
Robinson PJ	74	640	0.12	[0.09; 0.14]	0.1%	2.9%
Tran B	9	134	0.07	[0.03; 0.12]	0.0%	2.8%
Tran B	11	148	0.07	[0.04; 0.13]	0.0%	2.8%
Tran B	15	131	0.11	[0.07; 0.18]	0.0%	2.8%
Tran B	20	92	0.22	[0.14; 0.32]	0.0%	2.7%
Matheï C	129	367	0.35	[0.30; 0.40]	0.0%	2.9%
Andersen R	8	52	0.15	[0.07; 0.28]	0.0%	2.5%
Samefors M	48	333	0.14	[0.11; 0.19]	0.0%	2.9%
Cougnard−Grégoire A	190	697	0.27	[0.24; 0.31]	0.1%	2.9%
Rabenberg M	540	1816	0.30	[0.28; 0.32]	0.2%	2.9%
Santos A	594	1500	0.40	[0.37; 0.42]	0.1%	2.9%
Wyskida M	355	3472	0.10	[0.09; 0.11]	0.3%	3.0%
Solis−Urra P	181	686	0.26	[0.23; 0.30]	0.1%	2.9%
Crowe FL	299730	993895	0.30	[0.30; 0.30]	97.7%	3.0%
Duarte C	65	249	0.26	[0.21; 0.32]	0.0%	2.8%
Duarte C	91	304	0.30	[0.25; 0.35]	0.0%	2.9%
Hribar M	36	155	0.23	[0.17; 0.31]	0.0%	2.8%
Black LJ	15	331	0.05	[0.03; 0.07]	0.0%	2.9%
Yousef S	63	1361	0.05	[0.04; 0.06]	0.1%	2.9%
NHANES(2015...2016)	23	1231	0.02	[0.01; 0.03]	0.1%	2.9%
**Common effect model**		**1017288**	**0.30**	**[0.30; 0.30]**	**100.0%**	**−−**
**Random effects model**			**0.15**	**[0.12; 0.19]**	**−−**	**100.0%**

Heterogeneity: $I^2 = 99\%$, $\tau^2 = 0.0253$, $p = 0$

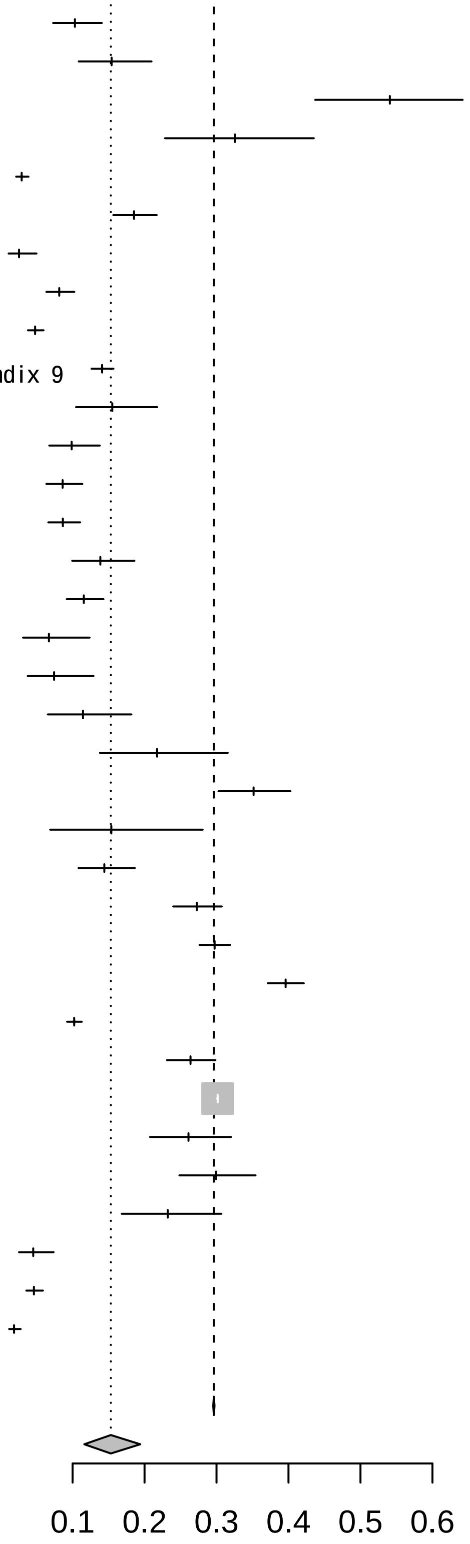

Supplementary figure 20 The prevalence of serum 25(OH)D < 30 nmol/L among people aged > 65

Appendix 9: The prevalence of vitamin D deficiency by age

Study	Events	Total	Proportion	95%−CI	Weight (common)	Weight (random)
Gordon CM	129	307	0.42	[0.36; 0.48]	0.2%	0.9%
Moussavi M	147	318	0.46	[0.41; 0.52]	0.2%	0.9%
Hatun S	39	89	0.44	[0.33; 0.55]	0.1%	0.9%
Harkness LS	200	370	0.54	[0.49; 0.59]	0.2%	0.9%
Andersen R	35	37	0.95	[0.82; 0.99]	0.0%	0.9%
Hintzpeter B	5895	9001	0.65	[0.65; 0.66]	5.8%	0.9%
Bener A	315	458	0.69	[0.64; 0.73]	0.3%	0.9%
Mansbach JM	268	1799	0.15	[0.13; 0.17]	1.2%	0.9%
Mansbach JM	578	2759	0.21	[0.19; 0.23]	1.8%	0.9%
Gilbert−Diamond D	208	479	0.43	[0.39; 0.48]	0.3%	0.9%
Maguire JL	29	91	0.32	[0.22; 0.42]	0.1%	0.9%
Mallah EM	517	915	0.57	[0.53; 0.60]	0.6%	0.9%
Rodríguez−Rodríguez E	52	102	0.51	[0.41; 0.61]	0.1%	0.9%
Saliba W	3014	6518	0.46	[0.45; 0.47]	4.2%	0.9%
Sioen I	207	357	0.58	[0.53; 0.63]	0.2%	0.9%
Santos BR	85	234	0.36	[0.30; 0.43]	0.2%	0.9%
Zhu Z	85	183	0.46	[0.39; 0.54]	0.1%	0.9%
Zhu Z	498	2269	0.22	[0.20; 0.24]	1.5%	0.9%
Zhu Z	582	1440	0.40	[0.38; 0.43]	0.9%	0.9%
Tolppanen AM	2158	7560	0.29	[0.28; 0.30]	4.9%	0.9%
Aucoin M	318	756	0.42	[0.39; 0.46]	0.5%	0.9%
El Hayek J	54	508	0.11	[0.08; 0.14]	0.3%	0.9%
Andersen R	47	54	0.87	[0.75; 0.95]	0.0%	0.9%
Kouda K	26	400	0.06	[0.04; 0.09]	0.3%	0.9%
Muhairi SJ	143	315	0.45	[0.40; 0.51]	0.2%	0.9%
Djennane M	130	435	0.30	[0.26; 0.34]	0.3%	0.9%
Riverin B	22	52	0.42	[0.29; 0.57]	0.0%	0.9%
Madsen KH	24	340	0.07	[0.05; 0.10]	0.2%	0.9%
Bjarnadottir A	103	158	0.65	[0.57; 0.73]	0.1%	0.9%
Rafraf M	207	216	0.96	[0.92; 0.98]	0.1%	0.9%
Chung IH	1805	2314	0.78	[0.76; 0.80]	1.5%	0.9%
Alyahya K	229	232	0.99	[0.96; 1.00]	0.1%	0.9%
Karagüzel G	265	746	0.36	[0.32; 0.39]	0.5%	0.9%
Zhou SJ	44	221	0.20	[0.15; 0.26]	0.1%	0.9%
Sarafin K	498	1579	0.32	[0.29; 0.34]	1.0%	0.9%
Shady MM	23	200	0.12	[0.07; 0.17]	0.1%	0.9%
Wakayo T	48	77	0.62	[0.51; 0.73]	0.0%	0.9%
Hazell TJ	57	488	0.12	[0.09; 0.15]	0.3%	0.9%
Al−Daghri NM	1901	2225	0.85	[0.84; 0.87]	1.4%	0.9%
Al−Saleh Y	1187	2226	0.53	[0.51; 0.55]	1.4%	0.9%
Voortman T	1241	4167	0.30	[0.28; 0.31]	2.7%	0.9%
Smith G	3	41	0.07	[0.02; 0.20]	0.0%	0.9%
Al Shaikh AM	184	204	0.90	[0.85; 0.94]	0.1%	0.9%
Al Shaikh AM	1829	1906	0.96	[0.95; 0.97]	1.2%	0.9%
Al−Daghri NM	711	808	0.88	[0.86; 0.90]	0.5%	0.9%
Sulimani RA	1191	1618	0.74	[0.71; 0.76]	1.0%	0.9%
Bezrati I	191	225	0.85	[0.80; 0.89]	0.1%	0.9%
Science M	152	743	0.20	[0.18; 0.24]	0.5%	0.9%
Manios Y	1235	2353	0.52	[0.50; 0.55]	1.5%	0.9%
Naqvi A	11	86	0.13	[0.07; 0.22]	0.1%	0.9%
Sharawat IK	63	100	0.63	[0.53; 0.72]	0.1%	0.9%
Nikooyeh B	622	667	0.93	[0.91; 0.95]	0.4%	0.9%
Saki F	388	477	0.81	[0.78; 0.85]	0.3%	0.9%
Byun EJ	1843	2515	0.73	[0.72; 0.75]	1.6%	0.9%
Rahmadhani R	410	1011	0.41	[0.38; 0.44]	0.7%	0.9%
Cairncross CT	642	1329	0.48	[0.46; 0.51]	0.9%	0.9%
Karin Z	151	260	0.58	[0.52; 0.64]	0.2%	0.9%
Sochorová L	252	419	0.60	[0.55; 0.65]	0.3%	0.9%
Hansen L	321	527	0.61	[0.57; 0.65]	0.3%	0.9%
Kapil U	307	374	0.82	[0.78; 0.86]	0.2%	0.9%
Kapil U	618	848	0.73	[0.70; 0.76]	0.5%	0.9%
Ní Chaoimh C	198	741	0.27	[0.24; 0.30]	0.5%	0.9%
Al−Taiar A	1140	1416	0.81	[0.78; 0.83]	0.9%	0.9%
Oberg J	536	890	0.60	[0.57; 0.63]	0.6%	0.9%
Fernández Bustillo JM	101	153	0.66	[0.58; 0.73]	0.1%	0.9%
Hansen L	31	75	0.41	[0.30; 0.53]	0.0%	0.9%
Bodin J	85	95	0.89	[0.81; 0.95]	0.1%	0.9%
Kunz C	205	327	0.63	[0.57; 0.68]	0.2%	0.9%
Kunz C	283	456	0.62	[0.57; 0.67]	0.3%	0.9%
Kunz C	318	520	0.61	[0.57; 0.65]	0.3%	0.9%
Kunz C	325	528	0.62	[0.57; 0.66]	0.3%	0.9%
Houghton LA	2	433	0.00	[0.00; 0.02]	0.3%	0.9%
Benjeddou K	114	239	0.48	[0.41; 0.54]	0.2%	0.9%
Chlebna−Sokó.. D	606	720	0.84	[0.81; 0.87]	0.5%	0.9%
White Z	4	59	0.07	[0.02; 0.16]	0.0%	0.9%
Jayatissa R	1484	2525	0.59	[0.57; 0.61]	1.6%	0.9%
Herrick KA	102	1438	0.07	[0.06; 0.09]	0.9%	0.9%
Herrick KA	282	2060	0.14	[0.12; 0.15]	1.3%	0.9%
Herrick KA	648	2355	0.28	[0.26; 0.29]	1.5%	0.9%
Isa H	416	531	0.78	[0.75; 0.82]	0.3%	0.9%
de Oliveira CL	298	1152	0.26	[0.23; 0.28]	0.7%	0.9%
Li H	7985	10696	0.75	[0.74; 0.75]	6.9%	0.9%
Yu L	40	1200	0.03	[0.02; 0.05]	0.8%	0.9%
Yu L	87	1200	0.07	[0.06; 0.09]	0.8%	0.9%
Beer RJ	1346	6470	0.21	[0.20; 0.22]	4.2%	0.9%
Beer RJ	1887	6813	0.28	[0.27; 0.29]	4.4%	0.9%
Beer RJ	3587	16454	0.22	[0.21; 0.22]	10.6%	0.9%
Courraud J	121	177	0.68	[0.61; 0.75]	0.1%	0.9%
Ceccarelli M	939	2140	0.44	[0.42; 0.46]	1.4%	0.9%
Rahman A	352	410	0.86	[0.82; 0.89]	0.3%	0.9%
Al Shaikh A	3289	3613	0.91	[0.90; 0.92]	2.3%	0.9%
Masoud MS	170	170	1.00	[0.98; 1.00]	0.1%	0.9%
Nälsén C	87	206	0.42	[0.35; 0.49]	0.1%	0.9%
Mutua AM	8	302	0.03	[0.01; 0.05]	0.2%	0.9%
Drali O	375	621	0.60	[0.56; 0.64]	0.4%	0.9%
Horton−French K	112	692	0.16	[0.14; 0.19]	0.4%	0.9%
Yousef S	42	267	0.16	[0.12; 0.21]	0.2%	0.9%
Yousef S	231	932	0.25	[0.22; 0.28]	0.6%	0.9%
Yousef S	346	858	0.40	[0.37; 0.44]	0.6%	0.9%
Sherief LM	542	572	0.95	[0.93; 0.96]	0.4%	0.9%
Feketea GM	102	376	0.27	[0.23; 0.32]	0.2%	0.9%
Koyama S	138	492	0.28	[0.24; 0.32]	0.3%	0.9%
Mogire RM	111	1507	0.07	[0.06; 0.09]	1.0%	0.9%
Flores ME	208	713	0.29	[0.26; 0.33]	0.5%	0.9%
Flores ME	599	3482	0.17	[0.16; 0.18]	2.2%	0.9%
Al−Daghri NM	2609	3111	0.84	[0.83; 0.85]	2.0%	0.9%
Metwally ASM	1692	1864	0.91	[0.89; 0.92]	1.2%	0.9%
Abdulrahman MA	30	50	0.60	[0.45; 0.74]	0.0%	0.9%
Glatt DU	24	49	0.49	[0.34; 0.64]	0.0%	0.9%
NHANES(2015...2016)	500	2618	0.19	[0.18; 0.21]	1.7%	0.9%
Chung IH	710	1212	0.59	[0.56; 0.61]	0.8%	0.9%
**Common effect model**		**155486**	**0.45**	**[0.44; 0.45]**	**100.0%**	**−−**
**Random effects model**			**0.49**	**[0.43; 0.55]**	**−−**	**100.0%**

Heterogeneity: $I^2 = 100\%$, $\tau^2 = 0.1041$, $p = 0$

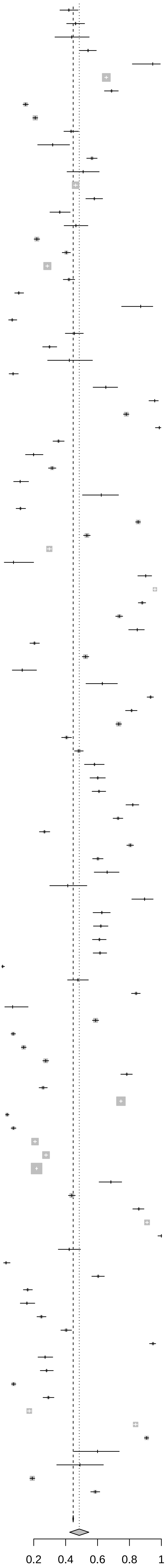

Supplementary figure 21 The prevalence of serum 25(OH)D < 50 nmol/L among people aged < 18

Appendix 9: The prevalence of vitamin D deficiency by age

Study	Events	Total	Proportion	95%−CI	Weight (common)	Weight (random)
Zargar AH	76	92	0.83	[0.73; 0.90]	0.2%	2.4%
Ramakrishnan S	74	237	0.31	[0.25; 0.38]	0.6%	2.4%
Shivane VK	805	1137	0.71	[0.68; 0.73]	2.8%	2.5%
Al−Kindi MK	100	100	1.00	[0.96; 1.00]	0.3%	2.4%
Chailurkit LO	151	2641	0.06	[0.05; 0.07]	6.6%	2.5%
Daly RM	308	1393	0.22	[0.20; 0.24]	3.5%	2.5%
Daly RM	762	2556	0.30	[0.28; 0.32]	6.4%	2.5%
Aucoin M	279	461	0.61	[0.56; 0.65]	1.2%	2.4%
Gebreegziabher T	165	196	0.84	[0.78; 0.89]	0.5%	2.4%
Gill TK	107	500	0.21	[0.18; 0.25]	1.2%	2.5%
Gill TK	110	510	0.22	[0.18; 0.25]	1.3%	2.5%
Guo S	34	100	0.34	[0.25; 0.44]	0.3%	2.4%
Madsen KH	15	198	0.08	[0.04; 0.12]	0.5%	2.4%
Nielsen NO	397	535	0.74	[0.70; 0.78]	1.3%	2.5%
Ganmaa D	415	420	0.99	[0.97; 1.00]	1.0%	2.4%
Sarafin K	550	1283	0.43	[0.40; 0.46]	3.2%	2.5%
Rabenberg M	1439	2456	0.59	[0.57; 0.61]	6.1%	2.5%
Junaid K	156	215	0.73	[0.66; 0.78]	0.5%	2.4%
Tønnesen R	373	700	0.53	[0.50; 0.57]	1.7%	2.5%
Mahmood S	47	80	0.59	[0.47; 0.70]	0.2%	2.4%
Sokolovic S	179	450	0.40	[0.35; 0.44]	1.1%	2.4%
Leung RY	290	464	0.62	[0.58; 0.67]	1.2%	2.4%
Leung RY	459	791	0.58	[0.55; 0.61]	2.0%	2.5%
Mechenro J	162	275	0.59	[0.53; 0.65]	0.7%	2.4%
Nadeem S	197	221	0.89	[0.84; 0.93]	0.6%	2.4%
Liu X	4200	10343	0.41	[0.40; 0.42]	25.8%	2.5%
Rabufetti A	179	1045	0.17	[0.15; 0.20]	2.6%	2.5%
Herrick KA	1119	3564	0.31	[0.30; 0.33]	8.9%	2.5%
Alyahya KO	96	104	0.92	[0.85; 0.97]	0.3%	2.4%
Duarte C	87	155	0.56	[0.48; 0.64]	0.4%	2.4%
Duarte C	236	355	0.66	[0.61; 0.71]	0.9%	2.4%
Nälsén C	8	44	0.18	[0.08; 0.33]	0.1%	2.3%
Shchubelka K	249	702	0.35	[0.32; 0.39]	1.8%	2.5%
Black LJ	99	399	0.25	[0.21; 0.29]	1.0%	2.4%
Black LJ	166	652	0.25	[0.22; 0.29]	1.6%	2.5%
Black LJ	192	730	0.26	[0.23; 0.30]	1.8%	2.5%
Horton−French K	112	400	0.28	[0.24; 0.33]	1.0%	2.4%
Contreras−Manzano A	120	381	0.31	[0.27; 0.36]	1.0%	2.4%
Contreras−Manzano A	150	477	0.31	[0.27; 0.36]	1.2%	2.4%
Abdulrahman MA	112	278	0.40	[0.34; 0.46]	0.7%	2.4%
NHANES(2015...2016)	753	2413	0.31	[0.29; 0.33]	6.0%	2.5%
**Common effect model**		**40053**	**0.38**	**[0.38; 0.39]**	**100.0%**	**−−**
**Random effects model**			**0.48**	**[0.39; 0.57]**	**−−**	**100.0%**

Heterogeneity: $I^2 = 99\%$, $\tau^2 = 0.0871$, $p = 0$

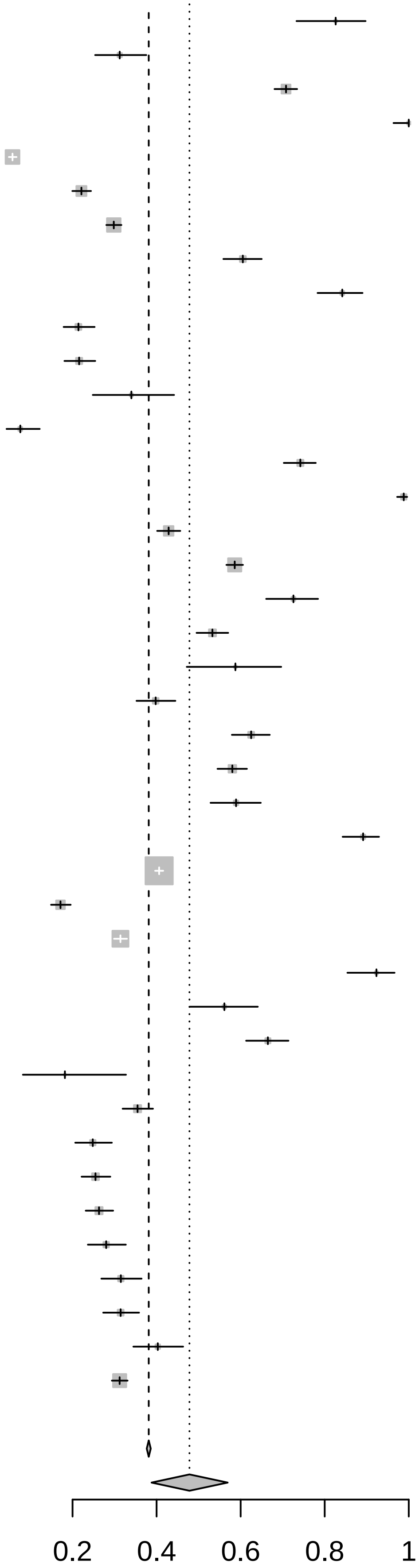

Supplementary figure 22 The prevalence of serum 25(OH)D < 50 nmol/L among people aged 19-44

Appendix 9: The prevalence of vitamin D deficiency by age

Study	Events	Total	Proportion	95%−CI	Weight (common)	Weight (random)
Moreno−Reyes R	306	401	0.76	[0.72; 0.80]	1.7%	4.8%
Daly RM	732	2011	0.36	[0.34; 0.39]	8.6%	4.8%
Daly RM	983	2884	0.34	[0.32; 0.36]	12.3%	4.8%
Tran B	107	148	0.72	[0.64; 0.79]	0.6%	4.7%
Seo JA	856	1081	0.79	[0.77; 0.82]	4.6%	4.8%
Gill TK	83	390	0.21	[0.17; 0.26]	1.7%	4.8%
Gill TK	130	486	0.27	[0.23; 0.31]	2.1%	4.8%
Madsen KH	28	227	0.12	[0.08; 0.17]	1.0%	4.7%
Rabenberg M	1700	2723	0.62	[0.61; 0.64]	11.6%	4.8%
Rabenberg M	1700	2723	0.62	[0.61; 0.64]	11.6%	4.8%
Sokolovic S	357	645	0.55	[0.51; 0.59]	2.8%	4.8%
Leung RY	397	1211	0.33	[0.30; 0.36]	5.2%	4.8%
Mechenro J	92	101	0.91	[0.84; 0.96]	0.4%	4.6%
Duarte C	383	608	0.63	[0.59; 0.67]	2.6%	4.8%
Duarte C	506	723	0.70	[0.66; 0.73]	3.1%	4.8%
Nälsén C	27	96	0.28	[0.19; 0.38]	0.4%	4.6%
Shchubelka K	182	412	0.44	[0.39; 0.49]	1.8%	4.8%
Black LJ	147	490	0.30	[0.26; 0.34]	2.1%	4.8%
Black LJ	194	648	0.30	[0.26; 0.34]	2.8%	4.8%
Ikonen H	857	3650	0.23	[0.22; 0.25]	15.6%	4.8%
NHANES(2015...2016)	332	1777	0.19	[0.17; 0.21]	7.6%	4.8%
**Common effect model**		**23435**	**0.43**	**[0.42; 0.43]**	**100.0%**	**−−**
**Random effects model**			**0.46**	**[0.36; 0.57]**	**−−**	**100.0%**

Heterogeneity: $I^2 = 99\%$, $\tau^2 = 0.0612$, $p = 0$

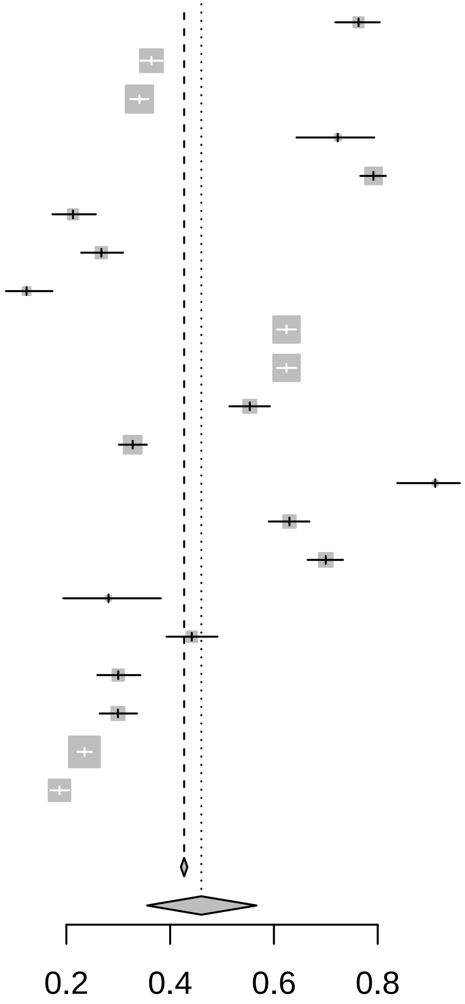

Supplementary figure 23 The prevalence of serum 25(OH)D < 50 nmol/L among people aged 45-64

Appendix 9: The prevalence of vitamin D deficiency by age

Study	Events	Total	Proportion	95%−CI	Weight (common)	Weight (random)
Oliveri B	229	339	0.68	[0.62; 0.73]	1.3%	2.3%
Black LJ	96	331	0.29	[0.24; 0.34]	1.3%	2.3%
Daly RM	354	810	0.44	[0.40; 0.47]	3.1%	2.3%
Daly RM	545	1564	0.35	[0.32; 0.37]	6.1%	2.3%
Gill TK	54	263	0.21	[0.16; 0.26]	1.0%	2.3%
Gill TK	66	265	0.25	[0.20; 0.31]	1.0%	2.3%
Hirani V	85	174	0.49	[0.41; 0.57]	0.7%	2.3%
Hirani V	124	304	0.41	[0.35; 0.47]	1.2%	2.3%
Hirani V	217	522	0.42	[0.37; 0.46]	2.0%	2.3%
Hirani V	287	659	0.44	[0.40; 0.47]	2.6%	2.3%
Robinson PJ	164	267	0.61	[0.55; 0.67]	1.0%	2.3%
Robinson PJ	337	640	0.53	[0.49; 0.57]	2.5%	2.3%
Tran B	76	92	0.83	[0.73; 0.90]	0.4%	2.2%
Tran B	92	134	0.69	[0.60; 0.76]	0.5%	2.2%
Tran B	102	131	0.78	[0.70; 0.85]	0.5%	2.2%
Tran B	106	148	0.72	[0.64; 0.79]	0.6%	2.2%
Matheï C	248	367	0.68	[0.63; 0.72]	1.4%	2.3%
Saraiva GL	123	214	0.57	[0.51; 0.64]	0.8%	2.3%
Scalco R	84	98	0.86	[0.77; 0.92]	0.4%	2.2%
Greene−Finestone LS	61	312	0.20	[0.15; 0.24]	1.2%	2.3%
Sarafin K	153	671	0.23	[0.20; 0.26]	2.6%	2.3%
Yousef S	305	1361	0.22	[0.20; 0.25]	5.3%	2.3%
Solis−Urra P	445	686	0.65	[0.61; 0.68]	2.7%	2.3%
Andersen R	25	52	0.48	[0.34; 0.62]	0.2%	2.2%
Dalgård C	359	669	0.54	[0.50; 0.57]	2.6%	2.3%
Orces CH	115	403	0.29	[0.24; 0.33]	1.6%	2.3%
Orces CH	162	724	0.22	[0.19; 0.26]	2.8%	2.3%
Cougnard−Grégoire A	580	697	0.83	[0.80; 0.86]	2.7%	2.3%
Klenk J	228	1418	0.16	[0.14; 0.18]	5.5%	2.3%
Rabenberg M	1201	1816	0.66	[0.64; 0.68]	7.0%	2.3%
Leung RY	400	899	0.44	[0.41; 0.48]	3.5%	2.3%
Duarte C	174	249	0.70	[0.64; 0.76]	1.0%	2.3%
Duarte C	252	304	0.83	[0.78; 0.87]	1.2%	2.3%
Santos A	1035	1500	0.69	[0.67; 0.71]	5.8%	2.3%
Miljkovic I	12	424	0.03	[0.01; 0.05]	1.6%	2.3%
Hribar M	98	155	0.63	[0.55; 0.71]	0.6%	2.3%
Pérez−Llamas F	50	86	0.58	[0.47; 0.69]	0.3%	2.2%
Nälsén C	11	66	0.17	[0.09; 0.28]	0.3%	2.2%
Samefors M	267	333	0.80	[0.75; 0.84]	1.3%	2.3%
Ten Haaf DSM	9	450	0.02	[0.01; 0.04]	1.7%	2.3%
Hirani V	968	2070	0.47	[0.45; 0.49]	8.0%	2.3%
Johnson MA	105	317	0.33	[0.28; 0.39]	1.2%	2.3%
NHANES(2015...2016)	170	1231	0.14	[0.12; 0.16]	4.8%	2.3%
Orwoll E	413	1606	0.26	[0.24; 0.28]	6.2%	2.3%
**Common effect model**		**25821**	**0.42**	**[0.41; 0.42]**	**100.0%**	**−−**
**Random effects model**			**0.46**	**[0.39; 0.54]**	**−−**	**100.0%**

Heterogeneity: $I^2 = 99\%$, $\tau^2 = 0.0682$, $p = 0$

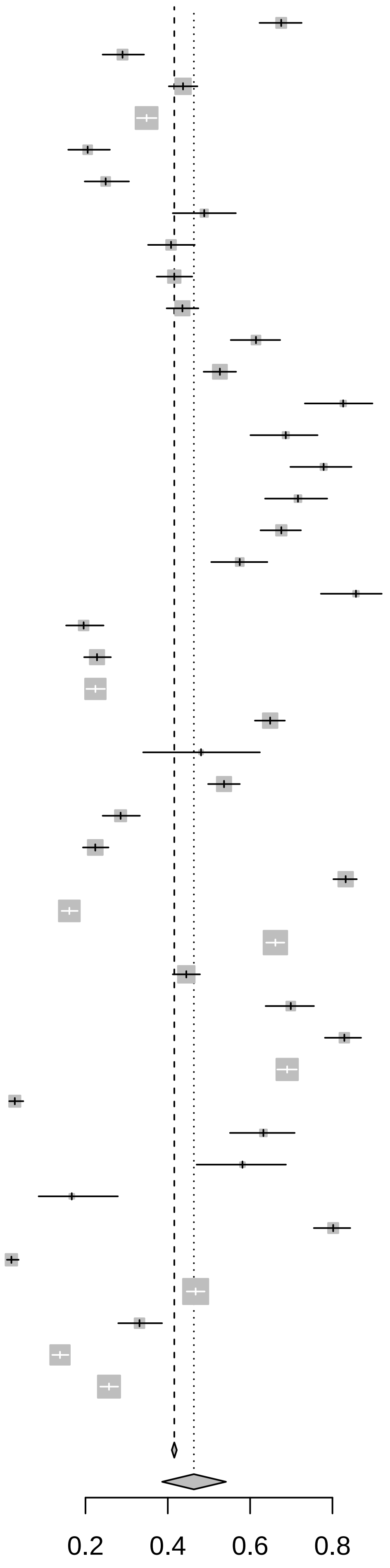

Supplementary figure 24 The prevalence of serum 25(OH)D < 50 nmol/L among people aged > 65

Appendix 9: The prevalence of vitamin D deficiency by age

Study	Events	Total	Proportion	95%−CI	Weight (common)	Weight (random)
Hintzpeter B	7911	9001	0.88	[0.87; 0.89]	11.8%	1.6%
Mansbach JM	1131	1799	0.63	[0.61; 0.65]	2.4%	1.6%
Mansbach JM	2013	2759	0.73	[0.71; 0.75]	3.6%	1.6%
Gilbert−Diamond D	430	479	0.90	[0.87; 0.92]	0.6%	1.6%
Maguire JL	75	91	0.82	[0.73; 0.90]	0.1%	1.6%
Saliba W	5237	6518	0.80	[0.79; 0.81]	8.5%	1.6%
Sioen I	350	357	0.98	[0.96; 0.99]	0.5%	1.6%
Santos BR	212	234	0.91	[0.86; 0.94]	0.3%	1.6%
Zhu Z	164	183	0.90	[0.84; 0.94]	0.2%	1.6%
Zhu Z	1271	1440	0.88	[0.86; 0.90]	1.9%	1.6%
Zhu Z	1555	2269	0.69	[0.67; 0.70]	3.0%	1.6%
Tolppanen AM	5631	7560	0.74	[0.73; 0.75]	9.9%	1.6%
Aucoin M	617	756	0.82	[0.79; 0.84]	1.0%	1.6%
El Hayek J	257	508	0.51	[0.46; 0.55]	0.7%	1.6%
Kouda K	198	400	0.50	[0.44; 0.55]	0.5%	1.6%
Djennane M	254	435	0.58	[0.54; 0.63]	0.6%	1.6%
Riverin B	41	52	0.79	[0.65; 0.89]	0.1%	1.5%
Madsen KH	197	340	0.58	[0.52; 0.63]	0.4%	1.6%
Karagüzel G	350	746	0.47	[0.43; 0.51]	1.0%	1.6%
Hazell TJ	203	488	0.42	[0.37; 0.46]	0.6%	1.6%
Voortman T	3020	4167	0.72	[0.71; 0.74]	5.4%	1.6%
Smith G	4	41	0.10	[0.03; 0.23]	0.1%	1.5%
Science M	565	743	0.76	[0.73; 0.79]	1.0%	1.6%
Manios Y	2266	2353	0.96	[0.95; 0.97]	3.1%	1.6%
Naqvi A	55	86	0.64	[0.53; 0.74]	0.1%	1.5%
Saki F	458	477	0.96	[0.94; 0.98]	0.6%	1.6%
Byun EJ	2456	2515	0.98	[0.97; 0.98]	3.3%	1.6%
Cairncross CT	1183	1329	0.89	[0.87; 0.91]	1.7%	1.6%
Karin Z	226	260	0.87	[0.82; 0.91]	0.3%	1.6%
Hansen L	482	527	0.91	[0.89; 0.94]	0.7%	1.6%
Kapil U	366	374	0.98	[0.96; 0.99]	0.5%	1.6%
Kapil U	767	848	0.90	[0.88; 0.92]	1.1%	1.6%
Ní Chaoimh C	530	741	0.72	[0.68; 0.75]	1.0%	1.6%
Al−Taiar A	1365	1416	0.96	[0.95; 0.97]	1.9%	1.6%
Oberg J	780	890	0.88	[0.85; 0.90]	1.2%	1.6%
Hansen L	56	75	0.75	[0.63; 0.84]	0.1%	1.5%
Kunz C	400	456	0.88	[0.84; 0.91]	0.6%	1.6%
Kunz C	469	528	0.89	[0.86; 0.91]	0.7%	1.6%
Kunz C	453	520	0.87	[0.84; 0.90]	0.7%	1.6%
Kunz C	241	327	0.74	[0.69; 0.78]	0.4%	1.6%
White Z	39	59	0.66	[0.53; 0.78]	0.1%	1.5%
Isa H	496	531	0.93	[0.91; 0.95]	0.7%	1.6%
de Oliveira CL	777	1152	0.67	[0.65; 0.70]	1.5%	1.6%
Yu L	832	1200	0.69	[0.67; 0.72]	1.6%	1.6%
Yu L	407	1200	0.34	[0.31; 0.37]	1.6%	1.6%
Ceccarelli M	1657	2140	0.77	[0.76; 0.79]	2.8%	1.6%
Rahman A	393	410	0.96	[0.93; 0.98]	0.5%	1.6%
Mutua AM	113	302	0.37	[0.32; 0.43]	0.4%	1.6%
Drali O	528	621	0.85	[0.82; 0.88]	0.8%	1.6%
Horton−French K	445	692	0.64	[0.61; 0.68]	0.9%	1.6%
Yousef S	179	267	0.67	[0.61; 0.73]	0.3%	1.6%
Yousef S	723	858	0.84	[0.82; 0.87]	1.1%	1.6%
Yousef S	727	932	0.78	[0.75; 0.81]	1.2%	1.6%
Sherief LM	566	572	0.99	[0.98; 1.00]	0.7%	1.6%
Feketea GM	249	376	0.66	[0.61; 0.71]	0.5%	1.6%
Koyama S	486	492	0.99	[0.97; 1.00]	0.6%	1.6%
Mogire RM	780	1507	0.52	[0.49; 0.54]	2.0%	1.6%
Flores ME	520	713	0.73	[0.70; 0.76]	0.9%	1.6%
Flores ME	2612	3482	0.75	[0.74; 0.76]	4.6%	1.6%
Abdulrahman MA	40	50	0.80	[0.66; 0.90]	0.1%	1.5%
NHANES(2015...2016)	1833	2618	0.70	[0.68; 0.72]	3.4%	1.6%
Chung IH	1177	1212	0.97	[0.96; 0.98]	1.6%	1.6%
**Common effect model**		**76474**	**0.80**	**[0.80; 0.80]**	**100.0%**	**−−**
**Random effects model**			**0.79**	**[0.74; 0.83]**	**−−**	**100.0%**

Heterogeneity: $I^2 = 99\%$, $\tau^2 = 0.0500$, $p = 0$

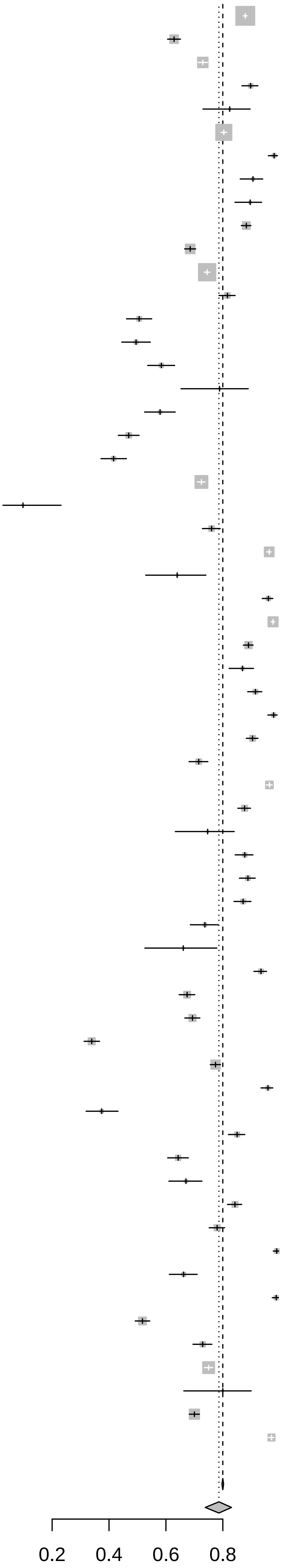

Supplementary figure 25 The prevalence of serum 25(OH)D < 75 nmol/L among people aged < 18

Appendix 9: The prevalence of vitamin D deficiency by age

Study	Events	Total	Proportion	95%−CI	Weight (common)	Weight (random)
Black LJ	250	399	0.63	[0.58; 0.67]	1.3%	3.7%
Black LJ	534	730	0.73	[0.70; 0.76]	2.4%	3.7%
Black LJ	438	652	0.67	[0.63; 0.71]	2.1%	3.7%
Daly RM	868	1393	0.62	[0.60; 0.65]	4.5%	3.7%
Daly RM	1823	2556	0.71	[0.70; 0.73]	8.3%	3.8%
Gill TK	346	510	0.68	[0.64; 0.72]	1.7%	3.7%
Gill TK	311	500	0.62	[0.58; 0.66]	1.6%	3.7%
Horton−French K	257	400	0.64	[0.59; 0.69]	1.3%	3.7%
Mahmood S	79	80	0.99	[0.93; 1.00]	0.3%	3.5%
Sokolovic S	263	450	0.58	[0.54; 0.63]	1.5%	3.7%
Tangoh DA	15	48	0.31	[0.19; 0.46]	0.2%	3.4%
Aucoin M	405	461	0.88	[0.85; 0.91]	1.5%	3.7%
Madsen KH	99	198	0.50	[0.43; 0.57]	0.6%	3.7%
Rabenberg M	2064	2456	0.84	[0.83; 0.85]	7.9%	3.8%
Leung RY	453	464	0.98	[0.96; 0.99]	1.5%	3.7%
Leung RY	755	791	0.95	[0.94; 0.97]	2.6%	3.7%
Ramakrishnan S	117	237	0.49	[0.43; 0.56]	0.8%	3.7%
Shivane VK	1055	1137	0.93	[0.91; 0.94]	3.7%	3.7%
Abdulrahman MA	161	278	0.58	[0.52; 0.64]	0.9%	3.7%
Contreras−Manzano A	287	381	0.75	[0.71; 0.80]	1.2%	3.7%
Contreras−Manzano A	367	477	0.77	[0.73; 0.81]	1.5%	3.7%
Ganmaa D	419	420	1.00	[0.99; 1.00]	1.4%	3.7%
Nadeem S	213	221	0.96	[0.93; 0.98]	0.7%	3.7%
Chailurkit LO	1194	2641	0.45	[0.43; 0.47]	8.5%	3.8%
Saeed BQ	244	287	0.85	[0.80; 0.89]	0.9%	3.7%
NHANES(2015...2016)	1730	2413	0.72	[0.70; 0.73]	7.8%	3.8%
Liu X	7985	10343	0.77	[0.76; 0.78]	33.4%	3.8%
**Common effect model**		**30923**	**0.75**	**[0.74; 0.75]**	**100.0%**	**−−**
**Random effects model**			**0.76**	**[0.68; 0.83]**	**−−**	**100.0%**

Heterogeneity: $I^2 = 99\%$, $\tau^2 = 0.0522$, $p = 0$

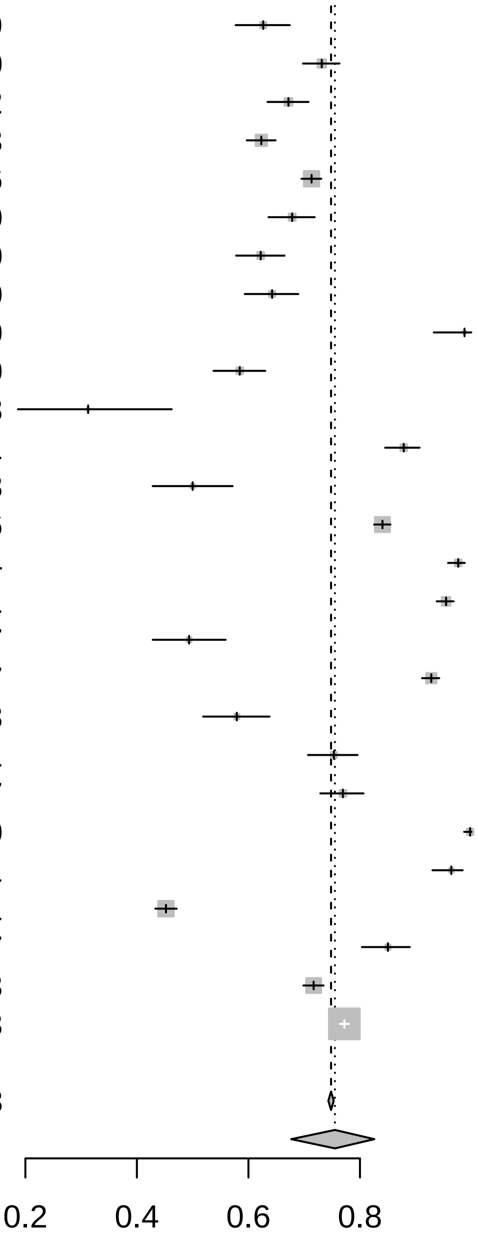

Supplementary figure 26 The prevalence of serum 25(OH)D < 75 nmol/L among people aged 19-44

Appendix 9: The prevalence of vitamin D deficiency by age

Study	Events	Total	Proportion	95%−CI	Weight (common)	Weight (random)
Daly RM	1598	2011	0.79	[0.78; 0.81]	10.1%	7.7%
Daly RM	2202	2884	0.76	[0.75; 0.78]	14.5%	7.7%
Gill TK	245	390	0.63	[0.58; 0.68]	2.0%	7.6%
Gill TK	333	486	0.69	[0.64; 0.73]	2.4%	7.7%
Madsen KH	112	227	0.49	[0.43; 0.56]	1.1%	7.6%
Rabenberg M	2465	2723	0.91	[0.89; 0.92]	13.7%	7.7%
Rabenberg M	2465	2723	0.91	[0.89; 0.92]	13.7%	7.7%
Leung RY	164	1211	0.14	[0.12; 0.16]	6.1%	7.7%
Sokolovic S	513	645	0.80	[0.76; 0.83]	3.2%	7.7%
Black LJ	348	490	0.71	[0.67; 0.75]	2.5%	7.7%
Black LJ	455	648	0.70	[0.67; 0.74]	3.3%	7.7%
Ikonen H	2597	3650	0.71	[0.70; 0.73]	18.4%	7.7%
NHANES(2015...2016)	992	1777	0.56	[0.53; 0.58]	8.9%	7.7%
**Common effect model**		**19865**	**0.74**	**[0.74; 0.75]**	**100.0%**	**−−**
**Random effects model**			**0.69**	**[0.57; 0.79]**	**−−**	**100.0%**

Heterogeneity: $I^2 = 100\%$, $\tau^2 = 0.0513$, $p = 0$

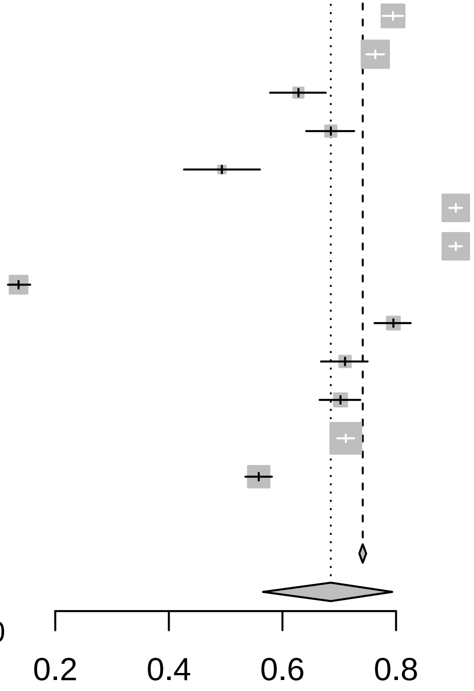

Supplementary figure 27 The prevalence of serum 25(OH)D < 75 nmol/L among people aged 45-64

Appendix 9: The prevalence of vitamin D deficiency by age

Study	Events	Total	Proportion	95%−CI	Weight (common)	Weight (random)
Oliveri B	307	339	0.91	[0.87; 0.93]	1.4%	3.4%
Black LJ	245	331	0.74	[0.69; 0.79]	1.4%	3.4%
Daly RM	674	810	0.83	[0.80; 0.86]	3.4%	3.5%
Daly RM	1235	1564	0.79	[0.77; 0.81]	6.6%	3.5%
Gill TK	165	263	0.63	[0.57; 0.69]	1.1%	3.4%
Gill TK	150	265	0.57	[0.50; 0.63]	1.1%	3.4%
Hirani V	133	174	0.76	[0.69; 0.83]	0.7%	3.4%
Hirani V	248	304	0.82	[0.77; 0.86]	1.3%	3.4%
Hirani V	421	522	0.81	[0.77; 0.84]	2.2%	3.5%
Hirani V	554	659	0.84	[0.81; 0.87]	2.8%	3.5%
Robinson PJ	238	267	0.89	[0.85; 0.93]	1.1%	3.4%
Robinson PJ	562	640	0.88	[0.85; 0.90]	2.7%	3.5%
Matheï C	323	367	0.88	[0.84; 0.91]	1.6%	3.4%
Lopes JB	363	415	0.87	[0.84; 0.90]	1.8%	3.4%
Greene−Finestone LS	185	312	0.59	[0.54; 0.65]	1.3%	3.4%
Yousef S	799	1361	0.59	[0.56; 0.61]	5.8%	3.5%
Orces CH	277	403	0.69	[0.64; 0.73]	1.7%	3.4%
Orces CH	510	724	0.70	[0.67; 0.74]	3.1%	3.5%
Klenk J	291	1418	0.21	[0.18; 0.23]	6.0%	3.5%
Rabenberg M	1687	1816	0.93	[0.92; 0.94]	7.7%	3.5%
Leung RY	808	899	0.90	[0.88; 0.92]	3.8%	3.5%
Wyskida M	3002	3472	0.86	[0.85; 0.88]	14.7%	3.5%
Miljkovic I	114	424	0.27	[0.23; 0.31]	1.8%	3.4%
Hribar M	131	155	0.85	[0.78; 0.90]	0.7%	3.4%
Samefors M	318	333	0.95	[0.93; 0.97]	1.4%	3.4%
Ten Haaf DSM	108	450	0.24	[0.20; 0.28]	1.9%	3.4%
Hirani V	1788	2070	0.86	[0.85; 0.88]	8.8%	3.5%
NHANES(2015...2016)	474	1231	0.39	[0.36; 0.41]	5.2%	3.5%
Orwoll E	1142	1606	0.71	[0.69; 0.73]	6.8%	3.5%
**Common effect model**		**23594**	**0.75**	**[0.74; 0.75]**	**100.0%**	**−−**
**Random effects model**			**0.74**	**[0.66; 0.81]**	**−−**	**100.0%**

Heterogeneity: $I^2 = 99\%$, $\tau^2 = 0.0562$, $p = 0$

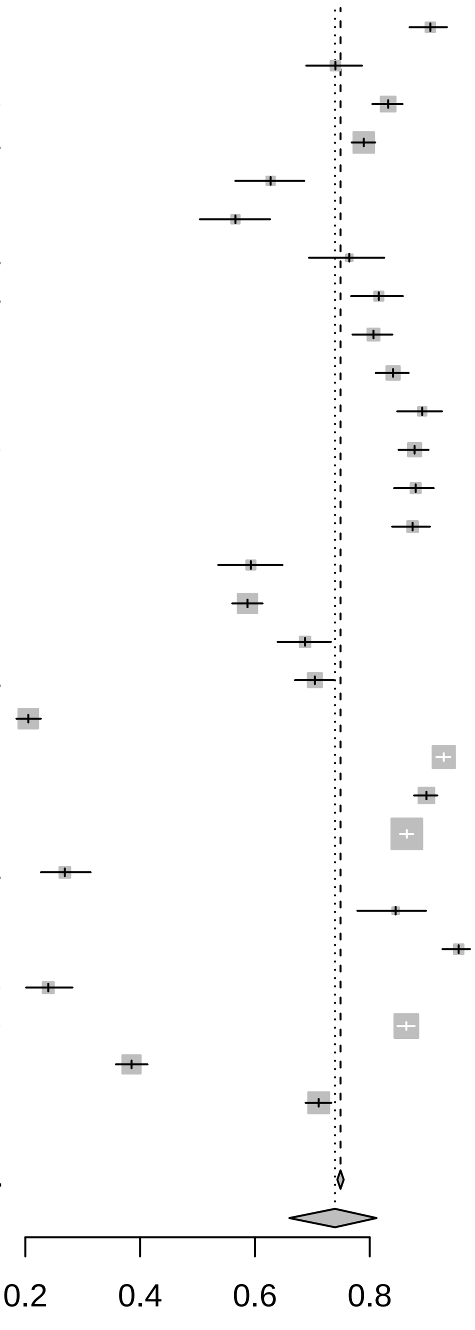

Supplementary figure 28 The prevalence of serum 25(OH)D < 75 nmol/L among people aged > 65

Appendix 10: The prevalence of vitamin D deficiency by gender

Study	Events	Total	Proportion	95%−CI	Weight (common)	Weight (random)
Tran B	29	342	0.08	[0.06; 0.12]	0.0%	1.2%
Daly RM	14	590	0.02	[0.01; 0.04]	0.0%	1.2%
Daly RM	20	1092	0.02	[0.01; 0.03]	0.0%	1.2%
Daly RM	31	1343	0.02	[0.02; 0.03]	0.0%	1.2%
Daly RM	49	923	0.05	[0.04; 0.07]	0.0%	1.2%
Daly RM	13	730	0.02	[0.01; 0.03]	0.0%	1.2%
Daly RM	10	362	0.03	[0.01; 0.05]	0.0%	1.2%
Cairncross CT	35	681	0.05	[0.04; 0.07]	0.0%	1.2%
Gill TK	5	1164	0.00	[0.00; 0.01]	0.0%	1.2%
Black LJ	10	158	0.06	[0.03; 0.11]	0.0%	1.1%
Black LJ	16	216	0.07	[0.04; 0.12]	0.0%	1.1%
Black LJ	7	297	0.02	[0.01; 0.05]	0.0%	1.2%
Black LJ	10	262	0.04	[0.02; 0.07]	0.0%	1.2%
Black LJ	8	227	0.04	[0.02; 0.07]	0.0%	1.1%
Black LJ	7	140	0.05	[0.02; 0.10]	0.0%	1.1%
Mansbach JM	9	904	0.01	[0.00; 0.02]	0.0%	1.2%
Mansbach JM	13	1359	0.01	[0.01; 0.02]	0.0%	1.2%
Greene−Finestone LS	16	401	0.04	[0.02; 0.06]	0.0%	1.2%
Greene−Finestone LS	10	384	0.03	[0.01; 0.05]	0.0%	1.2%
Greene−Finestone LS	3	130	0.02	[0.00; 0.07]	0.0%	1.1%
Sarafin K	532	5848	0.09	[0.08; 0.10]	0.2%	1.2%
Yousef S	643	5774	0.11	[0.10; 0.12]	0.2%	1.2%
Herrick KA	353	8013	0.04	[0.04; 0.05]	0.2%	1.2%
Andersen R	61	95	0.64	[0.54; 0.74]	0.0%	1.1%
Bjarnadottir A	2	73	0.03	[0.00; 0.10]	0.0%	1.1%
Borissova AM	143	948	0.15	[0.13; 0.18]	0.0%	1.2%
Capuano R	57	600	0.10	[0.07; 0.12]	0.0%	1.2%
Crowe FL	269517	619579	0.44	[0.43; 0.44]	18.0%	1.2%
Crowe FL	271453	638713	0.42	[0.42; 0.43]	18.6%	1.2%
Crowe FL	242108	612932	0.39	[0.39; 0.40]	17.8%	1.2%
Crowe FL	163749	465198	0.35	[0.35; 0.35]	13.5%	1.2%
Crowe FL	118838	378467	0.31	[0.31; 0.32]	11.0%	1.2%
Crowe FL	128300	426246	0.30	[0.30; 0.30]	12.4%	1.2%
Granlund L	12	111	0.11	[0.06; 0.18]	0.0%	1.1%
Hansen L	53	264	0.20	[0.15; 0.25]	0.0%	1.2%
Hansen L	157	1048	0.15	[0.13; 0.17]	0.0%	1.2%
Hintzpeter B	920	4582	0.20	[0.19; 0.21]	0.1%	1.2%
Hribar M	12	52	0.23	[0.13; 0.37]	0.0%	1.1%
Hribar M	15	76	0.20	[0.11; 0.30]	0.0%	1.1%
Karin Z	13	132	0.10	[0.05; 0.16]	0.0%	1.1%
Laird E	327	1364	0.24	[0.22; 0.26]	0.0%	1.2%
Lin LY	28507	208338	0.14	[0.14; 0.14]	6.1%	1.2%
Madsen KH	2	96	0.02	[0.00; 0.07]	0.0%	1.1%
Madsen KH	1	111	0.01	[0.00; 0.05]	0.0%	1.1%
Man PW	6	103	0.06	[0.02; 0.12]	0.0%	1.1%
Manios Y	38	1179	0.03	[0.02; 0.04]	0.0%	1.2%
Nälsén C	2	124	0.02	[0.00; 0.06]	0.0%	1.1%
Nälsén C	3	114	0.03	[0.01; 0.07]	0.0%	1.1%
Nielsen NO	232	1256	0.18	[0.16; 0.21]	0.0%	1.2%
Patel JV	678	988	0.69	[0.66; 0.72]	0.0%	1.2%
Pérez−Llamas F	6	29	0.21	[0.08; 0.40]	0.0%	1.0%
Petrenya N	12	2041	0.01	[0.00; 0.01]	0.1%	1.2%
Petrenya N	37	2041	0.02	[0.01; 0.02]	0.1%	1.2%
Rabenberg M	387	1179	0.33	[0.30; 0.36]	0.0%	1.2%
Rabenberg M	386	1271	0.30	[0.28; 0.33]	0.0%	1.2%
Rabenberg M	242	910	0.27	[0.24; 0.30]	0.0%	1.2%
Raposo L	81	214	0.38	[0.31; 0.45]	0.0%	1.1%
Santos A	194	628	0.31	[0.27; 0.35]	0.0%	1.2%
Wyskida M	211	1814	0.12	[0.10; 0.13]	0.1%	1.2%
Zhu Z	17	1454	0.01	[0.01; 0.02]	0.0%	1.2%
Zhu Z	16	1019	0.02	[0.01; 0.03]	0.0%	1.2%
Zhu Z	3	135	0.02	[0.00; 0.06]	0.0%	1.1%
Choi HR	43	269	0.16	[0.12; 0.21]	0.0%	1.2%
Sharawat IK	17	55	0.31	[0.19; 0.45]	0.0%	1.1%
Bachhel R	21	75	0.28	[0.18; 0.40]	0.0%	1.1%
Hovsepian S	73	243	0.30	[0.24; 0.36]	0.0%	1.1%
Nikooyeh B	424	655	0.65	[0.61; 0.68]	0.0%	1.2%
Nikooyeh B	133	322	0.41	[0.36; 0.47]	0.0%	1.2%
Saki F	25	241	0.10	[0.07; 0.15]	0.0%	1.1%
Saliba W	171	2790	0.06	[0.05; 0.07]	0.1%	1.2%
Bater J	2792	4828	0.58	[0.56; 0.59]	0.1%	1.2%
Jayatissa R	79	1178	0.07	[0.05; 0.08]	0.0%	1.2%
Gromova O	88	528	0.17	[0.14; 0.20]	0.0%	1.2%
AlQuaiz AM	217	962	0.23	[0.20; 0.25]	0.0%	1.2%
Al−Daghri NM	230	1187	0.19	[0.17; 0.22]	0.0%	1.2%
Al−Daghri NM	65	368	0.18	[0.14; 0.22]	0.0%	1.2%
Al−Daghri NM	620	2999	0.21	[0.19; 0.22]	0.1%	1.2%
Abiaka C	24	101	0.24	[0.16; 0.33]	0.0%	1.1%
Karagüzel G	21	189	0.11	[0.07; 0.16]	0.0%	1.1%
Chen J	364	2948	0.12	[0.11; 0.14]	0.1%	1.2%
Yan X	39	176	0.22	[0.16; 0.29]	0.0%	1.1%
Fang F	38	762	0.05	[0.04; 0.07]	0.0%	1.2%
Li H	1258	5517	0.23	[0.22; 0.24]	0.2%	1.2%
Asakura K	15	53	0.28	[0.17; 0.42]	0.0%	1.1%
Al Hayek S	18	172	0.10	[0.06; 0.16]	0.0%	1.1%
Arabi A	30	171	0.18	[0.12; 0.24]	0.0%	1.1%
Zhang FF	114	436	0.26	[0.22; 0.31]	0.0%	1.2%
**Common effect model**		**3434089**	**0.36**	**[0.35; 0.36]**	**100.0%**	**−−**
**Random effects model**			**0.14**	**[0.11; 0.17]**	**−−**	**100.0%**

Heterogeneity: $I^2 = 100\%$, $\tau^2 = 0.0452$, $p = 0$

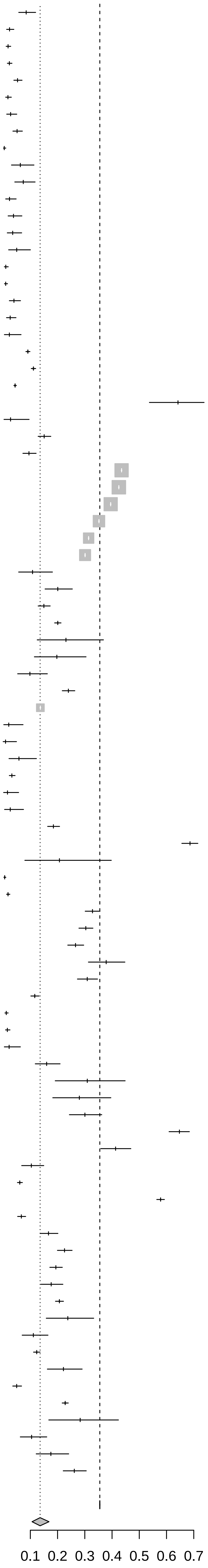

Supplementary figure 29 The prevalence of serum 25(OH)D < 30 nmol/L among males

Appendix 10: The prevalence of vitamin D deficiency by gender

Study	Events	Total	Proportion	95%−CI	Weight (common)	Weight (random)
Tran B	37	301	0.12	[0.09; 0.17]	0.0%	1.2%
Daly RM	31	803	0.04	[0.03; 0.05]	0.0%	1.2%
Daly RM	88	1464	0.06	[0.05; 0.07]	0.0%	1.2%
Daly RM	80	1541	0.05	[0.04; 0.06]	0.0%	1.2%
Daly RM	28	1088	0.03	[0.02; 0.04]	0.0%	1.2%
Daly RM	62	834	0.07	[0.06; 0.09]	0.0%	1.2%
Daly RM	56	448	0.12	[0.10; 0.16]	0.0%	1.2%
Cairncross CT	41	648	0.06	[0.05; 0.08]	0.0%	1.2%
Gill TK	16	1249	0.01	[0.01; 0.02]	0.0%	1.2%
Black LJ	4	241	0.02	[0.00; 0.04]	0.0%	1.1%
Black LJ	25	436	0.06	[0.04; 0.08]	0.0%	1.2%
Black LJ	21	433	0.05	[0.03; 0.07]	0.0%	1.2%
Black LJ	21	386	0.05	[0.03; 0.08]	0.0%	1.2%
Black LJ	15	263	0.06	[0.03; 0.09]	0.0%	1.1%
Black LJ	8	191	0.04	[0.02; 0.08]	0.0%	1.1%
Mansbach JM	8	895	0.01	[0.00; 0.02]	0.0%	1.2%
Mansbach JM	28	1400	0.02	[0.01; 0.03]	0.0%	1.2%
Greene−Finestone LS	4	422	0.01	[0.00; 0.02]	0.0%	1.2%
Greene−Finestone LS	8	393	0.02	[0.01; 0.04]	0.0%	1.2%
Greene−Finestone LS	5	182	0.03	[0.01; 0.06]	0.0%	1.1%
Sarafin K	334	5852	0.06	[0.05; 0.06]	0.2%	1.2%
Yousef S	549	5805	0.09	[0.09; 0.10]	0.2%	1.2%
Herrick KA	466	8167	0.06	[0.05; 0.06]	0.2%	1.2%
Andersen R	97	115	0.84	[0.76; 0.90]	0.0%	1.1%
Bjarnadottir A	3	85	0.04	[0.01; 0.10]	0.0%	1.1%
Borissova AM	287	1068	0.27	[0.24; 0.30]	0.0%	1.2%
Capuano R	80	600	0.13	[0.11; 0.16]	0.0%	1.2%
Crowe FL	266596	650233	0.41	[0.41; 0.41]	18.0%	1.2%
Crowe FL	260228	677677	0.38	[0.38; 0.39]	18.8%	1.2%
Crowe FL	201340	567154	0.36	[0.35; 0.36]	15.7%	1.2%
Crowe FL	136516	436154	0.31	[0.31; 0.31]	12.1%	1.2%
Crowe FL	104348	376707	0.28	[0.28; 0.28]	10.4%	1.2%
Crowe FL	171430	567649	0.30	[0.30; 0.30]	15.7%	1.2%
Granlund L	15	105	0.14	[0.08; 0.22]	0.0%	1.1%
Hansen L	27	263	0.10	[0.07; 0.15]	0.0%	1.1%
Hansen L	152	1517	0.10	[0.09; 0.12]	0.0%	1.2%
Hintzpeter B	877	4419	0.20	[0.19; 0.21]	0.1%	1.2%
Hribar M	20	73	0.27	[0.18; 0.39]	0.0%	1.1%
Hribar M	22	79	0.28	[0.18; 0.39]	0.0%	1.1%
Karin Z	19	128	0.15	[0.09; 0.22]	0.0%	1.1%
Laird E	315	1553	0.20	[0.18; 0.22]	0.0%	1.2%
Lin LY	32180	240263	0.13	[0.13; 0.14]	6.7%	1.2%
Madsen KH	0	102	0.00	[0.00; 0.04]	0.0%	1.1%
Madsen KH	1	106	0.01	[0.00; 0.05]	0.0%	1.1%
Man PW	34	313	0.11	[0.08; 0.15]	0.0%	1.2%
Manios Y	84	1174	0.07	[0.06; 0.09]	0.0%	1.2%
Nälsén C	7	144	0.05	[0.02; 0.10]	0.0%	1.1%
Nälsén C	7	92	0.08	[0.03; 0.15]	0.0%	1.1%
Nielsen NO	293	1621	0.18	[0.16; 0.20]	0.0%	1.2%
Patel JV	577	916	0.63	[0.60; 0.66]	0.0%	1.2%
Pérez−Llamas F	23	57	0.40	[0.28; 0.54]	0.0%	1.1%
Petrenya N	17	2424	0.01	[0.00; 0.01]	0.1%	1.2%
Petrenya N	46	2424	0.02	[0.01; 0.03]	0.1%	1.2%
Rabenberg M	369	1277	0.29	[0.26; 0.31]	0.0%	1.2%
Rabenberg M	418	1452	0.29	[0.26; 0.31]	0.0%	1.2%
Rabenberg M	298	906	0.33	[0.30; 0.36]	0.0%	1.2%
Raposo L	107	286	0.37	[0.32; 0.43]	0.0%	1.1%
Santos A	400	872	0.46	[0.43; 0.49]	0.0%	1.2%
Wyskida M	144	1658	0.09	[0.07; 0.10]	0.0%	1.2%
Zhu Z	9	815	0.01	[0.01; 0.02]	0.0%	1.2%
Zhu Z	13	421	0.03	[0.02; 0.05]	0.0%	1.2%
Zhu Z	2	43	0.05	[0.01; 0.16]	0.0%	1.1%
Choi HR	67	382	0.18	[0.14; 0.22]	0.0%	1.2%
Sharawat IK	17	45	0.38	[0.24; 0.53]	0.0%	1.1%
Bachhel R	39	75	0.52	[0.40; 0.64]	0.0%	1.1%
Hovsepian S	139	868	0.16	[0.14; 0.19]	0.0%	1.2%
Nikooyeh B	549	751	0.73	[0.70; 0.76]	0.0%	1.2%
Nikooyeh B	241	345	0.70	[0.65; 0.75]	0.0%	1.2%
Saki F	38	263	0.14	[0.10; 0.19]	0.0%	1.1%
Saliba W	720	3128	0.23	[0.22; 0.25]	0.1%	1.2%
Bater J	2900	4764	0.61	[0.59; 0.62]	0.1%	1.2%
Jayatissa R	255	1437	0.18	[0.16; 0.20]	0.0%	1.2%
Gromova O	283	819	0.35	[0.31; 0.38]	0.0%	1.2%
AlQuaiz AM	512	1870	0.27	[0.25; 0.29]	0.1%	1.2%
Al−Daghri NM	488	1038	0.47	[0.44; 0.50]	0.0%	1.2%
Al−Daghri NM	170	462	0.37	[0.32; 0.41]	0.0%	1.2%
Al−Daghri NM	1545	4361	0.35	[0.34; 0.37]	0.1%	1.2%
Abiaka C	55	105	0.52	[0.42; 0.62]	0.0%	1.1%
Karagüzel G	54	182	0.30	[0.23; 0.37]	0.0%	1.1%
Chen J	367	3066	0.12	[0.11; 0.13]	0.1%	1.2%
Yan X	42	126	0.33	[0.25; 0.42]	0.0%	1.1%
Fang F	101	1052	0.10	[0.08; 0.12]	0.0%	1.2%
Li H	1605	5179	0.31	[0.30; 0.32]	0.1%	1.2%
Asakura K	25	54	0.46	[0.33; 0.60]	0.0%	1.1%
Al Hayek S	15	172	0.09	[0.05; 0.14]	0.0%	1.1%
Arabi A	152	295	0.52	[0.46; 0.57]	0.0%	1.2%
Zhang FF	234	524	0.45	[0.40; 0.49]	0.0%	1.2%
**Common effect model**		**3607750**	**0.33**	**[0.32; 0.33]**	**100.0%**	**−−**
**Random effects model**			**0.18**	**[0.14; 0.22]**	**−−**	**100.0%**

Heterogeneity: $I^2 = 100\%$, $\tau^2 = 0.0602$, $p = 0$

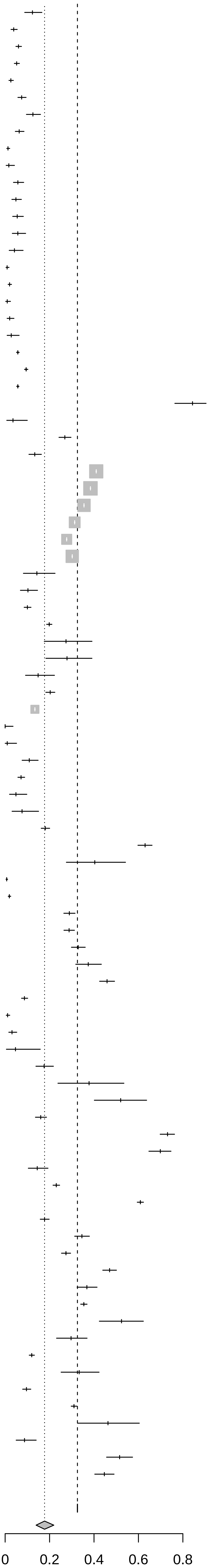

Supplementary figure 30 The prevalence of serum 25(OH)D < 30 nmol/L among females

Appendix 10: The prevalence of vitamin D deficiency by gender

Study	Events	Total	Proportion	95%−CI	Weight (common)	Weight (random)
Black LJ	45	158	0.28	[0.22; 0.36]	0.0%	0.8%
Black LJ	62	216	0.29	[0.23; 0.35]	0.1%	0.9%
Black LJ	65	297	0.22	[0.17; 0.27]	0.1%	0.9%
Black LJ	59	262	0.23	[0.18; 0.28]	0.1%	0.9%
Black LJ	71	227	0.31	[0.25; 0.38]	0.1%	0.9%
Black LJ	36	140	0.26	[0.19; 0.34]	0.0%	0.8%
Tran B	247	342	0.72	[0.67; 0.77]	0.1%	0.9%
Daly RM	98	590	0.17	[0.14; 0.20]	0.2%	0.9%
Daly RM	235	1092	0.22	[0.19; 0.24]	0.3%	0.9%
Daly RM	313	1343	0.23	[0.21; 0.26]	0.4%	0.9%
Daly RM	267	923	0.29	[0.26; 0.32]	0.3%	0.9%
Daly RM	161	730	0.22	[0.19; 0.25]	0.2%	0.9%
Daly RM	100	362	0.28	[0.23; 0.33]	0.1%	0.9%
Cairncross CT	295	681	0.43	[0.40; 0.47]	0.2%	0.9%
Gill TK	215	1164	0.18	[0.16; 0.21]	0.3%	0.9%
Djennane M	47	203	0.23	[0.18; 0.30]	0.1%	0.9%
Carrillo−Vega MF	145	550	0.26	[0.23; 0.30]	0.2%	0.9%
Flores ME	263	1741	0.15	[0.13; 0.17]	0.5%	0.9%
Greene−Finestone LS	62	401	0.15	[0.12; 0.19]	0.1%	0.9%
Greene−Finestone LS	96	384	0.25	[0.21; 0.30]	0.1%	0.9%
Greene−Finestone LS	51	130	0.39	[0.31; 0.48]	0.0%	0.8%
Sarafin K	2386	5848	0.41	[0.40; 0.42]	1.6%	0.9%
Yousef S	2289	5774	0.40	[0.38; 0.41]	1.6%	0.9%
de Oliveira CL	72	449	0.16	[0.13; 0.20]	0.1%	0.9%
Orces CH	123	1072	0.11	[0.10; 0.14]	0.3%	0.9%
Liu X	4460	12610	0.35	[0.35; 0.36]	3.5%	0.9%
Herrick KA	1851	8013	0.23	[0.22; 0.24]	2.2%	0.9%
Mansbach JM	135	904	0.15	[0.13; 0.17]	0.3%	0.9%
Mansbach JM	257	1359	0.19	[0.17; 0.21]	0.4%	0.9%
Andersen R	90	95	0.95	[0.88; 0.98]	0.0%	0.8%
Andersen S	30	48	0.62	[0.47; 0.76]	0.0%	0.8%
Bettencourt A	45	103	0.44	[0.34; 0.54]	0.0%	0.8%
Bjarnadottir A	45	73	0.62	[0.50; 0.73]	0.0%	0.8%
Borissova AM	667	948	0.70	[0.67; 0.73]	0.3%	0.9%
Capuano R	330	600	0.55	[0.51; 0.59]	0.2%	0.9%
Feketea GM	46	184	0.25	[0.19; 0.32]	0.1%	0.9%
Hansen L	629	1048	0.60	[0.57; 0.63]	0.3%	0.9%
Hansen L	164	264	0.62	[0.56; 0.68]	0.1%	0.9%
Hintzpeter B	2970	4582	0.65	[0.63; 0.66]	1.3%	0.9%
Hribar M	26	52	0.50	[0.36; 0.64]	0.0%	0.8%
Hribar M	46	76	0.61	[0.49; 0.72]	0.0%	0.8%
Karin Z	65	132	0.49	[0.40; 0.58]	0.0%	0.8%
Kull M Jr	133	167	0.80	[0.73; 0.85]	0.0%	0.9%
Laird E	837	1364	0.61	[0.59; 0.64]	0.4%	0.9%
Lin LY	115875	208338	0.56	[0.55; 0.56]	57.7%	0.9%
Madsen KH	4	77	0.05	[0.01; 0.13]	0.0%	0.8%
Madsen KH	8	96	0.08	[0.04; 0.16]	0.0%	0.8%
Madsen KH	4	85	0.05	[0.01; 0.12]	0.0%	0.8%
Madsen KH	11	111	0.10	[0.05; 0.17]	0.0%	0.8%
Man PW	70	103	0.68	[0.58; 0.77]	0.0%	0.8%
Manios Y	566	1179	0.48	[0.45; 0.51]	0.3%	0.9%
Nälsén C	47	114	0.41	[0.32; 0.51]	0.0%	0.8%
Nälsén C	30	124	0.24	[0.17; 0.33]	0.0%	0.8%
Nielsen NO	718	1256	0.57	[0.54; 0.60]	0.3%	0.9%
Patel JV	902	988	0.91	[0.89; 0.93]	0.3%	0.9%
Pérez−Llamas F	16	29	0.55	[0.36; 0.74]	0.0%	0.8%
Petrenya N	500	2041	0.24	[0.23; 0.26]	0.6%	0.9%
Rabenberg M	725	1179	0.61	[0.59; 0.64]	0.3%	0.9%
Rabenberg M	781	1271	0.61	[0.59; 0.64]	0.4%	0.9%
Rabenberg M	569	910	0.63	[0.59; 0.66]	0.3%	0.9%
Raposo L	181	214	0.85	[0.79; 0.89]	0.1%	0.9%
Santos A	300	628	0.48	[0.44; 0.52]	0.2%	0.9%
Vierucci F	111	213	0.52	[0.45; 0.59]	0.1%	0.9%
Abiaka C	82	101	0.81	[0.72; 0.88]	0.0%	0.8%
Al Hayek S	60	172	0.35	[0.28; 0.43]	0.0%	0.9%
Al Zarooni AAR	3790	4561	0.83	[0.82; 0.84]	1.3%	0.9%
Al−Daghri NM	941	1187	0.79	[0.77; 0.82]	0.3%	0.9%
Al−Daghri NM	273	368	0.74	[0.69; 0.79]	0.1%	0.9%
Al−Daghri NM	2183	2999	0.73	[0.71; 0.74]	0.8%	0.9%
AlQuaiz AM	695	962	0.72	[0.69; 0.75]	0.3%	0.9%
Arabi A	115	171	0.67	[0.60; 0.74]	0.0%	0.9%
Asakura K	41	53	0.77	[0.64; 0.88]	0.0%	0.8%
Batieha A	17	1128	0.02	[0.01; 0.02]	0.3%	0.9%
Bener A	153	238	0.64	[0.58; 0.70]	0.1%	0.9%
Chailurkit LO	25	1321	0.02	[0.01; 0.03]	0.4%	0.9%
Chen J	1141	2948	0.39	[0.37; 0.40]	0.8%	0.9%
Chung JY	4295	7957	0.54	[0.53; 0.55]	2.2%	0.9%
Cinar N	54	65	0.83	[0.72; 0.91]	0.0%	0.8%
El−Khateeb M	639	1183	0.54	[0.51; 0.57]	0.3%	0.9%
Fang F	304	762	0.40	[0.36; 0.43]	0.2%	0.9%
Feng X	134	310	0.43	[0.38; 0.49]	0.1%	0.9%
Gariballa S	63	157	0.40	[0.32; 0.48]	0.0%	0.8%
Gökta O	1110	2592	0.43	[0.41; 0.45]	0.7%	0.9%
Gromova O	296	528	0.56	[0.52; 0.60]	0.1%	0.9%
Ho−Pham LT	2	205	0.01	[0.00; 0.03]	0.1%	0.9%
Ho−Pham LT	2	205	0.01	[0.00; 0.03]	0.1%	0.9%
Hovsepian S	127	243	0.52	[0.46; 0.59]	0.1%	0.9%
Jayashri R	320	668	0.48	[0.44; 0.52]	0.2%	0.9%
Jayatissa R	553	1178	0.47	[0.44; 0.50]	0.3%	0.9%
Kapil U	144	187	0.77	[0.70; 0.83]	0.1%	0.9%
Kapil U	281	420	0.67	[0.62; 0.71]	0.1%	0.9%
Karagüzel G	119	189	0.63	[0.56; 0.70]	0.1%	0.9%
Kaykhaei MA	346	431	0.80	[0.76; 0.84]	0.1%	0.9%
Koyama S	55	247	0.22	[0.17; 0.28]	0.1%	0.9%
Lee J	25446	36759	0.69	[0.69; 0.70]	10.2%	0.9%
Leung RY	730	1583	0.46	[0.44; 0.49]	0.4%	0.9%
Liu X	25	96	0.26	[0.18; 0.36]	0.0%	0.8%
Mallah EM	76	99	0.77	[0.67; 0.85]	0.0%	0.8%
Mehboobali N	115	351	0.33	[0.28; 0.38]	0.1%	0.9%
Metwally ASM	462	549	0.84	[0.81; 0.87]	0.2%	0.9%
Meyer HE	29	85	0.34	[0.24; 0.45]	0.0%	0.8%
Moussavi M	28	153	0.18	[0.13; 0.25]	0.0%	0.8%
Naeem Z	32	83	0.39	[0.28; 0.50]	0.0%	0.8%
Nikooyeh B	299	322	0.93	[0.89; 0.95]	0.1%	0.9%
Saki F	189	241	0.78	[0.73; 0.83]	0.1%	0.9%
Saliba W	1047	2790	0.38	[0.36; 0.39]	0.8%	0.9%
Sharawat IK	35	55	0.64	[0.50; 0.76]	0.0%	0.8%
Sherchand O	45	109	0.41	[0.32; 0.51]	0.0%	0.8%
Suryanarayana P	107	187	0.57	[0.50; 0.64]	0.1%	0.9%
Yan X	121	176	0.69	[0.61; 0.76]	0.0%	0.9%
Zargar AH	49	64	0.77	[0.64; 0.86]	0.0%	0.8%
Zhang FF	352	436	0.81	[0.77; 0.84]	0.1%	0.9%
Zhao Y	526	1090	0.48	[0.45; 0.51]	0.3%	0.9%
Zhen D	1857	2902	0.64	[0.62; 0.66]	0.8%	0.9%
Zhu Z	302	1454	0.21	[0.19; 0.23]	0.4%	0.9%
Zhu Z	398	1019	0.39	[0.36; 0.42]	0.3%	0.9%
Zhu Z	60	135	0.44	[0.36; 0.53]	0.0%	0.8%
**Common effect model**		**361131**	**0.53**	**[0.53; 0.53]**	**100.0%**	**−−**
**Random effects model**			**0.45**	**[0.40; 0.50]**	**−−**	**100.0%**

Heterogeneity: $I^2 = 100\%$, $\tau^2 = 0.0718$, $p = 0$

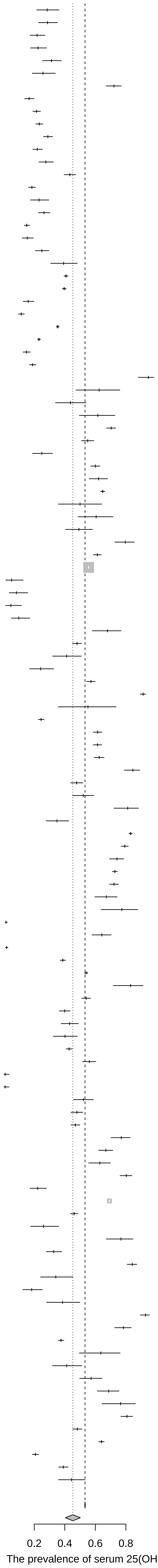

Supplementary figure 31 The prevalence of serum 25(OH)D < 50 nmol/L among males

Appendix 10: The prevalence of vitamin D deficiency by gender

Study	Events	Total	Proportion	95%−CI	Weight (common)	Weight (random)
Black LJ	54	241	0.22	[0.17; 0.28]	0.1%	0.9%
Black LJ	104	436	0.24	[0.20; 0.28]	0.1%	0.9%
Black LJ	127	433	0.29	[0.25; 0.34]	0.1%	0.9%
Black LJ	135	386	0.35	[0.30; 0.40]	0.1%	0.9%
Black LJ	76	263	0.29	[0.23; 0.35]	0.1%	0.9%
Black LJ	60	191	0.31	[0.25; 0.39]	0.0%	0.9%
Tran B	236	301	0.78	[0.73; 0.83]	0.1%	0.9%
Daly RM	210	803	0.26	[0.23; 0.29]	0.2%	0.9%
Daly RM	527	1464	0.36	[0.34; 0.39]	0.3%	0.9%
Daly RM	670	1541	0.43	[0.41; 0.46]	0.4%	0.9%
Daly RM	475	1088	0.44	[0.41; 0.47]	0.3%	0.9%
Daly RM	384	834	0.46	[0.43; 0.49]	0.2%	0.9%
Daly RM	254	448	0.57	[0.52; 0.61]	0.1%	0.9%
Cairncross CT	337	648	0.52	[0.48; 0.56]	0.2%	0.9%
Gill TK	334	1249	0.27	[0.24; 0.29]	0.3%	0.9%
Djennane M	83	232	0.36	[0.30; 0.42]	0.1%	0.9%
Carrillo−Vega MF	271	578	0.47	[0.43; 0.51]	0.1%	0.9%
Flores ME	336	1741	0.19	[0.17; 0.21]	0.4%	0.9%
Greene−Finestone LS	94	422	0.22	[0.18; 0.27]	0.1%	0.9%
Greene−Finestone LS	78	393	0.20	[0.16; 0.24]	0.1%	0.9%
Greene−Finestone LS	81	182	0.45	[0.37; 0.52]	0.0%	0.9%
Sarafin K	1920	5852	0.33	[0.32; 0.34]	1.4%	0.9%
Yousef S	1918	5805	0.33	[0.32; 0.34]	1.4%	0.9%
de Oliveira CL	183	703	0.26	[0.23; 0.29]	0.2%	0.9%
Orces CH	385	1302	0.30	[0.27; 0.32]	0.3%	0.9%
Liu X	5304	13400	0.40	[0.39; 0.40]	3.2%	0.9%
Herrick KA	1920	8167	0.24	[0.23; 0.24]	1.9%	0.9%
Mansbach JM	133	895	0.15	[0.13; 0.17]	0.2%	0.9%
Mansbach JM	336	1400	0.24	[0.22; 0.26]	0.3%	0.9%
Andersen R	111	115	0.97	[0.91; 0.99]	0.0%	0.8%
Andersen S	38	49	0.78	[0.63; 0.88]	0.0%	0.8%
Bettencourt A	50	95	0.53	[0.42; 0.63]	0.0%	0.8%
Bjarnadottir A	58	85	0.68	[0.57; 0.78]	0.0%	0.8%
Borissova AM	861	1068	0.81	[0.78; 0.83]	0.3%	0.9%
Capuano R	356	600	0.59	[0.55; 0.63]	0.1%	0.9%
Feketea GM	56	192	0.29	[0.23; 0.36]	0.0%	0.9%
Hansen L	653	1517	0.43	[0.41; 0.46]	0.4%	0.9%
Hansen L	158	263	0.60	[0.54; 0.66]	0.1%	0.9%
Hintzpeter B	2925	4419	0.66	[0.65; 0.68]	1.0%	0.9%
Hribar M	48	73	0.66	[0.54; 0.76]	0.0%	0.8%
Hribar M	52	79	0.66	[0.54; 0.76]	0.0%	0.8%
Karin Z	86	128	0.67	[0.58; 0.75]	0.0%	0.8%
Kull M Jr	135	200	0.68	[0.61; 0.74]	0.0%	0.9%
Laird E	809	1553	0.52	[0.50; 0.55]	0.4%	0.9%
Lin LY	132603	240263	0.55	[0.55; 0.55]	57.0%	0.9%
Madsen KH	5	84	0.06	[0.02; 0.13]	0.0%	0.8%
Madsen KH	7	102	0.07	[0.03; 0.14]	0.0%	0.8%
Madsen KH	5	94	0.05	[0.02; 0.12]	0.0%	0.8%
Madsen KH	17	106	0.16	[0.10; 0.24]	0.0%	0.8%
Man PW	166	313	0.53	[0.47; 0.59]	0.1%	0.9%
Manios Y	669	1174	0.57	[0.54; 0.60]	0.3%	0.9%
Nälsén C	40	92	0.43	[0.33; 0.54]	0.0%	0.8%
Nälsén C	30	144	0.21	[0.15; 0.28]	0.0%	0.8%
Nielsen NO	908	1621	0.56	[0.54; 0.58]	0.4%	0.9%
Patel JV	785	916	0.86	[0.83; 0.88]	0.2%	0.9%
Pérez−Llamas F	34	57	0.60	[0.46; 0.72]	0.0%	0.8%
Petrenya N	606	2424	0.25	[0.23; 0.27]	0.6%	0.9%
Rabenberg M	714	1277	0.56	[0.53; 0.59]	0.3%	0.9%
Rabenberg M	919	1452	0.63	[0.61; 0.66]	0.3%	0.9%
Rabenberg M	632	906	0.70	[0.67; 0.73]	0.2%	0.9%
Raposo L	247	286	0.86	[0.82; 0.90]	0.1%	0.9%
Santos A	635	872	0.73	[0.70; 0.76]	0.2%	0.9%
Vierucci F	102	214	0.48	[0.41; 0.55]	0.1%	0.9%
Abiaka C	98	105	0.93	[0.87; 0.97]	0.0%	0.8%
Al Hayek S	49	172	0.28	[0.22; 0.36]	0.0%	0.9%
Al Zarooni AAR	6524	7785	0.84	[0.83; 0.85]	1.8%	0.9%
Al−Daghri NM	960	1038	0.92	[0.91; 0.94]	0.2%	0.9%
Al−Daghri NM	346	462	0.75	[0.71; 0.79]	0.1%	0.9%
Al−Daghri NM	3207	4361	0.74	[0.72; 0.75]	1.0%	0.9%
AlQuaiz AM	1191	1870	0.64	[0.61; 0.66]	0.4%	0.9%
Arabi A	220	295	0.75	[0.69; 0.79]	0.1%	0.9%
Asakura K	47	54	0.87	[0.75; 0.95]	0.0%	0.8%
Batieha A	492	3462	0.14	[0.13; 0.15]	0.8%	0.9%
Bener A	162	230	0.70	[0.64; 0.76]	0.1%	0.9%
Chailurkit LO	122	1320	0.09	[0.08; 0.11]	0.3%	0.9%
Chen J	1350	3066	0.44	[0.42; 0.46]	0.7%	0.9%
Chung JY	7292	10348	0.70	[0.70; 0.71]	2.5%	0.9%
Cinar N	45	53	0.85	[0.72; 0.93]	0.0%	0.8%
El−Khateeb M	2176	2771	0.79	[0.77; 0.80]	0.7%	0.9%
Fang F	645	1052	0.61	[0.58; 0.64]	0.2%	0.9%
Feng X	359	376	0.95	[0.93; 0.97]	0.1%	0.9%
Gariballa S	233	491	0.47	[0.43; 0.52]	0.1%	0.9%
Gökta O	2344	9146	0.26	[0.25; 0.27]	2.2%	0.9%
Gromova O	646	819	0.79	[0.76; 0.82]	0.2%	0.9%
Ho−Pham LT	13	432	0.03	[0.02; 0.05]	0.1%	0.9%
Ho−Pham LT	1	432	0.00	[0.00; 0.01]	0.1%	0.9%
Hovsepian S	368	818	0.45	[0.42; 0.48]	0.2%	0.9%
Jayashri R	502	832	0.60	[0.57; 0.64]	0.2%	0.9%
Jayatissa R	932	1437	0.65	[0.62; 0.67]	0.3%	0.9%
Kapil U	163	187	0.87	[0.82; 0.92]	0.0%	0.9%
Kapil U	400	428	0.93	[0.91; 0.96]	0.1%	0.9%
Karagüzel G	146	182	0.80	[0.74; 0.86]	0.0%	0.9%
Kaykhaei MA	200	562	0.36	[0.32; 0.40]	0.1%	0.9%
Koyama S	83	245	0.34	[0.28; 0.40]	0.1%	0.9%
Lee J	25549	31698	0.81	[0.80; 0.81]	7.5%	0.9%
Leung RY	1579	3693	0.43	[0.41; 0.44]	0.9%	0.9%
Liu X	51	108	0.47	[0.38; 0.57]	0.0%	0.8%
Mallah EM	195	201	0.97	[0.94; 0.99]	0.0%	0.9%
Mehboobali N	386	507	0.76	[0.72; 0.80]	0.1%	0.9%
Metwally ASM	1230	1315	0.94	[0.92; 0.95]	0.3%	0.9%
Meyer HE	60	111	0.54	[0.44; 0.64]	0.0%	0.8%
Moussavi M	119	165	0.72	[0.65; 0.79]	0.0%	0.9%
Naeem Z	21	97	0.22	[0.14; 0.31]	0.0%	0.8%
Nikooyeh B	326	345	0.94	[0.92; 0.97]	0.1%	0.9%
Saki F	199	263	0.76	[0.70; 0.81]	0.1%	0.9%
Saliba W	1967	3128	0.63	[0.61; 0.65]	0.7%	0.9%
Sharawat IK	28	45	0.62	[0.47; 0.76]	0.0%	0.8%
Sherchand O	109	191	0.57	[0.50; 0.64]	0.0%	0.9%
Suryanarayana P	54	99	0.55	[0.44; 0.65]	0.0%	0.8%
Yan X	95	126	0.75	[0.67; 0.83]	0.0%	0.8%
Zargar AH	27	28	0.96	[0.82; 1.00]	0.0%	0.8%
Zhang FF	443	524	0.85	[0.81; 0.88]	0.1%	0.9%
Zhao Y	1151	1571	0.73	[0.71; 0.75]	0.4%	0.9%
Zhen D	5687	7136	0.80	[0.79; 0.81]	1.7%	0.9%
Zhu Z	196	815	0.24	[0.21; 0.27]	0.2%	0.9%
Zhu Z	184	421	0.44	[0.39; 0.49]	0.1%	0.9%
Zhu Z	23	43	0.53	[0.38; 0.69]	0.0%	0.8%
**Common effect model**		**421692**	**0.56**	**[0.55; 0.56]**	**100.0%**	**−−**
**Random effects model**			**0.53**	**[0.48; 0.59]**	**−−**	**100.0%**

Heterogeneity: $I^2 = 100\%$, $\tau^2 = 0.0812$, $p = 0$

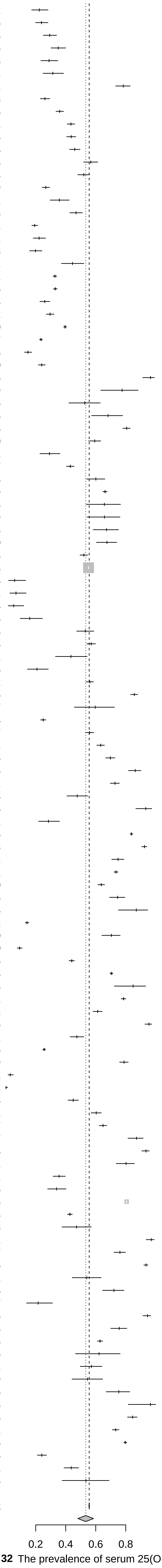

Supplementary figure 32 The prevalence of serum 25(OH)D < 50 nmol/L among females

Appendix 10: The prevalence of vitamin D deficiency by gender

Study	Events	Total	Proportion	95%−CI	Weight (common)	Weight (random)
Black LJ	101	158	0.64	[0.56; 0.71]	0.2%	1.3%
Black LJ	146	216	0.68	[0.61; 0.74]	0.3%	1.3%
Black LJ	238	297	0.80	[0.75; 0.85]	0.4%	1.3%
Black LJ	170	262	0.65	[0.59; 0.71]	0.3%	1.3%
Black LJ	155	227	0.68	[0.62; 0.74]	0.3%	1.3%
Black LJ	107	140	0.76	[0.69; 0.83]	0.2%	1.3%
Daly RM	348	590	0.59	[0.55; 0.63]	0.7%	1.3%
Daly RM	715	1092	0.65	[0.63; 0.68]	1.3%	1.3%
Daly RM	951	1343	0.71	[0.68; 0.73]	1.6%	1.3%
Daly RM	668	923	0.72	[0.69; 0.75]	1.1%	1.3%
Daly RM	499	730	0.68	[0.65; 0.72]	0.9%	1.3%
Daly RM	268	362	0.74	[0.69; 0.78]	0.4%	1.3%
Cairncross CT	588	681	0.86	[0.84; 0.89]	0.8%	1.3%
Gill TK	692	1164	0.59	[0.57; 0.62]	1.4%	1.3%
Flores ME	1259	1741	0.72	[0.70; 0.74]	2.1%	1.3%
Greene−Finestone LS	200	401	0.50	[0.45; 0.55]	0.5%	1.3%
Greene−Finestone LS	251	384	0.65	[0.60; 0.70]	0.5%	1.3%
Greene−Finestone LS	99	130	0.76	[0.68; 0.83]	0.2%	1.3%
Yousef S	4597	5774	0.80	[0.79; 0.81]	7.0%	1.3%
de Oliveira CL	247	449	0.55	[0.50; 0.60]	0.5%	1.3%
Orces CH	599	1072	0.56	[0.53; 0.59]	1.3%	1.3%
Liu X	9803	12610	0.78	[0.77; 0.78]	15.3%	1.3%
Mansbach JM	551	904	0.61	[0.58; 0.64]	1.1%	1.3%
Mansbach JM	977	1359	0.72	[0.69; 0.74]	1.6%	1.3%
Bettencourt A	78	103	0.76	[0.66; 0.84]	0.1%	1.2%
Capuano R	533	600	0.89	[0.86; 0.91]	0.7%	1.3%
Feketea GM	116	184	0.63	[0.56; 0.70]	0.2%	1.3%
Hansen L	243	264	0.92	[0.88; 0.95]	0.3%	1.3%
Hansen L	922	1048	0.88	[0.86; 0.90]	1.3%	1.3%
Hintzpeter B	4019	4582	0.88	[0.87; 0.89]	5.6%	1.3%
Hribar M	41	52	0.79	[0.65; 0.89]	0.1%	1.2%
Hribar M	65	76	0.86	[0.76; 0.93]	0.1%	1.2%
Karin Z	109	132	0.83	[0.75; 0.89]	0.2%	1.3%
Madsen KH	43	85	0.51	[0.40; 0.62]	0.1%	1.2%
Madsen KH	43	77	0.56	[0.44; 0.67]	0.1%	1.2%
Madsen KH	46	96	0.48	[0.38; 0.58]	0.1%	1.2%
Madsen KH	58	111	0.52	[0.43; 0.62]	0.1%	1.3%
Man PW	90	103	0.87	[0.79; 0.93]	0.1%	1.2%
Manios Y	1122	1179	0.95	[0.94; 0.96]	1.4%	1.3%
Petrenya N	1519	2041	0.74	[0.72; 0.76]	2.5%	1.3%
Rabenberg M	1010	1179	0.86	[0.84; 0.88]	1.4%	1.3%
Rabenberg M	1141	1271	0.90	[0.88; 0.91]	1.5%	1.3%
Rabenberg M	834	910	0.92	[0.90; 0.93]	1.1%	1.3%
Vierucci F	173	213	0.81	[0.75; 0.86]	0.3%	1.3%
Wyskida M	1502	1814	0.83	[0.81; 0.85]	2.2%	1.3%
Abiaka C	98	101	0.97	[0.92; 0.99]	0.1%	1.2%
Al Hayek S	108	172	0.63	[0.55; 0.70]	0.2%	1.3%
Al Zarooni AAR	4369	4561	0.96	[0.95; 0.96]	5.5%	1.3%
AlQuaiz AM	862	962	0.90	[0.88; 0.91]	1.2%	1.3%
Arabi A	159	171	0.93	[0.88; 0.96]	0.2%	1.3%
Bachhel R	66	75	0.88	[0.78; 0.94]	0.1%	1.2%
Batieha A	57	1128	0.05	[0.04; 0.06]	1.4%	1.3%
Chailurkit LO	431	1321	0.33	[0.30; 0.35]	1.6%	1.3%
Choi HR	261	269	0.97	[0.94; 0.99]	0.3%	1.3%
Chung JY	7149	7957	0.90	[0.89; 0.91]	9.7%	1.3%
Cinar N	51	63	0.81	[0.69; 0.90]	0.1%	1.2%
El−Khateeb M	1093	1183	0.92	[0.91; 0.94]	1.4%	1.3%
Feng X	60	310	0.19	[0.15; 0.24]	0.4%	1.3%
Gromova O	480	528	0.91	[0.88; 0.93]	0.6%	1.3%
Ho−Pham LT	41	205	0.20	[0.15; 0.26]	0.2%	1.3%
Hovsepian S	172	243	0.71	[0.65; 0.76]	0.3%	1.3%
Kapil U	403	420	0.96	[0.94; 0.98]	0.5%	1.3%
Kapil U	181	187	0.97	[0.93; 0.99]	0.2%	1.3%
Karagüzel G	119	189	0.63	[0.56; 0.70]	0.2%	1.3%
Kaykhaei MA	408	431	0.95	[0.92; 0.97]	0.5%	1.3%
Koyama S	241	247	0.98	[0.95; 0.99]	0.3%	1.3%
Leung RY	1462	1583	0.92	[0.91; 0.94]	1.9%	1.3%
Mallah EM	98	99	0.99	[0.95; 1.00]	0.1%	1.2%
Mehboobali N	281	351	0.80	[0.75; 0.84]	0.4%	1.3%
Naeem Z	73	83	0.88	[0.79; 0.94]	0.1%	1.2%
Saki F	229	241	0.95	[0.91; 0.97]	0.3%	1.3%
Saliba W	2109	2790	0.76	[0.74; 0.77]	3.4%	1.3%
Sherchand O	67	109	0.61	[0.52; 0.71]	0.1%	1.3%
Song HR	3407	3587	0.95	[0.94; 0.96]	4.4%	1.3%
Zhao Y	998	1090	0.92	[0.90; 0.93]	1.3%	1.3%
Zhu Z	119	135	0.88	[0.81; 0.93]	0.2%	1.3%
Zhu Z	905	1019	0.89	[0.87; 0.91]	1.2%	1.3%
Zhu Z	984	1454	0.68	[0.65; 0.70]	1.8%	1.3%
**Common effect model**		**82393**	**0.81**	**[0.81; 0.81]**	**100.0%**	**−−**
**Random effects model**			**0.77**	**[0.73; 0.82]**	**−−**	**100.0%**

Heterogeneity: $I^2 = 99\%$, $\tau^2 = 0.0531$, $p = 0$

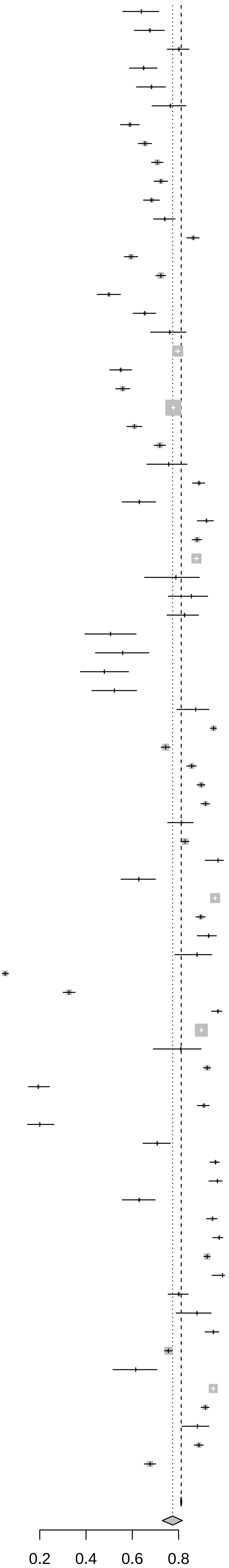

Supplementary figure 33 The prevalence of serum 25(OH)D < 75 nmol/L among males

Appendix 10: The prevalence of vitamin D deficiency by gender

Study	Events	Total	Proportion	95%−CI	Weight (common)	Weight (random)
Black LJ	149	241	0.62	[0.55; 0.68]	0.2%	1.3%
Black LJ	292	436	0.67	[0.62; 0.71]	0.4%	1.3%
Black LJ	296	433	0.68	[0.64; 0.73]	0.4%	1.3%
Black LJ	285	386	0.74	[0.69; 0.78]	0.4%	1.3%
Black LJ	193	263	0.73	[0.68; 0.79]	0.3%	1.3%
Black LJ	138	191	0.72	[0.65; 0.78]	0.2%	1.3%
Daly RM	520	803	0.65	[0.61; 0.68]	0.8%	1.3%
Daly RM	1108	1464	0.76	[0.73; 0.78]	1.4%	1.3%
Daly RM	1251	1541	0.81	[0.79; 0.83]	1.5%	1.3%
Daly RM	930	1088	0.85	[0.83; 0.88]	1.1%	1.3%
Daly RM	736	834	0.88	[0.86; 0.90]	0.8%	1.3%
Daly RM	406	448	0.91	[0.88; 0.93]	0.4%	1.3%
Cairncross CT	585	648	0.90	[0.88; 0.92]	0.6%	1.3%
Gill TK	861	1249	0.69	[0.66; 0.71]	1.2%	1.3%
Flores ME	1353	1741	0.78	[0.76; 0.80]	1.7%	1.3%
Greene−Finestone LS	265	422	0.63	[0.58; 0.67]	0.4%	1.3%
Greene−Finestone LS	233	393	0.59	[0.54; 0.64]	0.4%	1.3%
Greene−Finestone LS	157	182	0.86	[0.80; 0.91]	0.2%	1.3%
Yousef S	4213	5805	0.73	[0.71; 0.74]	5.6%	1.3%
de Oliveira CL	499	703	0.71	[0.67; 0.74]	0.7%	1.3%
Orces CH	1008	1302	0.77	[0.75; 0.80]	1.3%	1.3%
Liu X	10008	13400	0.75	[0.74; 0.75]	13.0%	1.3%
Mansbach JM	589	895	0.66	[0.63; 0.69]	0.9%	1.3%
Mansbach JM	1050	1400	0.75	[0.73; 0.77]	1.4%	1.3%
Bettencourt A	77	95	0.81	[0.72; 0.88]	0.1%	1.2%
Capuano R	520	600	0.87	[0.84; 0.89]	0.6%	1.3%
Feketea GM	133	192	0.69	[0.62; 0.76]	0.2%	1.3%
Hansen L	240	263	0.91	[0.87; 0.94]	0.3%	1.3%
Hansen L	1123	1517	0.74	[0.72; 0.76]	1.5%	1.3%
Hintzpeter B	3892	4419	0.88	[0.87; 0.89]	4.3%	1.3%
Hribar M	63	73	0.86	[0.76; 0.93]	0.1%	1.2%
Hribar M	66	79	0.84	[0.74; 0.91]	0.1%	1.2%
Karin Z	118	128	0.92	[0.86; 0.96]	0.1%	1.3%
Madsen KH	57	94	0.61	[0.50; 0.71]	0.1%	1.2%
Madsen KH	53	84	0.63	[0.52; 0.73]	0.1%	1.2%
Madsen KH	53	102	0.52	[0.42; 0.62]	0.1%	1.2%
Madsen KH	54	106	0.51	[0.41; 0.61]	0.1%	1.2%
Man PW	242	313	0.77	[0.72; 0.82]	0.3%	1.3%
Manios Y	1144	1174	0.97	[0.96; 0.98]	1.1%	1.3%
Petrenya N	1796	2424	0.74	[0.72; 0.76]	2.4%	1.3%
Rabenberg M	1054	1277	0.83	[0.80; 0.85]	1.2%	1.3%
Rabenberg M	1324	1452	0.91	[0.90; 0.93]	1.4%	1.3%
Rabenberg M	853	906	0.94	[0.92; 0.96]	0.9%	1.3%
Vierucci F	178	214	0.83	[0.77; 0.88]	0.2%	1.3%
Wyskida M	1500	1658	0.90	[0.89; 0.92]	1.6%	1.3%
Abiaka C	104	105	0.99	[0.95; 1.00]	0.1%	1.2%
Al Hayek S	107	172	0.62	[0.55; 0.69]	0.2%	1.3%
Al Zarooni AAR	7376	7785	0.95	[0.94; 0.95]	7.6%	1.3%
AlQuaiz AM	1553	1870	0.83	[0.81; 0.85]	1.8%	1.3%
Arabi A	268	295	0.91	[0.87; 0.94]	0.3%	1.3%
Bachhel R	69	75	0.92	[0.83; 0.97]	0.1%	1.2%
Batieha A	1291	3462	0.37	[0.36; 0.39]	3.4%	1.3%
Chailurkit LO	756	1320	0.57	[0.55; 0.60]	1.3%	1.3%
Choi HR	365	382	0.96	[0.93; 0.97]	0.4%	1.3%
Chung JY	9824	10348	0.95	[0.94; 0.95]	10.1%	1.3%
Cinar N	63	65	0.97	[0.89; 1.00]	0.1%	1.2%
El−Khateeb M	2455	2771	0.89	[0.87; 0.90]	2.7%	1.3%
Feng X	54	376	0.14	[0.11; 0.18]	0.4%	1.3%
Gromova O	751	819	0.92	[0.90; 0.93]	0.8%	1.3%
Ho−Pham LT	199	432	0.46	[0.41; 0.51]	0.4%	1.3%
Hovsepian S	588	818	0.72	[0.69; 0.75]	0.8%	1.3%
Kapil U	426	428	1.00	[0.98; 1.00]	0.4%	1.3%
Kapil U	184	187	0.98	[0.95; 1.00]	0.2%	1.3%
Karagüzel G	199	572	0.35	[0.31; 0.39]	0.6%	1.3%
Kaykhaei MA	532	562	0.95	[0.92; 0.96]	0.5%	1.3%
Koyama S	244	245	1.00	[0.98; 1.00]	0.2%	1.3%
Leung RY	3290	3693	0.89	[0.88; 0.90]	3.6%	1.3%
Mallah EM	200	201	1.00	[0.97; 1.00]	0.2%	1.3%
Mehboobali N	492	507	0.97	[0.95; 0.98]	0.5%	1.3%
Naeem Z	48	97	0.49	[0.39; 0.60]	0.1%	1.2%
Saki F	229	263	0.87	[0.82; 0.91]	0.3%	1.3%
Saliba W	3028	3128	0.97	[0.96; 0.97]	3.0%	1.3%
Sherchand O	169	191	0.88	[0.83; 0.93]	0.2%	1.3%
Song HR	5313	5389	0.99	[0.98; 0.99]	5.2%	1.3%
Zhao Y	1525	1571	0.97	[0.96; 0.98]	1.5%	1.3%
Zhu Z	42	43	0.98	[0.88; 1.00]	0.0%	1.2%
Zhu Z	366	421	0.87	[0.83; 0.90]	0.4%	1.3%
Zhu Z	571	815	0.70	[0.67; 0.73]	0.8%	1.3%
**Common effect model**		**102846**	**0.84**	**[0.84; 0.85]**	**100.0%**	**−−**
**Random effects model**			**0.82**	**[0.78; 0.85]**	**−−**	**100.0%**

Heterogeneity: $I^2 = 100\%$, $\tau^2 = 0.0479$, $p = 0$

Appendix 10

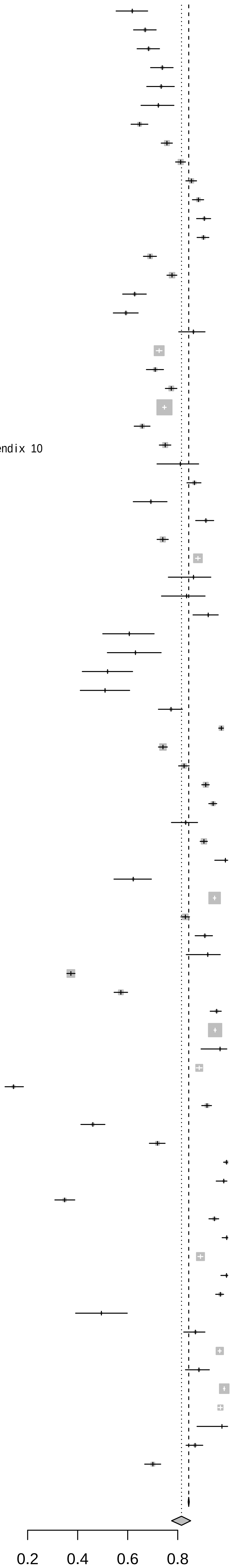

Supplementary figure 34 The prevalence of serum 25(OH)D < 75 nmol/L among females

Appendix 10: the Risk Ratio of prevalence among females and males

Study	female Events	female Total	male Events	male Total	RR	95%−CI	Weight (common)	Weight (random)
Tran B	37	301	29	342	1.45	[0.91; 2.30]	0.0%	1.2%
Daly RM	31	803	14	590	1.63	[0.87; 3.03]	0.0%	1.0%
Daly RM	88	1464	20	1092	3.28	[2.03; 5.30]	0.0%	1.2%
Daly RM	80	1541	31	1343	2.25	[1.50; 3.38]	0.0%	1.2%
Daly RM	28	1088	49	923	0.48	[0.31; 0.76]	0.0%	1.2%
Daly RM	62	834	13	730	4.17	[2.32; 7.53]	0.0%	1.0%
Daly RM	56	448	10	362	4.53	[2.34; 8.74]	0.0%	1.0%
Cairncross CT	41	648	35	681	1.23	[0.79; 1.91]	0.0%	1.2%
Gill TK	16	1249	5	1164	2.98	[1.10; 8.11]	0.0%	0.6%
Black LJ	4	241	10	158	0.26	[0.08; 0.82]	0.0%	0.5%
Black LJ	25	436	16	216	0.77	[0.42; 1.42]	0.0%	1.0%
Black LJ	21	433	7	297	2.06	[0.89; 4.78]	0.0%	0.8%
Black LJ	21	386	10	262	1.43	[0.68; 2.98]	0.0%	0.9%
Black LJ	15	263	8	227	1.62	[0.70; 3.75]	0.0%	0.8%
Black LJ	8	191	7	140	0.84	[0.31; 2.26]	0.0%	0.7%
Mansbach JM	8	895	9	904	0.90	[0.35; 2.32]	0.0%	0.7%
Mansbach JM	28	1400	13	1359	2.09	[1.09; 4.02]	0.0%	1.0%
Greene−Finestone LS	4	422	16	401	0.24	[0.08; 0.70]	0.0%	0.6%
Greene−Finestone LS	8	393	10	384	0.78	[0.31; 1.96]	0.0%	0.7%
Greene−Finestone LS	5	182	3	130	1.19	[0.29; 4.89]	0.0%	0.4%
Sarafin K	334	5852	532	5848	0.63	[0.55; 0.72]	0.0%	1.5%
Yousef S	549	5805	643	5774	0.85	[0.76; 0.95]	0.1%	1.5%
Herrick KA	466	8167	353	8013	1.30	[1.13; 1.48]	0.0%	1.5%
Andersen R	97	115	61	95	1.31	[1.11; 1.56]	0.0%	1.5%
Bjarnadottir A	3	85	2	73	1.29	[0.22; 7.50]	0.0%	0.3%
Borissova AM	287	1068	143	948	1.78	[1.49; 2.13]	0.0%	1.4%
Capuano R	80	600	57	600	1.40	[1.02; 1.93]	0.0%	1.3%
Crowe FL	266596	650233	269517	619579	0.94	[0.94; 0.95]	22.0%	1.5%
Crowe FL	260228	677677	271453	638713	0.90	[0.90; 0.91]	22.3%	1.5%
Crowe FL	201340	567154	242108	612932	0.90	[0.89; 0.90]	18.5%	1.5%
Crowe FL	136516	436154	163749	465198	0.89	[0.88; 0.89]	12.6%	1.5%
Crowe FL	104348	376707	118838	378467	0.88	[0.88; 0.89]	9.4%	1.5%
Crowe FL	171430	567649	128300	426246	1.00	[1.00; 1.01]	11.7%	1.5%
Granlund L	15	105	12	111	1.32	[0.65; 2.69]	0.0%	0.9%
Hansen L	27	263	53	264	0.51	[0.33; 0.79]	0.0%	1.2%
Hansen L	152	1517	157	1048	0.67	[0.54; 0.82]	0.0%	1.4%
Hintzpeter B	877	4419	920	4582	0.99	[0.91; 1.07]	0.1%	1.5%
Hribar M	20	73	12	52	1.19	[0.64; 2.21]	0.0%	1.0%
Hribar M	22	79	15	76	1.41	[0.79; 2.51]	0.0%	1.0%
Karin Z	19	128	13	132	1.51	[0.78; 2.92]	0.0%	1.0%
Laird E	315	1553	327	1364	0.85	[0.74; 0.97]	0.0%	1.5%
Lin LY	32180	240263	28507	208338	0.98	[0.96; 0.99]	2.4%	1.5%
Madsen KH	0	102	2	96	0.19	[0.01; 3.87]	0.0%	0.1%
Madsen KH	1	106	1	111	1.05	[0.07; 16.53]	0.0%	0.1%
Man PW	34	313	6	103	1.86	[0.81; 4.31]	0.0%	0.8%
Manios Y	84	1174	38	1179	2.22	[1.53; 3.23]	0.0%	1.3%
Nälsén C	7	144	2	124	3.01	[0.64; 14.24]	0.0%	0.4%
Nälsén C	7	92	3	114	2.89	[0.77; 10.87]	0.0%	0.5%
Nielsen NO	293	1621	232	1256	0.98	[0.84; 1.14]	0.0%	1.5%
Patel JV	577	916	678	988	0.92	[0.86; 0.98]	0.1%	1.5%
Pérez−Llamas F	23	57	6	29	1.95	[0.89; 4.25]	0.0%	0.8%
Petrenya N	17	2424	12	2041	1.19	[0.57; 2.49]	0.0%	0.9%
Petrenya N	46	2424	37	2041	1.05	[0.68; 1.61]	0.0%	1.2%
Rabenberg M	369	1277	387	1179	0.88	[0.78; 0.99]	0.0%	1.5%
Rabenberg M	418	1452	386	1271	0.95	[0.84; 1.06]	0.0%	1.5%
Rabenberg M	298	906	242	910	1.24	[1.07; 1.43]	0.0%	1.5%
Raposo L	107	286	81	214	0.99	[0.79; 1.24]	0.0%	1.4%
Santos A	400	872	194	628	1.48	[1.29; 1.70]	0.0%	1.5%
Wyskida M	144	1658	211	1814	0.75	[0.61; 0.91]	0.0%	1.4%
Zhu Z	9	815	17	1454	0.94	[0.42; 2.11]	0.0%	0.8%
Zhu Z	13	421	16	1019	1.97	[0.95; 4.05]	0.0%	0.9%
Zhu Z	2	43	3	135	2.09	[0.36; 12.12]	0.0%	0.3%
Choi HR	67	382	43	269	1.10	[0.77; 1.56]	0.0%	1.3%
Sharawat IK	17	45	17	55	1.22	[0.71; 2.11]	0.0%	1.1%
Bachhel R	39	75	21	75	1.86	[1.22; 2.84]	0.0%	1.2%
Hovsepian S	139	868	73	243	0.53	[0.42; 0.68]	0.0%	1.4%
Nikooyeh B	549	751	424	655	1.13	[1.05; 1.21]	0.0%	1.5%
Nikooyeh B	241	345	133	322	1.69	[1.46; 1.96]	0.0%	1.5%
Saki F	38	263	25	241	1.39	[0.87; 2.24]	0.0%	1.2%
Saliba W	720	3128	171	2790	3.76	[3.20; 4.40]	0.0%	1.5%
Bater J	2900	4764	2792	4828	1.05	[1.02; 1.09]	0.2%	1.5%
Jayatissa R	255	1437	79	1178	2.65	[2.08; 3.36]	0.0%	1.4%
Gromova O	283	819	88	528	2.07	[1.68; 2.56]	0.0%	1.4%
AlQuaiz AM	512	1870	217	962	1.21	[1.06; 1.39]	0.0%	1.5%
Al−Daghri NM	488	1038	230	1187	2.43	[2.12; 2.77]	0.0%	1.5%
Al−Daghri NM	170	462	65	368	2.08	[1.62; 2.68]	0.0%	1.4%
Al−Daghri NM	1545	4361	620	2999	1.71	[1.58; 1.86]	0.1%	1.5%
Abiaka C	55	105	24	101	2.20	[1.49; 3.27]	0.0%	1.3%
Karagüzel G	54	182	21	189	2.67	[1.68; 4.23]	0.0%	1.2%
Chen J	367	3066	364	2948	0.97	[0.85; 1.11]	0.0%	1.5%
Yan X	42	126	39	176	1.50	[1.04; 2.18]	0.0%	1.3%
Fang F	101	1052	38	762	1.93	[1.34; 2.76]	0.0%	1.3%
Li H	1605	5179	1258	5517	1.36	[1.28; 1.45]	0.1%	1.5%
Asakura K	25	54	15	53	1.64	[0.98; 2.74]	0.0%	1.1%
Al Hayek S	15	172	18	172	0.83	[0.43; 1.60]	0.0%	1.0%
Arabi A	152	295	30	171	2.94	[2.08; 4.14]	0.0%	1.3%
Zhang FF	234	524	114	436	1.71	[1.42; 2.05]	0.0%	1.4%
**Common effect model**		**3607750**		**3434089**	**0.92**	**[0.92; 0.93]**	**100.0%**	**−−**
**Random effects model**					**1.29**	**[1.16; 1.44]**	**−−**	**100.0%**

Heterogeneity: I^2 = 97%, τ^2 = 0.1937, p = 0

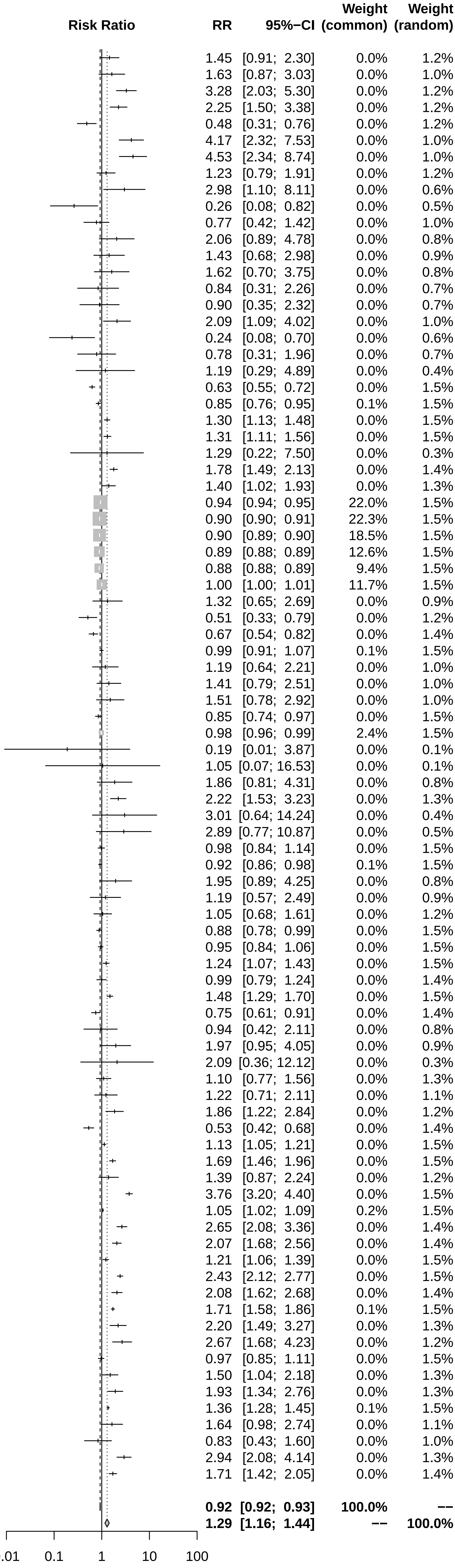

Supplementary figure 35 The Risk Ratio of serum 25(OH)D < 30 nmol/L among females and males

Appendix 10: the Risk Ratio of prevalence among females and males

Study	Experimental Events	Experimental Total	Control Events	Control Total	RR	95%−CI	Weight (common)	Weight (random)
Black LJ	54	241	45	158	0.79	[0.56; 1.11]	0.0%	0.8%
Black LJ	104	436	62	216	0.83	[0.64; 1.09]	0.0%	0.8%
Black LJ	127	433	65	297	1.34	[1.03; 1.74]	0.0%	0.8%
Black LJ	135	386	59	262	1.55	[1.19; 2.02]	0.0%	0.8%
Black LJ	76	263	71	227	0.92	[0.70; 1.21]	0.0%	0.8%
Black LJ	60	191	36	140	1.22	[0.86; 1.74]	0.0%	0.7%
Tran B	236	301	247	342	1.09	[0.99; 1.19]	0.1%	0.9%
Daly RM	210	803	98	590	1.57	[1.27; 1.95]	0.1%	0.9%
Daly RM	527	1464	235	1092	1.67	[1.47; 1.91]	0.1%	0.9%
Daly RM	670	1541	313	1343	1.87	[1.67; 2.09]	0.2%	0.9%
Daly RM	475	1088	267	923	1.51	[1.34; 1.70]	0.1%	0.9%
Daly RM	384	834	161	730	2.09	[1.79; 2.44]	0.1%	0.9%
Daly RM	254	448	100	362	2.05	[1.71; 2.47]	0.1%	0.9%
Cairncross CT	337	648	295	681	1.20	[1.07; 1.34]	0.1%	0.9%
Gill TK	334	1249	215	1164	1.45	[1.24; 1.68]	0.1%	0.9%
Djennane M	83	232	47	203	1.55	[1.14; 2.09]	0.0%	0.8%
Carrillo−Vega MF	271	578	145	550	1.78	[1.51; 2.10]	0.1%	0.9%
Flores ME	336	1741	263	1741	1.28	[1.10; 1.48]	0.1%	0.9%
Greene−Finestone LS	94	422	62	401	1.44	[1.08; 1.93]	0.0%	0.8%
Greene−Finestone LS	78	393	96	384	0.79	[0.61; 1.03]	0.0%	0.8%
Greene−Finestone LS	81	182	51	130	1.13	[0.87; 1.48]	0.0%	0.8%
Sarafin K	1920	5852	2386	5848	0.80	[0.77; 0.84]	1.2%	1.0%
Yousef S	1918	5805	2289	5774	0.83	[0.79; 0.87]	1.1%	1.0%
de Oliveira CL	183	703	72	449	1.62	[1.27; 2.08]	0.0%	0.8%
Orces CH	385	1302	123	1072	2.58	[2.14; 3.10]	0.1%	0.9%
Liu X	5304	13400	4460	12610	1.12	[1.08; 1.15]	2.2%	1.0%
Herrick KA	1920	8167	1851	8013	1.02	[0.96; 1.08]	0.9%	1.0%
Mansbach JM	133	895	135	904	1.00	[0.80; 1.24]	0.1%	0.9%
Mansbach JM	336	1400	257	1359	1.27	[1.10; 1.47]	0.1%	0.9%
Andersen R	111	115	90	95	1.02	[0.96; 1.08]	0.0%	1.0%
Andersen S	38	49	30	48	1.24	[0.95; 1.62]	0.0%	0.8%
Bettencourt A	50	95	45	103	1.20	[0.90; 1.61]	0.0%	0.8%
Bjarnadottir A	58	85	45	73	1.11	[0.88; 1.40]	0.0%	0.9%
Borissova AM	861	1068	667	948	1.15	[1.09; 1.21]	0.3%	1.0%
Capuano R	356	600	330	600	1.08	[0.98; 1.19]	0.2%	0.9%
Feketea GM	56	192	46	184	1.17	[0.84; 1.63]	0.0%	0.8%
Hansen L	653	1517	629	1048	0.72	[0.66; 0.77]	0.4%	1.0%
Hansen L	158	263	164	264	0.97	[0.84; 1.11]	0.1%	0.9%
Hintzpeter B	2925	4419	2970	4582	1.02	[0.99; 1.05]	1.4%	1.0%
Hribar M	48	73	26	52	1.32	[0.96; 1.81]	0.0%	0.8%
Hribar M	52	79	46	76	1.09	[0.85; 1.38]	0.0%	0.8%
Karin Z	86	128	65	132	1.36	[1.10; 1.69]	0.0%	0.9%
Kull M Jr	135	200	133	167	0.85	[0.75; 0.96]	0.1%	0.9%
Laird E	809	1553	837	1364	0.85	[0.80; 0.90]	0.4%	1.0%
Lin LY	132603	240263	115875	208338	0.99	[0.99; 1.00]	60.7%	1.0%
Madsen KH	5	84	4	77	1.15	[0.32; 4.11]	0.0%	0.2%
Madsen KH	7	102	8	96	0.82	[0.31; 2.18]	0.0%	0.3%
Madsen KH	5	94	4	85	1.13	[0.31; 4.07]	0.0%	0.2%
Madsen KH	17	106	11	111	1.62	[0.80; 3.29]	0.0%	0.4%
Man PW	166	313	70	103	0.78	[0.66; 0.92]	0.1%	0.9%
Manios Y	669	1174	566	1179	1.19	[1.10; 1.28]	0.3%	1.0%
Nälsén C	40	92	47	114	1.05	[0.77; 1.45]	0.0%	0.8%
Nälsén C	30	144	30	124	0.86	[0.55; 1.34]	0.0%	0.6%
Nielsen NO	908	1621	718	1256	0.98	[0.92; 1.05]	0.4%	1.0%
Patel JV	785	916	902	988	0.94	[0.91; 0.97]	0.4%	1.0%
Pérez−Llamas F	34	57	16	29	1.08	[0.73; 1.60]	0.0%	0.7%
Petrenya N	606	2424	500	2041	1.02	[0.92; 1.13]	0.3%	0.9%
Rabenberg M	714	1277	725	1179	0.91	[0.85; 0.97]	0.4%	1.0%
Rabenberg M	919	1452	781	1271	1.03	[0.97; 1.09]	0.4%	1.0%
Rabenberg M	632	906	569	910	1.12	[1.04; 1.19]	0.3%	1.0%
Raposo L	247	286	181	214	1.02	[0.95; 1.10]	0.1%	1.0%
Santos A	635	872	300	628	1.52	[1.39; 1.67]	0.2%	0.9%
Vierucci F	102	214	111	213	0.91	[0.76; 1.11]	0.1%	0.9%
Abiaka C	98	105	82	101	1.15	[1.03; 1.28]	0.0%	0.9%
Al Hayek S	49	172	60	172	0.82	[0.60; 1.12]	0.0%	0.8%
Al Zarooni AAR	6524	7785	3790	4561	1.01	[0.99; 1.03]	2.3%	1.0%
Al−Daghri NM	960	1038	941	1187	1.17	[1.13; 1.21]	0.4%	1.0%
Al−Daghri NM	346	462	273	368	1.01	[0.93; 1.09]	0.1%	0.9%
Al−Daghri NM	3207	4361	2183	2999	1.01	[0.98; 1.04]	1.3%	1.0%
AlQuaiz AM	1191	1870	695	962	0.88	[0.84; 0.93]	0.4%	1.0%
Arabi A	220	295	115	171	1.11	[0.98; 1.26]	0.1%	0.9%
Asakura K	47	54	41	53	1.13	[0.94; 1.34]	0.0%	0.9%
Batieha A	492	3462	17	1128	9.43	[5.84; 15.22]	0.0%	0.6%
Bener A	162	230	153	238	1.10	[0.97; 1.24]	0.1%	0.9%
Chailurkit LO	122	1320	25	1321	4.88	[3.20; 7.46]	0.0%	0.7%
Chen J	1350	3066	1141	2948	1.14	[1.07; 1.21]	0.6%	1.0%
Chung JY	7292	10348	4295	7957	1.31	[1.27; 1.34]	2.4%	1.0%
Cinar N	45	53	54	65	1.02	[0.87; 1.20]	0.0%	0.9%
El−Khateeb M	2176	2771	639	1183	1.45	[1.37; 1.54]	0.4%	1.0%
Fang F	645	1052	304	762	1.54	[1.39; 1.70]	0.2%	0.9%
Feng X	359	376	134	310	2.21	[1.94; 2.51]	0.1%	0.9%
Gariballa S	233	491	63	157	1.18	[0.96; 1.46]	0.0%	0.9%
Gökta O	2344	9146	1110	2592	0.60	[0.57; 0.63]	0.8%	1.0%
Gromova O	646	819	296	528	1.41	[1.29; 1.53]	0.2%	0.9%
Ho−Pham LT	13	432	2	205	3.08	[0.70; 13.54]	0.0%	0.2%
Ho−Pham LT	1	432	2	205	0.24	[0.02; 2.60]	0.0%	0.1%
Hovsepian S	368	818	127	243	0.86	[0.75; 0.99]	0.1%	0.9%
Jayashri R	502	832	320	668	1.26	[1.14; 1.39]	0.2%	0.9%
Jayatissa R	932	1437	553	1178	1.38	[1.29; 1.48]	0.3%	1.0%
Kapil U	163	187	144	187	1.13	[1.03; 1.25]	0.1%	0.9%
Kapil U	400	428	281	420	1.40	[1.30; 1.50]	0.1%	1.0%
Karagüzel G	146	182	119	189	1.27	[1.12; 1.45]	0.1%	0.9%
Kaykhaei MA	200	562	346	431	0.44	[0.39; 0.50]	0.2%	0.9%
Koyama S	83	245	55	247	1.52	[1.14; 2.04]	0.0%	0.8%
Lee J	25549	31698	25446	36759	1.16	[1.15; 1.17]	11.5%	1.0%
Leung RY	1579	3693	730	1583	0.93	[0.87; 0.99]	0.5%	1.0%
Liu X	51	108	25	96	1.81	[1.23; 2.68]	0.0%	0.7%
Mallah EM	195	201	76	99	1.26	[1.13; 1.41]	0.0%	0.9%
Mehboobali N	386	507	115	351	2.32	[1.98; 2.72]	0.1%	0.9%
Metwally ASM	1230	1315	462	549	1.11	[1.07; 1.16]	0.3%	1.0%
Meyer HE	60	111	29	85	1.58	[1.13; 2.23]	0.0%	0.8%
Moussavi M	119	165	28	153	3.94	[2.78; 5.58]	0.0%	0.7%
Naeem Z	21	97	32	83	0.56	[0.35; 0.89]	0.0%	0.6%
Nikooyeh B	326	345	299	322	1.02	[0.98; 1.06]	0.2%	1.0%
Saki F	199	263	189	241	0.96	[0.88; 1.06]	0.1%	0.9%
Saliba W	1967	3128	1047	2790	1.68	[1.59; 1.77]	0.5%	1.0%
Sharawat IK	28	45	35	55	0.98	[0.72; 1.32]	0.0%	0.8%
Sherchand O	109	191	45	109	1.38	[1.07; 1.78]	0.0%	0.8%
Suryanarayana P	54	99	107	187	0.95	[0.77; 1.19]	0.0%	0.9%
Yan X	95	126	121	176	1.10	[0.95; 1.26]	0.0%	0.9%
Zargar AH	27	28	49	64	1.26	[1.08; 1.47]	0.0%	0.9%
Zhang FF	443	524	352	436	1.05	[0.99; 1.11]	0.2%	1.0%
Zhao Y	1151	1571	526	1090	1.52	[1.42; 1.63]	0.3%	1.0%
Zhen D	5687	7136	1857	2902	1.25	[1.21; 1.28]	1.3%	1.0%
Zhu Z	196	815	302	1454	1.16	[0.99; 1.36]	0.1%	0.9%
Zhu Z	184	421	398	1019	1.12	[0.98; 1.28]	0.1%	0.9%
Zhu Z	23	43	60	135	1.20	[0.86; 1.68]	0.0%	0.8%
**Common effect model**		**421692**		**361131**	**1.05**	**[1.04; 1.05]**	**100.0%**	**−−**
**Random effects model**					**1.19**	**[1.12; 1.27]**	**−−**	**100.0%**

Heterogeneity: $I^2 = 97\%$, $\tau^2 = 0.1066$, $p = 0$

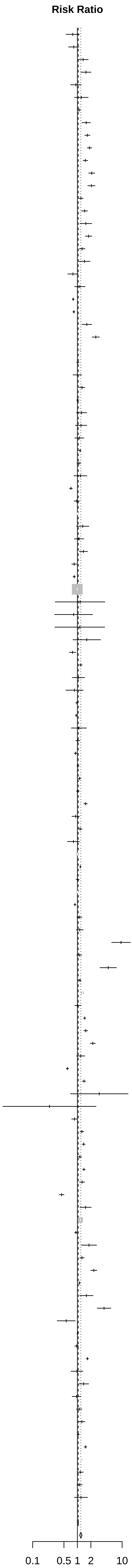

Supplementary figure 36 The Risk Ratio of serum 25(OH)D < 50 nmol/L among females and males

Appendix 10: the Risk Ratio of prevalence among females and males

Study	Experimental Events	Experimental Total	Control Events	Control Total	RR	95%−CI	Weight (common)	Weight (random)
Black LJ	149	241	101	158	0.97	[0.83; 1.13]	0.2%	1.2%
Black LJ	292	436	146	216	0.99	[0.88; 1.11]	0.3%	1.3%
Black LJ	296	433	238	297	0.85	[0.78; 0.93]	0.4%	1.3%
Black LJ	285	386	170	262	1.14	[1.02; 1.27]	0.3%	1.3%
Black LJ	193	263	155	227	1.07	[0.96; 1.21]	0.2%	1.3%
Black LJ	138	191	107	140	0.95	[0.83; 1.07]	0.2%	1.3%
Daly RM	520	803	348	590	1.10	[1.01; 1.19]	0.6%	1.3%
Daly RM	1108	1464	715	1092	1.16	[1.10; 1.22]	1.2%	1.3%
Daly RM	1251	1541	951	1343	1.15	[1.10; 1.20]	1.4%	1.3%
Daly RM	930	1088	668	923	1.18	[1.13; 1.24]	1.0%	1.3%
Daly RM	736	834	499	730	1.29	[1.22; 1.36]	0.7%	1.3%
Daly RM	406	448	268	362	1.22	[1.14; 1.31]	0.4%	1.3%
Cairncross CT	585	648	588	681	1.05	[1.01; 1.09]	0.8%	1.3%
Gill TK	861	1249	692	1164	1.16	[1.09; 1.23]	1.0%	1.3%
Flores ME	1353	1741	1259	1741	1.07	[1.03; 1.12]	1.8%	1.3%
Greene−Finestone LS	265	422	200	401	1.26	[1.11; 1.42]	0.3%	1.3%
Greene−Finestone LS	233	393	251	384	0.91	[0.81; 1.01]	0.4%	1.3%
Greene−Finestone LS	157	182	99	130	1.13	[1.01; 1.27]	0.2%	1.3%
Yousef S	4213	5805	4597	5774	0.91	[0.89; 0.93]	6.5%	1.3%
de Oliveira CL	499	703	247	449	1.29	[1.17; 1.42]	0.4%	1.3%
Orces CH	1008	1302	599	1072	1.39	[1.30; 1.47]	0.9%	1.3%
Liu X	10008	13400	9803	12610	0.96	[0.95; 0.97]	14.2%	1.3%
Mansbach JM	589	895	551	904	1.08	[1.01; 1.16]	0.8%	1.3%
Mansbach JM	1050	1400	977	1359	1.04	[1.00; 1.09]	1.4%	1.3%
Bettencourt A	77	95	78	103	1.07	[0.92; 1.24]	0.1%	1.2%
Capuano R	520	600	533	600	0.98	[0.94; 1.02]	0.8%	1.3%
Feketea GM	133	192	116	184	1.10	[0.95; 1.27]	0.2%	1.2%
Hansen L	240	263	243	264	0.99	[0.94; 1.04]	0.3%	1.3%
Hansen L	1123	1517	922	1048	0.84	[0.81; 0.87]	1.5%	1.3%
Hintzpeter B	3892	4419	4019	4582	1.00	[0.99; 1.02]	5.6%	1.3%
Hribar M	63	73	41	52	1.09	[0.93; 1.29]	0.1%	1.2%
Hribar M	66	79	65	76	0.98	[0.85; 1.12]	0.1%	1.2%
Karin Z	118	128	109	132	1.12	[1.02; 1.23]	0.2%	1.3%
Madsen KH	57	94	43	85	1.20	[0.92; 1.56]	0.1%	1.0%
Madsen KH	53	84	43	77	1.13	[0.87; 1.46]	0.1%	1.0%
Madsen KH	53	102	46	96	1.08	[0.82; 1.43]	0.1%	1.0%
Madsen KH	54	106	58	111	0.97	[0.75; 1.26]	0.1%	1.0%
Man PW	242	313	90	103	0.88	[0.80; 0.97]	0.2%	1.3%
Manios Y	1144	1174	1122	1179	1.02	[1.01; 1.04]	1.6%	1.3%
Petrenya N	1796	2424	1519	2041	1.00	[0.96; 1.03]	2.3%	1.3%
Rabenberg M	1054	1277	1010	1179	0.96	[0.93; 1.00]	1.5%	1.3%
Rabenberg M	1324	1452	1141	1271	1.02	[0.99; 1.04]	1.7%	1.3%
Rabenberg M	853	906	834	910	1.03	[1.00; 1.05]	1.2%	1.3%
Vierucci F	178	214	173	213	1.02	[0.94; 1.12]	0.2%	1.3%
Wyskida M	1500	1658	1502	1814	1.09	[1.06; 1.12]	2.0%	1.3%
Abiaka C	104	105	98	101	1.02	[0.98; 1.06]	0.1%	1.3%
Al Hayek S	107	172	108	172	0.99	[0.84; 1.17]	0.2%	1.2%
Al Zarooni AAR	7376	7785	4369	4561	0.99	[0.98; 1.00]	7.8%	1.3%
AlQuaiz AM	1553	1870	862	962	0.93	[0.90; 0.95]	1.6%	1.3%
Arabi A	268	295	159	171	0.98	[0.92; 1.03]	0.3%	1.3%
Bachhel R	69	75	66	75	1.05	[0.94; 1.16]	0.1%	1.3%
Batieha A	1291	3462	57	1128	7.38	[5.71; 9.54]	0.1%	1.0%
Chailurkit LO	756	1320	431	1321	1.76	[1.60; 1.92]	0.6%	1.3%
Choi HR	365	382	261	269	0.98	[0.96; 1.01]	0.4%	1.3%
Chung JY	9824	10348	7149	7957	1.06	[1.05; 1.07]	11.4%	1.3%
Cinar N	63	65	51	63	1.20	[1.05; 1.36]	0.1%	1.3%
El−Khateeb M	2455	2771	1093	1183	0.96	[0.94; 0.98]	2.2%	1.3%
Feng X	54	376	60	310	0.74	[0.53; 1.04]	0.1%	0.9%
Gromova O	751	819	480	528	1.01	[0.98; 1.04]	0.8%	1.3%
Ho−Pham LT	199	432	41	205	2.30	[1.72; 3.08]	0.1%	1.0%
Hovsepian S	588	818	172	243	1.02	[0.93; 1.11]	0.4%	1.3%
Kapil U	426	428	403	420	1.04	[1.02; 1.06]	0.6%	1.3%
Kapil U	184	187	181	187	1.02	[0.98; 1.05]	0.3%	1.3%
Karagüzel G	199	572	119	189	0.55	[0.47; 0.65]	0.3%	1.2%
Kaykhaei MA	532	562	408	431	1.00	[0.97; 1.03]	0.7%	1.3%
Koyama S	244	245	241	247	1.02	[1.00; 1.04]	0.3%	1.3%
Leung RY	3290	3693	1462	1583	0.96	[0.95; 0.98]	2.9%	1.3%
Mallah EM	200	201	98	99	1.01	[0.98; 1.03]	0.2%	1.3%
Mehboobali N	492	507	281	351	1.21	[1.15; 1.28]	0.5%	1.3%
Naeem Z	48	97	73	83	0.56	[0.45; 0.70]	0.1%	1.1%
Saki F	229	263	229	241	0.92	[0.87; 0.97]	0.3%	1.3%
Saliba W	3028	3128	2109	2790	1.28	[1.25; 1.31]	3.1%	1.3%
Sherchand O	169	191	67	109	1.44	[1.23; 1.68]	0.1%	1.2%
Song HR	5313	5389	3407	3587	1.04	[1.03; 1.05]	5.8%	1.3%
Zhao Y	1525	1571	998	1090	1.06	[1.04; 1.08]	1.7%	1.3%
Zhu Z	42	43	119	135	1.11	[1.03; 1.20]	0.1%	1.3%
Zhu Z	366	421	905	1019	0.98	[0.94; 1.02]	0.7%	1.3%
Zhu Z	571	815	984	1454	1.04	[0.98; 1.10]	1.0%	1.3%
**Common effect model**		**102846**		**82393**	**1.04**	**[1.03; 1.04]**	**100.0%**	**−−**
**Random effects model**					**1.07**	**[1.01; 1.13]**	**−−**	**100.0%**

Heterogeneity: $I^2 = 96\%$, $\tau^2 = 0.0573$, $p = 0$

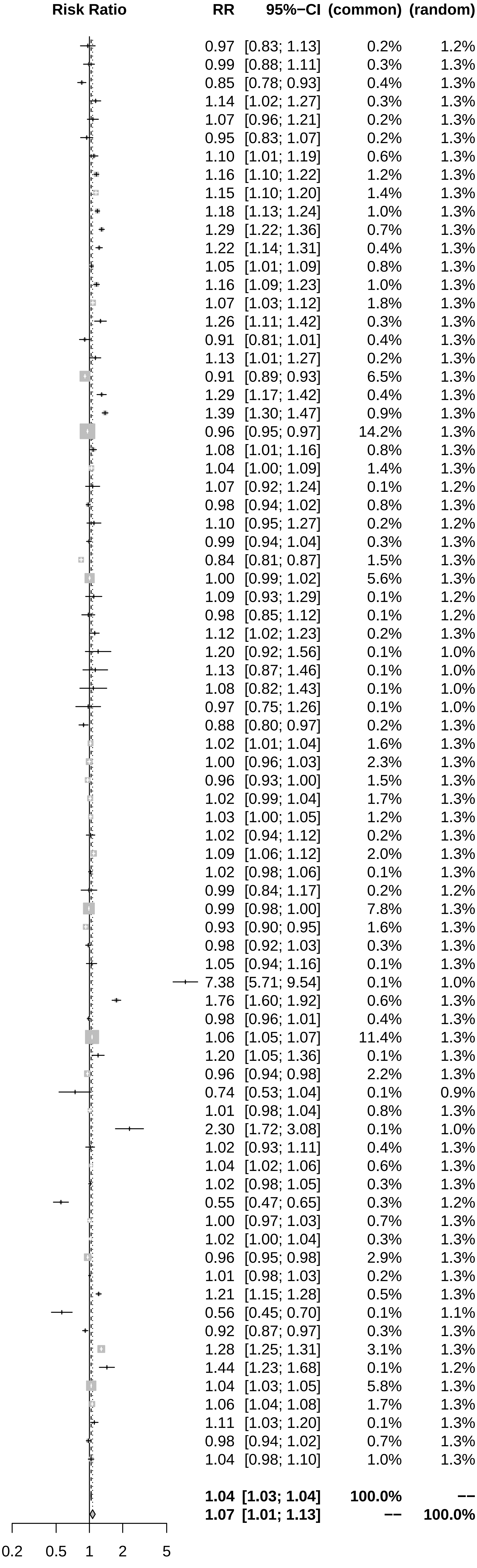

Supplementary figure 37 The Risk Ratio of serum 25(OH)D < 75 nmol/L among females and males

Appendix 11: the Risk Ratio of prevalence in Winter-spring and Summer-autumn

Study	Winter-spring Events	Winter-spring Total	Summer-autumn Events	Summer-autumn Total	RR	95%−CI	Weight (common)	Weight (random)
Vierucci F 2014	147	248	66	178	1.60	[1.29; 1.99]	0.1%	4.9%
Hribar M 2020	131	164	40	116	2.32	[1.78; 3.01]	0.0%	4.7%
Johnson MA 2008	10	41	8	39	1.19	[0.52; 2.70]	0.0%	2.4%
Beer RJ 2020	3608	11606	4263	20235	1.48	[1.42; 1.53]	2.5%	5.3%
Cougnard−Grégoire A 2015	363	1160	217	1159	1.67	[1.44; 1.94]	0.2%	5.1%
de Oliveira CL 2020	197	769	53	383	1.85	[1.40; 2.44]	0.1%	4.7%
Zhou SJ 2015	23	115	21	106	1.01	[0.59; 1.71]	0.0%	3.5%
Hirani V 2013	415	843	254	816	1.58	[1.40; 1.79]	0.2%	5.2%
Gill TK 2014	393	1356	157	1062	1.96	[1.66; 2.32]	0.1%	5.1%
Daly RM 2012	2758	7516	1049	3850	1.35	[1.27; 1.43]	1.1%	5.3%
Black LJ 2021	705	1938	211	1312	2.26	[1.97; 2.59]	0.2%	5.2%
Ní Chaoimh C 2018	157	347	41	394	4.35	[3.18; 5.94]	0.0%	4.5%
Djennane M 2014	169	408	130	435	1.39	[1.15; 1.67]	0.1%	5.0%
Saliba W 2012	32349	54561	18749	44508	1.41	[1.39; 1.43]	16.4%	5.3%
Middelkoop K 2022	104	965	32	850	2.86	[1.95; 4.21]	0.0%	4.2%
Lin LY 2021	153627	220789	95280	227812	1.66	[1.65; 1.67]	74.4%	5.3%
Gökta.. O 2020	5345	7047	3296	4687	1.08	[1.05; 1.10]	3.1%	5.3%
Byun EJ 2017	913	1050	930	1465	1.37	[1.31; 1.43]	0.6%	5.3%
Lin L 2021	36	466	13	1244	7.39	[3.96; 13.81]	0.0%	3.1%
Karagüzel G 2014	349	375	265	371	1.30	[1.21; 1.40]	0.2%	5.3%
Chen J 2017	534	1264	1802	4750	1.11	[1.03; 1.20]	0.6%	5.3%
**Common effect model**		**313028**		**315772**	**1.59**	**[1.58; 1.60]**	**100.0%**	**−−**
**Random effects model**					**1.70**	**[1.44; 2.02]**	**−−**	**100.0%**

Heterogeneity: $I^2 = 99\%$, $\tau^2 = 0.1400$, $p = 0$

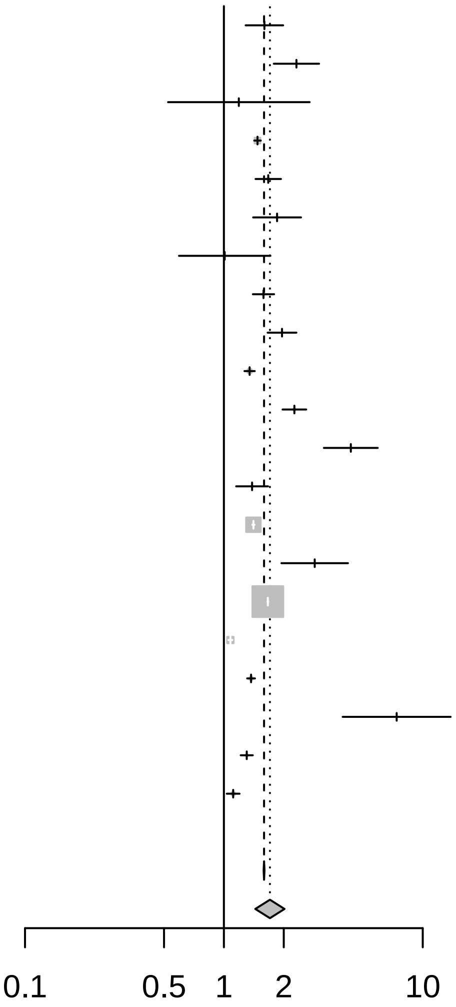

Supplementary figure 38 The Risk Ratio of serum 25(OH)D < 50 nmol/L in Winter-spring and Summer-autumn

Appendix 12: Meta-regression analyses

Supplementary Table 9 The univariate meta-regression analyses on the prevalence of serum 25-hydroxyvitamin D levels less than 30 nmol/l.

	**Beta (95% CI)**	***p* Values**	**R^2**
Latitude	-0.0246[-0.0449, -0.0042]	0.0181	1.80%
Year of publication	-0.0920[-0.1455, -0.0385]	0.0007	3.79%
World Bank income groups	-0.0003[-0.0244, 0.0238]	0.9813	0.00%
WHO regions	-0.0542[-0.0735, -0.0349]	<0.0001	10.30%
Age	0.0144[-0.0427, 0.0715]	0.6208	0.00%
Female (%)	-0.0083[-0.0318, 0.0152]	0.4884	0.00%
Diagnostic method	-0.0113[-0.0276, -0.0382]	<0.0001	0.35%
Time of data collection	0.0120[-0.0164, 0.0405]	0.4072	0.00%
Sampling method	0.1015[0.0448, 0.1581]	0.0004	4.19%
Study area	-0.0726[-0.1168, -0.0283]	0.0013	3.46%
population	-0.0974[-0.1518, -0.0431]	0.0004	4.18%
Risk of bias	-0.1056[-0.1718, -0.0393]	0.0018	3.32%

CI: confidence interval.

Appendix 12: Meta-regression analyses

Supplementary Table 10 The univariate meta-regression analyses on the prevalence of serum 25-hydroxyvitamin D levels less than 50 nmol/l.

	**Beta (95% CI)**	***p* Values**	**R^2**
Latitude	-0.0324[-0.0517, -0.0131]	0.001	2.62%
Year of publication	-0.1142[-0.1723, -0.0561]	0.0001	3.58%
World Bank income groups	-0.0243[-0.0474, -0.0012]	0.0393	0.85%
WHO regions	-0.0271[-0.0466, -0.0077]	0.0062	1.73%
Age	0.0314[-0.0285, 0.0914]	0.3039	0.01%
Female (%)	-0.0298[-0.0550, -0.0046]	0.0203	1.18%
Diagnostic method	-0.0208[-0.0377, -0.0039]	0.0156	1.32%
Time of data collection	0.0071[-0.0223, 0.0365]	0.6364	0.00%
Sampling method	0.0901[0.0302, 0.1500]	0.0032	2.06%
Study area	-0.0615[-0.1080, -0.0149]	0.0096	1.49%
population	0.1020[0.0588, 0.1452]	<0.0001	5.16%
Risk of bias	0.1307[0.0573, 0.2042]	0.0005	2.99%
Season	-0.1829[-0.3371, -0.0287]	0.0201	9.80%

CI: confidence interval.

Appendix 13: Sensitivity analyses

Study	Proportion	95%−CI
Omitting Beer RJ	0.16	[0.14; 0.18]
Omitting Beer RJ	0.16	[0.14; 0.18]
Omitting Beer RJ	0.16	[0.14; 0.18]
Omitting Beer RJ	0.16	[0.14; 0.18]
Omitting Chin KY	0.16	[0.14; 0.18]
Omitting Gebreegziabher T	0.16	[0.14; 0.18]
Omitting Jayatissa R	0.16	[0.14; 0.18]
Omitting Lin L	0.16	[0.14; 0.18]
Omitting Lin L	0.16	[0.14; 0.18]
Omitting Lin L	0.16	[0.14; 0.18]
Omitting Mechenro J	0.16	[0.14; 0.18]
Omitting Mechenro J	0.16	[0.14; 0.18]
Omitting Mechenro J	0.16	[0.14; 0.18]
Omitting Meyer HE	0.16	[0.14; 0.18]
Omitting Paul TV	0.16	[0.14; 0.18]
Omitting Shetty S	0.16	[0.14; 0.18]
Omitting Shivane VK	0.16	[0.14; 0.18]
Omitting Smith G	0.16	[0.14; 0.18]
Omitting Smith G	0.16	[0.14; 0.18]
Omitting Abdulrahman MA	0.16	[0.14; 0.18]
Omitting Abdulrahman MA	0.16	[0.14; 0.18]
Omitting Abdulrahman MA	0.16	[0.14; 0.18]
Omitting Abiaka C	0.16	[0.14; 0.18]
Omitting Al Hayek S	0.16	[0.14; 0.18]
Omitting Al Shaikh A	0.16	[0.14; 0.18]
Omitting Al Shaikh AM	0.16	[0.14; 0.18]
Omitting Al Shaikh AM	0.16	[0.14; 0.18]
Omitting Al−Daghri NM	0.16	[0.14; 0.18]
Omitting Al−Daghri NM	0.16	[0.14; 0.18]
Omitting Al−Daghri NM	0.16	[0.14; 0.18]
Omitting Al−Daghri NM	0.16	[0.14; 0.18]
Omitting Al−Daghri NM	0.16	[0.14; 0.18]
Omitting Al−Daghri NM	0.16	[0.14; 0.18]
Omitting Al−Kindi MK	0.16	[0.14; 0.18]
Omitting AlQuaiz AM	0.16	[0.14; 0.18]
Omitting Al−Saleh Y	0.16	[0.14; 0.18]
Omitting Al−Taiar A	0.16	[0.14; 0.18]
Omitting Alyahya K	0.16	[0.14; 0.18]
Omitting Alyahya KO	0.15	[0.13; 0.18]
Omitting Arabi A	0.16	[0.14; 0.18]
Omitting Ardawi MS	0.16	[0.14; 0.18]
Omitting Ardawi MS	0.16	[0.14; 0.18]
Omitting Arya V	0.16	[0.14; 0.18]
Omitting Bachhel R	0.16	[0.14; 0.18]
Omitting Bezrati I	0.16	[0.14; 0.18]
Omitting Bhatt SP	0.16	[0.14; 0.18]
Omitting Capuano R	0.16	[0.14; 0.18]
Omitting Chakrabarty S	0.16	[0.14; 0.18]
Omitting Chakrabarty S	0.16	[0.14; 0.18]
Omitting Chen J	0.16	[0.14; 0.18]
Omitting Choi HR	0.16	[0.14; 0.18]
Omitting Djennane M	0.16	[0.14; 0.18]
Omitting Drali O	0.16	[0.14; 0.18]
Omitting Duarte C	0.16	[0.14; 0.18]
Omitting Fang F	0.16	[0.14; 0.18]
Omitting Golbahar J	0.16	[0.14; 0.18]
Omitting Goswami R	0.15	[0.13; 0.18]
Omitting Hashemipour S	0.16	[0.14; 0.18]
Omitting Hatun S	0.16	[0.14; 0.18]
Omitting Herrick KA	0.16	[0.14; 0.18]
Omitting Herrick KA	0.16	[0.14; 0.18]
Omitting Herrick KA	0.16	[0.14; 0.18]
Omitting Herrick KA	0.16	[0.14; 0.18]
Omitting Herrick KA	0.16	[0.14; 0.18]
Omitting Herrick KA	0.16	[0.14; 0.18]
Omitting Hovsepian S	0.16	[0.14; 0.18]
Omitting Hutchings N	0.16	[0.14; 0.18]
Omitting Islam MZ	0.16	[0.14; 0.18]
Omitting Islam MZ	0.16	[0.14; 0.18]
Omitting Jiang W	0.16	[0.14; 0.18]
Omitting Joukar F	0.16	[0.14; 0.18]
Omitting Kaddam IM	0.16	[0.14; 0.18]
Omitting Kaddam IM	0.16	[0.14; 0.18]
Omitting Kim SY	0.16	[0.14; 0.18]
Omitting Li H	0.16	[0.14; 0.18]
Omitting Li H	0.16	[0.14; 0.18]
Omitting Mallah EM	0.16	[0.14; 0.18]
Omitting Manios Y	0.16	[0.14; 0.18]
Omitting Mansbach JM	0.16	[0.14; 0.18]
Omitting Mansbach JM	0.16	[0.14; 0.18]
Omitting Masoud MS	0.16	[0.14; 0.18]
Omitting Meddeb N	0.16	[0.14; 0.18]
Omitting Nakamura K	0.16	[0.14; 0.18]
Omitting Nakhaee S	0.16	[0.14; 0.18]
Omitting Niafar M	0.16	[0.14; 0.18]
Omitting Nichols EK	0.16	[0.14; 0.18]
Omitting Nikooyeh B	0.16	[0.14; 0.18]
Omitting Nikooyeh B	0.16	[0.14; 0.18]
Omitting Öztürk ZA	0.16	[0.14; 0.18]
Omitting Pérez−Llamas F	0.16	[0.14; 0.18]
Omitting Qorbani M	0.16	[0.14; 0.18]
Omitting Rodríguez−Rodríguez E	0.16	[0.14; 0.18]
Omitting Saeed BQ	0.16	[0.14; 0.18]
Omitting Saki F	0.16	[0.14; 0.18]
Omitting Saliba W	0.16	[0.14; 0.18]
Omitting Saliba W	0.16	[0.14; 0.18]
Omitting Santos A	0.16	[0.14; 0.18]
Omitting Sharawat IK	0.16	[0.14; 0.18]
Omitting Sulimani RA	0.16	[0.14; 0.18]
Omitting Tseng M	0.16	[0.14; 0.18]
Omitting Yan X	0.16	[0.14; 0.18]
Omitting Yu S	0.16	[0.14; 0.18]
Omitting Zhang FF	0.16	[0.14; 0.18]
Omitting Zhu Z	0.16	[0.14; 0.18]
Omitting Zhu Z	0.16	[0.14; 0.18]
Omitting Zhu Z	0.16	[0.14; 0.18]
Omitting Alkerwi A	0.16	[0.14; 0.18]
Omitting Andersen R	0.16	[0.14; 0.18]
Omitting Andersen R	0.16	[0.14; 0.18]
Omitting Andersen R	0.16	[0.14; 0.18]
Omitting Andersen R	0.16	[0.14; 0.18]
Omitting Andersen R	0.16	[0.14; 0.18]
Omitting Andersen R	0.16	[0.14; 0.18]
Omitting Arnljots R	0.16	[0.14; 0.18]
Omitting Asakura K	0.16	[0.14; 0.18]
Omitting Aspell N	0.16	[0.14; 0.18]
Omitting Aucoin M	0.16	[0.14; 0.18]
Omitting Aucoin M	0.16	[0.14; 0.18]
Omitting Bater J	0.16	[0.14; 0.18]
Omitting Borissova AM	0.16	[0.14; 0.18]
Omitting Cashman KD	0.16	[0.14; 0.18]
Omitting Ceccarelli M	0.16	[0.14; 0.18]
Omitting Chao YS	0.16	[0.14; 0.18]
Omitting Chirita−Emandi A	0.16	[0.14; 0.18]
Omitting Chlebna−Sokó.. D	0.16	[0.14; 0.18]
Omitting Cougnard−Grégoire A	0.16	[0.14; 0.18]
Omitting Crowe FL	0.16	[0.14; 0.18]
Omitting Crowe FL	0.16	[0.14; 0.18]
Omitting Crowe FL	0.16	[0.14; 0.18]
Omitting Crowe FL	0.16	[0.14; 0.18]
Omitting Crowe FL	0.16	[0.14; 0.18]
Omitting Crowe FL	0.16	[0.14; 0.18]
Omitting El Hayek J	0.16	[0.14; 0.18]
Omitting Fernández Bustillo JM	0.16	[0.14; 0.18]
Omitting Ganmaa D	0.15	[0.14; 0.18]
Omitting Ginter JK	0.16	[0.14; 0.18]
Omitting Glatt DU	0.16	[0.14; 0.18]
Omitting Greene−Finestone LS	0.16	[0.14; 0.18]
Omitting Greene−Finestone LS	0.16	[0.14; 0.18]
Omitting Greene−Finestone LS	0.16	[0.14; 0.18]
Omitting Griffin TP	0.16	[0.14; 0.18]
Omitting Gromova O	0.16	[0.14; 0.18]
Omitting Hansen L	0.16	[0.14; 0.18]
Omitting Hansen L	0.16	[0.14; 0.18]
Omitting Hintzpeter B	0.16	[0.14; 0.18]
Omitting Hirani V	0.16	[0.14; 0.18]
Omitting Hoge A	0.16	[0.14; 0.18]
Omitting Hribar M	0.16	[0.14; 0.18]
Omitting Hribar M	0.16	[0.14; 0.18]
Omitting Jääskeläinen T	0.16	[0.14; 0.18]
Omitting Jolliffe DA	0.16	[0.14; 0.18]
Omitting Jorde R	0.16	[0.14; 0.18]
Omitting Karagüzel G	0.16	[0.14; 0.18]
Omitting Karin Z	0.16	[0.14; 0.18]
Omitting Kunz C	0.16	[0.14; 0.18]
Omitting Kunz C	0.16	[0.14; 0.18]
Omitting Kunz C	0.16	[0.14; 0.18]
Omitting Kunz C	0.16	[0.14; 0.18]
Omitting Laird E	0.16	[0.14; 0.18]
Omitting Langlois K	0.16	[0.14; 0.18]
Omitting Lardner E	0.16	[0.14; 0.18]
Omitting Le Goaziou MF	0.16	[0.14; 0.18]
Omitting Lin LY	0.16	[0.14; 0.18]
Omitting Madsen KH	0.16	[0.14; 0.18]
Omitting Maguire JL	0.16	[0.14; 0.18]
Omitting Man PW	0.16	[0.14; 0.18]
Omitting Matheï C	0.16	[0.14; 0.18]
Omitting Mitchell DM	0.16	[0.14; 0.18]
Omitting Moreno−Reyes R	0.16	[0.14; 0.18]
Omitting Nälsén C	0.16	[0.14; 0.18]
Omitting Nälsén C	0.16	[0.14; 0.18]
Omitting NHANES (2015...2016)	0.16	[0.14; 0.18]
Omitting NHANES(2015...2016)	0.16	[0.14; 0.18]
Omitting NHANES(2015...2016)	0.16	[0.14; 0.18]
Omitting NHANES(2015...2016)	0.16	[0.14; 0.18]
Omitting NHANES(2017...2018)	0.16	[0.14; 0.18]
Omitting NHANES(2017...2018)	0.16	[0.14; 0.18]
Omitting NHANES(2017...2018)	0.16	[0.14; 0.18]
Omitting NHANES(2017...2018)	0.16	[0.14; 0.18]
Omitting Ní Chaoimh C	0.16	[0.14; 0.18]
Omitting Orwoll E	0.16	[0.14; 0.18]
Omitting Patel JV	0.16	[0.14; 0.18]
Omitting Perna L	0.16	[0.14; 0.18]
Omitting Rabenberg M	0.16	[0.14; 0.18]
Omitting Rabenberg M	0.16	[0.14; 0.18]
Omitting Rabenberg M	0.16	[0.14; 0.18]
Omitting Rabufetti A	0.16	[0.14; 0.18]
Omitting Raposo L	0.16	[0.14; 0.18]
Omitting Riverin B	0.16	[0.14; 0.18]
Omitting Riverin B	0.16	[0.14; 0.18]
Omitting Sarafin K	0.16	[0.14; 0.18]
Omitting Schramm S	0.16	[0.14; 0.18]
Omitting Science M	0.16	[0.14; 0.18]
Omitting Shchubelka K	0.16	[0.14; 0.18]
Omitting Sioen I	0.16	[0.14; 0.18]
Omitting Smith N	0.16	[0.14; 0.18]
Omitting Sochorová L	0.16	[0.14; 0.18]
Omitting Sokolovic S	0.16	[0.14; 0.18]
Omitting Sokolovic S	0.16	[0.14; 0.18]
Omitting Sokolovic S	0.16	[0.14; 0.18]
Omitting Souberbielle JC	0.16	[0.14; 0.18]
Omitting Thuesen B	0.16	[0.14; 0.18]
Omitting Tolppanen AM	0.16	[0.14; 0.18]
Omitting Tønnesen R	0.16	[0.14; 0.18]
Omitting Uush T	0.16	[0.14; 0.18]
Omitting Uush T	0.16	[0.14; 0.18]
Omitting Voortman T	0.16	[0.14; 0.18]
Omitting Wyskida M	0.16	[0.14; 0.18]
Omitting Yousef S	0.16	[0.14; 0.18]
Omitting Yousef S	0.16	[0.14; 0.18]
Omitting Yousef S	0.16	[0.14; 0.18]
Omitting Yousef S	0.16	[0.14; 0.18]
Omitting Yousef S	0.16	[0.14; 0.18]
Omitting Zgaga L	0.16	[0.14; 0.18]
Omitting Bjarnadottir A	0.16	[0.14; 0.18]
Omitting Courraud J	0.16	[0.14; 0.18]
Omitting Dalgård C	0.16	[0.14; 0.18]
Omitting El Hayek J	0.16	[0.14; 0.18]
Omitting Granlund L	0.16	[0.14; 0.18]
Omitting Ikonen H	0.16	[0.14; 0.18]
Omitting Nielsen NO	0.16	[0.14; 0.18]
Omitting Oberg J	0.16	[0.14; 0.18]
Omitting Petrenya N	0.16	[0.14; 0.18]
Omitting Samefors M	0.16	[0.14; 0.18]
Omitting Lima−Costa MF	0.16	[0.14; 0.18]
Omitting Maldonado G	0.16	[0.14; 0.18]
Omitting Black LJ	0.16	[0.14; 0.18]
Omitting Black LJ	0.16	[0.14; 0.18]
Omitting Black LJ	0.16	[0.14; 0.18]
Omitting Black LJ	0.16	[0.14; 0.18]
Omitting Black LJ	0.16	[0.14; 0.18]
Omitting Bolland MJ	0.16	[0.14; 0.18]
Omitting Brinkmann K	0.16	[0.14; 0.18]
Omitting Cairncross CT	0.16	[0.14; 0.18]
Omitting Daly RM	0.16	[0.14; 0.18]
Omitting Daly RM	0.16	[0.14; 0.18]
Omitting Daly RM	0.16	[0.14; 0.18]
Omitting Daly RM	0.16	[0.14; 0.18]
Omitting Daly RM	0.16	[0.14; 0.18]
Omitting Daly RM	0.16	[0.14; 0.18]
Omitting Gill TK	0.16	[0.14; 0.18]
Omitting Hirani V	0.16	[0.14; 0.18]
Omitting Hirani V	0.16	[0.14; 0.18]
Omitting Hirani V	0.16	[0.14; 0.18]
Omitting Hirani V	0.16	[0.14; 0.18]
Omitting Lategan R	0.16	[0.14; 0.18]
Omitting Lucas JA	0.16	[0.14; 0.18]
Omitting Oliveri B	0.16	[0.14; 0.18]
Omitting Robinson PJ	0.16	[0.14; 0.18]
Omitting Robinson PJ	0.16	[0.14; 0.18]
Omitting Saraiva GL	0.16	[0.14; 0.18]
Omitting Scalco R	0.16	[0.14; 0.18]
Omitting Skull SA	0.16	[0.14; 0.18]
Omitting Solis−Urra P	0.16	[0.14; 0.18]
Omitting Solis−Urra P	0.16	[0.14; 0.18]
Omitting Tran B	0.16	[0.14; 0.18]
Omitting Tran B	0.16	[0.14; 0.18]
Omitting Tran B	0.16	[0.14; 0.18]
Omitting Tran B	0.16	[0.14; 0.18]
Omitting Tran B	0.16	[0.14; 0.18]
Omitting von Hurst PR	0.16	[0.14; 0.18]
Omitting Zhou SJ	0.16	[0.14; 0.18]
**Random effects model**	**0.16**	**[0.14; 0.18]**

−0.15 −0.05 0 0.05 0.1 0.15

Supplementary figure 39 The sensitivity analyses for prevalence of serum 25-hydroxyvitamin D levels less than 30 nmol/l

Appendix 13: Sensitivity analyses

Study	Proportion	95%−CI
Omitting Houghton LA	0.48	[0.45; 0.51]
Omitting Smith G	0.48	[0.45; 0.51]
Omitting White Z	0.48	[0.45; 0.51]
Omitting Middelkoop K	0.48	[0.45; 0.51]
Omitting Lin L	0.48	[0.45; 0.51]
Omitting Middelkoop K	0.48	[0.45; 0.51]
Omitting Mutua AM	0.48	[0.45; 0.51]
Omitting Ten Haaf DSM	0.48	[0.45; 0.51]
Omitting Naqvi A	0.48	[0.45; 0.51]
Omitting Tangoh DA	0.48	[0.45; 0.51]
Omitting Miljkovic I	0.48	[0.45; 0.51]
Omitting Lategan R	0.48	[0.45; 0.51]
Omitting Ho−Pham LT	0.48	[0.45; 0.51]
Omitting Middelkoop K	0.48	[0.45; 0.51]
Omitting Lin L	0.48	[0.45; 0.51]
Omitting Riverin B	0.48	[0.45; 0.51]
Omitting Lin L	0.48	[0.45; 0.51]
Omitting Mechenro J	0.48	[0.45; 0.51]
Omitting Middelkoop K	0.48	[0.45; 0.51]
Omitting Shady MM	0.48	[0.45; 0.51]
Omitting Madsen KH	0.48	[0.45; 0.51]
Omitting Glatt DU	0.48	[0.45; 0.51]
Omitting Smith G	0.48	[0.45; 0.51]
Omitting Andersen R	0.48	[0.45; 0.51]
Omitting Kouda K	0.48	[0.45; 0.51]
Omitting Ginter JK	0.48	[0.45; 0.51]
Omitting Maguire JL	0.48	[0.45; 0.51]
Omitting Fayet−Moore F	0.48	[0.45; 0.51]
Omitting Abdulrahman MA	0.48	[0.45; 0.51]
Omitting Guo S	0.48	[0.45; 0.51]
Omitting Abdulrahman MA	0.48	[0.45; 0.51]
Omitting Bolland MJ	0.48	[0.45; 0.51]
Omitting Andersen R	0.48	[0.45; 0.51]
Omitting Middelkoop K	0.48	[0.45; 0.51]
Omitting Pan T	0.48	[0.45; 0.51]
Omitting Hatun S	0.48	[0.45; 0.51]
Omitting Yu L	0.48	[0.45; 0.51]
Omitting Madsen KH	0.48	[0.45; 0.51]
Omitting Yousef S	0.48	[0.45; 0.51]
Omitting Zhou SJ	0.48	[0.45; 0.51]
Omitting Kagotho E	0.48	[0.45; 0.51]
Omitting Andersen R	0.48	[0.45; 0.51]
Omitting Maldonado G	0.48	[0.45; 0.51]
Omitting Bi X	0.48	[0.45; 0.51]
Omitting Middelkoop K	0.48	[0.45; 0.51]
Omitting Srimani S	0.48	[0.45; 0.51]
Omitting Pérez−Llamas F	0.48	[0.45; 0.51]
Omitting Chin KY	0.48	[0.45; 0.51]
Omitting González G	0.48	[0.45; 0.51]
Omitting Rodríguez−Rodríguez E	0.48	[0.45; 0.51]
Omitting Naeem Z	0.48	[0.45; 0.51]
Omitting Gill TK	0.48	[0.45; 0.51]
Omitting El Hayek J	0.48	[0.45; 0.51]
Omitting Hazell TJ	0.48	[0.45; 0.51]
Omitting Nälsén C	0.48	[0.45; 0.51]
Omitting Greene−Finestone LS	0.48	[0.45; 0.51]
Omitting Sharawat IK	0.48	[0.45; 0.51]
Omitting Smith G	0.48	[0.45; 0.51]
Omitting Gill TK	0.48	[0.45; 0.51]
Omitting Andersen R	0.48	[0.45; 0.51]
Omitting Lardner E	0.48	[0.45; 0.51]
Omitting Andersen S	0.48	[0.45; 0.51]
Omitting Hribar M	0.48	[0.45; 0.51]
Omitting Wakayo T	0.48	[0.45; 0.51]
Omitting Cabral MA	0.48	[0.45; 0.51]
Omitting Ramakrishnan S	0.48	[0.45; 0.51]
Omitting Paul TV	0.48	[0.45; 0.51]
Omitting Tran B	0.48	[0.45; 0.51]
Omitting Zargar AH	0.48	[0.45; 0.51]
Omitting Liu X	0.48	[0.45; 0.51]
Omitting Gill TK	0.48	[0.45; 0.51]
Omitting Scalco R	0.48	[0.45; 0.51]
Omitting Hirani V	0.48	[0.45; 0.51]
Omitting Santos BR	0.48	[0.45; 0.51]
Omitting Zhu Z	0.48	[0.45; 0.51]
Omitting Bodin J	0.48	[0.45; 0.51]
Omitting Yu L	0.48	[0.45; 0.51]
Omitting Nälsén C	0.48	[0.45; 0.51]
Omitting Asakura K	0.48	[0.45; 0.51]
Omitting Hussain T	0.48	[0.45; 0.51]
Omitting Meyer HE	0.48	[0.45; 0.51]
Omitting Smith N	0.48	[0.45; 0.51]
Omitting Tran B	0.48	[0.45; 0.51]
Omitting Andersen R	0.48	[0.45; 0.51]
Omitting Mechenro J	0.48	[0.45; 0.51]
Omitting Bettencourt A	0.48	[0.45; 0.51]
Omitting Black LJ	0.48	[0.45; 0.51]
Omitting Alyahya KO	0.48	[0.45; 0.51]
Omitting Hribar M	0.48	[0.45; 0.51]
Omitting Vupputuri MR	0.48	[0.45; 0.51]
Omitting Cinar N	0.48	[0.45; 0.51]
Omitting Al−Kindi MK	0.48	[0.45; 0.51]
Omitting AlFaris NA	0.48	[0.45; 0.51]
Omitting Fernández Bustillo JM	0.48	[0.45; 0.51]
Omitting Tran B	0.48	[0.45; 0.51]
Omitting Feketea GM	0.48	[0.45; 0.51]
Omitting Herrick KA	0.48	[0.45; 0.51]
Omitting Bjarnadottir A	0.48	[0.45; 0.51]
Omitting Brinkmann K	0.48	[0.45; 0.51]
Omitting Johnson MA	0.48	[0.45; 0.51]
Omitting Tran B	0.48	[0.45; 0.51]
Omitting Gill TK	0.48	[0.45; 0.51]
Omitting Tran B	0.48	[0.45; 0.51]
Omitting Skull SA	0.48	[0.45; 0.51]
Omitting Al Hayek S	0.48	[0.45; 0.51]
Omitting Gill TK	0.48	[0.45; 0.51]
Omitting Mogire RM	0.48	[0.45; 0.51]
Omitting Horton−French K	0.48	[0.45; 0.51]
Omitting Horton−French K	0.48	[0.45; 0.51]
Omitting Abdulrahman MA	0.48	[0.45; 0.51]
Omitting Benjeddou K	0.48	[0.45; 0.51]
Omitting Orces CH	0.48	[0.45; 0.51]
Omitting Contreras−Manzano A	0.48	[0.45; 0.51]
Omitting Courraud J	0.48	[0.45; 0.51]
Omitting Meyer HE	0.48	[0.45; 0.51]
Omitting Saraiva GL	0.48	[0.45; 0.51]
Omitting Hirani V	0.48	[0.45; 0.51]
Omitting Contreras−Manzano A	0.48	[0.45; 0.51]
Omitting NHANES(2017...2018)	0.48	[0.45; 0.51]
Omitting Chao YS	0.48	[0.45; 0.51]
Omitting Gordon CM	0.48	[0.45; 0.51]
Omitting Djennane M	0.48	[0.45; 0.51]
Omitting Gill TK	0.48	[0.45; 0.51]
Omitting Shetty S	0.48	[0.45; 0.51]
Omitting Vallianou N	0.48	[0.45; 0.51]
Omitting Koyama S	0.48	[0.45; 0.51]
Omitting Muhairi SJ	0.48	[0.45; 0.51]
Omitting Janssen HC	0.48	[0.45; 0.51]
Omitting Jolliffe DA	0.48	[0.45; 0.51]
Omitting Black LJ	0.48	[0.45; 0.51]
Omitting Moussavi M	0.48	[0.45; 0.51]
Omitting Contreras−Manzano A	0.48	[0.45; 0.51]
Omitting Karin Z	0.48	[0.45; 0.51]
Omitting Chailurkit LO	0.48	[0.45; 0.51]
Omitting Science M	0.48	[0.45; 0.51]
Omitting Sherchand O	0.48	[0.45; 0.51]
Omitting Greene−Finestone LS	0.48	[0.45; 0.51]
Omitting Junaid K	0.48	[0.45; 0.51]
Omitting Granlund L	0.48	[0.45; 0.51]
Omitting Le Goaziou MF	0.48	[0.45; 0.51]
Omitting Harinarayan CV	0.48	[0.45; 0.51]
Omitting Suryanarayana P	0.48	[0.45; 0.51]
Omitting Orces CH	0.48	[0.45; 0.51]
Omitting Mechenro J	0.48	[0.45; 0.51]
Omitting Robinson PJ	0.48	[0.45; 0.51]
Omitting Vasudevan B	0.48	[0.45; 0.51]
Omitting Gebreegziabher T	0.48	[0.45; 0.51]
Omitting Masoud MS	0.48	[0.45; 0.51]
Omitting NHANES(2015...2016)	0.48	[0.45; 0.51]
Omitting Lappe JM	0.48	[0.45; 0.51]
Omitting Black LJ	0.48	[0.45; 0.51]
Omitting Islam MZ	0.48	[0.45; 0.51]
Omitting Sheikh A	0.48	[0.45; 0.51]
Omitting Greene−Finestone LS	0.48	[0.45; 0.51]
Omitting Santos BR	0.48	[0.45; 0.51]
Omitting Sokolovic S	0.48	[0.45; 0.51]
Omitting Rabufetti A	0.48	[0.45; 0.51]
Omitting Abiaka C	0.48	[0.45; 0.51]
Omitting Niafar M	0.48	[0.45; 0.51]
Omitting Al Shaikh AM	0.48	[0.45; 0.51]
Omitting Black LJ	0.48	[0.45; 0.51]
Omitting Black LJ	0.48	[0.45; 0.51]
Omitting von Hurst PR	0.48	[0.45; 0.51]
Omitting Bezrati I	0.48	[0.45; 0.51]
Omitting Batieha A	0.48	[0.45; 0.51]
Omitting Nadeem S	0.48	[0.45; 0.51]
Omitting Ní Chaoimh C	0.48	[0.45; 0.51]
Omitting Harkness LS	0.48	[0.45; 0.51]
Omitting Andersen R	0.48	[0.45; 0.51]
Omitting Kunz C	0.48	[0.45; 0.51]
Omitting Sioen I	0.48	[0.45; 0.51]
Omitting Rafraf M	0.48	[0.45; 0.51]
Omitting Gilbert−Diamond D	0.48	[0.45; 0.51]
Omitting Flores ME	0.48	[0.45; 0.51]
Omitting Orces CH	0.48	[0.45; 0.51]
Omitting Nakamura K	0.48	[0.45; 0.51]
Omitting Vierucci F	0.48	[0.45; 0.51]
Omitting Yan X	0.48	[0.45; 0.51]
Omitting Hirani V	0.48	[0.45; 0.51]
Omitting Sakyi SA	0.48	[0.45; 0.51]
Omitting Klenk J	0.48	[0.45; 0.51]
Omitting Oliveri B	0.48	[0.45; 0.51]
Omitting Alyahya K	0.48	[0.45; 0.51]
Omitting Yousef S	0.48	[0.45; 0.51]
Omitting Man PW	0.48	[0.45; 0.51]
Omitting Mitchell DM	0.48	[0.45; 0.51]
Omitting Matheï C	0.48	[0.45; 0.51]
Omitting Sochorová L	0.48	[0.45; 0.51]
Omitting Zhu W	0.48	[0.45; 0.51]
Omitting Gill TK	0.48	[0.45; 0.51]
Omitting Moy FM	0.48	[0.45; 0.51]
Omitting Mallah EM	0.48	[0.45; 0.51]
Omitting Karagüzel G	0.48	[0.45; 0.51]
Omitting Alloubani A	0.48	[0.45; 0.51]
Omitting Samefors M	0.48	[0.45; 0.51]
Omitting Kull M Jr	0.48	[0.45; 0.51]
Omitting Mansbach JM	0.48	[0.45; 0.51]
Omitting Khan AH	0.48	[0.45; 0.51]
Omitting Aucoin M	0.48	[0.45; 0.51]
Omitting Jääskeläinen T	0.48	[0.45; 0.51]
Omitting Herrick KA	0.48	[0.45; 0.51]
Omitting Kunz C	0.48	[0.45; 0.51]
Omitting Gariballa S	0.48	[0.45; 0.51]
Omitting Hirani V	0.48	[0.45; 0.51]
Omitting Majumdar V	0.48	[0.45; 0.51]
Omitting Hekimsoy Z	0.48	[0.45; 0.51]
Omitting Nakhaee S	0.48	[0.45; 0.51]
Omitting de Oliveira CL	0.48	[0.45; 0.51]
Omitting Yousef S	0.48	[0.45; 0.51]
Omitting Moreno−Reyes R	0.48	[0.45; 0.51]
Omitting Kapil U	0.48	[0.45; 0.51]
Omitting Daly RM	0.48	[0.45; 0.51]
Omitting Souberbielle JC	0.48	[0.45; 0.51]
Omitting Bener A	0.48	[0.45; 0.51]
Omitting Aucoin M	0.48	[0.45; 0.51]
Omitting Kunz C	0.48	[0.45; 0.51]
Omitting Hansen L	0.48	[0.45; 0.51]
Omitting Kunz C	0.48	[0.45; 0.51]
Omitting NHANES (2015...2016)	0.48	[0.45; 0.51]
Omitting González−Molero I	0.48	[0.45; 0.51]
Omitting Arabi A	0.48	[0.45; 0.51]
Omitting NHANES(2017...2018)	0.48	[0.45; 0.51]
Omitting Robinson PJ	0.48	[0.45; 0.51]
Omitting Yousef S	0.48	[0.45; 0.51]
Omitting Misra P	0.48	[0.45; 0.51]
Omitting Rahman A	0.48	[0.45; 0.51]
Omitting Daly RM	0.48	[0.45; 0.51]
Omitting Sokolovic S	0.48	[0.45; 0.51]
Omitting Feng X	0.48	[0.45; 0.51]
Omitting Dalgård C	0.48	[0.45; 0.51]
Omitting Lima−Costa MF	0.48	[0.45; 0.51]
Omitting Tønnesen R	0.48	[0.45; 0.51]
Omitting Drali O	0.48	[0.45; 0.51]
Omitting Saki F	0.48	[0.45; 0.51]
Omitting Hoge A	0.48	[0.45; 0.51]
Omitting Al−Daghri NM	0.48	[0.45; 0.51]
Omitting Rahmadhani R	0.48	[0.45; 0.51]
Omitting Orwoll E	0.48	[0.45; 0.51]
Omitting Ganmaa D	0.48	[0.45; 0.51]
Omitting NHANES(2017...2018)	0.48	[0.45; 0.51]
Omitting Isa H	0.48	[0.45; 0.51]
Omitting Carrillo−Vega MF	0.48	[0.45; 0.51]
Omitting Li S	0.48	[0.45; 0.51]
Omitting Sokolovic S	0.48	[0.45; 0.51]
Omitting González−Gross M	0.48	[0.45; 0.51]
Omitting Raposo L	0.48	[0.45; 0.51]
Omitting Ardawi MS	0.48	[0.45; 0.51]
Omitting Golbahar J	0.48	[0.45; 0.51]
Omitting Nimitphong H	0.48	[0.45; 0.51]
Omitting Solis−Urra P	0.48	[0.45; 0.51]
Omitting Arnljots R	0.48	[0.45; 0.51]
Omitting Cashman KD	0.48	[0.45; 0.51]
Omitting Riverin B	0.48	[0.45; 0.51]
Omitting Herrick KA	0.48	[0.45; 0.51]
Omitting Zhu Z	0.48	[0.45; 0.51]
Omitting NHANES(2015...2016)	0.48	[0.45; 0.51]
Omitting Marzban M	0.48	[0.45; 0.51]
Omitting Nichols EK	0.48	[0.45; 0.51]
Omitting Hovsepian S	0.48	[0.45; 0.51]
Omitting Oberg J	0.48	[0.45; 0.51]
Omitting Mehboobali N	0.48	[0.45; 0.51]
Omitting Sherief LM	0.48	[0.45; 0.51]
Omitting Daly RM	0.48	[0.45; 0.51]
Omitting Moy FM	0.48	[0.45; 0.51]
Omitting Mansbach JM	0.48	[0.45; 0.51]
Omitting Cougnard−Grégoire A	0.48	[0.45; 0.51]
Omitting Zhu Z	0.48	[0.45; 0.51]
Omitting Flores ME	0.48	[0.45; 0.51]
Omitting Chlebna−Sokó.. D	0.48	[0.45; 0.51]
Omitting Kapil U	0.48	[0.45; 0.51]
Omitting Al−Daghri NM	0.48	[0.45; 0.51]
Omitting Nikooyeh B	0.48	[0.45; 0.51]
Omitting NHANES(2017...2018)	0.48	[0.45; 0.51]
Omitting Solis−Urra P	0.48	[0.45; 0.51]
Omitting Cairncross CT	0.48	[0.45; 0.51]
Omitting Herrick KA	0.48	[0.45; 0.51]
Omitting Hutchings N	0.48	[0.45; 0.51]
Omitting Capuano R	0.48	[0.45; 0.51]
Omitting Chung IH	0.48	[0.45; 0.51]
Omitting Al−Daghri NM	0.48	[0.45; 0.51]
Omitting Daly RM	0.48	[0.45; 0.51]
Omitting Alkerwi A	0.48	[0.45; 0.51]
Omitting NHANES(2015...2016)	0.48	[0.45; 0.51]
Omitting Karonova T	0.48	[0.45; 0.51]
Omitting Daly RM	0.48	[0.45; 0.51]
Omitting Lucas JA	0.48	[0.45; 0.51]
Omitting Zhang FF	0.48	[0.45; 0.51]
Omitting Shivane VK	0.48	[0.45; 0.51]
Omitting Li L	0.48	[0.45; 0.51]
Omitting Jayashri R	0.48	[0.45; 0.51]
Omitting Kaykhaei MA	0.48	[0.45; 0.51]
Omitting Shchubelka K	0.48	[0.45; 0.51]
Omitting Herrick KA	0.48	[0.45; 0.51]
Omitting Seo JA	0.48	[0.45; 0.51]
Omitting Ikonen H	0.48	[0.45; 0.51]
Omitting Öztürk ZA	0.48	[0.45; 0.51]
Omitting Penrose K	0.48	[0.45; 0.51]
Omitting Ardawi MS	0.48	[0.45; 0.51]
Omitting Ceccarelli M	0.48	[0.45; 0.51]
Omitting Gromova O	0.48	[0.45; 0.51]
Omitting Fang F	0.48	[0.45; 0.51]
Omitting Hirani V	0.48	[0.45; 0.51]
Omitting Daly RM	0.48	[0.45; 0.51]
Omitting Santos A	0.48	[0.45; 0.51]
Omitting Jorde R	0.48	[0.45; 0.51]
Omitting Petrenya N	0.48	[0.45; 0.51]
Omitting Aleteng Q	0.48	[0.45; 0.51]
Omitting Herrick KA	0.48	[0.45; 0.51]
Omitting Al−Taiar A	0.48	[0.45; 0.51]
Omitting Al−Saleh Y	0.48	[0.45; 0.51]
Omitting Sulimani RA	0.48	[0.45; 0.51]
Omitting Rabenberg M	0.48	[0.45; 0.51]
Omitting Yu S	0.48	[0.45; 0.51]
Omitting Manios Y	0.48	[0.45; 0.51]
Omitting Voortman T	0.48	[0.45; 0.51]
Omitting Hansen L	0.48	[0.45; 0.51]
Omitting Nikooyeh B	0.48	[0.45; 0.51]
Omitting Beer RJ	0.48	[0.45; 0.51]
Omitting Rabenberg M	0.48	[0.45; 0.51]
Omitting Jayatissa R	0.48	[0.45; 0.51]
Omitting Borissova AM	0.48	[0.45; 0.51]
Omitting Nielsen NO	0.48	[0.45; 0.51]
Omitting Zhao Y	0.48	[0.45; 0.51]
Omitting Yang K	0.48	[0.45; 0.51]
Omitting AlQuaiz AM	0.48	[0.45; 0.51]
Omitting Metwally ASM	0.48	[0.45; 0.51]
Omitting Rabenberg M	0.48	[0.45; 0.51]
Omitting Beer RJ	0.48	[0.45; 0.51]
Omitting Chirita−Emandi A	0.48	[0.45; 0.51]
Omitting Zgaga L	0.48	[0.45; 0.51]
Omitting Patel JV	0.48	[0.45; 0.51]
Omitting Kim YS	0.48	[0.45; 0.51]
Omitting Al Shaikh AM	0.48	[0.45; 0.51]
Omitting Byun EJ	0.48	[0.45; 0.51]
Omitting Beer RJ	0.48	[0.45; 0.51]
Omitting Al−Daghri NM	0.48	[0.45; 0.51]
Omitting Schramm S	0.48	[0.45; 0.51]
Omitting Tolppanen AM	0.48	[0.45; 0.51]
Omitting Duarte C	0.48	[0.45; 0.51]
Omitting Laird E	0.48	[0.45; 0.51]
Omitting Leung RY	0.48	[0.45; 0.51]
Omitting Chen J	0.48	[0.45; 0.51]
Omitting Al−Daghri NM	0.48	[0.45; 0.51]
Omitting Joukar F	0.48	[0.45; 0.51]
Omitting Liu X	0.48	[0.45; 0.51]
Omitting Al−Daghri NM	0.48	[0.45; 0.51]
Omitting Liu X	0.48	[0.45; 0.51]
Omitting El−Khateeb M	0.48	[0.45; 0.51]
Omitting Saliba W	0.48	[0.45; 0.51]
Omitting Ward M	0.48	[0.45; 0.51]
Omitting Thuesen B	0.48	[0.45; 0.51]
Omitting Yousef S	0.48	[0.45; 0.51]
Omitting Al Shaikh A	0.48	[0.45; 0.51]
Omitting Aspell N	0.48	[0.45; 0.51]
Omitting Gökta O	0.48	[0.45; 0.51]
Omitting Beer RJ	0.48	[0.45; 0.51]
Omitting Perna L	0.48	[0.45; 0.51]
Omitting Li H	0.48	[0.45; 0.51]
Omitting Li H	0.48	[0.45; 0.51]
Omitting Sarafin K	0.48	[0.45; 0.51]
Omitting Liu X	0.48	[0.45; 0.51]
Omitting Han B	0.48	[0.45; 0.51]
Omitting Hintzpeter B	0.48	[0.45; 0.51]
Omitting Song HR	0.48	[0.45; 0.51]
Omitting Jiang W	0.48	[0.45; 0.51]
Omitting Zhen D	0.48	[0.45; 0.51]
Omitting Griffin TP	0.48	[0.45; 0.51]
Omitting Al Zarooni AAR	0.48	[0.45; 0.51]
Omitting Chung JY	0.48	[0.45; 0.51]
Omitting Eloi M	0.48	[0.45; 0.51]
Omitting Lee J	0.48	[0.45; 0.51]
Omitting Kim SY	0.48	[0.45; 0.51]
Omitting Saliba W	0.48	[0.45; 0.51]
Omitting Lin LY	0.48	[0.45; 0.51]
**Random effects model**	**0.48**	**[0.45; 0.51]**

−0.4 −0.2 0 0.2 0.4

Supplementary figure 40 The sensitivity analyses for prevalence of serum 25-hydroxyvitamin D levels less than 50 nmol/l

Appendix 13: Sensitivity analyses

Study	Proportion	95%−CI
Omitting Oliveri B	0.76	[0.74; 0.79]
Omitting Harinarayan CV	0.77	[0.74; 0.79]
Omitting Hintzpeter B	0.76	[0.74; 0.79]
Omitting Mansbach JM	0.76	[0.74; 0.79]
Omitting Mansbach JM	0.76	[0.74; 0.79]
Omitting Orwoll E	0.76	[0.74; 0.79]
Omitting Gilbert−Diamond D	0.76	[0.74; 0.79]
Omitting Hekimsoy Z	0.76	[0.74; 0.79]
Omitting Ardawi MS	0.76	[0.74; 0.79]
Omitting Batieha A	0.77	[0.74; 0.79]
Omitting Chailurkit LO	0.77	[0.74; 0.79]
Omitting González−Molero I	0.76	[0.74; 0.79]
Omitting Greene−Finestone LS	0.76	[0.74; 0.79]
Omitting Greene−Finestone LS	0.76	[0.74; 0.79]
Omitting Greene−Finestone LS	0.76	[0.74; 0.79]
Omitting Ho−Pham LT	0.77	[0.74; 0.79]
Omitting Hovsepian S	0.76	[0.74; 0.79]
Omitting Kaykhaei MA	0.76	[0.74; 0.79]
Omitting Le Goaziou MF	0.76	[0.74; 0.79]
Omitting Maguire JL	0.76	[0.74; 0.79]
Omitting Mallah EM	0.76	[0.74; 0.79]
Omitting Miljkovic I	0.77	[0.74; 0.79]
Omitting Naeem Z	0.76	[0.74; 0.79]
Omitting Ramakrishnan S	0.76	[0.74; 0.79]
Omitting Shivane VK	0.76	[0.74; 0.79]
Omitting Ardawi MS	0.76	[0.74; 0.79]
Omitting Daly RM	0.76	[0.74; 0.79]
Omitting Daly RM	0.76	[0.74; 0.79]
Omitting Daly RM	0.76	[0.74; 0.79]
Omitting Daly RM	0.76	[0.74; 0.79]
Omitting Daly RM	0.76	[0.74; 0.79]
Omitting Daly RM	0.76	[0.74; 0.79]
Omitting González−Gross M	0.76	[0.74; 0.79]
Omitting Hirani V	0.76	[0.74; 0.79]
Omitting Khan AH	0.76	[0.74; 0.79]
Omitting Mitchell DM	0.76	[0.74; 0.79]
Omitting Penrose K	0.76	[0.74; 0.79]
Omitting Saliba W	0.76	[0.74; 0.79]
Omitting Saliba W	0.76	[0.74; 0.79]
Omitting Santos BR	0.76	[0.74; 0.79]
Omitting Sheikh A	0.76	[0.74; 0.79]
Omitting Sioen I	0.76	[0.74; 0.79]
Omitting Tolppanen AM	0.76	[0.74; 0.79]
Omitting Vallianou N	0.76	[0.74; 0.79]
Omitting Zhu Z	0.76	[0.74; 0.79]
Omitting Zhu Z	0.76	[0.74; 0.79]
Omitting Zhu Z	0.76	[0.74; 0.79]
Omitting Abiaka C	0.76	[0.74; 0.79]
Omitting Aucoin M	0.76	[0.74; 0.79]
Omitting Aucoin M	0.76	[0.74; 0.79]
Omitting Cabral MA	0.76	[0.74; 0.79]
Omitting Chung JY	0.76	[0.74; 0.79]
Omitting El Hayek J	0.76	[0.74; 0.79]
Omitting Ginter JK	0.77	[0.74; 0.79]
Omitting Hirani V	0.76	[0.74; 0.79]
Omitting Hirani V	0.76	[0.74; 0.79]
Omitting Hirani V	0.76	[0.74; 0.79]
Omitting Hirani V	0.76	[0.74; 0.79]
Omitting Janssen HC	0.76	[0.74; 0.79]
Omitting Klenk J	0.77	[0.74; 0.79]
Omitting Kouda K	0.76	[0.74; 0.79]
Omitting Matheï C	0.76	[0.74; 0.79]
Omitting Riverin B	0.76	[0.74; 0.79]
Omitting Robinson PJ	0.76	[0.74; 0.79]
Omitting Robinson PJ	0.76	[0.74; 0.79]
Omitting Chung IH	0.76	[0.74; 0.79]
Omitting Cinar N	0.76	[0.74; 0.79]
Omitting Djennane M	0.76	[0.74; 0.79]
Omitting Ganmaa D	0.76	[0.73; 0.79]
Omitting Gill TK	0.76	[0.74; 0.79]
Omitting Gill TK	0.76	[0.74; 0.79]
Omitting Gill TK	0.76	[0.74; 0.79]
Omitting Gill TK	0.76	[0.74; 0.79]
Omitting Gill TK	0.76	[0.74; 0.79]
Omitting Gill TK	0.76	[0.74; 0.79]
Omitting Karagüzel G	0.77	[0.74; 0.79]
Omitting Madsen KH	0.76	[0.74; 0.79]
Omitting Madsen KH	0.76	[0.74; 0.79]
Omitting Oberg J	0.76	[0.74; 0.79]
Omitting Rafraf M	0.76	[0.74; 0.79]
Omitting Riverin B	0.76	[0.74; 0.79]
Omitting Samefors M	0.76	[0.74; 0.79]
Omitting Song HR	0.76	[0.74; 0.79]
Omitting Vierucci F	0.76	[0.74; 0.79]
Omitting Alkerwi A	0.76	[0.74; 0.79]
Omitting Brinkmann K	0.76	[0.74; 0.79]
Omitting Chirita−Emandi A	0.76	[0.74; 0.79]
Omitting Hazell TJ	0.77	[0.74; 0.79]
Omitting Hoge A	0.76	[0.74; 0.79]
Omitting Mehboobali N	0.76	[0.74; 0.79]
Omitting Orces CH	0.76	[0.74; 0.79]
Omitting Orces CH	0.76	[0.74; 0.79]
Omitting Orces CH	0.76	[0.74; 0.79]
Omitting Rabenberg M	0.76	[0.74; 0.79]
Omitting Rabenberg M	0.76	[0.74; 0.79]
Omitting Rabenberg M	0.76	[0.74; 0.79]
Omitting Voortman T	0.76	[0.74; 0.79]
Omitting Eloi M	0.76	[0.74; 0.79]
Omitting Feng X	0.76	[0.74; 0.79]
Omitting Granlund L	0.76	[0.74; 0.79]
Omitting Jolliffe DA	0.76	[0.74; 0.79]
Omitting Karonova T	0.76	[0.74; 0.79]
Omitting Man PW	0.76	[0.74; 0.79]
Omitting Smith G	0.77	[0.74; 0.79]
Omitting Smith G	0.77	[0.74; 0.79]
Omitting Smith G	0.77	[0.74; 0.79]
Omitting Souberbielle JC	0.76	[0.74; 0.79]
Omitting Aleteng Q	0.76	[0.74; 0.79]
Omitting Arnljots R	0.76	[0.74; 0.79]
Omitting Cairncross CT	0.76	[0.74; 0.79]
Omitting Jääskeläinen T	0.76	[0.74; 0.79]
Omitting Leung RY	0.76	[0.74; 0.79]
Omitting Maldonado G	0.76	[0.74; 0.79]
Omitting Manios Y	0.76	[0.74; 0.79]
Omitting Naqvi A	0.76	[0.74; 0.79]
Omitting Öztürk ZA	0.76	[0.74; 0.79]
Omitting Saki F	0.76	[0.74; 0.79]
Omitting Schramm S	0.76	[0.74; 0.79]
Omitting Science M	0.76	[0.74; 0.79]
Omitting Sokolovic S	0.76	[0.74; 0.79]
Omitting Sokolovic S	0.76	[0.74; 0.79]
Omitting Sokolovic S	0.76	[0.74; 0.79]
Omitting Srimani S	0.76	[0.74; 0.79]
Omitting Al Hayek S	0.76	[0.74; 0.79]
Omitting AlQuaiz AM	0.76	[0.74; 0.79]
Omitting Al−Taiar A	0.76	[0.74; 0.79]
Omitting Bettencourt A	0.76	[0.74; 0.79]
Omitting Hansen L	0.76	[0.74; 0.79]
Omitting Hansen L	0.76	[0.74; 0.79]
Omitting Kagotho E	0.76	[0.74; 0.79]
Omitting Kapil U	0.76	[0.74; 0.79]
Omitting Kapil U	0.76	[0.74; 0.79]
Omitting Karin Z	0.76	[0.74; 0.79]
Omitting Kunz C	0.76	[0.74; 0.79]
Omitting Kunz C	0.76	[0.74; 0.79]
Omitting Kunz C	0.76	[0.74; 0.79]
Omitting Kunz C	0.76	[0.74; 0.79]
Omitting Liu X	0.76	[0.74; 0.79]
Omitting Liu X	0.76	[0.74; 0.79]
Omitting Liu X	0.76	[0.74; 0.79]
Omitting Nadeem S	0.76	[0.74; 0.79]
Omitting Ní Chaoimh C	0.76	[0.74; 0.79]
Omitting Pan T	0.76	[0.74; 0.79]
Omitting Sherchand O	0.76	[0.74; 0.79]
Omitting Tangoh DA	0.77	[0.74; 0.79]
Omitting Zhu W	0.77	[0.74; 0.79]
Omitting Al Zarooni AAR	0.76	[0.74; 0.79]
Omitting El−Khateeb M	0.76	[0.74; 0.79]
Omitting Ten Haaf DSM	0.77	[0.74; 0.79]
Omitting White Z	0.76	[0.74; 0.79]
Omitting Ceccarelli M	0.76	[0.74; 0.79]
Omitting de Oliveira CL	0.76	[0.74; 0.79]
Omitting Duarte C	0.76	[0.74; 0.79]
Omitting Gromova O	0.76	[0.74; 0.79]
Omitting Hribar M	0.76	[0.74; 0.79]
Omitting Hribar M	0.76	[0.74; 0.79]
Omitting Isa H	0.76	[0.74; 0.79]
Omitting Jayashri R	0.76	[0.74; 0.79]
Omitting Jiang W	0.76	[0.74; 0.79]
Omitting Li L	0.76	[0.74; 0.79]
Omitting Lima−Costa MF	0.76	[0.74; 0.79]
Omitting Mutua AM	0.77	[0.74; 0.79]
Omitting Petrenya N	0.76	[0.74; 0.79]
Omitting Rahman A	0.76	[0.74; 0.79]
Omitting Yang K	0.76	[0.74; 0.79]
Omitting Arabi A	0.76	[0.74; 0.79]
Omitting Black LJ	0.76	[0.74; 0.79]
Omitting Black LJ	0.76	[0.74; 0.79]
Omitting Black LJ	0.76	[0.74; 0.79]
Omitting Black LJ	0.76	[0.74; 0.79]
Omitting Black LJ	0.76	[0.74; 0.79]
Omitting Capuano R	0.76	[0.74; 0.79]
Omitting Contreras−Manzano A	0.76	[0.74; 0.79]
Omitting Contreras−Manzano A	0.76	[0.74; 0.79]
Omitting Contreras−Manzano A	0.76	[0.74; 0.79]
Omitting Drali O	0.76	[0.74; 0.79]
Omitting Feketea GM	0.76	[0.74; 0.79]
Omitting Flores ME	0.76	[0.74; 0.79]
Omitting Flores ME	0.76	[0.74; 0.79]
Omitting Horton−French K	0.76	[0.74; 0.79]
Omitting Horton−French K	0.76	[0.74; 0.79]
Omitting Ikonen H	0.76	[0.74; 0.79]
Omitting Koyama S	0.76	[0.74; 0.79]
Omitting Mogire RM	0.76	[0.74; 0.79]
Omitting Sherief LM	0.76	[0.74; 0.79]
Omitting Yousef S	0.76	[0.74; 0.79]
Omitting Yousef S	0.76	[0.74; 0.79]
Omitting Yousef S	0.76	[0.74; 0.79]
Omitting Yousef S	0.76	[0.74; 0.79]
Omitting Yousef S	0.76	[0.74; 0.79]
Omitting Zhao Y	0.76	[0.74; 0.79]
Omitting Abdulrahman MA	0.76	[0.74; 0.79]
Omitting Abdulrahman MA	0.76	[0.74; 0.79]
Omitting Abdulrahman MA	0.76	[0.74; 0.79]
Omitting Lin L	0.77	[0.74; 0.79]
Omitting Lin L	0.77	[0.74; 0.79]
Omitting Lin L	0.77	[0.74; 0.79]
Omitting NHANES (2015...2016)	0.76	[0.74; 0.79]
Omitting NHANES(2015...2016)	0.77	[0.74; 0.79]
Omitting NHANES(2015...2016)	0.76	[0.74; 0.79]
Omitting NHANES(2015...2016)	0.76	[0.74; 0.79]
Omitting NHANES(2017...2018)	0.77	[0.74; 0.79]
Omitting NHANES(2017...2018)	0.76	[0.74; 0.79]
Omitting NHANES(2017...2018)	0.76	[0.74; 0.79]
Omitting NHANES(2017...2018)	0.76	[0.74; 0.79]
**Random effects model**	**0.76**	**[0.74; 0.79]**

−0.5 0 0.5

Supplementary figure 41 The sensitivity analyses for prevalence of serum 25-hydroxyvitamin D levels less than 75 nmol/l

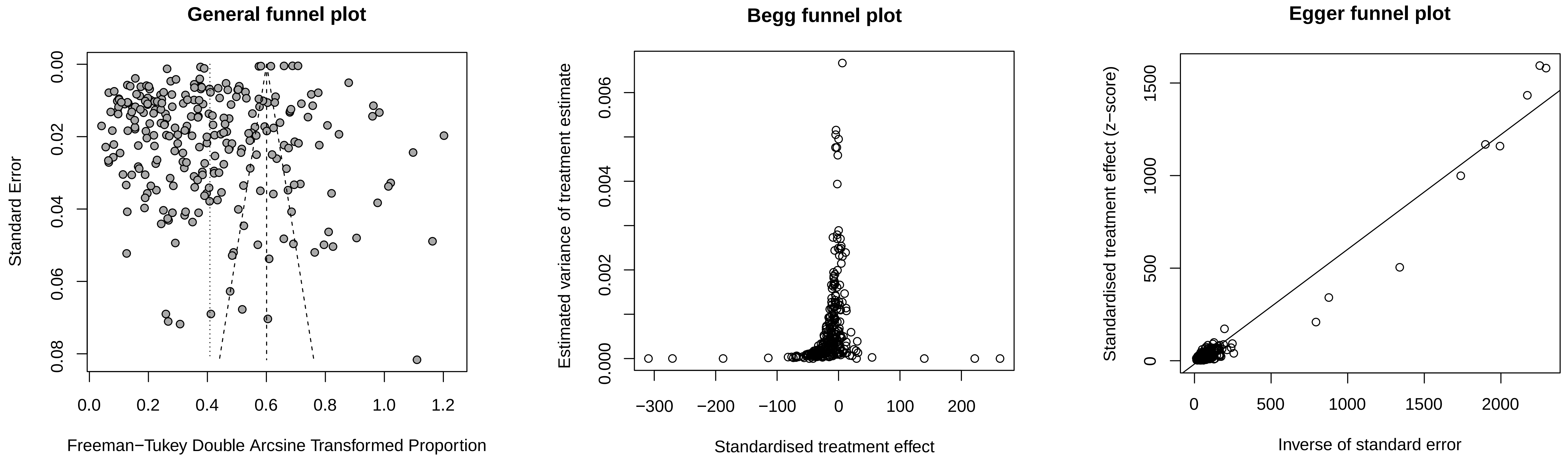

Supplementary figure 42 The publication bias analyses for studies on prevalence of serum 25-hydroxyvitamin D levels less than 30 nmol/l

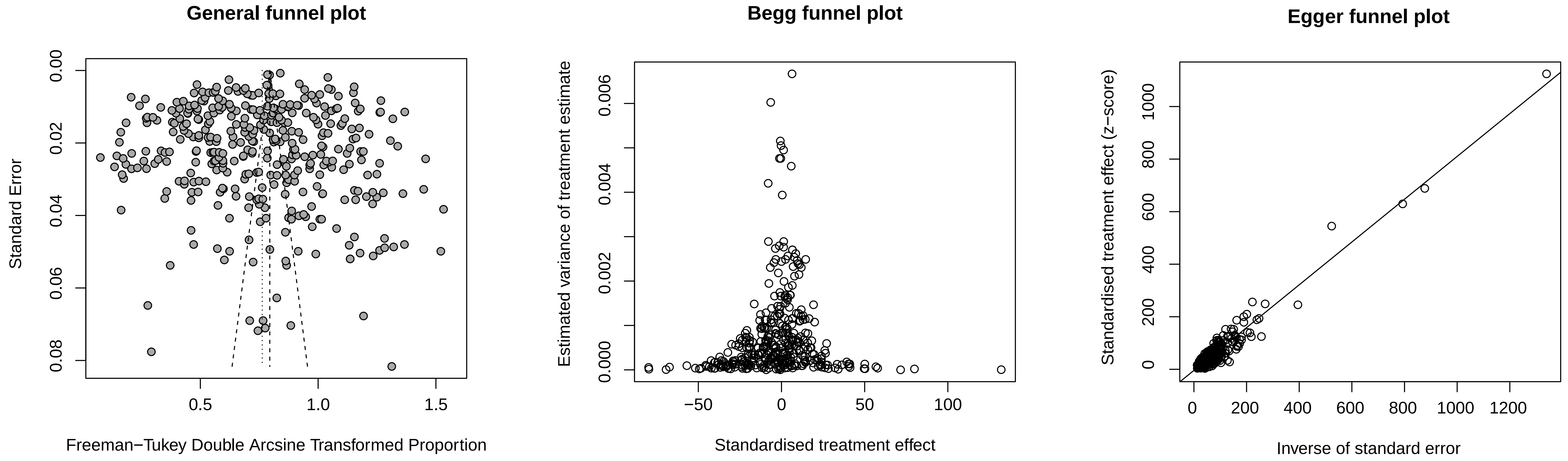

Supplementary figure 43 The publication bias analyses for studies on prevalence of serum 25-hydroxyvitamin D levels less than 50 nmol/l

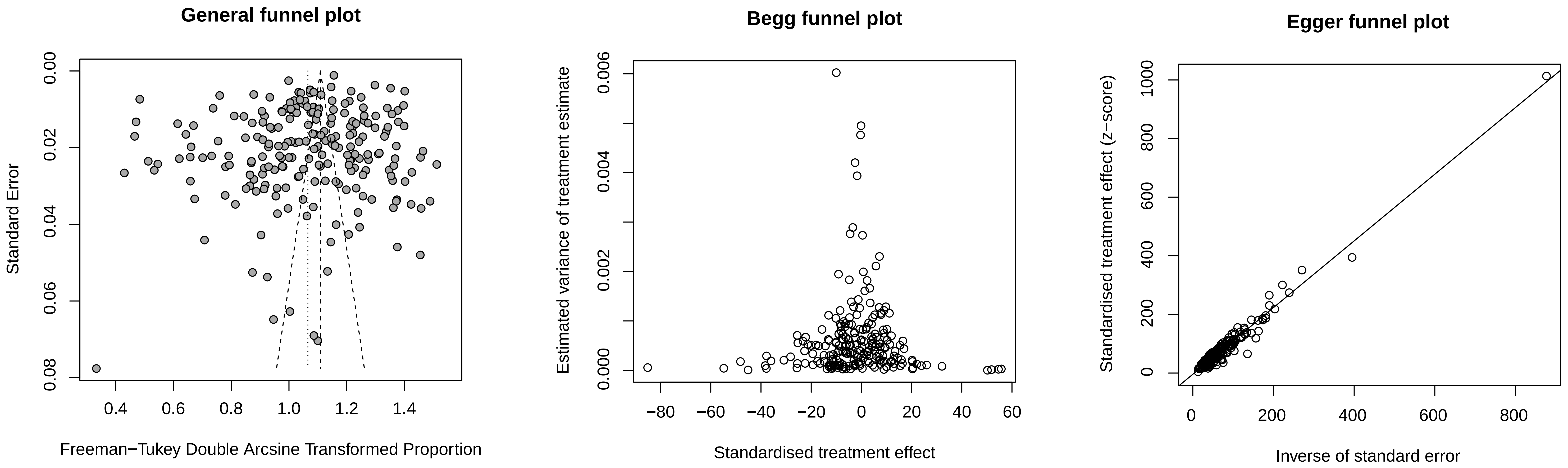

Supplementary figure 44 The publication bias analyses for studies on prevalence of serum 25-hydroxyvitamin D levels less than 75 nmol/l

Appendix 15: References

1. Abdulrahman MA, Alkass SY, Mohammed NI. Total and free vitamin D status among apparently healthy adults living in Duhok Governorate. *Sci Rep* 2022; **12**(1): 1778.
2. Abiaka C, Delghandi M, Kaur M, Al-Saleh M. Vitamin d status and anthropometric indices of an omani study population. *Sultan Qaboos Univ Med J* 2013; **13**(2): 224-31.
3. Abu-Samak MS, AbuRuz ME, Masa'Deh R, Khuzai R, Jarrah S. Correlation of selected stress associated factors with vitamin D deficiency in Jordanian men and women. *International journal of general medicine* 2019; **12**: 225-33.
4. Al Hayek S, Matar Bou Mosleh J, Ghadieh R, El Hayek Fares J. Vitamin D status and body composition: a cross-sectional study among employees at a private university in Lebanon. *BMC Nutr* 2018; **4**: 31.
5. Al Shaikh A, Farahat F, Abaalkhail B, et al. Prevalence of Obesity and Overweight among School-Aged Children in Saudi Arabia and Its Association with Vitamin D Status. *Acta Biomed* 2020; **91**(4): e2020133.
6. Al Shaikh AM, Abaalkhail B, Soliman A, et al. Prevalence of Vitamin D Deficiency and Calcium Homeostasis in Saudi Children. *J Clin Res Pediatr Endocrinol* 2016; **8**(4): 461-7.
7. Al Zarooni AAR, Al Marzouqi FI, Al Darmaki SH, Prinsloo EAM, Nagelkerke N. Prevalence of vitamin D deficiency and associated comorbidities among Abu Dhabi Emirates population. *BMC Res Notes* 2019; **12**(1): 503.
8. Al-Daghri NM, Al-Saleh Y, Aljohani N, et al. Vitamin D Deficiency and Cardiometabolic Risks: A Juxtaposition of Arab Adolescents and Adults. *PLoS One* 2015; **10**(7): e0131315.
9. Al-Daghri NM, Al-Saleh Y, Khan N, et al. Sun exposure, skin color and vitamin D status in Arab children and adults. *J Steroid Biochem Mol Biol* 2016; **164**: 235-8.
10. Al-Daghri NM, Hussain SD, Ansari MGA, et al. Decreasing prevalence of vitamin D deficiency in the central region of Saudi Arabia (2008-2017). *J Steroid Biochem Mol Biol* 2021; **212**: 105920.
11. Aleteng Q, Zhao L, Lin H, et al. Optimal Vitamin D Status in a Middle-Aged and Elderly Population Residing in Shanghai, China. *Med Sci Monit* 2017; **23**: 6001-11.
12. AlFaris NA, AlKehayez NM, AlMushawah FI, AlNaeem AN, AlAmri ND, AlMudawah ES. Vitamin D Deficiency and Associated Risk Factors in Women from Riyadh, Saudi Arabia. *Sci Rep* 2019; **9**(1): 20371.
13. Alkerwi A, Sauvageot N, Gilson G, Stranges S. Prevalence and Correlates of Vitamin D Deficiency and Insufficiency in Luxembourg Adults: Evidence from the Observation of Cardiovascular Risk Factors (ORISCAV-LUX) Study. *Nutrients* 2015; **7**(8): 6780-96.
14. Al-Kindi MK. Vitamin D Status in Healthy Omani Women of Childbearing Age: Study of female staff at the Royal Hospital, Muscat, Oman. *Sultan Qaboos Univ Med J* 2011; **11**(1): 56-61.

15. Alloubani A, Akhu-Zaheya L, Samara R, Abdulhafiz I, Saleh A, Altowijri A. Relationship between Vitamin D Deficiency, Diabetes, and Obesity. *Diabetes Metab Syndr* 2019; **13**(2): 1457-61.
16. AlQuaiz AM, Kazi A, Fouda M, Alyousefi N. Age and gender differences in the prevalence and correlates of vitamin D deficiency. *Arch Osteoporos* 2018; **13**(1): 49.
17. Al-Saleh Y, Al-Daghri NM, Khan N, et al. Vitamin D status in Saudi school children based on knowledge. *BMC Pediatr* 2015; **15**: 53.
18. Al-Taiar A, Rahman A, Al-Sabah R, Shaban L, Al-Harbi A. Vitamin D status among adolescents in Kuwait: a cross-sectional study. *BMJ Open* 2018; **8**(7): e021401.
19. Alyahya K, Lee WT, Al-Mazidi Z, Morgan J, Lanham-New S. Risk factors of low vitamin D status in adolescent females in Kuwait: implications for high peak bone mass attainment. *Arch Osteoporos* 2014; **9**: 178.
20. Alyahya KO. Poor dietary consumption and limited sun exposure are risk factors for vitamin D deficiency in premenopausal Kuwaiti women: A cross-sectional study. *Qatar Med J* 2020; **2020**(1): 15.
21. Andersen R, Molgaard C, Skovgaard LT, et al. Teenage girls and elderly women living in northern Europe have low winter vitamin D status. *Eur J Clin Nutr* 2005; **59**(4): 533-41.
22. Andersen R, Molgaard C, Skovgaard LT, et al. Pakistani immigrant children and adults in Denmark have severely low vitamin D status. *Eur J Clin Nutr* 2008; **62**(5): 625-34.
23. Andersen R, Brot C, Jakobsen J, et al. Seasonal changes in vitamin D status among Danish adolescent girls and elderly women: the influence of sun exposure and vitamin D intake. *Eur J Clin Nutr* 2013; **67**(3): 270-4.
24. Andersen S, Jakobsen A, Laurberg P. Vitamin D status in North Greenland is influenced by diet and season: indicators of dermal 25-hydroxy vitamin D production north of the Arctic Circle. *Br J Nutr* 2013; **110**(1): 50-7.
25. Arabi A, Chamoun N, Nasrallah MP, Tamim HM. Vitamin D Deficiency in Lebanese Adults: Prevalence and Predictors from a Cross-Sectional Community-Based Study. *Int J Endocrinol* 2021; **2021**: 3170129.
26. Ardawi MS, Qari MH, Rouzi AA, Maimani AA, Raddadi RM. Vitamin D status in relation to obesity, bone mineral density, bone turnover markers and vitamin D receptor genotypes in healthy Saudi pre- and postmenopausal women. *Osteoporos Int* 2011; **22**(2): 463-75.
27. Ardawi MS, Sibiany AM, Bakhsh TM, Qari MH, Maimani AA. High prevalence of vitamin D deficiency among healthy Saudi Arabian men: relationship to bone mineral density, parathyroid hormone, bone turnover markers, and lifestyle factors. *Osteoporos Int* 2012; **23**(2): 675-86.
28. Arnljots R, Thorn J, Elm M, Moore M, Sundvall PD. Vitamin D deficiency was common among nursing home residents and associated with dementia: a cross sectional study of 545 Swedish nursing home residents. *BMC Geriatr* 2017; **17**(1): 229.

29. Arya V, Bhambri R, Godbole MM, Mithal A. Vitamin D status and its relationship with bone mineral density in healthy Asian Indians. *Osteoporos Int* 2004; **15**(1): 56-61.
30. Asakura K, Etoh N, Imamura H, et al. Vitamin D Status in Japanese Adults: Relationship of Serum 25-Hydroxyvitamin D with Simultaneously Measured Dietary Vitamin D Intake and Ultraviolet Ray Exposure. *Nutrients* 2020; **12**(3).
31. Aspell N, Laird E, Healy M, Shannon T, Lawlor B, O'Sullivan M. The Prevalence and Determinants of Vitamin D Status in Community-Dwelling Older Adults: Results from the English Longitudinal Study of Ageing (ELSA). *Nutrients* 2019; **11**(6).
32. Aucoin M, Weaver R, Thomas R, Jones L. Vitamin D status of refugees arriving in Canada: findings from the Calgary Refugee Health Program. *Can Fam Physician* 2013; **59**(4): e188-94.
33. Bachhel R, Singh NR, Sidhu JS. Prevalence of vitamin D deficiency in north-west Punjab population: A cross-sectional study. *Int J Appl Basic Med Res* 2015; **5**(1): 7-11.
34. Bater J, Bromage S, Jambal T, et al. Prevalence and Determinants of Vitamin D Deficiency in 9595 Mongolian Schoolchildren: A Cross-Sectional Study. *Nutrients* 2021; **13**(11).
35. Batieha A, Khader Y, Jaddou H, et al. Vitamin D status in Jordan: dress style and gender discrepancies. *Ann Nutr Metab* 2011; **58**(1): 10-8.
36. Beer RJ, Herran OF, Villamor E. Prevalence and correlates of vitamin D deficiency in a tropical setting: results from a nationally representative survey. *Am J Clin Nutr* 2020; **112**(4): 1088-98.
37. Bener A, Al-Ali M, Hoffmann GF. High prevalence of vitamin D deficiency in young children in a highly sunny humid country: a global health problem. *Minerva Pediatr* 2009; **61**(1): 15-22.
38. Benjeddou K, Qandoussi L, Mekkaoui B, et al. Effect of multiple micronutrient fortified milk consumption on vitamin D status among school-aged children in rural region of Morocco. *Appl Physiol Nutr Metab* 2019; **44**(5): 461-7.
39. Bettencourt A, Boleixa D, Reis J, et al. Serum 25-hydroxyvitamin D levels in a healthy population from the North of Portugal. *J Steroid Biochem Mol Biol* 2018; **175**: 97-101.
40. Bezrati I, Ben Fradj MK, Ouerghi N, Feki M, Chaouachi A, Kaabachi N. Vitamin D inadequacy is widespread in Tunisian active boys and is related to diet but not to adiposity or insulin resistance. *Libyan J Med* 2016; **11**: 31258.
41. Bhatt SP, Misra A, Sharma M, et al. Vitamin D insufficiency is associated with abdominal obesity in urban Asian Indians without diabetes in North India. *Diabetes Technol Ther* 2014; **16**(6): 392-7.
42. Bhattoa HP, Nagy E, More C, et al. Prevalence and seasonal variation of hypovitaminosis D and its relationship to bone metabolism in healthy Hungarian men over 50 years of age: the HunMen Study. *Osteoporos Int* 2013; **24**(1): 179-86.
43. Bi X, Tey SL, Leong C, Quek R, Henry CJ. Prevalence of Vitamin D Deficiency

in Singapore: Its Implications to Cardiovascular Risk Factors. *PLoS One* 2016; **11**(1): e0147616.

44. Bjarnadottir A, Kristjansdottir AG, Hrafnkelsson H, Johannsson E, Magnusson KT, Thorsdottir I. Insufficient autumn vitamin D intake and low vitamin D status in 7-year-old Icelandic children. *Public Health Nutr* 2015; **18**(2): 208-17.
45. Black LJ, Dunlop E, Lucas RM, Pearson G, Farrant B, Shepherd CCJ. Prevalence and predictors of vitamin D deficiency in a nationally representative sample of Australian Aboriginal and Torres Strait Islander adults. *Br J Nutr* 2021; **126**(1): 101-9.
46. Bodin J, Mihret A, Holm-Hansen C, et al. Vitamin D Deficiency is Associated with Increased Use of Antimicrobials among Preschool Girls in Ethiopia. *Nutrients* 2019; **11**(3).
47. Bolland MJ, Grey AB, Ames RW, et al. Determinants of vitamin D status in older men living in a subtropical climate. *Osteoporos Int* 2006; **17**(12): 1742-8.
48. Borissova AM, Shinkov A, Vlahov J, et al. Vitamin D status in Bulgaria--winter data. *Arch Osteoporos* 2013; **8**: 133.
49. Brinkmann K, Le Roy C, Iniguez G, Borzutzky A. [Severe vitamin D deficiency in children from Punta Arenas, Chile: Influence of nutritional status on the response to supplementation]. *Rev Chil Pediatr* 2015; **86**(3): 182-8.
50. Byun EJ, Heo J, Cho SH, Lee JD, Kim HS. Suboptimal vitamin D status in Korean adolescents: a nationwide study on its prevalence, risk factors including cotinine-verified smoking status and association with atopic dermatitis and asthma. *BMJ Open* 2017; **7**(7): e016409.
51. Cabral MA, Borges CN, Maia JM, Aires CA, Bandeira F. Prevalence of vitamin D deficiency during the summer and its relationship with sun exposure and skin phototype in elderly men living in the tropics. *Clin Interv Aging* 2013; **8**: 1347-51.
52. Cairncross CT, Stonehouse W, Conlon CA, et al. Predictors of vitamin D status in New Zealand preschool children. *Matern Child Nutr* 2017; **13**(3).
53. Capuano R, Marchese F, Sica R, et al. Epidemiologic Data of Vitamin D Deficiency and Its Implication in Cardio-Cerebrovascular Risk in a Southern Italian Population. *J Nutr Metab* 2021; **2021**: 5550222.
54. Carrillo-Vega MF, Garcia-Pena C, Gutierrez-Robledo LM, Perez-Zepeda MU. Vitamin D deficiency in older adults and its associated factors: a cross-sectional analysis of the Mexican Health and Aging Study. *Arch Osteoporos* 2017; **12**(1): 8.
55. Cashman KD, Muldowney S, McNulty B, et al. Vitamin D status of Irish adults: findings from the National Adult Nutrition Survey. *Br J Nutr* 2013; **109**(7): 1248-56.
56. Ceccarelli M, Chiappini E, Arancio R, et al. Vitamin D deficiency in a population of migrant children: an Italian retrospective cross-sectional multicentric study. *Eur J Public Health* 2020; **30**(3): 551-6.
57. Chailurkit LO, Aekplakorn W, Ongphiphadhanakul B. Regional variation and determinants of vitamin D status in sunshine-abundant Thailand. *BMC Public Health* 2011; **11**: 853.
58. Chao YS, Brunel L, Faris P, Veugelers PJ. Vitamin D status of Canadians employed

in northern latitudes. *Occup Med (Lond)* 2013; **63**(7): 485-93.
59. Chao YS, Ekwaru JP, Ohinmaa A, Griener G, Veugelers PJ. Vitamin D and health-related quality of life in a community sample of older Canadians. *Qual Life Res* 2014; **23**(9): 2569-75.
60. Chen J, Yun C, He Y, Piao J, Yang L, Yang X. Vitamin D status among the elderly Chinese population: a cross-sectional analysis of the 2010-2013 China national nutrition and health survey (CNNHS). *Nutr J* 2017; **16**(1): 3.
61. Chin KY, Ima-Nirwana S, Ibrahim S, Mohamed IN, Wan Ngah WZ. Vitamin D status in Malaysian men and its associated factors. *Nutrients* 2014; **6**(12): 5419-33.
62. Chirita-Emandi A, Socolov D, Haivas C, Calapis A, Gheorghiu C, Puiu M. Vitamin D Status: A Different Story in the Very Young versus the Very Old Romanian Patients. *PLoS One* 2015; **10**(5): e0128010.
63. Chlebna-Sokol D, Konstantynowicz J, Abramowicz P, et al. Evidence of a significant vitamin D deficiency among 9-13-year-old Polish children: results of a multicentre study. *Eur J Nutr* 2019; **58**(5): 2029-36.
64. Choi HR, Lee SW, Yeom H, Jeon DH, Kim HC, Youm Y. Association between vitamin D status and asymmetric dimethylarginine (ADMA) concentration in the Korean elderly population. *Maturitas* 2017; **102**: 13-7.
65. Chung IH, Kim HJ, Chung S, Yoo EG. Vitamin D deficiency in Korean children: prevalence, risk factors, and the relationship with parathyroid hormone levels. *Ann Pediatr Endocrinol Metab* 2014; **19**(2): 86-90.
66. Chung JY, Hong SH. Vitamin D status and its association with cardiometabolic risk factors in Korean adults based on a 2008-2010 Korean National Health and Nutrition Examination Survey. *Nutr Res Pract* 2013; **7**(6): 495-502.
67. Cinar N, Harmanci A, Yildiz BO, Bayraktar M. Vitamin D status and seasonal changes in plasma concentrations of 25-hydroxyvitamin D in office workers in Ankara, Turkey. *Eur J Intern Med* 2014; **25**(2): 197-201.
68. Contreras-Manzano A, Mejia-Rodriguez F, Villalpando S, Rebollar R, Flores-Aldana M. Vitamin D status in Mexican women at reproductive age, Ensanut 2018-19. *Salud Publica Mex* 2021; **63**(3 May-Jun): 394-400.
69. Cougnard-Gregoire A, Merle BM, Korobelnik JF, et al. Vitamin D Deficiency in Community-Dwelling Elderly Is Not Associated with Age-Related Macular Degeneration. *J Nutr* 2015; **145**(8): 1865-72.
70. Courraud J, Quist JS, Kontopodi E, et al. Dietary habits, metabolic health and vitamin D status in Greenlandic children. *Public Health Nutr* 2020; **23**(5): 904-13.
71. Crowe FL, Jolly K, MacArthur C, et al. Trends in the incidence of testing for vitamin D deficiency in primary care in the UK: a retrospective analysis of The Health Improvement Network (THIN), 2005-2015. *BMJ Open* 2019; **9**(6): e028355.
72. Dalgard C, Petersen MS, Schmedes AV, Brandslund I, Weihe P, Grandjean P. High latitude and marine diet: vitamin D status in elderly Faroese. *Br J Nutr* 2010; **104**(6): 914-8.
73. Daly RM, Gagnon C, Lu ZX, et al. Prevalence of vitamin D deficiency and its

determinants in Australian adults aged 25 years and older: a national, population-based study. *Clin Endocrinol (Oxf)* 2012; **77**(1): 26-35.

74. de Oliveira CL, Cureau FV, Cople-Rodrigues CDS, et al. Prevalence and factors associated with hypovitaminosis D in adolescents from a sunny country: Findings from the ERICA survey. *J Steroid Biochem Mol Biol* 2020; **199**: 105609.
75. Djennane M, Lebbah S, Roux C, Djoudi H, Cavalier E, Souberbielle JC. Vitamin D status of schoolchildren in Northern Algeria, seasonal variations and determinants of vitamin D deficiency. *Osteoporos Int* 2014; **25**(5): 1493-502.
76. Drali O, Arab M, Lamdjadani N, Guechi Z, Berrah H. Vitamin D status in preschool children in Algeria. *Arch Pediatr* 2021; **28**(3): 215-21.
77. Duarte C, Carvalheiro H, Rodrigues AM, et al. Prevalence of vitamin D deficiency and its predictors in the Portuguese population: a nationwide population-based study. *Arch Osteoporos* 2020; **15**(1): 36.
78. El Hayek J, Egeland G, Weiler H. Vitamin D status of Inuit preschoolers reflects season and vitamin D intake. *J Nutr* 2010; **140**(10): 1839-45.
79. El Hayek J, Pham TT, Finch S, et al. Vitamin D status in Montreal preschoolers is satisfactory despite low vitamin D intake. *J Nutr* 2013; **143**(2): 154-60.
80. El-Khateeb M, Khader Y, Batieha A, et al. Vitamin D deficiency and associated factors in Jordan. *SAGE open medicine* 2019; **7**: 2050312119876151.
81. Eloi M, Horvath DV, Szejnfeld VL, et al. Vitamin D deficiency and seasonal variation over the years in Sao Paulo, Brazil. *Osteoporos Int* 2016; **27**(12): 3449-56.
82. Fang F, Wei H, Wang K, et al. High prevalence of vitamin D deficiency and influencing factors among urban and rural residents in Tianjin, China. *Arch Osteoporos* 2018; **13**(1): 64.
83. Fayet-Moore F, Brock KE, Wright J, et al. Determinants of vitamin D status of healthy office workers in Sydney, Australia. *J Steroid Biochem Mol Biol* 2019; **189**: 127-34.
84. Feketea GM, Bocsan IC, Tsiros G, Voila P, Stanciu LA, Zdrenghea M. Vitamin D Status in Children in Greece and Its Relationship with Sunscreen Application. *Children (Basel, Switzerland)* 2021; **8**(2).
85. Feng X, Guo T, Wang Y, et al. The vitamin D status and its effects on life quality among the elderly in Jinan, China. *Arch Gerontol Geriatr* 2016; **62**: 26-9.
86. Fernandez Bustillo JM, Fernandez Pombo A, Gomez Bahamonde R, Sanmartin Lopez E, Gualillo O. Vitamin D levels in a pediatric population of a primary care centre: a public health problem? *BMC Res Notes* 2018; **11**(1): 801.
87. Flores ME, Rivera-Pasquel M, Valdez-Sanchez A, et al. Vitamin D status in Mexican children 1 to 11 years of age: an update from the Ensanut 2018-19. *Salud Publica Mex* 2021; **63**(3 May-Jun): 382-93.
88. Ganmaa D, Holick MF, Rich-Edwards JW, et al. Vitamin D deficiency in reproductive age Mongolian women: a cross sectional study. *J Steroid Biochem Mol Biol* 2014; **139**: 1-6.
89. Gariballa S, Yasin J, Abluwi G, Al Essa A. Vitamin D deficiency associations with metabolic, bone turnover and adverse general health markers in community free

living adults. *BMC Endocr Disord* 2022; **22**(1): 17.
90. Gebreegziabher T, Stoecker BJ. Vitamin D insufficiency in a sunshine-sufficient area: southern Ethiopia. *Food Nutr Bull* 2013; **34**(4): 429-33.
91. Gilbert-Diamond D, Baylin A, Mora-Plazas M, et al. Vitamin D deficiency and anthropometric indicators of adiposity in school-age children: a prospective study. *Am J Clin Nutr* 2010; **92**(6): 1446-51.
92. Gill TK, Hill CL, Shanahan EM, et al. Vitamin D levels in an Australian population. *BMC Public Health* 2014; **14**: 1001.
93. Ginter JK, Krithika S, Gozdzik A, Hanwell H, Whiting S, Parra EJ. Vitamin D status of older adults of diverse ancestry living in the Greater Toronto Area. *BMC Geriatr* 2013; **13**: 66.
94. Glatt DU, McSorley E, Pourshahidi LK, et al. Vitamin D Status and Health Outcomes in School Children in Northern Ireland: Year One Results from the D-VinCHI Study. *Nutrients* 2022; **14**(4).
95. Goktas O, Ersoy C, Ercan I, Can FE. Vitamin D status in the adult population of Bursa-Turkey. *Eur J Gen Pract* 2020; **26**(1): 156-62.
96. Golbahar J, Al-Saffar N, Altayab Diab D, Al-Othman S, Darwish A, Al-Kafaji G. Predictors of vitamin D deficiency and insufficiency in adult Bahrainis: a cross-sectional study. *Public Health Nutr* 2014; **17**(4): 732-8.
97. Gonzalez G, Alvarado JN, Rojas A, Navarrete C, Velasquez CG, Arteaga E. High prevalence of vitamin D deficiency in Chilean healthy postmenopausal women with normal sun exposure: additional evidence for a worldwide concern. *Menopause* 2007; **14**(3 Pt 1): 455-61.
98. Gonzalez-Gross M, Valtuena J, Breidenassel C, et al. Vitamin D status among adolescents in Europe: the Healthy Lifestyle in Europe by Nutrition in Adolescence study. *Br J Nutr* 2012; **107**(5): 755-64.
99. Gonzalez-Molero I, Morcillo S, Valdes S, et al. Vitamin D deficiency in Spain: a population-based cohort study. *Eur J Clin Nutr* 2011; **65**(3): 321-8.
100. Gordon CM, DePeter KC, Feldman HA, Grace E, Emans SJ. Prevalence of vitamin D deficiency among healthy adolescents. *Arch Pediatr Adolesc Med* 2004; **158**(6): 531-7.
101. Goswami R, Marwaha RK, Gupta N, et al. Prevalence of vitamin D deficiency and its relationship with thyroid autoimmunity in Asian Indians: a community-based survey. *Br J Nutr* 2009; **102**(3): 382-6.
102. Granlund L, Ramnemark A, Andersson C, Lindkvist M, Fharm E, Norberg M. Prevalence of vitamin D deficiency and its association with nutrition, travelling and clothing habits in an immigrant population in Northern Sweden. *Eur J Clin Nutr* 2016; **70**(3): 373-9.
103. Greene-Finestone LS, Berger C, de Groh M, et al. 25-Hydroxyvitamin D in Canadian adults: biological, environmental, and behavioral correlates. *Osteoporos Int* 2011; **22**(5): 1389-99.
104. Griffin TP, Wall D, Blake L, et al. Vitamin D Status of Adults in the Community, in Outpatient Clinics, in Hospital, and in Nursing Homes in the West of Ireland. *J Gerontol A Biol Sci Med Sci* 2020; **75**(12): 2418-25.

105. Gromova O, Doschanova A, Lokshin V, et al. Vitamin D deficiency in Kazakhstan: Cross-Sectional study. *J Steroid Biochem Mol Biol* 2020; **199**: 105565.
106. Guo S, Gies P, King K, Lucas RM. Sun exposure and vitamin D status as Northeast Asian migrants become acculturated to life in Australia. *Photochem Photobiol* 2014; **90**(6): 1455-61.
107. Han B, Wang X, Wang N, et al. Investigation of vitamin D status and its correlation with insulin resistance in a Chinese population. *Public Health Nutr* 2017; **20**(9): 1602-8.
108. Hansen L, Tjonneland A, Koster B, et al. Vitamin D Status and Seasonal Variation among Danish Children and Adults: A Descriptive Study. *Nutrients* 2018; **10**(11).
109. Harinarayan CV, Ramalakshmi T, Prasad UV, et al. High prevalence of low dietary calcium, high phytate consumption, and vitamin D deficiency in healthy south Indians. *Am J Clin Nutr* 2007; **85**(4): 1062-7.
110. Harkness LS, Cromer BA. Vitamin D deficiency in adolescent females. *J Adolesc Health* 2005; **37**(1): 75.
111. Hashemipour S, Larijani B, Adibi H, et al. Vitamin D deficiency and causative factors in the population of Tehran. *BMC Public Health* 2004; **4**: 38.
112. Hatun S, Islam O, Cizmecioglu F, et al. Subclinical vitamin D deficiency is increased in adolescent girls who wear concealing clothing. *J Nutr* 2005; **135**(2): 218-22.
113. Hazell TJ, Pham TT, Jean-Philippe S, et al. Vitamin D status is associated with bone mineral density and bone mineral content in preschool-aged children. *J Clin Densitom* 2015; **18**(1): 60-7.
114. Hekimsoy Z, Dinc G, Kafesciler S, et al. Vitamin D status among adults in the Aegean region of Turkey. *BMC Public Health* 2010; **10**: 782.
115. Herrick KA, Storandt RJ, Afful J, et al. Vitamin D status in the United States, 2011-2014. *Am J Clin Nutr* 2019; **110**(1): 150-7.
116. Hintzpeter B, Scheidt-Nave C, Muller MJ, Schenk L, Mensink GB. Higher prevalence of vitamin D deficiency is associated with immigrant background among children and adolescents in Germany. *J Nutr* 2008; **138**(8): 1482-90.
117. Hirani V. Vitamin D status and pain: analysis from the Health Survey for England among English adults aged 65 years and over. *Br J Nutr* 2012; **107**(7): 1080-4.
118. Hirani V, Cumming RG, Blyth FM, et al. Vitamin D status among older community dwelling men living in a sunny country and associations with lifestyle factors: the Concord Health and Ageing in Men Project, Sydney, Australia. *J Nutr Health Aging* 2013; **17**(7): 587-93.
119. Hoge A, Donneau AF, Streel S, et al. Vitamin D deficiency is common among adults in Wallonia (Belgium, 51 degrees 30' North): findings from the Nutrition, Environment and Cardio-Vascular Health study. *Nutr Res* 2015; **35**(8): 716-25.
120. Ho-Pham LT, Nguyen ND, Lai TQ, Eisman JA, Nguyen TV. Vitamin D status and parathyroid hormone in a urban population in Vietnam. *Osteoporos Int* 2011; **22**(1): 241-8.
121. Horton-French K, Dunlop E, Lucas RM, Pereira G, Black LJ. Prevalence and predictors of vitamin D deficiency in a nationally representative sample of

Australian adolescents and young adults. *Eur J Clin Nutr* 2021; **75**(11): 1627-36.
122. Houghton LA, Brown RC, Beaumont S, et al. Micronutrient status differs among Maasai and Kamba preschoolers in a supplementary feeding programme in Kenya. *Matern Child Nutr* 2019; **15**(3): e12805.
123. Hovsepian S, Amini M, Aminorroaya A, Amini P, Iraj B. Prevalence of vitamin D deficiency among adult population of Isfahan City, Iran. *J Health Popul Nutr* 2011; **29**(2): 149-55.
124. Hribar M, Hristov H, Gregoric M, et al. Nutrihealth Study: Seasonal Variation in Vitamin D Status Among the Slovenian Adult and Elderly Population. *Nutrients* 2020; **12**(6).
125. Hussain T, Eimal Latif AH, Malik S, et al. Vitamin D Deficiency and Associated Risk Factors in Muslim Housewives of Quetta, Pakistan: A Cross-Sectional Study. *Cureus* 2021; **13**(9): e17643.
126. Hutchings N, Babalyan V, Heijboer AC, et al. Vitamin D status in Armenian women: a stratified cross-sectional cluster analysis. *Eur J Clin Nutr* 2022; **76**(2): 220-6.
127. Ikonen H, Lumme J, Seppala J, et al. The determinants and longitudinal changes in vitamin D status in middle-age: a Northern Finland Birth Cohort 1966 study. *Eur J Nutr* 2021; **60**(8): 4541-53.
128. Isa H, Almaliki M, Alsabea A, Mohamed A. Vitamin D deficiency in healthy children in Bahrain: do gender and age matter? *East Mediterr Health J* 2020; **26**(3): 260-7.
129. Islam MZ, Lamberg-Allardt C, Karkkainen M, Outila T, Salamatullah Q, Shamim AA. Vitamin D deficiency: a concern in premenopausal Bangladeshi women of two socio-economic groups in rural and urban region. *Eur J Clin Nutr* 2002; **56**(1): 51-6.
130. Islam MZ, Shamim AA, Kemi V, et al. Vitamin D deficiency and low bone status in adult female garment factory workers in Bangladesh. *Br J Nutr* 2008; **99**(6): 1322-9.
131. Jaaskelainen T, Itkonen ST, Lundqvist A, et al. The positive impact of general vitamin D food fortification policy on vitamin D status in a representative adult Finnish population: evidence from an 11-y follow-up based on standardized 25-hydroxyvitamin D data. *Am J Clin Nutr* 2017; **105**(6): 1512-20.
132. Janssen HC, Emmelot-Vonk MH, Verhaar HJ, van der Schouw YT. Determinants of vitamin D status in healthy men and women aged 40-80 years. *Maturitas* 2013; **74**(1): 79-83.
133. Jayashri R, Venkatesan U, Shanthirani CS, et al. Prevalence of vitamin D deficiency in urban south Indians with different grades of glucose tolerance. *Br J Nutr* 2020: 1-8.
134. Jayatissa R, Lekamwasam S, Ranbanda JM, Ranasingha S, Perera AG, De Silva KH. Vitamin D deficiency among children aged 10-18 years in Sri Lanka. *Ceylon Med J* 2019; **64**(4): 146-54.
135. Jiang W, Wu DB, Xiao GB, Ding B, Chen EQ. An epidemiology survey of vitamin D deficiency and its influencing factors. *Med Clin (Barc)* 2020; **154**(1): 7-12.

136. Johnson MA, Davey A, Park S, Hausman DB, Poon LW, Georgia Centenarian S. Age, race and season predict vitamin D status in African American and white octogenarians and centenarians. *J Nutr Health Aging* 2008; **12**(10): 690-5.
137. Jolliffe DA, Hanifa Y, Witt KD, et al. Environmental and genetic determinants of vitamin D status among older adults in London, UK. *J Steroid Biochem Mol Biol* 2016; **164**: 30-5.
138. Jorde R, Sneve M, Hutchinson M, Emaus N, Figenschau Y, Grimnes G. Tracking of serum 25-hydroxyvitamin D levels during 14 years in a population-based study and during 12 months in an intervention study. *Am J Epidemiol* 2010; **171**(8): 903-8.
139. Joukar F, Naghipour M, Hassanipour S, Fakhrieh Asl S, Pourshams A, Mansour-Ghanaei F. Vitamin D deficiency associated with reproductive factors in northern Iranian women: The PERSIAN Guilan Cohort Study (PGCS). *Clin Nutr ESPEN* 2020; **38**: 271-6.
140. Junaid K, Rehman A, Jolliffe DA, Wood K, Martineau AR. High prevalence of vitamin D deficiency among women of child-bearing age in Lahore Pakistan, associating with lack of sun exposure and illiteracy. *BMC Womens Health* 2015; **15**: 83.
141. Kaddam IM, Al-Shaikh AM, Abaalkhail BA, et al. Prevalence of vitamin D deficiency and its associated factors in three regions of Saudi Arabia. *Saudi Med J* 2017; **38**(4): 381-90.
142. Kagotho E, Omuse G, Okinda N, Ojwang P. Vitamin D status in healthy black African adults at a tertiary hospital in Nairobi, Kenya: a cross sectional study. *BMC Endocr Disord* 2018; **18**(1): 70.
143. Kapil U, Pandey RM, Sharma B, et al. Prevalence of Vitamin D Deficiency in Children (6-18 years) Residing in Kullu and Kangra Districts of Himachal Pradesh, India. *Indian J Pediatr* 2018; **85**(5): 344-50.
144. Karaguzel G, Dilber B, Can G, Okten A, Deger O, Holick MF. Seasonal vitamin D status of healthy schoolchildren and predictors of low vitamin D status. *J Pediatr Gastroenterol Nutr* 2014; **58**(5): 654-60.
145. Karin Z, Gilic B, Supe Domic D, et al. Vitamin D Status and Analysis of Specific Correlates in Preschool Children: A Cross-Sectional Study in Southern Croatia. *Int J Environ Res Public Health* 2018; **15**(11).
146. Karonova T, Andreeva A, Nikitina I, et al. Prevalence of Vitamin D deficiency in the North-West region of Russia: A cross-sectional study. *J Steroid Biochem Mol Biol* 2016; **164**: 230-4.
147. Kaykhaei MA, Hashemi M, Narouie B, et al. High prevalence of vitamin D deficiency in Zahedan, southeast Iran. *Ann Nutr Metab* 2011; **58**(1): 37-41.
148. Khan AH, Iqbal R, Naureen G, Dar FJ, Ahmed FN. Prevalence of vitamin D deficiency and its correlates: results of a community-based study conducted in Karachi, Pakistan. *Arch Osteoporos* 2012; **7**: 275-82.
149. Kim SY, Jeon SW, Lim WJ, et al. Vitamin D deficiency and suicidal ideation: A cross-sectional study of 157,211 healthy adults. *J Psychosom Res* 2020; **134**: 110125.

150. Kim YS, Hwang JH, Song MR. The Association Between Vitamin D Deficiency and Metabolic Syndrome in Korean Adolescents. *J Pediatr Nurs* 2018; **38**: e7-e11.
151. Klenk J, Rapp K, Denkinger MD, et al. Seasonality of vitamin D status in older people in Southern Germany: implications for assessment. *Age Ageing* 2013; **42**(3): 404-8.
152. Kouda K, Nakamura H, Fujita Y, Ohara K, Iki M. Vitamin D status and body fat measured by dual-energy X-ray absorptiometry in a general population of Japanese children. *Nutrition* 2013; **29**(10): 1204-8.
153. Koyama S, Kubota T, Naganuma J, Arisaka O, Ozono K, Yoshihara S. Incidence rate of vitamin D deficiency and FGF23 levels in 12- to 13-year-old adolescents in Japan. *J Bone Miner Metab* 2021; **39**(3): 456-62.
154. Kremer R, Campbell PP, Reinhardt T, Gilsanz V. Vitamin D status and its relationship to body fat, final height, and peak bone mass in young women. *J Clin Endocrinol Metab* 2009; **94**(1): 67-73.
155. Kull M, Jr., Kallikorm R, Tamm A, Lember M. Seasonal variance of 25-(OH) vitamin D in the general population of Estonia, a Northern European country. *BMC Public Health* 2009; **9**: 22.
156. Kunz C, Hower J, Knoll A, Ritzenthaler KL, Lamberti T. No improvement in vitamin D status in German infants and adolescents between 2009 and 2014 despite public recommendations to increase vitamin D intake in 2012. *European Journal of Nutrition* 2018; **58**(4): 1711-22.
157. Laird E, O'Halloran AM, Carey D, et al. The Prevalence of Vitamin D Deficiency and the Determinants of 25(OH)D Concentration in Older Irish Adults: Data From The Irish Longitudinal Study on Ageing (TILDA). *J Gerontol A Biol Sci Med Sci* 2018; **73**(4): 519-25.
158. Langlois K, Greene-Finestone L, Little J, Hidiroglou N, Whiting S. Vitamin D status of Canadians as measured in the 2007 to 2009 Canadian Health Measures Survey. *Health reports* 2010; **21**(1): 47-55.
159. Lappe JM, Davies KM, Travers-Gustafson D, Heaney RP. Vitamin D status in a rural postmenopausal female population. *J Am Coll Nutr* 2006; **25**(5): 395-402.
160. Lardner E, Fitzgibbon M, Wilson S, Griffin D, Mulkerrin E. Hypovitaminosis D in a healthy female population, aged from 40 to 85 years, in the west of Ireland. *Ir J Med Sci* 2011; **180**(1): 115-9.
161. Lategan R, Van den Berg VL, Ilich JZ, Walsh CM. Vitamin D status, hypertension and body mass index in an urban black community in Mangaung, South Africa. *Afr J Prim Health Care Fam Med* 2016; **8**(1): e1-e5.
162. Le Goaziou MF, Contardo G, Dupraz C, Martin A, Laville M, Schott-Pethelaz AM. Risk factors for vitamin D deficiency in women aged 20-50 years consulting in general practice: a cross-sectional study. *Eur J Gen Pract* 2011; **17**(3): 146-52.
163. Lee J, Park HK, Kwon MJ, Ham SY, Lim SY, Song JU. Decreased lung function is associated with vitamin D deficiency in apparently health, middle aged Koreans: the Kangbuk Samsung Health Study. *Eur J Clin Nutr* 2021; **75**(3): 501-12.
164. Leung RY, Cheung BM, Nguyen US, Kung AW, Tan KC, Cheung CL. Optimal vitamin D status and its relationship with bone and mineral metabolism in Hong

Kong Chinese. *Bone* 2017; **97**: 293-8.
165. Li H, Huang T, Xiao P, et al. Widespread vitamin D deficiency and its sex-specific association with adiposity in Chinese children and adolescents. *Nutrition* 2020; **71**: 110646.
166. Li L, Li K, Li J, et al. Ethnic, geographic, and seasonal differences of vitamin D status among adults in south-west China. *J Clin Lab Anal* 2020; **34**(12): e23532.
167. Li S, Ou Y, Zhang H, et al. Vitamin D status and its relationship with body composition, bone mineral density and fracture risk in urban central south Chinese postmenopausal women. *Ann Nutr Metab* 2014; **64**(1): 13-9.
168. Lima-Costa MF, Mambrini JVM, de Souza-Junior PRB, et al. Nationwide vitamin D status in older Brazilian adults and its determinants: The Brazilian Longitudinal Study of Aging (ELSI). *Sci Rep* 2020; **10**(1): 13521.
169. Lin LY, Smeeth L, Langan S, Warren-Gash C. Distribution of vitamin D status in the UK: a cross-sectional analysis of UK Biobank. *BMJ Open* 2021; **11**(1): e038503.
170. Liu X, Baylin A, Levy PD. Vitamin D deficiency and insufficiency among US adults: prevalence, predictors and clinical implications. *Br J Nutr* 2018; **119**(8): 928-36.
171. Liu X, Ke L, Ho J, et al. Sleep duration is associated with vitamin D deficiency in older women living in Macao, China: A pilot cross-sectional study. *PLoS One* 2020; **15**(3): e0229642.
172. Lopes JB, Danilevicius CF, Takayama L, et al. Vitamin D insufficiency: a risk factor to vertebral fractures in community-dwelling elderly women. *Maturitas* 2009; **64**(4): 218-22.
173. Lucas JA, Bolland MJ, Grey AB, et al. Determinants of vitamin D status in older women living in a subtropical climate. *Osteoporos Int* 2005; **16**(12): 1641-8.
174. Madsen KH, Rasmussen LB, Mejborn H, et al. Vitamin D status and its determinants in children and adults among families in late summer in Denmark. *Br J Nutr* 2014; **112**(5): 776-84.
175. Maguire JL, Birken CS, O'Connor DL, et al. Prevalence and predictors of low vitamin D concentrations in urban Canadian toddlers. *Paediatr Child Health* 2011; **16**(2): e11-5.
176. Middelkoop K, Walker N, Stewart J, et al. Prevalence and Determinants of Vitamin D Deficiency in 1825 Cape Town Primary Schoolchildren: A Cross-Sectional Study. *Nutrients*. 2022;**14**(6):1263.
177. Majumdar V, Nagaraja D, Christopher R. Vitamin D status and metabolic syndrome in Asian Indians. *Int J Obes (Lond)* 2011; **35**(8): 1131-4.
178. Maldonado G, Paredes C, Guerrero R, Rios C. Determination of Vitamin D Status in a Population of Ecuadorian Subjects. *ScientificWorldJournal* 2017; **2017**: 3831275.
179. Mallah EM, Hamad MF, Elmanaseer MA, et al. Plasma concentrations of 25-hydroxyvitamin D among Jordanians: Effect of biological and habitual factors on vitamin D status. *BMC Clin Pathol* 2011; **11**: 8.
180. Man PW, Lin W, van der Meer IM, et al. Vitamin D status in the Chinese

population in the Netherlands: The DRAGON study. *J Steroid Biochem Mol Biol* 2016; **164**: 194-8.

181. Manios Y, Moschonis G, Hulshof T, et al. Prevalence of vitamin D deficiency and insufficiency among schoolchildren in Greece: the role of sex, degree of urbanisation and seasonality. *Br J Nutr* 2017; **118**(7): 550-8.
182. Mansbach JM, Ginde AA, Camargo CA, Jr. Serum 25-hydroxyvitamin D levels among US children aged 1 to 11 years: do children need more vitamin D? *Pediatrics* 2009; **124**(5): 1404-10.
183. Masoud MS, Yakout SM, Al-Attas OS, Alokail MS, Al-Daghri NM. The association between iron and vitamin D status in Arab adolescents. *Public Health Nutr* 2020; **23**(7): 1208-13.
184. Penrose K, Hunter Adams J, Nguyen T, Cochran J, Geltman PL. Vitamin D deficiency among newly resettled refugees in Massachusetts. *J Immigr Minor Health* 2012; **14**(6): 941-8.
185. Mathei C, Van Pottelbergh G, Vaes B, Adriaensen W, Gruson D, Degryse JM. No relation between vitamin D status and physical performance in the oldest old: results from the Belfrail study. *Age Ageing* 2013; **42**(2): 186-90.
186. Mechenro J, Venugopal G, Buvnesh Kumar M, Balakrishnan D, Ramakrishna BS. Vitamin D status in Kancheepuram District, Tamil Nadu, India. *BMC Public Health* 2018; **18**(1): 1345.
187. Meddeb N, Sahli H, Chahed M, et al. Vitamin D deficiency in Tunisia. *Osteoporos Int* 2005; **16**(2): 180-3.
188. Mehboobali N, Iqbal SP, Iqbal MP. High prevalence of vitamin D deficiency and insufficiency in a low income peri-urban community in Karachi. *J Pak Med Assoc* 2015; **65**(9): 946-49.
189. Metwally ASM, Yakout SM, Khattak MNK, Alkhaldi G, Al-Daghri NM. Vitamin D Status and Its Association with Multiple Intelligence among Arab Adolescents. *Int J Environ Res Public Health* 2021; **18**(24).
190. Meyer HE, Falch JA, Sogaard AJ, Haug E. Vitamin D deficiency and secondary hyperparathyroidism and the association with bone mineral density in persons with Pakistani and Norwegian background living in Oslo, Norway, The Oslo Health Study. *Bone* 2004; **35**(2): 412-7.
191. Meyer HE, Holvik K, Lofthus CM, Tennakoon SU. Vitamin D status in Sri Lankans living in Sri Lanka and Norway. *Br J Nutr* 2008; **99**(5): 941-4.
192. Miljkovic I, Bodnar LM, Cauley JA, et al. Low prevalence of vitamin D deficiency in elderly Afro-Caribbean men. *Ethn Dis* 2011; **21**(1): 79-84.
193. Misra P, Srivastava R, Misra A, Kant S, Kardam P, Vikram NK. Vitamin D status of adult females residing in Ballabgarh health and demographic surveillance system: A community-based study. *Indian J Public Health* 2017; **61**(3): 194-8.
194. Mitchell DM, Henao MP, Finkelstein JS, Burnett-Bowie SA. Prevalence and predictors of vitamin D deficiency in healthy adults. *Endocr Pract* 2012; **18**(6): 914-23.
195. Mogire RM, Morovat A, Muriuki JM, et al. Prevalence and predictors of vitamin D deficiency in young African children. *BMC Med* 2021; **19**(1): 115.

196. Moreno-Reyes R, Carpentier YA, Boelaert M, et al. Vitamin D deficiency and hyperparathyroidism in relation to ethnicity: a cross-sectional survey in healthy adults. *Eur J Nutr* 2009; **48**(1): 31-7.
197. Moussavi M, Heidarpour R, Aminorroaya A, Pournaghshband Z, Amini M. Prevalence of vitamin D deficiency in Isfahani high school students in 2004. *Horm Res* 2005; **64**(3): 144-8.
198. Moy FM. Vitamin D status and its associated factors of free living Malay adults in a tropical country, Malaysia. *J Photochem Photobiol B* 2011; **104**(3): 444-8.
199. Moy FM, Hoe VC, Hairi NN, Vethakkan SR, Bulgiba A. Vitamin D deficiency and depression among women from an urban community in a tropical country. *Public Health Nutr* 2017; **20**(10): 1844-50.
200. Muhairi SJ, Mehairi AE, Khouri AA, et al. Vitamin D deficiency among healthy adolescents in Al Ain, United Arab Emirates. *BMC Public Health* 2013; **13**: 33.
201. Mutua AM, Nampijja M, Elliott AM, et al. Vitamin D Status Is Not Associated with Cognitive or Motor Function in Pre-School Ugandan Children. *Nutrients* 2020; **12**(6).
202. Nadeem S, Munim TF, Hussain HF, Hussain DF. Determinants of Vitamin D deficiency in asymptomatic healthy young medical students. *Pak J Med Sci* 2018; **34**(5): 1248-52.
203. Naeem Z, Almohaimeed A, Sharaf FK, Ismail H, Shaukat F, Inam SB. Vitamin D status among population of Qassim Region, Saudi Arabia. *Int J Health Sci (Qassim)* 2011; **5**(2): 116-24.
204. Nakamura K, Tsugawa N, Saito T, et al. Vitamin D status, bone mass, and bone metabolism in home-dwelling postmenopausal Japanese women: Yokogoshi Study. *Bone* 2008; **42**(2): 271-7.
205. Nakhaee S, Ali Yaghoubi M, Zarban A, et al. Vitamin D deficiency and its associated risk factors in normal adult population of Birjand, Iran. *Clin Nutr ESPEN* 2019; **32**: 113-7.
206. Nalsen C, Becker W, Pearson M, et al. Vitamin D status in children and adults in Sweden: dietary intake and 25-hydroxyvitamin D concentrations in children aged 10-12 years and adults aged 18-80 years. *J Nutr Sci* 2020; **9**: e47.
207. Naqvi A, Solomons NW, Campos R, et al. Vitamin D status among indigenous Mayan (Kekchi) and Afro-Caribe (Garifuna) adolescents from Guatemala: a comparative description between two ethnic groups residing on the Rio Dulce at the Caribbean coast in Izabal Province, Guatemala. *Public Health Nutr* 2017; **20**(10): 1729-37.
208. Alessi D, Borre S, Barale A, et al. [Seroprevalence of anti-SARS-CoV-2 IgG/IgM antibodies in Borgosesia (Piedmont Region, Northern Italy) population: a surveillance strategy in post-lockdown period?]. *Epidemiol Prev* 2020; **44**(5-6 Suppl 2): 200-6.
209. Ni Chaoimh C, McCarthy EK, Hourihane JO, et al. Low vitamin D deficiency in Irish toddlers despite northerly latitude and a high prevalence of inadequate intakes. *Eur J Nutr* 2018; **57**(2): 783-94.
210. Niafar M, Bahrami A, Aliasgharzadeh A, Aghamohammadzadeh N, Najafipour F,

Mobasseri M. Vitamin D status in healthy postmenopausal Iranian women. *J Res Med Sci* 2009; **14**(3): 171-7.

211. Nichols EK, Khatib IM, Aburto NJ, et al. Vitamin D status and associated factors of deficiency among Jordanian children of preschool age. *Eur J Clin Nutr* 2015; **69**(1): 90-5.
212. Nielsen NO, Jorgensen ME, Friis H, et al. Decrease in vitamin D status in the Greenlandic adult population from 1987-2010. *PLoS One* 2014; **9**(12): e112949.
213. Nikooyeh B, Abdollahi Z, Hajifaraji M, et al. Vitamin D status and cardiometabolic risk factors across latitudinal gradient in Iranian adults: National food and nutrition surveillance. *Nutr Health* 2017; **23**(2): 87-94.
214. Nimitphong H, Chailurkit LO, Chanprasertyothin S, Sritara P, Ongphiphadhanakul B. The Association of vitamin D status and fasting glucose according to body fat mass in young healthy Thais. *BMC Endocr Disord* 2013; **13**: 60.
215. Oberg J, Jorde R, Almas B, Emaus N, Grimnes G. Vitamin D deficiency and lifestyle risk factors in a Norwegian adolescent population. *Scand J Public Health* 2014; **42**(7): 593-602.
216. Oliveri B, Plantalech L, Bagur A, et al. High prevalence of vitamin D insufficiency in healthy elderly people living at home in Argentina. *Eur J Clin Nutr* 2004; **58**(2): 337-42.
217. Orces CH. Vitamin D Status among Older Adults Residing in the Littoral and Andes Mountains in Ecuador. *ScientificWorldJournal* 2015; **2015**: 545297.
218. Orwoll E, Nielson CM, Marshall LM, et al. Vitamin D deficiency in older men. *J Clin Endocrinol Metab* 2009; **94**(4): 1214-22.
219. Ozturk ZA, Gol M, Turkbeyler IH. Prevalence of vitamin D deficiency in otherwise healthy individuals between the ages of 18 and 90 years in southeast Turkey. *Wien Klin Wochenschr* 2017; **129**(21-22): 854-5.
220. Pan T, Banerjee R, Dasgupta A, Paul B. Vitamin D status among women aged 40 years and above in a rural area of West Bengal: A community-based study. *Journal of family medicine and primary care* 2018; **7**(6): 1263-7.
221. Patel JV, Chackathayil J, Hughes EA, Webster C, Lip GY, Gill PS. Vitamin D deficiency amongst minority ethnic groups in the UK: a cross sectional study. *Int J Cardiol* 2013; **167**(5): 2172-6.
222. Paul TV, Thomas N, Seshadri MS, Oommen R, Jose A, Mahendri NV. Prevalence of osteoporosis in ambulatory postmenopausal women from a semiurban region in Southern India: relationship to calcium nutrition and vitamin D status. *Endocr Pract* 2008; **14**(6): 665-71.
223. Perez-Llamas F, Lopez-Contreras MJ, Blanco MJ, Lopez-Azorin F, Zamora S, Moreiras O. Seemingly paradoxical seasonal influences on vitamin D status in nursing-home elderly people from a Mediterranean area. *Nutrition* 2008; **24**(5): 414-20.
224. Perna L, Haug U, Schottker B, et al. Public health implications of standardized 25-hydroxyvitamin D levels: a decrease in the prevalence of vitamin D deficiency among older women in Germany. *Prev Med* 2012; **55**(3): 228-32.
225. Peters BS, dos Santos LC, Fisberg M, Wood RJ, Martini LA. Prevalence of vitamin

D insufficiency in Brazilian adolescents. *Ann Nutr Metab* 2009; **54**(1): 15-21.
226. Petrenya N, Lamberg-Allardt C, Melhus M, Broderstad AR, Brustad M. Vitamin D status in a multi-ethnic population of northern Norway: the SAMINOR 2 Clinical Survey. *Public Health Nutr* 2020; **23**(7): 1186-200.
227. Qorbani M, Heidari-Beni M, Ejtahed HS, et al. Association of vitamin D status and cardio-metabolic risk factors in children and adolescents: the CASPIAN-V study. *BMC Nutr* 2021; **7**(1): 71.
228. Rabenberg M, Scheidt-Nave C, Busch MA, Rieckmann N, Hintzpeter B, Mensink GB. Vitamin D status among adults in Germany--results from the German Health Interview and Examination Survey for Adults (DEGS1). *BMC Public Health* 2015; **15**: 641.
229. Rabufetti A, Milani GP, Lava SAG, et al. Vitamin D Status Among Male Late Adolescents Living in Southern Switzerland: Role of Body Composition and Lifestyle. *Nutrients* 2019; **11**(11).
230. Rafraf M, Hasanabad SK, Jafarabadi MA. Vitamin D status and its relationship with metabolic syndrome risk factors among adolescent girls in Boukan, Iran. *Public Health Nutr* 2014; **17**(4): 803-9.
231. Rahmadhani R, Zaharan NL, Mohamed Z, Moy FM, Jalaludin MY. The associations between VDR BsmI polymorphisms and risk of vitamin D deficiency, obesity and insulin resistance in adolescents residing in a tropical country. *PLoS One* 2017; **12**(6): e0178695.
232. Rahman A, Al-Taiar A, Shaban L, Al-Sabah R, Mojiminiyi O. The routine chemiluminescence assay for plasma 25-hydroxyvitamin D analysis does not overestimate the prevalence of vitamin D deficiency in adolescents. *Nutr Res* 2020; **79**: 60-7.
233. Ramakrishnan S, Bhansali A, Bhadada SK, et al. Vitamin D status and its seasonal variability in healthy young adults in an Asian Indian urban population. *Endocr Pract* 2011; **17**(2): 185-91.
234. Raposo L, Martins S, Ferreira D, Guimaraes JT, Santos AC. Vitamin D, parathyroid hormone and metabolic syndrome - the PORMETS study. *BMC Endocr Disord* 2017; **17**(1): 71.
235. Riverin B, Dewailly E, Cote S, Johnson-Down L, Morin S, Dodin S. Prevalence of vitamin D insufficiency and associated factors among Canadian Cree: a cross-sectional study. *Can J Public Health* 2013; **104**(4): e291-7.
236. Riverin B, Dewailly E, Cote S, Johnson-Down L, Morin S, Dodin S. Prevalence of vitamin D insufficiency among healthy school-age Cree children. *Paediatr Child Health* 2014; **19**(3): e15-9.
237. Robinson PJ, Bell RJ, Lanzafame A, et al. The prevalence of vitamin D deficiency and relationship with fracture risk in older women presenting in Australian general practice. *Australas J Ageing* 2013; **32**(3): 177-83.
238. Rodriguez-Rodriguez E, Aparicio A, Lopez-Sobaler AM, Ortega RM. Vitamin D status in a group of Spanish schoolchildren. *Minerva Pediatr* 2011; **63**(1): 11-8.
239. Saeed BQ, Jairoun AA, Ashraf Khamis A, et al. Vitamin D Deficiency and Insufficiency Among University Students: Prevalence, Risk Factors, and the

Association Between Vitamin D Deficiency and Episodes of Respiratory Tract Infections. *Risk Manag Healthc Policy* 2021; **14**: 2733-41.
240. Saki F, Dabbaghmanesh MH, Omrani GR, Bakhshayeshkaram M. Vitamin D deficiency and its associated risk factors in children and adolescents in southern Iran. *Public Health Nutr* 2017; **20**(10): 1851-6.
241. Sakyi SA, Antwi MH, Ahenkorah Fondjo L, et al. Vitamin D Deficiency Is Common in Ghana despite Abundance of Sunlight: A Multicentre Comparative Cross-Sectional Study. *J Nutr Metab* 2021; **2021**: 9987141.
242. Saliba W, Rennert HS, Kershenbaum A, Rennert G. Serum 25(OH)D concentrations in sunny Israel. *Osteoporos Int* 2012; **23**(2): 687-94.
243. Samefors M, Ostgren CJ, Molstad S, Lannering C, Midlov P, Tengblad A. Vitamin D deficiency in elderly people in Swedish nursing homes is associated with increased mortality. *Eur J Endocrinol* 2014; **170**(5): 667-75.
244. Santos A, Amaral TF, Guerra RS, et al. Vitamin D status and associated factors among Portuguese older adults: results from the Nutrition UP 65 cross-sectional study. *BMJ Open* 2017; **7**(6): e016123.
245. Santos BR, Mascarenhas LP, Satler F, Boguszewski MC, Spritzer PM. Vitamin D deficiency in girls from South Brazil: a cross-sectional study on prevalence and association with vitamin D receptor gene variants. *BMC Pediatr* 2012; **12**: 62.
246. Santos BR, Costa NC, Silva TR, et al. Prevalence of vitamin D deficiency in women from southern Brazil and association with vitamin D-binding protein levels and GC-DBP gene polymorphisms. *PLoS One* 2019; **14**(12): e0226215.
247. Sarafin K, Durazo-Arvizu R, Tian L, et al. Standardizing 25-hydroxyvitamin D values from the Canadian Health Measures Survey. *Am J Clin Nutr* 2015; **102**(5): 1044-50.
248. Saraiva GL, Cendoroglo MS, Ramos LR, et al. Influence of ultraviolet radiation on the production of 25 hydroxyvitamin D in the elderly population in the city of Sao Paulo (23 degrees 34'S), Brazil. *Osteoporos Int* 2005; **16**(12): 1649-54.
249. Scalco R, Premaor MO, Froehlich PE, Furlanetto TW. High prevalence of hypovitaminosis D and secondary hyperparathyroidism in elders living in nonprofit homes in South Brazil. *Endocrine* 2008; **33**(1): 95-100.
250. Schramm S, Lahner H, Jockel KH, et al. Impact of season and different vitamin D thresholds on prevalence of vitamin D deficiency in epidemiological cohorts-a note of caution. *Endocrine* 2017; **56**(3): 658-66.
251. Science M, Maguire JL, Russell ML, Smieja M, Walter SD, Loeb M. Prevalence and predictors of low serum 25-hydroxyvitamin D levels in rural Canadian children. *Paediatr Child Health* 2017; **22**(3): 125-9.
252. Seo JA, Eun CR, Cho H, et al. Low vitamin D status is associated with nonalcoholic Fatty liver disease independent of visceral obesity in Korean adults. *PLoS One* 2013; **8**(10): e75197.
253. Abu Shady MM, Youssef MM, Shehata MA, Salah El-Din EM, ElMalt HA. Association of Serum 25-Hydroxyvitamin D with Life Style and Dietary Factors in Egyptian Prepubescent Children. *Open Access Macedonian Journal of Medical Sciences* 2015; **3**(1): 80-4.

254. Sharawat IK, Dawman L. Bone mineral density and its correlation with vitamin D status in healthy school-going children of Western India. *Arch Osteoporos* 2019; **14**(1): 13.
255. Shchubelka K. Vitamin D status in adults and children in Transcarpathia, Ukraine in 2019. *BMC Nutr* 2020; **6**(1): 48.
256. Sheikh A, Saeed Z, Jafri SA, Yazdani I, Hussain SA. Vitamin D levels in asymptomatic adults--a population survey in Karachi, Pakistan. *PLoS One* 2012; **7**(3): e33452.
257. Sherchand O, Sapkota N, Chaudhari RK, et al. Association between vitamin D deficiency and depression in Nepalese population. *Psychiatry Res* 2018; **267**: 266-71.
258. Sherief LM, Ali A, Gaballa A, et al. Vitamin D status and healthy Egyptian adolescents: Where do we stand? *Medicine (Baltimore)* 2021; **100**(29): e26661.
259. Shetty S, Kapoor N, Naik D, et al. Osteoporosis in healthy South Indian males and the influence of life style factors and vitamin d status on bone mineral density. *J Osteoporos* 2014; **2014**: 723238.
260. Shivane VK, Sarathi V, Bandgar T, Menon P, Shah NS. High prevalence of hypovitaminosis D in young healthy adults from the western part of India. *Postgrad Med J* 2011; **87**(1030): 514-8.
261. Sioen I, Mouratidou T, Kaufman JM, et al. Determinants of vitamin D status in young children: results from the Belgian arm of the IDEFICS (Identification and Prevention of Dietary- and Lifestyle-Induced Health Effects in Children and Infants) Study. *Public Health Nutr* 2012; **15**(6): 1093-9.
262. Skull SA, Ngeow JY, Biggs BA, Street A, Ebeling PR. Vitamin D deficiency is common and unrecognized among recently arrived adult immigrants from The Horn of Africa. *Intern Med J* 2003; **33**(1-2): 47-51.
263. Smith G, Wimalawansa SJ, Laillou A, et al. High Prevalence of Vitamin D Deficiency in Cambodian Women: A Common Deficiency in a Sunny Country. *Nutrients* 2016; **8**(5).
264. Smith N, Sievert LL, Muttukrishna S, et al. Mismatch: a comparative study of vitamin D status in British-Bangladeshi migrants. *Evol Med Public Health* 2021; **9**(1): 164-73.
265. Sochorova L, Hanzlikova L, Cerna M, et al. Assessment of vitamin D status in Czech children. *Cent Eur J Public Health* 2018; **26**(4): 260-4.
266. Sokolovic S, Alimanovic-Alagic R, Dzananovic L, Cavaljuga S, Beslic N, Ferhatbegovic-Opankovic E. Vitamin D status in Bosnia and Herzegovina: the cross-sectional epidemiological analysis. *Osteoporos Int* 2017; **28**(3): 1021-5.
267. Solis-Urra P, Cristi-Montero C, Romero-Parra J, Zavala-Crichton JP, Saez-Lara MJ, Plaza-Diaz J. Passive Commuting and Higher Sedentary Time Is Associated with Vitamin D Deficiency in Adult and Older Women: Results from Chilean National Health Survey 2016(-)2017. *Nutrients* 2019; **11**(2).
268. Song HR, Kweon SS, Choi JS, et al. High prevalence of vitamin D deficiency in adults aged 50 years and older in Gwangju, Korea: the Dong-gu Study. *J Korean Med Sci* 2014; **29**(1): 149-52.

269. Souberbielle JC, Massart C, Brailly-Tabard S, Cavalier E, Chanson P. Prevalence and determinants of vitamin D deficiency in healthy French adults: the VARIETE study. *Endocrine* 2016; **53**(2): 543-50.
270. Srimani S, Saha I, Chaudhuri D. Prevalence and association of metabolic syndrome and vitamin D deficiency among postmenopausal women in a rural block of West Bengal, India. *PLoS One* 2017; **12**(11): e0188331.
271. Sulimani RA, Mohammed AG, Alfadda AA, et al. Vitamin D deficiency and biochemical variations among urban Saudi adolescent girls according to season. *Saudi Med J* 2016; **37**(9): 1002-8.
272. Suryanarayana P, Arlappa N, Sai Santhosh V, et al. Prevalence of vitamin D deficiency and its associated factors among the urban elderly population in Hyderabad metropolitan city, South India. *Ann Hum Biol* 2018; **45**(2): 133-9.
273. Tangoh DA, Apinjoh TO, Mahmood Y, et al. Vitamin D Status and Its Associated Risk Factors among Adults in the Southwest Region of Cameroon. *J Nutr Metab* 2018; **2018**: 4742574.
274. Ten Haaf DSM, Balvers MGJ, Timmers S, Eijsvogels TMH, Hopman MTE, Klein Gunnewiek JMT. Determinants of vitamin D status in physically active elderly in the Netherlands. *Eur J Nutr* 2019; **58**(8): 3121-8.
275. Thuesen B, Husemoen L, Fenger M, et al. Determinants of vitamin D status in a general population of Danish adults. *Bone* 2012; **50**(3): 605-10.
276. Tolppanen AM, Fraser A, Fraser WD, Lawlor DA. Risk factors for variation in 25-hydroxyvitamin D(3) and D(2) concentrations and vitamin D deficiency in children. *J Clin Endocrinol Metab* 2012; **97**(4): 1202-10.
277. Tonnesen R, Hovind PH, Jensen LT, Schwarz P. Determinants of vitamin D status in young adults: influence of lifestyle, sociodemographic and anthropometric factors. *BMC Public Health* 2016; **16**: 385.
278. Tran B, Armstrong BK, McGeechan K, et al. Predicting vitamin D deficiency in older Australian adults. *Clin Endocrinol (Oxf)* 2013; **79**(5): 631-40.
279. Tseng M, Giri V, Bruner DW, Giovannucci E. Prevalence and correlates of vitamin D status in African American men. *BMC Public Health* 2009; **9**: 191.
280. Unger MD, Cuppari L, Titan SM, et al. Vitamin D status in a sunny country: where has the sun gone? *Clin Nutr* 2010; **29**(6): 784-8.
281. Uush T. Prevalence of classic signs and symptoms of rickets and vitamin D deficiency in Mongolian children and women. *J Steroid Biochem Mol Biol* 2013; **136**: 207-10.
282. Vallejo MS, Blumel JE, Arteaga E, et al. Gender differences in the prevalence of vitamin D deficiency in a southern Latin American country: a pilot study. *Climacteric* 2020; **23**(4): 410-6.
283. Vallianou N, Bountziouka V, Akalestos T, et al. Vitamin D status and health correlates among apparently healthy participants in an urban, sunny region. *Cent Eur J Public Health* 2012; **20**(4): 262-9.
284. Vasudevan B, Karunakaran U, Antony A, Ramachandran R. Vitamin D status and associated factors among peri menopausal women in two selected districts of Kerala. *Indian J Public Health* 2021; **65**(2): 166-71.

285. Vierucci F, Del Pistoia M, Fanos M, Erba P, Saggese G. Prevalence of hypovitaminosis D and predictors of vitamin D status in Italian healthy adolescents. *Ital J Pediatr* 2014; **40**: 54.
286. von Hurst PR, Stonehouse W, Coad J. Vitamin D status and attitudes towards sun exposure in South Asian women living in Auckland, New Zealand. *Public Health Nutr* 2010; **13**(4): 531-6.
287. Voortman T, van den Hooven EH, Heijboer AC, Hofman A, Jaddoe VW, Franco OH. Vitamin D deficiency in school-age children is associated with sociodemographic and lifestyle factors. *J Nutr* 2015; **145**(4): 791-8.
288. Vupputuri MR, Goswami R, Gupta N, Ray D, Tandon N, Kumar N. Prevalence and functional significance of 25-hydroxyvitamin D deficiency and vitamin D receptor gene polymorphisms in Asian Indians. *Am J Clin Nutr* 2006; **83**(6): 1411-9.
289. Wakayo T, Belachew T, Vatanparast H, Whiting SJ. Vitamin D deficiency and its predictors in a country with thirteen months of sunshine: the case of school children in central Ethiopia. *PLoS One* 2015; **10**(3): e0120963.
290. Ward M, Berry DJ, Power C, Hypponen E. Working patterns and vitamin D status in mid-life: a cross-sectional study of the 1958 British birth cohort. *Occupational and Environmental Medicine* 2011; **68**(12): 902-7.
291. White Z, White S, Dalvie T, Kruger MC, Van Zyl A, Becker P. Bone Health, Body Composition, and Vitamin D Status of Black Preadolescent Children in South Africa. *Nutrients* 2019; **11**(6).
292. Wyskida M, Owczarek A, Szybalska A, et al. Socio-economic determinants of vitamin D deficiency in the older Polish population: results from the PolSenior study. *Public Health Nutr* 2018; **21**(11): 1995-2003.
293. Yan X, Zhang N, Cheng S, Wang Z, Qin Y. Gender Differences in Vitamin D Status in China. *Med Sci Monit* 2019; **25**: 7094-9.
294. Yang K, Liu J, Fu S, et al. Vitamin D Status and Correlation with Glucose and Lipid Metabolism in Gansu Province, China. *Diabetes Metab Syndr Obes* 2020; **13**: 1555-63.
295. Yousef S, Manuel D, Colman I, et al. Vitamin D Status among First-Generation Immigrants from Different Ethnic Groups and Origins: An Observational Study Using the Canadian Health Measures Survey. *Nutrients* 2021; **13**(8).
296. Yu L, Ke HJ, Che D, Luo SL, Guo Y, Wu JL. Effect of Pandemic-Related Confinement on Vitamin D Status Among Children Aged 0-6 Years in Guangzhou, China: A Cross-Sectional Study. *Risk Manag Healthc Policy* 2020; **13**: 2669-75.
297. Yu S, Fang H, Han J, et al. The high prevalence of hypovitaminosis D in China: a multicenter vitamin D status survey. *Medicine (Baltimore)* 2015; **94**(8): e585.
298. Zargar AH, Ahmad S, Masoodi SR, et al. Vitamin D status in apparently healthy adults in Kashmir Valley of Indian subcontinent. *Postgrad Med J* 2007; **83**(985): 713-6.
299. Zgaga L, Theodoratou E, Farrington SM, et al. Diet, environmental factors, and lifestyle underlie the high prevalence of vitamin D deficiency in healthy adults in Scotland, and supplementation reduces the proportion that are severely deficient.

J Nutr 2011; **141**(8): 1535-42.
300. Zhang FF, Al Hooti S, Al Zenki S, et al. Vitamin D deficiency is associated with high prevalence of diabetes in Kuwaiti adults: results from a national survey. *BMC Public Health* 2016; **16**: 100.
301. Zhao Y, Zhao W, Hao Q, et al. Vitamin D status and obesity markers in older adults: results from West China Health and Aging Trends study. *BMC Geriatr* 2021; **21**(1): 528.
302. Zhen D, Liu L, Guan C, Zhao N, Tang X. High prevalence of vitamin D deficiency among middle-aged and elderly individuals in northwestern China: its relationship to osteoporosis and lifestyle factors. *Bone* 2015; **71**: 1-6.
303. Zhou SJ, Skeaff M, Makrides M, Gibson R. Vitamin D status and its predictors among pre-school children in Adelaide. *J Paediatr Child Health* 2015; **51**(6): 614-9.
304.Zhu W, Heil DP. Associations of vitamin D status with markers of metabolic health: A community-based study in Shanghai, China. *Diabetes Metab Syndr* 2018; **12**(5): 727-32.
305. Zhu Z, Zhan J, Shao J, et al. High prevalence of vitamin D deficiency among children aged 1 month to 16 years in Hangzhou, China. *BMC Public Health* 2012; **12**: 126.
306. Chakrabarty S. Prevalence and Covariates of Vitamin D Deficiencies (VDD) among Adolescents in India. *Indian J Pediatr*. 2022;**89**(8):751-758.
307. Marzban M, Kalantarhormozi M, Mahmudpour M, et al. Prevalence of vitamin D deficiency and its associated risk factors among rural population of the northern part of the Persian Gulf. *BMC Endocr Disord*. 2021;**21**(1):219.
308. Lin L, Ou Q, Lin L, et al. Low prevalence of vitamin D deficiency in adult residents in Hainan, the tropical island province of China. *Ann Palliat Med*. 2021;**10**(5):5580-5589.
